# Chemoenzymatic modular assembly of O-GalNAc glycans for functional glycomics

**DOI:** 10.1038/s41467-021-23428-x

**Published:** 2021-06-11

**Authors:** Shuaishuai Wang, Congcong Chen, Madhusudhan Reddy Gadi, Varma Saikam, Ding Liu, He Zhu, Roni Bollag, Kebin Liu, Xi Chen, Fengshan Wang, Peng George Wang, Peixue Ling, Wanyi Guan, Lei Li

**Affiliations:** 1 Department of Chemistry, Georgia State University, Atlanta, GA 30303 USA; 2 National Glycoengineering Research Center, Shandong Provincial Key Laboratory of Glycochemistry and Glycobiology, Shandong University, Qingdao, 266237 Shandong China; 3 Shandong Academy of Pharmaceutical Science, Key Laboratory of Biopharmaceuticals, Engineering Laboratory of Polysaccharide Drugs, National-Local Joint Engineering Laboratory of Polysaccharide Drugs, Jinan, 250101 Shandong China; 4 Georgia Cancer Center, Augusta University, Augusta, GA 30912 USA; 5 Department of Biochemistry and Molecular Biology, Medical College of Georgia, Augusta, GA 30912 USA; 6 Department of Chemistry, University of California, Davis, CA 95616 USA; 7 Key Laboratory of Chemical Biology (Ministry of Education), Institute of Biochemical and Biotechnological Drug, School of Pharmaceutical Science, Shandong University, Jinan, 250012 Shandong China; 8 College of Life Science, Hebei Normal University, Shijiazhuang, 050024 Hebei China; 9 Present Address: School of Medicine, Southern University of Science and Technology, Shenzhen, 518055 Guangdong China

**Keywords:** Carbohydrates, Glycobiology, Diversity-oriented synthesis

## Abstract

O-GalNAc glycans (or mucin O-glycans) play pivotal roles in diverse biological and pathological processes, including tumor growth and progression. Structurally defined O-GalNAc glycans are essential for functional studies but synthetic challenges and their inherent structural diversity and complexity have limited access to these compounds. Herein, we report an efficient and robust chemoenzymatic modular assembly (CEMA) strategy to construct structurally diverse O-GalNAc glycans. The key to this strategy is the convergent assembly of O-GalNAc cores 1–4 and 6 from three chemical building blocks, followed by enzymatic diversification of the cores by 13 well-tailored enzyme modules. A total of 83 O-GalNAc glycans presenting various natural glycan epitopes are obtained and used to generate a unique synthetic mucin O-glycan microarray. Binding specificities of glycan-binding proteins (GBPs) including plant lectins and selected anti-glycan antibodies towards these O-GalNAc glycans are revealed by this microarray, promoting their applicability in functional O-glycomics. Serum samples from colorectal cancer patients and healthy controls are assayed using the array reveal higher bindings towards less common cores 3, 4, and 6 than abundant cores 1 and 2, providing insights into O-GalNAc glycan structure-activity relationships.

## Abstract (web summary)

O-GalNAc glycans are essential in many biological and pathological processes, but difficult to access due to their structural complexity and synthetic challenges. Here, the authors report an efficient chemoenzymatic modular assembly strategy to construct structurally diverse O-GalNAc glycans, use the synthesised glycans to generate a synthetic mucin O-glycan microarray and profile binding specificities of glycan-binding proteins.

## Introduction

O-GalNAc glycans (also known as mucin O-glycans) represent a major component of the mammalian glycocalyx and are involved in various biological processes via glycan-protein interactions^1^. All O-GalNAc glycans share a common structural feature containing the monosaccharide *N*-acetylgalactosamine (GalNAc) α-linked to the hydroxyl group of serine (Ser) or threonine (Thr) residues in proteins^2^. O-GalNAc glycosylation decorates over 80% of secretory and cell surface proteins^3^. It confers many critical biological functions ranging from structural roles to immune responses and cell-cell interactions. For example, mucin is a major part of the mucosal barrier on gastrointestinal, respiratory, reproductive, and urinary tracts that protects humans from pathogens and aids clearance of microbes^4^. O-GalNAc glycans on mucins influence the configuration and exposure of peptide epitopes and the adhesive properties of glycoproteins^1^. On the other hand, pathogens could harness cell surface glycans as entry receptors to initiate infection^5^, or produce surface glycans mimicking host O-GalNAc glycans to escape from the host immune surveillance^6^. Aberrant O-GalNAc glycosylation is correlated with tumor growth and metastasis by modulating tumor cell–matrix interactions^7–9^. As a result, some O-GalNAc glycans and glycopeptides have been used and explored as cancer biomarkers for clinical diagnosis and as antigenic targets for the development of therapeutic antibodies^10,11^.

**Fig. 1:**
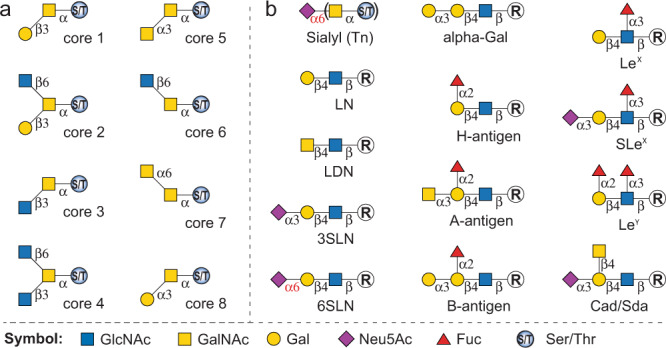
Structures of O-GalNAc glycan cores and their common glycan epitope extensions. **a** O-GalNAc glycan cores 1–8. **b** Common glycan epitopes found on O-GalNAc glycans. LN, *N*-acetyllactosamine (LacNAc); LDN, *N*,*N’*-diacetyllactosamine (LacdiNAc); 3SLN, 3’-sialyl LacNAc; 6SLN, 6’-sialyl LacNAc; Le^X^, Lewis X; SLe^X^, sialyl-Lewis X; Le^Y^, Lewis Y. Abbreviations: Gal, galactose; Fuc, L-fucose; GlcNAc, *N*-acetylglucosamine; GalNAc, *N*-acetylgalactosamine; Neu5Ac, *N*-acetylneuraminic acid; Ser, serine; Thr, threonine.

O-GalNAc glycans are structurally more diverse than N-glycans. A single GalNAc residue α-linked to Ser/Thr forms the Tn antigen (GalNAcα-Ser/Thr), which is often α2-6 sialylated, providing the sialyl-Tn antigen (Siaα2-6GalNAcα-Ser/Thr). Tn can also be extended to generate four major O-GalNAc cores (cores 1–4) and four rare cores (cores 5–8). As shown in Fig. 1, the attachment of a galactose (Gal) residue to the Tn antigen via a β1-3 linkage generates core 1, also named T antigen. Core 2 is formed by adding an *N*-acetylglucosamine (GlcNAc) residue to the GalNAc of core 1 via a β1-6 linkage. Cores 1 and 2 glycans are the most common O-GalNAc structures and nearly ubiquitously expressed on mucins and other glycoproteins. Cores 3, 4, and 6 are β-GlcNAcylated on C3-hydroxyl (C3-OH) and/or C6-OH of the initiating GalNAc, whereas cores 5, 7, and 8 contain α-linked extensions (α1-3GalNAc, α1-6GalNAc, and α1-3Gal, respectively) (Fig. 1a). Cores 3 and 4 are less common than cores 1 and 2, and are more restricted to glycoproteins in bronchial and gastrointestinal tissues^1^. Cores 5–8 are linear structures with low occurrence and abundance. The diversity and complexity of O-GalNAc glycans stem from multiple cores and additional glycosylation presenting different epitopes. Sialylations are observed on all types of cores. Common epitopes identified on cores 1–4 and 6 include ABO blood group antigens (A-, B-, and H-antigen), Lewis antigens (e.g., Lewis X, Lewis Y, and sialyl-Lewis X antigens), *N*-acetyllactosamine (LacNAc, LN), *N*,*N*′-diacetyllactosamine (LDN), 3′-sialyl LacNAc (3SLN), 6′-sialyl LacNAc (6SLN), alpha-Gal, and Cad/Sd^a^ (Fig. 1b)^1,10,12,13^.

The access to structurally diverse O-GalNAc glycans in sufficient quantity and purity is essential to their structure-function relationship studies. In the last decade, shotgun glycomics^14,15^, oxidative release^16^, O-glycome beam search^17^, and preparative cellular O-glycome reporter/amplification^18^ have been developed to obtain O-GalNAc glycans from natural sources. However, these methods do not allow access to complex O-GalNAc glycans in sufficient quantity, especially for those with rare cores and low abundant glycoforms. Chemical and chemoenzymatic methods have thus been used to obtain a variety of sialylated O-GalNAc glycans^19–23^, including cores 1–4 and 6 that contain poly-LacNAc motifs^24,25^. In addition, solid-phase approaches have been developed for synthesizing O-GalNAc glycopeptides, but only those with simple core structures or their sialylated forms were normally obtained^26–29^. A strategy for the systematic preparation of O-GalNAc glycans with varied natural epitopes is still missing, and the lack of these structures had been a major barrier to functional O-glycomic studies.

Herein, we describe the implementation of a chemoenzymatic modular assembly (CEMA) strategy that deploys three synthetic building blocks and 13 enzyme modules in a precisely controlled manner for the rapid access of structurally diverse O-GalNAc glycans. The method yields a collection of 83 O-GalNAc glycans presenting important glycan epitopes on cores 1–4 and 6. The attached Ser or Thr enables the easy attachment of O-glycans to glass surfaces for the fabrication of a unique O-GalNAc glycan microarray, which is a useful tool to investigate the glycan-binding specificity of glycan binding proteins (GBPs) and probe anti-mucin antibodies.

## Results and discussion

### The chemoenzymatic modular assembly (CEMA) strategy

**Fig. 2:**
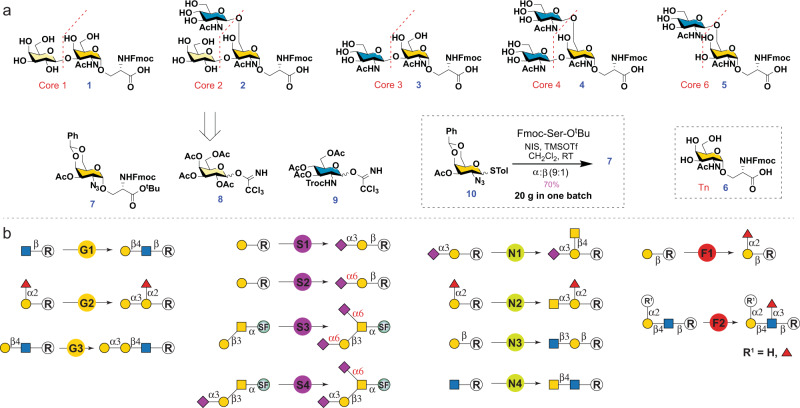
Retrosynthetic analysis of O-GalNAc glycans. **a** Retrosynthetic analysis of O-GalNAc cores with three synthetic building blocks. **b** Retrosynthetic analysis of O-GalNAc epitopes with 13 enzyme modules. Enzyme modules: G stands for galactosylation, S stands for sialylation, N stands for *N*-acetylhexosaminylation, F stands for fucosylation. G1: β1-4 galactosylation with *Neisseria meningitidis* β1-4 galactosyltransferase (NmLgtB)^47^ and donor uridine 5′-diphosphogalactose (UDP-Gal); G2: α1–3 galactosylation with human GTB and UDP-Gal; G3: α1-3 galactosylation with bovine α1-3 GalT (Bα3GalT)^48^ and UDP-Gal; S1: α2-3 sialylation with *Pasteurella multocida* α2-3 sialyltransferase 1 mutant M144D (PmST1-M144D)^52^, *N. meningitidis* CMP-sialic acid synthetase (NmCSS)^69^, cytidine 5′-triphosphate (CTP), and *N*-acetylneuraminic acid (Neu5Ac); S2: α2-6 sialylation with PmST1-P34H/M144L^49^, NmCSS, CTP, and Neu5Ac; S3: α2–6 sialylation with *Photobacterium damselae* α2–6 sialyltransferase (Pd2,6ST)^50^, NmCSS, CTP, and Neu5Ac; S4: α2-6 sialylation with human ST6GalNAc-IV, NmCSS, CTP, and Neu5Ac; N1: β1-4 *N*-acetylgalactosaminylation with *Campylobacter jejuni* β1-4 *N*-acetylgalatosaminyltransferase (CjCgtA)^70^ and uridine 5′-diphosphate-*N*-acetylgalactosamine (UDP-GalNAc); N2: α1-3 *N*-acetylgalactosaminylation with *Helicobacter mustelae* α1-3 *N*-acetyl-galactosaminyltransferase (HmBgtA)^56^ and UDP-GalNAc; N3: β1-3 *N*-acetylglucosaminylation with *Helicobacter pylori* β1-3 *N*-acetylglucosaminyltransferase (HpLgtA)^71^ and uridine 5’-diphosphate-*N*-acetylglucosamine (UDP-GlcNAc); N4: β1-4 *N*-acetylgalactosaminylation with b4GalT-Y289L/C342T (b4GalTm)^72^ and UDP-GalNAc; F1: α1-2 fucosylation with *H. mustelae* α1-2 fucosyltransferase (Hm2FT)^55^ and guanosine 5′-diphospho-L-fucose (GDP-Fuc); F2: α1-3 fucosylation with *H. pylori* α1-3 fucosyltransferase C-terminal 66 amino acid truncation (Hp3FT)^73^ and GDP-Fuc. SF, Fmoc protected Ser.

The CEMA strategy includes (1) diversity-oriented and scalable chemical assembly of O-GalNAc glycan cores, and (2) highly efficient enzyme modules to glycosylate the cores with precise control on regio- and stereoselectivity. All O-GalNAc cores are branched from either the C3-OH or/and the C6-OH of the initiating GalNAc (Fig. 1a). Thus, a suitable Tn antigen selectively protected at C3/6-OH to allow late-stage selective deprotection is the key for assembling the cores. To access cores 1–4 and 6 (**1**–**5**, Fig. 2a), we designed three synthetic building blocks, including a versatile protected glycosyl amino acid **7** with C3-OH protected by an acetyl (Ac) group and C4/6-OH masked by a benzylidene acetal, and two monosaccharide Schmidt donors **8**^30^ and **9**^31^. The Fmoc group is introduced to the Ser residue of **7** to facilitate reaction monitoring and product purification^32^. The Ac group on **7** would be selectively removed under mild basic conditions. Subsequent branching at the free C3-OH by glycosylation with **8** and **9** would produce core 1 and 3, respectively. In addition, deprotection of benzylidene acetal in the resulting disaccharides to expose C4-OH and C6-OH followed by regioselective glycosylation at the more active C6-OH with *N*-Troc-protected module **9** will yield cores 2 and 4, respectively. On the other hand, core 6 could be obtained by selective deprotection of C6-OH of **7** followed by the regioselective glycosylation with **9**.

Stereoselective synthesis of Tn antigen with an α-linked GalNAc remains a major challenging step for mucin O-glycan synthesis^33,34^. High α-selectivity is usually achieved by using a glycosyl donor with a non-participating group at C2, such as an azide group^35^. Extensive efforts have also been made on evaluating structural modification of sugar donors with various protective groups^36–39^, leaving groups^40^, and reaction conditions^41,42^. For the purpose of preparing structurally diverse O-GalNAc glycans, a simple, efficient, and diversity-oriented route encompassing a fluorescent core (e.g., Fmoc) would be more advantageous^32^. A stable thio glycosyl donor **10**^43^ was thus selected and obtained by efficient synthetic methods^44^. Ser with a Fmoc-protected amino group and a *tert*-butyl ester protected carboxyl group (Fmoc-Ser-O^t^Bu) was selected as the glycosyl acceptor^45^. Glycosylation of the Fmoc-Ser-O^t^Bu with the glycosyl donor **10** at room temperature promoted by NIS and TMSOTf produced **7**^41,46^ with a satisfying yield of 70% and a good stereoselectivity of 9:1 (α:β) (Fig. 2a). Importantly, the presence of benzylidene acetal allowed easy separation of the anomers on TLC (R_f_: α-0.7, β-0.4, 50% ethyl acetate/hexanes) and efficient purification by silica gel flash column chromatography. The synthesis was easily scaled up to obtain 20 grams of **7** in one batch.

While chemical approaches are powerful in synthesizing simple cores for up to gram scales, enzyme-catalyzed reactions are advantageous in preparing large complex glycans owing to their unique regio- and stereoselectivity. To assemble common epitopes (Fig. 1b) on O-GalNAc cores 1–4 and 6, a total of 13 enzyme modules were employed (Fig. 2b), including three galactosylation modules (G1–G3), four sialylation modules (S1–S4), four *N*-acetylhexosaminylation modules (N1–N4), and two fucosylation modules (F1 and F2). Enzymes used in these modules are well characterized and robust glycosyltransferases (GTs). They are specific to generate only the desired epitopes. For example, *Neisseria meningitidis* β1-4 galactosyltransferase (NmLgtB) is used for β1-4 galactosylation (module G1)^47^. Human GTB (module G2) and bovine α1-3 GalT (Bα3GalT)^48^ (module G3) are employed for α1-3 galactosylation to generate B-antigen and alpha-Gal respectively, according to their acceptor specificities. For α2-6 sialylation, three enzyme modules are proposed: *Pasteurella multocida* α2-3 sialyltransferase 1 mutant P34H/M144L (PmST1-P34H/M144L) that is highly selective for sialylating non-reducing terminal Gal residues (S2)^49^, *Photobacterium damselae* α2-6 sialyltransferase (Pd2,6ST) that is highly active and recognizes all terminal and internal Gal and GalNAc (S3)^50^, and human ST6GalNAc-IV that only recognizes the initiating GalNAc residue (S4)^51^. All enzyme modules are well-tailored for specific acceptors according to their substrate specificities to avoid side reactions and achieve precise control for the synthesis of desired glycans.

### Chemical modular assembly of O-GalNAc cores 1–4 and 6

**Fig. 3:**
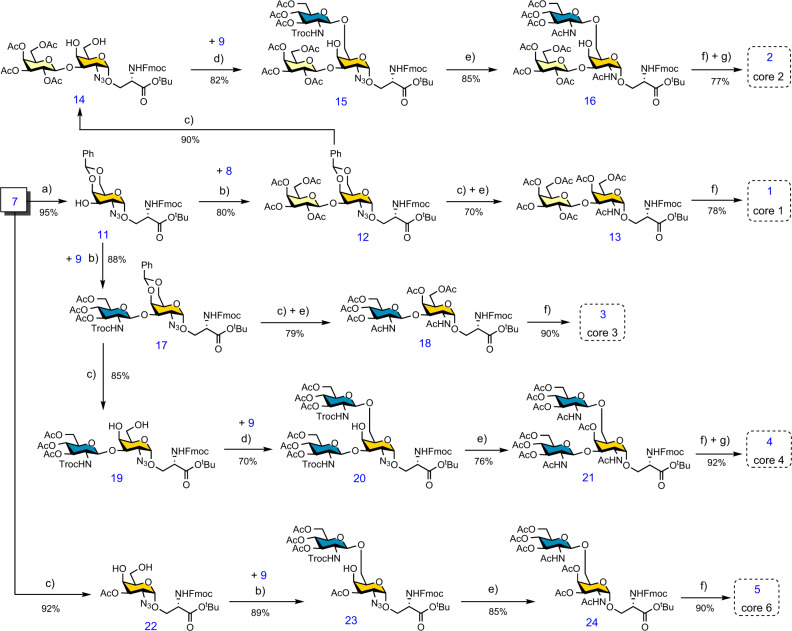
Chemical modular assembly of O-GalNAc cores 1–4 and 6 structures. Reagents and conditions: **a** NaOMe, MeOH. **b** TMSOTf, CH_2_Cl_2_, −78 °C. **c** *p*-toluenesulfonic acid, MeOH. **d** TMSOTf, CH_2_Cl_2_, -40 °C. **e** i. Zn, AcOH, CH_2_Cl_2_; ii. Py, Ac_2_O. **f** i. TFA, CH_2_Cl_2_; ii. NaOMe, MeOH. **g** FmocOSu, NaHCO_3_.

Module **7** serves as an advanced intermediate that can be extended properly at C3 or/and C6 positions to obtain all O-GalNAc core structures (Fig. 3). To assemble core 1, the O-Ac group at C3-OH was deprotected under basic conditions to yield compound **11**. The Schmidt donor module **8** was then used to glycosylate **11** in the presence of catalytic TMSOTf to provide the β1-3-linked disaccharide **12**. Subsequent deprotection of benzylidene acetal, azide reduction, and acetylation without any intermediate purification produced the protected core 1 (**13**). Finally, *tert*-butyl and Ac groups were removed under strong acidic and mild basic conditions successively to obtain Fmoc-protected core 1 (**1**). It is worth noting to avoid the loss of Fmoc, the pH of the solution should not exceed 8.5 during the final Ac ester deprotection step. To synthesize core 2, the benzylidene acetal on **12** was removed to obtain diol **14**. Regioselective installation of *N*-Troc protected module **9**^31^ to the C6-OH of **14** using TMSOTf as a promoter resulted in the β-linked trisaccharide **15** exclusively. Successive deprotection of the *N*-Troc group, reduction of azide, and *N*-acetylation resulted in the protected core 2 trisaccharide **16**. Final deprotection of the *tert*-butyl and Ac esters produced core 2 (**2**). However, despite maintaining the pH of the reaction below 8.5 during Zemplén deprotection, nearly 50% of Fmoc was lost. Fmoc was therefore quantitavely reintroduced under basic conditions using Fmoc *N*-hydroxysuccinimide ester before product purification^32^. Core 2 (**2**) was obtained with a yield of 77% over the last three steps.

Cores 3, 4, and 6 are GlcNAc extended structures of Tn antigen at C3 and/or C6 positions, respectively. As illustrated in Fig. 3, to obtain core 3, glycosyl acceptor **11** was glycosylated with **9** in the presence of catalytic TMSOTf to obtain exclusively β-linked disaccharide **17**. Subsequent deprotection of benzylidene acetal followed by one-pot Zn-mediated reduction of azide and deprotection of *N*-Troc formed a disaccharide with a free amino group at C-2, which was acetylated without purification to obtain **18**. The ^t^Bu and Ac esters were then deprotected to obtain core 3 (**3**). On the other hand, diol **19** obtained by benzylidene deprotection of **17** was glycosylated with **9** at the C6 position under Lewis acid conditions to provide **20** in a yield of 70%. The protected derivative **20** was converted to the peracetylated compound **21** which was deprotected followed by the re-introduction of Fmoc to form core 4 (**4**) similar to that described above for the synthesis of core 2. To access core 6, benzylidene acetal was firstly removed to obtain **22** followed by regioselective glycosylation with **9** at C6, which provided β-linked disaccharide **23** exclusively. Subsequent *N*-Troc deprotection, azide reduction, and peracetylation yielded compound **24**, which was converted to core 6 (**5**) by *tert*-butyl and Ac ester deprotection. The overall yields of cores 1–4 and 6 starting from module **7** are decent, ranging from 35% to 63%. Lastly, the Tn antigen (**6**) was converted from the common intermediate **10** by azide reduction and global deprotection using contemporary chemical routes as described for the synthesis of core 1 (Supplementary Information).

### Enzymatic modular assembly of O-GalNAc glycans

With chemically synthesized Tn antigen, cores 1–4, and core 6 in hand, structurally diverse O-GalNAc glycans were prepared using 13 enzyme modules (Fig. 2b) in a well-designed sequential manner. Specifically, the Ser-linked STn antigen (**25**) was prepared from **6** by enzyme module S3. Briefly, Tn antigen (**6**) was incubated in 100 mM Tris-HCl (pH 8.0) with the highly active Pd2,6ST, *N. meningitidis* CMP-sialic acid synthetase (NmCSS), cytidine 5’-triphosphate (CTP), *N*-acetylneuraminic acid (Neu5Ac), and MgCl_2_. NmCSS enabled in situ generation of the sialyltransferase sugar nucleotide donor CMP-Neu5Ac. The reaction was carried out at 37 °C for 3 h and stopped by boiling for 5 min. After brief centrifugation, the supernatant was concentrated and purified by reverse-phase (RP)-HPLC (Supplementary Information) to afford the STn antigen (**25**). The bulky hydrophobic UV-detectable fluorescent Fmoc group facilitates real-time monitoring of reaction processes and makes the product separation by RP-HPLC easier^32^.

**Fig. 4:**
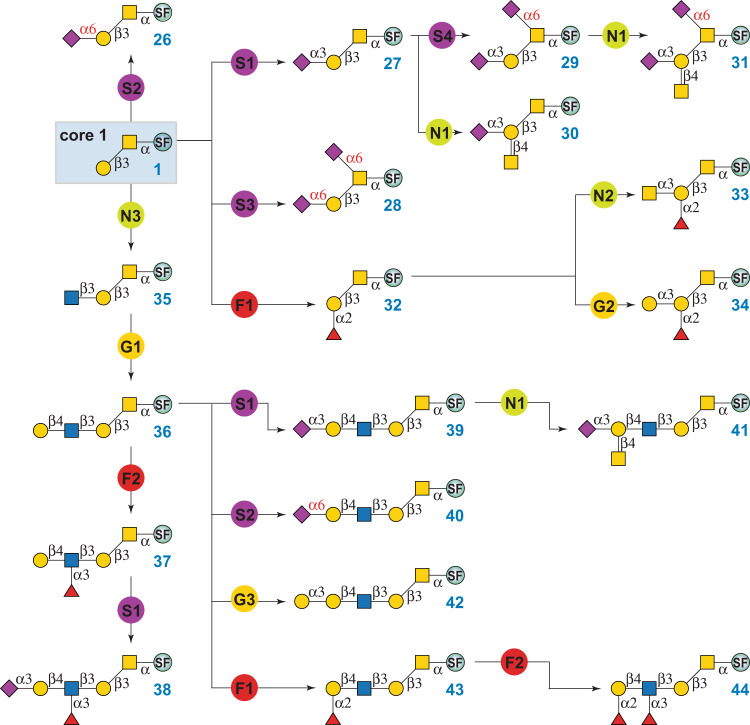
Enzymatic modular synthesis of core 1 O-GalNAc glycans. G1: β1-4 galactosylation with NmLgtB and UDP-Gal; G2: α1-3 galactosylation with human GTB and UDP-Gal; G3: α1-3 galactosylation with Bα3GalT and UDP-Gal; S1: α2-3 sialylation with PmST1-M144D, NmCSS, CTP, and Neu5Ac; S2: α2-6 sialylation with PmST1-P34H/M144L, NmCSS, CTP, and Neu5Ac; S3: α2-6 sialylation with Pd2,6ST, NmCSS, CTP, and Neu5Ac; S4: α2-6 sialylation with human ST6GalNAc-IV, NmCSS, CTP, and Neu5Ac; N1: β1-4 *N*-acetylgalactosaminylation with CjCgtA and UDP-GalNAc; N2: α1-3 *N*-acetylgalactosaminylation with HmBgtA and UDP-GalNAc; N3: β1-3 *N*-acetylglucosaminylation with HpLgtA and UDP-GlcNAc; F1: α1-2 fucosylation with Hm2FT and GDP-Fuc; F2: α1-3 fucosylation with Hp3FT and GDP-Fuc. SF, Fmoc protected Ser.

Core 1 O-GalNAc glycans are commonly found on mucins and other glycoproteins. Most core 1 structures are sialylated. As depicted in Fig. 4, enzymatic modular assembly starting from **1** yielded 19 extended core 1 structures, including 9 sialylated ones. Mono-sialylated glycans **26** and **27** were prepared by enzyme modules S2 and S1, respectively. As mentioned above, PmST1-P34H/M144L in module S2 is an engineered regioselective α2-6 sialyltransferase^49^, whereas PmST1-M144D in S1 is a regioselective α2-3 sialyltransferase^52^. Both enzymes are highly selective for sialylating non-reducing end Gal residues. Core 1 with two α2-6-linked sialic acids (**28**) was prepared by using module S3, which contains Pd2,6ST, a highly active α2-6 sialyltransferase with substrate promiscuity that recognizes both terminal and internal Gal/GalNAc residues. Given that Pd2,6ST could sialylate Gal even in the presence of α2-3 sialyation^53^, the synthesis of di-sialylated **29** Neu5Acα2-3Galβ1-3(Neu5Acα2-6)GalNAcα-FmocSer was achieved via another α2-6 sialyation module S4, in which human ST6GalNAc-IV catalyzed regioselective α2-6 sialylation of the initiating GalNAc^51^. O-GalNAc glycans **30** and **31** that presenting the Cad/Sd^a^ antigen were assembled by module N1 from **27** and **29**, respectively. Enzyme module N1 contained *C. jejuni* β1-4-*N*-acetylgalactosaminyltransferase (CjCgtA), which catalyzed the transfer of GalNAc onto the Gal residue of the Siaα2-3Gal motif^54^.

Additionally, core 1 with blood group H-antigen (**32**) was prepared by incubating **1** with enzyme module F1 that contained *H. mustelae* α1,2-fucosyltransferase (Hm2FT)^55^ and the sugar nucleotide GDP-Fuc. We found that Hm2FT not only recognized lactose^55^, but also tolerated LacNAc and Galβ1-3GalNAc. It was used to synthesize O-GalNAc glycans bearing Type II and III H-antigens (e.g., **43** and **32**) in excellent yields. Glycans with blood group A- or B-antigens were subsequently prepared by *H. mustelae* α1,3-*N*-acetylgalactosamanyltransferase (HmBgtA) (module N2) and human GTB (module G2), respectively. Both enzymes have broad acceptor specificities towards all five subtypes of H-antigens^56,57^.

Similarly, extended core 1 structures presenting LacNAc (**36**), Le^X^ (**37**), SLe^X^ (**38**), 3SLN (**39**), 6SLN (**40**), Cad/Sd^a^ (**41**), alpha-Gal (**42**), H-antigen (**43**), and Le^Y^ (**44**) epitopes were prepared by the stepwise enzymatic modular assembly (Fig. 4). The assembly route was designed according to the acceptor specificity of each glycosyltransferase to avoid undesired side reactions. To further eliminate undesired side products, glycan products were purified by RP-HPLC to homogeneity (>98%) before subjected to the next step. All purified glycans were characterized by analytical HPLC to confirm purity, and NMR and mass spectrometry to confirm structures (Supplementary Information). Please note that instead of multi-enzyme systems as that for in situ generation of CMP-Neu5Ac, partially purified (P2-chromatography to >70%) sugar donors UPD-Gal, UDP-GalNAc, UDP-GlcNAc, and GDP-Fuc were used in modules G1–G3, N1–N4, and F1–F2, to reduce incubation times, increase yields, and simplify the HPLC purification process.

**Fig. 5:**
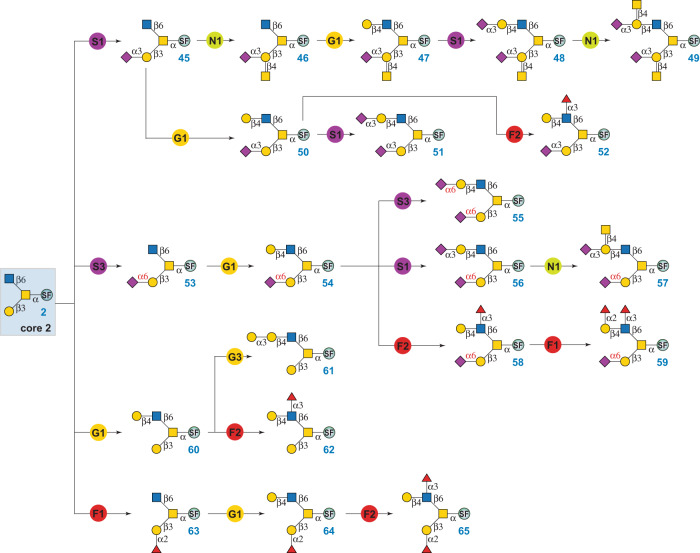
Enzymatic modular synthesis of core 2 O-GalNAc glycans. G1: β1-4 galactosylation with NmLgtB and UDP-Gal; G3: α1-3 galactosylation with Bα3GalT and UDP-Gal; S1: α2-3 sialylation with PmST1-M144D, NmCSS, CTP, and Neu5Ac; S3: α2-6 sialylation with Pd2,6ST, NmCSS, CTP, and Neu5Ac; N1: β1-4 *N*-acetylgalactosaminylation with CjCgtA and UDP-GalNAc; F1: α1-2 fucosylation with Hm2FT and GDP-Fuc; F2: α1-3 fucosylation with Hp3FT and GDP-Fuc. SF, Fmoc protected Ser.

Core 2 O-glycans are branched structures where the β1-3Gal branch is commonly sialylated or attached with the Cad/Sd^a^ antigen and the β1-6GlcNAc branch presents varied epitopes^12^. Starting from **2**, a total of 21 core 2 structures were prepared via a sequential modular assembly of the β1-3Gal branch and then the β1-6GlcNAc branch (Fig. 5). For example, to prepare core 2 glycans with Cad/Sd^a^ antigen at the β1-3Gal branch (compounds **46**–**49**), α2-3 sialylation of core 2 (**2**) was firstly performed using module S1 to form **45**. Subsequent treatment of **45** by module N1 (CjCgtA and UDP-GalNAc) formed the β1-3Gal branch in the fully assembled compound **46**. Sequential glycosylations of **46** at the β1-6GlcNAc branch by enzyme modules G1, S1, and N1 yielded **47**–**49**, which were previously identified in mammalian cells by CORA and β-elimination methods^12,58^. In addition, the β1-6GlcNAc branch of **45** was directly extended by modules G1, S1, and F2, respectively, to produce glycans **50**–**52**. In parallel, core 2 structures with α2-6 sialylation at the β1-3Gal branch (compounds **53**–**59**) were assembled in a similar sequential manner (Fig. 5). It is worth pointing out that the synthesis of compound **59** from **54** can also be achieved by Hm2FT-catalyzed α1,2 fucosylation (module F1) followed by Hp3FT-catalyzed α1,3-fucosylation (module F2), as both fucosyltransferases have relatively relaxed acceptor specificities and can tolerate both non-fucosylated and mono-fucosylated acceptor substrates^59^. Non-sialylated core 2 O-glycans have also been found in mammalian cells. For example, compounds **60**–**62**, **64**, and **65** were previously identified from several cell lines including primary cells^12^. These structures were synthesized from **2** via a sequential modular assembly shown in Fig. 5. Specifically, the alpha-Gal epitope-presenting glycan **61** was obtained via direct α1-3 galactosylation of the β1-6GlcNAc branch in **60** by module G3. G3 contains bovine α1,3-galactosyltransferase (Bα3GalT) that can use both LacNAc and Lac disaccharides as acceptor substrates^48^.

After validating the application of the CEMA strategy in the synthesis of cores 1 and 2 glycans, we turned our attention to synthesize low abundant O-GalNAc glycans. As shown in Supplementary Figures 1-3, fourteen core 3-type glycans (**66**–**79**), ten core 4-type glycans (**80**–**89**), and twelve core 6-type glycans (**90**–**101**) were synthesized. Collectively, 83 Ser-linked O-GalNAc glycans (Table 1, Supplementary Figure 4) were prepared through the robust CEMA strategy. All products were purified by RP-HPLC and characterized by NMR and mass spectrometry. Such glyco-amino acids can be applied, with or without protection by peracetylation, for solid-phase peptide synthesis^60^.

**Table 1:** Ser-linked O-GalNAc glycans prepared in this study.

### Microarray assays to probe the specificity of glycan-binding proteins (GBPs)

**Fig. 6:**
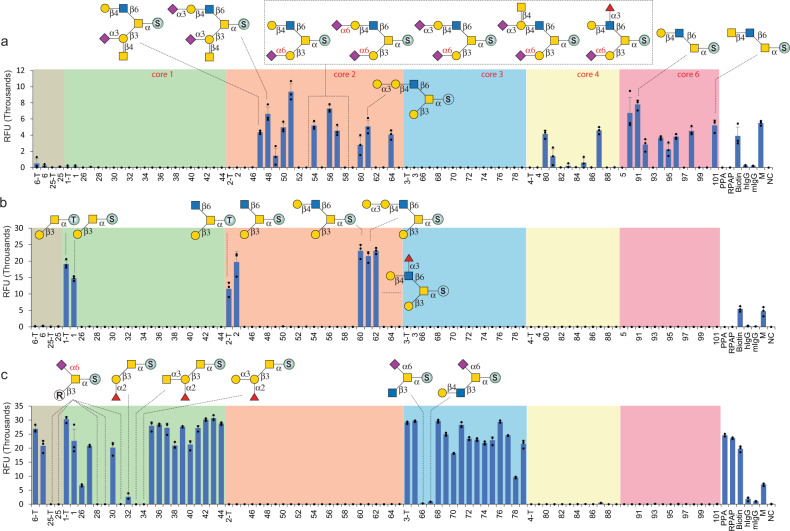
Binding profiles of lectins towards O-GalNAc glycans. **a** The binding profile of STL towards O-GalNAc glycans. **b** The binding profile of PNA towards O-GalNAc glycans. **c** The binding profile of Jacalin towards O-GalNAc glycans. The *x*-axis shows glycans, and the *y*-axis shows relative fluorescence readout using Cy5-streptavidin (1 μg/mL). PPA = APGS(GalNAcα-)TAPP (100 µM); RPAP = TSAPD(GalNAcα-)TRPAP (100 µM); x-T, Thr-linked counterparts of Ser-linked O-glycans; Biotin = biotinylated PEG amine (0.01 mg/mL); hIgG = human IgG (0.1 mg/mL), mIgG = mouse IgG (0.1 mg/mL); M = Marker (0.01 mg/mL Cy3-conjugated anti-Human IgG + 0.01 mg/mL Alexs647-conjugated anti-Human IgG); NC = printing buffer negative control. *n* = 3 independent replicates. The individual data points are shown as dots. Data are presented as mean values. Error bars represent standard deviation. Source data are provided as a Source Data file.

Microarrays represent a major tool for functional glycomics studies. While various glycan microarrays have been developed during the last two decades, O-glycan structures in these arrays are still limited^17,61^. To explore the utility of the O-GalNAc-glycans synthesized, we prepared a synthetic glycan microarray according to the MIRAGE guidelines (Supplementary Table 1) by immobilizing Fmoc-deprotected glycans **1**–**6** and **25**–**101** on *N*-hydroxysuccinimide (NHS)-activated glass slides. We assayed 17 commonly used GBPs and 2 recombinant influenza hemagglutinin (HA) proteins (Supplementary Table 2). The GBPs include fucose-binding lectins (AAL, UEA-I, LTL), sialic acid-binding lectins (MAL-I, SNA), LacNAc or GlcNAc-binding lectins (RCA-I, ECL, GSL-II, STL), Tn antigen-binding lectins (SBA, VVL, DBA), T antigen-binding lectins (PNA, Jacalin), and antibodies that are specifically against sLe^X^, STn, and MUC1 (Fig. 6, Supplementary Figs. 5–10).

The glycan microarray results confirmed successful spotting of the glycans and revealed fine specificities of GBPs tested toward O-GalNAc glycans. For example, the chitin-binding *Solanum tuberosum* lectin (STL)^62^ showed moderate binding to core 2 and 6 glycans presenting a terminal LacNAc motif (with/without further modifications) but not to core 1 and 3 structures (Fig. 6a), indicating its surprisingly strict preference toward the LacNAc on the β1-6GlcNAc branch. Such specificity may be applied to distinguish O-GalNAc core structures in heterogeneous mixtures. The T antigen-targeting *Arachis hypogaea* (peanut) lectin (PNA) and Jacalin were used as efficient tools for cancer diagnosis/prognosis and O-glycopeptide capturing^63^. We found that PNA selectively bound to T antigen (**1**) and core 2 glycans with an unmodified β1-3Gal branch (**2**, **60**–**62**) (Fig. 6b), suggesting the requirement of a free Gal attached to the initiating GalNAc for proper recognition^64^. The results also showed that Jacalin bound strongly to all core 3 and nearly all core 1 glycans (devoid of α2-6 sialylation) at the same level of the Tn antigen, but did not bind to any core 2, 4, or 6 structures (Fig. 6c), suggesting a strict requirement for the free C6-OH on the initial GalNAc^65^. Binding specificities and fine details of all tested lectins are summarized in Supplementary Table 3. We then compared our lectin binding results with those obtained from the Consortium for Functional Glycomics (CFG) glycan microarray (Supplementary Table 4 summarizes O-GalNAc glycan structures in the array) (https://ncfg.hms.harvard.edu/ncfg-data/microarray-data/lectin-quality-assurancequality-control). The binding profiles of STL, PNA, VVL, SBA, and AAL to O-GalNAc glycans on the CFG array (Supplementary Figure 11) were similar to our results, further confirmed the successful fabrication of our synthetic O-GalNAc-glycan array.

**Fig. 7:**
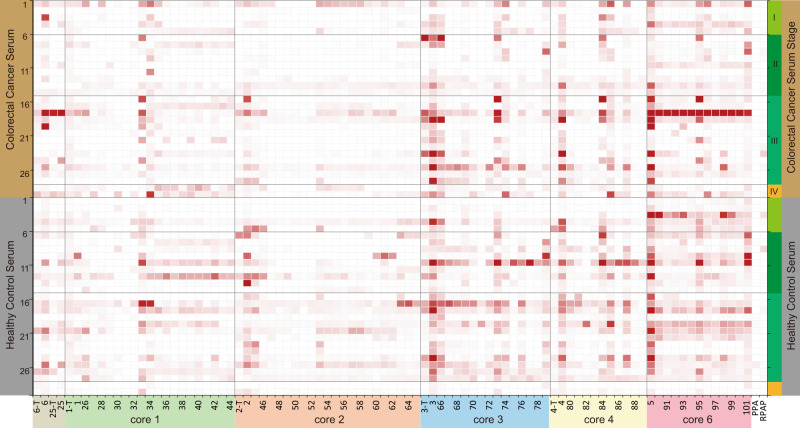
Heatmap of lgM bindings on the O-GalNAc glycan microarray in sera from colorectal cancer patients and healthy control people. PPA = APGS(GalNAcα-)TAPP (100 µM); RPAP = TSAPD(GalNAcα-)TRPAP (100 µM); x-T, Thr-linked counterparts of Ser-linked O-glycans; Biotin = biotinylated PEG amine (0.01 mg/mL); NC = printing buffer negative control. *n* = 3 independent replicates. Data are presented as mean values. Source data are provided as a Source Data file.

Altered expression levels of some serum anti-glycan antibodies have been linked to many diseases including cancer^66–68^, which are presumably the consequences of aberrant expression of the corresponding glycans including O-GalNAc glycans^7–9^. The synthetic O-GalNAc glycan microarray can be used to analyze the changes of anti-O-GalNAc glycan antibodies (IgG and IgM) in the sera of cancer patients and can serve as a promising platform for cancer biomarker discovery. To explore this opportunity, 29 serum samples from patients with colorectal cancer (Supplementary Table 5) and 29 from healthy controls were analyzed. The sera were diluted 50-fold and the antibodies bound to glycans on the array were probed with DyLight 650-conjugated anti-human IgG Fc antibody and DyLight 550-conjugated anti-human IgM antibody. For IgG, other than those bound strongly to O-glycans presenting A- or B-antigen (**33**, **34**, **73**, **74**, **84**, **85**, **95**, **96**) which are most likely the natural anti-A and anti-B antibodies, no apparent specific binding was observed (Supplementary Fig. 12). On the other hand, the overall IgM binding signals were high and varied significantly among individual serum samples (Fig. 7). Serum samples from both colorectal cancer patients and healthy controls showed higher overall IgM binding toward low abundant cores 3, 4, and 6 (compounds **3**, **4**, **5**) than to highly abundant cores 1 and 2 (compounds **1**, **2**). The sera from patients with stage 3 colorectal cancer showed higher IgM binding than those from other patients, which was also observed previously by others in lung cancer^18^. However, no significant difference was observed between colorectal cancer samples and healthy controls. Interestingly, even though similar binding profiles to Ser-linked and Thr-linked O-glycans were observed for lectins (Fig. 6), the Ser or Thr amino acid residue in the O-GalNAc glycans made a significant difference in antibody binding. Stronger bindings to Ser-linked Tn (**6**), core 2 (**2**), core 3 (**3**), and core 4 (**4**) than their Thr-linked counterparts (**6**-**T**, **2**-**T**, **3**-**T**, and **4**-**T**) (Fig. 7, *p* < 0.01) were observed for serum samples from both colorectal cancer patients and healthy controls. Moreover, glycan isomers presenting the same terminal epitopes, such as di-sialylated core 2 structures (**51**, **55**, and **56**), showed a significant binding difference (*p* < 0.01) by IgM. A similar IgM binding difference was seen for glycan isomers **26**/**27** and **69**/**71** (*p* < 0.01). The results suggested differential recognition of glycan isomers by serum anti-glycan antibodies. Another observation was that the extended O-glycans had lower overall IgM bindings than core structures (Fig. 7). Collectively, our results showed different serum antibody bindings to individual O-GalNAc cores as well as isomeric structures, providing insights to O-GalNAc glycan structure-activity relationship. Particularly, serum antibodies to rare O-GalNAc core 6 and less common cores 3 and 4 may serve as targets for cancer biomarker discovery.

In summary, we disclose an efficient chemoenzymatic modular assembly (CEMA) synthetic strategy that is used for the construction of a comprehensive cores 1–4 and 6-derived O-GalNAc glycan library presenting numerous natural epitopes including LN, LDN, 3-sialyl, 6-sialyl, 3SLN, 6SLN, Le^X^, SLe^X^, Le^Y^, and blood group H, A, B, and Cad/Sd^a^ antigens. The CEMA strategy enables rapid access to 83 structurally diverse O-GalNAc glycans by deploying 3 chemical synthetic building blocks (a universal monosaccharide-amino acid acceptor **7** and two glycosyl donors **8** and **9**) and 13 well-tailored glycosyltransferase modules in a precisely controlled sequential manner. The strategy can be readily adopted for the synthesis of cores 5, 7, and 8-derived O-GalNAc glycans and other complex carbohydrates. The synthetic O-GalNAc glycan microarray represents a powerful platform to investigate the structure-function relationship of mucin O-glycans. In addition to being used directly in glycan–protein interaction studies, the structurally diverse, ready-to-conjugate glycan-amino acid probes can also be used as building blocks for solid-phase synthesis of glycopeptides.

## Methods

### General procedure of high-performance liquid chromatography

An analytical GL Science Inertsil ODS-4 column (100 Å, 5 μm, 4.6 mm × 250 mm) was used to monitor reactions and for final purity analysis. The signals were monitored by a UV detector (260 nm) or fluorescent detector (Ex 260 nm, Em 310 nm). Analysis was performed under a gradient running condition (solvent A: H_2_O with 0.1% TFA; solvent B: acetonitrile with 0.1% TFA; flow rate: 1 mL/min; B%: 20–40 within 25 min). With similar running conditions, the analytical Inertsil ODS-4 column was used for separating up to 3 mg of products, and a semipreparative Inertsil ODS-4 column (100 Å, 5 μm, 10 mm × 250 mm) was used for larger scales with a flow rate of 4 mL/min.

### General procedure for enzymatic extensions

Reaction mixtures contain Tris-HCl (100 mM, pH 7.5 or 8.0), an acceptor glycan (10 mM), a donor (15 mM), MgCl_2_ (10 mM), and an appropriate amount of enzyme. Reactions were incubated at 37 °C and monitored by HPLC and/or MALDI-TOF MS. After over 90% acceptor was converted, the reaction was quenched, concentrated and subject to HPLC separation. Product-containing fractions were pooled and lyophilized for characterization and next step modular assembly.

### Method for microarray fabrication

The O-GalNAc microarray was printed according to the guidelines of MIRAGE as summarized in Supplementary Table 1. Thr-linked O-glycans, O-glycopeptides PPA [APGS(GalNAcα-)TAPP] and PPAP [TSAPD(GalNAcα-)TRPAP] (Z Biotech), and Ser-linked O-glycans prepared in this study were prepared at a concentration of 100 μM in the printing buffer (150 mM phosphate, pH 8.5), and printed on Nexterion slide H-3D hydrogel coated glass microarray slides (Applied Microarrays Inc), each for 400 pL in triplicates. Printing buffer was printed as a negative control, biotinylated PEG amine (0.01 mg/mL), mouse IgG (0.1 mg/mL) and human IgG (0.1 mg/mL) were printed in three replicates to serve as positive controls. A marker containing anti-human IgG-Cy3 conjugate (0.01 mg/mL) and anti-human IgG-Alexa647 conjugate (0.01 mg/mL) was printed in triplicates. A sciFLEXARRAYER S3 non-contacting ultralow volume dispensing system equipped with two PDC 80 Piezo dispense capillaries (Scienion) was used to spot glycans onto NHS-activated glass slides (16 subarrays on each slide). The spotting was carried out at room temperature with a humidity of 60%, followed by overnight dehumidification. Printed slides were then soaked in blocking buffer (50 mM ethanolamine, 100 mM Tris-HCl, pH 9.0) for 2 h, washed twice using MilliQ water, desiccated, and stored at −20 °C until use.

### Method for microarray assay

All steps were performed at room temperature. Before assay, each subarray was separated by fitting the slides with ProPlate 16-well microarray modules and incubated using 100 μL of TSMTB buffer (20 mM Tris-HCl, 150 mM NaCl, 2 mM CaCl_2_, 2 mM MgCl_2_, 0.05% (v/v) Tween-20, 1% (w/v) BSA, pH 7.4) for 10 min. For assay, TSMTB was aspirated and 100 μL of GBPs or serum samples at appropriate concentrations in TSMTB were added. The slides were then sealed and incubated for 1 h with gentle shaking, followed by washing for four times with TSMT buffer (TSMTB buffer without BSA). Next, 100 μL of fluorescence-labeled secondary antibody or Cy5-streptavidin was added to each subarray, sealed, and incubated for 1 h with gentle shaking. After incubation, the slides were washed four times with TSMT, TSM (TSMT without Tween-20), and MilliQ water, respectively, and dried by brief centrifugation for scanning. A Genepix 4100A microarray scanner (Molecular Devices) was used to image the slides at 80% power and 500 or 600 PMT gains. The resultant images were analyzed using the Genepix Pro 6.1 software and processed using Excel to obtain microarray results. Biotin-labeled lectins were detected by Cy5-streptavidin (1 µg/mL). Anti-STn antibody, anti-MUC-1 antibody, anti-CD15 antibody (10 µg/mL) and anti-CD15s antibody (10 µg/mL) were detected by corresponding fluorescent-labeled secondary antibody (5 µg/mL). Recombinant influenza A virus hemagglutinin proteins were detected with Alexa 647-conjugated anti-His-tag antibody (5 µg/mL). Human serum specimens from colorectal cancer patients and healthy people were provided by Georgia Cancer Center at Augusta University and stored at −80 °C until use. The protocol for serum specimen preparation was approved by the Institutional Review Board of Augusta University and was performed in accordance with the Helsinki Declaration. All participants gave written informed consent. Human serum specimens were analyzed in a 1:50 dilution and detected using Dylight 650 anti-human IgG Fc (Invitrogen) and Dylight 550 anti-human IgM antibodies (Invitrogen) (5 µg/mL).

### Reporting summary

Further information on research design is available in the Nature Research Reporting Summary linked to this article.

## Supplementary information

Supplementary Information

Reporting Summary

## Source data

Source Data

## Data Availability

The data supporting the findings of this study are available within the article and its Supplementary Information. Other relevant data are available from the corresponding author upon reasonable request. The Source data underlying Figs. 6, 7, Supplementary Figs. 5–11 are provided as a Source Data file. Source data are provided with this paper.
