# Supplementary material for: Chemoenzymatic modular assembly of O-GalNAc glycans for functional glycomics
Source: Nat Commun. 2021 Jun 11;12:3573. doi: 10.1038/s41467-021-23428-x (PMC8196059; doi:10.1038/s41467-021-23428-x)
Supplement: Supplementary file 1 — Supplementary Information [file 41467_2021_23428_MOESM1_ESM.pdf]

SUPPLEMENTARY INFORMATION

Chemoenzymatic Modular Assembly of O-GalNAc Glycans for Functional Glycomics

Shuaishuai Wang[1], Congcong Chen[1,2,3], Madhusudhan Reddy Gadi[1], Varma Saikam[1], Ding Liu[1], He Zhu[1], Roni Bollag[4], Kebin Liu[5], Xi Chen[6], Fengshan Wang[7], Peng George Wang[1,8]*, Peixue Ling[2,3,7]*, Wanyi Guan[9]*, and Lei Li[1]*

[1]Department of Chemistry, Georgia State University, Atlanta, GA 30303, USA

[2]National Glycoengineering Research Center, Shandong Provincial Key Laboratory of Glycochemistry and Glycobiology, Shandong University, Qingdao, Shandong 266237, China

[3]Shandong Academy of Pharmaceutical Science, Key Laboratory of Biopharmaceuticals, Engineering Laboratory of Polysaccharide Drugs, National-Local Joint Engineering Laboratory of Polysaccharide Drugs, Jinan, Shandong 250101, China

[4]Georgia Cancer Center, Augusta University, Augusta, GA 30912, USA

[5]Department of Biochemistry and Molecular Biology, Medical College of Georgia, Augusta, GA 30912, USA

[6]Department of Chemistry, University of California, Davis, CA 95616, USA

[7]Key Laboratory of Chemical Biology (Ministry of Education), Institute of Biochemical and Biotechnological Drug, School of Pharmaceutical Science, Shandong University, Jinan, Shandong 250012, China

[8]Present Address: School of Medicine, Southern University of Science and Technology, Shenzhen, Guangdong 518055, China

[9]College of Life Science, Hebei Normal University, Shijiazhuang, Hebei 050024, China

These authors contributed equally: Shuaishuai Wang, Congcong Chen, Madhusudhan Reddy Gadi, Varma Saikam

*Correspondence should be addressed to L.L. (email: lli22@gsu.edu) or to W.G. (email: guanwanyi@hebtu.edu.cn) or to P.L. (email: lpxsdf@163.com) or to P.G.W (email: wangp6@sustech.edu.cn)

Table of contents

I. Supplementary Figures and Tables 3

Supplementary Figure 1. Enzymatic modular assembly of core 3 O-GalNAc glycans. 3

Supplementary Figure 2. Enzymatic modular assembly of core 4 O-GalNAc glycans. 4

Supplementary Figure 3. Enzymatic modular assembly of core 6 O-GalNAc glycans. 5

Supplementary Figure 4. Structures of all O-GalNAc glycans prepared in this study. 6

Supplementary Figure 5. Binding profile of Fuc-specific lectins towards the O-GalNAc glycan microarray 7

Supplementary Figure 6. Binding profile of Sia-specific lectins towards the O-GalNAc glycan microarray 8

Supplementary Figure 7. Binding profile of LacNAc-specific lectins and GlcNAc-specific lectin towards the O-GalNAc glycan microarray 9

Supplementary Figure 8. Binding profile of GalNAc-specific lectins towards the O-GalNAc glycan microarray 10

Supplementary Figure 9. Binding profile of anti-glycan antibodies towards the O-GalNAc glycan microarray 11

Supplementary Figure 10. Binding profile of recombinant HA proteins towards the O-GalNAc glycan microarray 12

Supplementary Figure 11. Binding profile of lectins towards the O-GalNAc glycans (Supplementary Table 4) on the CFG microarray. 13

Supplementary Figure 12. Heatmap of lgG bindings on the O-GalNAc glycan microarray in sera from colorectal cancer patients and healthy control people 14

Supplementary Table 1. Glycan microarray information based on MIRAGE. 15

Supplementary Table 2. Glycan binding proteins used in this study. 16

Supplementary Table 3. Summary of binding specificity and fine details of tested GBPs towards O-GalNAc glycans 17

Supplementary Table 4. List of O-glycans from the CFG glycan microarray. 18

Supplementary Table 5. Colorectal cancer patients and healthy control people serum specimens used in this study. 20

II. Chemical Modular Assembly of cores 1–4 and 6 21

General information 21

Chemical procedures with analytical data for the synthesis of O-GalNAc cores 1–4 and 6 21

III. Enzymatic Modular Assembly to Diversify O-GalNAc Glycans 35

Materials 35

General HPLC methods 35

Enzyme Modules 35

IV. Glycan Microarray Fabrication and Assay 39

Method for removing Fmoc 39

Method for microarray fabrication 39

Method for microarray assay 39

V. HPLC, Mass Spectrometry, and NMR Data of Enzymatically Assembled Glycans 41

VI. NMR Spectra 118

VII. Supplementary References 188

I. Supplementary Figures and Tables

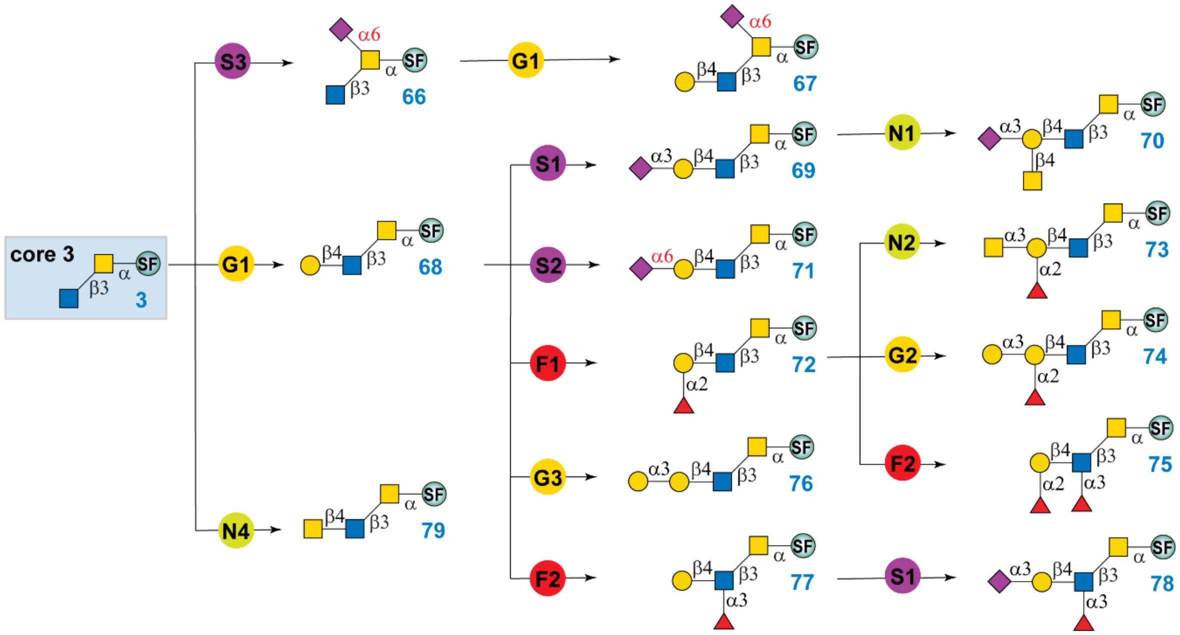

Supplementary Figure 1. Enzymatic modular assembly of core 3 O-GalNAc glycans.

Enzyme modules: G stands for galactosylation, S stands for sialylation, N stands for *N*-acetylhexosaminylation, F stands for fucosylation. G1: β1-4 galactosylation with *Neisseria meningitidis* β1-4 galactosyltransferase (NmLgtB) and donor uridine 5'-diphosphogalactose (UDP-Gal); G2: α1-3 galactosylation with human GTB and UDP-Gal; G3: α1-3 galactosylation with bovine α1-3 GalT (Bα3GalT) and UDP-Gal; S1: α2-3 sialylation with *Pasteurella multocida* α2-3 sialyltransferase 1 mutant M144D (PmST1-M144D), *N. meningitidis* CMP-sialic acid synthetase (NmCSS), cytidine 5'-triphosphate (CTP), and *N*-acetylneuraminic acid (Neu5Ac); S2: α2-6 sialylation with PmST1-P34H/M144L, NmCSS, CTP, and Neu5Ac; S3: α2-6 sialylation with *Photobacterium damselae* α2-6 sialyltransferase (Pd2,6ST), NmCSS, CTP, and Neu5Ac; N1: β1-4 *N*-acetylgalactosaminylation with *Campylobacter jejuni* β1-4 *N*-acetylgalatosaminyltransferase (CjCgtA) and UDP-GalNAc; N2: α1-3 *N*-acetylgalactosaminylation with *Helicobacter mustelae* α1-3 *N*-acetyl-galactosaminyltransferase (HmBgtA) and UDP-GalNAc; N4: β1-4 *N*-acetylgalactosaminylation with b4GalT-Y289L/C342T (b4GalTm) and UDP-GalNAc; F1: α1-2 fucosylation with *H. mustelae* α1-2 fucosyltransferase (Hm2FT) and guanosine 5'-diphospho-L-fucose (GDP-Fuc); F2: α1-3 fucosylation with *H. pylori* α1-3 fucosyltransferase C-terminal 66 amino acid truncation (Hp3FT) and GDP-Fuc. Abbreviations: Gal, galactose; Fuc, L-fucose; GlcNAc, *N*-acetylglucosamine; GalNAc, *N*-acetylgalactosamine; Neu5Ac, *N*-acetylneuraminic acid; SF, Fmoc protected Ser.

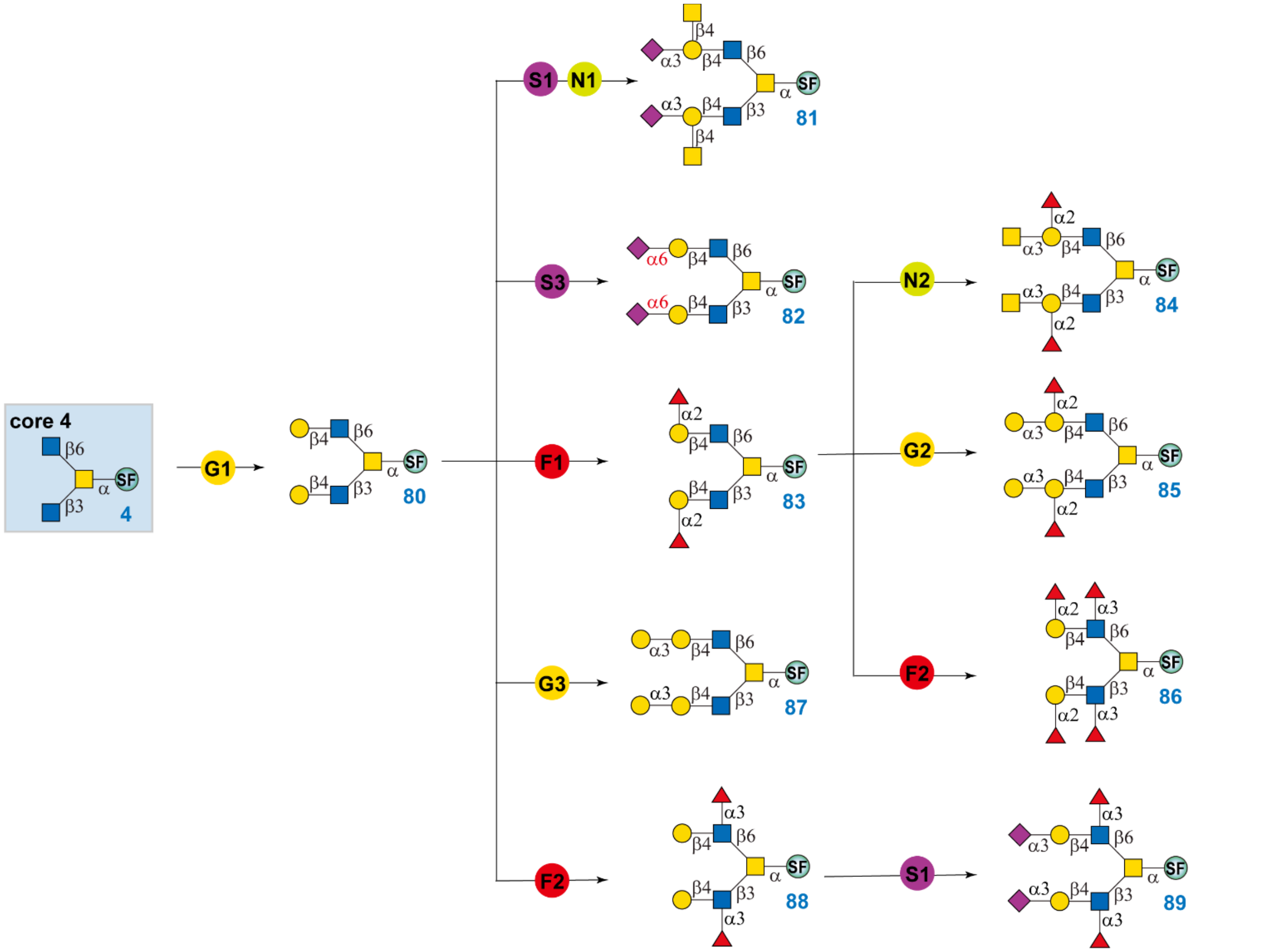

Supplementary Figure 2. Enzymatic modular assembly of core 4 O-GalNAc glycans.

Enzyme modules: G stands for galactosylation, S stands for sialylation, N stands for *N*-acetylhexosaminylation, F stands for fucosylation. G1: β1-4 galactosylation with *Neisseria meningitidis* β1-4 galactosyltransferase (NmLgtB) and donor uridine 5'-diphosphogalactose (UDP-Gal); G2: α1-3 galactosylation with human GTB and UDP-Gal; G3: α1-3 galactosylation with bovine α1-3 GalT (Bα3GalT) and UDP-Gal; S1: α2-3 sialylation with *Pasteurella multocida* α2-3 sialyltransferase 1 mutant M144D (PmST1-M144D), *N. meningitidis* CMP-sialic acid synthetase (NmCSS), cytidine 5'-triphosphate (CTP), and *N*-acetylneuraminic acid (Neu5Ac); S3: α2-6 sialylation with *Photobacterium damselae* α2-6 sialyltransferase (Pd2,6ST), NmCSS, CTP, and Neu5Ac; N1: β1-4 *N*-acetylgalactosaminylation with *Campylobacter jejuni* β1-4 *N*-acetylgalatosaminyltransferase (CjCgtA) and UDP-GalNAc; N2: α1-3 *N*-acetylgalactosaminylation with *Helicobacter mustelae* α1-3 *N*-acetyl-galactosaminyltransferase (HmBgtA) and UDP-GalNAc; F1: α1-2 fucosylation with *H. mustelae* α1-2 fucosyltransferase (Hm2FT) and guanosine 5'-diphospho-L-fucose (GDP-Fuc); F2: α1-3 fucosylation with *H. pylori* α1-3 fucosyltransferase C-terminal 66 amino acid truncation (Hp3FT) and GDP-Fuc. SF, Fmoc protected Ser.

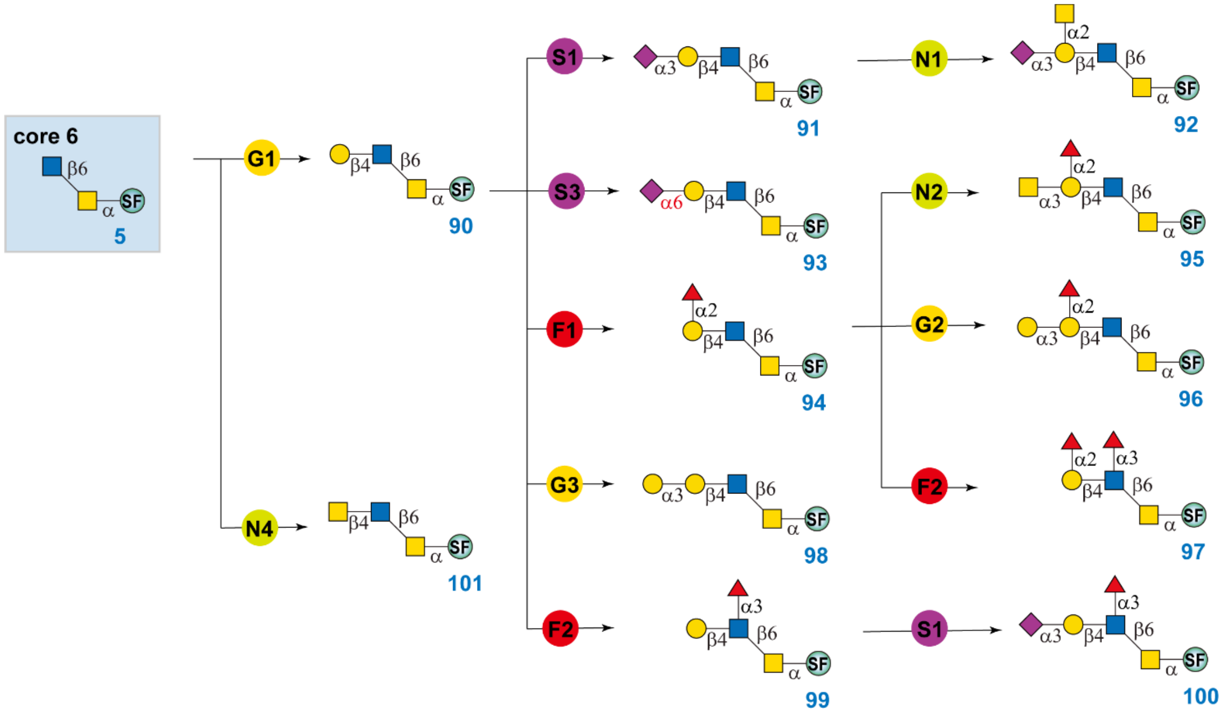

Supplementary Figure 3. Enzymatic modular assembly of core 6 O-GalNAc glycans.

Enzyme modules: G stands for galactosylation, S stands for sialylation, N stands for *N*-acetylhexosaminylation, F stands for fucosylation. G1: β1-4 galactosylation with *Neisseria meningitidis* β1-4 galactosyltransferase (NmLgtB) and donor uridine 5'-diphosphogalactose (UDP-Gal); G2: α1-3 galactosylation with human GTB and UDP-Gal; G3: α1-3 galactosylation with bovine α1-3 GalT (Bα3GalT) and UDP-Gal; S1: α2-3 sialylation with *Pasteurella multocida* α2-3 sialyltransferase 1 mutant M144D (PmST1-M144D), *N. meningitidis* CMP-sialic acid synthetase (NmCSS), cytidine 5'-triphosphate (CTP), and *N*-acetylneuraminic acid (Neu5Ac); S3: α2-6 sialylation with *Photobacterium damselae* α2-6 sialyltransferase (Pd2,6ST), NmCSS, CTP, and Neu5Ac; N1: β1-4 *N*-acetylgalactosaminylation with *Campylobacter jejuni* β1-4 *N*-acetylgalatosaminyltransferase (CjCgtA) and UDP-GalNAc; N2: α1-3 *N*-acetylgalactosaminylation with *Helicobacter mustelae* α1-3 *N*-acetyl-galactosaminyltransferase (HmBgtA) and UDP-GalNAc; N4: β1-4 *N*-acetylgalactosaminylation with b4GalT-Y289L/C342T (b4GalTm) and UDP-GalNAc; F1: α1-2 fucosylation with *H. mustelae* α1-2 fucosyltransferase (Hm2FT) and guanosine 5'-diphospho-L-fucose (GDP-Fuc); F2: α1-3 fucosylation with *H. pylori* α1-3 fucosyltransferase C-terminal 66 amino acid truncation (Hp3FT) and GDP-Fuc. SF, Fmoc protected Ser.

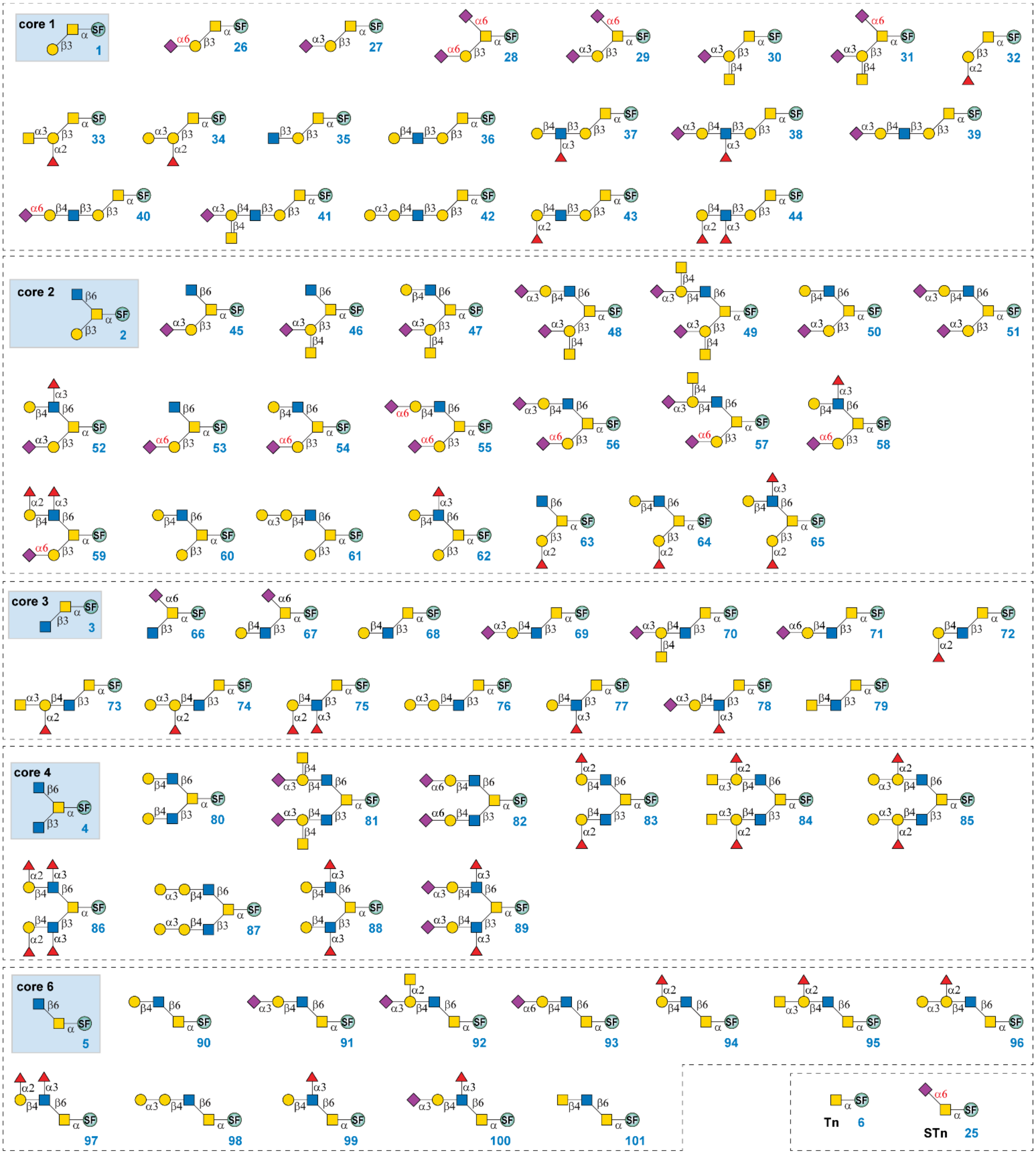

Supplementary Figure 4. **Structures of all O-GalNAc glycans prepared in this study.**

Microarray Results

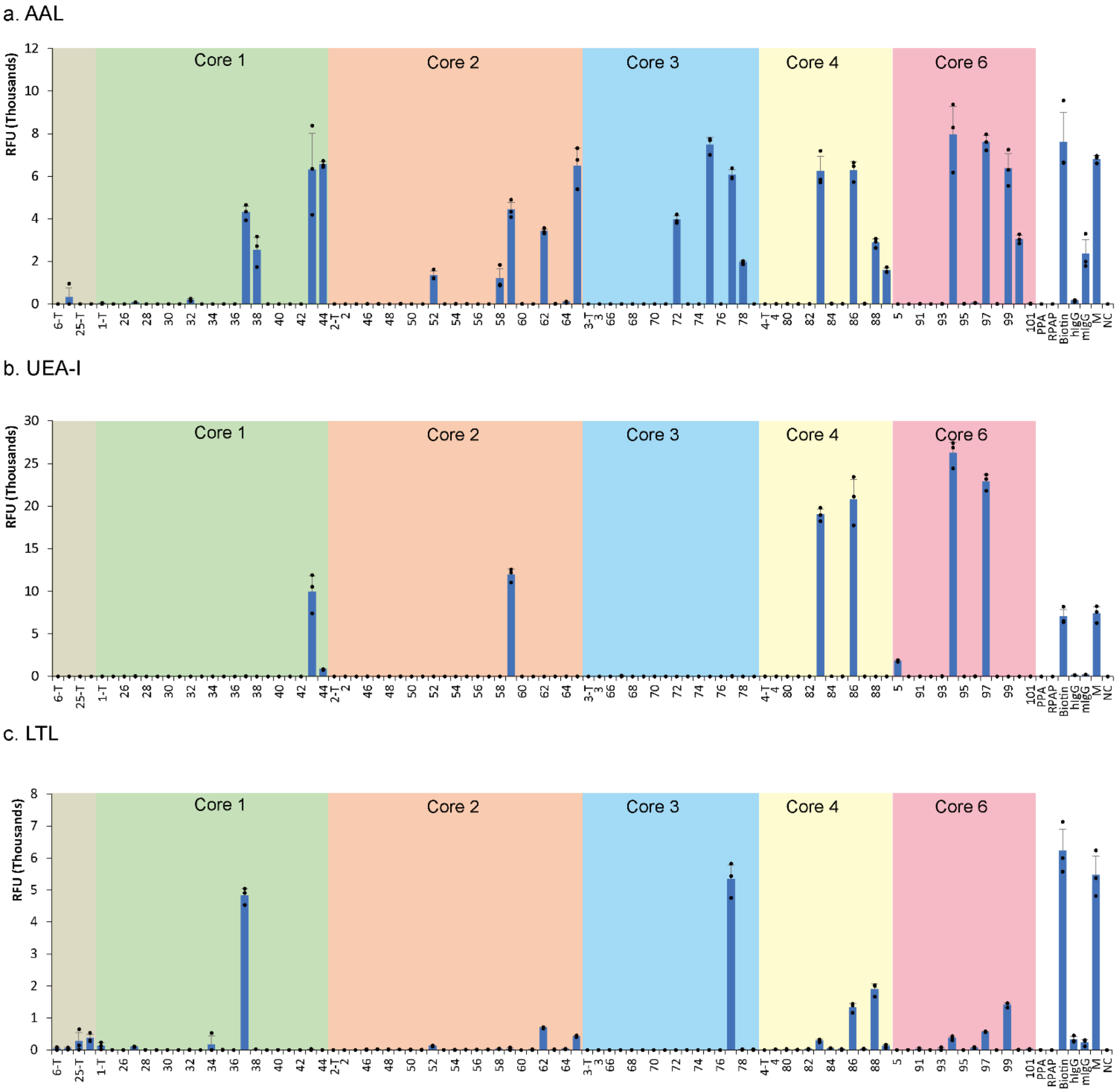

Supplementary Figure 5. Binding profile of Fuc-specific lectins towards the O-GalNAc glycan microarray

The *x*-axis shows the glycans, and the *y*-axis shows the relative fluorescence (readout by Cy5-streptavidin, 1 µg/mL). PPA = APGS(GalNAcα-)TAPP (100 µM); RPAP = TSAPD(GalNAcα-)TRPAP (100 µM); Biotin = biotinylated PEG amine (0.01mg/mL); hIgG = human IgG (0.1 mg/mL), mIgG = mouse IgG (0.1 mg/mL); M = Marker (0.01 mg/mL Cy3-conjugated anti-Human IgG + 0.01 mg/mL Alexs647-conjugated anti-Human IgG; NC = printing buffer. n=3 independent replicates. The individual data points are shown as dots. Data are presented as mean values; error bars represent standard deviation. Source data are provided as a Source Data file.

a. MAL-I

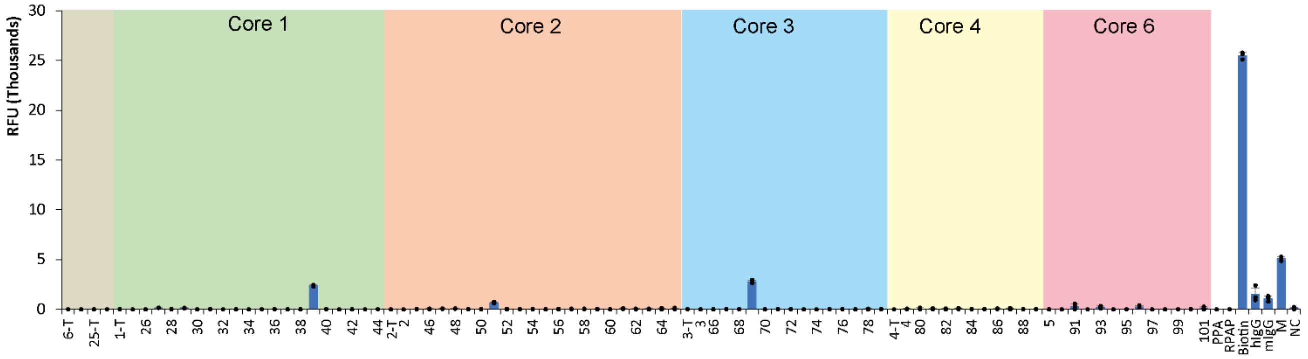

b. SNA

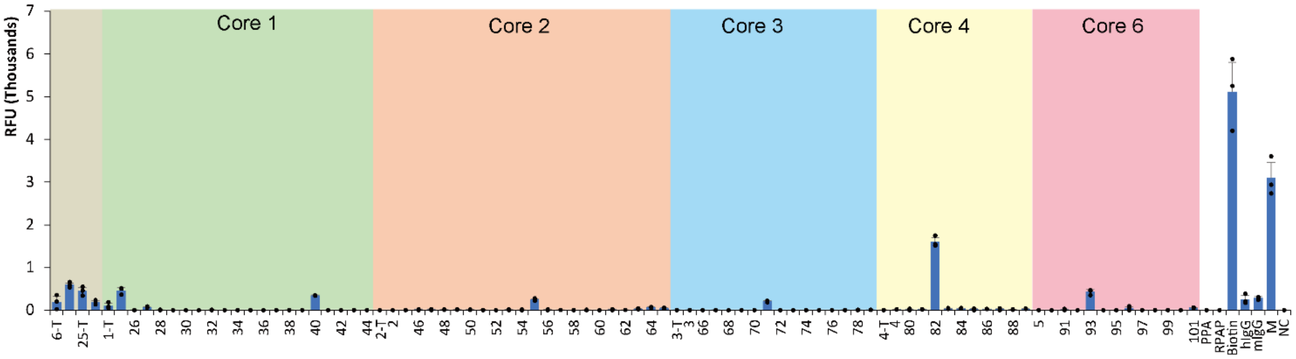

Supplementary Figure 6. Binding profile of Sia-specific lectins towards the O-GalNAc glycan microarray
The *x*-axis shows the glycans, and the *y*-axis shows the relative fluorescence (readout by Cy5-streptavidin, 1 µg/mL). PPA = APGS(GalNAcα-)TAPP (100 µM); RPAP = TSAPD(GalNAcα-)TRPAP (100 µM); Biotin = biotinylated PEG amine (0.01mg/mL); hIgG = human IgG (0.1 mg/mL), mIgG = mouse IgG (0.1 mg/mL); M = Marker (0.01 mg/mL Cy3-conjugated anti-Human IgG + 0.01 mg/mL Alexs647-conjugated anti-Human IgG; NC = printing buffer. n=3 independent replicates. The individual data points are shown as dots. Data are presented as mean values; error bars represent standard deviation. Source data are provided as a Source Data file.

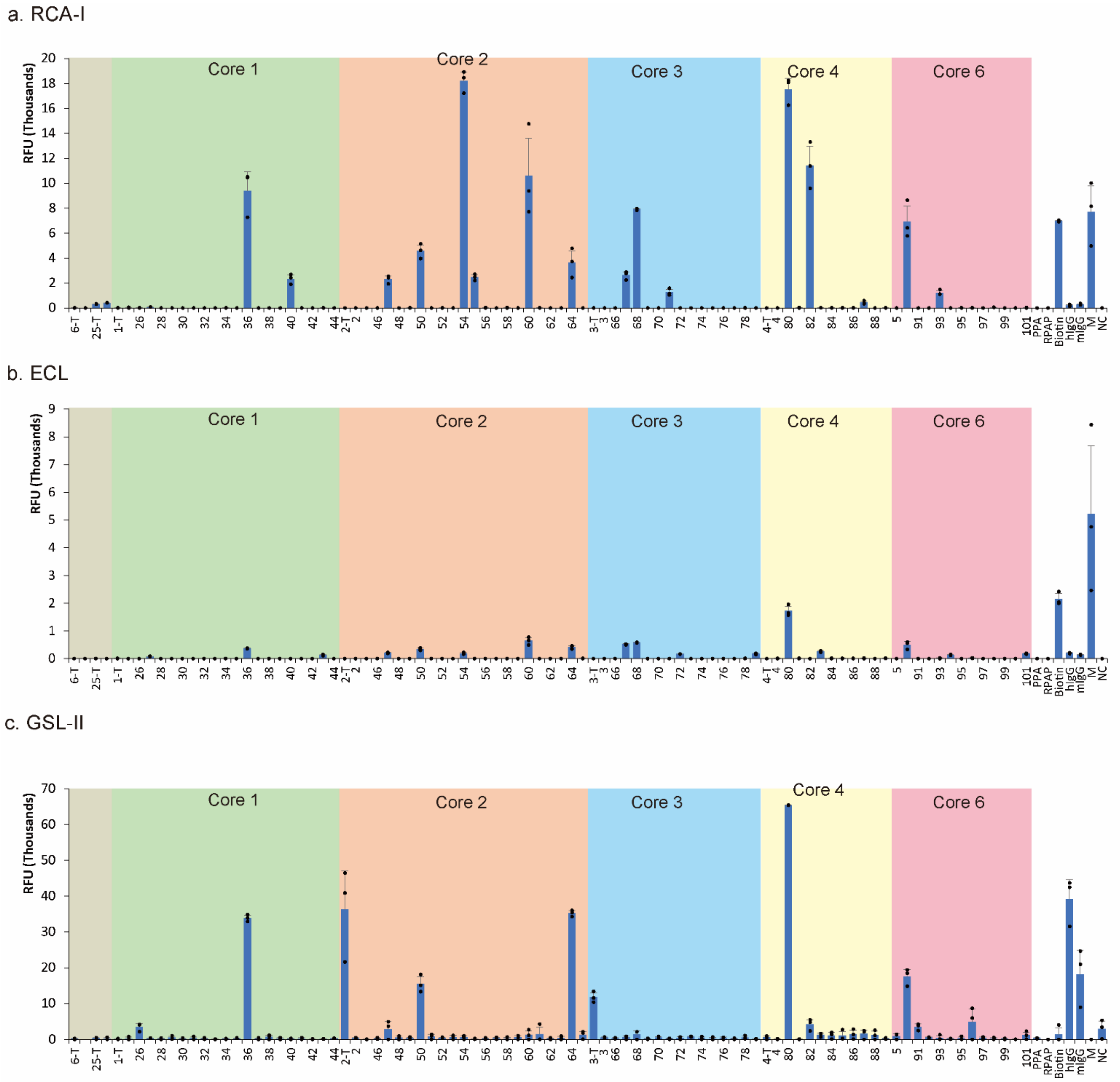

Supplementary Figure 7. Binding profile of LacNAc-specific lectins and GlcNAc-specific lectin towards the O-GalNAc glycan microarray

The *x*-axis shows the glycans, and the *y*-axis shows the relative fluorescence (readout by Cy5-streptavidin, 1 μg/mL). PPA = APGS(GalNAcα-)TAPP (100 μM); RPAP = TSAPD(GalNAcα-)TRPAP (100 μM); Biotin = biotinylated PEG amine (0.01mg/mL); hIgG = human IgG (0.1 mg/mL), mIgG = mouse IgG (0.1 mg/mL); M = Marker (0.01 mg/mL Cy3-conjugated anti-Human IgG + 0.01 mg/mL Alexs647-conjugated anti-Human IgG; NC = printing buffer. n=3 independent replicates. The individual data points are shown as dots. Data are presented as mean values; error bars represent standard deviation. Source data are provided as a Source Data file.

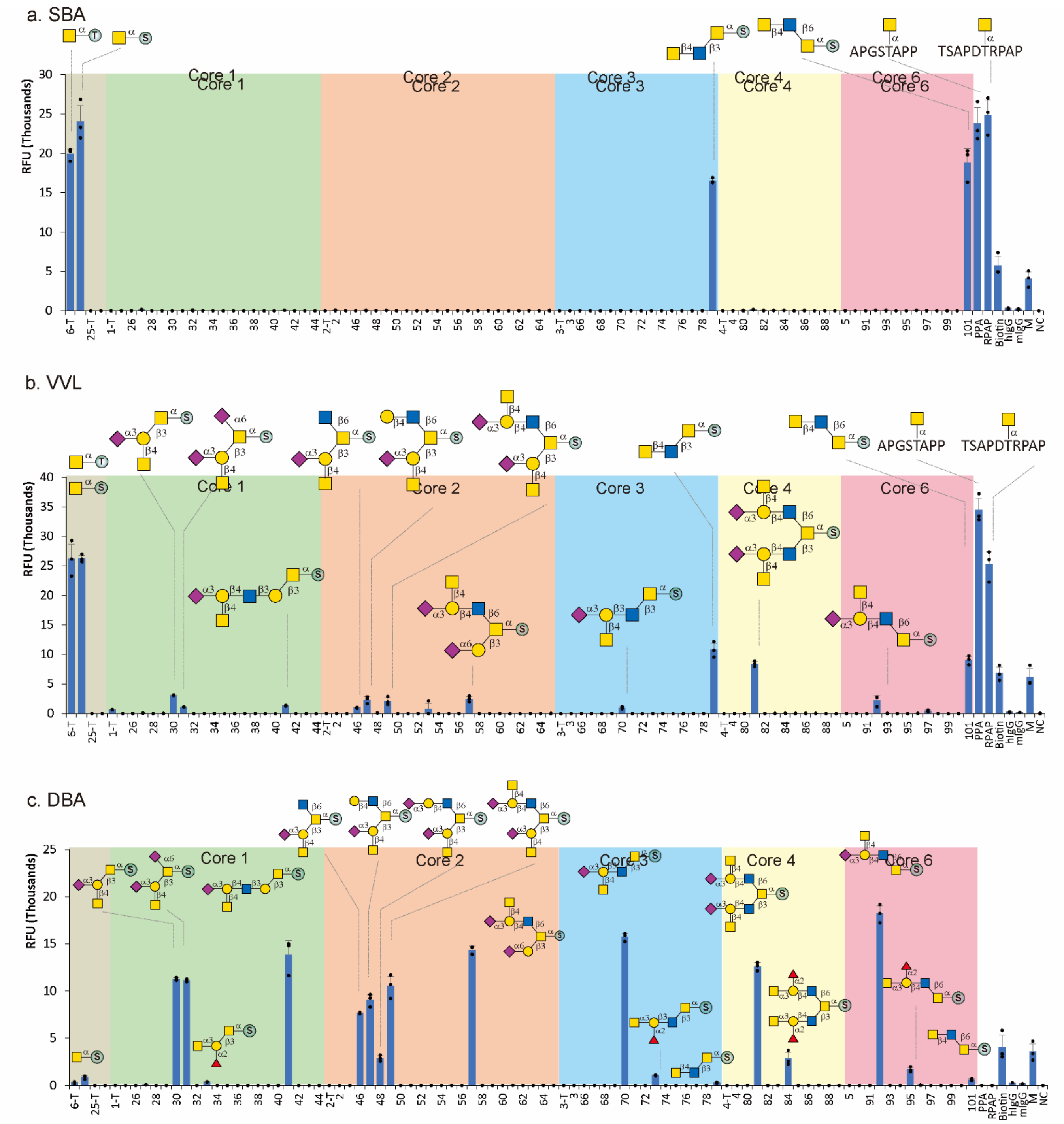

Supplementary Figure 8. Binding profile of GalNAc-specific lectins towards the O-GalNAc glycan microarray

The *x*-axis shows the glycans, and the *y*-axis shows the relative fluorescence (readout by Cy5-streptavidin, 1 µg/mL). PPA = APGS(GalNAcα-)TAPP (100 µM); RPAP = TSAPD(GalNAcα-)TRPAP (100 µM); Biotin = biotinylated PEG amine (0.01mg/mL); hIgG = human IgG (0.1 mg/mL), mIgG = mouse IgG (0.1 mg/mL); M = Marker (0.01 mg/mL Cy3-conjugated anti-Human IgG + 0.01 mg/mL Alexs647-conjugated anti-Human IgG; NC = printing buffer. n=3 independent replicates. The individual data points are shown as dots. Data are presented as mean values; error bars represent standard deviation. Source data are provided as a Source Data file.

a. Anti-CD15s

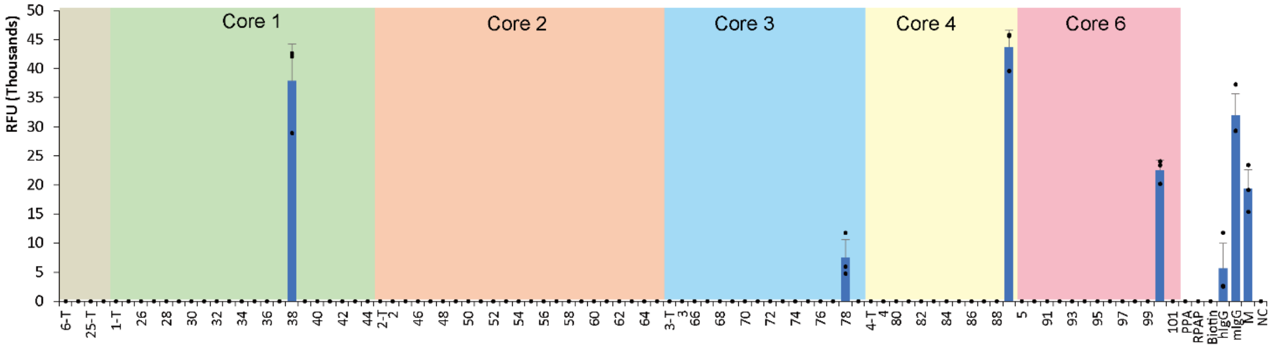

b. STn219

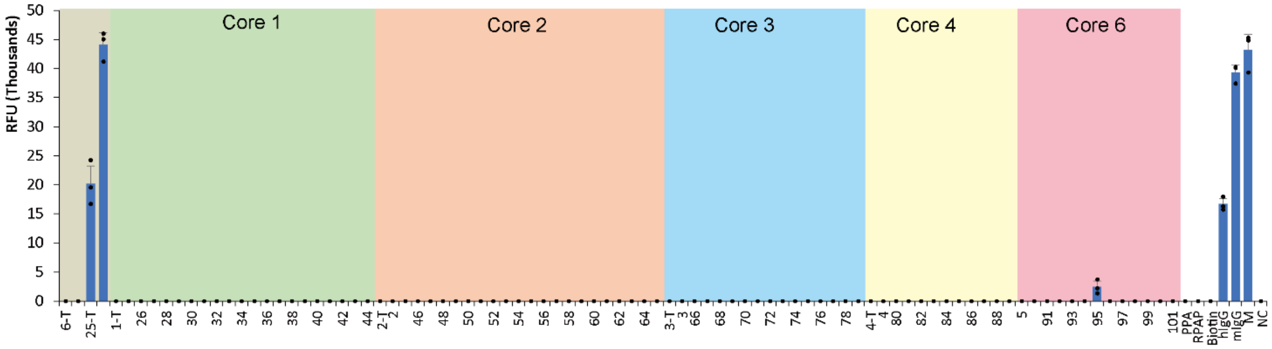

c. Anti-MUC-1

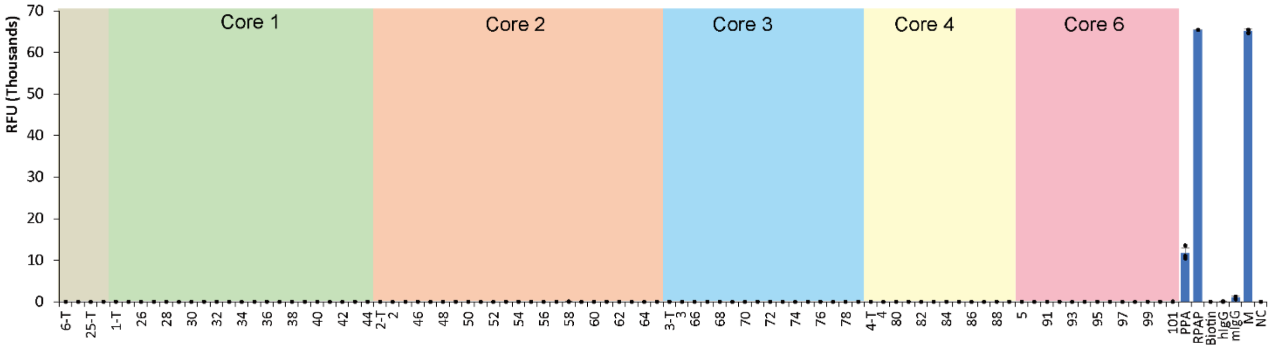

Supplementary Figure 9. Binding profile of anti-glycan antibodies towards the O-GalNAc glycan microarray

The *x*-axis shows the glycans, and the *y*-axis shows the relative fluorescence (readout by goat anti-mouse IgG-Alexa Fluor 647 conjugate, anti-Sheep IgG (H+L) CF™ 633 antibody produced in donkey, 5 µg/mL). PPA = APGS(GalNAcα-)TAPP (100 µM); RPAP = TSAPD(GalNAcα-)TRPAP (100 µM); Biotin = biotinylated PEG amine (0.01mg/mL); hIgG = human IgG (0.1 mg/mL), mIgG = mouse IgG (0.1 mg/mL); M = Marker (0.01 mg/mL Cy3-conjugated anti-Human IgG + 0.01 mg/mL Alexs647-conjugated anti-Human IgG; NC = printing buffer. n=3 independent replicates. The individual data points are shown as dots. Data are presented as mean values; error bars represent standard deviation. Source data are provided as a Source Data file.

a. H3

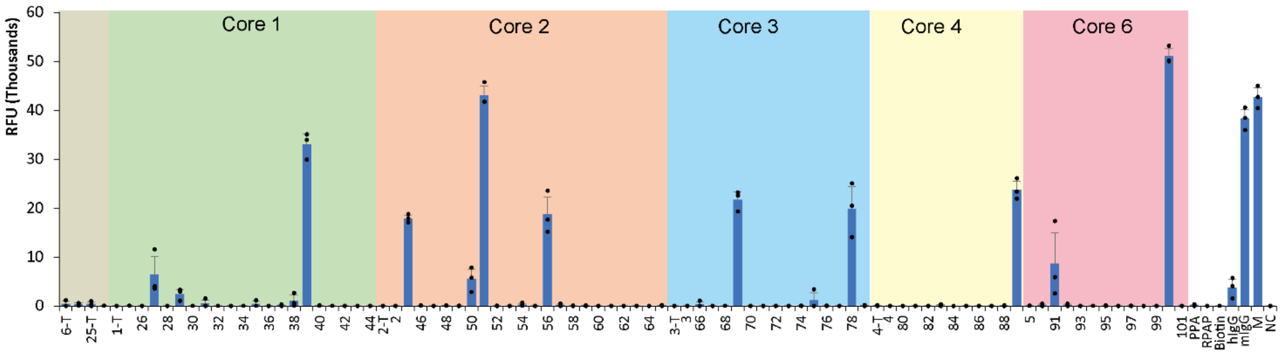

b. H1

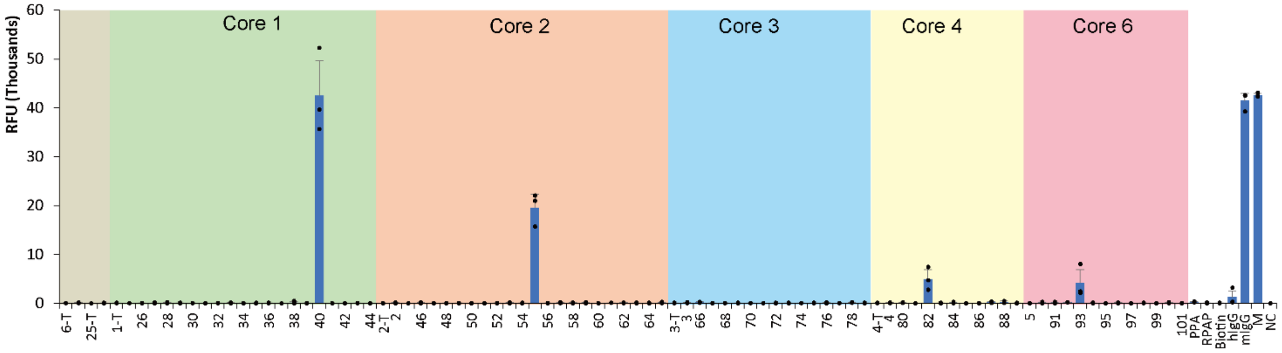

Supplementary Figure 10. Binding profile of recombinant HA proteins towards the O-GalNAc glycan microarray

The *x*-axis shows the glycans, and the *y*-axis shows the relative fluorescence (readout by mouse anti-His-tag antibody and goat anti-mouse IgG antibody with Alexa Fluor 647). PPA = APGS(GalNAcα-)TAPP (100 μM); RPAP = TSAPD(GalNAcα-)TRPAP (100 μM); Biotin = biotinylated PEG amine (0.01mg/mL); hIgG = human IgG (0.1 mg/mL), mIgG = mouse IgG (0.1 mg/mL); M = Marker (0.01 mg/mL Cy3-conjugated anti-Human IgG + 0.01 mg/mL Alexs647-conjugated anti-Human IgG; NC = printing buffer. n=3 independent replicates. The individual data points are shown as dots. Data are presented as mean values; error bars represent standard deviation. Source data are provided as a Source Data file.

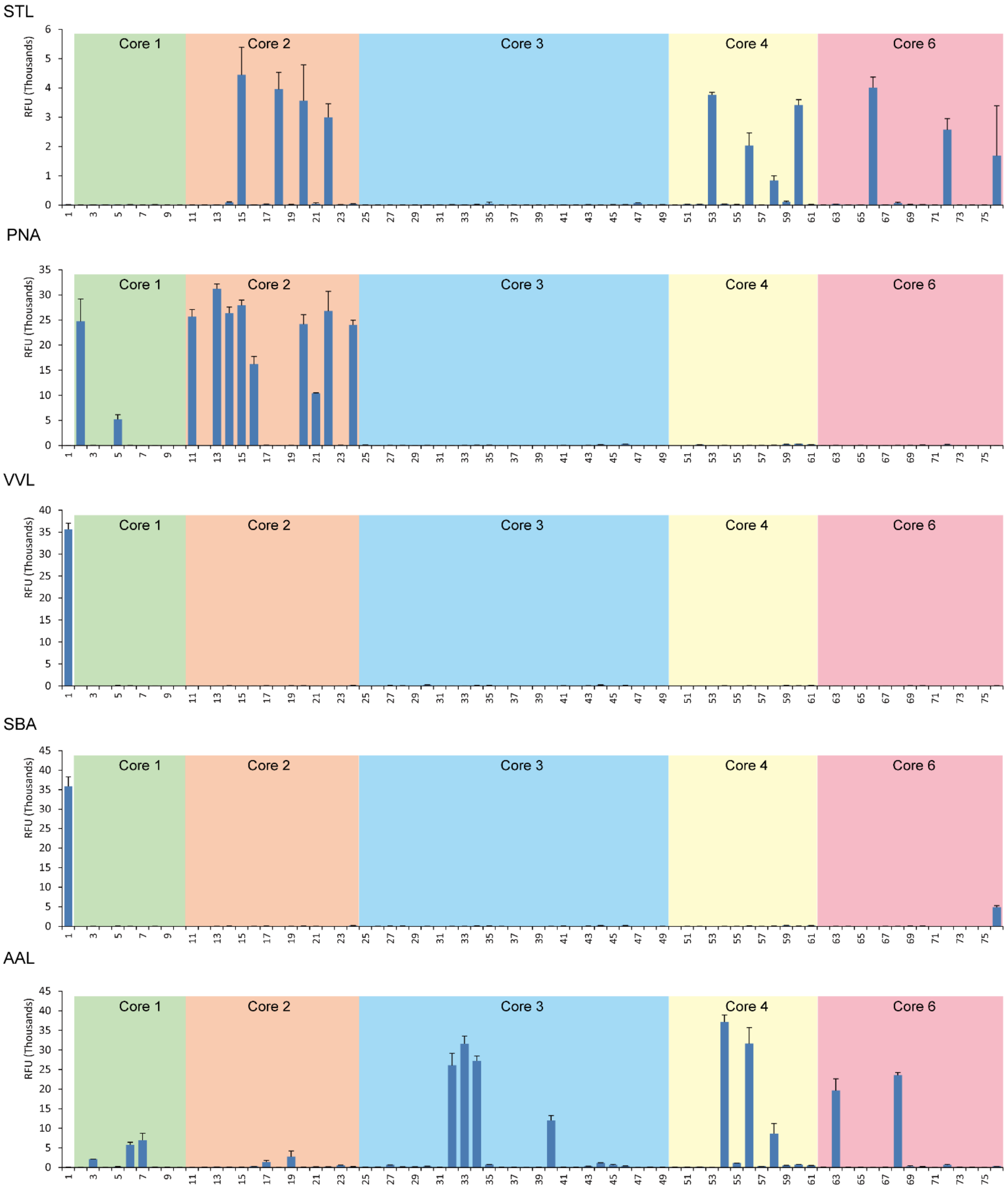

Supplementary Figure 11. Binding profile of lectins towards the O-GalNAc glycans (Supplementary Table 4) on the CFG microarray.

Data were obtained from NCFG website (https://ncfg.hms.harvard.edu/ncfg-data/microarray-data/lectin-quality-assurancequality-control).

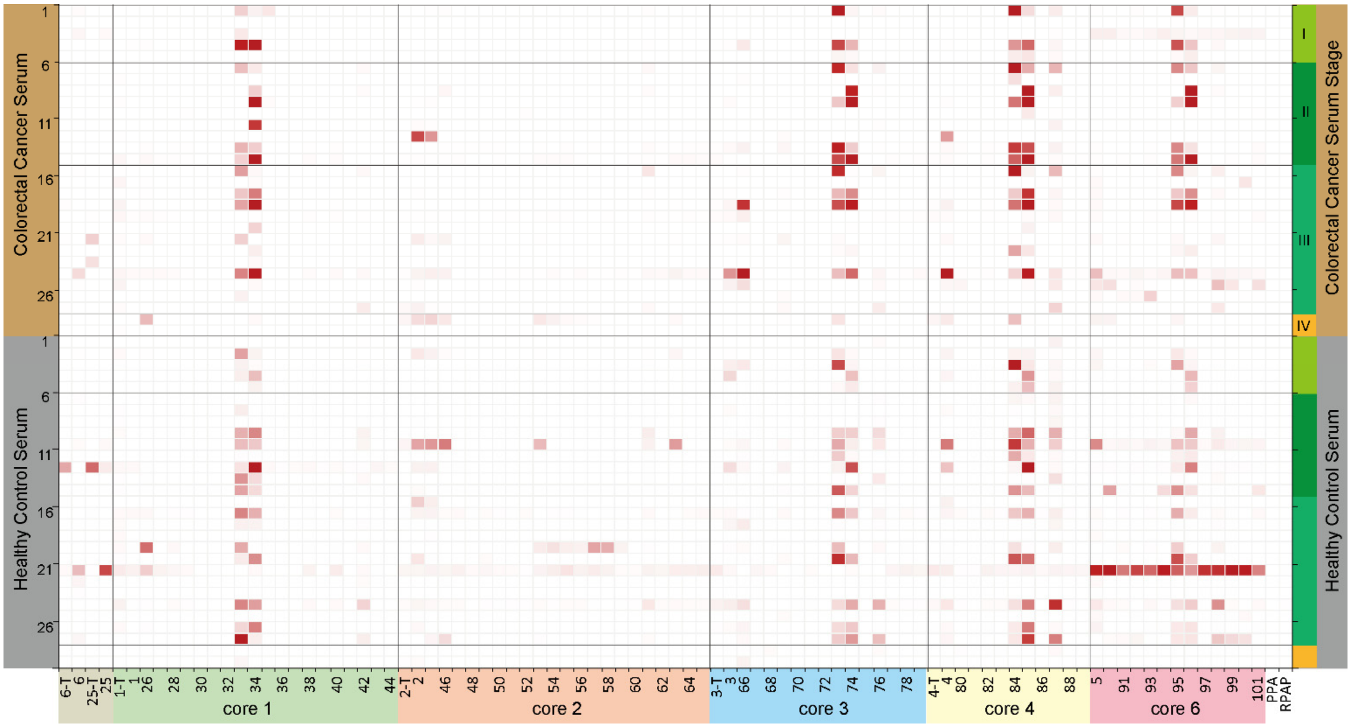

Supplementary Figure 12. **Heatmap of lgG bindings on the O-GalNAc glycan microarray in sera from colorectal cancer patients and healthy control people**

PPA = APGS(GalNAcα-)TAPP (100 μM); RPAP = TSAPD(GalNAcα-)TRPAP (100 μM); Biotin = biotinylated PEG amine (0.01mg/mL); NC = printing buffer. n=3 independent replicates. Data are presented as mean values. Source data are provided as a Source Data file.

Supplementary Table 1. Glycan microarray information based on MIRAGE.

Classification	Guidelines
**1. Sample: Glycan Binding Sample**	
Description of Sample	Human serum samples from colorectal cancer patients and healthy people were provided by Georgia Cancer Center at Augusta University. All lectins and antibodies were purchased as described in Section III. Method for microarray assay.
Sample modifications	Not applicable.
Assay protocol	Microarray analyses were performed essentially as described Section III. Method for microarray assay.
**2. Glycan Library**	
Glycan description for defined glycans	All glycans were synthesized as described in main text.
Glycan description for undefined glycans	Not applicable.
Glycan modifications	Glycans were linked with Ser.
**3. Printing Surface; e.g., Microarray Slide**	
Description of surface	Nexterion slide H-3D hydrogel coated glass microarray slides.
Manufacturer	Applied Microarrays Inc (Tempe, AZ, USA)
Custom preparation of surface	Not relevant.
Covalent Immobilization	Glycans were linked with Ser for robotically arraying and the amine group could covalently be immobilized on NHS ester coated glass slide.
**4. Arrayer (Printer)**	
Description of Arrayer	sciFLEXARRAYER S3 spotter (Scienion) with two PDC 80 Piezo Dispense Capillary, and 16 subarrays were printed on each slide
Dispensing mechanism	Non-contact liquid delivery.
Glycan deposition	Each glycan probe was printed at 1 deposit in 3 replicates.
Printing conditions	Samples were prepared at a concentration of 100 μM in the printing buffer (300 mM phosphate, pH 8.5), printing was performed at room temperature and relative humidity of 60%.
**5. Glycan Microarray with "Map"**	
Array layout	Each array slide contained 16 identical subarrays (pads). Each subarray contained up to 94 unique samples.
Glycan identification and QC	Quality control included analyses with plant lectins.
**6. Detector and Data Processing**	
Scanning hardware	GenePix 4000B Microarray Scanner (Molecular Devices, LLC)
Scanner settings	Laser channel: wavelength 635 nm or 535 nm<br>PMT gain: 600<br>Scan power:100%
Image analysis software	GnePix Pro (Molecular Devices, LLC)
Data processing	The gpr files were processed with in-house excel macro to obtain basic descriptive statistics. No particular normalization method or statistical analysis was used.
**7. Glycan Microarray Data Presentation**	
Data presentation	The microarray binding results are in Figures 6,7, Supplementary Figures 5-11, and Supplementary Table 3. Binding results are presented as relative fluorescence intensity units (RFU) of binding in mean and S.D. The individual data points are shown as dots.
**8. Interpretation and Conclusion from Microarray Data**	
Data interpretation	No software or algorithms were used to interpret processed data.
Conclusions	Described in Results parts.

Supplementary Table 2. Glycan binding proteins used in this study.

GBPs		Concentration	Binding Residues
AAL	Vector Lab	0.1μg/mL	Fucose
UEA-I	Vector Lab	20 μg/mL	
LTL	Vector Lab	20 μg/mL	
MAL-I	Vector Lab	10 μg/mL	Sialic acid
SNA	Vector Lab	20 μg/mL	
RCA-I	Vector Lab	20 μg/mL	LacNAc
ECL	Vector Lab	1 μg/mL	
GSL-II	EYLabs	10 μg/mL	GlcNAc
STL	Vector Lab	20 μg/mL	
PNA	Vector Lab	20 μg/mL	T antigen (Gal β1-3GalNAc α-)
Jacalin	Vector Lab	20 μg/mL	
SBA	Vector Lab	20 μg/mL	Tn antigen (GalNAc α -)
VVL	Vector Lab	2 μg/mL	
DBA	Vector Lab	20 μg/mL	
Anti-CD15s antibody	BD	20 μg/mL	SLe^X
STn219	Fisher	1:10	STn antigen
MUC1 antibody	R&D Systems	1:50	MUC
H3 of A/Brisbane/10/2007	BEI resources	10 μg/mL	Sialic acid
H1 of A/NewYork/18/2009	BEI resources	10 μg/mL	

Supplementary Table 3. Summary of binding specificity and fine details of tested GBPs towards O-GalNAc glycans

GBPs	Primary Ligand	Fine details (towards tested O-GalNAc glycans)	O-GalNAc core				
			**1**	**2**	**3**	**4**	**6**
AAL	α-Fuc	Bind α1-2/3Fuc; exclude A- and B-antigen	+[a]	+	+	+	+
UEA-I	α-Fuc	Specific to α1-2Fuc; exclude A- and B-antigen; strongly prefer β1-6GlcNAc branch	+	++[b]	-[c]	++	++
LTL	α-Fuc	Strongly prefer α1-3Fuc	+	+	+	+	+
MAL I	3SLN	Prefer β1-3Gal/GlcNAc branch	Weak binding				
SNA	6SLN	Specific for 6SLN	Weak binding				
RCA-I	LacNAc	Tolerate modification on β1-3Gal branch when β1-6GlcNAc branch presents LacNAc	++	++	++	++	++
ECL	LacNAc	Tolerate modification on β1-3Gal branch (excluding α2-6sialylation) when β1-6branch presents LacNAc	+	+	+	+	+
GSL-II	GlcNAc	Specific for terminal GlcNAc; in core 2 glycans with a free β1-6GlcNAc, α2-6Neu5Ac or β1-3GlcNAc on β1-3Gal branch blocks binding	++	++	+	++	+
STL	LacNAc LDN	Specific for ligands on β1-6GlcNAc branch; tolerate all modifications on ligands excluding α2-6 sialylation and α1-3 fucosylation	-	++	-	++	++
PNA	T antigen	Require free Gal on the Galβ1-3GalNAcα unit; tolerate β1-6GlcNAc branch modifications	++	++	-	-	-
Jacalin	Tn antigen	Any O-GalNAc glycans without modification on C6-OH of the initiating GalNAc	++	-	++	-	-
SBA	GalNAc	Terminal α/β-linked GalNAc excluding Cad/Sd[a] and A-antigen	-	-	+	-	-
VVL	GalNAc	Terminal α/β-linked GalNAc excluding A-antigen; weak binding to Cad/Sd[a]	+	+	+	+	+
DBA	GalNAc	Strong binding to Cad/Sd[a]; weak binding to Tn antigen, A-antigen and LDN	++	++	++	++	++
Anti-CD15s	SLe^X	Specific for SLe^X	++	-	+	++	++
H3N2 (A/Brisbane/10/2007)	Neu5Ac α2-3Gal	Bind to nearly all O-glycans with Neu5Acα2-3Gal, modifications on opposing branch could affect binding	++	++	++	++	++
H1N1 (A/New York/18/2009)	6SLN	Specific for 6SLN	++	++	-	+	+

[a]weak to moderate binding, [b]strong binding, [c]no binding based on tested O-GalNAc glycans.

Supplementary Table 4. List of O-glycans from the CFG glycan microarray.

Number	Structures
1	GalNAcα-Sp8
2	Galβ1-3GalNAcα-Sp14
3	KDNα2-3Galβ1-3GalNAcα-Sp14
4	Neu5Acα2-3Galβ1-3GalNAcα-Sp14
5	Neu5Acα2-6(Galβ1-3)GalNAcα-Sp14
6	Neu5Acα2-6(Neu5Acα2-3Galβ1-3)GalNAcα-Sp14
7	Fucα1-2Galβ1-3GalNAcα-Sp14
8	GlcNAcα1-4Galβ1-3GalNAc-Sp14
9	GlcNAβ1-3Galβ1-3GalNAc-Sp14
10	Galβ1-4GlcNAcβ1-3Galβ1-3GalNAcα-Sp14
11	GlcNAcβ1-6(Galβ1-3)GalNAcα-Sp14
12	GlcNAcβ1-6(Neu5Acα2-3Galβ1-3)GalNAcα-Sp14
13	Galβ1-3GlcNAcβ1-6(Galβ1-3)GalNAc-Sp14
14	Galβ1-4GlcNAcβ1-6(Galβ1-3)GalNAc-Sp14
15	Neu5Acα2-3Galβ1-4GlcNAcβ1-6(Galβ1-3)GalNAcα-Sp14
16	Neu5Acα2-6Galβ1-4GlcNAcβ1-6(Galβ1-3)GalNAcα-Sp14
17	Neu5Acα2-3Galβ1-4(Fucα1-3)GlcNAcβ1-6(Galβ1-3)GalNAcα-Sp14
18	Neu5Acα2-3Galβ1-4GlcNAcβ1-6(Neu5Acα2-3Galβ1-3)GalNAcα-Sp14
19	Neu5Acα2-3Galβ1-4(Fucα1-3)GlcNAcβ1-6(Neu5Acα2-3Galβ1-3)GalNAc-Sp14
20	Neu5Acα2-3Galβ1-4GlcNAcβ1-3Galβ1-4GlcNAcβ1-6(Galβ1-3)GalNAcα-Sp14
21	Neu5Aca2-6Galβ1-4GlcNAcβ1-3Galβ1-4GlcNAcβ1-6(Galβ1-3)GalNAca-Sp14
22	GlcNAcβ1-3Galβ1-4GlcNAcβ1-6(Galβ1-3)GalNAca-Sp14
23	GlcNAcβ1-3Galβ1-4GlcNAcβ1-6(GlcNAcβ1-3Galβ1-3)GalNAca-Sp14
24	Galβ1-4GlcNAcβ1-3Galβ1-4GlcNAcβ1-6(Galβ1-3)GalNAca-Sp14
25	GlcNAcβ1-3GalNAca-Sp14
26	Galβ1-3GlcNAcβ1-3GalNAca-Sp14
27	Galβ1-4GlcNAcβ1-3GalNAc-Sp14
28	Neu5Aca2-3Galβ1-3GlcNAcβ1-3GalNAca-Sp14
29	Neu5Aca2-3Galβ1-4GlcNAcβ1-3GalNAc-Sp14
30	Neu5Aca2-6Galβ1-4GlcNAcβ1-3GalNAc-Sp14
31	Fucα1-2Galβ1-3GlcNAcβ1-3GalNAc-Sp14
32	Fuca1-2Galβ1-4GlcNAcβ1-3GalNAcα–Sp14
33	Galβ1-4(Fucα1-3)GlcNAcβ1-3GalNAcα–Sp14
34	Neu5Acα2-3Galβ1-4(Fucα1-3)GlcNAcβ1-3GalNAcα–Sp14
35	Fucα1-2Galβ1-4(Fucα1-3)GlcNAcβ1-3GalNAcα–Sp14
36	Galα1-3Galβ1-3GlcNAcβ1-3GalNAc-Sp14
37	Galα1-3Galβ1-4GlcNAcβ1-3GalNAcα–Sp14
38	Galα1-3(Fucα1-2)Galβ1-3GlcNAcβ1-3GalNAc-Sp14
39	Galα1-3(Fucα1-2)Galβ1-4GlcNAcβ1-3GalNAcα–Sp14
40	Galα1-3(Fucα1-2)Galβ1-4(Fucα1-3)GlcNAcβ1-3GalNAc-Sp14
41	GalNAcα1-3(Fucα1-2)Galβ1-3GlcNAcβ1-3GalNAc-Sp14
42	GalNAcα1-3(Fucα1-2)Galβ1-4GlcNAcβ1-3GalNAcα–Sp14
43	GalNAcα1-3(Fucα1-2)Galβ1-4(Fucα1-3)GlcNAcβ1-3GalNAc-Sp14
44	GalNAcβ1-4(Neu5Acα2-3)Galβ1-4GlcNAcβ1-3GalNAcα–Sp14
45	GlcNAcβ1-3Galβ1-4GlcNAcβ1-3GalNAcα–Sp14
46	Galβ1-4GlcNAcβ1-3Galβ1-4GlcNAcβ1-3GalNAcα–Sp14

47	Neu5Acα2-3Galβ1-4GlcNAcβ1-3Galβ1-4GlcNAcβ1-3GalNAcα−Sp14
48	Neu5Acα2-6Galβ1-4GlcNAcβ1-3Galβ1-4GlcNAcβ1-3GalNAcα−Sp14
49	GlcNAcβ1-3Galβ1-4GlcNAcβ1-3Galβ1-4GlcNAcβ1-3GalNAcα−Sp14
50	GlcNAcβ1-6(GlcNAcβ1-3)GalNAcα−Sp14
51	Galβ1-4GlcNAcβ1-6(Galβ1-4GlcNAcβ1-3)GalNAc-Sp14
52	GlcNAcβ1-3Galβ1-4GlcNAcβ1-6(GlcNAcβ1-3Galβ1-4GlcNAcβ1-3)GalNAcα−Sp14
53	Neu5Acα2-3Galβ1-4GlcNAcβ1-6(Neu5Acα2-3Galβ1-4GlcNAcβ1-3)GalNAcα−Sp14
54	Neu5Acα2-6Galβ1-4 GlcNAcβ1-6(Neu5Acα2-6Galβ1-4GlcNAcβ1-3)GalNAcα−Sp14
55	Fucα1-2Galβ1-3GlcNAcβ1-6(Fucα1-2Galβ1-3GlcNAcβ1-3)GalNAcα−Sp14
56	Fucα1-2Galβ1-4GlcNAcβ1-6(Fucα1-2Galβ1-4GlcNAcβ1-3)GalNAc-Sp14
57	Galα1-3(Fucα1-2)Galβ1-4GlcNAcβ1-6(Galα1-3(Fucα1-2)Galβ1-4GlcNAcβ1-3)GalNAc-Sp14
58	GalNAcα1-3(Fucα1-2)Galβ1-4GlcNAcβ1-6(GalNAcα1-3(Fucα1-2)Galβ1-4GlcNAcβ1-3)GalNAc-Sp14
59	Galβ1-4GlcNAcβ1-3Galβ1-4GlcNAcβ1-6(Galβ1-4GlcNAcβ1-3Galβ1-4GlcNAcβ1-3)GalNAcα−Sp14
60	Neu5Acα2-3Galβ1-4GlcNAcβ1-3Galβ1-4GlcNAcβ1-6(Neu5Acα2-3Galβ1-4GlcNAcβ1-3Galβ1-4GlcNAcβ1-3)GalNAcα−Sp14
61	Neu5Acα2-6Galβ1-4GlcNAcβ1-3Galβ1-4GlcNAcβ1-6(Neu5Acα2-6Galβ1-4GlcNAcβ1-3Galβ1-4GlcNAcβ1-3)GalNAcα−Sp14
62	GlcNAcβ1-6GalNAcα−Sp14
63	Galβ1-4GlcNAcβ1-6GalNAc-Sp14
64	Galβ1-3GlcNAcβ1-6GalNAcα−Sp14
65	Neu5Acα2-3Galβ1-3GlcNAcβ1-6GalNAcα−Sp14
66	Neu5Acα2-3Galβ1-4GlcNAcβ1-6GalNAcα−Sp14
67	Neu5Acα2-6Galβ1-4GlcNAcβ1-6GalNAcα−Sp14
68	Fucα1-2Galβ1-4GlcNAcβ1-6GalNAcα−Sp14
69	Galβ1-3(Fucα1-4)GlcNAcβ1-6GalNAcα−Sp14
70	Galβ1-4(Fucα1-3)GlcNAcβ1-6GalNAc-Sp14
71	Galα1-3Galβ1-3GlcNAcβ1-6GalNAcα−Sp14
72	Galα1-3Galβ1-4GlcNAcβ1-6GalNAcα−Sp14
73	Galα1-3(Fucα1-2)Galβ1-3GlcNAcβ1-6GalNAc-Sp14
74	Galα1-3(Fucα1-2)Galβ1-4GlcNAcβ1-6GalNAcα−Sp14
75	GalNAcα1-3(Fucα1-2)Galβ1-3GlcNAcβ1-6GalNAcα−Sp14
76	GalNAcα1-3(Fucα1-2)Galβ1-4GlcNAcβ1-6GalNAc-Sp14

Supplementary Table 5. Colorectal cancer patients and healthy control people serum specimens used in this study.

#	TNM Stage	Stage Group
**C01**	no final staging	I
**C02**	pT1N0Mx	I
**C03**	pT2N0Mx	I
**C04**	pT2N0Mx	I
**C05**	pT2N0Mx	I
**C06**	pT2N0Mx	I
**C07**	pT3N0Mx	IIA
**C08**	pT3N0Mx	IIA
**C09**	pT3N0Mx	IIA
**C10**	pT3N0Mx	IIA
**C11**	pT3N0Mx	IIA
**C12**	pT3N0Mx	IIA
**C13**	pT3N0Mx	IIA
**C14**	pT3N0Mx	IIA
**C15**	pT4bN0	IIC
**C16**	pT2N1aMx	IIIA
**C17**	pT3N1aMx	IIIB
**C18**	pT3N1aMx	IIIB
**C19**	pT3N1aMx	IIIB
**C20**	pT3N1aMx	IIIB
**C21**	pT3N1aMx	IIIB
**C22**	pT3N1bMx	IIIB
**C23**	pT3N2aMx	IIIB
**C24**	pT3N2aMx	IIIB
**C25**	pT3N2bMx	IIIC
**C26**	pT4aN2bMx	IIIC
**C27**	pT4aN2bMx	IIIC
**C28**	pT3N1cM1	IVA
**C29**	pT4bN2bM1a	IVA

II. Chemical Modular Assembly of cores 1–4 and 6

General information

ESI-mass spectrometry were performed on an LTQ-Orbitrap Elite mass spectrometer (Thermo Fisher) equipped with EASY-spray source and nano-LC UltiMate 3000 high-performance liquid chromatography system (Thermo Fisher). Samples were transmitted into MS with a silica column. LTQ-Orbitrap Elite mass spectrometer was operated in the data-dependent mode. A full-scan survey MS experiment (*m/z* range was set according to the molecular weight of O-glycan; automatic gain control target, 1,000,000 ions; resolution at 400 *m/z*, 240,000; maximum ion accumulation time, 200 ms) was acquired by the Orbitrap mass spectrometer. MALDI-TOF MS analyses were performed on UltrafleXtreme MALDI TOF/TOF Mass Spectrometer (Bruker). Scan range of MS was set according to the molecular weight of O-glycans, and reflector mode was used for O-glycan analysis. Mass spectra were obtained in both positive and negative extraction mode with the following voltage settings: ion source 1 (19.0 kV), ion source 2 (15.9 kV), and lens (9.3 kV). The reflector voltage was set to 20 kV. The laser was pulsed at 7 Hz and the pulsed ion extraction time was set at 400 ns. The laser power was kept in the range of 40–90%. Anhydrous dichloromethane, TMSOTf, PTSA.H_2O, solid sodium methoxide and FmocOSu was purchased from Sigma Aldrich. TFA was purchased from Alfa Aesar. ^{1}H and ^{13}C NMR spectra were recorded on a Bruker AVANCE 400 (400 MHz) or Bruker AVANCE 600 (600 MHz) spectrometer at 25 °C. All ^{1}H Chemical shifts (in ppm) were assigned according to $CDCl_3$ (δ= 7.24 ppm) and D_2O (δ = 4.79 ppm) and all ^{13}C NMR was calibrated with $CDCl_3$ (*δ* = 77.00 ppm).

Chemical procedures with analytical data for the synthesis of O-GalNAc cores 1–4 and 6

Compound 7:

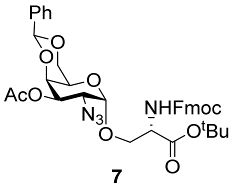

7

The mixture of thio glycosyl donor **10**[1] (24.6 g, 55.3 mmol) and Fmoc-Ser-O^{t}Bu[2] (16.4 g, 48.3 mmol) acceptor was co-evaporated with anhydrous toluene twice (2×30 mL) and dried under high vacuum for a period of 3 h. The above mixture was dissolved in anhydrous dichloromethane (250 mL). To the above solution, NIS (16.3 g, 72.5 mmol) and TMSOTf (2.2 g, 9.6 mmol) was added successively at room temperature and stirred until completion. The reaction was quenched with aq. $NaHCO_3$ (5 mL) slowly and washed with 400 mL of 1:1 aq. $NaHCO_3/Na_2S_2O_3$. The aq. layer was extracted with dichloromethane (2×150 mL) and finally the combined organic layers was washed the brine and concentrated. The crude liquid thus obtained was purified by silica gel flash column chromatography to obtain the α-glycosyl amino acid **7** (20.9 g, 70%). ^{1}H NMR (400 MHz, $CDCl_3$) δ 7.81 (d, J = 7.5 Hz, 2H), 7.67 (t, J = 6.3 Hz, 2H), 7.54 (dd, J = 7.3, 1.7 Hz, 2H), 7.41 (td, J = 13.3, 7.5 Hz, 7H), 5.92 (d, J = 7.7 Hz, 1H), 5.53 (s, 1H), 5.31 (dd, J = 11.1, 2.9 Hz, 1H), 5.08 (d, J = 3.0 Hz, 1H), 4.51 – 4.38 (m, 4H), 4.31 – 4.21 (m, 2H), 4.15 (dd, J = 10.6, 2.7 Hz, 1H), 4.06 – 3.93 (m, 3H), 3.83 (s, 1H), 2.20 (s, 3H), 1.56 (s, 9H). ^{13}C NMR (101 MHz, CDCl3) δ 170.25, 168.61, 155.76, 143.66, 143.61, 141.14, 141.11, 137.30, 128.95, 128.06, 127.60, 127.00, 126.97, 125.95, 125.03, 124.97, 119.88, 100.49, 99.56, 82.91, 77.20, 73.09, 69.65, 69.06, 68.78, 67.12, 62.80, 57.05, 54.82, 46.89, 27.80, 20.81. HRMS calcd for **7** $C_{37}H_{40}N_4O_{10}$ $[M + Na]^+$ *m/z* = 723.2642, found: 723.2644.

Compound 11:

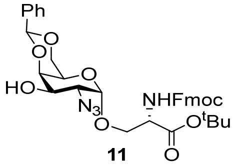

To a solution of compound **7** (10 g, 14.2 mmol) in anhydrous methanol (100 mL), solid sodium methoxide was added until the pH reaches 8.5 at 0 °C and stirred at room temperature until completion. The reaction was neutralized using Amberlite® IRC120 H acidic resin and filtered over Celite® 545. The solution was concentrated and purified by silica gel flash column chromatography to obtain compound **11** (8.9 g, 95%) as a white solid. ^{1}H NMR (400 MHz, $CDCl_3$) δ 7.75 (d, J = 7.5 Hz, 2H), 7.60 (t, J = 7.0 Hz, 2H), 7.47 (dd, J = 6.6, 3.0 Hz, 2H), 7.38 (dd, J = 12.7, 5.3 Hz, 3H), 7.36 – 7.29 (m, 4H), 5.86 (d, J = 7.8 Hz, 1H), 5.50 (s, 1H), 4.95 (d, J = 3.1 Hz, 1H), 4.42 (dd, J = 10.5, 7.1 Hz, 2H), 4.33 (dd, J = 10.4, 7.3 Hz, 1H), 4.21 (dd, J = 8.4, 5.1 Hz, 3H), 4.12 – 4.02 (m, 3H), 3.96 (dd, J = 10.7, 2.6 Hz, 1H), 3.90 (d, J = 12.5 Hz, 1H), 3.72 (s, 1H), 3.58 (dd, J = 10.6, 3.4 Hz, 1H), 1.50 (s, 9H). ^{13}C NMR (101 MHz, $CDCl_3$) δ 168.82, 155.92, 143.86, 143.74, 141.33, 137.32, 129.37, 128.35, 127.82, 127.17, 127.14, 126.23, 125.18, 125.07, 120.10, 101.17, 99.88, 83.10, 77.35, 75.37, 69.68, 69.06, 67.22, 63.27, 60.61, 54.99, 47.09, 27.99. HRMS calcd for **11** $C_{35}H_{38}N_4O_9$ $[M + Na]^+$ *m/z* = 681.2536, found: 681.2504.

Compound 12:

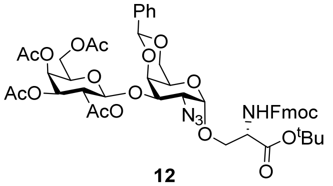

A mixture of glycosyl donor **8**[3] (1.23 g, 2.5 mmol) and glycosyl acceptor **11** (1.5 g, 2.3 mmol) was co-evaporated with anhydrous toluene (2×5 mL) and dried under high vacuum over 6 h. The mixture was dissolved in anhydrous dichloromethane (30 mL) and powdered dry 4 Å molecular sieves (2 g) was added and stirred for 1 h under argon atmosphere. The solution was cooled to -78 °C and TMSOTf (50.4 mg, 0.2 mmol) was added slowly using a micro syringe. The reaction was stirred for 1 h and quenched with 50 µL of DIPEA. The reaction was allowed to reach room temperature over 30 min, filtered over Celite® 545 and concentrated. The crude thus obtained was purified by silica gel flash column chromatography to obtain the disaccharide **12** (1.8 g, 80%). ^{1}H NMR (400 MHz, $CDCl_3$) δ 7.77 (d, J = 7.5 Hz, 2H), 7.63 – 7.57 (m, 2H), 7.52 – 7.47 (m, 2H), 7.40 (t, J = 7.4 Hz, 2H), 7.33 (dd, J = 16.6, 7.5 Hz, 5H), 5.78 (d, J = 7.8 Hz, 1H), 5.50 (s, 1H), 5.34 (d, J = 2.9 Hz, 1H), 5.25 (dd, J = 10.3, 8.0 Hz, 1H), 5.01 – 4.95 (m, 2H), 4.64 (d, J = 7.9 Hz, 1H), 4.45 – 4.38 (m, 2H), 4.35 (dd, J = 11.1, 5.1 Hz, 2H), 4.21 (dd, J = 13.5, 9.9 Hz, 2H), 4.14 – 4.03 (m, 3H), 4.02 – 3.91 (m, 3H), 3.84 – 3.73 (m, 2H), 3.71 (s, 1H), 2.13 (s, 3H), 2.03 (s, 3H), 1.99 (s, 3H), 1.97 (s, 3H), 1.50 (s, 9H). ^{13}C NMR (101 MHz, $CDCl_3$) δ 170.27, 170.19, 170.11, 169.35, 168.72, 155.82, 143.65, 141.25, 137.51, 128.86, 128.10, 127.82, 127.12, 126.05, 125.07, 125.02, 120.09, 102.38, 100.55, 99.92, 83.04, 75.69, 75.53, 70.93, 70.81, 69.51, 68.97, 68.55, 67.16, 66.90, 63.47, 61.40, 58.70, 54.95, 47.03, 27.92, 20.65, 20.54. HRMS calcd for **12** $C_{49}H_{56}N_4O_{18}$ $[M + H]^+$ *m/z* = 989.3668, found: 989.3658.

Compound 13:

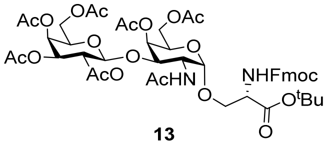

To a solution of compound **12** (1.5 g, 1.5 mmol) in anhydrous methanol, PTSA.H_2O (57.7 mg, 0.3 mmol) was added and stirred at 30 °C until completion. The reaction was quenched with DIPEA (50 µL), concentrated and dried under vacuum for 2 h. The crude was dissolved in a mixture of anhydrous dichloromethane (8 mL), AcOH (2 mL) and activated Zn (0.9 g, 15.0 mmol) dust was added and stirred until completion. The reaction mixture was filtered over Celite® 545 and concentrated to dryness and dried over high vacuum for 2 h. The crude was dissolved in pyridine (5 mL) and acetic anhydride (2 mL) was added slowly over 10 min at 0 °C. The reaction was allowed to stir at room temperature until completion. The solvent was evaporated and dissolved in EtOAc (150 mL) and washed with aq. $NaHCO_3$ (50 mL), 1N HCl (50 mL), and brine (50 mL). The organic layer was dried over anhydrous Na_2SO_4 and concentrated. The crude thus obtained was purified using silica gel flash column chromatography to obtain protected disaccharide **13** (1 g, 70%). ^{1}H NMR (400 MHz, $CDCl_3$) δ 7.76 (d, J = 7.5 Hz, 2H), 7.57 (d, J = 7.4 Hz, 2H), 7.40 (t, J = 7.4 Hz, 2H), 7.31 (t, J = 7.4 Hz, 2H), 5.76 (d, J = 8.8 Hz, 1H), 5.69 (s, 1H), 5.33 (s, 2H), 5.13 – 5.05 (m, 1H), 4.93 (d, J = 10.8 Hz, 1H), 4.84 (s, 1H), 4.58 – 4.33 (m, 5H), 4.22 (t, J = 6.6 Hz, 1H), 4.14 – 4.08 (m, 3H), 4.02 (d, J = 7.3 Hz, 1H), 3.98 – 3.90 (m, 2H), 3.84 (d, J = 6.0 Hz, 2H), 3.73 (s, 1H), 2.14 (s, 3H), 2.11 (s, 3H), 2.04 (s, 3H), 2.00 (s, 6H), 1.95 (s, 3H), 1.92 (s, 3H), 1.47 (s, 9H). ^{13}C NMR (101 MHz, $CDCl_3$) δ 170.49, 170.40, 170.35, 170.13, 170.09, 169.68, 169.05, 155.81, 143.65, 141.32, 141.29, 127.87, 127.10, 124.86, 120.11, 100.54, 98.53, 83.13, 77.20, 73.04, 70.86, 70.71, 68.65, 68.57, 67.81, 67.16, 66.72, 62.65, 61.06, 54.82, 48.65, 47.08, 28.00, 23.24, 20.70, 20.66, 20.54. HRMS calcd for **13** $C_{48}H_{60}N_2O_{21}$ $[M + Na]^+$ m/z = 1023.3587, found: 1023.3570.

Compound 1 (core 1):

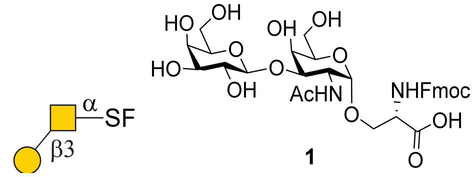

To the purified compound **13** (0.5 g, 0.5 mmol), a mixture of TFA/Anisole (9:1) (5 mL) was added at 0 °C and stirred until completion (15 min). The reaction mixture was concentrated below 30 °C and co-evaporated with toluene (2×5 mL) and dried under high vacuum for 1 h. The crude was dissolved in anhydrous MeOH (5 mL) and solid NaOMe was added slowly at 0 °C until the pH reached 8.5. The reaction was allowed to stir at room temperature until completion. The reaction was neutralized using Amberlite® IRC120 H acidic resin and filtered over Celite® 545. The solution was concentrated and purified by C18 reverse phase flash column chromatography using H_2O and acetonitrile to obtain compound **1** (0.27 g, 78% over two steps) as a white solid. ^{1}H NMR (400 MHz, D_2O) δ 7.76 (d, J = 7.3 Hz, 2H), 7.60 (dd, J = 14.3, 7.3 Hz, 2H), 7.36 (dt, J = 15.3, 7.3 Hz, 4H), 4.44 (dd, *J* = 9.9, 5.9 Hz, 2H), 4.31 (d, *J* = 7.4 Hz, 1H), 4.25 (dd, *J* = 11.0, 3.6 Hz, 1H), 4.10 (d, *J* = 17.3 Hz, 3H), 3.85 (dt, *J* = 20.1, 12.7 Hz, 4H), 3.75 – 3.39 (m, 9H), 1.93 (s, 3H). ^{13}C NMR (101 MHz, D_2O) δ 176.24, 174.53, 157.47, 143.75, 140.86, 127.92, 127.41, 127.37, 124.91, 120.08, 104.62, 97.88, 77.25, 74.73, 72.45, 70.61, 70.53, 68.85, 68.60, 68.49, 66.26, 60.99, 60.78, 56.23, 48.39, 46.76, 22.07. HRMS (m/z): calcd for $C_{32}H_{40}N_2O_{15}$ $[M-H]^-$ 691.2429; found $[M-H]^-$ 691.2433. Compound was characterized by HPLC, T_R =19.859 min.

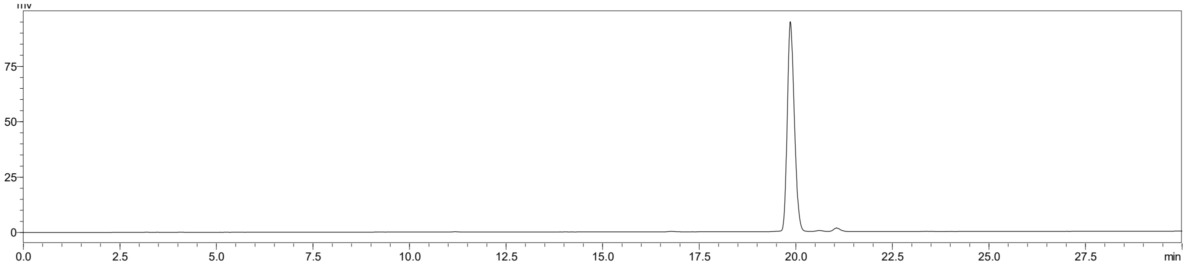

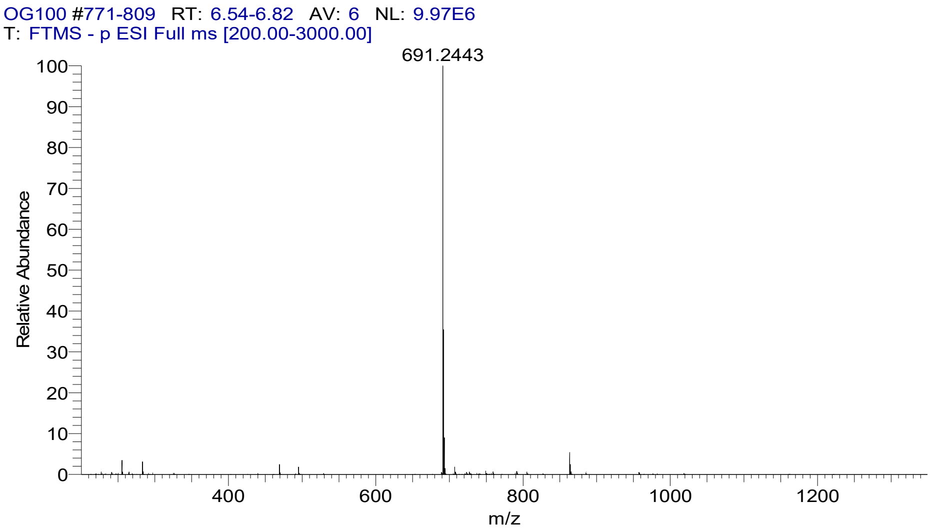

Compound 14:

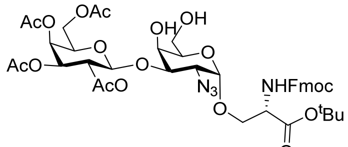

14

To a solution of compound **12** (1.5 g, 1.5 mmol) in anhydrous methanol, PTSA.H_2O (57.7 mg, 0.3 mmol) was added and stirred at 30 °C until completion. The reaction was quenched with DIPEA (50 μL), concentrated and purified by silica gel flash column chromatography to obtain diol **14** (1.2 g, 90%). ^{1}H NMR (400 MHz, $CDCl_3$) δ 7.76 (d, *J* = 7.5 Hz, 2H), 7.61 (d, *J* = 7.4 Hz, 2H), 7.40 (t, *J* = 7.4 Hz, 2H), 7.31 (t, *J* = 7.4 Hz, 2H), 5.95 (d, *J* = 7.9 Hz, 1H), 5.35 (s, 1H), 5.24 (dd, *J* = 10.3, 8.0 Hz, 1H), 4.98 (dd, *J* = 10.5, 3.3 Hz, 1H), 4.88 (d, *J* = 3.2 Hz, 1H), 4.58 (d, *J* = 8.0 Hz, 1H), 4.48 – 4.32 (m, 3H), 4.20 (t, *J* = 6.8 Hz, 1H), 4.13 – 4.00 (m, 4H), 3.93 (dd, *J* = 24.1, 10.8 Hz, 2H), 3.87 – 3.78 (m, 3H), 3.73 (dd, *J* = 14.2, 7.5 Hz, 1H), 3.60 (dd, *J* = 10.5, 3.4 Hz, 1H), 2.14 (s, 3H), 2.05 (s, 3H), 1.98 (d, *J* = 5.8 Hz, 6H), 1.49 (s, 9H). ^{13}C NMR (101 MHz, $CDCl_3$) δ 170.40, 170.10, 170.05, 169.54, 168.87, 155.86, 143.81, 143.68, 141.33, 127.78, 127.12, 125.16, 120.07, 101.78, 99.67, 83.06, 78.08, 77.20, 71.28, 70.61, 70.05, 69.91, 69.03, 68.28, 66.93, 62.66, 61.55, 58.41, 55.02, 47.17, 27.93, 20.62, 20.53. HRMS calcd for **14** $C_{42}H_{52}N_4O_{18}$ $[M + Na]^+$ *m/z* = 923.3175, found: 923.3157.

Compound 15:

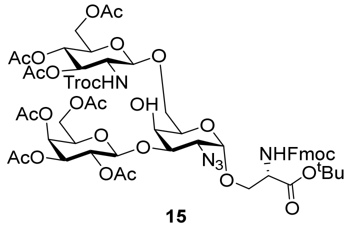

15

A mixture of glycosyl donor **9**[4] (1.1 g, 1.8 mmol) and glycosyl acceptor **14** (1.5 g, 1.6 mmol) was co-evaporated with anhydrous toluene (2×5 mL) and dried under high vacuum over 6 h. The mixture was dissolved in anhydrous dichloromethane (30 mL) and powdered dry 4 Å molecular sieves (2 g) was added and stirred for 1 h under argon atmosphere. The solution was cooled to -40 °C and TMSOTf (53.3 mg, 0.2 mmol) was added slowly. The reaction was allowed to stir for 1 h and quenched with DIPEA (50 μL). The reaction was allowed to reach room

temperature, filtered over Celite® 545 and concentrated. The crude thus obtained was purified by silica gel flash column chromatography to obtain the trisaccharide **15** (1.8 g, 82%). ^{1}H NMR (400 MHz, $CDCl_3$) δ 7.80 (d, *J* = 7.5 Hz, 2H), 7.65 (d, *J* = 7.4 Hz, 2H), 7.50 – 7.31 (m, 4H), 6.19 (d, *J* = 7.5 Hz, 1H), 5.92 (d, *J* = 7.3 Hz, 1H), 5.65 (t, *J* = 10.0 Hz, 1H), 5.40 (d, *J* = 3.3 Hz, 1H), 5.26 (dd, *J* = 10.5, 7.9 Hz, 1H), 5.12 – 4.94 (m, 3H), 4.91 – 4.75 (m, 3H), 4.64 (dd, *J* = 13.6, 9.9 Hz, 2H), 4.51 – 4.39 (m, 2H), 4.28 (t, *J* = 7.1 Hz, 1H), 4.22 – 4.02 (m, 6H), 4.02 – 3.86 (m, 5H), 3.79 – 3.65 (m, 2H), 3.60 (dd, *J* = 10.5, 3.7 Hz, 1H), 3.36 (q, *J* = 8.8 Hz, 1H), 2.17 (s, 3H), 2.08 (s, 3H), 2.06 – 1.98 (m, 15H), 1.52 (s, 9H). ^{13}C NMR (101 MHz, $CDCl_3$) δ 170.50, 170.29, 170.06, 169.97, 169.56, 169.47, 168.59, 155.72, 153.76, 143.82, 143.47, 141.30, 141.22, 127.84, 127.74, 127.19, 127.02, 124.99, 120.10, 101.70, 100.01, 98.15, 95.54, 83.19, 78.00, 77.32, 77.20, 77.00, 76.68, 74.22, 71.51, 71.09, 70.89, 70.61, 69.77, 69.26, 69.01, 68.26, 67.99, 67.08, 66.76, 62.06, 61.23, 60.32, 58.30, 56.86, 54.47, 47.03, 27.87, 20.57, 20.56, 20.48. . HRMS calcd for **15** $C_{57}H_{70}Cl_3N_5O_{27}$ $[M + Na]^+$ *m/z* =1384.3222, found: 1384.3201.

Compound 16:

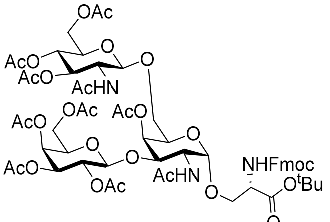

16

Compound **15** (1 g, 0.7 mmol) was dissolved in a mixture of anhydrous dichloromethane (15 mL), AcOH (5 mL) and activated Zn (0.72 g, 11.0 mmol) dust was added and stirred at 40 °C until completion (12 h). The reaction mixture was filtered over Celite® 545 and concentrated to dryness and dried over high vacuum for 2 h. The crude was dissolved in pyridine (10 mL) and acetic anhydride (5 mL) was added slowly over 10 min at 0 °C. The reaction was allowed to stir at room temperature until completion. The solvent was evaporated and dissolved in EtOAc (150 mL) and washed with aq. $NaHCO_3$ (50 mL), 1N HCl (50 mL), and brine (50 mL). The organic layer was dried over anhydrous Na_2SO_4 and concentrated. The crude thus obtained was purified using silica gel flash column chromatography to obtain protected trisaccharide **16** (0.8 g, 85%). ^{1}H NMR (600 MHz, $CDCl_3$) δ 7.76 (d, *J* = 7.2 Hz, 2H), 7.60 (d, *J* = 4.9 Hz, 2H), 7.39 (t, *J* = 7.1 Hz, 2H), 7.31 (t, *J* = 6.7 Hz, 2H), 6.11 (d, *J* = 6.5 Hz, 1H), 6.00 (d, *J* = 7.1 Hz, 1H), 5.81 (d, *J* = 8.4 Hz, 1H), 5.44 (t, *J* = 9.3 Hz, 1H), 5.32 (d, *J* = 2.6 Hz, 1H), 5.24 (s, 1H), 5.06 (dd, *J* = 10.0, 8.2 Hz, 1H), 5.01 – 4.88 (m, 2H), 4.84 – 4.69 (m, 2H), 4.53 (d, *J* = 7.8 Hz, 1H), 4.47 (s, 2H), 4.36 (d, *J* = 4.2 Hz, 2H), 4.23 (d, *J* = 6.6 Hz, 2H), 4.12 – 4.00 (m, 3H), 3.93 (s, 2H), 3.88 – 3.78 (m, 3H), 3.62 (dd, *J* = 44.9, 9.1 Hz, 3H), 3.43 – 3.35 (m, 1H), 2.13 (s, 3H), 2.09 (s, 3H), 2.03 (s, 3H), 2.02 – 1.97 (m, 12H), 1.94 (d, *J* = 10.7 Hz, 6H), 1.88 (s, 3H), 1.47 (s, 9H). ^{13}C NMR (151 MHz, $CDCl_3$) δ 170.64, 170.46, 170.39, 170.29, 170.23, 170.11, 169.69, 169.48, 169.27, 156.00, 143.67, 141.29, 127.80, 127.14, 124.95, 120.06, 100.65, 100.15, 97.85, 82.90, 77.21, 77.00, 76.79, 73.24, 71.63, 70.81, 70.68, 69.09, 68.79, 68.69, 67.07, 66.70, 61.97, 61.00, 55.58, 54.73, 48.69, 47.05, 28.00, 23.27, 20.74, 20.69, 20.65, 20.62, 20.51. HRMS calcd for **16** $C_{60}H_{77}N_3O_{28}$ $[M + Na]^+$ *m/z* = 1310.4592, found: 1310.4570.

Compound 2 (core 2):

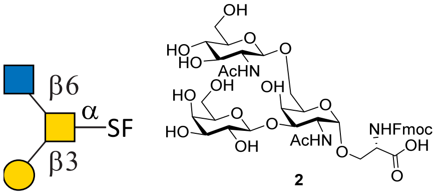

2

To the purified compound **16** (0.7 g, 0.5 mmol), a mixture of TFA/Anisole (9:1) (7 mL) was added at 0 °C and stirred until completion (15 min). The reaction mixture was concentrated below 30 °C and co-evaporated with toluene (2×5 mL) and dried under high vacuum for 1 h. The crude was dissolved in anhydrous MeOH (5 mL) and solid NaOMe was added slowly at 0 °C until the pH reached 8.5. The reaction was allowed to stir at room temperature until completion. The reaction was neutralized using Amberlite® IRC120 H acidic resin and filtered over Celite® 545. The solution was concentrated and dissolved in a mixture of acetone (6 mL) and H_2O (3 mL). To the vigorously stirring solution, $NaHCO_3$ (63 mg, 0.75 mmol) and Fmoc-OSu (168 mg, 0.5 mmol) were added successively and stirred at room temperature until completion. The solution was concentrated and purified by C18 reverse phase flash column chromatography using H_20 and acetonitrile to obtain compound **2** (0.37 g, 77% over three steps) as a white solid. ^{1}H NMR (600 MHz, D_2O) δ 7.86 (t, *J* = 7.7 Hz, 2H), 7.73 – 7.61 (m, 2H), 7.48 (dt, *J* = 14.7, 7.4 Hz, 2H), 7.45 – 7.36 (m, 2H), 4.77 (d, *J* = 2.9 Hz, 3H), 4.58 (dd, *J* = 10.7, 5.6 Hz, 1H), 4.49 (d, *J* = 8.3 Hz, 2H), 4.37 (d, *J* = 7.8 Hz, 1H), 4.30 – 4.20 (m, 2H), 4.12 (d, *J* = 9.1 Hz, 2H), 3.91 (dd, *J* = 22.9, 12.6 Hz, 4H), 3.83 – 3.70 (m, 5H), 3.57 (dddd, *J* = 30.7, 25.5, 21.8, 10.6 Hz, 7H), 3.36 (d, *J* = 3.8 Hz, 2H), 1.97 (s, 6H). ^{13}C NMR (151 MHz, D_2O) δ 176.26, 174.57, 174.27, 157.45, 143.88, 140.93, 128.02, 127.52, 125.02, 120.18, 120.13, 104.63, 101.23, 97.89, 76.87, 75.80, 74.82, 73.81, 72.49, 70.57, 69.93, 69.37, 69.03, 68.79, 68.53, 66.31, 60.84, 60.69, 56.13, 55.66, 48.37, 46.90, 22.21, 22.11. HRMS, calcd for $C_{40}H_{53}N_3O_{20}$ 895.3222; found $[M-H]^-$ 894.3261. Compound was characterized by HPLC, T_R =15.749 min.

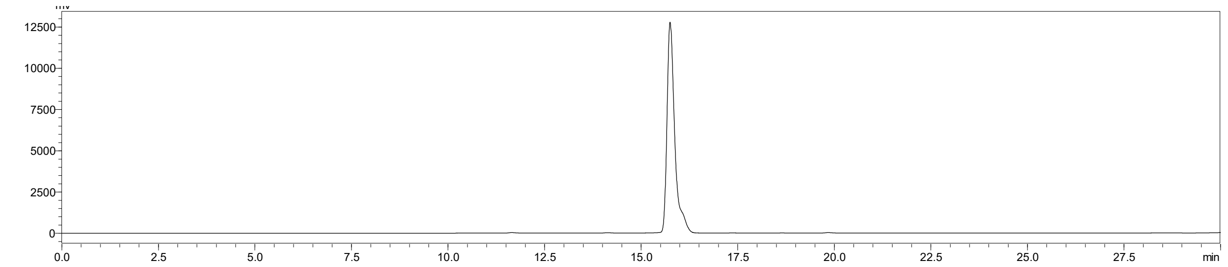

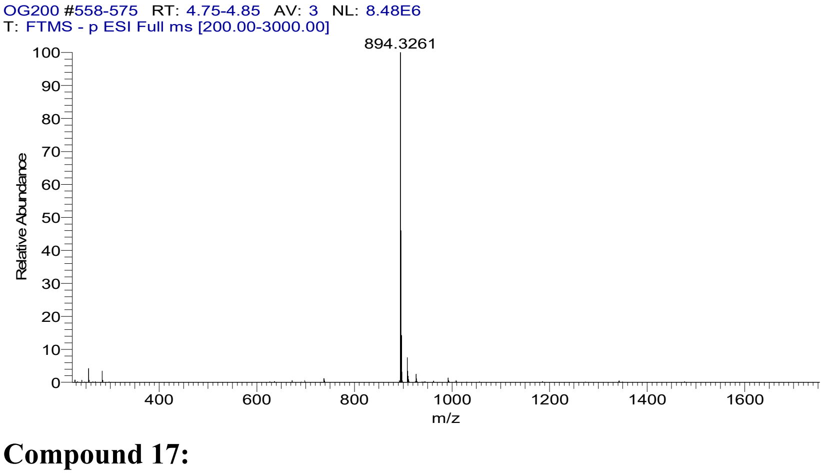

Compound 17:

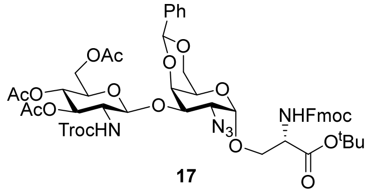

17

A mixture of glycosyl donor **9**[4] (1.6 g, 2.6 mmol) and glycosyl acceptor **11** (1.4 g, 2.1 mmol) was co-evaporated with anhydrous toluene (2×5 mL) and dried under high vacuum over 6 h. The mixture was dissolved in anhydrous dichloromethane (30 mL) and powdered dry 4 Å molecular sieves (2 g) was added and stirred for 1 h under argon atmosphere. The solution was cooled to -78 ºC and TMSOTf (94 mg, 0.4 mmol) was added slowly. The reaction

was allowed to stir for 1 h and quenched with DIPEA (100 µL). The reaction was allowed to reach room temperature, filtered over Celite® 545 and concentrated. The crude thus obtained was purified by silica gel flash column chromatography to obtain the disaccharide **17** (2.1 g, 88%). ^{1}H NMR (400 MHz, $CDCl_3$) δ 7.80 (d, *J* = 7.4 Hz, 2H), 7.62 (dd, *J* = 7.1, 4.2 Hz, 2H), 7.47 (d, *J* = 6.4 Hz, 2H), 7.41 (t, *J* = 7.7 Hz, 2H), 7.37 – 7.30 (m, 5H), 5.74 (d, *J* = 8.0 Hz, 1H), 5.50 (s, 1H), 5.21 (t, *J* = 9.9 Hz, 1H), 5.02 – 4.91 (m, 3H), 4.70 (d, *J* = 12.0 Hz, 1H), 4.59 (dd, *J* = 23.4, 10.1 Hz, 2H), 4.48 – 4.40 (m, 2H), 4.37 (d, *J* = 2.2 Hz, 1H), 4.34 – 4.16 (m, 4H), 4.05 – 3.96 (m, 3H), 3.95 – 3.91 (m, 1H), 3.86 (dd, *J* = 10.7, 3.2 Hz, 1H), 3.74 (s, 1H), 3.59 (dd, *J* = 18.5, 8.8 Hz, 1H), 3.36 (d, *J* = 8.3 Hz, 1H), 2.01 (s, 3H), 1.98 (d, *J* = 4.9 Hz, 6H), 1.49 (s, 10H). ^{13}C NMR (101 MHz, $CDCl_3$) δ 170.42, 170.31, 169.40, 168.63, 155.89, 153.86, 143.75, 143.48, 141.24, 137.57, 128.87, 128.10, 127.89, 127.27, 127.18, 126.05, 125.34, 125.10, 120.28, 101.64, 100.55, 99.51, 95.28, 83.09, 76.34, 75.39, 74.50, 71.67, 71.58, 69.44, 68.99, 68.28, 67.21, 63.46, 61.40, 58.69, 56.09, 54.90, 47.06, 27.94, 20.71, 20.62, 20.59. HRMS calcd for **17** $C_{50}H_{56}Cl_3N_5O_{18}$ $[M + H]^+$ *m/z* = 1120.2764, found: 1120.2750.

Compound 18:

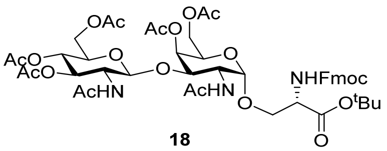

18

To a solution of compound **17** (2.0 g, 1.7 mmol) in anhydrous methanol (20 mL), PTSA.H_2O (68 mg, 0.3 mmol) was added and stirred at 30 °C until completion. The reaction was quenched with DIPEA (60 µL), concentrated and dried under vacuum for 2 h. The crude was dissolved in a mixture of anhydrous dichloromethane (10 mL), AcOH (5 mL) and activated Zn (1.6 g, 25.5 mmol) dust was added and stirred at 40 °C until completion. The reaction mixture was filtered over Celite® 545 and concentrated to dryness and dried over high vacuum for 2 h. The crude was dissolved in pyridine (10 mL) and acetic anhydride (5 mL) was added slowly over 10 min at 0 °C. The reaction was allowed to stir at RT until completion. The solvent was evaporated and dissolved in EtOAc (150 mL) and washed with aq. $NaHCO_3$ (50 mL), 1N HCl (50 mL), and brine (50 mL). The organic layer was dried over anhydrous Na_2SO_4 and concentrated. The crude thus obtained was purified using silica gel flash column chromatography to obtain protected disaccharide **18** (1.4 g, 79%). ^{1}H NMR (600 MHz, $CDCl_3$) δ 7.73 (d, *J* = 7.2 Hz, 2H), 7.57 (s, 2H), 7.36 (t, *J* = 7.3 Hz, 2H), 7.28 (t, *J* = 7.3 Hz, 2H), 6.18 (d, *J* = 7.7 Hz, 1H), 6.02 (d, *J* = 5.5 Hz, 1H), 5.85 (d, *J* = 8.0 Hz, 1H), 5.31 (dd, *J* = 21.6, 6.4 Hz, 2H), 5.00 (t, *J* = 9.7 Hz, 1H), 4.89 (dd, *J* = 11.6, 5.7 Hz, 2H), 4.48 – 4.41 (m, 1H), 4.37 (d, *J* = 4.2 Hz, 3H), 4.26 – 4.16 (m, 2H), 4.11 – 4.05 (m, 2H), 4.03 – 3.98 (m, 1H), 3.95 – 3.83 (m, 3H), 3.77 (d, *J* = 9.0 Hz, 1H), 3.63 (d, *J* = 9.5 Hz, 1H), 3.51 (d, *J* = 8.8 Hz, 1H), 2.06 (s, 3H), 2.03 (s, 3H), 1.99 – 1.97 (m, 9H), 1.92 (s, 3H), 1.90 (s, 3H), 1.44 (s, 9H). ^{13}C NMR (151 MHz, $CDCl_3$) δ 171.11, 170.73, 170.62, 170.41, 169.89, 169.23, 168.94, 155.85, 143.63, 141.17, 127.73, 127.02, 124.85, 119.98, 99.25, 98.34, 82.88, 72.88, 71.87, 68.87, 68.55, 68.32, 67.57, 66.95, 62.50, 61.35, 55.31, 54.79, 48.81, 47.01, 27.90, 23.22, 23.12, 20.60, 20.55, 20.51. HRMS calcd for **18** $C_{48}H_{61}N_3O_{20}$ $[M+H]^+$ *m/z* = 1000.3927, found: 1000.3925.

Compound 3 (core 3):

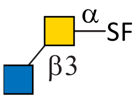

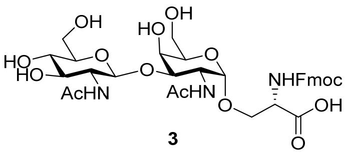

3

To the purified compound **18** (1.0 g, 1.0 mmol), a mixture of TFA/Anisole (9:1) (10 mL) was added at 0 °C and stirred until completion (15 min). The reaction mixture was concentrated below 30 °C and coevaporated with toluene (2×5 mL) and dried under high vacuum for 1 h. The crude was dissolved in anhydrous MeOH (5 mL) and solid NaOMe was added slowly at 0 °C until the pH reached 8.5. The reaction was allowed to stir at room temperature until completion. The reaction was neutralized using Amberlite® IRC120 H acidic resin and filtered over Celite® 545. The solution was concentrated and purified by C18 reverse phase flash column chromatography

using H_2O and acetonitrile to obtain compound **3** (0.6 g, 90% over two steps) as a white solid. ^{1}H NMR (400 MHz, D_2O) δ 7.31 – 7.12 (m, 4H), 7.08 – 6.89 (m, 4H), 4.65 (s, 1H), 4.38 (d, *J* = 8.1 Hz, 1H), 4.06 (dd, *J* = 41.3, 11.9 Hz, 4H), 3.83 – 3.33 (m, 13H), 3.17 (s, 1H), 1.91 (d, *J* = 7.6 Hz, 6H). ^{13}C NMR (101 MHz, D_2O) δ 176.18, 174.33, 173.60, 157.18, 143.45, 140.69, 127.66, 127.16, 124.76, 119.85, 102.41, 97.84, 76.84, 75.43, 73.54, 70.42, 69.31, 68.59, 61.04, 60.10, 56.18, 55.49, 48.89, 48.20, 46.55, 22.28, 22.22. HRMS, calcd $C_{34}H_{43}N_3O_{15}$ for : 733.2694; found $[M+Na]^+$ 756.3203.

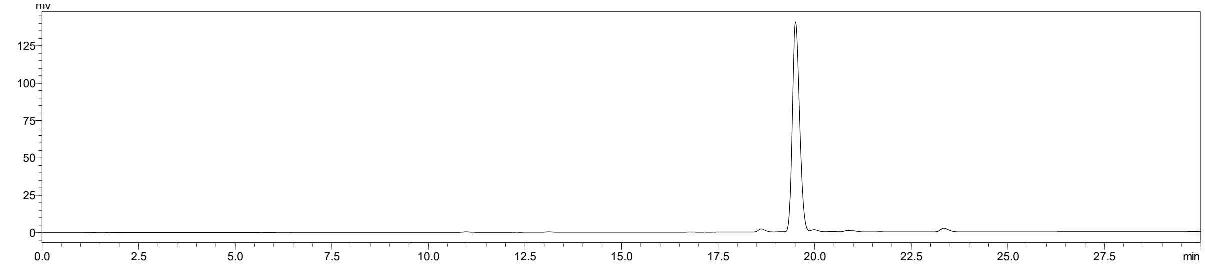

Compound was characterized by HPLC, T_R =19.507 min

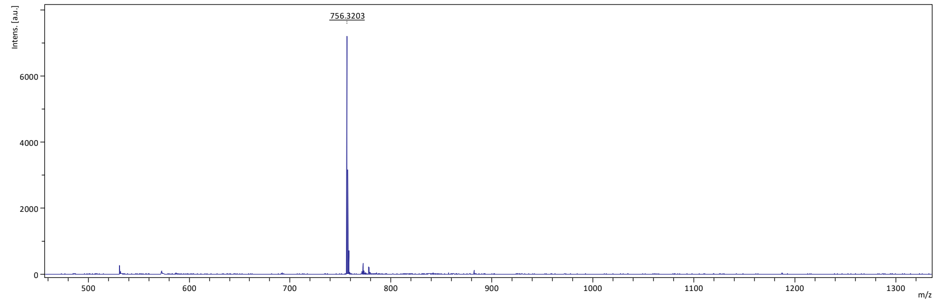

Compound 19:

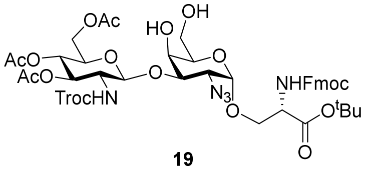

19

To a solution of compound **17** (3.0 g, 2.6 mmol) in anhydrous methanol (20 mL), PTSA.H_2O (101 mg, 0.5 mmol) was added and stirred at 30 °C until completion. The reaction was quenched with DIPEA (100 µL), concentrated and purified by silica gel flash column chromatography to obtain diol **19** (2.3 g, 85%). ^{1}H NMR (400 MHz, $CDCl_3$) δ 7.76 (d, *J* = 7.4 Hz, 2H), 7.61 (t, *J* = 7.9 Hz, 2H), 7.39 (dd, *J* = 13.8, 6.9 Hz, 2H), 7.31 (dt, *J* = 11.6, 5.9 Hz, 2H), 5.97 (d, *J* = 8.2 Hz, 1H), 5.32 (d, *J* = 8.6 Hz, 1H), 5.24 (t, *J* = 10.0 Hz, 1H), 4.97 – 4.85 (m, 2H), 4.71 (d, *J* = 11.5 Hz, 2H), 4.58 (d, *J* = 12.0 Hz, 1H), 4.49 – 4.40 (m, 2H), 4.35 – 4.27 (m, 1H), 4.21 (t, *J* = 7.0 Hz, 1H), 4.10 (t, *J* = 9.0 Hz, 3H), 4.02 (dd, *J* = 11.0, 4.0 Hz, 1H), 3.95 – 3.82 (m, 4H), 3.77 – 3.71 (m, 1H), 3.69 – 3.59 (m, 2H), 3.54 – 3.47 (m, 1H), 3.07 (s, 1H), 2.02 (s, 3H), 2.01 (s, 3H), 2.00 (s, 3H), 1.47 (s, 9H). ^{13}C NMR (101 MHz, $CDCl_3$) δ 170.63, 170.33, 169.42, 168.79, 155.88, 154.13, 143.82, 143.52, 141.18, 127.75, 127.14, 125.31, 125.08, 120.09, 100.85, 99.36, 95.23, 82.95, 77.94, 74.46, 71.93, 71.21, 69.91, 68.75, 68.47, 67.00, 62.41, 61.82, 58.47, 56.04, 54.93, 47.08, 27.88, 20.55. HRMS calcd for **19** $C_{43}H_{52}Cl_3N_5O_{18}$ $[M+H]^+$ *m/z* = 1032.2451, found: 1032.2430.

Compound 20:

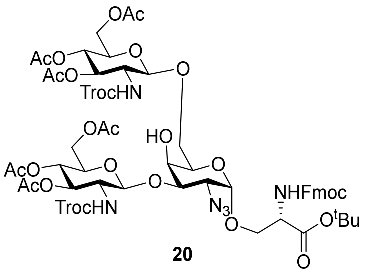

20

A mixture of glycosyl donor **9**[4] (0.6 g, 1.0 mmol) and glycosyl acceptor **19** (1.0 g, 0.97 mmol) was coevaporated with anhydrous toluene (2×5 mL) and dried under high vacuum over 6 h. The mixture was dissolved in anhydrous dichloromethane (20 mL) and powdered dry 4 Å molecular sieves (1.5 g) was added and stirred for 1 h under argon atmosphere. The solution was cooled to -78 °C and TMSOTf (43 mg, 0.19 mmol) was added slowly. The reaction was allowed to stir for 1 h and quenched with DIPEA (40 μL). The reaction was allowed to reach room temperature, filtered over Celite® 545 and concentrated. The crude thus obtained was purified by silica gel flash column chromatography to obtain the trisaccharide **20** (1.0 g, 70%). ^{1}H NMR (400 MHz, $CDCl_3$) δ 7.79 (d, J = 7.4 Hz, 2H), 7.69 – 7.58 (m, 2H), 7.44 – 7.29 (m, 4H), 6.34 (d, J = 7.0 Hz, 1H), 5.91 (d, J = 7.0 Hz, 1H), 5.69 (t, J = 9.7 Hz, 1H), 5.32 (s, 1H), 5.22 (d, J = 8.9 Hz, 1H), 5.18 – 5.10 (m, 1H), 4.99 (ddd, J = 23.7, 14.5, 7.0 Hz, 2H), 4.92 – 4.78 (m, 3H), 4.73 (d, J = 12.1 Hz, 1H), 4.64 – 4.54 (m, 2H), 4.51 – 4.40 (m, 2H), 4.35 (dd, J = 17.6, 7.1 Hz, 1H), 4.23 (ddd, J = 22.3, 19.5, 7.5 Hz, 3H), 4.16 – 4.06 (m, 2H), 4.06 – 3.97 (m, 3H), 3.91 (dd, J = 30.1, 10.3 Hz, 3H), 3.71 – 3.54 (m, 3H), 3.49 (d, J = 8.9 Hz, 1H), 3.30 (dd, J = 18.6, 9.2 Hz, 1H), 3.23 (dd, J = 17.9, 8.2 Hz, 1H), 2.06 (s, 4H), 2.04 (s, 3H), 2.00 (d, J = 5.0 Hz, 9H), 1.96 (s, 3H), 1.49 (s, 9H). ^{13}C NMR (101 MHz, $CDCl_3$) δ 170.74, 170.61, 170.56, 170.11, 169.61, 169.35, 155.87, 154.07, 153.76, 143.81, 143.51, 141.28, 127.85, 127.25, 125.18, 125.00, 120.22, 101.14, 97.73, 95.64, 95.44, 83.24, 77.47, 77.35, 77.15, 76.83, 75.02, 74.90, 74.49, 74.20, 71.74, 71.39, 70.67, 69.75, 69.18, 68.19, 67.25, 61.99, 61.42, 59.41, 56.95, 56.16, 54.47, 53.50, 47.03, 27.95, 20.68, 20.60. HRMS calcd for **20** $C_{58}H_{70}Cl_6N_6O_{27}$ $[M+H]^+$ *m/z* = 1493.2498, found: 1493.2463.

Compound 21:

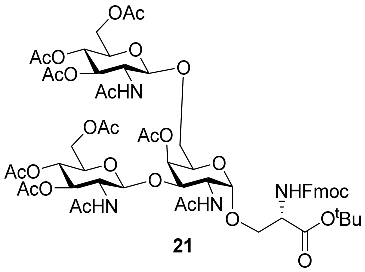

21

Compound **20** (1 g, 0.67 mmol) was dissolved in a mixture of anhydrous dichloromethane (15 mL), AcOH (5 mL) and activated Zn (0.7 g, 10.0mmol) dust was added and stirred at 40 °C until completion. The reaction mixture was filtered over Celite® 545 and concentrated to dryness and dried over high vacuum for 2 h. The crude was dissolved in pyridine (10 mL) and acetic anhydride (5 mL) was added slowly over 10 min at 0 °C. The reaction was allowed to stir at room temperature until completion. The solvent was evaporated and dissolved in EtOAc (150 mL) and washed with aq. $NaHCO_3$ (50 mL), 1N HCl (50 mL), and brine (50 mL). The organic layer was dried over anhydrous Na_2SO_4 and concentrated. The crude thus obtained was purified using silica gel flash column chromatography to obtain protected trisaccharide **21** (0.6 g, 76%). ^{1}H NMR (400 MHz, MeOD) δ 7.81 (d, *J* = 7.6 Hz, 2H), 7.74 – 7.65 (m, 2H), 7.41 (t, *J* = 7.1 Hz, 2H), 7.34 (t, *J* = 7.4 Hz, 2H), 5.49 (dd, *J* = 12.9, 6.8 Hz, 1H), 5.25 (t, *J* = 9.9 Hz, 1H), 5.10 – 4.92 (m, 3H), 4.71 (dd, *J* = 13.1, 5.8 Hz, 2H), 4.55 – 4.36 (m, 3H), 4.36 – 4.27 (m, 2H), 4.27 – 4.19 (m, 3H), 4.19 – 4.06 (m, 3H), 3.98 – 3.83 (m, 4H), 3.78 (dd, *J* = 17.7, 6.4 Hz, 4H), 3.63 – 3.48 (m, 2H), 2.01 (dt, *J* = 12.2, 5.7 Hz, 24H), 1.90 (d, *J* = 11.3 Hz, 6H), 1.47 (s, 9H). ^{13}C NMR (101 MHz,

MeOD) δ 171.86, 171.75, 171.62, 170.81, 170.74, 170.44, 170.38, 169.86, 157.07, 149.85, 148.76, 143.89, 141.26, 137.08, 127.55, 126.95, 124.83, 124.25, 119.76, 101.33, 100.94, 98.37, 82.12, 76.37, 72.68, 71.67, 71.52, 71.37, 71.16, 70.28, 69.62, 68.90, 66.91, 66.54, 62.05, 61.44, 55.47, 55.23, 54.20, 47.07, 29.51, 27.10, 22.14, 21.78, 19.67, 19.53, 19.40, 19.37, 19.34. HRMS calcd for **21** $C_{60}H_{78}N_4O_{27}$ $[M+H]^+$ *m/z* = 1287.4932, found: 1287.4930.

Compound 4 (core 4):

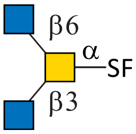

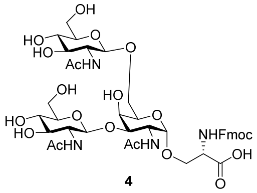

4

To the purified compound **21** (0.5 g, 0.39 mmol), a mixture of TFA/Anisole (9:1) (5 mL) was added at 0 °C and stirred until completion (15 min). The reaction mixture was concentrated below 30 °C and coevaporated with toluene (2×5 mL) and dried under high vacuum for 1 h. The crude was dissolved in anhydrous MeOH (5 mL) and solid NaOMe was added slowly at 0 oC until the pH reached 8.5. The reaction was allowed to stir at room temperature until completion. The reaction was neutralized using Amberlite® IRC120 H acidic resin and filtered over Celite® 545. The solution was concentrated and dissolved in a mixture of acetone (3 mL) and H_2O (1.5 mL). To the vigorously stirring solution, NaHCO3 (49 mg, 0.58 mmol) and FmocOSu (131 mg, 0.39 mmol) were added successively and stirred at room temperature until completion. The solution was concentrated and purified by C18 reverse phase flash column chromatography using H_2O and acetonitrile to obtain compound **4** (0.33 g, 92% over three steps) as a white solid. ^{1}H NMR (600 MHz, D_2O) δ 8.23 (d, J = 7.0 Hz, 2H), 8.04 (s, 2H), 7.82 (d, J = 7.8 Hz, 2H), 7.79 – 7.71 (m, 2H), 4.66 (d, J = 4.5 Hz, 2H), 4.50 (d, J = 7.8 Hz, 2H), 4.44 (s, 1H), 4.32 – 4.06 (m, 7H), 4.03 – 3.91 (m, 4H), 3.85 (t, J = 8.8 Hz, 2H), 3.83 – 3.76 (m, 1H), 3.70 (s, 3H), 2.32 (s, 3H), 2.29 (s, 3H), 2.27 (s, 3H). ^{13}C NMR (101 MHz, D_2O) δ 174.32, 174.11, 173.63, 173.59, 157.23, 143.55, 140.75, 127.82, 127.30, 124.79, 120.00, 102.38, 101.23, 97.77, 75.76, 75.44, 73.78, 73.47, 69.90, 69.37, 69.13, 69.08, 68.73, 66.25, 61.20, 60.66, 60.18, 55.47, 48.09, 46.62, 22.23. HRMS, calcd for $C_{42}H_{56}N_4O_{20}$: 936.3488; found $[M-H]^-$ 935.352. HPLC, T_R =15.324 min.

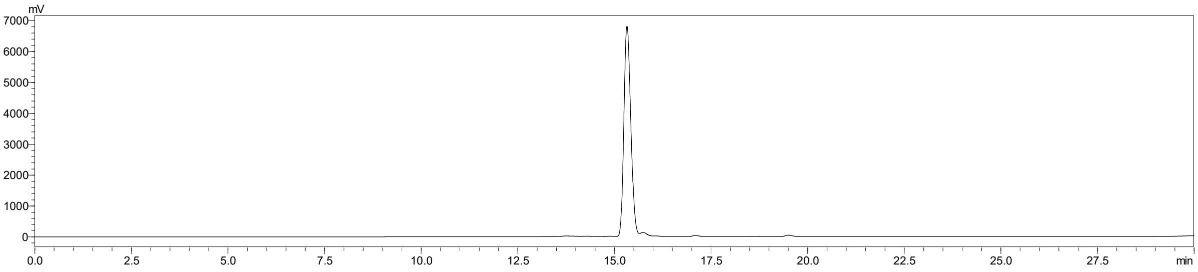

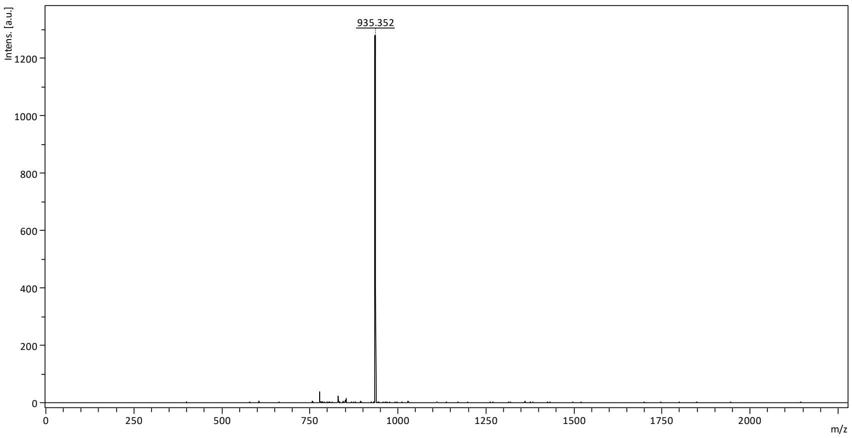

Compound 22:

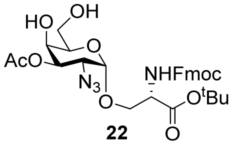

22

To a solution of compound **7** (2.0 g, 3.0 mmol) in anhydrous methanol (20 mL), PTSA.H_2O (115 mg, 0.6 mmol) was added and stirred at 30 °C until completion. The reaction was quenched with DIPEA (100 µL), concentrated and purified by silica gel flash column chromatography to obtain diol **22** (1.71 g, 92%). ^{1}H NMR (600 MHz, $CDCl_3$) δ 7.75 (d, *J* = 7.5 Hz, 2H), 7.61 (d, *J* = 7.4 Hz, 2H), 7.38 (t, *J* = 7.4 Hz, 2H), 7.34 – 7.29 (m, 2H), 6.02 (d, *J* = 8.1 Hz, 1H), 5.15 (dd, *J* = 11.0, 2.8 Hz, 1H), 4.90 (d, *J* = 3.5 Hz, 1H), 4.40 (ddd, *J* = 8.1, 6.1, 2.7 Hz, 3H), 4.23 – 4.18 (m, 2H), 4.16 (dd, *J* = 11.1, 3.0 Hz, 1H), 3.88 (dd, *J* = 10.7, 3.1 Hz, 2H), 3.80 – 3.73 (m, 3H), 2.15 (s, 3H), 1.48 (s, 9H). ^{13}C NMR (151 MHz, $CDCl_3$) δ 169.92, 168.84, 155.92, 143.77, 141.31, 127.69, 127.12, 125.15, 125.10, 119.97, 99.96, 83.15, 70.78, 70.43, 69.80, 68.95, 66.92, 63.24, 57.27, 55.09, 47.13, 27.91, 20.93. HRMS calcd for **22** $C_{30}H_{36}N_4O_{10}$ $[M+H]^+$ *m/z* = 613.2501, found: 613.2492.

Compound 23:

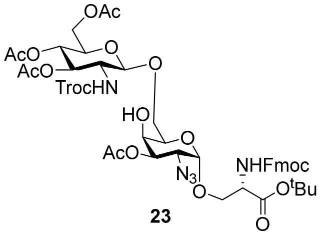

23

A mixture of glycosyl donor **9**[4] (0.71 g, 1.1 mmol) and glycosyl acceptor **22** (0.64 g, 1.0 mmol) was coevaporated with anhydrous toluene (2×5 mL) and dried under high vacuum over 6 h. The mixture was dissolved in anhydrous dichloromethane (20 mL) and powdered dry 4 Å molecular sieves (1.5 g) was added and stirred for 1 h under argon atmosphere. The solution was cooled to -78 °C and TMSOTf (44.4 mg, 0.2 mmol) was added slowly. The reaction was allowed to stir for 1 h and quenched with DIPEA (50 µL). The reaction was allowed to reach room temperature, filtered over Celite® 545 and concentrated. The crude thus obtained was purified by silica gel flash column chromatography to obtain the disaccharide **23** (1.0 g, 89%). ^{1}H NMR (600 MHz, $CDCl_3$) δ 7.76 (d, *J* = 7.5 Hz, 2H), 7.62 (dd, *J* = 13.1, 7.4 Hz, 2H), 7.39 (td, *J* = 7.3, 3.6 Hz, 2H), 7.33 (dd, *J* = 13.7, 6.9 Hz, 2H), 5.89 (d, *J* = 7.2 Hz, 1H), 5.49 (d, *J* = 7.8 Hz, 1H), 5.39 (t, *J* = 9.8 Hz, 1H), 5.16 (dd, *J* = 11.0, 2.7 Hz, 1H), 4.97 – 4.87 (m, 2H), 4.70 (dd, *J* = 23.3, 10.1 Hz, 2H), 4.58 (d, *J* = 12.1 Hz, 1H), 4.46 – 4.38 (m, 2H), 4.34 – 4.29 (m, 1H), 4.25 (t,

J = 7.3 Hz, 1H), 4.14 (d, J = 12.2 Hz, 1H), 4.08 (s, 1H), 4.05 – 3.99 (m, 2H), 3.96 (d, J = 8.4 Hz, 1H), 3.92 (t, J = 5.6 Hz, 1H), 3.90 – 3.85 (m, 1H), 3.74 (d, J = 10.9 Hz, 1H), 3.66 (dd, J = 9.8, 5.7 Hz, 1H), 3.61 – 3.56 (m, 1H), 3.25 (dd, J = 18.2, 8.4 Hz, 1H), 2.16 (s, 3H), 2.01 (s, 3H), 1.99 (s, 3H), 1.98 (s, 3H), 1.49 (s, 9H). ^{13}C NMR (151 MHz, $CDCl_3$) δ 170.76, 170.31, 169.88, 169.46, 168.58, 155.82, 153.92, 143.93, 143.69, 141.23, 141.16, 127.79, 127.25, 125.27, 120.02, 100.21, 98.67, 95.44, 83.20, 74.30, 71.69, 71.06, 70.52, 68.91, 68.68, 68.36, 67.27, 66.69, 61.79, 57.27, 56.26, 54.69, 47.07, 27.93, 20.98, 20.60. HRMS calcd for **23** $C_{45}H_{54}Cl_3N_5O_{19}$ $[M + Na]^+$ m/z = 1096.2377, found: 1096.2356.

Compound 24:

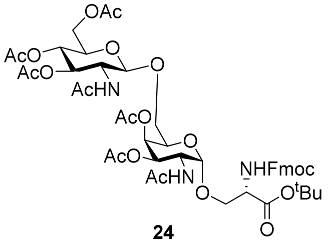

24

Compound **23** (0.85 g, 0.79 mmol) was dissolved in a mixture of anhydrous dichloromethane (15 mL), AcOH (5 mL) and activated Zn (0.77 g, 11.8 mmol) dust was added and stirred at 40 °C until completion. The reaction mixture was filtered over Celite® 545 and concentrated to dryness and dried over high vacuum for 2 h. The crude was dissolved in pyridine (10 mL) and acetic anhydride (5 mL) was added slowly over 10 min at 0 °C. The reaction was allowed to stir at room temperature until completion. The solvent was evaporated and dissolved in EtOAc (150 mL) and washed with aq. NaHCO3 (50 mL), 1N HCl (50 mL), and brine (50 mL). The organic layer was dried over anhydrous Na_2SO_4 and concentrated. The crude thus obtained was purified using silica gel flash column chromatography to obtain protected disaccharide **24** (0.67 g, 85%). ^{1}H NMR (600 MHz, $CDCl_3$) δ 7.75 (d, J = 7.5 Hz, 2H), 7.61 (t, J = 7.5 Hz, 2H), 7.39 (t, J = 7.4 Hz, 2H), 7.31 (dd, J = 13.5, 6.7 Hz, 2H), 6.07 (d, J = 7.5 Hz, 1H), 5.92 (d, J = 7.6 Hz, 1H), 5.72 (d, J = 9.6 Hz, 1H), 5.48 (t, J = 9.8 Hz, 1H), 5.29 (d, J = 2.9 Hz, 1H), 5.05 (d, J = 11.2 Hz, 1H), 4.93 (t, J = 9.5 Hz, 1H), 4.89 – 4.76 (m, 2H), 4.54 (t, J = 8.8 Hz, 1H), 4.45 – 4.41 (m, 1H), 4.38 (d, J = 5.9 Hz, 2H), 4.26 – 4.19 (m, 2H), 4.04 (s, 1H), 4.00 (d, J = 12.0 Hz, 1H), 3.95 (d, J = 7.1 Hz, 1H), 3.74 (dd, J = 10.1, 5.0 Hz, 2H), 3.63 (d, J = 6.4 Hz, 1H), 3.52 (dd, J = 10.4, 7.4 Hz, 1H), 3.42 (d, J = 9.4 Hz, 1H), 2.11 (s, 3H), 1.99 (s, 3H), 1.97 (d, J = 3.1 Hz, 9H), 1.91 (s, 5H), 1.87 (s, 3H), 1.47 (s, 9H). ^{13}C NMR (151 MHz, $CDCl_3$) δ 170.84, 170.64, 170.55, 170.41, 170.23, 170.00, 169.47, 169.01, 155.98, 143.70, 141.26, 127.79, 127.16, 127.14, 125.06, 120.02, 99.70, 98.21, 82.98, 77.21, 77.00, 76.79, 71.64, 71.42, 68.82, 68.62, 68.36, 68.17, 67.67, 67.23, 62.00, 55.79, 54.69, 47.53, 47.03, 28.01, 23.22, 20.72, 20.69, 20.63, 20.59. HRMS calcd for **24** $C_{48}H_{61}N_3O_{20}$ $[M+H]^+$ m/z = 1022.3927, found: 1000.3897.

Compound 5 (core 6):

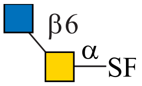

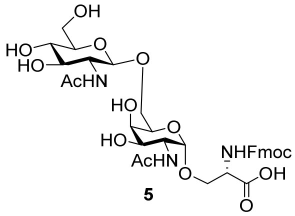

5

To the purified compound **24** (0.5 g, 0.5 mmol), a mixture of TFA/Anisole (9:1) (5 mL) at 0 °C was added and stirred until completion (15 min). The reaction mixture was concentrated below 30 °C and coevaporated with toluene (2×5 mL) and dried under high vacuum for 1 h. The crude was dissolved in anhydrous MeOH (5 mL) and

solid NaOMe was added slowly at 0 °C until the pH reached 8.5. The reaction was allowed to stir at room temperature until completion. The reaction was neutralized using Amberlite® IRC120 H acidic resin and filtered over Celite® 545. The solution was concentrated and purified by C18 reverse phase flash column chromatography using H_2O and acetonitrile to obtain compound **5** (0.33 g, 90% over two steps) as a white solid. ^{1}H NMR (600 MHz, D_2O) δ 8.19 (s, 2H), 7.99 (s, 2H), 7.85 – 7.63 (m, 4H), 4.98 (s, 3H), 4.62 (s, 1H), 4.37 (s, 1H), 4.26 (s, 1H), 4.24 – 3.87 (m, 9H), 3.85 (s, 1H), 3.73 (s, 2H), 2.26 (s, 6H). ^{13}C DEPT 135 NMR (101 MHz, $CDCl_3$) δ 127.79, 127.26, 124.98, 124.82, 119.96, 101.20, 97.79, 75.80, 73.36, 69.91, 69.90, 69.31, 69.28, 68.66, 68.50, 68.40, 67.68, 67.63, 66.47, 60.65, 56.11, 55.60, 49.65, 46.65, 22.28, 22.13. HRMS, calcd $C_{34}H_{43}N_3O_{15}$: 733.2694; found $[M-H]^-$ 732.2716. HPLC, T_R =18.614 min.

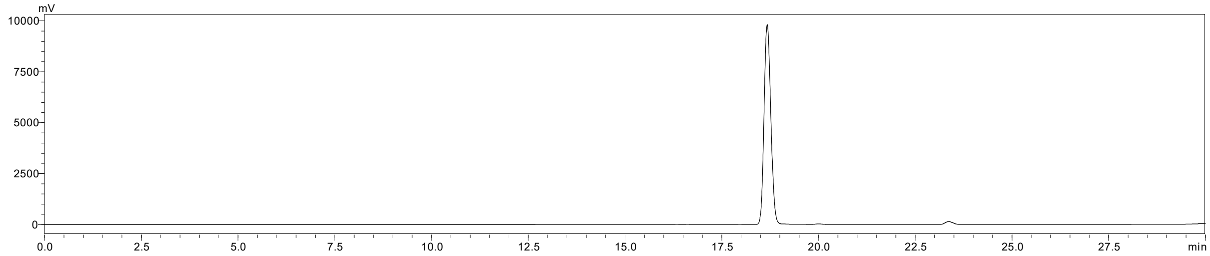

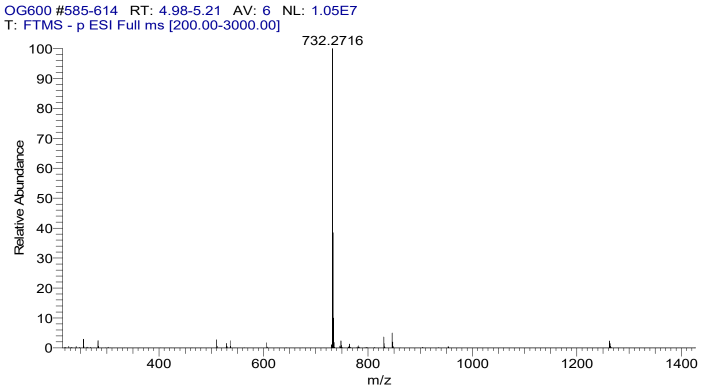

Compound 6 (Tn-antigen, GalNAcα-Ser-Fmoc):

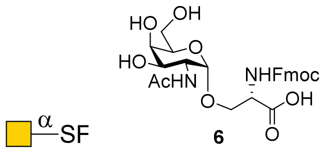

To a solution of compound **7** (2.0 g, 3.0 mmol) in 20 mL of anhydrous methanol, PTSA.H_2O (115 mg, 0.6 mmol) was added and stirred at 30 ºC until completion. The reaction was quenched with DIPEA (100 µL), concentrated and the crude was used for the next step without purification. To the crude, 20 mL of precooled TFA/Anisole (9:1) mixture was added at 0 °C and allowed to reach room temperature over 15 min. After confirming the tert-butyl deprotection on TLC, solvent was evaporated under high vacuum below 30 °C. The crude was co-evaporated twice with toluene (2 × 5 mL) and dried under high vacuum for 1 hr. The crude solid thus obtained was dissolved in 20 mL of anhydrous methanol. Solid NaOMe was added slowly and carefully such that the pH does not exceed 8.5. The reaction was stirred at room temperature and frequently checked for completion on TLC. After completion, the reaction was neutralized using Amberlite® IRC120 H acidic resin and filtered over Celite® 545. The solution was concentrated and purified by silica gel flash column chromatography to obtain compound **6** (1.19 g, 81% over three steps) as a white solid. ^{1}H NMR (400 MHz, MeOD) δ 7.81 (d, J = 7.5 Hz, 2H), 7.71 – 7.66 (m, 2H), 7.40 (t, J = 7.4 Hz, 2H), 7.33 (t, J = 7.4 Hz, 2H), 4.80 (d, J = 3.2 Hz, 1H), 4.38 (d, J = 6.8 Hz, 2H),

4.31 – 4.19 (m, 3H), 3.97 – 3.89 (m, 2H), 3.75 (ddt, J = 20.3, 16.8, 5.5 Hz, 6H), 2.01 (s, 3H). ^{13}C NMR (101 MHz, MeOD) δ 175.57, 172.84, 156.70, 143.91, 141.19, 127.39, 126.79, 124.81, 119.53, 98.27, 71.06, 69.04, 68.90, 68.63, 66.57, 61.40, 56.27, 50.00, 21.51. HRMS, calcd $C_{26}H_{30}N_2O_{10}$: 530.1900; found $[M+Na]^+$ 553.188. HPLC, T_R = 23.262 min.

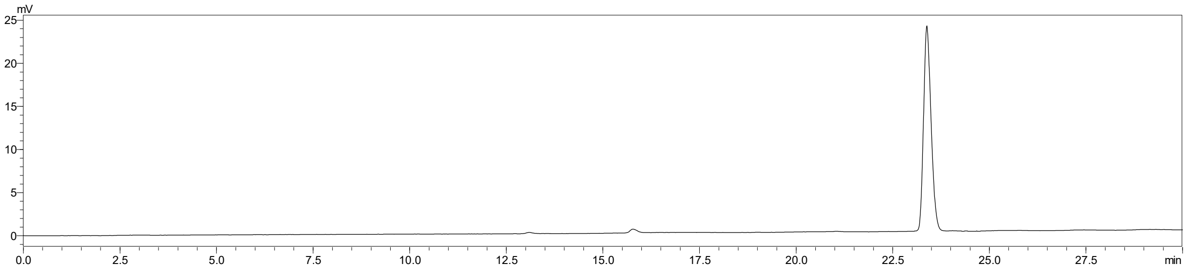

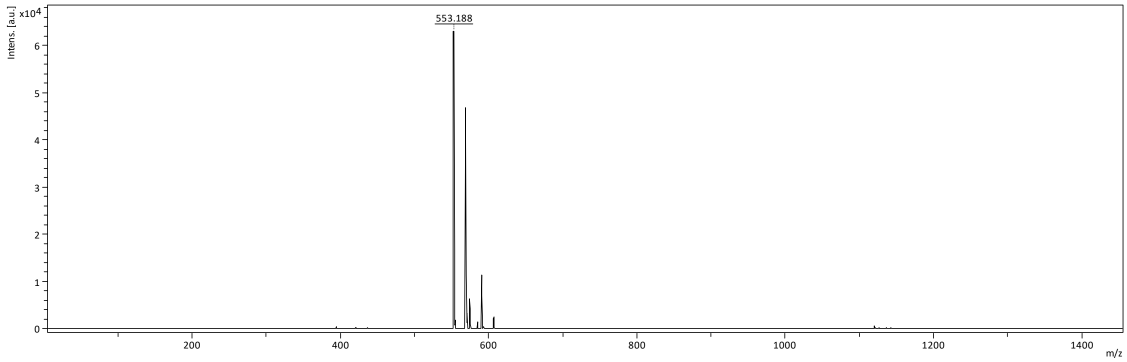

III. Enzymatic Modular Assembly to Diversify O-GalNAc Glycans

Materials

Unless otherwise stated, chemicals were purchased and used without further purification. Sugar nucleotides, such as uridine 5'-diphospho-galactose (UDP-Gal),[5] guanosine 5'-diphospho-L-fucose (GDP-Fuc),[6] uridine 5'-diphosphate-*N*-acetylglucosamine (UDP-GlcNA),[6] uridine 5'-diphosphate-*N*-acetylgalactosamine (UDP-GalNAc)[6] were prepared as described previously reported. Enzymes including *Neisseria meningitides* β1-4 galactosyltransferase (NmLgtB),[7] human α1-3 galactosyltransferase (GTB),[8] bovine α1-3 galactosyltransferase (Bα3GalT),[9] *N. meningitidis* CMP-sialic acid synthetase (NmCSS),[10] *Pasteurella multocida* α2-3 sialyltransferase mutant M144D (PmST1-M144D),[11] *P. multocida* α2-6sialyltransferase with selectively sialylation activity toward only terminal galactose residue (PmST1- P34H/M144L),[12] *Photobacterium damsela* α2-6 sialyltransferase (Pd2,6ST)[13], *Campylobacter jejuni* β1-4 *N*-acetylgalactosaminetransferase (CjCgtA),[14] *Helicobacter mustelae* α1-3 *N*-acetylgalactosaminyltransferase (HmBgtA),[15] *H. pylori* β1-3 *N*-acetylglucosaminyltransferase (HpLgtA),[16] *H. mustelae* α1-2 fucosyltransferase (Hm2FT),[17] *H. pylori* α1-3fucosyltransferase C-terminal 66 amnio acid truncation (Hp3FT),[18] bovine β1-4 galactosyltransferase mutant Y289L/C342T (b4GalTm)[19] and human ST6GalNAc-IV[20] were expressed and purified as previously described or purchased from Sigma.

General HPLC methods

<u>HPLC method for monitoring reactions and purity analysis of final products</u>: An analytical GL Science Inertsil ODS-4 column (100 Å, 5 µm, 4.6 mm × 250 mm) was used and the signal was monitored by a UV detector (260 nm) or fluorescent detector (E_x 260nm, E_m 310 nm).[21] The running solvents are solvent A(H_2O with 0.1% TFA) and solvent B (acetonitrile with 0.1% TFA). The running condition is gradient elution with solvent B% linear increased from 20% to 40% within 25 mins, with a total flow rate of 1 mL/min.

<u>HPLC method for purifying final products</u>: An analytical GL Science Inertsil ODS-4 column (100 Å, 5 µm, 4.6 mm × 250 mm) was used for separating small reactions with 3 mg or less products, and a semipreparative Inertsil ODS-4 column (100 Å, 5 µm, 10 mm × 250 mm) was used for separating products with over 3 mgs. The method for using the analytical column is same as above and monitored by a UV detector (260 nm). The method for using the semipreparative column is similar as that for the analytical column described above, with the only difference of flow rate at 4 mL/min instead.

Enzyme Modules

<u>G1. β1-4 galactosylation with NmLgtB</u>

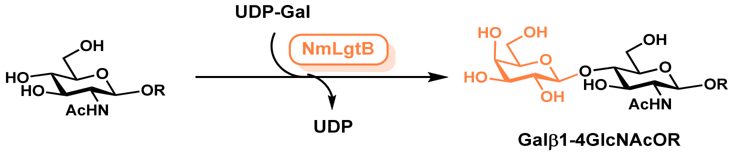

Reaction mixtures contain Tris-HCl (100 mM, pH 7.5), an acceptor glycan (10 mM), UDP-Gal (15 mM), $MgCl_2$ (10 mM), and an appropriate amount of NmLgtB. Reactions were incubated at 37 °C overnight and monitored by HPLC and/or MALDI-TOF MS. After over 95% acceptor was converted, the reaction was quenched, concentrated and subject for HPLC separation. Product-containing fractions were pooled and lyophilized for characterization and next step modular assembly.

G2. α1-3 galactosylation with GTB

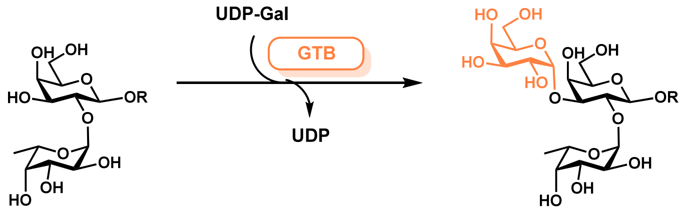

Reaction mixtures contain Tris-HCl (100 mM, pH 7.5), an acceptor glycan (10 mM), UDP-Gal (15 mM), $MgCl_2$ (10 mM), and an appropriate amount of GTB. Reactions were incubated at 37 °C overnight and monitored by HPLC and/or MALDI-TOF MS. After over 95% acceptor was converted, the reaction was quenched, concentrated and subject for HPLC separation. Product-containing fractions were pooled and lyophilized for characterization and next step modular assembly.

G3. α1-3 galactosylation with Bα3GalT

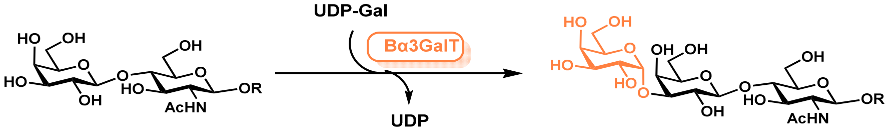

Reaction mixtures contain Tris-HCl (100 mM, pH 7.5), an acceptor glycan (10 mM), UDP-Gal (15 mM), $MgCl_2$ (10 mM), and an appropriate amount of α3GalT. Reactions were incubated at 37 °C overnight and monitored by HPLC and/or MALDI-TOF MS. After over 95% acceptor was converted, the reaction was quenched, concentrated and subject for HPLC separation. Product-containing fractions were pooled and lyophilized for characterization and next step modular assembly.

S1. α2-3 sialylation with PmST1-M144D

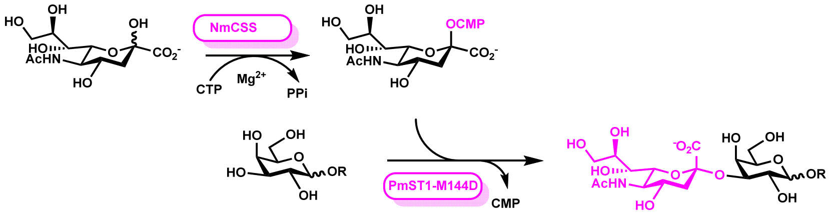

Reaction mixtures contain Tris-HCl (100 mM, pH 8.0), an acceptor glycan (10 mM), CTP (15 mM), Neu5A (15 mM), $MgCl_2$ (10 mM), and appropriate amount of NmCSS and PmST1-M144D. PmST1-M144D-catalyzed reactions were incubated at 37 °C for 3 h and monitored by HPLC and/or MALDI-TOF MS. After over 95% acceptor was converted, the reaction was quenched, concentrated and subject for HPLC separation. Product-containing fractions were pooled and lyophilized for characterization and next step modular assembly.

S2. α2-6 sialylation with PmST1-P34H/M144L

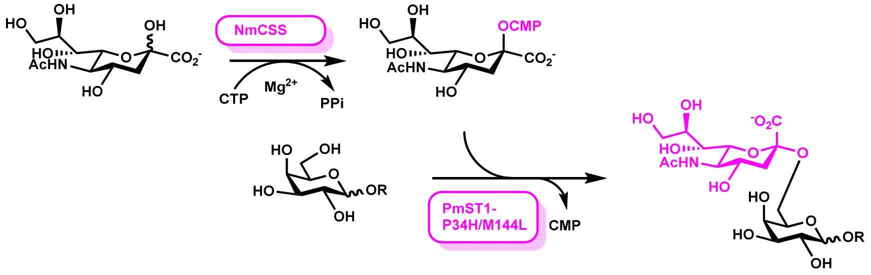

Reaction mixtures contain Tris-HCl (100 mM, pH 8.0), an acceptor glycan (10 mM), CTP (15 mM), Neu5Ac (15 mM), $MgCl_2$ (10 mM), and appropriate amount of NmCSS and PmST1-P34H/M144A. PmST1-P34H/M144L - catalyzed reactions were incubated at 37 °C for 3 h and monitored by HPLC and/or MALDI-TOF MS. After over 95% acceptor was converted, the reaction was quenched, concentrated and subject for HPLC separation. Product-containing fractions were pooled and lyophilized for characterization and next step modular assembly.

S3. α2-6 sialylation with Pd2,6ST

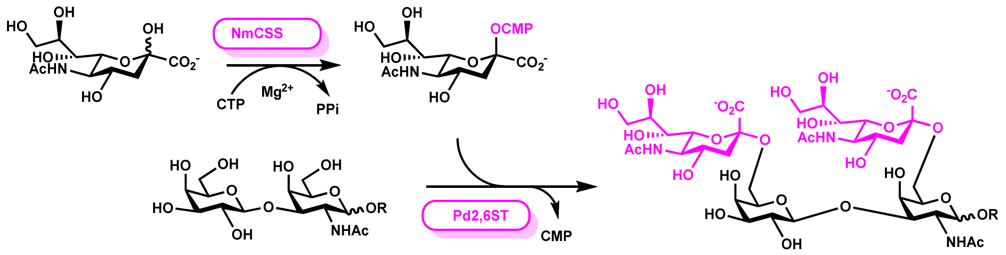

Reaction mixtures contain Tris-HCl (100 mM, pH 8.0), an acceptor glycan (10 mM), CTP (15 mM), Neu5Ac (15 mM), $MgCl_2$ (10 mM), and appropriate amount of NmCSS and Pd2,6ST. Reactions were incubated at 37 °C for 3 h and monitored by HPLC and/or MALDI-TOF MS. After over 95% acceptor was converted, the reaction was quenched, concentrated and subject for HPLC separation. Product-containing fractions were pooled and lyophilized for characterization and next step modular assembly.

S4. α2-6 sialylation with ST6GalNAc-IV

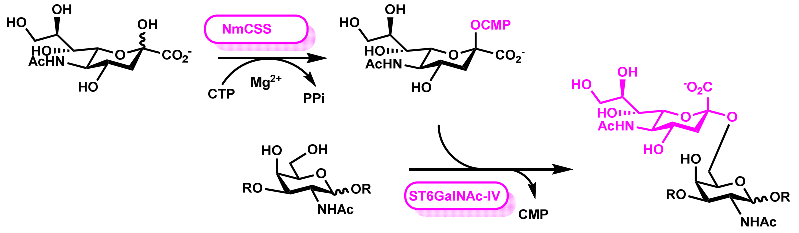

Reaction mixtures contain Tris-HCl (100 mM, pH 8.0), an acceptor glycan (10 mM), CTP (15 mM), Neu5Ac (15 mM), $MgCl_2$ (10 mM), and appropriate amount of NmCSS and human ST6GalNAc-IV. Reactions were incubated at 37 °C overnight and monitored by HPLC and/or MALDI-TOF MS. After over 95% acceptor was converted, the reaction was quenched, concentrated and subject for HPLC separation. Product-containing fractions were pooled and lyophilized for characterization and next step modular assembly.

N1. β1-4-*N*-acetylgalatosaminylation with CjCgtA

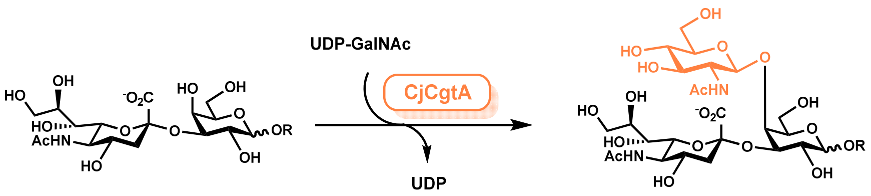

Reaction mixtures contain Tris-HCl (100 mM, pH 7.5), an acceptor glycan (10 mM), UDP-GalNAc (15 mM), $MgCl_2$ (10 mM), and appropriate amount of CjCgtA. Reactions were incubated at 37 °C overnight and were monitored by HPLC and/or MALDI-TOF MS. After over 95% acceptor was converted, reactions were quenched, concentrated before HPLC separation. Product-containing fractions were pooled and lyophilized for characterization and next step modular assembly.

N2. α1-3-*N*-acetylgalatosaminylation with BgtA

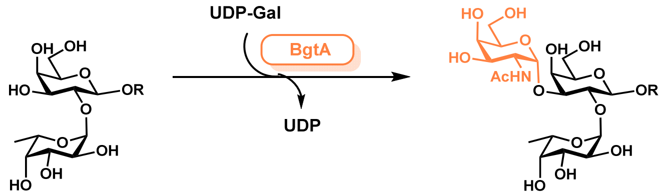

Reaction mixtures contain Tris-HCl (100 mM, pH 7.5), an acceptor glycan (10 mM), UDP-GalNAc (15 mM), $MgCl_2$ (10 mM), and appropriate amount of BgtA. Reactions were incubated at 37 °C overnight and were monitored by HPLC and/or MALDI-TOF MS. After over 95% acceptor was converted, reactions were quenched, concentrated before HPLC separation. Product-containing fractions were pooled and lyophilized for characterization and next step modular assembly.

N3. β1-3-*N*-acetylglucosamine with HpLgtA

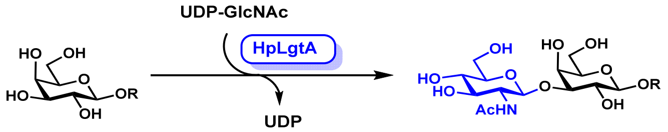

Reaction mixtures contain Tris-HCl (100 mM, pH 7.5), an acceptor glycan (10 mM), UDP-GlcNAc (15 mM), $MgCl_2$ (10 mM), and appropriate amount of HpLgtA. Reactions were incubated at 37 °C overnight and were monitored by HPLC and/or MALDI-TOF MS. After over 95% acceptor was converted, reactions were quenched, concentrated before HPLC separation. Product-containing fractions were pooled and lyophilized for characterization and next step modular assembly.

N4. β1-3-*N*-acetylgalatosaminylation with b4GalTm

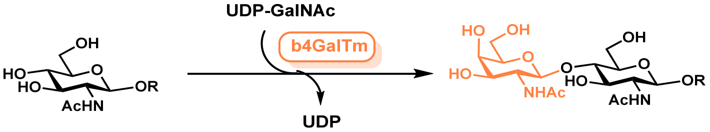

Reaction mixtures contain Tris-HCl (100 mM, pH 7.5), an acceptor glycan (10 mM), UDP-GalNAc (15 mM), $MgCl_2$ (10 mM), and appropriate amount of b4GalTm. Reactions were incubated at 37 °C overnight and were monitored by HPLC and/or MALDI-TOF MS. After over 95% acceptor was converted, reactions were quenched, concentrated before HPLC separation. Product-containing fractions were pooled and lyophilized for characterization and next step modular assembly.

F1. α1-2 fucosylation with Hm2FT

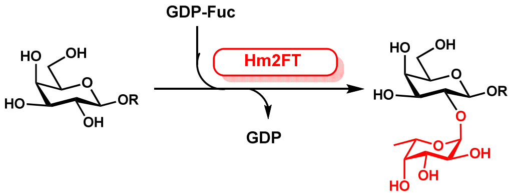

Reaction mixtures contain Tris-HCl (100 mM, pH 7.5), an acceptor glycan (10 mM), GDP-Fuc (15 mM), $MgCl_2$ (10 mM), and appropriate amount of Hm2FT. Reactions were incubated at 37 °C overnight and were monitored by HPLC and/or MALDI-TOF. After over 95% acceptor was converted, reactions were quenched, concentrated before HPLC separation. Product-containing fractions were pooled and lyophilized for characterization and next step modular assembly.

F2. α1-3 fucosylation with Hp3FT

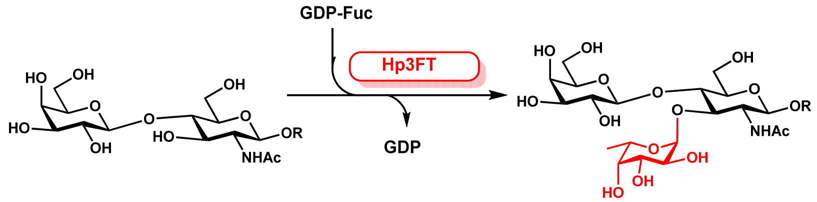

Reaction mixtures contain Tris-HCl (100 mM, pH 7.5), an acceptor glycan (10 mM), GDP-Fuc (15 mM), $MgCl_2$ (10 mM), and appropriate amount of Hp3FT. Reactions were incubated at 37 °C overnight and were monitored by HPLC and/or MALDI-TOF. After over 95% acceptor was converted, reactions were quenched, concentrated before HPLC separation. Product-containing fractions were pooled and lyophilized for characterization and next step modular assembly.

IV. Glycan Microarray Fabrication and Assay

Method for removing Fmoc

O-GalNAc glycans (50 μg) were dissolved in 200 μL H_2O, and 30 μL triethylamine was added to remove the Fmoc group at room temperature for 4 h. The reactions were then lyophilized, and hexane extraction was applied to remove free Fmoc.

Method for microarray fabrication

The O-GalNAc microarray was printed according to the guidelines of MIRAGE as summarized in Supplementary Table 1. O-GalNAc glycans and PPA [PGST(GalNAcα-)APP], PPAP [TSAPDT(GalNAcα-)RPAP] were prepared at a concentration of 100 μM in the printing buffer (300 mM phosphate, pH 8.5), and printed on Nexterion slide H-3D hydrogel coated glass microarray slides (Applied Microarrays Inc), each for 400 pL in replicates of three. Non-contact printing was performed at room temperature with a humidity of 60% by a sciFLEXARRAYER S3 spotter (Scienion) with two PDC 80 Piezo Dispense Capillary, and 8 subarrays were printed on each slide. After overnight dehumidification under room temperature, the slides were washed with MilliQ water and subsequently blocked with 50 mM ethanolamine in 100 mM Tris buffer (pH 9.0) for 2 hours. The blocked slides were then washed with MilliQ water twice, dried, and stored desiccated at -20 °C until use. Print buffer was printed as a negative control. In addition, biotinylated PEG amine (0.01mg/mL), Mouse IgG (0.1 mg/mL) and Human IgG (0.1 mg/mL) were printed in six replicates in print buffer to serve as a positive control. A marker containing anti-human IgG conjugate with Cy3 (0.01 mg/mL) and anti-human IgG conjugate with Alexa 647 (0.01 mg/mL) was also printed in triplicates.

Method for microarray assay

Materials: All biotinylated lectins were purchased from EYLabs (San Mateo, CA) and Vector Lab (Burlingame, CA), including three Fuc-specific lectins (*Aleuria aurantia* lectin, AAL; *Ulex europaeus* agglutinin I, UEA-1; *Lotus Tetragonolobus* lectin, LTL, two Sia-specific lectins (*Maackia amurensis* lectin I, MAL-I; *Sambucus nigra* lectin, SNA), two LacNAc-specific lectins (*Ricinus communis* agglutinin I, RCA-I; *Erythrina cristagalli* lectin, ECL), and two GlcNAc-specific lectins (*Griffonia simplicifolia* lectin II, GSL-II; *Solanum tuberosum* lectin, STL), two T antigen specific lectins (*Arachis hypogaea* lectin, PNA; *Artocarpus integrifolia* lectin, Jacalin), three Tn antigen specific antigen (*Soybean* agglutinin, SBA; *Vicia villosa* lectin, VVL; *Dolichos biflorus* agglutinin, DBA). Monoclonal mouse anti-human CD15s (sialyl-lewis X) antibody was purchased from BD Biosciences (Franklin Lakers, NJ). Sheep anti-human MUC-1 antibody was purchased from R&D Systems (Minneapolis, MN). Mouse anti-human sialyl-Tn antibody (STn219), Cy5-streptavidin, goat anti-mouse IgG-Alexa Fluor 647 conjugate, His6 Alexa Fluor 647 Conjugate were purchased from Thermo Fisher Scientific (Waltham, MA). Anti-Sheep IgG (H+L) CF™ 633 antibody produced in donkey was purchased from Sigma (St. Louis, MO). Influenza A viruses recombinant H3 Hemagglutinin (HA) of virus A/Brisbane/10/2007 (H3N2) with His-Tag (FR-61), and recombinant H1 HA of virus A/New York/18/2009 (H1N1) (NR-19441), were kindly provided by Dr. Xiu-Feng

Wan from University of Missouri (acquired from BEI resources). The concentration of IgG and IgM of serum specimens was measured by Human IgG Total ELISA Kit and Human IgM ELISA Kit (Invitrogen).
Human serum specimens (Supplementary Table 5) from colorectal cancer patients and normal people were provided by Georgia Cancer Center at Augusta University and stored at -80 °C until use. The protocol for serum specimen preparation was approved by the Institutional Review Board of Augusta University and was performed in accordance with the Helsinki Declaration. All participants gave written informed consent.

Procedures: Microarray slides were blocked in blocking buffer (50 mM ethanolamine in 50 mM sodium borate, pH 9.2) for 1 hour and washed with H2O before assay. Slides were fitted with ProPlate 16-well microarray modules to divide into subarrays, and then rehydrated for 10 min with 100 µL TSMTB Buffer (20 mM Tris-HCl, pH 7.4, 150 mM NaCl, 2 mM CaCl2, 2 mM MgCl2, 0.05% (v/v) Tween-20, 1% (w/v) BSA) at room temperature. Next, the buffer was aspirated and 100 □L of GBPs or serum samples at appropriate concentrations in TSMTB were added into each subarray, sealed and incubated at room temperature for 1 hour with gentle shaking. Slides were then washed with TSMT Buffer (20 mM Tris-HCl, pH 7.4, 150 mM NaCl, 2 mM CaCl2, 2 mM MgCl2, 0.05% (v/v) Tween-20) for four times. Next, slides were added with 100 µL fluorescence-labeled secondary antibody, sealed, and incubated at room temperature for 1 hour with gentle shaking. Finally, slides were washed 4 times with TSMT, TSM (20 mM Tris-HCl, pH 7.4, 150 mM NaCl, 2 mM CaCl2, 2 mM MgCl2), and MilliQ water, respectively, and dried by brief centrifugation. Slides were scanned with a Genepix 4100A microarray scanner (Molecular Devices Corp) using 500 or 600 PMT gains and 80% power, and image analyses were carried out using Genepix Pro 6.1.

Biotin-labelled lectins were detected by Cy5-streptavidin (1 µg/mL). Anti-STn antibody (1:10), anti-MUC-1 antibody (1:50), anti-CD15s antibody (10 µg/mL) were detected by corresponding second antibody with fluorescent label (5 µg/mL). Influenza A Hemagglutinin were detected with 6x-His Tag Monoclonal Antibody (4E3D10H2/E3), Alexa Fluor 647 (5 µg/mL). Human serum specimens were analyzed in a 1/50 dilution and detected using Dylight 650 anti-human IgG Fc (Invitrogen) and Dylight 550 anti-human IgM antibodies (Invitrogen) with a concentration of 5 µg/mL.

V. HPLC, Mass Spectrometry, and NMR Data of Enzymatically Assembled Glycans

Neu5Acα2-6GalNAcα-Ser-Fmoc (25)

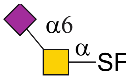

Compound **STn** was prepared according to general procedure of α2-6 sialylation with Pd2,6ST. After lyophilization, **STn** was obtained as white solid (202 mg, 93%). Compound was characterized by HPLC, T_R = 18.188 min. ^{1}H NMR (600 MHz, D_2O) δ 7.81 - 7.63 (m, 2H), 7.61 - 7.52 (m, 2H), 7.41 - 7.24 (m, 4H), 4.63 - 4.43 (m, 2H), 4.47 (s, 1H), 4.33 (s, 1H), 4.19 - 4.00 (m, 2H), 3.94 - 3.50 (m, 13H), 2.68 (m, 1H), 2.07 – 2.02 (m, 3H), 2.00 – 1.90(m, 3H), 1.73 (t, J = 12.1 Hz, 1H). HRMS, $C_{37}H_{47}N_3O_{18}$, Calcd for: 821.2855; found $[M+Na]^+$ 844.265.

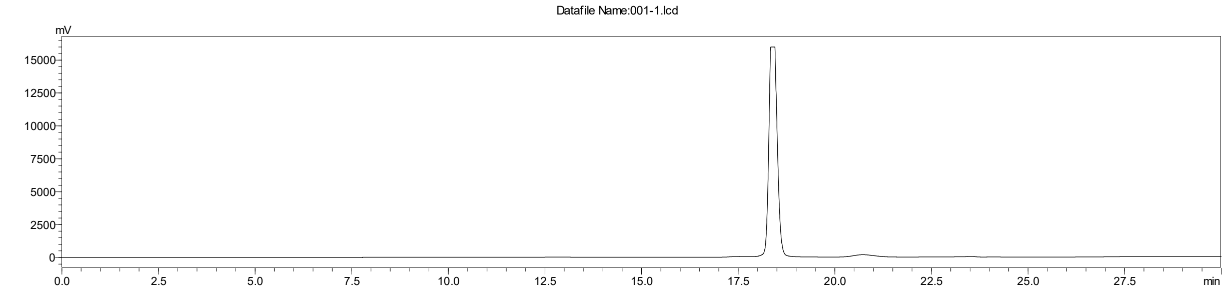

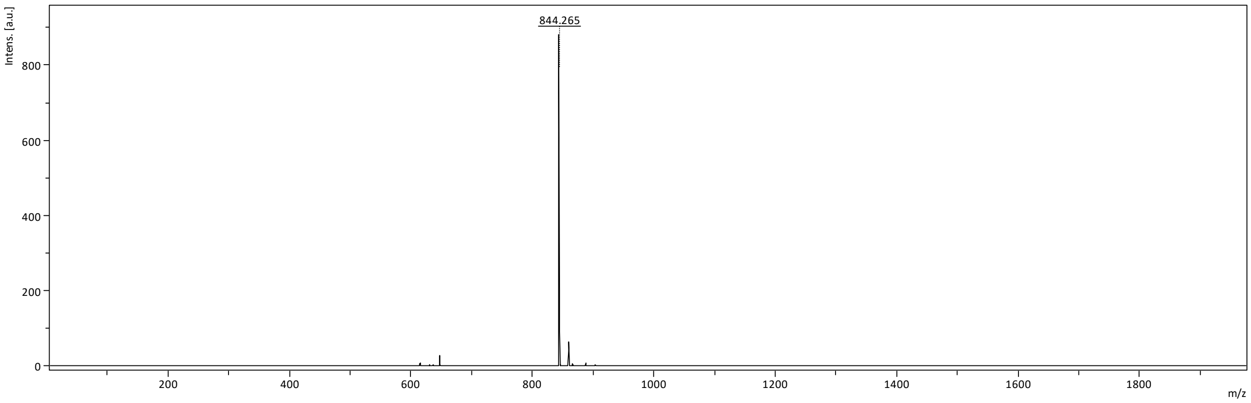

Neu5Acα2-6Galβ1-3GalNAcα-Ser-Fmoc (26)

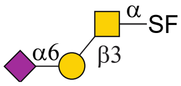

Compound **26** was prepared according to general procedure of α2-6 sialylation with PmST1-P34H/M144L. After lyophilization, **26** was obtained as white solid. Compound was characterized by HPLC, T_R =16.587 min. ^{1}H NMR (600 MHz, D_2O) δ 7.86 - 7.76 (m, 2H), 7.71 - 7.54 (m, 2H), 7.50 - 7.32 (m, 4H), 4.66 - 4.49 (m, 2H), 4.41 - 4.29 (m, 1H), 4.28 - 4.12 (m, 3H), 4.11 - 4.05 (s, 1H), 3.97 - 3.43 (m, 19H), 2.71 (dd, *J* = 12.5, 4.7 Hz, 1H), 2.08 - 2.02 (m, 3H), 2.00 - 1.90 (m, 3H), 1.71 (t, *J* = 12.2 Hz, 1H). HRMS, $C_{43}H_{57}N_3O_{23}$ Calcd for: 983.3383; found $[M-H]^-$ 982.3436.

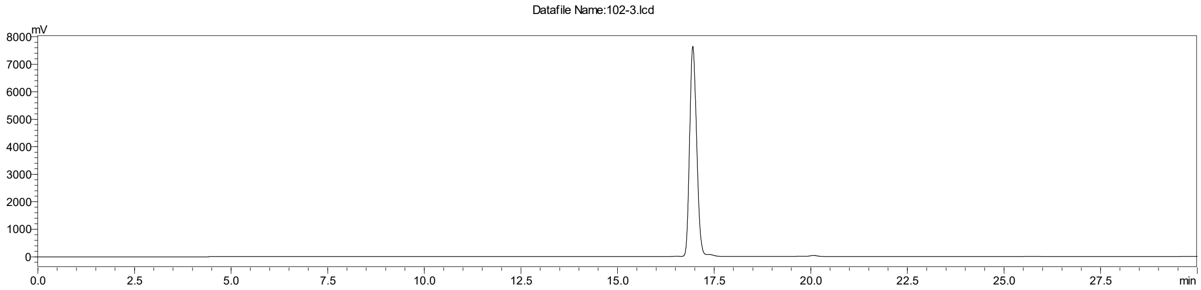

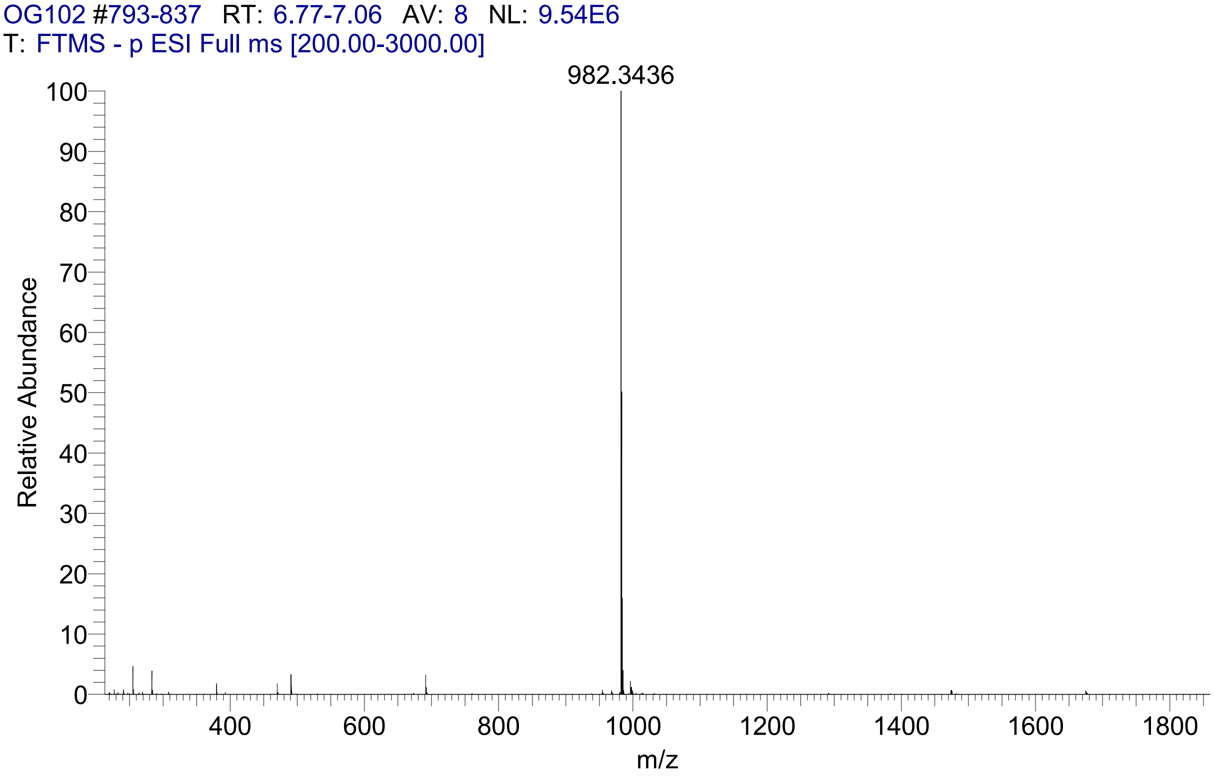

Neu5Acα2-3Galβ1-3GalNAcα-Ser-Fmoc (27)

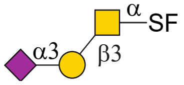

Compound **27** was prepared according to general procedure of α2-3 sialylation with PmST1-M144D. After lyophilization, **27** was obtained as white solid. Compound was characterized by HPLC, T_R = 16.789min. ^{1}H NMR (600 MHz, D_2O) δ 7.68 - 7.54 (m, 2H), 7.53 - 7.35 (m, 2H), 7.34 - 7.12 (m, 4H), 4.76 - 4.71 (m, 2H), 4.49 - 4.34 (m, 3H), 4.38 (s, 1H), 4.20 - 4.12 (m, 1H), 4.11 - 4.05 (s, 1H), 4.00 (dd, *J* = 10.5, 3.6 Hz, 1H), 3.92 - 3.42 (m, 17H), 2.69 (dd, *J* = 12.4, 4.4 Hz, 1H), 2.00 - 1.94 (m, 3H), 1.93 - 1.85 (m, 3H), 1.80 (t, *J* = 11.22 Hz, 1H). HRMS, $C_{43}H_{57}N_3O_{23}$, Calcd for: 983.3383; found $[M-H]^-$ 982.3439.

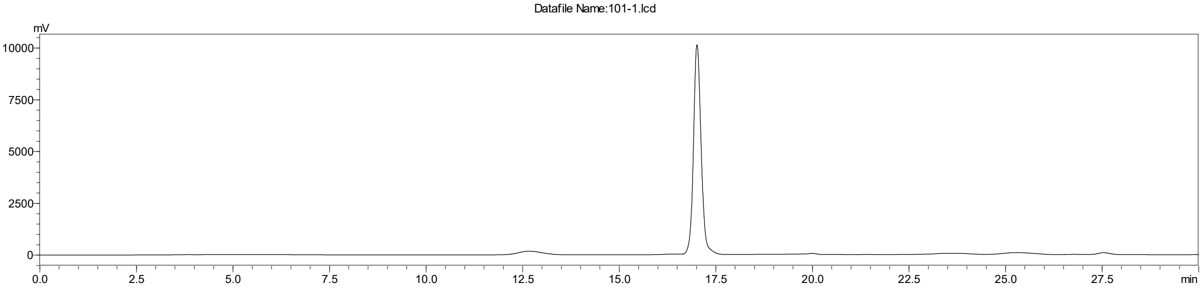

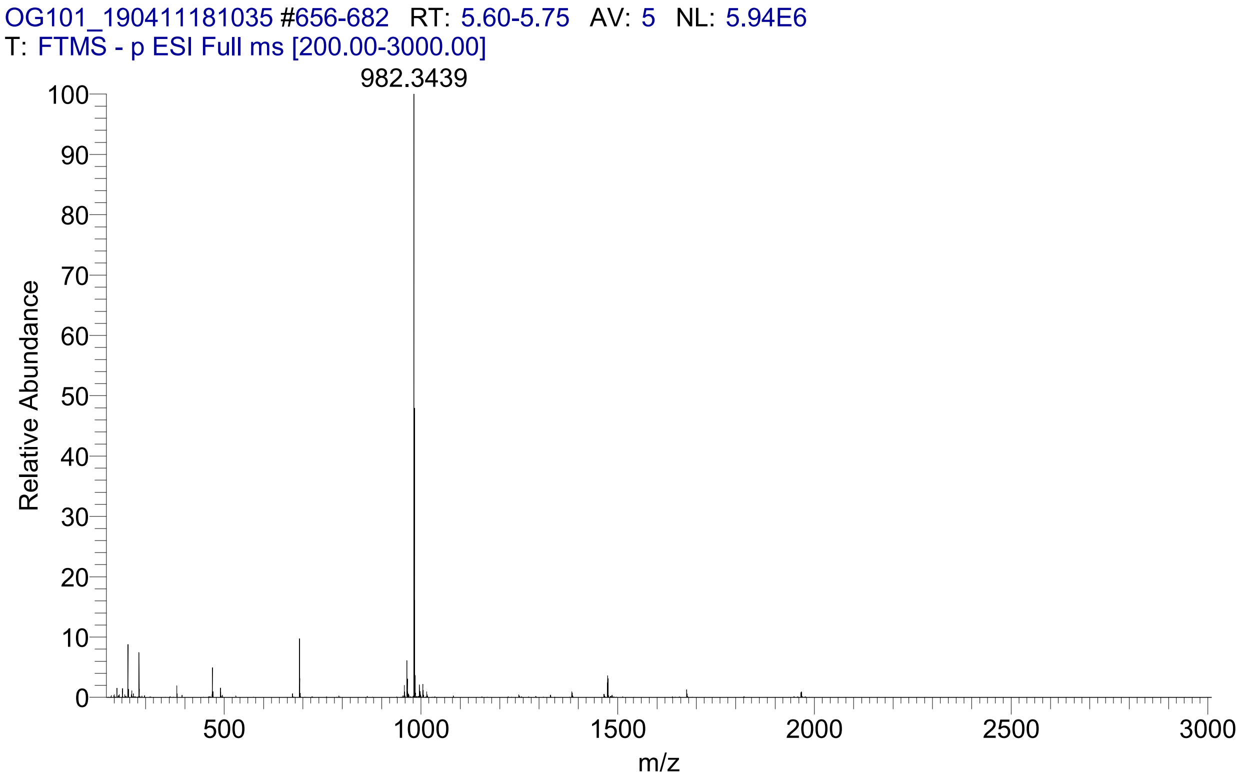

Neu5Acα2-6Galβ1-3(Neu5Acα2-6)GalNAcα-Ser-Fmoc (28)

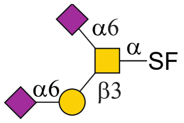

Compound **28** was prepared according to general procedure of α2-6 sialylation with Pd2,6ST. After lyophilization, **28** was obtained as white solid. Compound was characterized by HPLC, T_R =13.052 min. ^{1}H NMR (600 MHz, D_2O) δ 7.87 - 7.79 (m, 2H), 7.70 - 7.54 (m, 2H), 7.49 - 7.30 (m, 4H), 4.66 - 4.49 (m, 2H), 4.36 (s, 1H), 4.31 - 4.23 (m, 2H), 4.21 - 4.08 (m, 2H), 4.04 - 3.97 (m, 1H), 3.93- 3.37 (m, 25H), 2.70 - 2.52 (m, 2H), 2.01 - 1.85 (m, 9H), 1.72 - 1.57 (m, 2H). HRMS, $C_{54}H_{74}N_4O_{31}$, Calcd for: 1274.4337; found $[M-H]^-$ 1273.433.

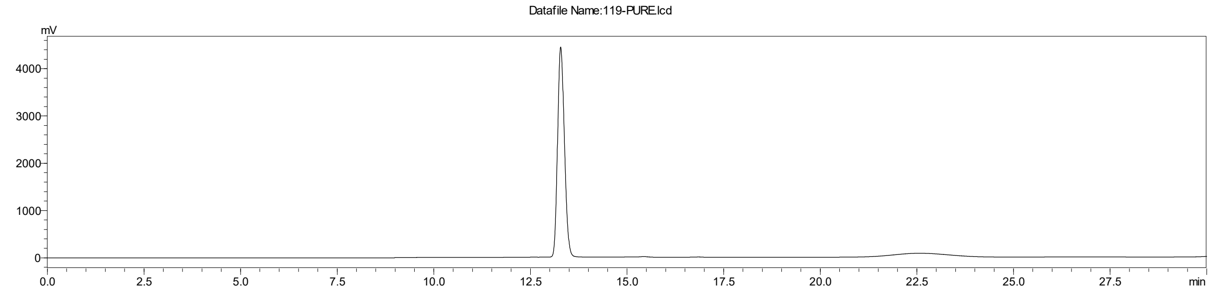

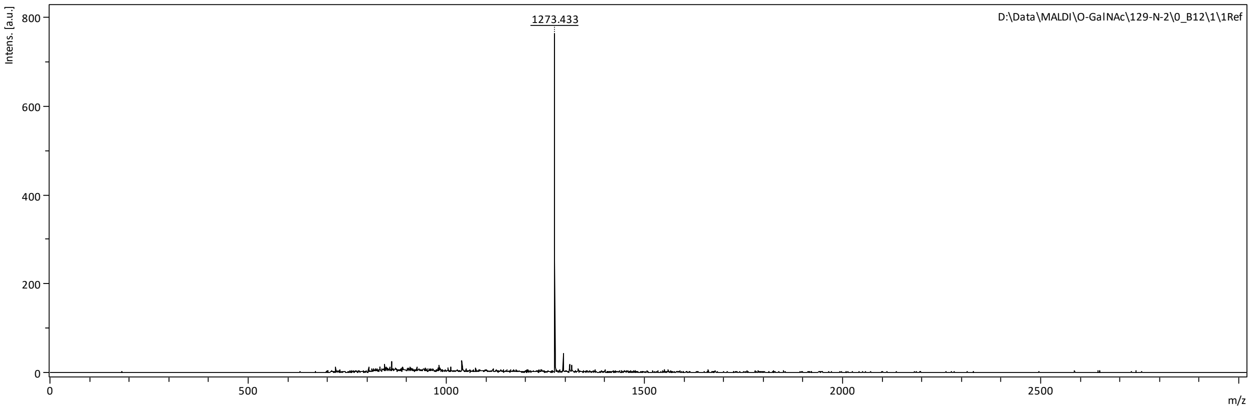

Neu5Acα2-3Galβ1-3(Neu5Acα2-6)GalNAcα-Ser-Fmoc (29)

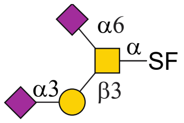

Compound **29** was prepared according to general procedure of α2-6 sialylation with ST6GalNAc-IV. After lyophilization, **29** was obtained as white solid. Compound was characterized by HPLC, T_R =12.381 min. ^{1}H NMR (600 MHz, D_2O) δ 7.89 - 7.77 (m, 2H), 7.70 - 7.54 (m, 2H), 7.49 - 7.30 (m, 4H), 4.69 - 4.49 (m, 2H), 4.42 - 4.20 (m, 3H), 4.19 - 4.07 (m, 2H), 4.04 - 4.39 (m, 1H), 3.93- 3.73 (m, 11H), 3.71 - 3.37 (m, 14H), 2.71 (dd, *J* = 12.6, 3.9 Hz, 1H), 2.59 (dd, *J* = 12.3, 4.5 Hz, 1H), 1.98 (s, 3H), 2.01 - 1.96 (m, 6H), 1.93 - 1.85 (m, 3H), 1.77 (t, *J* = 12.3 Hz, 1H), 1.59 (t, *J* = 12.2 Hz, 1H). HRMS, $C_{54}H_{74}N_4O_{31}$, Calcd for: 1274.4337; found $[M-H]^-$ 1273.412.

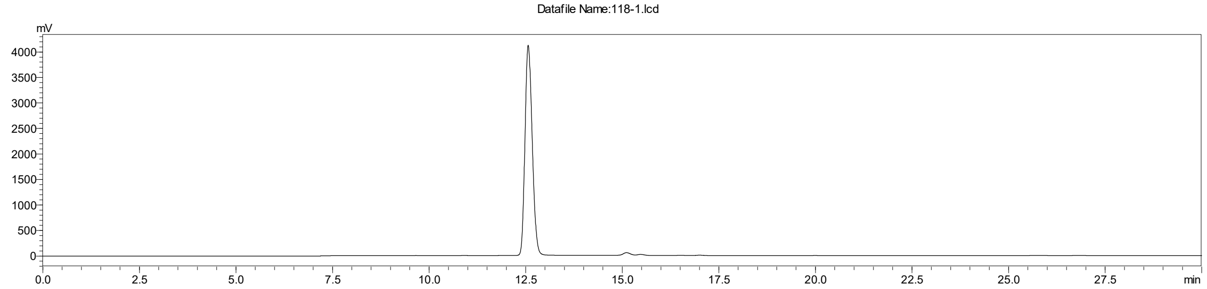

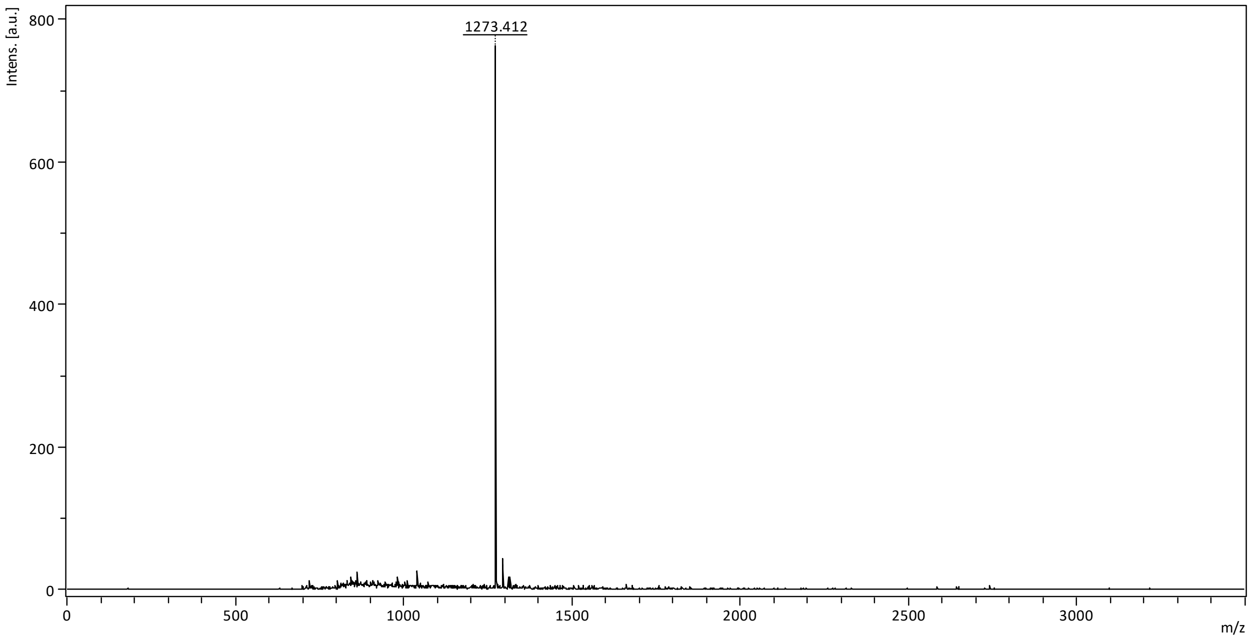

Neu5Acα2-3(GalNAcβ1-4)Galβ1-3GalNAcα-Ser-Fmoc (30)

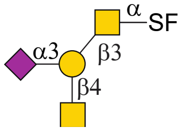

Compound **30** was prepared according to general procedure of β1-4-*N*-acetylgalatosaminylation with CgtA. After lyophilization, **30** was obtained as white solid. Compound was characterized by HPLC, T_R =15.664 min. ^{1}H NMR (600 MHz, D_2O) δ 7.98 - 7.86 (m, 2H), 7.77 - 7.63 (m, 2H), 7.57 - 7.39 (m, 4H), 4.67 - 4.61 (m, 1H), 4.53 - 4.41 (m, 1H), 4.40 - 4.31 (m, 2H), 4.28 - 4.18 (m, 1H), 4.16 - 4.03 (m, 3H), 3.97 - 3.59 (m, 24H), 3.54 (d, *J* = 10.2 Hz, 1H), 3.36 – 3.31 (m, 1H), 2.73 – 2.64 (m, 1H), 2.05 (s, 3H), 2.01 (s, 3H), 1.94 (s, 3H). HRMS, $C_{51}H_{70}N_4O_{28}$, Calcd for: 1186.4177; found $[M-H]^-$ 1185.983.

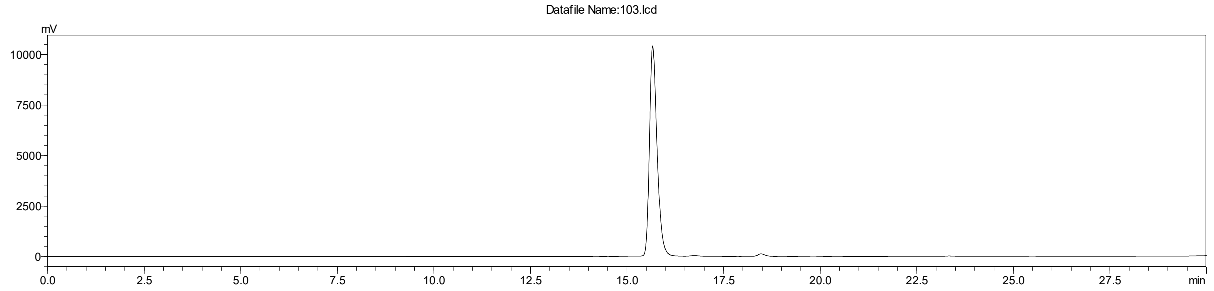

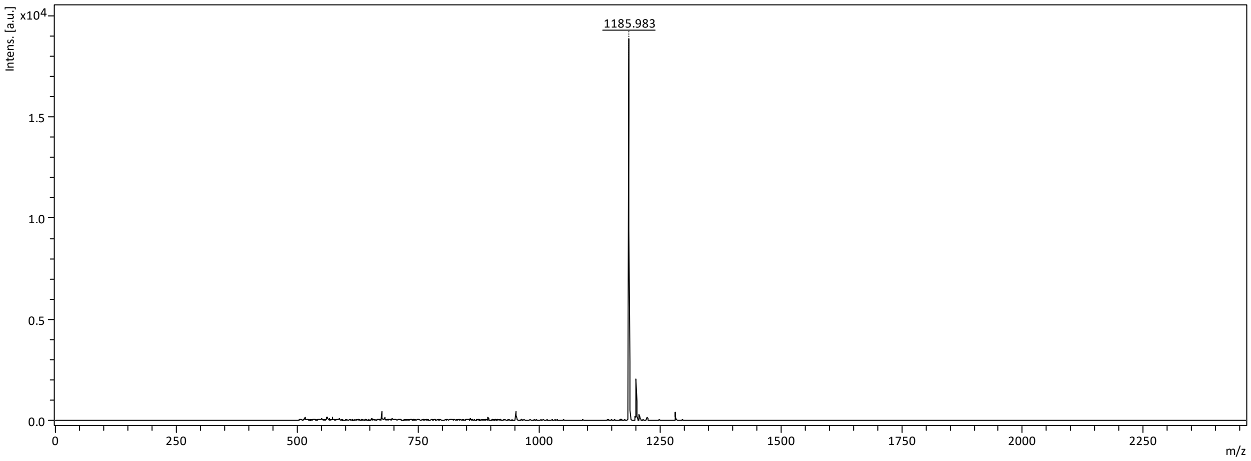

Neu5Acα2-3(GalNAcβ1-4)Galβ1-3(Neu5Acα2-6)GalNAcα-Ser-Fmoc (31)

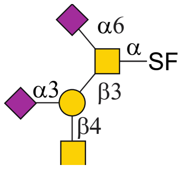

Compound **31** was prepared according to general procedure of α2-6 sialylation with Pd2,6ST. After lyophilization, **31** was obtained as white solid. Compound was characterized by HPLC, T_R =11.657 min. ^{1}H NMR (600 MHz, D_2O) δ 7.98 - 7.87 (m, 2H), 7.78 - 7.62 (m, 2H), 7.57 - 7.38 (m, 4H), 4.65 (s, 1H), 4.50 - 4.16 (m, 4H), 4.15 - 4.03 (m, 3), 3.98 - 3.49 (m, 31H), 3.32 (t, J = 8.7 Hz, 1H), 2.74 -2.61 (m, 2H), 2.05 (s, 3H), 2.04 (s, 3H), 2.01 (s, 3H), 1.94 (s, 3H), 1.76 - 1.62 (m, 2H). HRMS, $C_{62}H_{87}N_5O_{36}$, Calcd for: 1477.5131; found $[M-H]^-$ 1476.531.

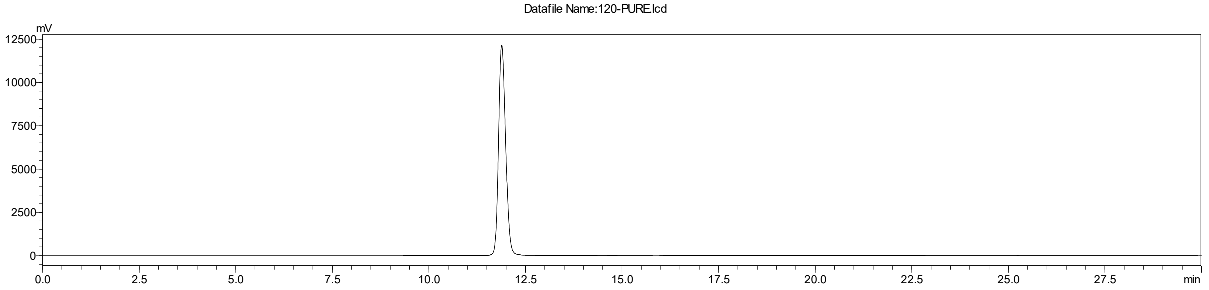

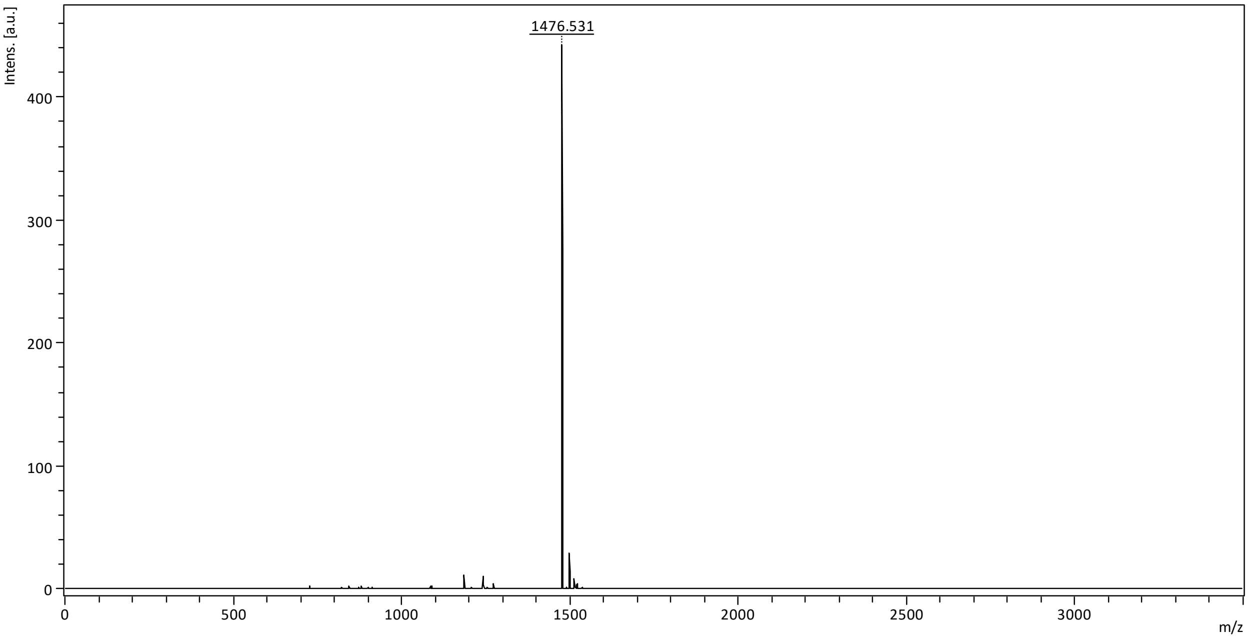

Fucα1-2Galβ1-3GalNAcα-Ser-Fmoc (32)

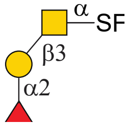

Compound **32** was prepared according to general procedure of α1-2 fucosylation with Hm2FT. After lyophilization, **32** was obtained as white solid. Compound was characterized by HPLC, T_R = 17.912 min. ^{1}H NMR (600 MHz, D_2O) δ 7.95 - 7.76 (m, 2H), 7.76 - 7.57 (m, 2H), 7.55 - 7.34 (m, 4H), 5.20 (d, *J* = 3.9 Hz, 1H), 4.56 - 4.45 (m, 1H), 4.35 - 4.22 (m, 2H), 4.18 - 4.03 (m, 3H), 3.99 - 3.86 (m, 2H), 3.85 - 3.51 (m, 15H), 2.02 - 1.90 (m, 3H), 1.12 (d, *J* = 6.6 Hz, 3H). HRMS, $C_{38}H_{50}N_2O_{19}$, Calcd for: 838.3008; found $[M-H]^-$ 837.791.

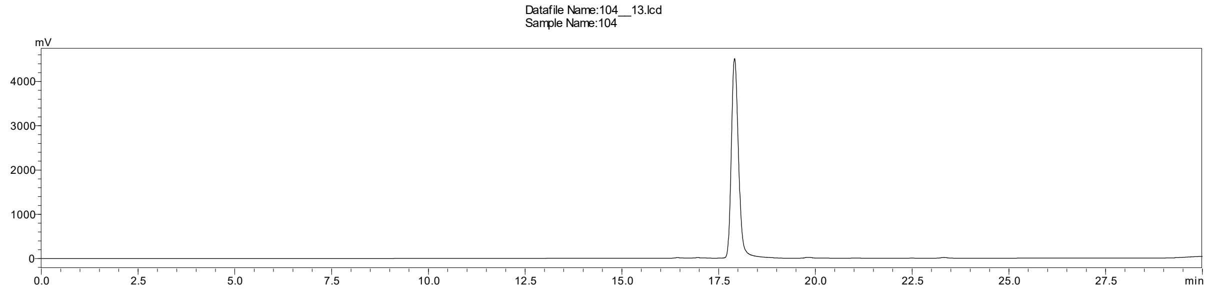

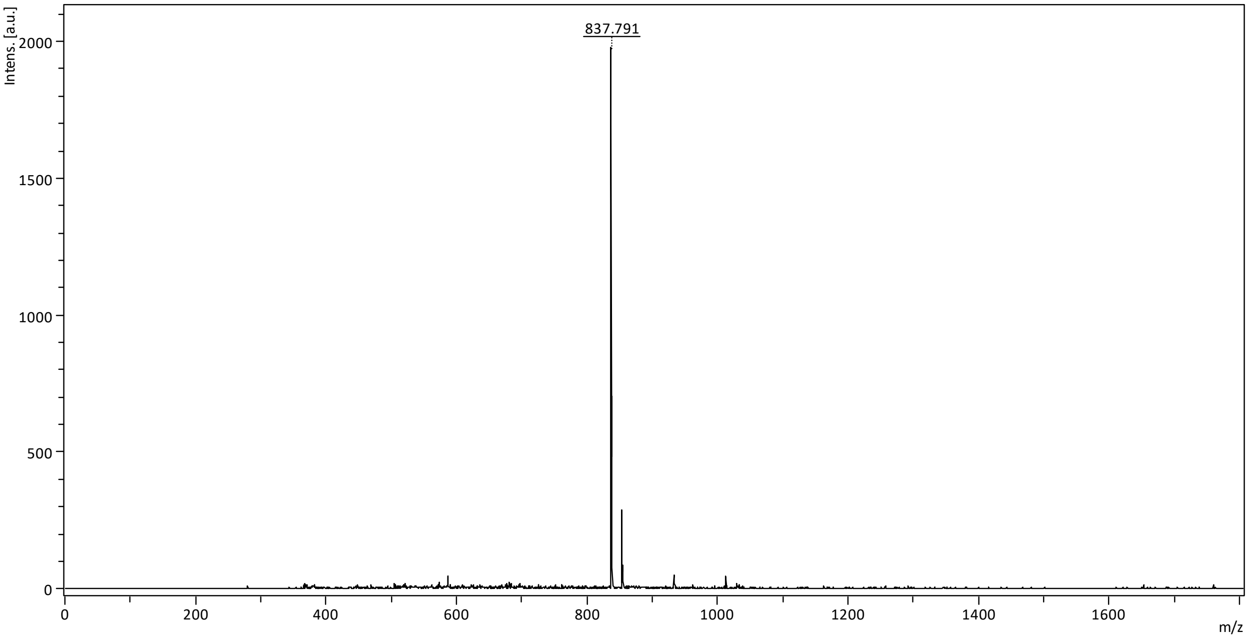

GalNAcα1-3(Fucα1-2)Galβ1-3GalNAcα-Ser-Fmoc (33)

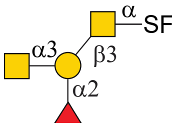

Compound **33** was prepared according to general procedure of α1-3-*N*-acetylgalatosaminylation with BgtA. After lyophilization, **33** was obtained as white solid. Compound was characterized by HPLC, T_R = 16.614 min. ^{1}H NMR (600 MHz, D_2O) δ 7.89 - 7.81 (m, 2H), 7.72 - 7.57 (m, 2H), 7.49 - 7.34 (m, 4H), 5.20 (d, *J* = 4.2 Hz, 1H), 5.14 (d, *J* = 3.7 Hz, 1H), 4.70 - 4.63 (m, 1H), 4.62 - 4.58 (m, 1H), 4.57 - 4.50 (m, 1H), 4.32 - 4.25 (m, 2H), 4.24 - 4.15 (m, 4H), 4.12 - 4.09 (m, 1H), 4.02 - 3.84 (m, 5H), 3.83 - 3.47 (m, 14H), 1.99 (s, 3H), 1.96 - 1.88 (m, 3H), 1.09 (d, *J* = 6.6 Hz, 3H). HRMS, $C_{46}H_{63}N_3O_{24}$, Calcd for: 1041.3801; found $[M-H]^-$ 1040.3857.

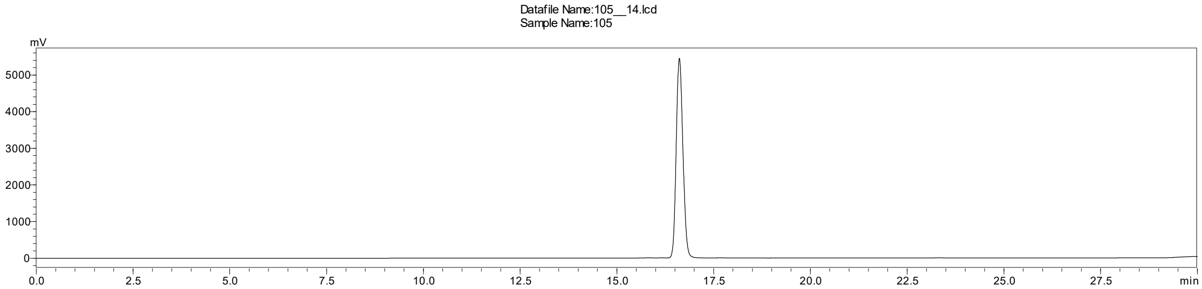

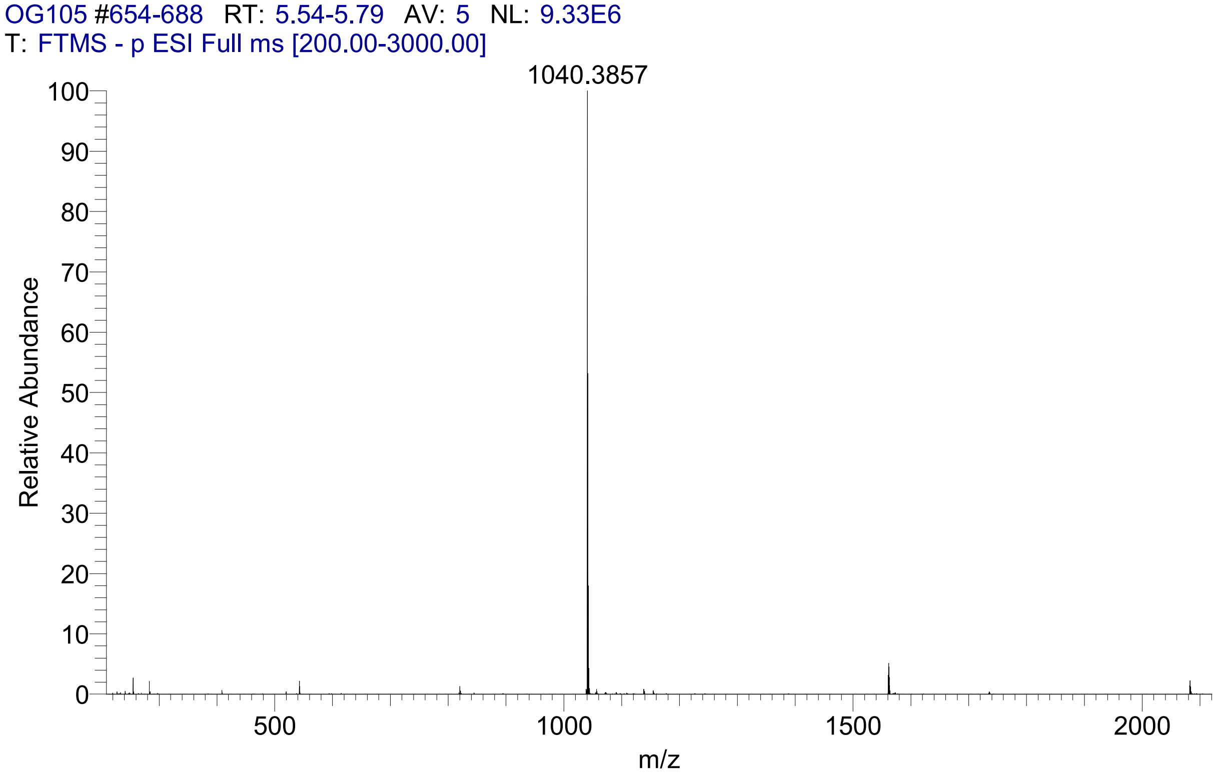

Galα1-3(Fucα1-2)Galβ1-3GalNAcα-Ser-Fmoc (34)

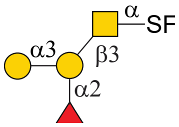

Compound **34** was prepared according to general procedure of α1-3 galactosylation with GTB. After lyophilization, **34** was obtained as white solid. Compound was characterized by HPLC, T_R = 16.612 min. ^{1}H NMR (600 MHz, D_2O) δ 7.89 - 7.81 (m, 2H), 7.72 - 7.57 (m, 2H), 7.49 - 7.34 (m, 4H), 5.20 (d, *J* = 3.5 Hz, 1H), 5.18 (d, *J* = 4.2 Hz, 1H), 4.70 - 4.51 (m, 4H), 4.31 - 4.15 (m, 5H), 4.12 - 4.09 (m, 1H), 4.01 - 3.79 (m, 8H), 3.78 - 3.47 (m, 11H), 1.96 - 1.88 (m, 3H), 1.08 (d, *J* = 6.5 Hz, 3H). HRMS, $C_{44}H_{60}N_2O_{24}$, Calcd for: 1000.3536; found $[M-H]^-$ 999.3583.

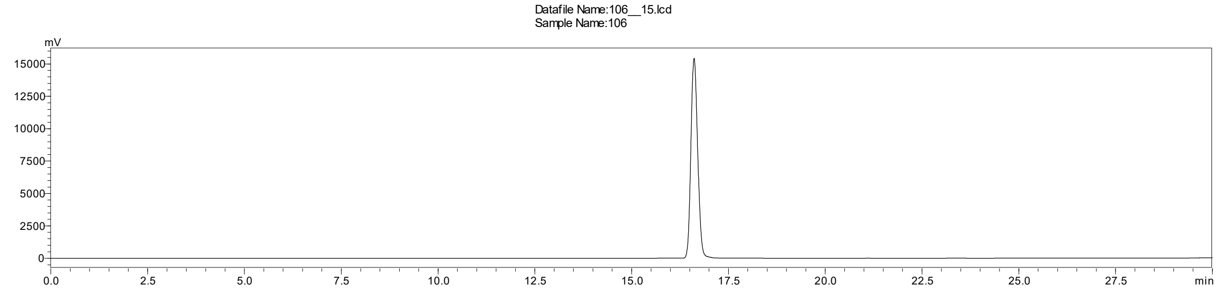

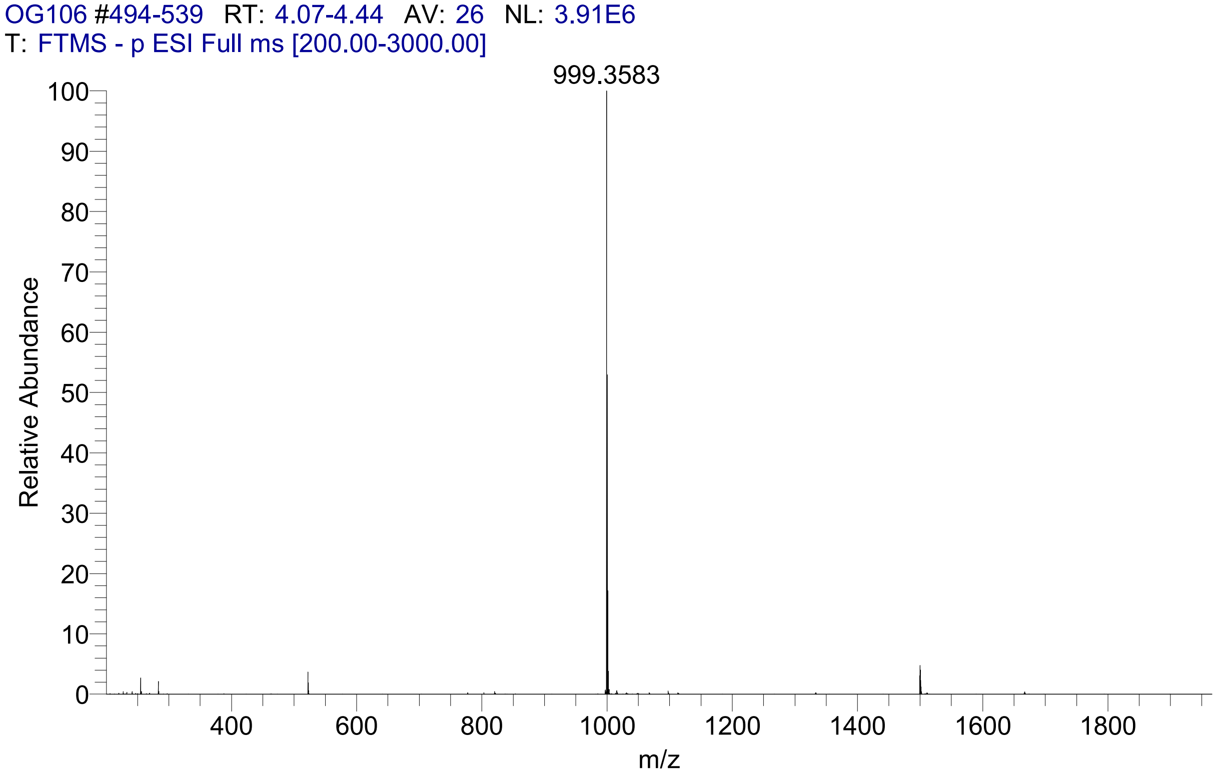

GlcNAcβ1-3Galβ1-3GalNAcα-Ser-Fmoc (35)

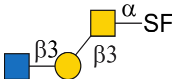

Compound **35** was prepared according to general procedure of β1-3-*N*-acetylgalatosaminylation with HpLgtA. After lyophilization, **35** was obtained as white solid. Compound was characterized by HPLC, T_R = 18.151 min. ^1H NMR (600 MHz, D_2O) δ 7.98 - 7.90 (m, 2H), 7.79 - 7.67 (m, 2H), 7.56 - 7.41 (m, 4H), 4.42 - 4.30 (m, 3H), 4.29 - 4.21 (m, 2H), 4.18 - 4.10 (m, 3H), 3.96 - 3.41 (m, 17H), 1.99 (s, 3H), 2.04 (s, 3H), 1.95 (s, 3H). HRMS, $C_{40}H_{53}N_3O_{20}$, Calcd for: 895.3222; found $[M+H]^+$ 896.3262.

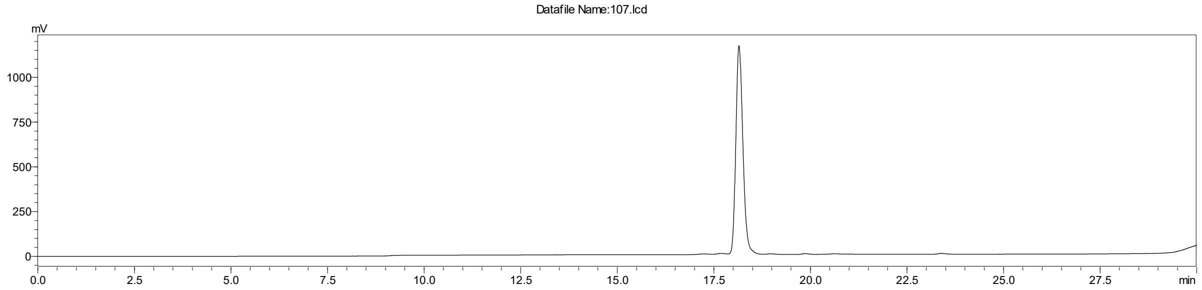

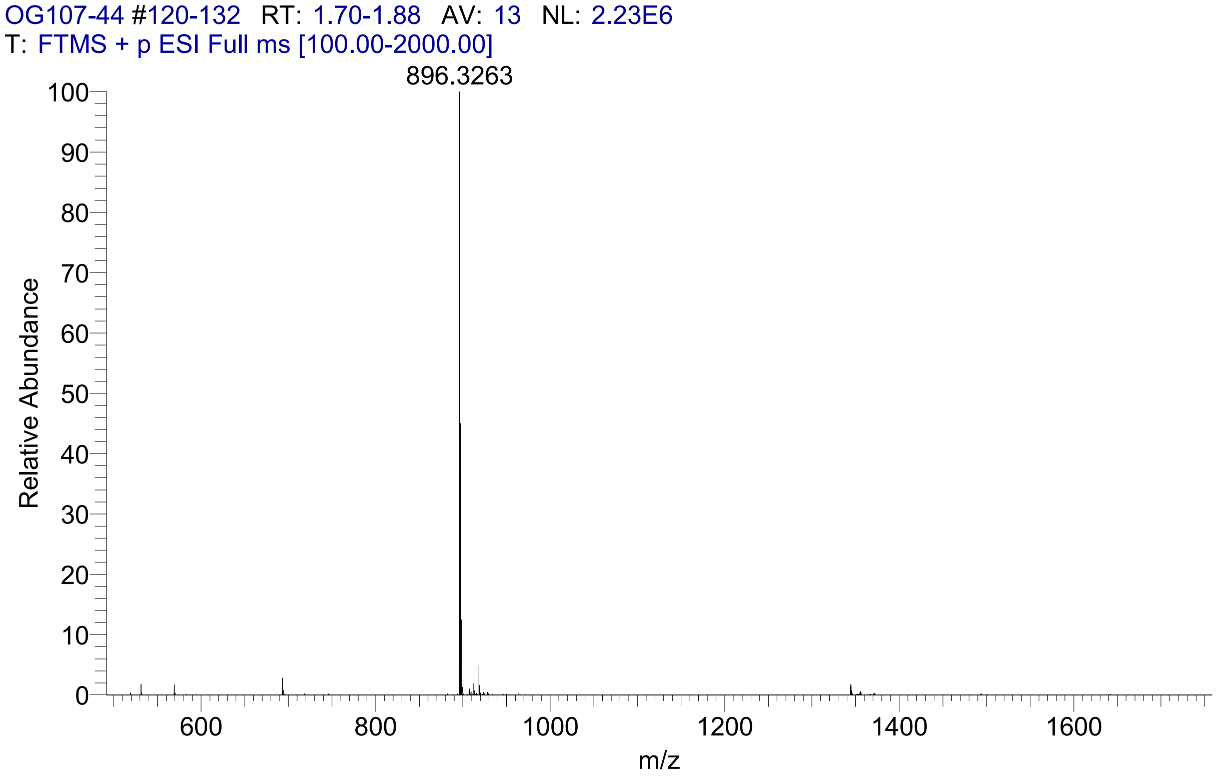

Galβ1-4GlcNAcβ1-3Galβ1-3GalNAcα-Ser-Fmoc (36)

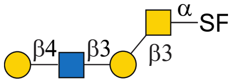

Compound **36** was prepared according to general procedure of β1-4 galactosylation with NmLgtB. After lyophilization, **36** was obtained as white solid. Compound was characterized by HPLC, T_R =16.779 min. ^{1}H NMR (600 MHz, D_2O) δ 7.96 - 7.88 (m, 2H), 7.78 - 7.65 (m, 2H), 7.55 - 7.40 (m, 4H), 4.49 (d, J = 7.8 Hz, 1H), 4.33 (s, 1H), 4.31 (s, 1H), 4.29 - 4.22 (m, 1H), 4.21 - 4.09 (m, 4H), 4.02 - 3.48 (m, 25H), 2.01 (s, 3H), 1.95 (s, 3H). HRMS, $C_{46}H_{63}N_3O_{25}$, Calcd for: 1057.3751; found $[M-H]^-$ 1056.3818.

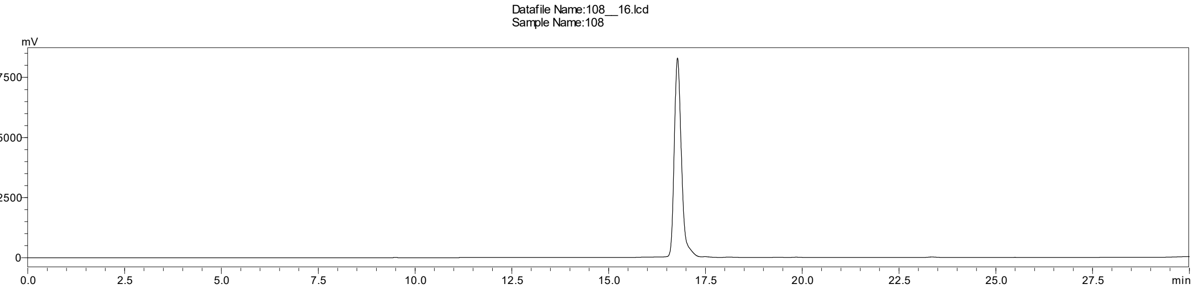

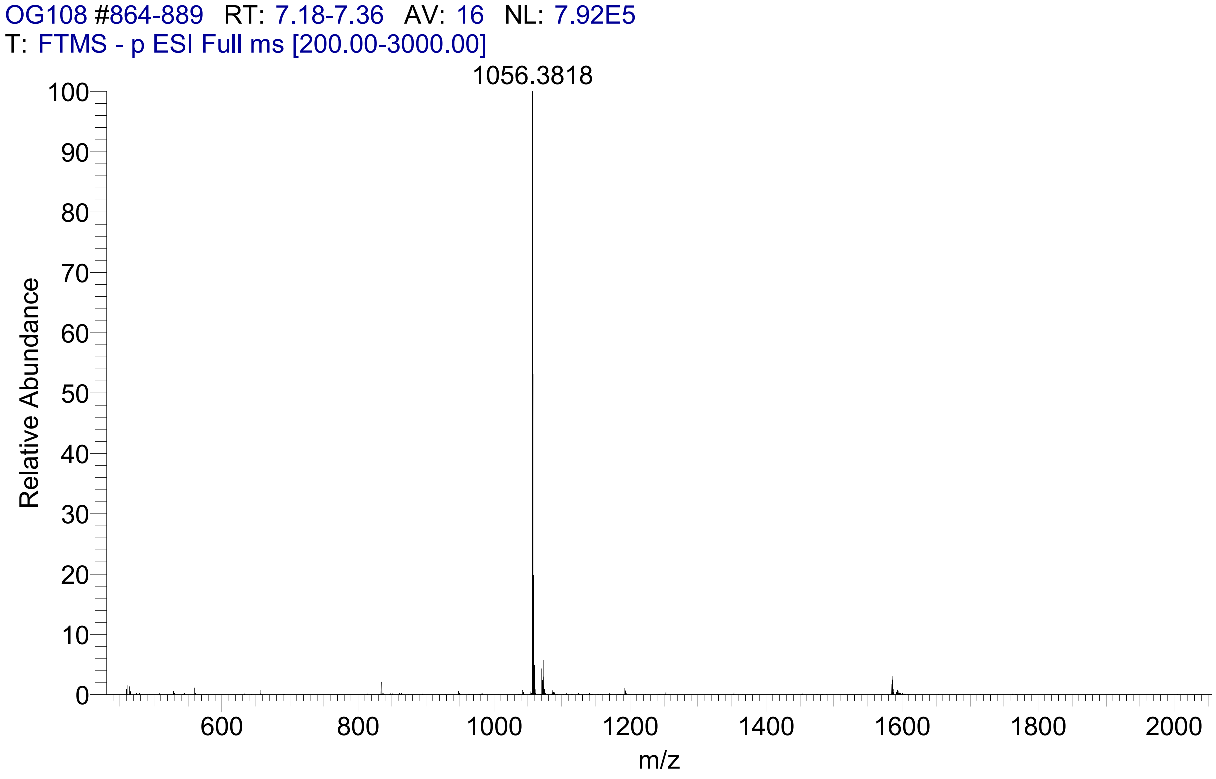

Galβ1-4(Fucα1-3)GlcNAcβ1-3Galβ1-3GalNAcα-Ser-Fmoc (37)

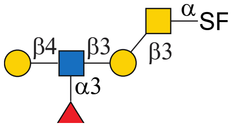

Compound **37** was prepared according to general procedure of α1-3 fucosylation with Hp3FT. After lyophilization, **37** was obtained as white solid. Compound was characterized by HPLC, T_R =16.215 min. ^{1}H NMR (600 MHz, D_2O) δ 7.97 - 7.87 (m, 2H), 7.78 - 7.64 (m, 2H), 7.57 - 7.40 (m, 4H), 5.14 (d, J = 4.0 Hz, 1H), 4.69 - 4.60 (m, 2H), 4.48 (d, J = 7.9 Hz, 1H), 4.38 - 4.18 (m, 4H), 4.14 (s, 1H), 4.11 (s, 1H), 4.02 - 3.79 (m, 12H), 3.78 - 3.48 (m, 16H), 2.02 (s, 3H), 1.95 (s, 3H), 1.20 (d, J = 6.6 Hz, 3H). HRMS, $C_{52}H_{73}N_3O_{29}$, Calcd for: 1203.4330; found $[M-H]^-$ 1202.4408.

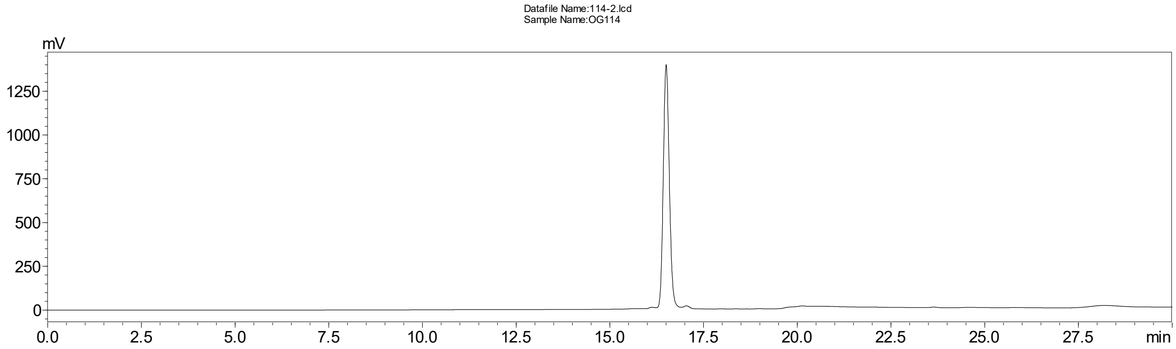

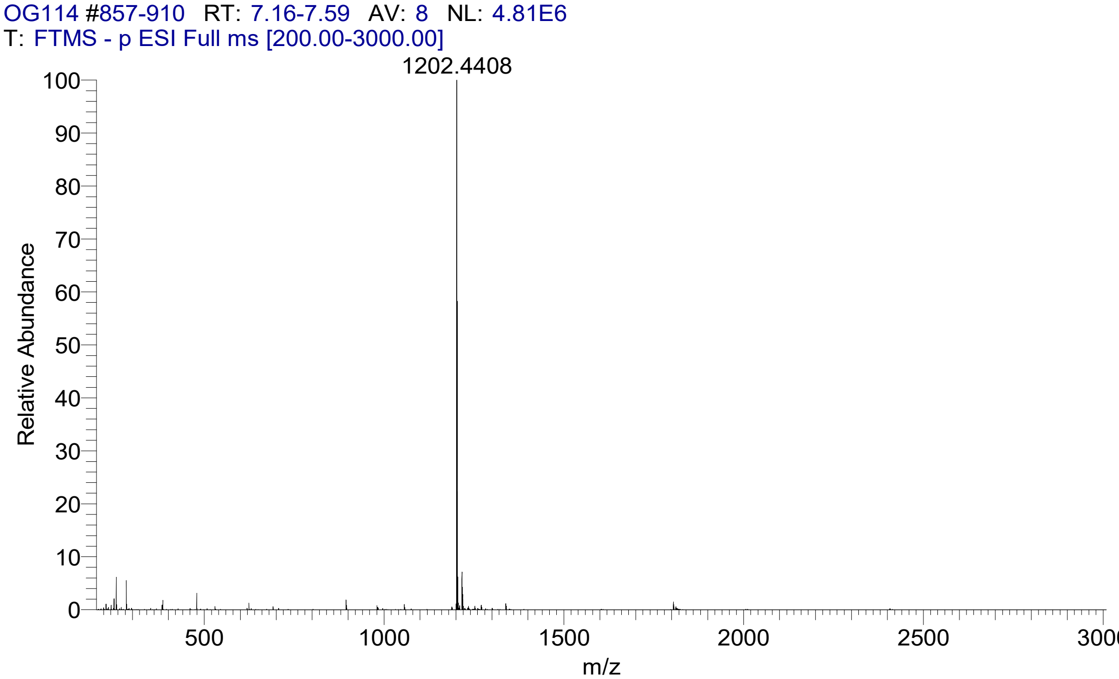

Neu5Acα2-3Galβ1-4(Fucα1-3)GlcNAcβ1-3Galβ1-3GalNAcα-Ser-Fmoc (38)

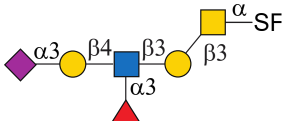

Compound **38** was prepared according to general procedure of α2-3 sialylation with PmST1-M144D. After lyophilization, **38** was obtained as white solid. Compound was characterized by HPLC, T_R =15.145 min. ^{1}H NMR (600 MHz, D_2O) δ 7.91 - 7.82 (m, 2H), 7.74 - 7.59 (m, 2H), 7.49 - 7.33 (m, 4H), 5.07 (d, *J* = 4.1 Hz, 1H), 4.69 - 4.55 (m, 3H), 4.48 (d, *J* = 7.6 Hz, 1H), 4.33 - 4.21 (m, 3H), 4.20 - 4.01 (m, 4H), 3.97- 3.70 (m, 15H), 3.69 - 3.42 (m, 18H), 2.71 (dd, *J* = 12.6, 4.8 Hz, 1H), 1.98 (s, 3H), 1.95 (s, 3H), 1.88 (s, 3H), 1.75 (t, *J* = 12.3 Hz, 1H), 1.12 (d, *J* = 6.5 Hz, 3H). HRMS, $C_{63}H_{90}N_4O_{37}$, Calcd for: 1494.5284; found $[M-H]^-$ 1493.520.

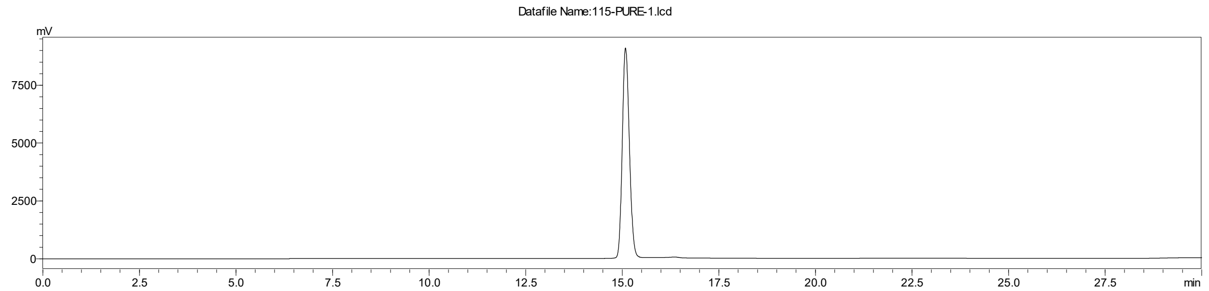

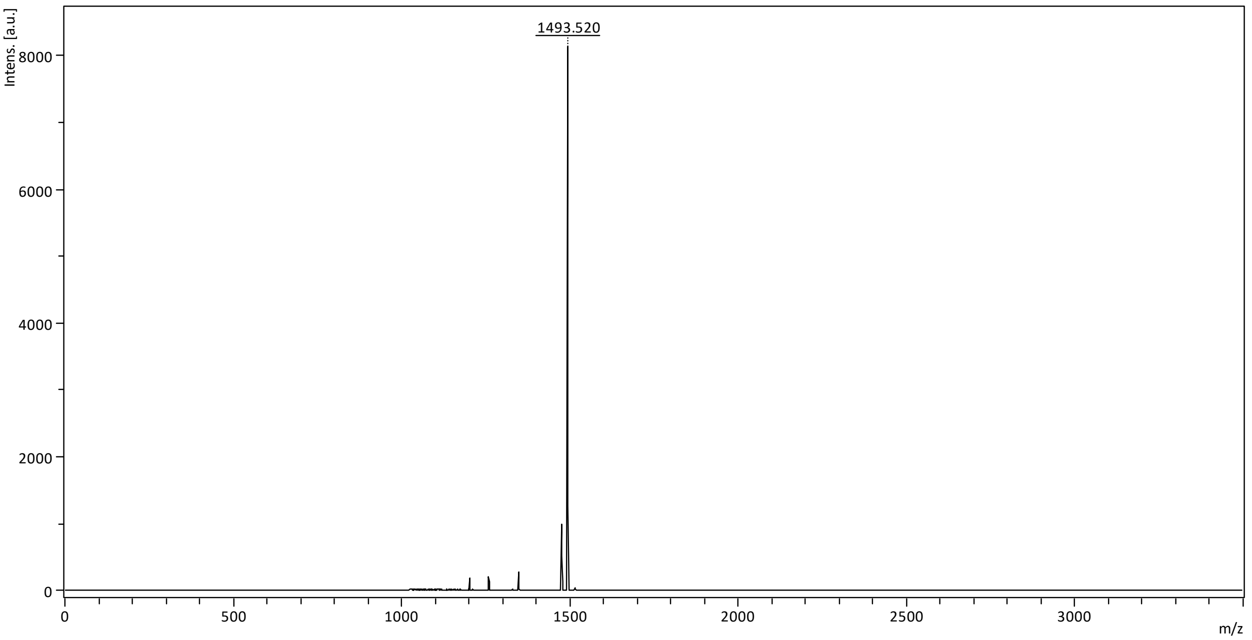

Neu5Acα2-3Galβ1-4GlcNAcβ1-3Galβ1-3GalNAcα-Ser-Fmoc (39)

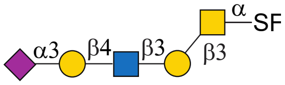

Compound **39** was prepared according to general procedure of α2-3 sialylation with PmST1-M144D. After lyophilization, **39** was obtained as white solid. Compound was characterized by HPLC, T_R = 15.693 min. ^{1}H NMR (600 MHz, D_2O) δ 7.96 - 7.88 (m, 2H), 7.78 - 7.65 (m, 2H), 7.55 - 7.40 (m, 4H), 4.69 (t, *J* = 8.5 Hz, 1H), 4.63 (s, 1H), 4.57 (d, *J* = 7.7 Hz, 1H), 4.39 - 4.18 (m, 6H), 4.03 - 3.49 (m, 35H), 2.77 (dd, *J* = 12.5, 4.62 Hz, 1H), 2.04 (s, 3H), 2.03 (s, 3H), 1.94 (s, 3H), 1.83 (t, *J* = 12.3 Hz, 1H). HRMS, $C_{57}H_{80}N_4O_{33}$, Calcd for: 1348.4705; found $[M-H]^-$ 1347.4560.

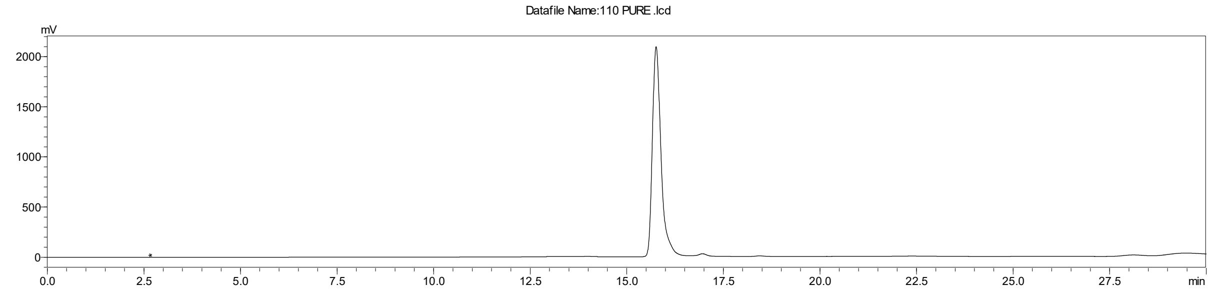

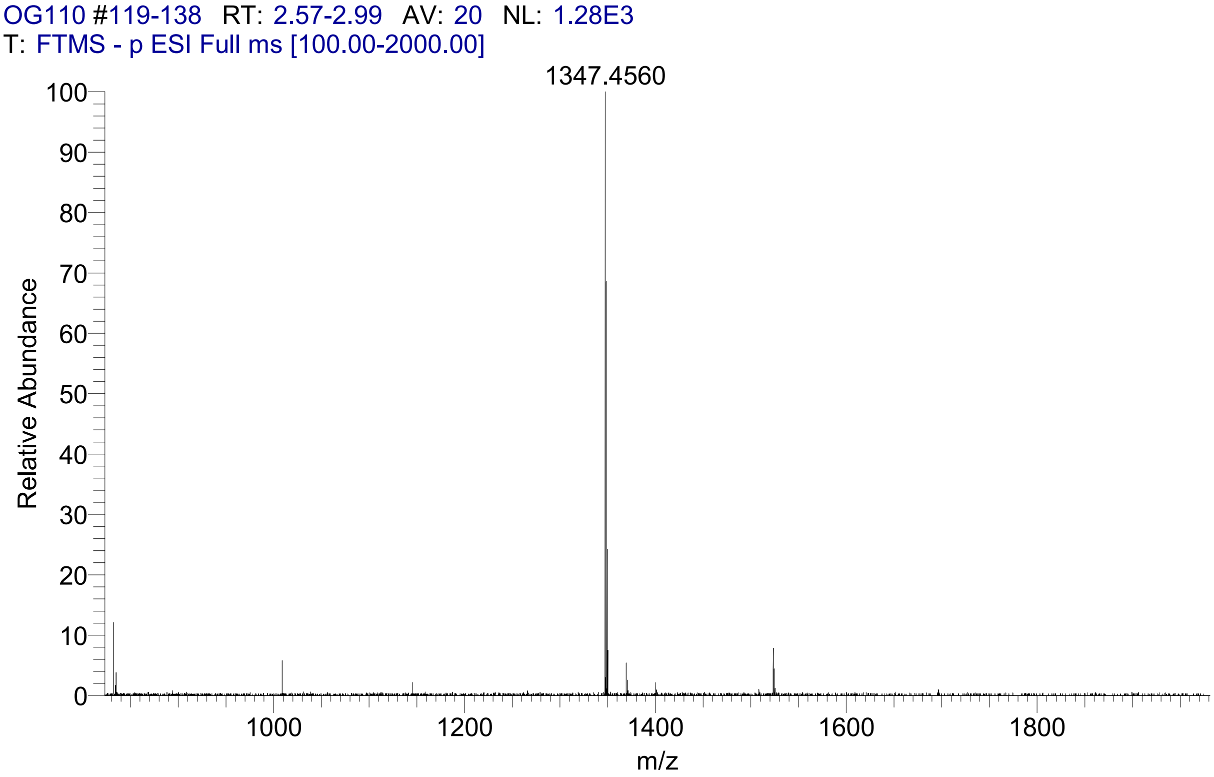

Neu5Acα2-6Galβ1-4GlcNAcβ1-3Galβ1-3GalNAcα-Ser-Fmoc (40)

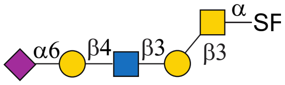

Compound **40** was prepared according to general procedure of α2-6 sialylation with PmST1-P34H/M144L. After lyophilization, **40** was obtained as white solid. Compound was characterized by HPLC, T_R = 15.645 min. ^{1}H NMR (600 MHz, D_2O) δ 7.90 - 7.81 (m, 2H), 7.72 - 7.57 (m, 2H), 7.49 - 7.34 (m, 4H), 4.71 - 4.60 (m, 1H), 4.59 - 4.53 (m, 1H), 4.40 (d, J = 8.0 Hz, 1H), 4.32 - 4.23 (m, 2H), 4.21 - 4.15 (m, 1H), 4.09 (s, 1H), 4.06 (s, 1H), 3.97 (t, J = 9.7 Hz, 1H), 3.94 - 3.44 (m, 31H), 2.61 (dd, J = 12.4 Hz, 4.5, 1H), 1.99 (s, 3H), 1.96 (s, 3H), 1.87 (s, 3H), 1.72 (t, J = 12.2 Hz, 1H). HRMS, $C_{57}H_{80}N_4O_{33}$, Calcd for: 1348.4705; found $[M-H]^-$ 1348.279.

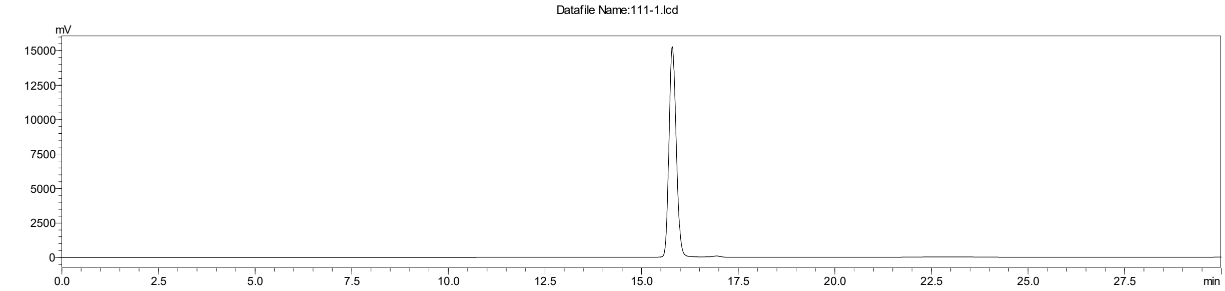

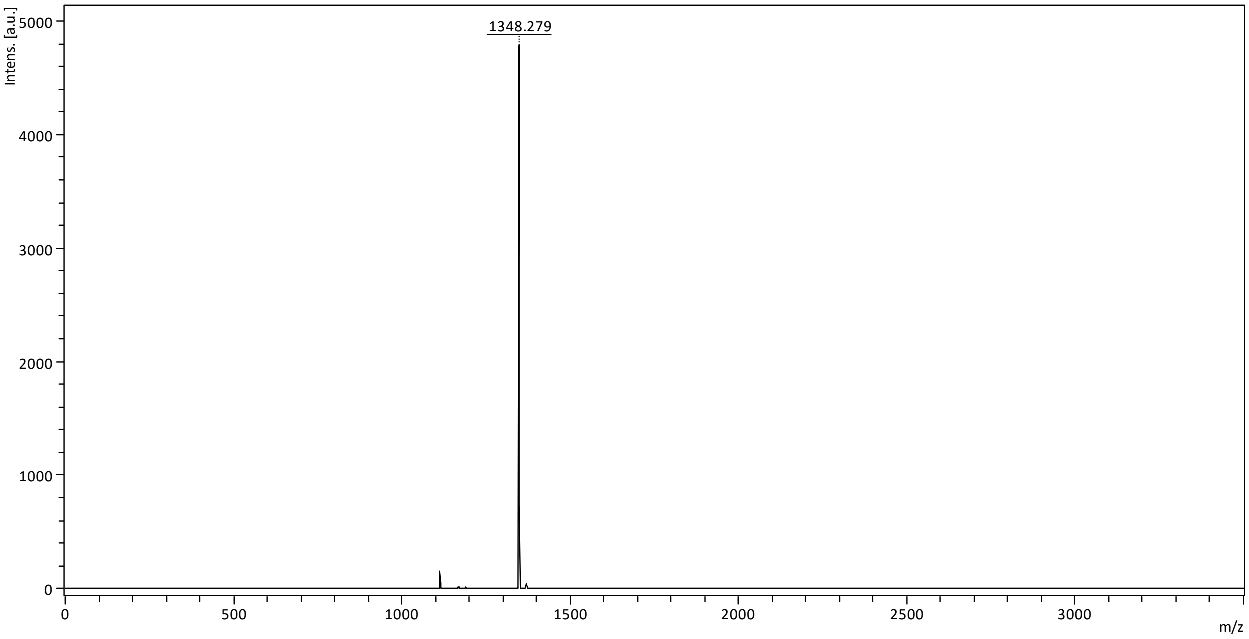

Neu5Acα2-3(GalNAcβ1-4)Galβ1-4GlcNAcβ1-3Galβ1-3GalNAcα-Ser-Fmoc (41)

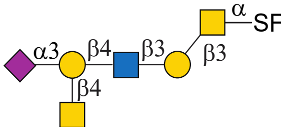

Compound **41** was prepared according to general procedure of β1-4-*N*-acetylgalatosaminylation with CgtA. After lyophilization, **41** was obtained as white solid. Compound was characterized by HPLC, T_R = 14.610 min. ^{1}H NMR (600 MHz, D_2O) δ 7.86 - 7.75 (m, 2H), 7.68 - 7.53 (m, 2H), 7.46 - 7.29 (m, 4H), 4.63 (d, *J* = 8.5 Hz, 1H), 4.62 - 4.57 (m, 2H), 4.54 - 4.50 (m, 1H), 4.48 (d, *J* = 7.9 Hz, 1H), 4.31 - 4.19 (m, 3H), 4.18 - 4.13 (m, 1H), 4.11 - 4.03 (m, 3H), 3.93 - 3.36 (m, 35H), 3.31 (t, *J* = 8.8 Hz, 1H), 2.60 (dd, *J* = 12.8, 4.7 Hz, 1H), 1.96 (s, 3H), 1.95 (s, 3H), 1.94 (s, 3H), 1.86 (s, 3H). HRMS, $C_{65}H_{93}N_5O_{38}$, Calcd for: 1551.5499; found $[M-H]^-$ 1550.5361, $[M-2H]^{2-}$ 774.7646.

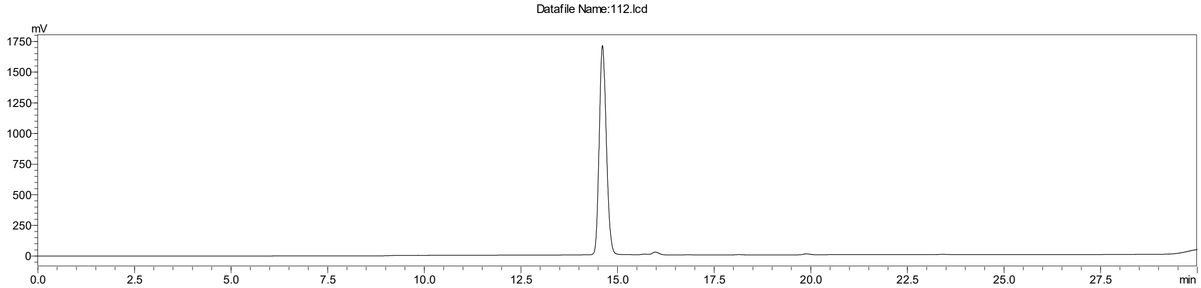

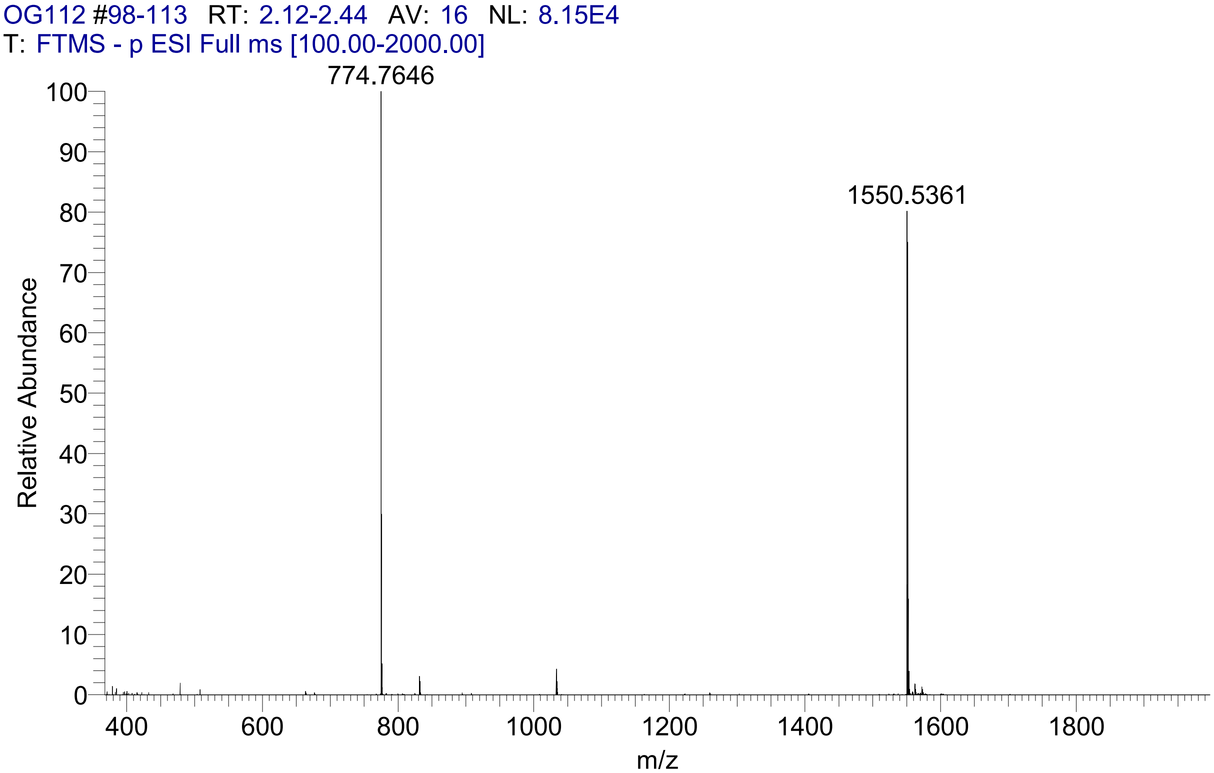

Galα1-3Galβ1-4GlcNAcβ1-3Galβ1-3GalNAcα-Ser-Fmoc (42)

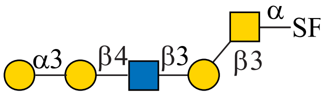

Compound **42** was prepared according to general procedure of α1-3 galactosylation with α3GalT. After lyophilization, **42** was obtained as white solid (202 mg, 93%). Compound was characterized by HPLC, T_R = 15.797 min. ^{1}H NMR (600 MHz, D_2O) δ 7.96 - 7.88 (m, 2H), 7.78 - 7.65 (m, 2H), 7.55 - 7.40 (m, 4H), 5.16 (d, *J* = 3.9 Hz, 1H), 4.57 (d, *J* = 7.8 Hz, 1H), 4.39 - 4.18 (m, 6H), 4.15 (s, 1H), 4.12 (s, 1H), 4.05 - 3.94 (m, 3H), 3.91 - 3.57 (m, 21+5H), 3.53 (t, *J* = 10.3 Hz, 1H), 2.03 (s, 3H), 1.95 (s, 3H). HRMS, $C_{52}H_{73}N_3O_{30}$, Calcd for: 1219.4279; found $[M-H]^-$ 1218.4358.

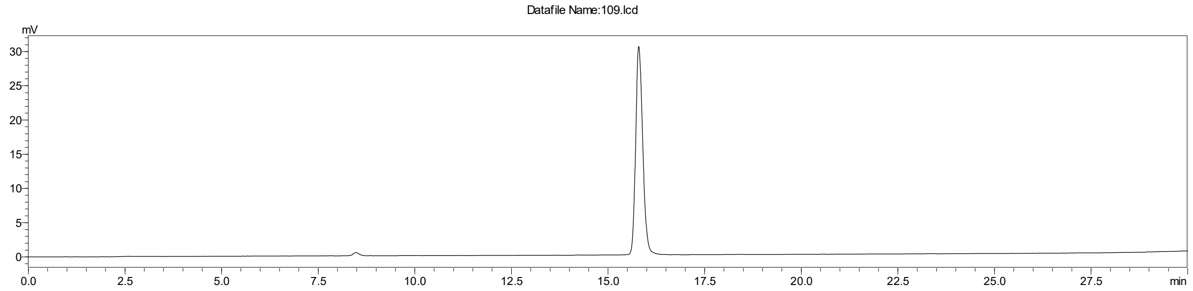

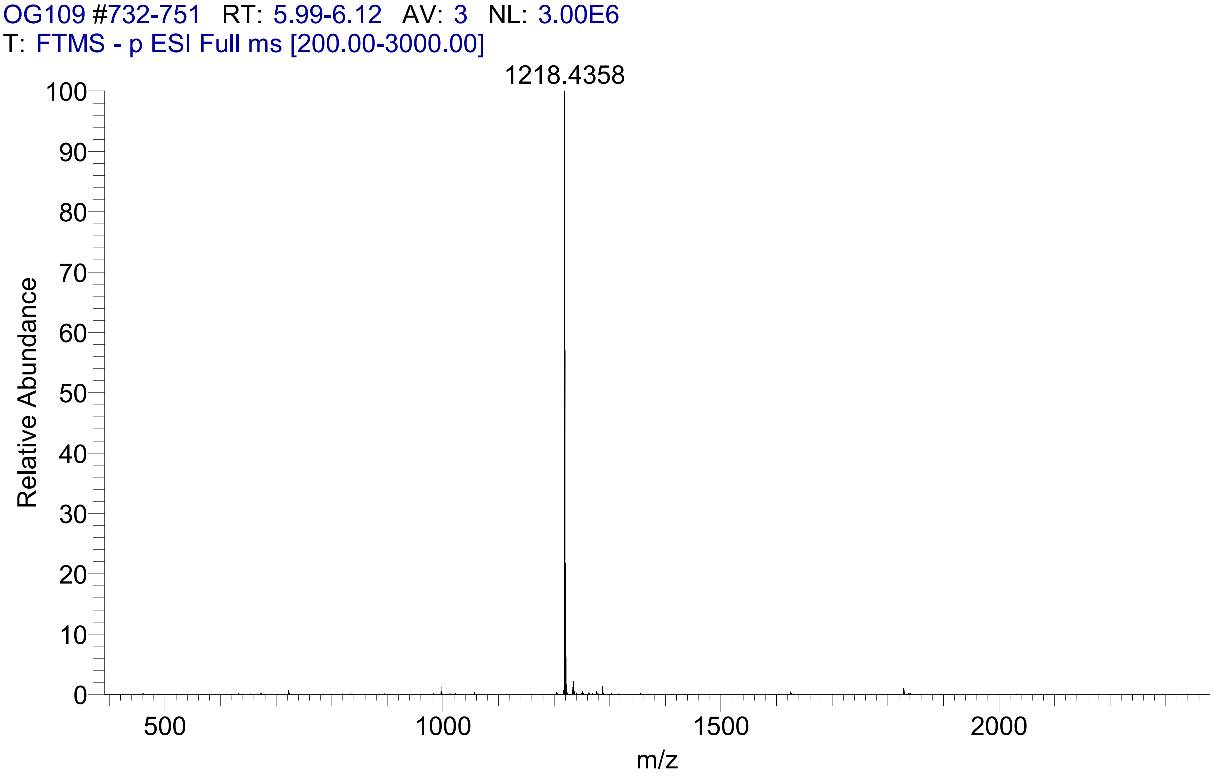

Fucα1-2Galβ1-4GlcNAcβ1-3Galβ1-3GalNAcα-Ser-Fmoc (43)

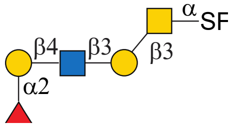

Compound **43** was prepared according to general procedure of α1-2 fucosylation with Hm2FT. After lyophilization, **43** was obtained as white solid. Compound was characterized by HPLC, T_R =15.868 min. ^{1}H NMR (600 MHz, D_2O) δ 7.93 - 7.82 (m, 2H), 7.74 - 7.59 (m, 2H), 7.53 - 7.36 (m, 4H), 5.32 (d, *J* = 3.4 Hz, 1H), 4.69 - 4.58 (m, 2H), 4.55 (d, *J* = 7.8 Hz, 1H), 4.37 - 4.18 (m, 5H), 4.14 (s, 1H), 4.11 (s, 1H), 4.02 - 3.94 (m, 1H), 3.93 - 3.42 (m, 26H), 2.04 (s, 3H), 1.94 (s, 3H), 1.24 (d, *J* = 6.6 Hz, 3H). HRMS, $C_{52}H_{73}N_3O_{29}$, Calcd for: 1203.4330; found $[M-H]^-$ 1202.4413.

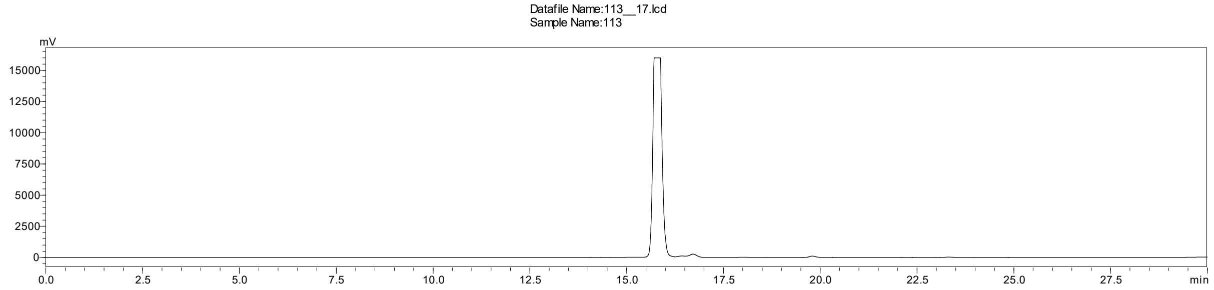

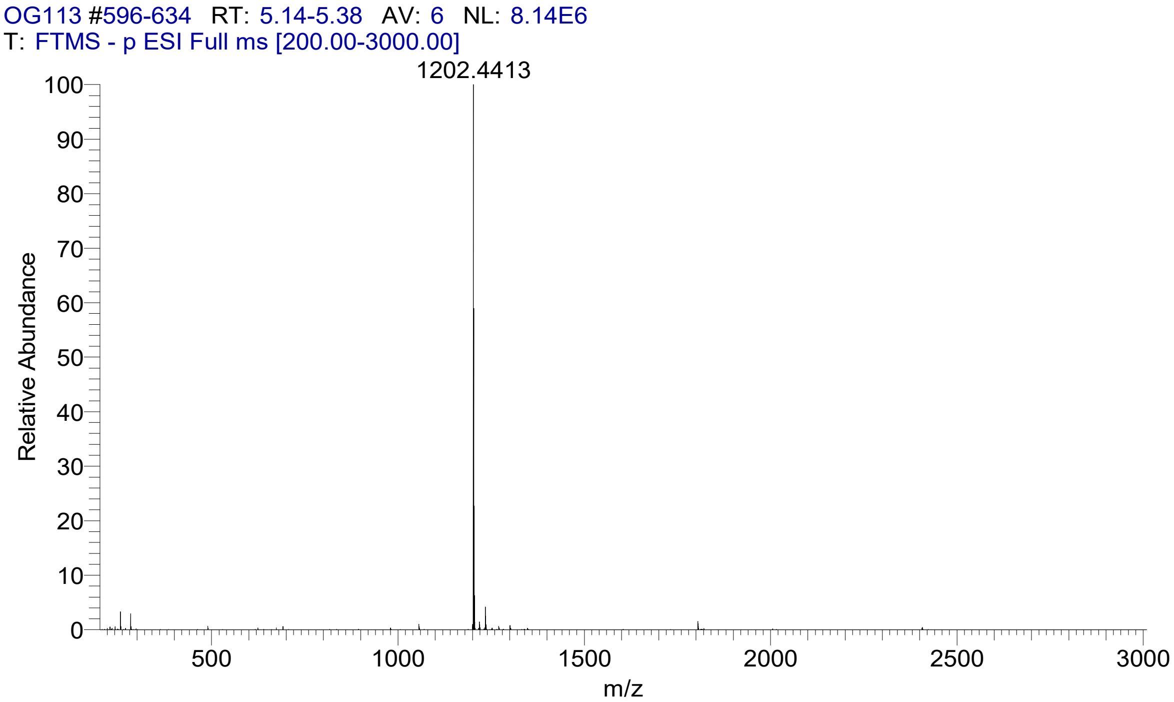

Fucα1-2Galβ1-4(Fucα1-3)GlcNAcβ1-3Galβ1-3GalNAcα-Ser-Fmoc (44)

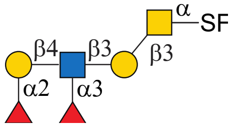

Compound **44** was prepared according to general procedure of α1-3 fucosylation with Hp3FT. After lyophilization, **44** was obtained as white solid. Compound was characterized by HPLC, T_R =15.189 min. ^{1}H NMR (600 MHz, D_2O) δ 7.97 - 7.87 (m, 2H), 7.78 - 7.61 (m, 2H), 7.55 - 7.37 (m, 4H), 5.28 (d, *J* = 3.5 Hz, 1H), 5.13 (d, *J* = 3.5 Hz, 1H), 4.52 (d, *J* = 7.5 Hz, 1H), 4.40 - 4.29 (m, 3H), 4.28 - 4.18 (m, 2), 4.14 (s, 1H), 4.11 (s, 1H), 4.06 - 3.43 (m, 30H), 2.02 (s, 3H), 2.02 (s, 3H), 1.93 (s, 3H), 1.27 (d, *J* = 6.6 Hz, 3H), 1.24 (d, *J* = 6.5 Hz, 3H). HRMS, $C_{58}H_{83}N_3O_{33}$, Calcd for: 1349.4909; found $[M-H]^-$ 1348.510.

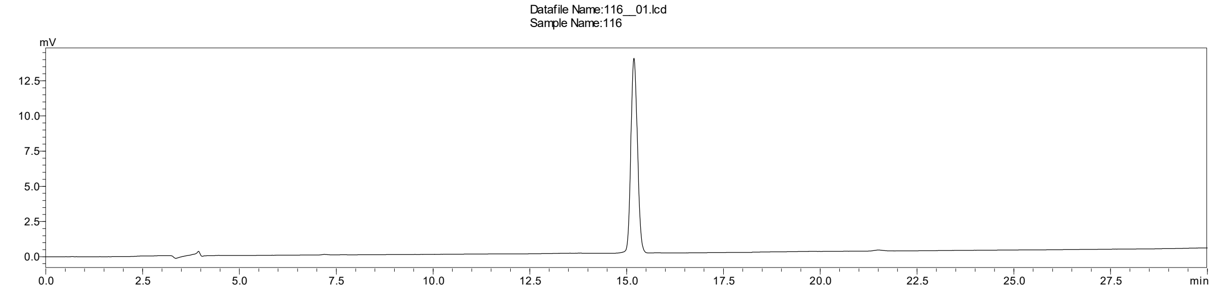

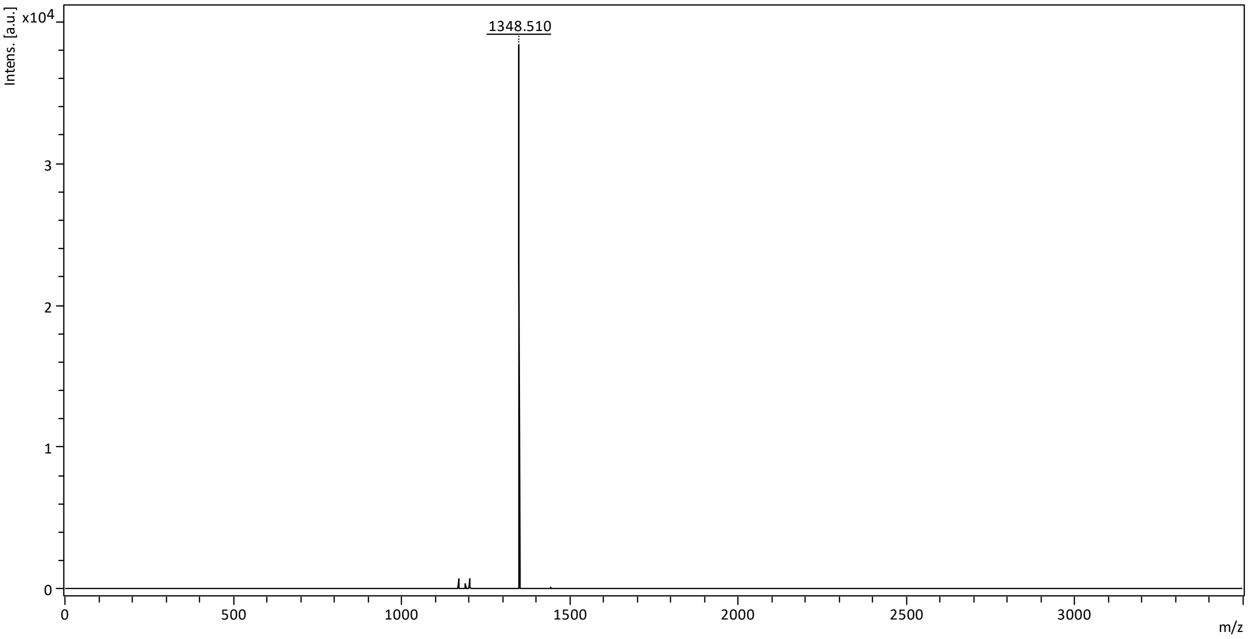

Neu5Acα2-3Galβ1-3(GlcNAcβ1-6)GalNAcα-Ser-Fmoc (45)

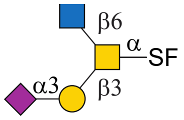

Compound **45** was prepared according to general procedure of α2-3 sialylation with PmST1-M144D. After lyophilization, **45** was obtained as white solid. Compound was characterized by HPLC, T_R =12.908 min. ^{1}H NMR (600 MHz, D_2O) δ 7.80 - 7.64 (m, 2H), 7.62 - 7.42 (m, 2H), 7.41 - 7.21 (m, 4H), 4.70 (s, 1H), 4.58 -4.43 (m, 2H), 4.40 (d, *J* = 8.4 Hz, 1H), 4.36 (m, 1H), 4.28 (s, 1H), 4.18 – 4.08 (m, 2H), 4.06 (s, 1H), 4.00 (dd, *J* = 9.8, 3.3 Hz, 1H), 3.93 - 3.27 (m, 24H), 2.69 (dd, *J* = 11.9, 3.9 Hz, 1H), 1.97 (s, 3H), 1.88 (s, 3H), 1.85 (s, 3H). HRMS, $C_{51}H_{70}N_4O_{28}$, Calcd for: 1186.4177; found $[M+Na-2H]^-$ 1208.168.

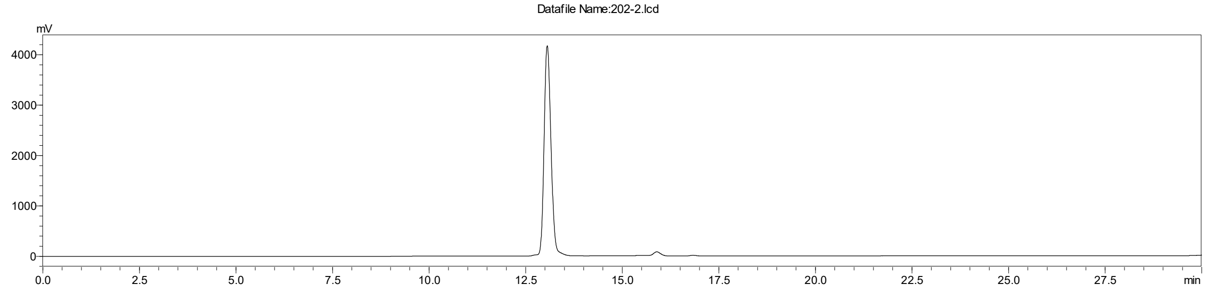

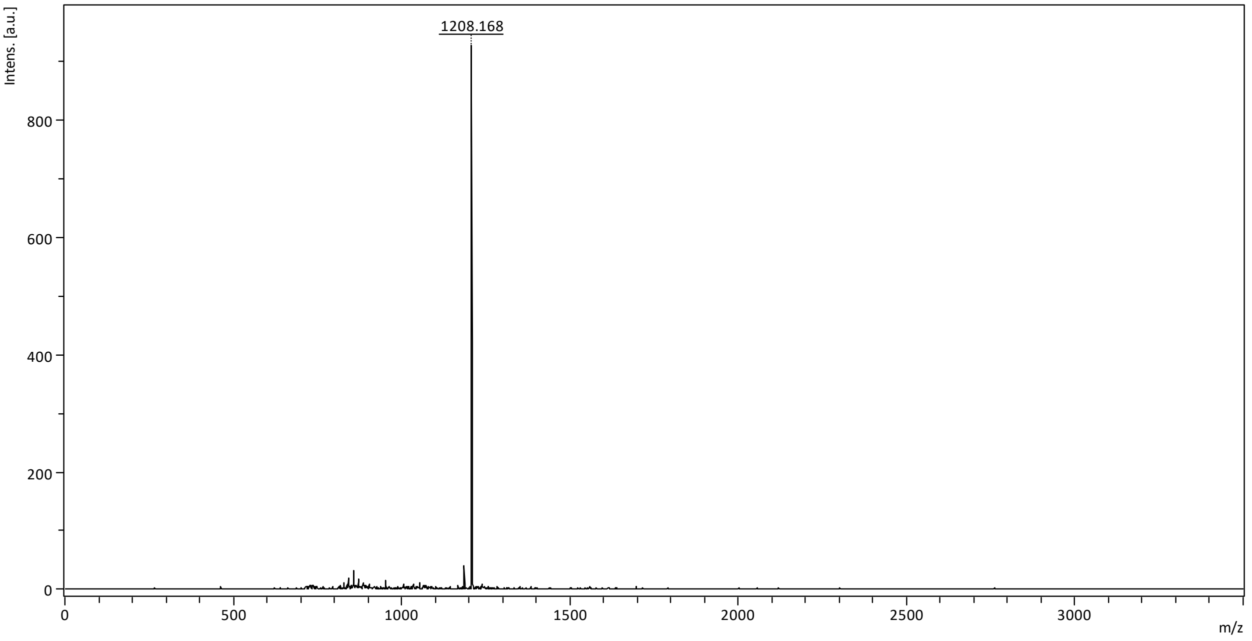

Neu5Acα2-3(GalNAcβ1-4)Galβ1-3(GlcNAcβ1-6)GalNAcα-Ser-Fmoc (46)

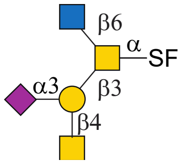

Compound **46** was prepared according to general procedure of β1-4-N-acetylgalatosaminylation with CgtA. After lyophilization, **46** was obtained as white solid. Compound was characterized by HPLC, T_R =12.060 min. ^{1}H NMR (600 MHz, D_2O) δ 7.96 - 7.85 (m, 2H), 7.77 – 7.61 (m, 2H), 7.55 - 7.40 (m, 4H), 4.49 (d, J = 8.5 Hz, 1H), 4.43 (m, 1H), 4.38 – 4.28 (m, 2H), 4.26- 4.18 (m, 1H), 4.14 – 4.04 (m, 3H), 4.02 –3.36 (m, 32H), 3.32 (t, J = 8.7 Hz, 1H), 2.68 (dd, 12.3, 4.5, 1H), 2.05 (s, 3H), 2.01 (s, 3H), 1.96 (s, 3H), 1.93 (s, 3H). HRMS, $C_{59}H_{83}N_5O_{33}$, Calcd for: 1389.497; found $[M-H]^-$ 1388.276.

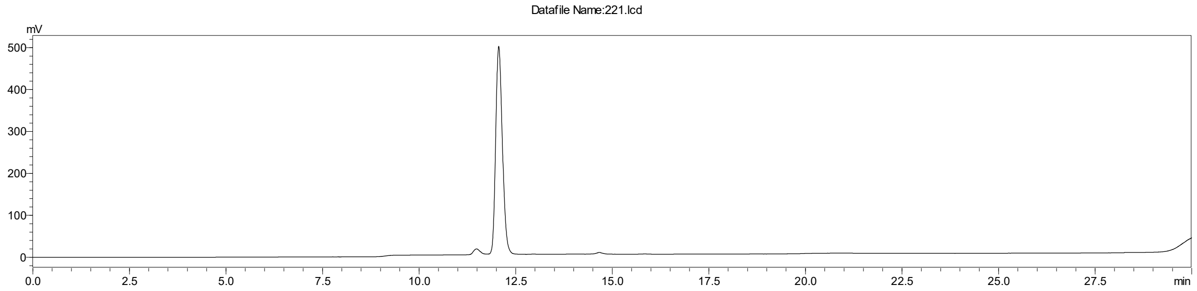

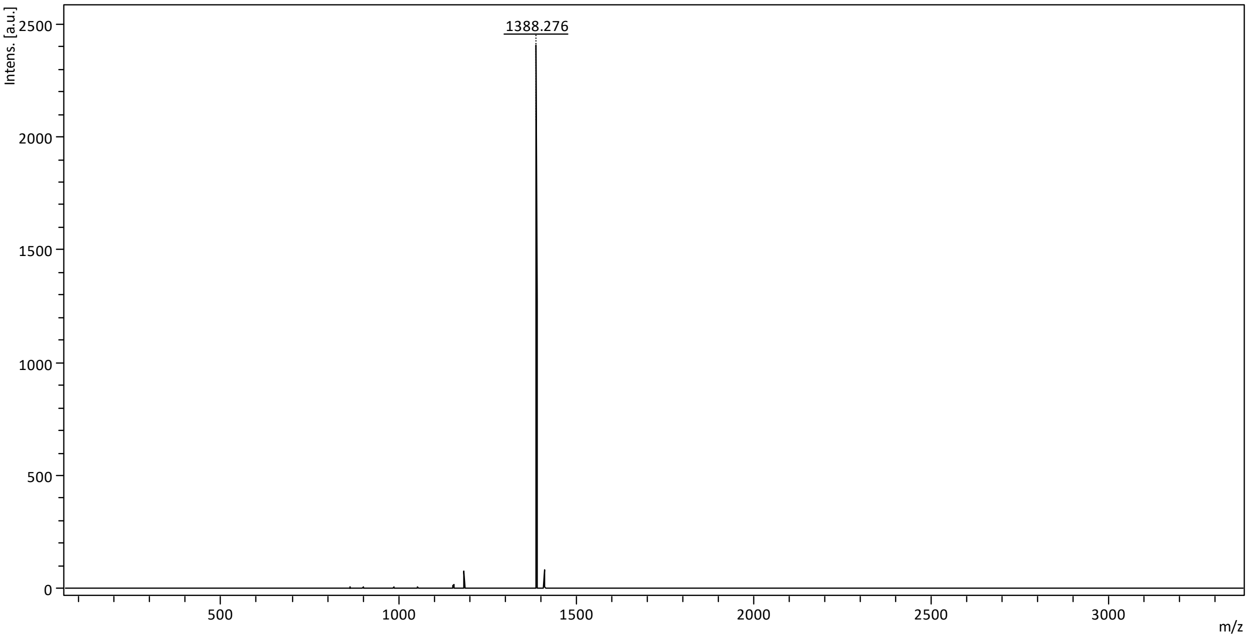

Neu5Acα2-3(GalNAcβ1-4)Galβ1-3(Galβ1-4GlcNAcβ1-6)GalNAcα-Ser-Fmoc (47)

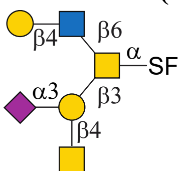

Compound **47** was prepared according to general procedure of β1-4 galactosylation with NmLgtB. After lyophilization, **47** was obtained as white solid. Compound was characterized by HPLC, T_R =10.723 min. ^{1}H NMR (600 MHz, D_2O) δ 7.94 - 7.82 (m, 2H), 7.77 – 7.61 (m, 2H), 7.54 - 7.35 (m, 4H), 4.70 (d, *J* = 8.5 Hz, 1H), 4.68 – 4.63 (m, 1H), 4.60 – 4.54 (m, 1H), 4.46 (d, *J* = 8.0 Hz, 1H), 4.42 (d, *J* = 7.9 Hz, 1H), 4.38 – 4.28 (m, 3H), 4.23-4.14 (m, 1H), 4.10 – 4.00 (m, 3H), 3.96 –3.40 (m, 33H), 3.28 (t, *J* = 8.8 Hz, 1H), 3.16 (dd, 14.7, 7.3, 1H), 2.63 (dd, 12.6, 4.6, 1H), 2.00 (s, 3H), 1.97 (s, 3H), 1.92 (s, 3H), 1.90 (s, 3H), 1.24 (t, *J* = 7.3 Hz, 1H). HRMS, $C_{65}H_{93}N_5O_{38}$, Calcd for: 1551.5499; found $[M-H]^-$ 1550.207.

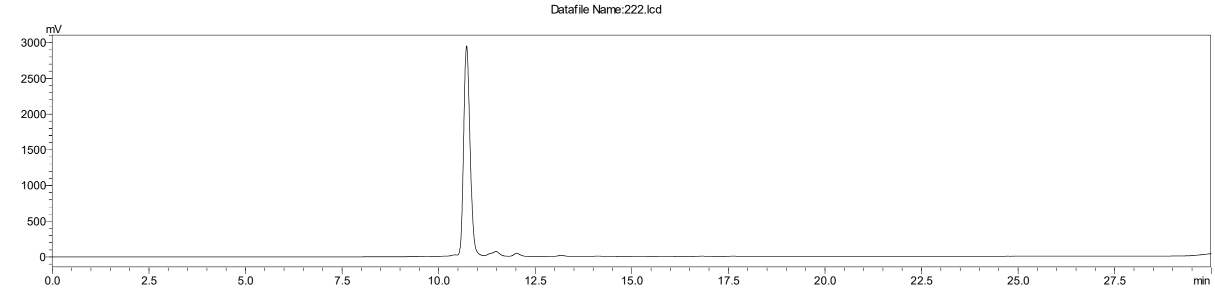

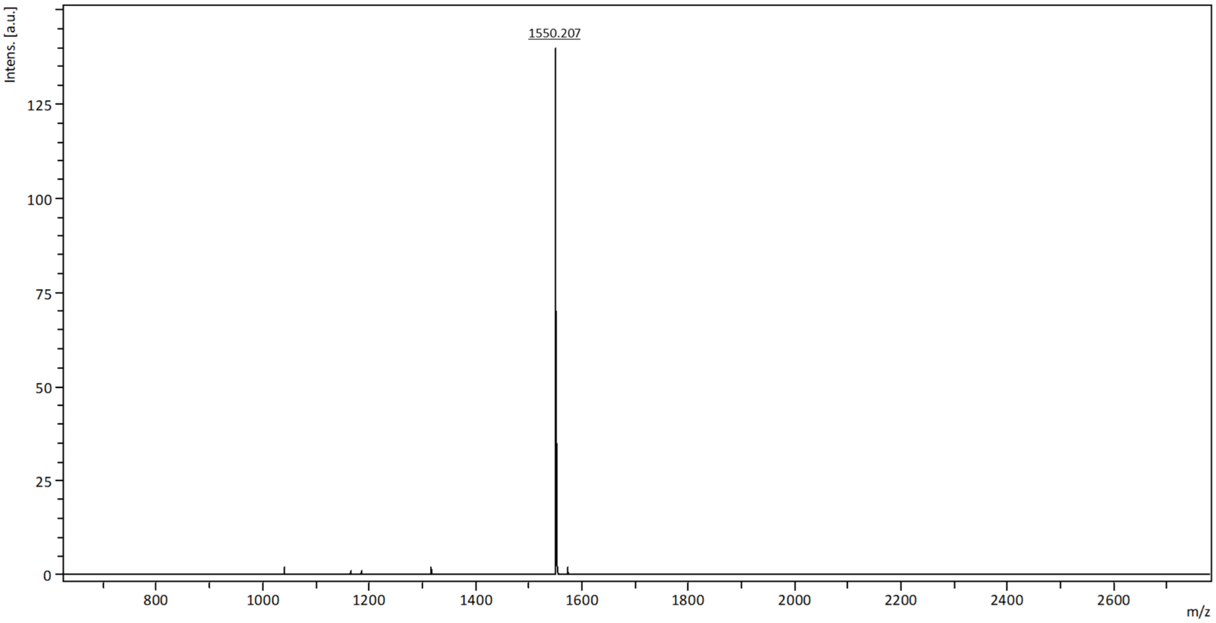

Neu5Acα2-3(GalNAcβ1-4)Galβ1-3(Neu5Acα2-3Galβ1-4GlcNAcβ1-6)GalNAcα-Ser-Fmoc (48)

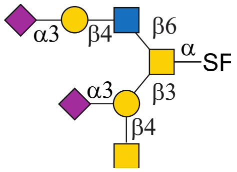

Compound **48** was prepared according to general procedure of α2-3 sialylation with PmST1-M144D. After lyophilization, **48** was obtained as white solid. Compound was characterized by HPLC, T_R =9.379 min. ^{1}H NMR (600 MHz, D_2O) δ 7.98 - 7.88 (m, 2H), 7.79 – 7.64 (m, 2H), 7.57 - 7.40 (m, 4H), 4.64 (m, 1H), 4.53 – 4.48 (m, 1H), 4.46 (d, *J* = 7.9 Hz, 1H), 4.43 – 4.33 (m, 2H), 4.26- 4.18 (m, 1H), 4.14 – 4.05 (m, 4H), 3.01 –3.46 (m, 38H), 3.33 (t, *J* = 8.8 Hz, 1H), 3.16 (dd, 14.7, 7.3, 1H), 2.76 (dd, 12.5, 4.6, 1H), 2.68 (m, 1H), 2.04 (m, 6H), 2.01 (s, 3H), 1.96 (s, 3H), 1.94 (s, 3H), 1.83 (t, *J* = 12.2 Hz, 1H). HRMS, $C_{76}H_{110}N_6O_{46}$, Calcd for: 1842.6453; found $[M-2H]^{2-}$ 920.3254.

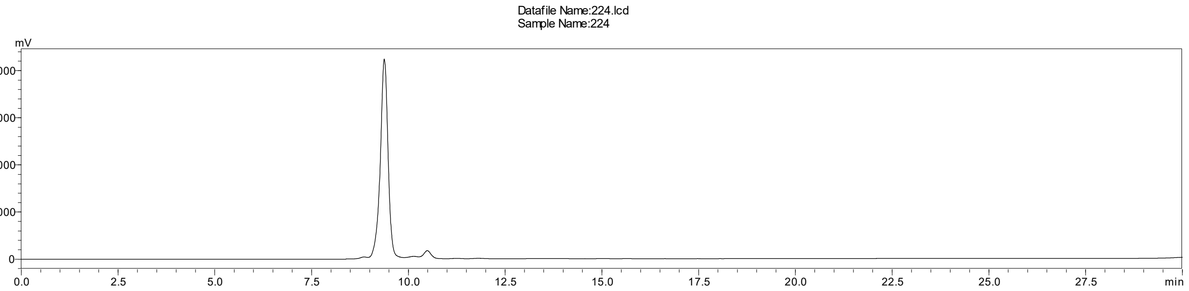

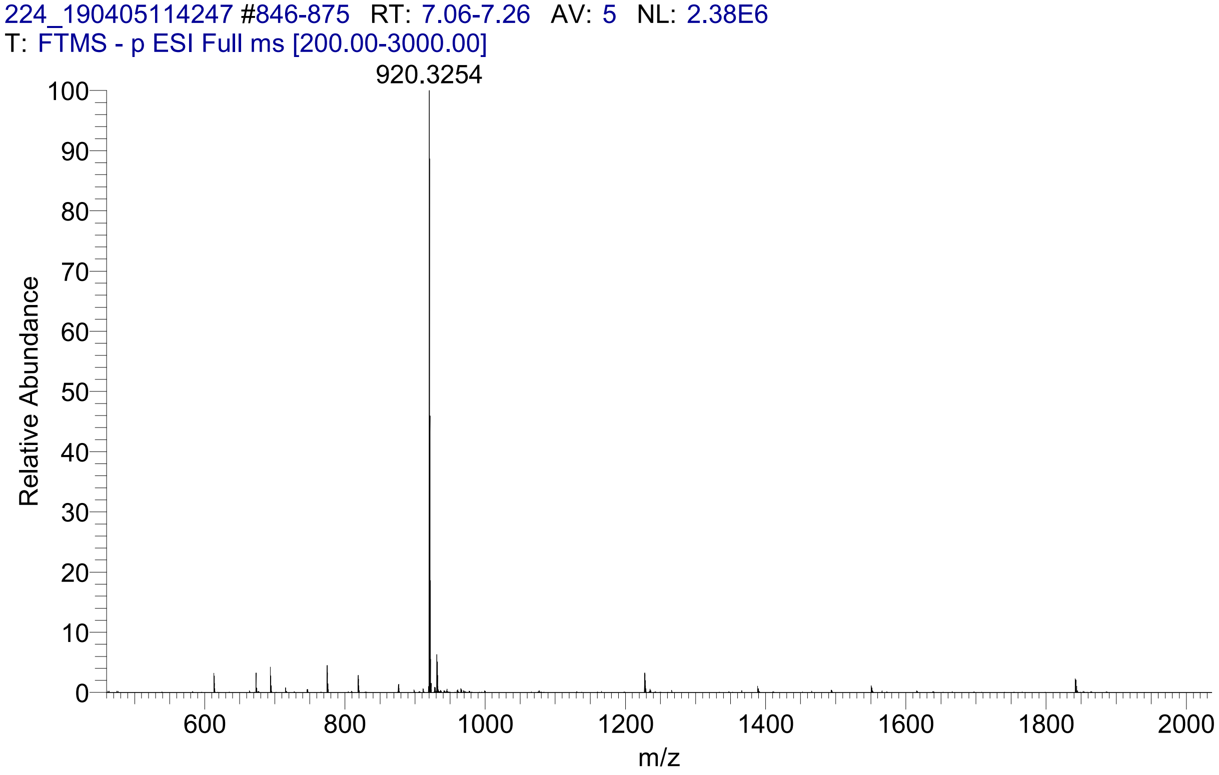

Neu5Acα2-3(GalNAcβ1-4)Galβ1-3[Neu5Acα2-3(GalNAcβ1-4)Galβ1-4GlcNAcβ1-6]GalNAcα-Ser-Fmoc (49)

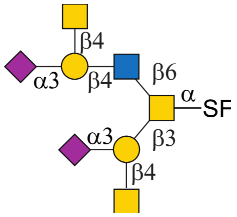

Compound **49** was prepared according to general procedure of β1-4-N-acetylgalatosaminylation with CgtA. After lyophilization, **49** was obtained as white solid. Compound was characterized by HPLC, T_R =8.328 min. ^{1}H NMR (600 MHz, D_2O) δ 7.94 - 7.83 (m, 2H), 7.76 – 7.60 (m, 2H), 7.54 - 7.35 (m, 4H), 4.64 (m, 1H), 4.53 – 4.48 (m, 1H), 4.46 (d, *J* = 7.9 Hz, 1H), 4.43 – 4.33 (m, 2H), 4.26- 4.18 (m, 1H), 4.14 – 4.05 (m, 4H), 3.01 –3.46 (m, 38H), 3.33 (t, *J* = 8.8 Hz, 1H), 3.16 (dd, 14.7, 7.3, 1H), 2.76 (dd, 12.5, 4.6, 1H), 2.68 (m, 1H), 2.04 (m, 6H), 2.01 (s, 3H), 1.96 (s, 3H), 1.94 (s, 3H), 1.83 (t, *J* = 12.2 Hz, 1H). HRMS, $C_{84}H_{123}N_7O_{51}$, Calcd for: 2045.7246; found [M-H]$^-$ 2044.693.

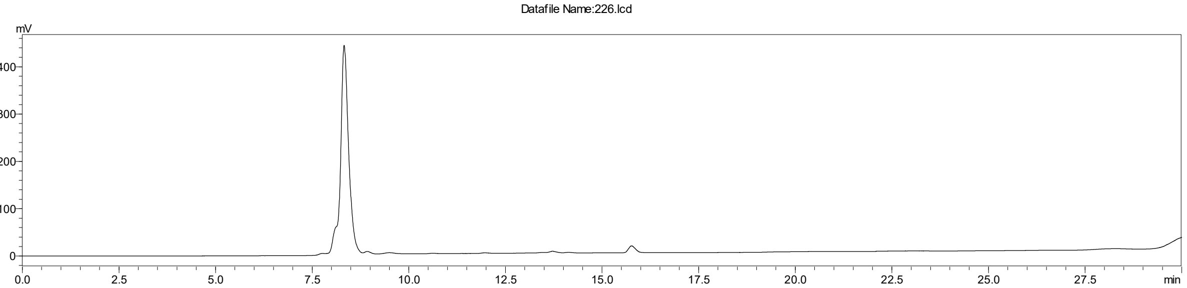

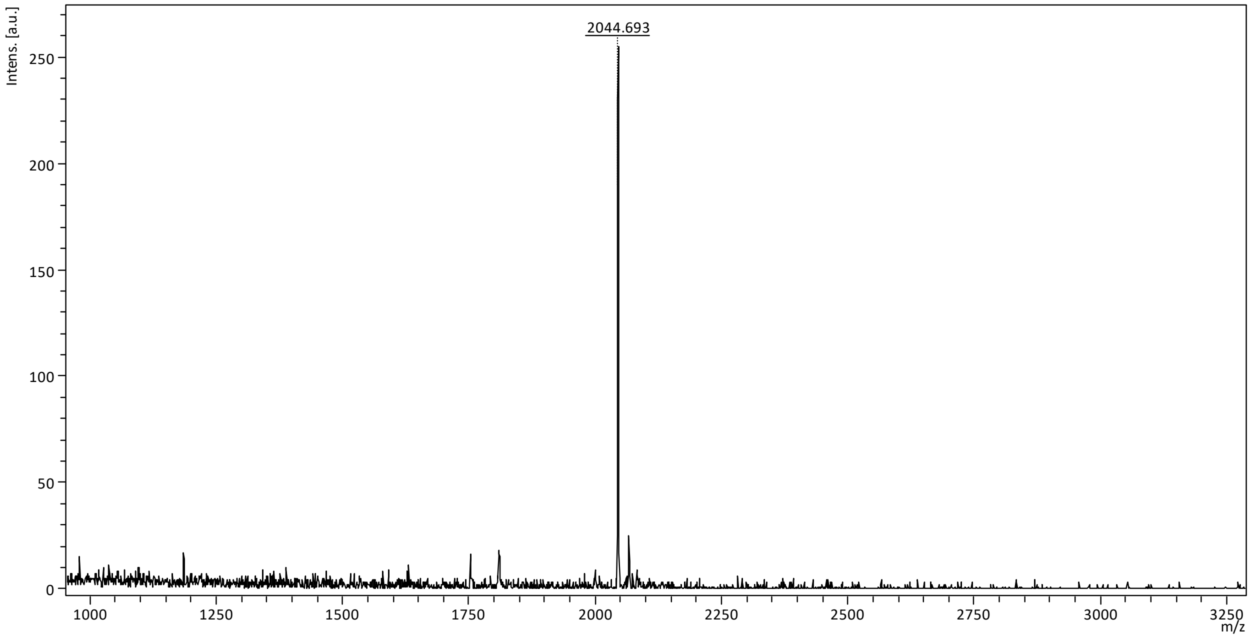

Neu5Acα2-3Galβ1-3(Galβ1-4GlcNAcβ1-6)GalNAcα-Ser-Fmoc (50)

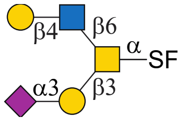

Compound **50** was prepared according to general procedure of β1-4 galactosylation with NmLgtB. After lyophilization, **50** was obtained as white solid. Compound was characterized by HPLC, T_R =11.331 min. ^{1}H NMR (600 MHz, D_2O) δ 7.95 - 7.82 (m, 2H), 7.77 - 7.58 (m, 2H), 7.55 - 7.36 (m, 4H), 4.51 (dd, *J* = 8.4, 4.6 Hz,1H), 4.46 (m, 1H), 4.42 -4.34 (m, 2H), 4.22 (m, 1H), 4.15 (s, 1H), 4.11 -4.04 (m, 2H), 4.03 - 3.48 (m, 32H), 2.77 (dd, *J* = 12.1, 4.6 Hz, 1H), 2.05(s, 3H), 1.95 (s, 3H), 1.94 (s, 3H), 1.86 (t, *J* = 12.5 Hz, 1H). HRMS, $C_{57}H_{80}N_4O_{33}$, Calcd for: 1348.4705; found $[M-H]^-$ 1347.475.

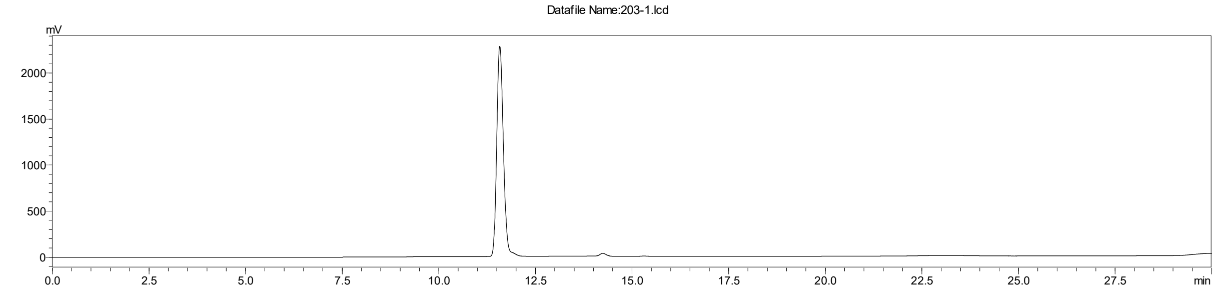

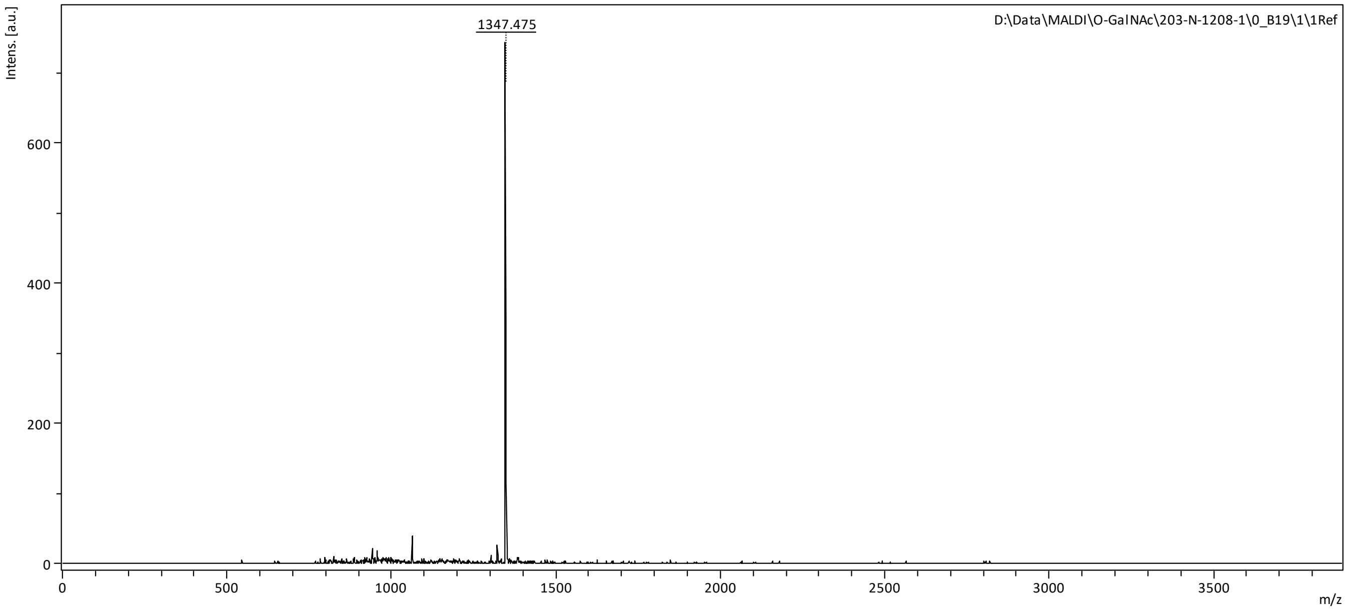

Neu5Acα2-3Galβ1-3(Neu5Acα2-3Galβ1-4GlcNAcβ1-6)GalNAcα-Ser-Fmoc (51)

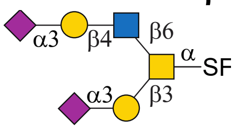

Compound **51** was prepared according to general procedure of α2-3 sialylation with PmST1-M144D. After lyophilization, **51** was obtained as white solid. Compound was characterized by HPLC, T_R =10.192 min. ^{1}H NMR (600 MHz, D_2O) δ 7.95 - 7.82 (m, 2H), 7.77 - 7.58 (m, 2H), 7.55 - 7.37 (m, 4H), 4.48 (d, *J* = 7.8 Hz, 1H), 4.46 (d, *J* = 7.8 Hz, 1H), 4.11 (m, 1H), 4.31 (m, 1H), 4.21 (m, 1H), 4.13 -4.04 (m, 2H), 4.03 - 3.44 (m, 39H), 2.76 (dd, *J* = 12.2, 4.6 Hz, 2H), 2.10 - 2.02 (m, 6H), 2.01 - 1.92 (m, 6H), 1.87 (t, *J* = 12.2 Hz, 2H). HRMS, $C_{68}H_{97}N_5O_{41}$, Calcd for: 1639.5659; found $[M-2H]^{2-}$ 818.7860.

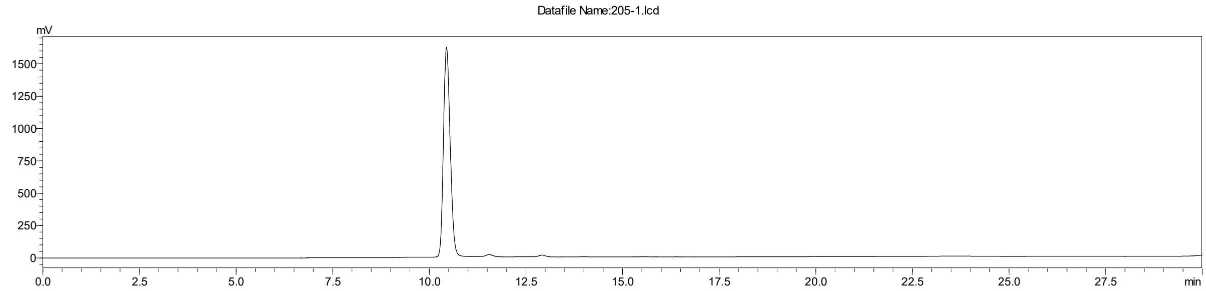

205_190322225755 #695-710 RT: 6.14-6.19 AV: 3 NL: 2.02E6
T: FTMS - p ESI Full ms [200.00-2000.00]

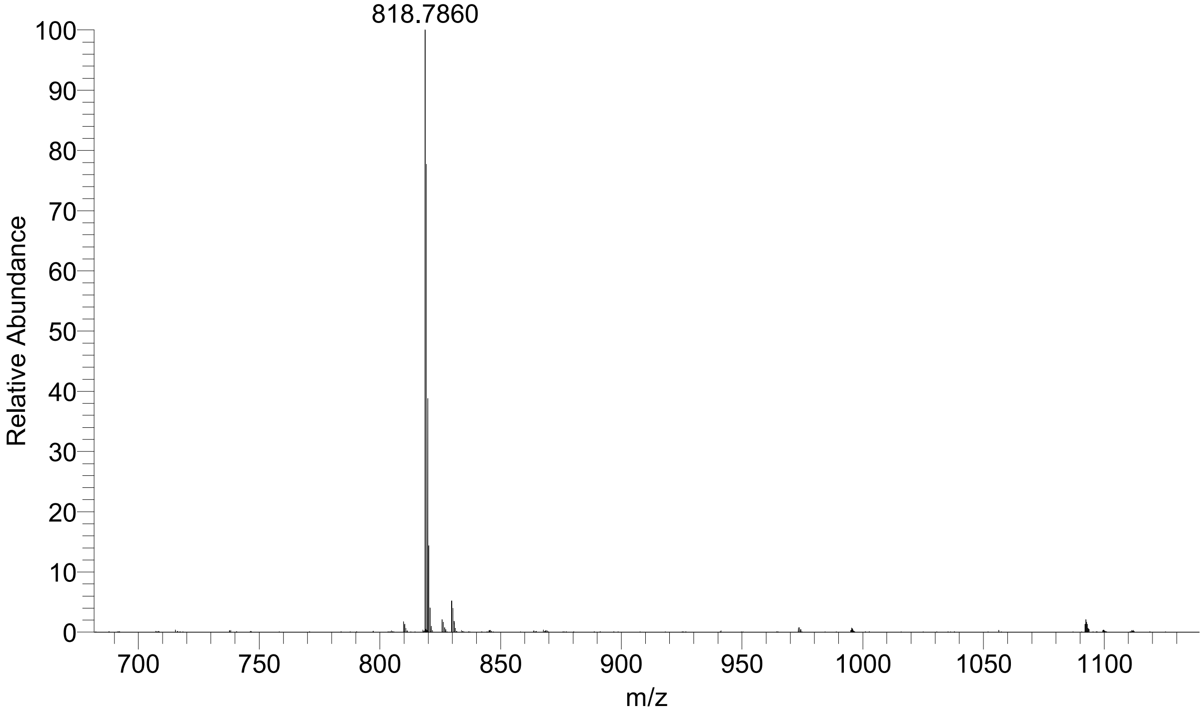

Neu5Acα2-3Galβ1-3[Galβ1-4(Fucα1-3)GlcNAcβ1-6]GalNAcα-Ser-Fmoc (52)

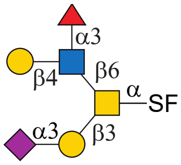

Compound **52** was prepared according to general procedure of α1-3 fucosylation with Hp3FT. After lyophilization, **52** was obtained as white solid. Compound was characterized by HPLC, T_R =10.031 min. ^{1}H NMR (600 MHz, D_2O) δ 7.95 - 7.82 (m, 2H), 7.77 - 7.58 (m, 2H), 7.53 - 7.37 (m, 4H), 5.09 (d, *J* = 3.6 Hz, 1H), 4.61 (t, *J* = 6.2 Hz, 1H), 4.51 (d, *J* = 8.0 Hz, 1H), 4.45 (d, *J* = 7.8 Hz, 1H), 4.39 (s, 1H), 4.35 (m, 1H), 4.30 (m, 1H), 4.21 (m, 1H), 4.14 (d, *J* = 3.1 Hz, 1H), 4.07 (dd, *J* = 9.6, 3.3 Hz, 2H), 4.02 - 3.54 (m, 35H), 2.76 (dd, *J* = 12.2, 4.6 Hz, 1H), 2.08 - 2.02 (m, 3H), 2.01 - 1.91 (m, 6H), 1.17 (d, *J* = 6.6 Hz, 3H). HRMS, $C_{63}H_{90}N_4O_{37}$, Calcd for: 1494.5284; found $[M-H]^-$ 1493.5409, $[M-2H]^{2-}$ 746.2663.

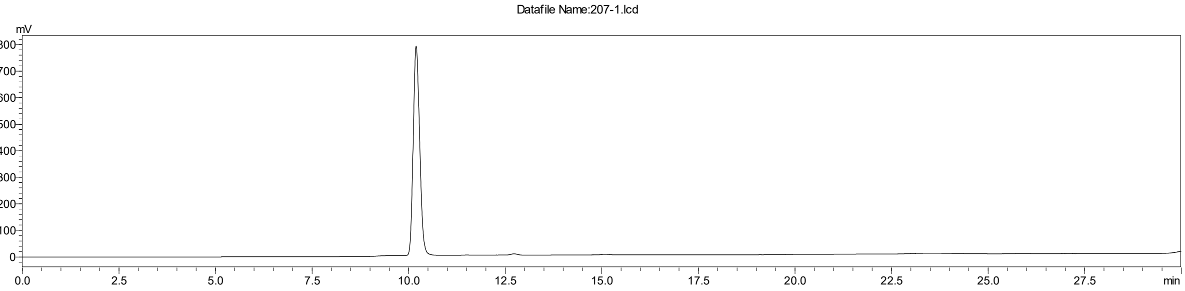

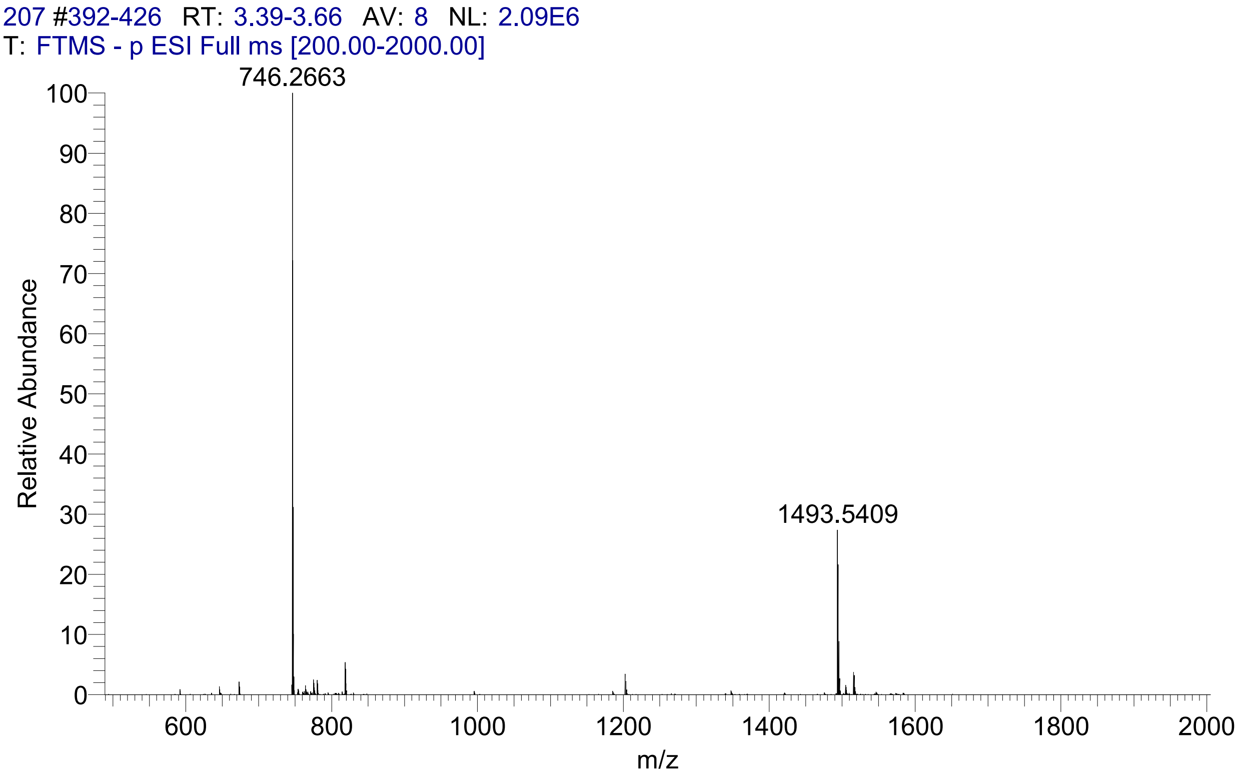

Neu5Acα2-6Galβ1-3(GlcNAcβ1-6)GalNAcα-Ser-Fmoc (53)

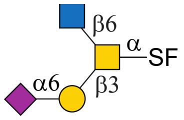

Compound **53** was prepared according to general procedure of α2-6 sialylation with Pd2,6ST. After lyophilization, **3** was obtained as white solid. Compound was characterized by HPLC, T_R =13.455 min. ^1H NMR (600 MHz, D_2O) δ 7.83 - 7.69 (m, 2H), 7.68 - 7.49 (m, 2H), 7.47 - 7.30 (m, 4H), 4.57 (m, 1H), 4.51 (d, *J* = 8.5 Hz, 1H), 4.48 (d, *J* = 8.4 Hz, 1H), 4.38 (s, 1H), 4.21 (m, 1H), 4.17 (dd, *J* = 10.9, 3.6 Hz, 1H), 4.09 - 4.04 (m, 1H), 3.97- 3.37 (m, 26H), 2.70 (dd, *J* = 12.6, 4.6 Hz, 1H), 2.09 - 2.02 (m, 3H), 1.99 (s, 3H), 1.92 (s, 3H), 1.17 (t, *J* = 12.7 Hz, 1H). HRMS, $C_{51}H_{70}N_4O_{28}$, Calcd for: 1186.4177; found $[M-H]^-$ 1185.994.

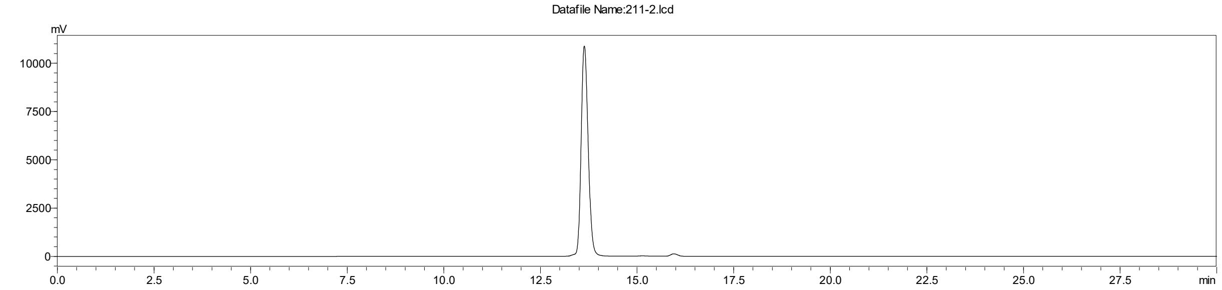

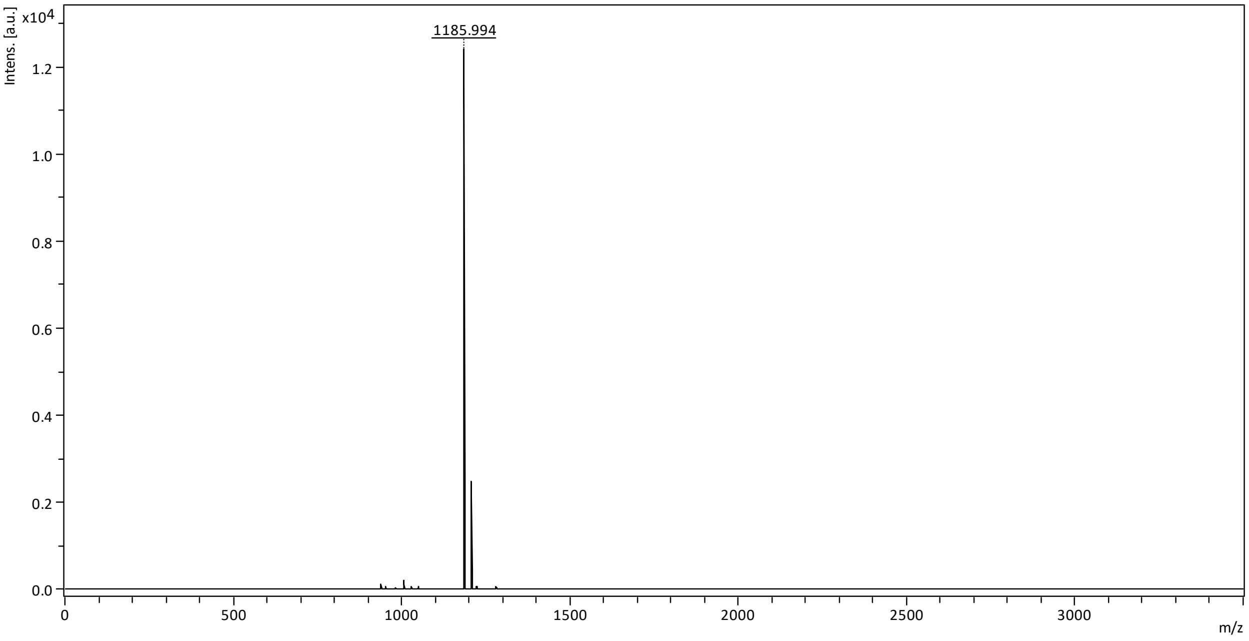

Neu5Acα2-6Galβ1-3(Galβ1-4GlcNAcβ1-6)GalNAcα-Ser-Fmoc (54)

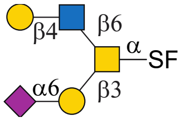

Compound **54** was prepared according to general procedure of β1-4 galactosylation with NmLgtB. After lyophilization, **54** was obtained as white solid. Compound was characterized by HPLC, T_R =11.926 min. ^{1}H NMR (600 MHz, D_2O) δ 7.91 - 7.77 (m, 2H), 7.75 - 7.56 (m, 2H), 7.54 - 7.34 (m, 4H), 4.66 (m, 1H), 4.61 (m, 1H), 4.53 (d, *J* = 8.2 Hz, 1H), 4.41 (s, 1H), 4.38 (d, *J* = 7.7 Hz, 1H), 4.29 (m, 1H), 4.21 - 4.13 (m, 2H), 4.17 (dd, *J* = 10.9, 3.6 Hz, 1H), 4.01 – 3.41 (m, 32H), 2.69 (dd, *J* = 12.5, 4.5 Hz, 1H), 2.03 (s, 3H), 1.99 (s, 3H), 1.93 (s, 3H), 1.71 (t, *J* = 11.7 Hz, 1H). HRMS, Calcd for: 1348.4705; found $[M-H]^-$ 1348.465.

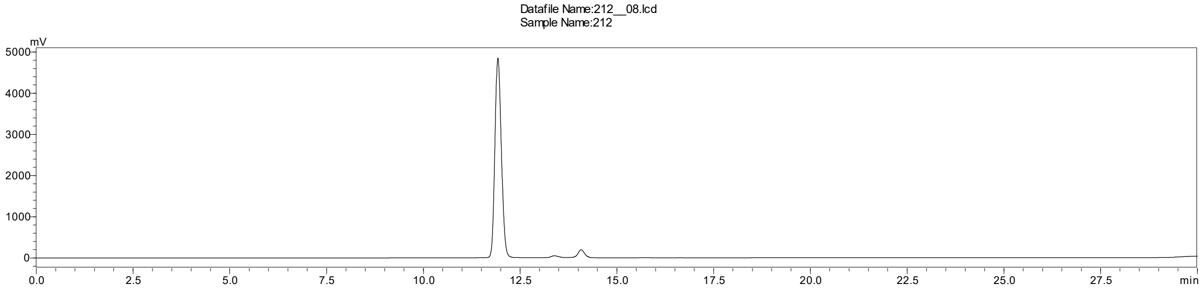

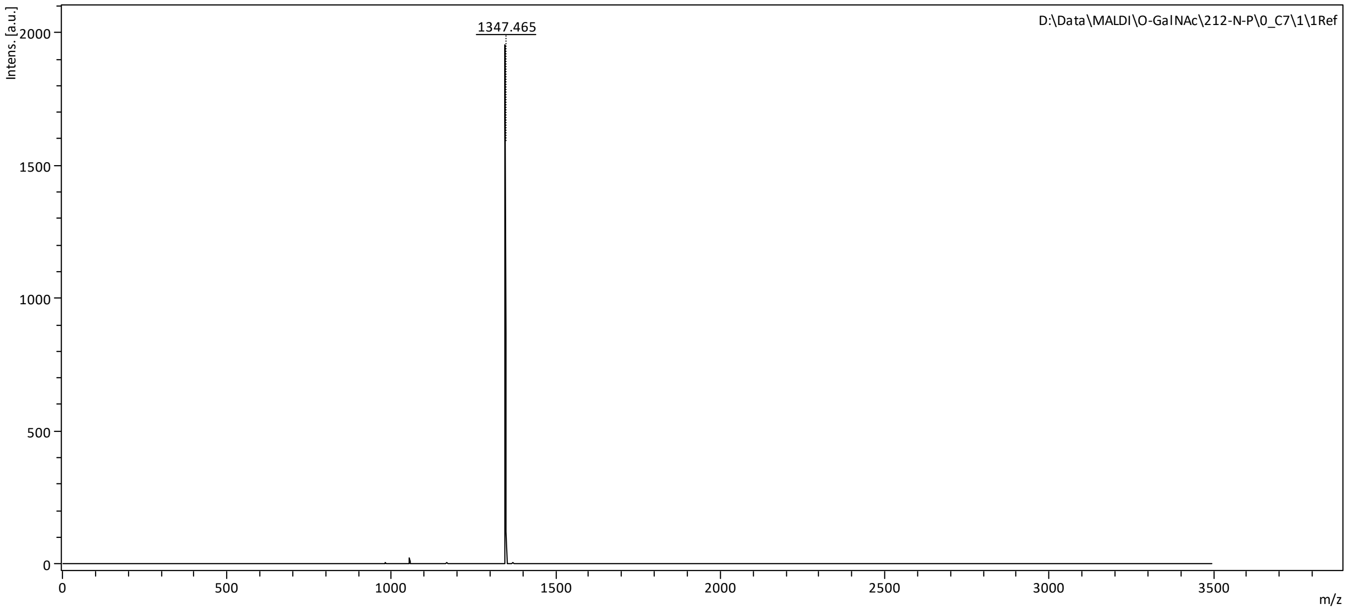

Neu5Acα2-6Galβ1-3(Neu5Acα2-6Galβ1-4GlcNAcβ1-6)GalNAcα-Ser-Fmoc (55)

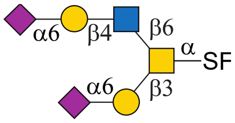

Compound **55** was prepared according to general procedure of α2-6 sialylation with Pd2,6ST. After lyophilization, **55** was obtained as white solid. Compound was characterized by HPLC, T_R =10.175 min. ^{1}H NMR (600 MHz, D_2O) δ 7.97 - 7.87 (m, 2H), 7.79 - 7.62 (m, 2H), 7.58 - 7.40 (m, 4H), 4.68 - 4.61 (m, 2H), 4.56 (d, *J* = 7.7 Hz, 1H), 4.42 – 4.35 (m, 2H), 4.33 (d, *J* = 7.7 Hz, 1H), 4.22 (m, 1H), 4.12 (m, 1H), 4.05 - 4.45 (m, 39H), 2.73 (d, *J* = 11.8 Hz, 1H), 2.68 (d, *J* = 10.8 Hz, 1H), 2.08 - 1.97 (m, 9H), 1.93 (s, 3H), 1.75 (t, *J* = 12.3 Hz, 1H), 1.67 (t, *J* = 12.2 Hz, 1H). HRMS, $C_{68}H_{97}N_5O_{41}$, Calcd for: 1639.5659; found $[M-H]^-$ 1638.5747; $[M-2H]^{2-}$ 818.7841.

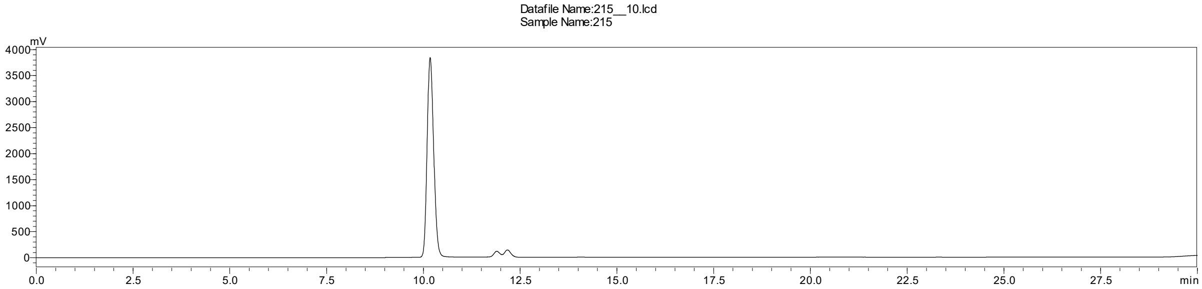

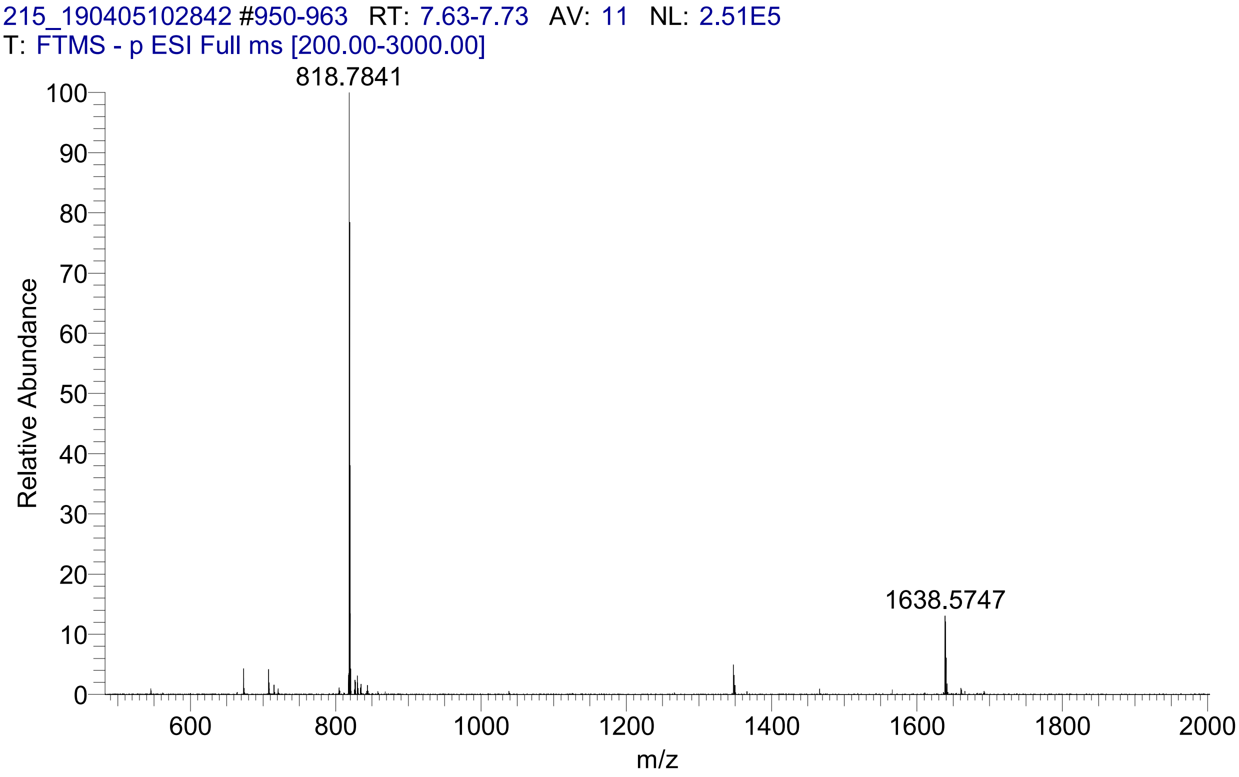

Neu5Acα2-6Galβ1-3(Neu5Acα2-3Galβ1-4GlcNAcβ1-6)GalNAcα-Ser-Fmoc (56)

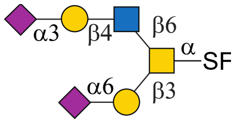

Compound **56** was prepared according to general procedure of α2-3 sialylation with PmST1-M144D. After lyophilization, **56** was obtained as white solid. Compound was characterized by HPLC, T_R =10.691 min. ^{1}H NMR (600 MHz, D_2O) δ 7.88 - 7.76 (m, 2H), 7.69 - 7.52 (m, 2H), 7.47 - 7.29 (m, 4H), 4.72 (s, 1H), 4.59 (s, 1H), 4.54 (s, 1H), 4.41 (m, 1H), 4.38 - 4.17 (m, 4H), 4.16 - 3.94 (m, 4H), 3.93 – 3.34 (m, 35H), 2.67 (d, *J* = 12.2 Hz, 1H), 2.61 (d, *J* = 12.4 Hz, 1H), 1.94 (s, 3H), 1.93 (s, 3H), 1.89 (s, 3H), 1.84 (s, 3H), 1.75 (t, *J* = 13.2 Hz, 1H), 1.59 (t, *J* = 9.6 Hz, 1H). HRMS, $C_{68}H_{97}N_5O_{41}$, Calcd for: 1639.5659; found $[M-2H]^{2-}$ 818.7839.

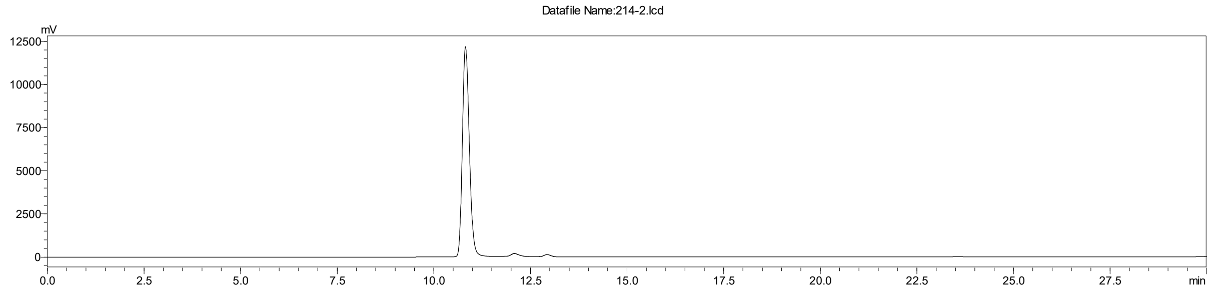

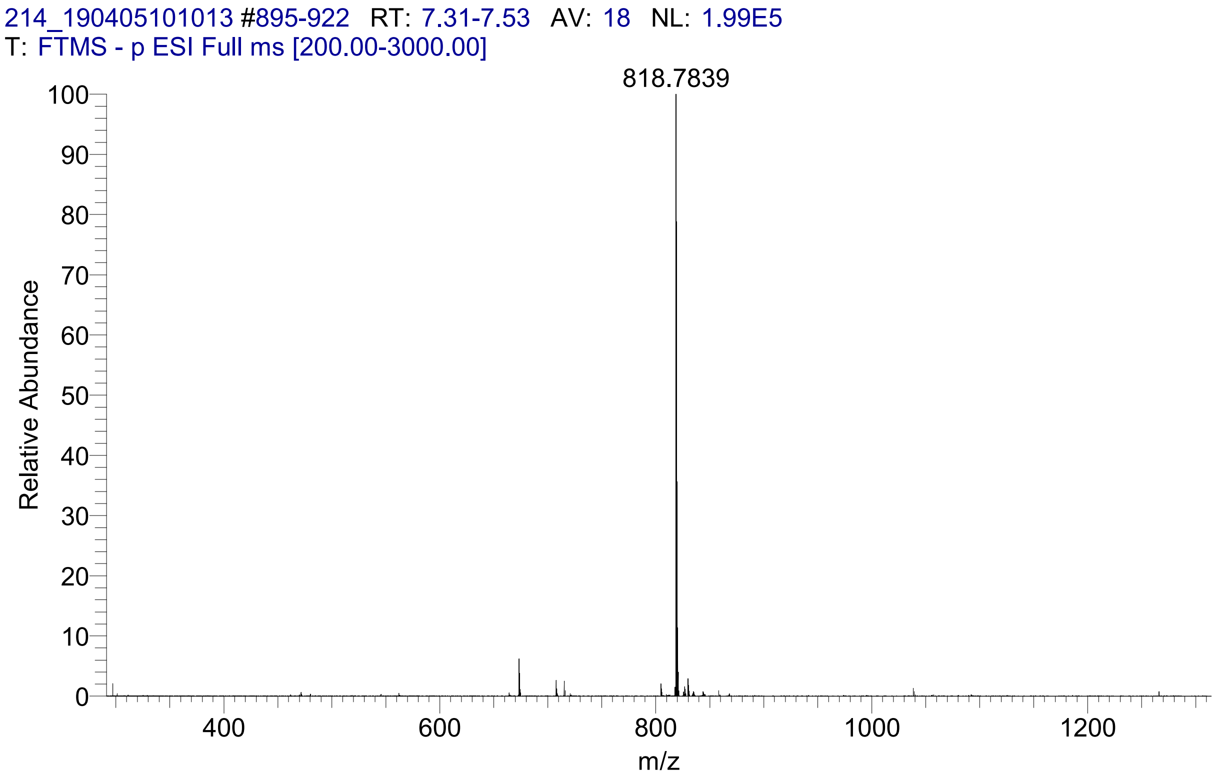

Neu5Acα2-6Galβ1-3[Neu5Acα2-3(GalNAc β1-4)Galβ1-4GlcNAcβ1-6]GalNAcα-Ser-Fmoc (57)

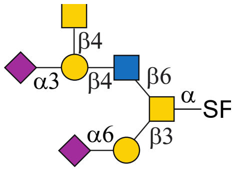

Compound **57** was prepared according to general procedure of β1-4-N-acetylgalatosaminylation with CgtA. After lyophilization, **57** was obtained as white solid. Compound was characterized by HPLC, T_R =9.586 min. ^{1}H NMR (600 MHz, D_2O) δ 7.87 (dd, J = 22.3, 8.1 Hz, 2H), 7.76 - 7.59 (m, 2H), 7.53 - 7.36 (m, 4H), 4.69 (d, J = 8.4 Hz, 2H), 4.62 (m, 1H), 4.52 (d, J = 8.2 Hz, 1H), 4.45 (d, J = 7.9 Hz, 1H), 4.41 (s, 1H), 4.34 - 4.25 (m, 2H), 4.23 - 4.12 (m, 3H), 4.10 (m, 1H), 4.01 (s, 1H), 3.99 – 3.43 (m, 43 H), 2.70 (dd, J = 12.9, 4.6 Hz, 1H), 2.68 (dd, J = 13.2, 4.5 Hz, 1H), 2.05 (s, 3H), 2.04 (s, 3H), 2.03 (s, 3H), 2.01 – 1.92 (m, 6H), 1.73 (t, J = 12.5 Hz, 1H). HRMS, $C_{76}H_{110}N_6O_{46}$, Calcd for: 1843.6453; found $[M-H]^-$ 1842.6609, $[M-2H]^{2-}$ 920.3250.

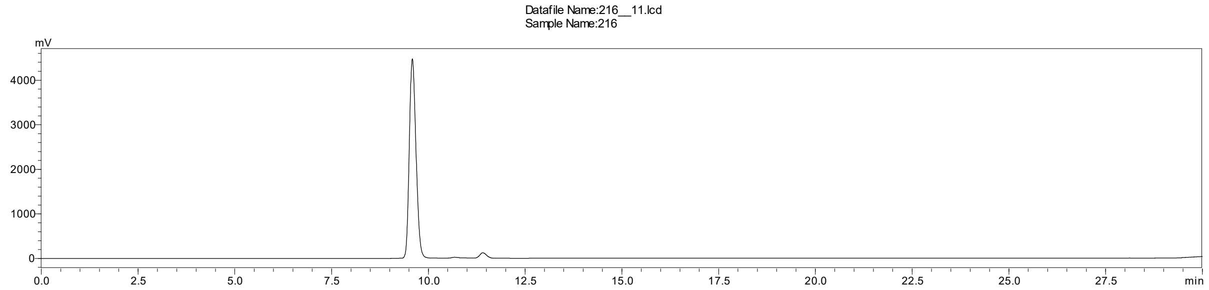

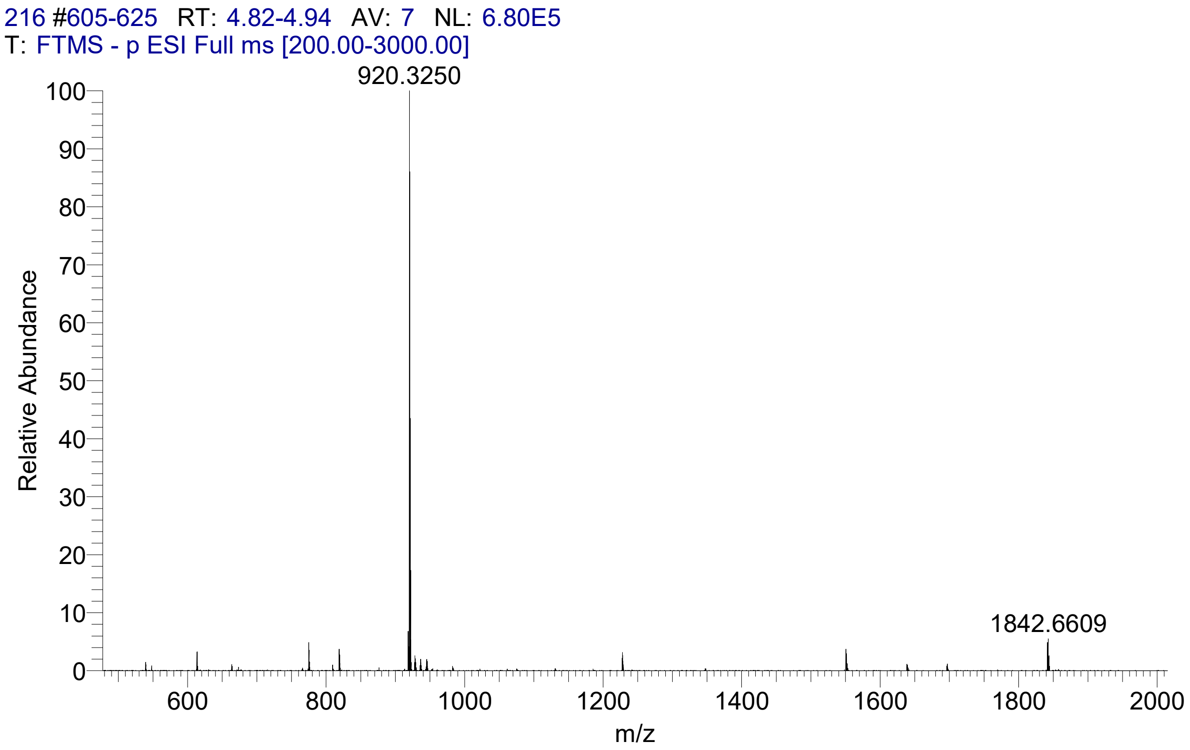

Neu5Acα2-6Galβ1-3[Galβ1-4(Fucα1-3)GlcNAcβ1-6]GalNAcα-Ser-Fmoc (58)

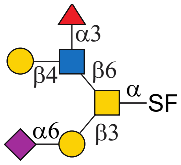

Compound **58** was prepared according to general procedure of α1-3 fucosylation with Hp3FT. After lyophilization, **58** was obtained as white solid. Compound was characterized by HPLC, T_R =10.587 min. ^{1}H NMR (600 MHz, D_2O) δ 7.95 - 7.87 (m, 2H), 7.73 (dd, J = 14.5, 7.3 Hz, 2H), 7.55 - 7.38 (m, 4H), 5.10 (d, J = 3.9 Hz, 1H), 4.69 (t, J = 8.7 Hz, 1H), 4.54 (d, J = 8.5 Hz, 1H), 4.41 (s, 1H), 4.38 (d, J = 7.4 Hz, 1H), 4.35 (m, 1H), 4.25 (d, J = 7.5 Hz, 2H), 4.20 (m, 1H), 4.06 (m, 1H), 4.03 – 3.45 (m, 35 H), 2.71 (dd, J = 12.5, 4.6 Hz, 1H), 2.05 (s, 3H), 2.00 (s, 3H), 1.94 (s, 3H), 1.70 (t, J = 12.2 Hz, 1H) , 1.18 (d, J = 6.5 Hz, 3H). HRMS, $C_{63}H_{90}N_4O_{37}$, Calcd for: 1494.5284; found $[M-H]^-$ 1493.5374, $[M-2H]^{2-}$ 746.2651.

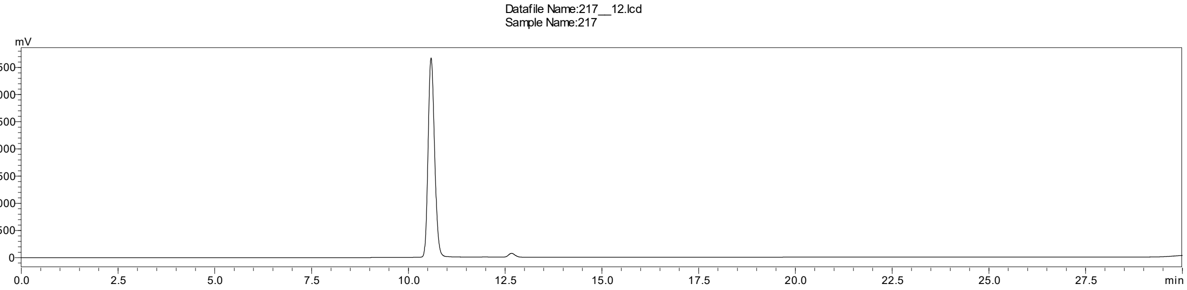

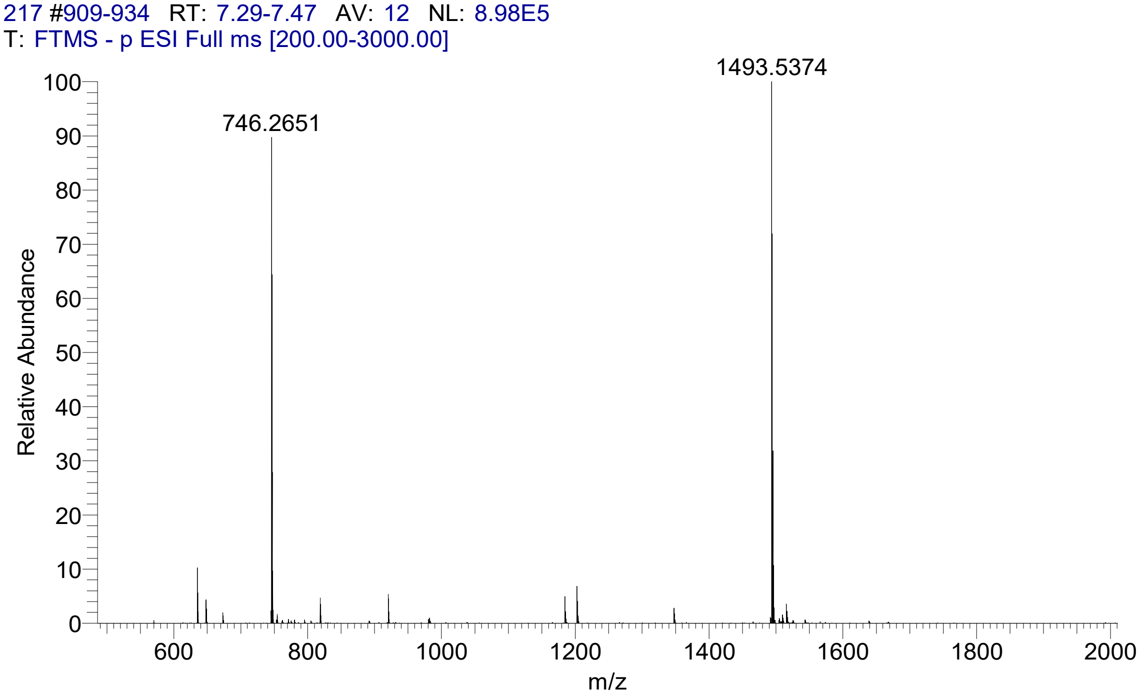

Neu5Acα2-6Galβ1-3[Fucα1-2Galβ1-4(Fucα1-3)GlcNAcβ1-6]GalNAcα-Ser-Fmoc (59)

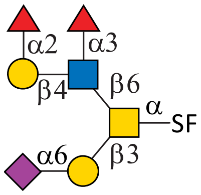

Compound **59** was prepared according to general procedure of α1-2 fucosylation with Hm2FT. After lyophilization, **59** was obtained as white solid. Compound was characterized by HPLC, T_R =10.034 min. ^{1}H NMR (600 MHz, D_2O) δ 7.93 - 7.80 (m, 2H), 7.76 – 7.57 (dd, *J* = 14.6, 7.6 Hz, 2H), 7.51 - 7.33 (m, 4H), 5.20 (t, *J* = 3.4 Hz, 1H), 5.16 (d, *J* = 4.0 Hz, 1H), 5.03 (s, 1H), 4.68 – 4.60 (m, 1H), 4.60 – 4.45 (m, 2H), 4.45- 4.24 (m, 4H), 4.22 – 3.99 (m, 4H), 3.99 – 3.31 (m, 36H), 2.69 (dd, *J* = 12.3, 4.6 Hz, 1H), 2.04 – 1.84 (m, 9H), 1.60 (t, *J* = 12.0 Hz, 1H), 1.18 (dd, *J* = 12.4, 6.3 Hz, 3H), 1.08 (d, *J* = 6.6 Hz, 3H). HRMS, $C_{69}H_{100}N_4O_{41}$, Calcd for: 1640.5863; found $[M-2H]^{2-}$ 819.2965.

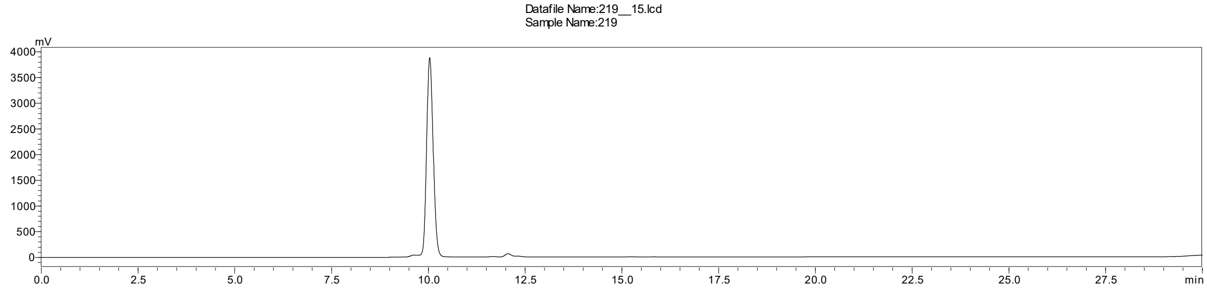

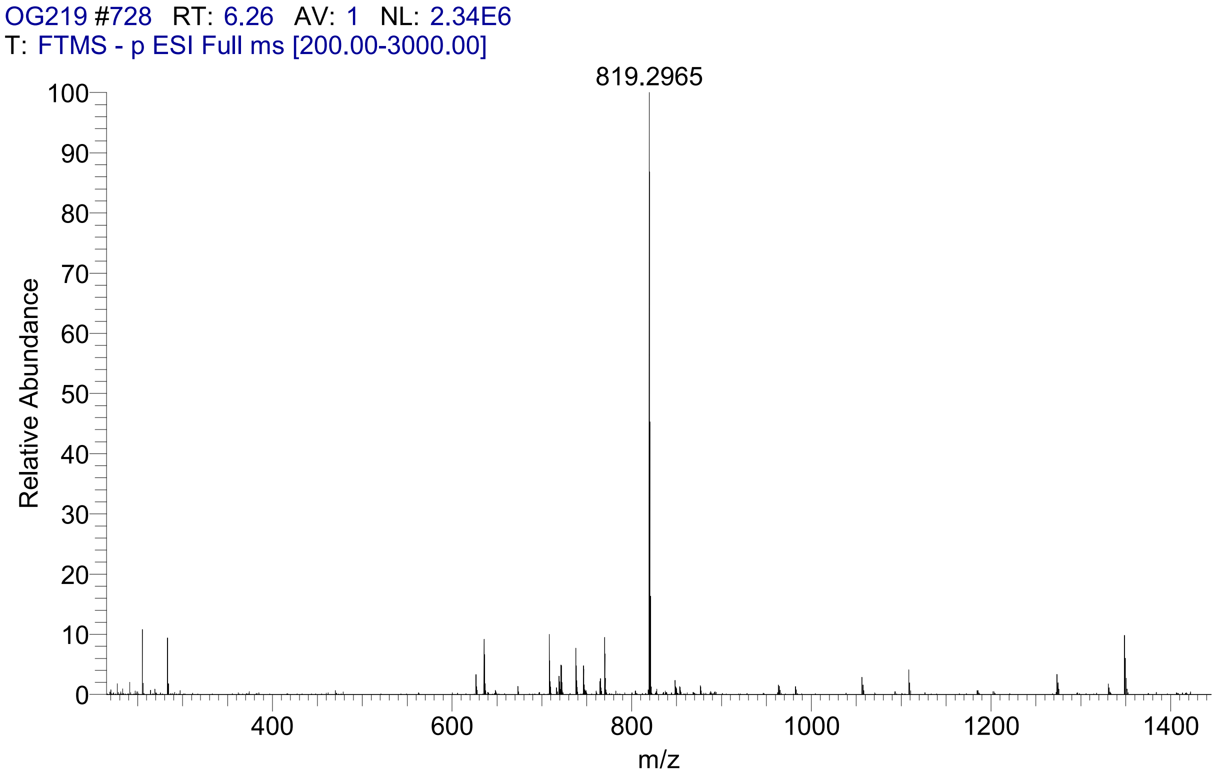

Galβ1-3(Galβ1-4GlcNAcβ1-6)GalNAcα-Ser-Fmoc (60)

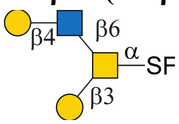

Compound **60** was prepared according to general procedure of β1-4 galactosylation with NmLgtB. After lyophilization, **60** was obtained as white solid. Compound was characterized by HPLC, T_R =14.073 min. ^{1}H NMR (600 MHz, D_2O) δ 7.95 - 7.84 (m, 2H), 7.77 - 7.61 (m, 2H), 7.54 - 7.3 (m, 4H), 4.68 -4.56 (m, 2H), 4.51 (d, J = 8.1 Hz, 1H), 4.37 (m, 4H), 4.24 (m, 1H), 4.15 (s, 1H), 4.02 - 3.44 (m, 24H), 1.96 (s, 3H), 1.94 (s, 3H). HRMS, $C_{46}H_{63}N_3O_{25}$, Calcd for: 1057.3751; found $[M-H]^-$ 1056.3804.

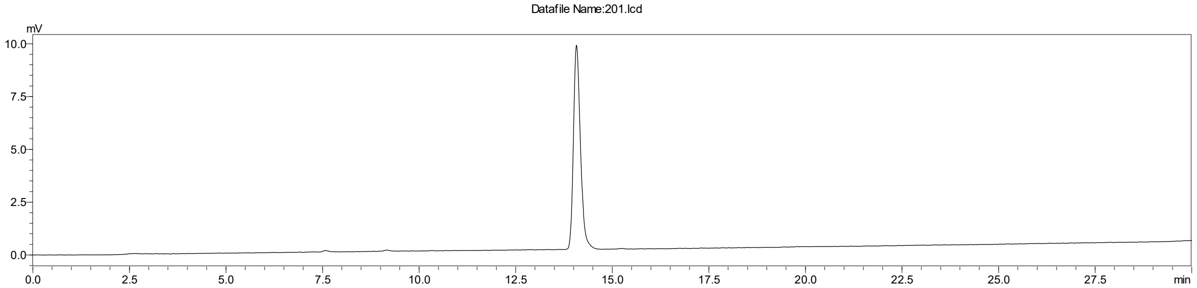

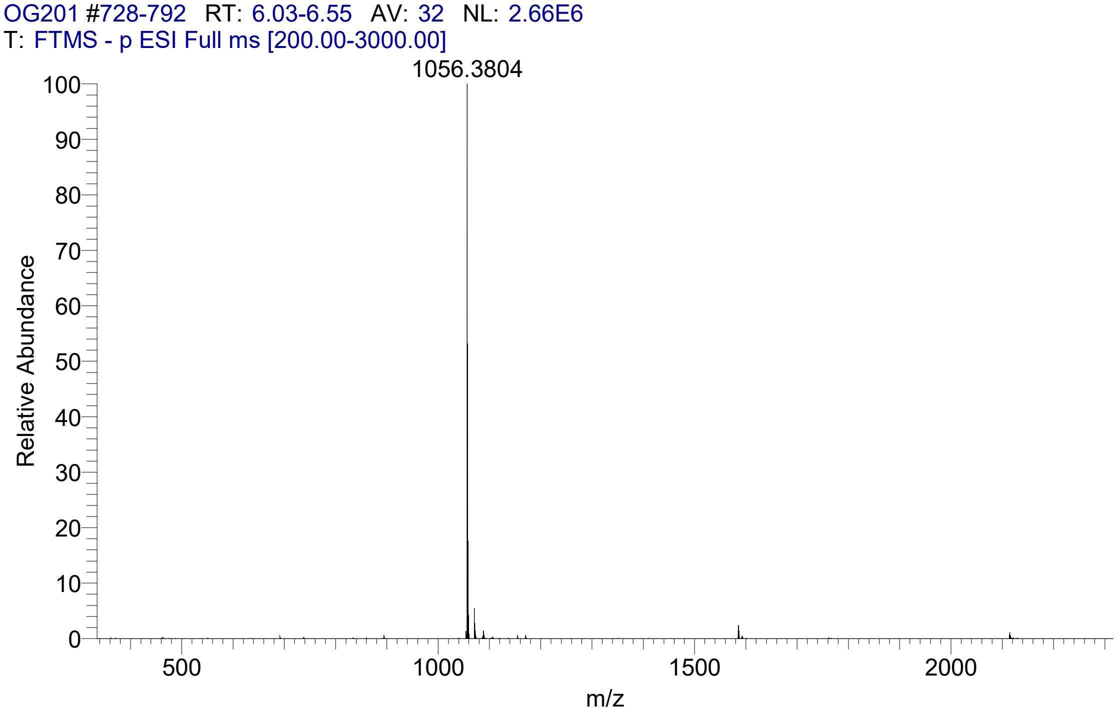

Galβ1-3(Galα1-3Galβ1-4GlcNAcβ1-6)GalNAcα-Ser-Fmoc (61)

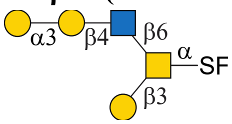

Compound **61** was prepared according to general procedure of α1-3 galactosylation with α3GalT. After lyophilization, **61** was obtained as white solid. Compound was characterized by HPLC, T_R =13.271 min. ^{1}H NMR (600 MHz, D_2O) δ 7.91 – 7.80 (m, 2H), 7.75 – 7.57 (m, 2H), 7.52 - 7.34 (m, 4H), 5.14 (d, *J* = 3.9 Hz, 1H), 4.64 – 4.54 (m, 2H), 4.50 (d, *J* = 8.0 Hz, 1H), 4.44 (d, *J* = 7.8 Hz, 1H), 4.39 - 4.32 (m, 2H), 4.28 (s, 1H), 4.25 – 4.12 (m, 4H), 4.02 (d, *J* = 3.3 Hz, 1H), 3.96 (d, *J* = 3.3 Hz, 1H), 3.94 (d, *J* = 3.3 Hz, 1H), 3.93 - 3.89 (m, 2H), 3.88 (d, *J* = 3.8 Hz, 1H), 3.86 (d, *J* = 3.8 Hz, 1H), 3.84 – 3.63 (m, 21H), 1.95 (s, 3H), 1.94 (s, 3H). HRMS, $C_{52}H_{73}N_3O_{30}$, Calcd for: 1219.4279; found $[M-H]^-$ 1218.4334, $[M-2H]^{2-}$ 631.7163.

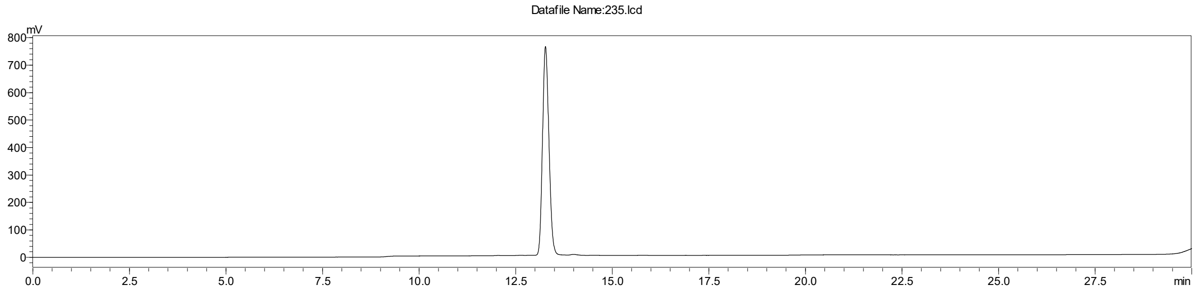

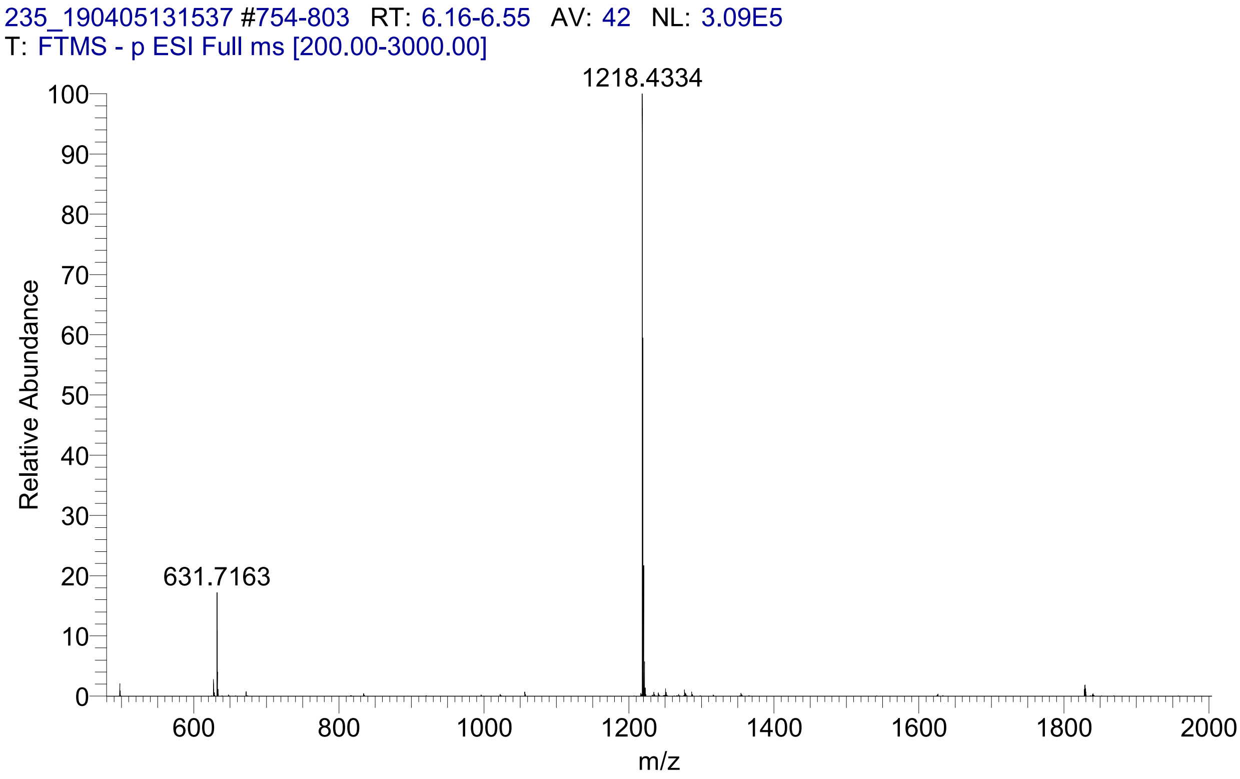

Galβ1-3[Galβ1-4(Fucα1-3)GlcNAcβ1-6]GalNAcα-Ser-Fmoc (62)

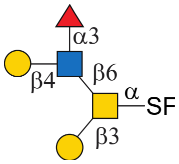

Compound **62** was prepared according to general procedure of α1-3 fucosylation with Hp3FT. After lyophilization, **62** was obtained as white solid. Compound was characterized by HPLC, T_R =12.594 min. ^{1}H NMR (600 MHz, D_2O) δ 7.96 - 7.85 (m, 2H), 7.78 – 7.67 (m, 2H), 7.55 - 7.39 (m, 4H), 5.10 (d, *J* = 3.1 Hz, 1H), 4.62 (d, *J* = 5.6 Hz, 1H), 4.52 (d, *J* = 8.1 Hz, 1H), 4.47 – 4.29 (m, 4H), 4.24 (m, 1H), 4.15 (s, 1H), 4.04 – 3.15 (m, 29 H), 1.97 (s, 3H), 1.94 (s, 3H), 1.17 (d, *J* = 6.5 Hz, 3H). HRMS, $C_{52}H_{73}N_3O_{29}$, Calcd for: 1203.433; found $[M-H]^-$ 1202.4382.

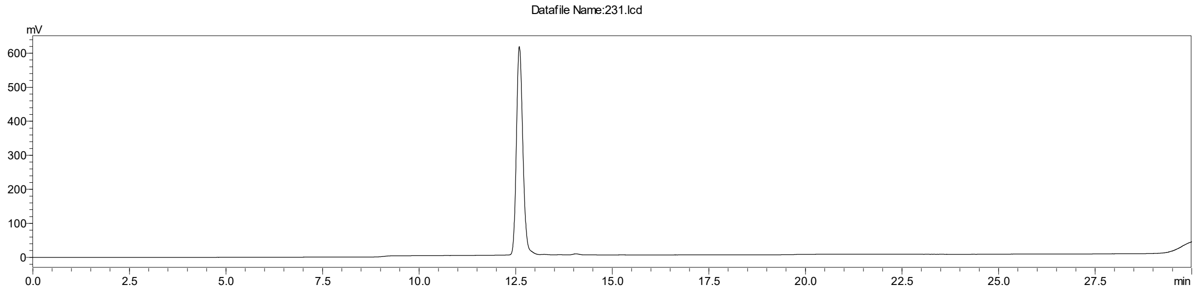

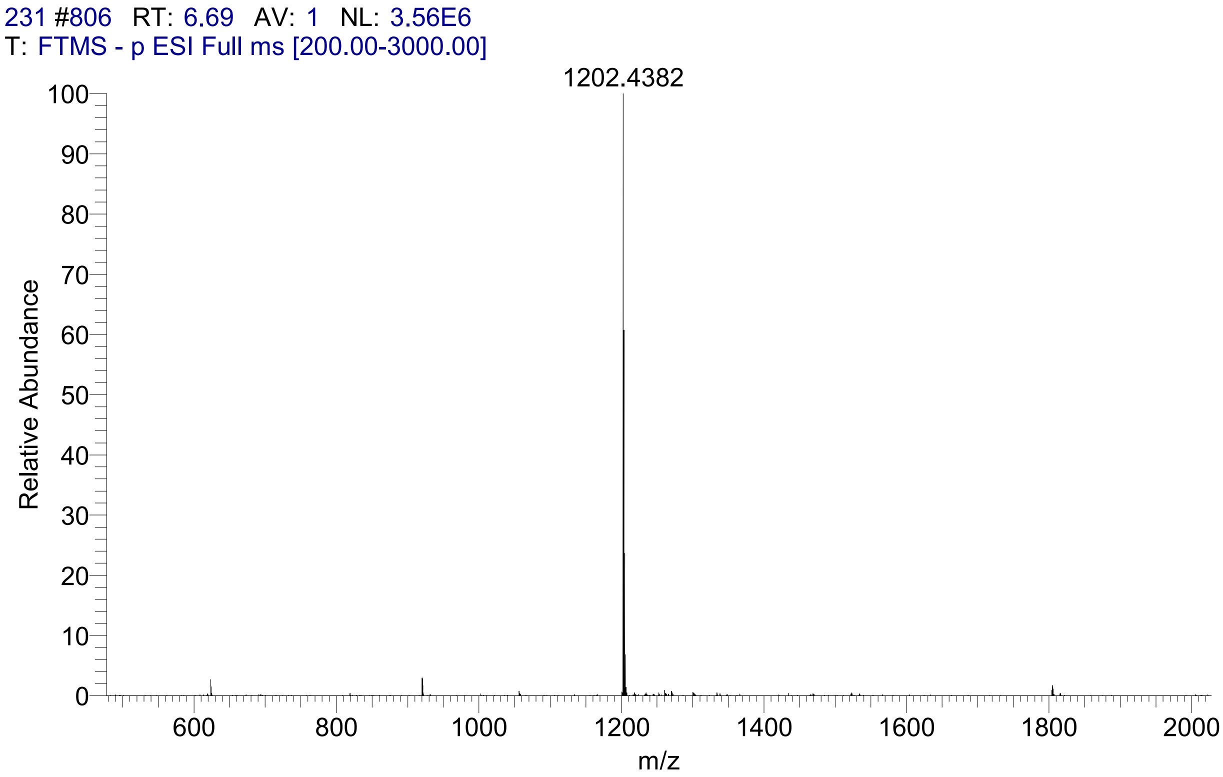

Fucα1-2Galβ1-3(GlcNAcβ1-6)GalNAcα-Ser-Fmoc (63)

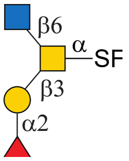

Compound **63** was prepared according to general procedure of α1-2 fucosylation with Hm2FT. After lyophilization, **63** was obtained as white solid. Compound was characterized by HPLC, T_R =13.589 min. ^{1}H NMR (600 MHz, D_2O) δ 7.85 (d, *J* = 7.6 Hz, 1H), 7.81 (d, *J* = 7.4 Hz, 1H), 7.81 (d, *J* = 7.6 Hz, 1H), 7.81 (d, *J* = 7.3 Hz, 1H), 7.52 - 7.35 (m, 4H), 5.18 (d, *J* = 4.2 Hz, 1H), 4.67 – 4.57 (m, 2H), 4.49 (d, *J* = 8.4 Hz, 1H), 4.45 (d, *J* = 7.6 Hz, 1H), 4.25 (s, 1H), 4.21 (s, 1H), 4.16 – 4.09 (m, 1H), 4.09 – 4.02 (m, 2H), 3.99 – 3.87 (m, 4H), 3.83 (d, *J* = 12.1 Hz, 1H), 3.80 – 3.33 (m, 16 H), 1.96 (s, 3H), 1.95 (s, 3H), 1.11 (d, *J* = 6.5 Hz, 3H). HRMS, $C_{46}H_{63}N_3O_{24}$, Calcd for: 1041.3801; found $[M-H]^-$ 1040.3837.

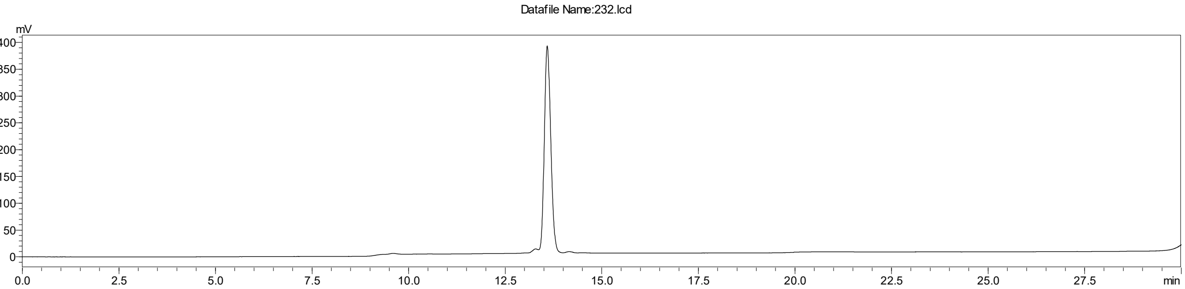

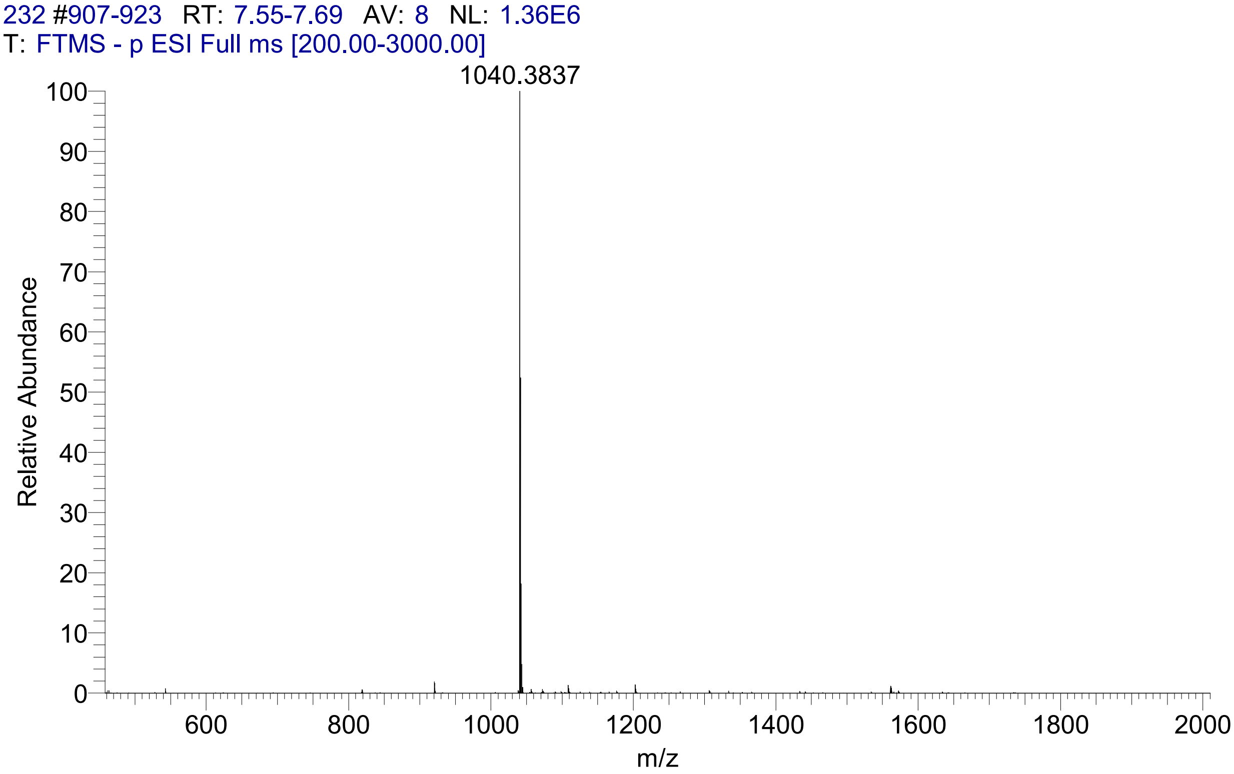

Fucα1-2Galβ1-3(Galβ1-4GlcNAcβ1-6)GalNAcα-Ser-Fmoc (64)

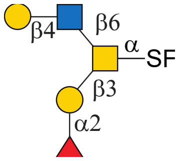

Compound **64** was prepared according to general procedure of β1-4 galactosylation with NmLgtB. After lyophilization, **64** was obtained as white solid. Compound was characterized by HPLC, T_R =12.010 min. ^{1}H NMR (600 MHz, D_2O) δ 7.90 (d, *J* = 6.9 Hz, 1H), 7.86 (d, *J* = 7.3 Hz, 1H), 7.74 (d, *J* = 7.5 Hz, 1H), 7.71 (d, *J* = 7.2 Hz, 1H), 7.52 - 7.35 (m, 4H), 4.60 – 4.53 (m, 2H), 4.51 – 4.46 (m, 1H), 4.42 (d, *J* = 7.7 Hz, 1H), 4.28 (t, *J* = 8.3 Hz, 2H), 4.13 (s, 1H), 4.05 (m, 1H), 4.03 – 3.97 (m, 2H), 3.96 – 3.77 (m, 8H), 3.76 – 3.39 (m, 20H), 1.88 (s, 3H), 1.87 (s, 3H), 1.05 (d, *J* = 6.9 Hz, 3H). HRMS, $C_{52}H_{73}N_3O_{29}$, Calcd for: 1203.433; found [M-H]$^-$ 1202.4388.

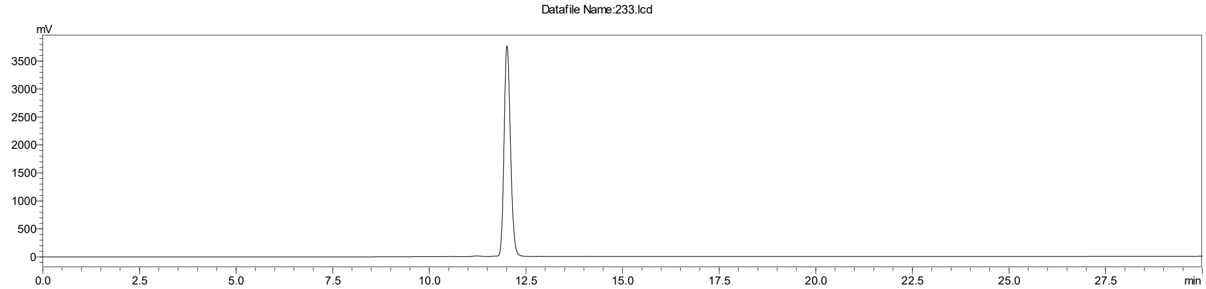

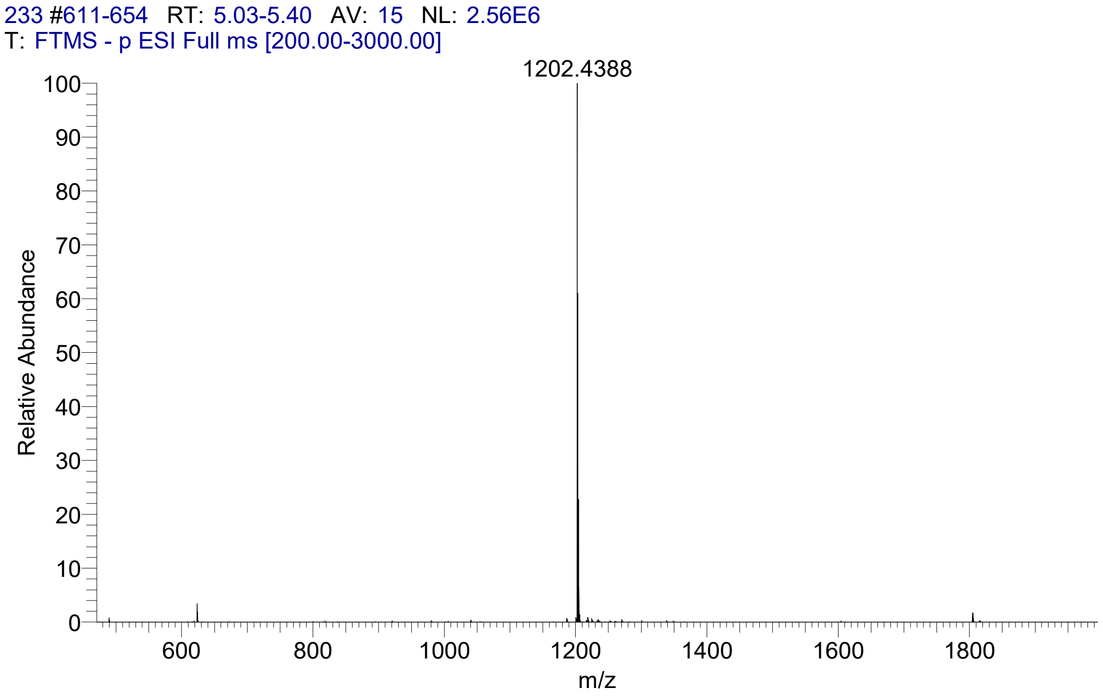

Fucα1-2Galβ1-3[Galβ1-4(Fucα1-3)GlcNAcβ1-6)GalNAcα-Ser-Fmoc (65)

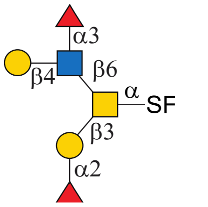

Compound **65** was prepared according to general procedure of α1-3 fucosylation with Hp3FT. After lyophilization, **65** was obtained as white solid. Compound was characterized by HPLC, T_R =10.555 min. ^{1}H NMR (600 MHz, D_2O) δ 7.80 (d, J = 7.5 Hz, 1H), 7.74 (d, J = 7.3 Hz, 1H), 7.66 (d, J = 7.4 Hz, 1H), 7.71 (d, J = 6.9 Hz, 1H), 7.48 - 7.30 (m, 4H), 5.13 (d, J = 3.8 Hz, 1H), 5.05 (d, J = 3.9 Hz, 1H), 4.59 (m, 1H), 4.56 (d, J = 7.9 Hz, 1H), 4.48 (d, J = 8.3 Hz, 1H), 4.39 (d, J = 7.5 Hz, 1H), 4.33 (m, 1H), 4.21 (s, 1H), 4.56 (d, J = 6.9 Hz, 1H), 4.03 – 3.97 (m, 2H), 3.92 - 3.39 (m, 32 H), 1.93 (s, 3H), 1.90 (s, 3H), 1.14 (d, J = 6.5 Hz, 3H), 1.05 (d, J = 6.5 Hz, 3H). HRMS, $C_{58}H_{83}N_3O_{33}$, Calcd for: 1349.4909; found $[M-H]^-$ 1348.4974, $[M-2H]^{2-}$ 696.7485.

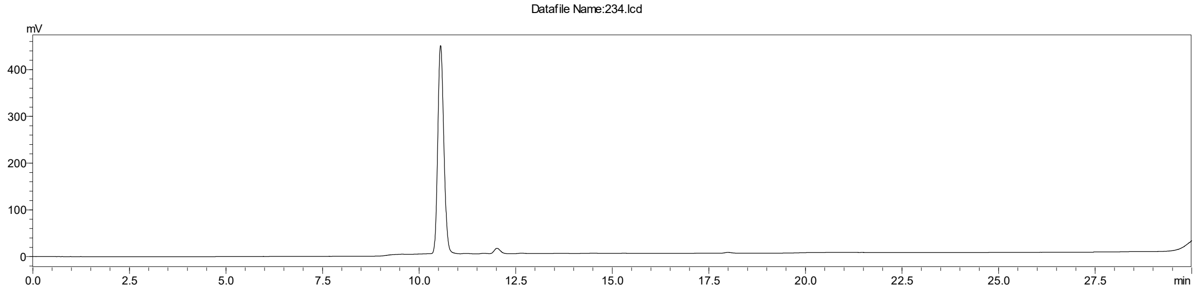

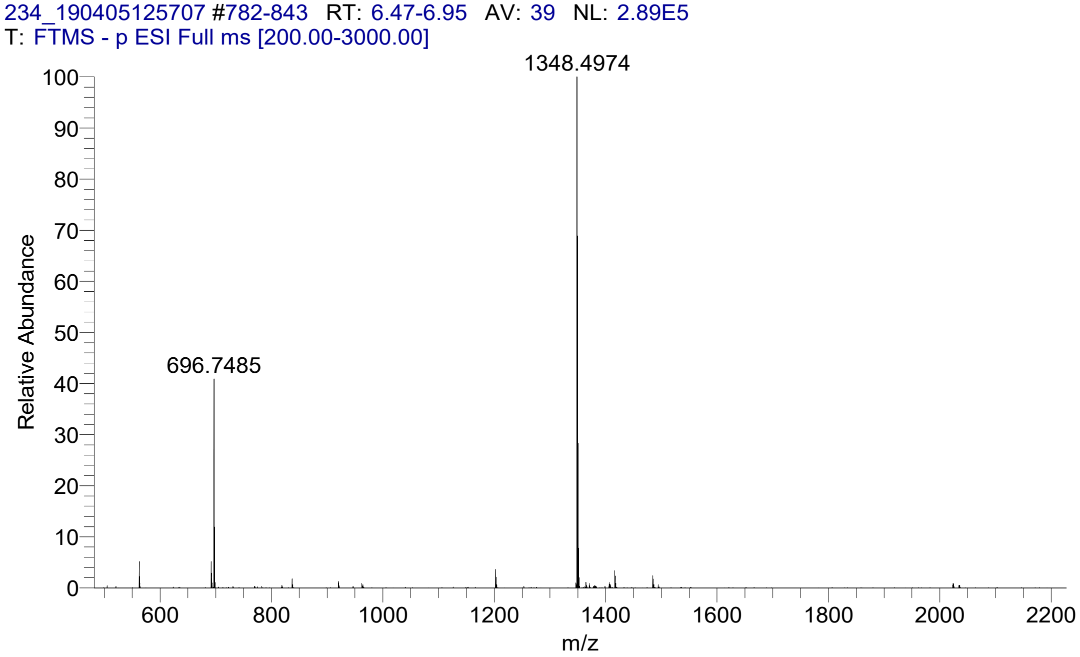

GlcNAcβ1-3(Neu5Acα2-6)GalNAcα-Ser-Fmoc (66)

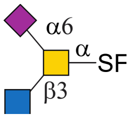

Compound **66** was prepared according to general procedure of α2-6 sialylation with Pd2,6ST. After lyophilization, **66** was obtained as white solid. Compound was characterized by HPLC, T_R =15.128 min. ^{1}H NMR (600 MHz, D_2O) δ 7.91 – 7.75 (m, 2H), 7.73 – 7.51 (m, 2H), 7.51 - 7.31 (m, 4H), 4.59 (d, *J* = 5.9 Hz, 2H), 4.45 (d, *J* = 8.3 Hz, 1H), 4.32 (s, 1H), 4.24 (s, 1H), 4.16 – 4.07 (m, 2H), 3.96 – 3.12 (m, 19 H), 2.63 (dd, *J* = 12.4, 4.5 Hz, 1H), 2.03 (s, 3H), 1.98 (s, 3H), 1.92 (s, 3H), 1.67 (t, *J* = 12.6 Hz, 1H). HRMS, $C_{45}H_{60}N_4O_{23}$, Calcd for: 1024.3648; found $[M-H]^-$ 1023.323.

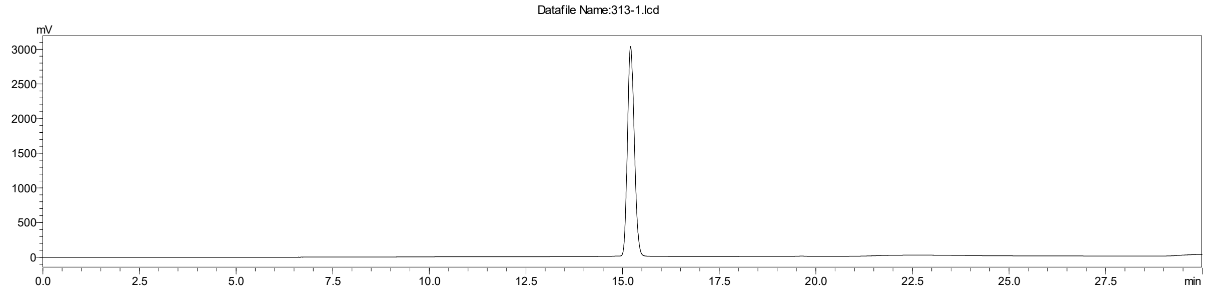

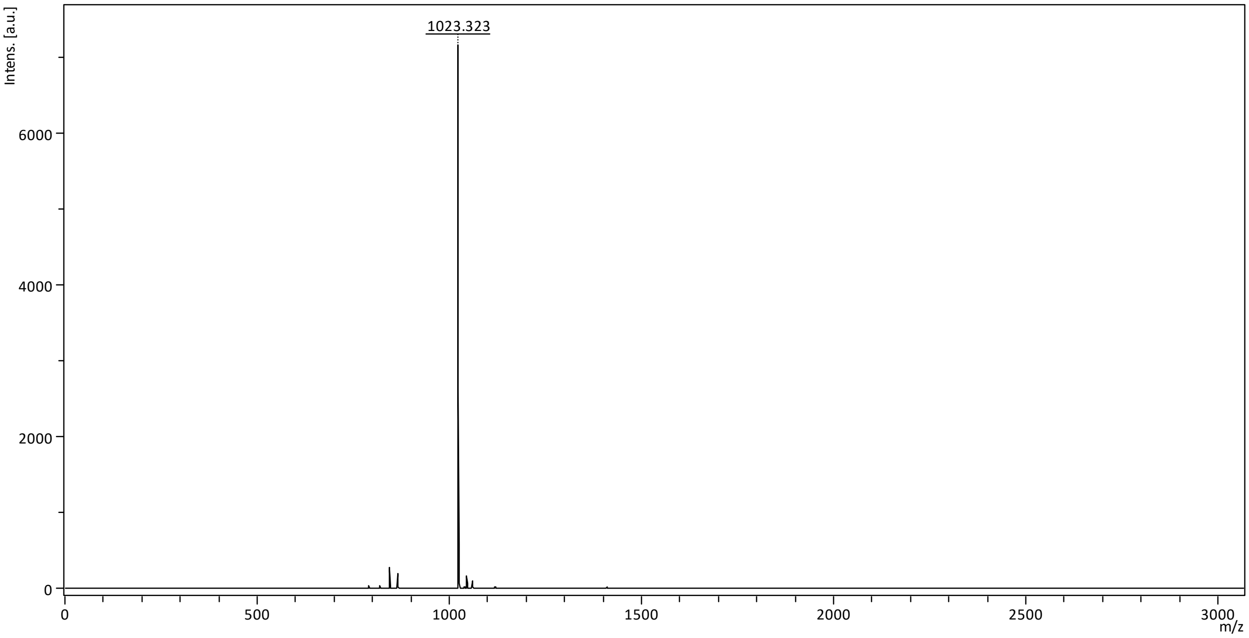

GlcNAcβ1-3(Neu5Acα2-6)GalNAcα-Ser-Fmoc (67)

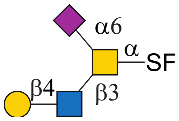

Compound **67** was prepared according to general procedure of β1-4 galactosylation with NmLgtB. After lyophilization, **67** was obtained as white solid. Compound was characterized by HPLC, T_R =13.449 min.
^{1}H NMR (600 MHz, D_2O) δ 7.76 – 7.72 (m, 2H), 7.67 – 7.52 (m, 2H), 7.47 - 7.26 (m, 4H), 4.71 (d, *J* = 3.7 Hz, 1H), 4.62 – 4.50 (m, 2H), 4.46 (d, *J* = 7.5 Hz, 2H), 4.33 (s, 1H), 4.23 - 4.07 (m, 3H), 3.93 – 3.34 (m, 24 H), 2.58 (dd, *J* = 12.4, 4.5 Hz, 1H), 2.01 (s, 3H), 1.96 (s, 3H), 1.91 (s, 3H), 1.69 (t, *J* = 12.3 Hz, 1H). HRMS, $C_{51}H_{70}N_4O_{28}$, Calcd for: 1186.4177; found $[M-H]^-$ 1185.485.

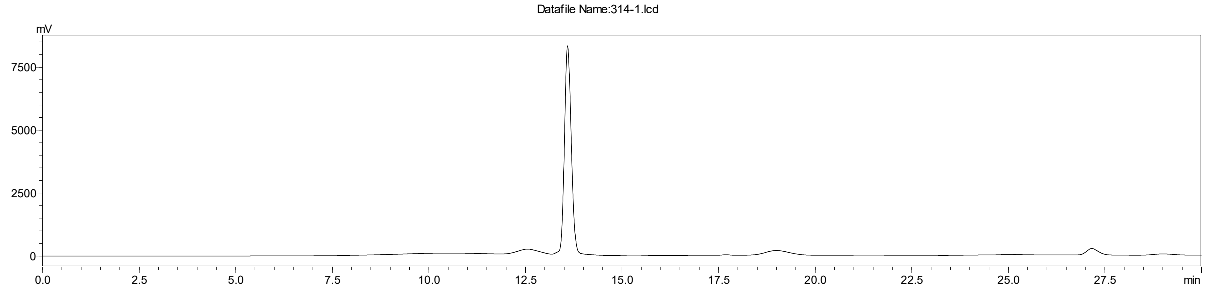

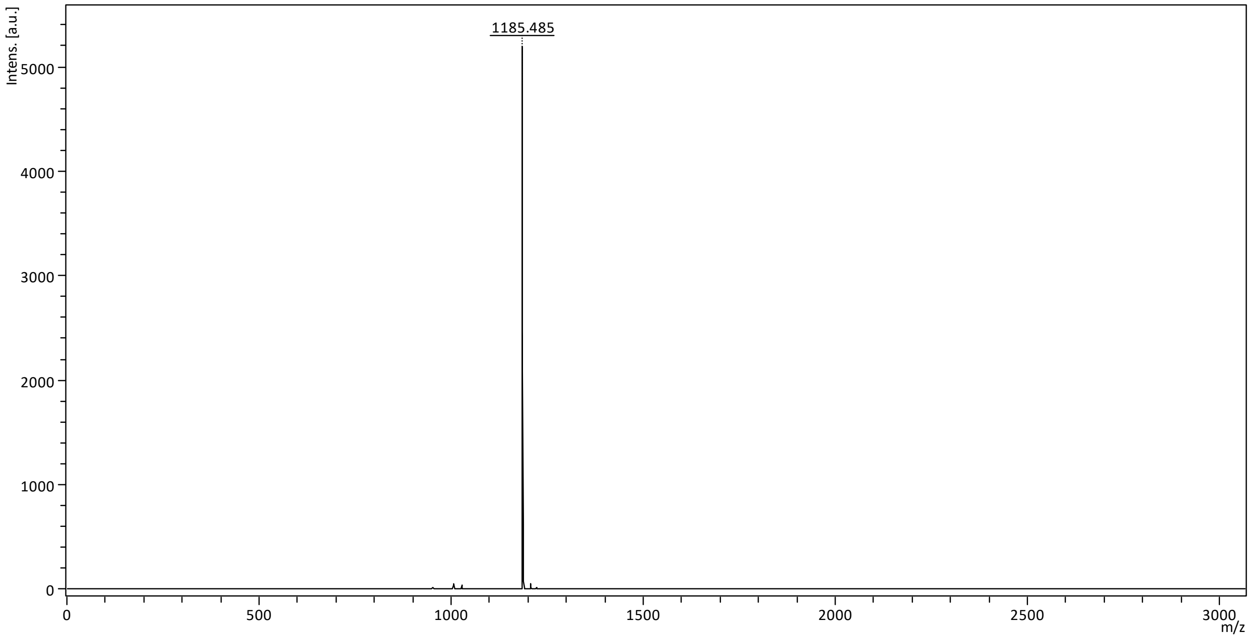

Galβ1-4GlcNAcβ1-3GalNAcα-Ser-Fmoc (68)

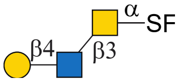

Compound **68** was prepared according to general procedure of β1-4 galactosylation with NmLgtB. After lyophilization, **68** was obtained as white solid. Compound was characterized by HPLC, T_R =17.724 min. ^{1}H NMR (600 MHz, D_2O) δ 7.93 – 7.75 (m, 2H), 7.73 – 7.56 (m, 2H), 7.55 - 7.32 (m, 4H), 4.72 (s, 1H), 4.60 (m, 2H), 4.48 (d, *J* = 7.9 Hz, 2H), 4.29 – 4.09(m, 4H), 3.95 - 3.59 (m, 15H), 3.59 – 3.42(m, 2H), 1.98 (s, 3H), 1.93 (s, 3H). HRMS, $C_{40}H_{53}N_3O_{20}$, Calcd for: 895.3222; found $[M+Na]^+$ 918.750, $[M+K]^+$ 934.754.

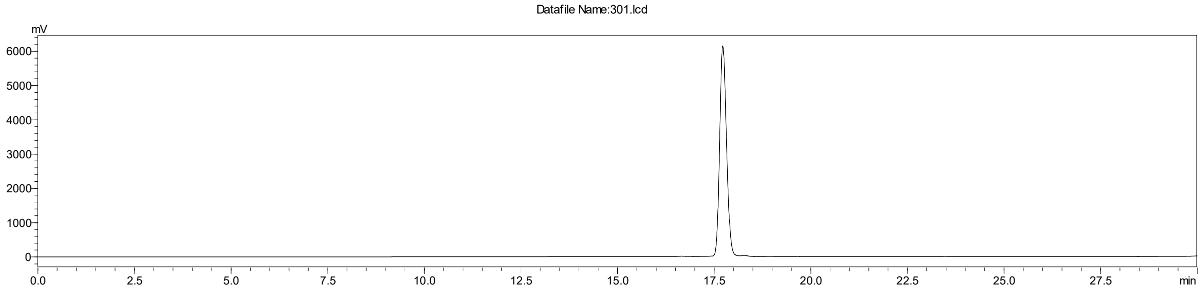

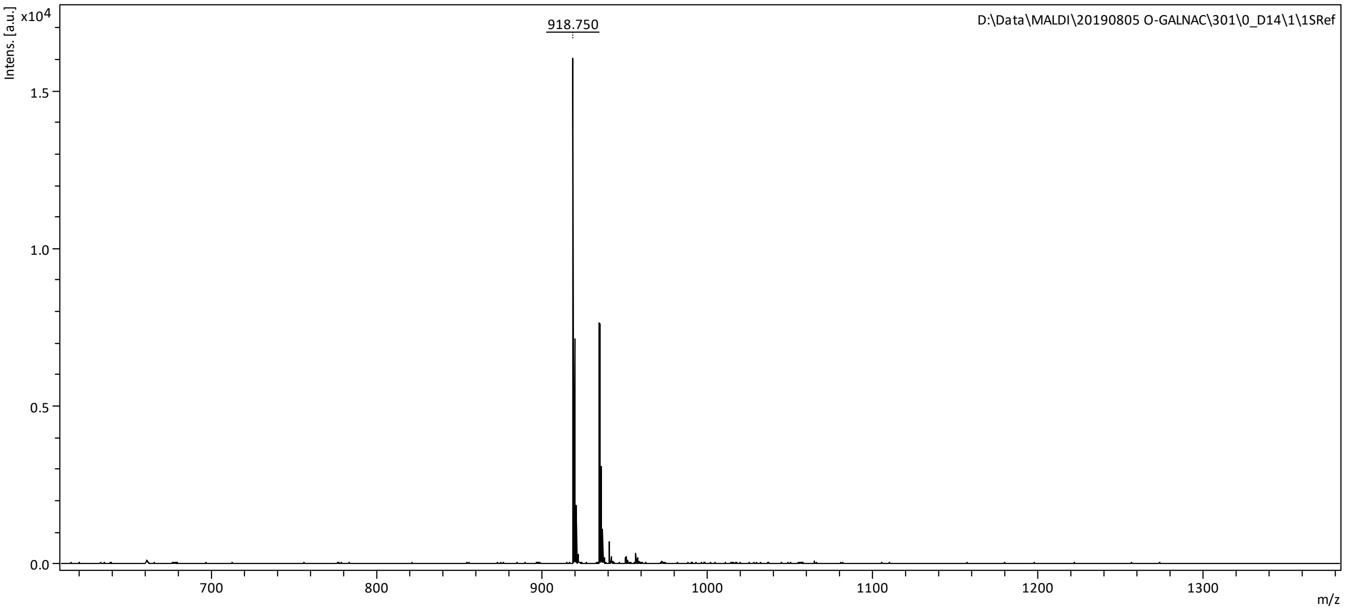

Neu5Acα2-3Galβ1-4GlcNAcβ1-3GalNAcα-Ser-Fmoc (69)

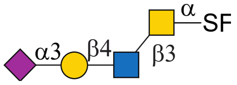

Compound **69** was prepared according to general procedure of α2-3 sialylation with PmST1-M144D. After lyophilization, **69** was obtained as white solid. Compound was characterized by HPLC, T_R =15.915 min.
^{1}H NMR (600 MHz, D_2O) δ 7.97 – 7.85 (m, 2H), 7.77 – 7.61 (m, 2H), 7.56 - 7.37 (m, 4H), 4.70 – 4.62 (m, 1H), 4.60 – 4.44 (m, 2H), 4.33 (s, 2H), 4.21 – 4.10 (m, 3H), 4.00 – 3.41 (m, 25H), 2.78 (dd, *J* = 12.2, 4.5 Hz, 1H), 2.04 (s, 3H), 2.00 (s, 3H), 1.94 (s, 3H), 1.86 (t, *J* = 12.3 Hz, 1H). HRMS, $C_{51}H_{70}N_4O_{28}$, Calcd for: 1186.4177; found $[M-H]^-$ 1185.120, $[M+Na-2H]^-$ 1207.092.

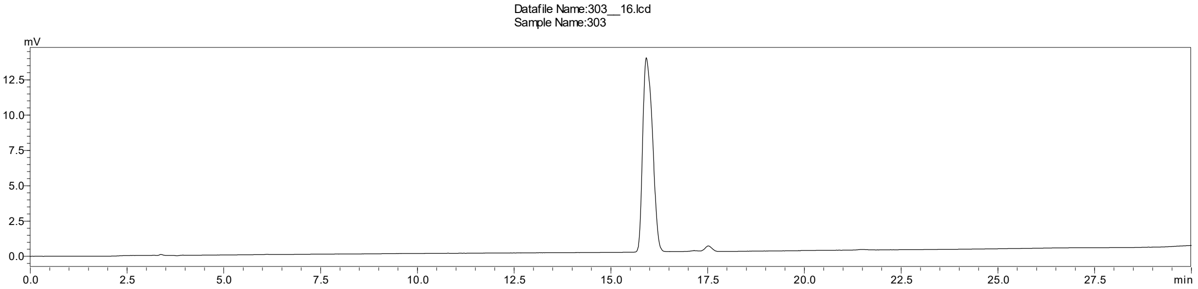

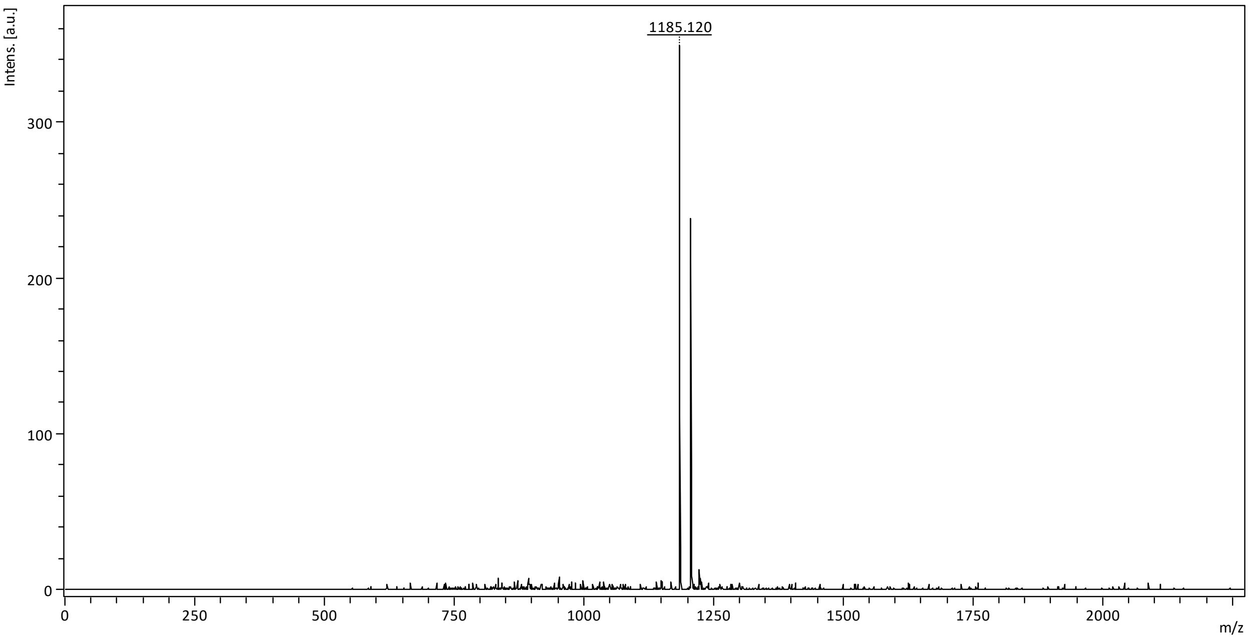

Neu5Acα2-3(GalNAcβ1-4)Galβ1-4GlcNAcβ1-3GalNAcα-Ser-Fmoc (70)

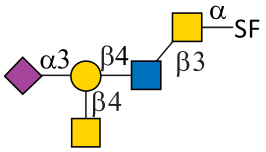

Compound **70** was prepared according to general procedure of β1-4-*N*-acetylgalatosaminylation with CgtA. After lyophilization, **70** was obtained as white solid. Compound was characterized by HPLC, T_R =14.682 min. ^{1}H NMR (600 MHz, D_2O) δ 7.94 – 7.81 (m, 2H), 7.73 – 7.56 (m, 2H), 7.53 - 7.33 (m, 4H), 4.75 -4.67 (m, 3H), 4.56 (d, *J* = 7.9 Hz, 1H), 4.43 (m, 1H), 4.34 – 4.23 (m, 2H), 4.19 – 4.06 (m, 4H), 3.97 – 3.33 (m, 30 H), 2.68 (dd, *J* = 12.5, 3.8 Hz, 1H), 2.02 (s, 3H), 2.00 (s, 3H), 1.97 (s, 3H), 1.92 (s, 3H), 1.75 (t, *J* = 12.3Hz, 1H). HRMS, $C_{59}H_{83}N_5O_{33}$, Calcd for: 1389.497; HRMS, found $[M-H]^-$ 1388.471.

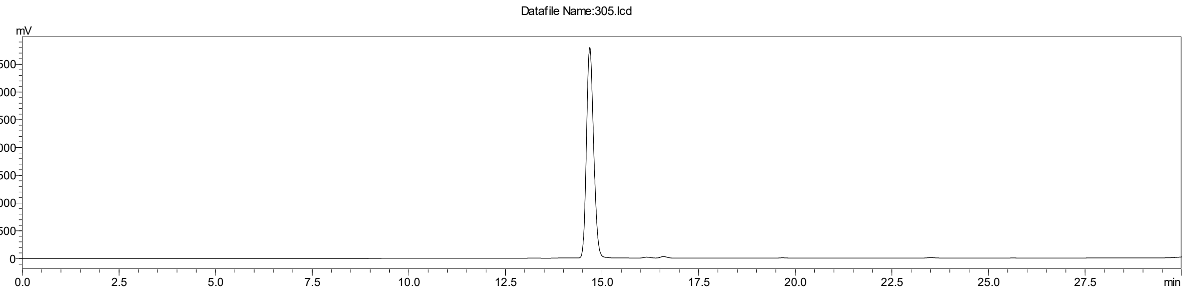

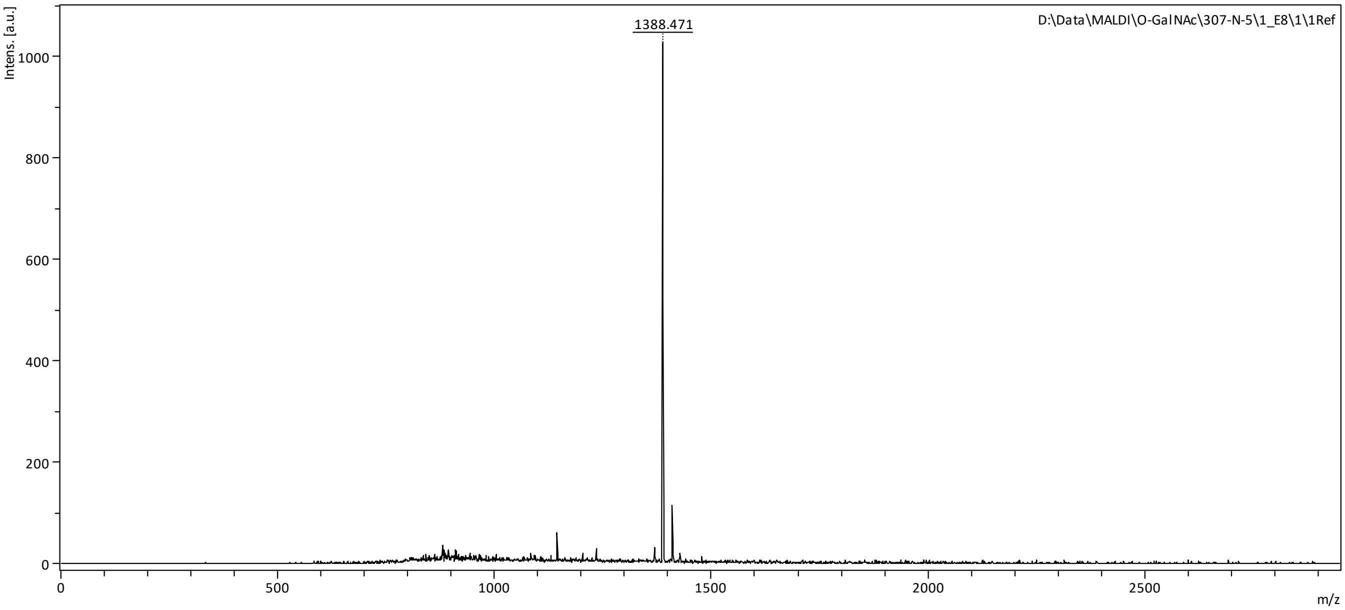

Neu5Acα2-6Galβ1-4GlcNAcβ1-3GalNAcα-Ser-Fmoc (71)

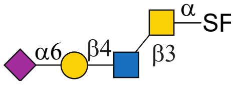

Compound **71** was prepared according to general procedure of α2-6 sialylation with PmST1-P34H/M144L. After lyophilization, **71** was obtained as white solid. Compound was characterized by HPLC, T_R =16.081 min. ^{1}H NMR (600 MHz, D_2O) δ 7.98 – 7.85 (m, 2H), 7.79 – 7.62 (m, 2H), 7.56 - 7.35 (m, 4H), 4.67 (s, 1H), 4.56 – 4.40 (m, 3H), 4.33 (s, 1H), 4.24 – 4.09 (m, 3H), 4.03 – 3.45 (m, 24H), 2.66 (dd, *J* = 11.4, 3.5 Hz, 1H), 2.02 (s, 3H), 2.01 (s, 3H), 1.94 (s, 3H), 1.70 (t, *J* = 12.1 Hz, 1H). HRMS, $C_{51}H_{70}N_4O_{28}$, Calcd for: 1186.4177; found $[M-H]^-$ 1185.320.

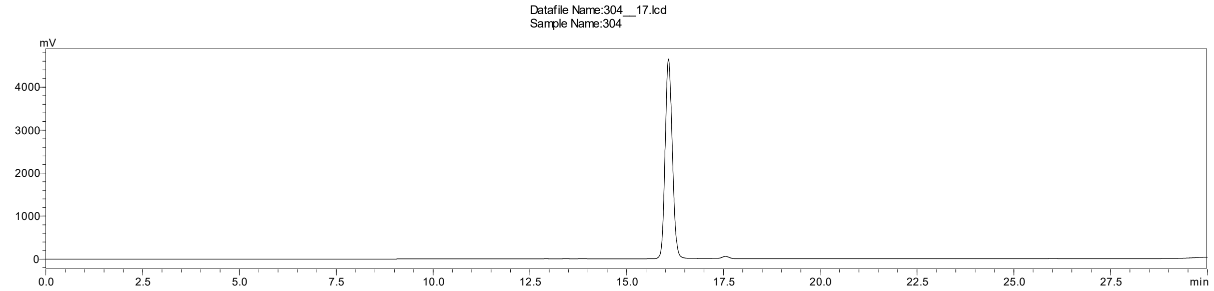

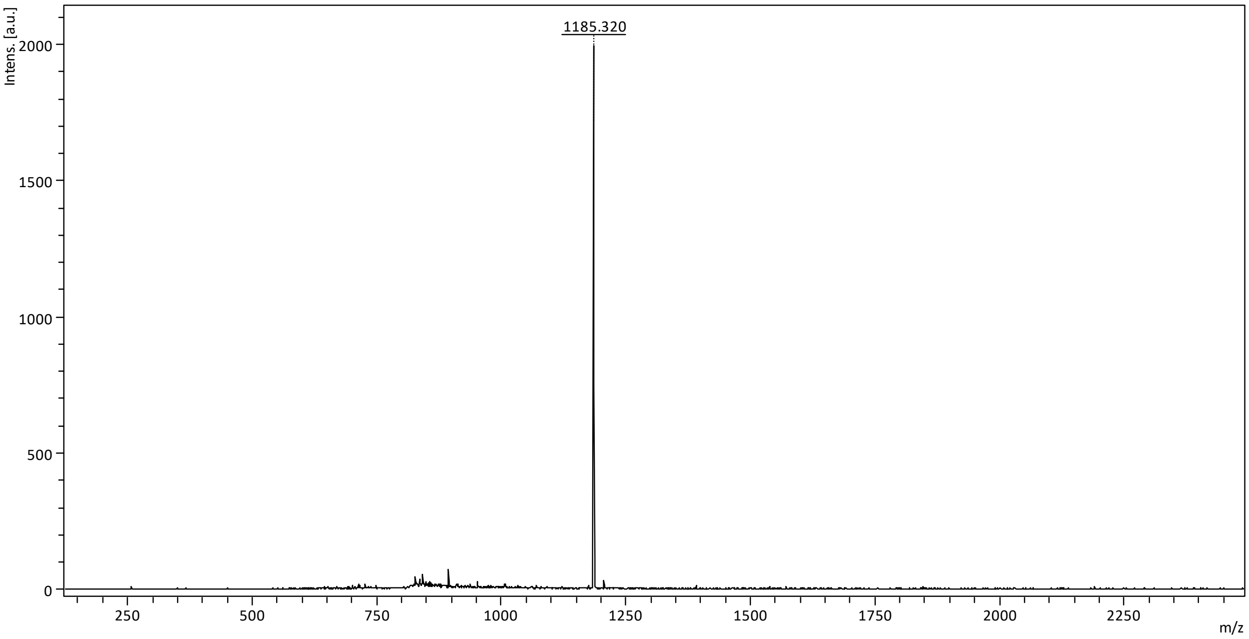

Fucα1-2Galβ1-4GlcNAcβ1-3GalNAcα-Ser-Fmoc (72)

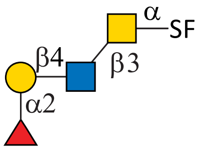

Compound **72** was prepared according to general procedure of α1-2 fucosylation with Hm2FT. After lyophilization, **72** was obtained as white solid. Compound was characterized by HPLC, T_R =16.637 min. ^{1}H NMR (600 MHz, D_2O) δ 7.83 – 7.70 (m, 2H), 7.66 – 7.45 (m, 2H), 7.44 - 7.25 (m, 4H), 5.27 (d, *J* = 3.5 Hz, 1H), 4.65 - 4.44 (m, 4H), 4.31 – 4.08 (m, 5H), 3.98 – 3.57 (m, 20 H), 3.46 – 3.32 (m, 1H), 1.98 (s, 3H), 1.92 (s, 3H), 1.18 (d, *J* = 7.4 Hz, 3H). HRMS, $C_{46}H_{63}N_3O_{24}$, Calcd for: 1041.3801; found $[M-H]^-$ 1040.348.

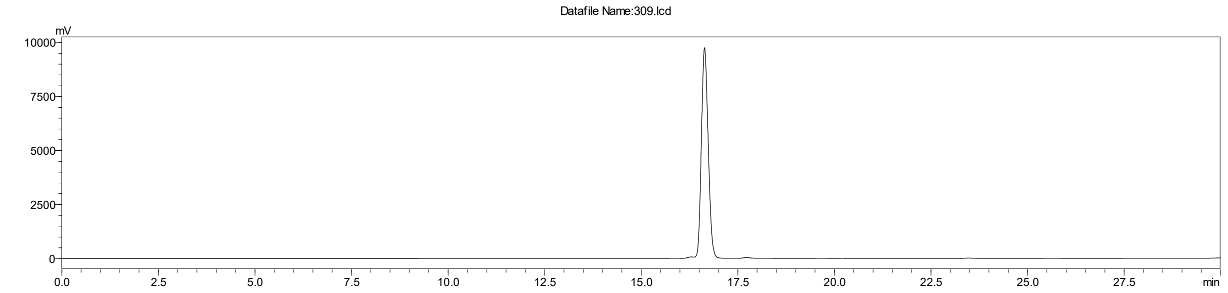

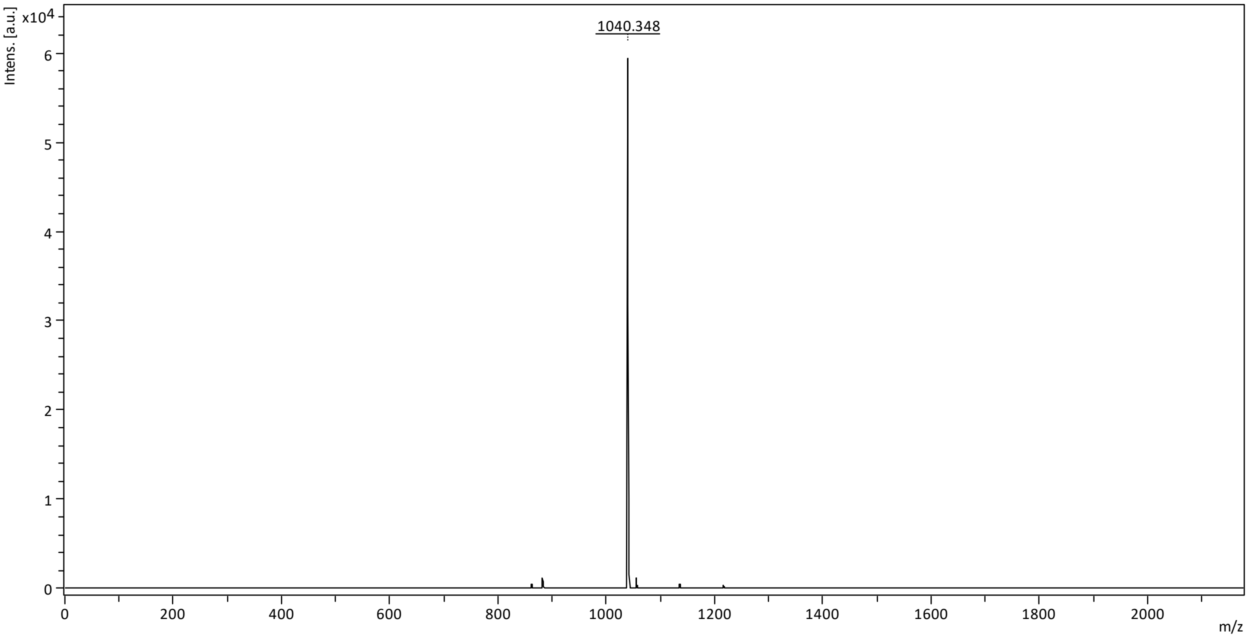

GalNAcα1-3(Fucα1-2)Galβ1-4GlcNAcβ1-3GalNAcα-Ser-Fmoc (73)

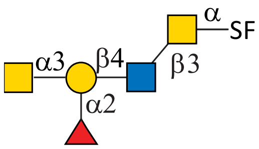

Compound **73** was prepared according to general procedure of α1-3-*N*-acetylgalatosaminylation with BgtA. After lyophilization, **73** was obtained as white solid. Compound was characterized by HPLC, T_R =16.033 min. ^{1}H NMR (600 MHz, D_2O) δ 7.95 – 7.82 (m, 2H), 7.75 – 7.58 (m, 2H), 7.55 - 7.33 (m, 4H), 5.34 (d, *J* = 3.6 Hz, 1H), 5.16 (d, *J* = 3.7 Hz, 1H), 4.68 - 4.50 (m, 4H), 4.36 – 4.08 (m, 8 H), 4.04 – 3.57 (m, 23 H), 3.39 (m, 1H), 2.03 (s, 3H), 1.98 (s, 3H), 1.93 (s, 3H), 1.23 (d, *J* = 6.6 Hz, 3H). HRMS, $C_{54}H_{76}N_4O_{29}$, Calcd for: 1244.4595; found $[M-H]^-$ 1243.428.

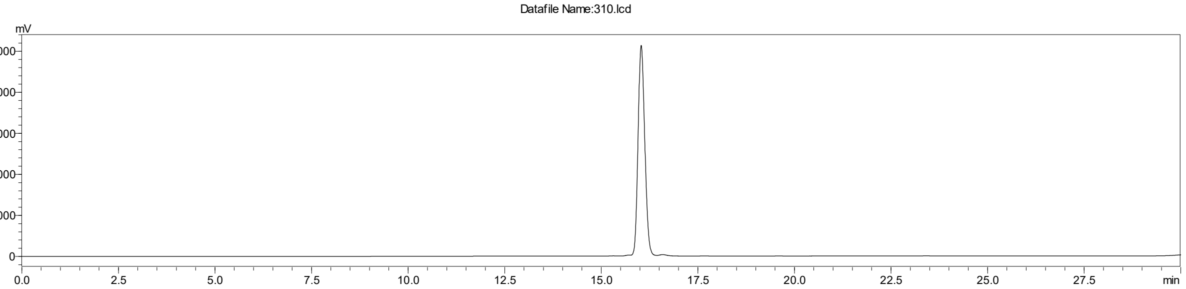

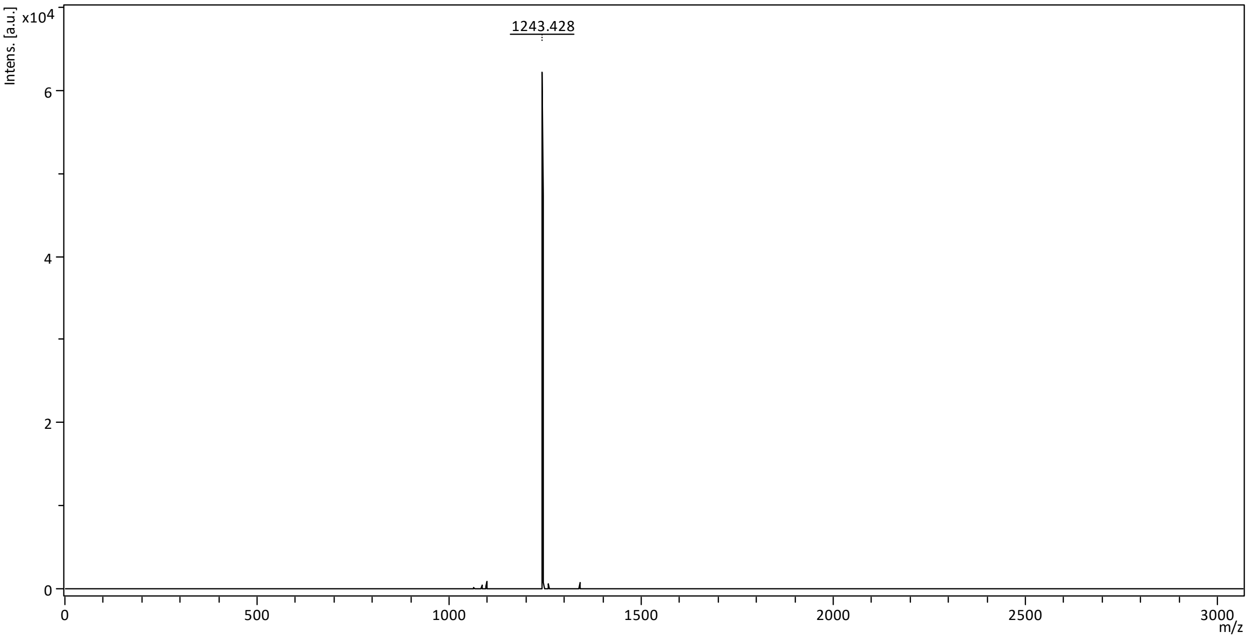

Galα1-3(Fucα1-2)Galβ1-4GlcNAcβ1-3GalNAcα-Ser-Fmoc (74)

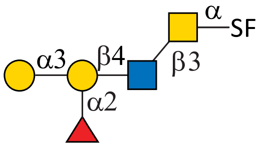

Compound **74** was prepared according to general procedure of α1-3 galactosylation with GTB. After lyophilization, **74** was obtained as white solid. Compound was characterized by HPLC, T_R =15.736 min. ^{1}H NMR (600 MHz, D_2O) δ 7.95 – 7.82 (m, 2H), 7.75 – 7.59 (m, 2H), 7.54 - 7.34 (m, 4H), 5.31 (s, 1H), 5.23 (s, 1H), 4.66 - 4.49 (m, 4H), 4.37 – 4.25 (m, 3 H), 4.24 – 4.10 (m, 3 H), 4.06 – 3.57 (m, 23H), 3.43 – 3.30 (m, 3H), 1.99 (s, 3H), 1.93 (s, 3H), 1.22 (d, J = 6.5 Hz, 3H). HRMS, $C_{52}H_{73}N_3O_{29}$, Calcd for: 1203.4330; found $[M-H]^-$ 1202.426.

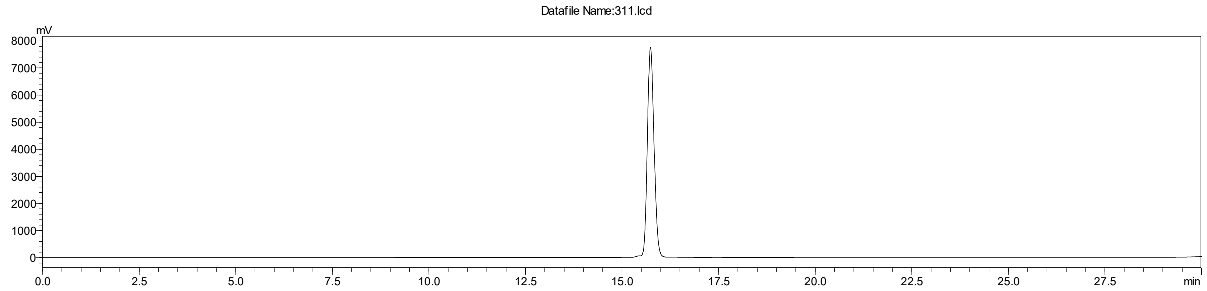

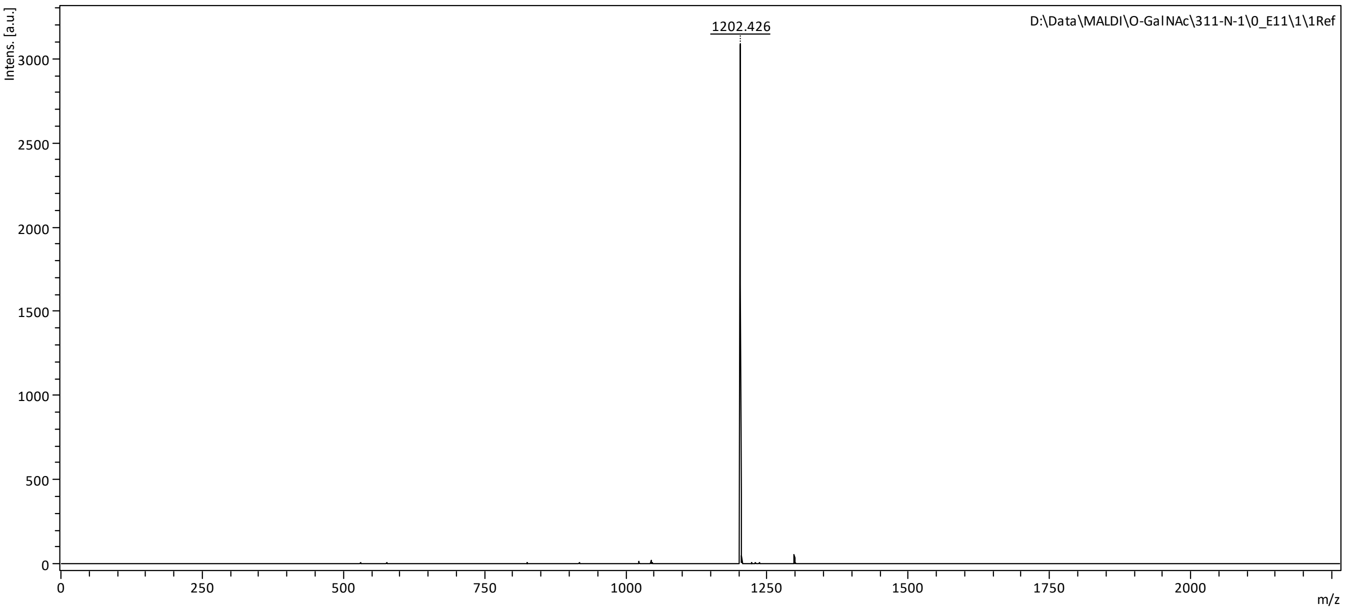

Fucα1-2Galβ1-4(Fucα1-3)GlcNAcβ1-3GalNAcα-Ser-Fmoc (75)

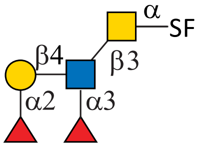

Compound **75** was prepared according to general procedure of α1-3 fucosylation with Hp3FT. After lyophilization, **75** was obtained as white solid. Compound was characterized by HPLC, T_R =15.661 min. ^{1}H NMR (600 MHz, D_2O) δ 7.92 – 7.78 (m, 2H), 7.73 – 7.55 (m, 2H), 7.52 - 7.31 (m, 4H), 5.24 (d, *J* = 3.5 Hz, 1H), 5.07 (d, *J* = 3.2 Hz, 1H), 4.73 (s, 1H), 4.65 - 4.53 (m, 2H), 4.50 (d, *J* = 13.8 Hz, 1H), 4.31 – 4.06 (m, 5 H), 4.00 – 3.51 (m, 23 H), 3.46 – 3.35 (m, 2H), 1.97 (s, 3H), 1.92 (s, 3H), 1.19 (d, *J* = 5.4 Hz, 6H). HRMS, $C_{52}H_{73}N_3O_{28}$, Calcd for: 1187.4381; found $[M-H]^-$ 1186.427.

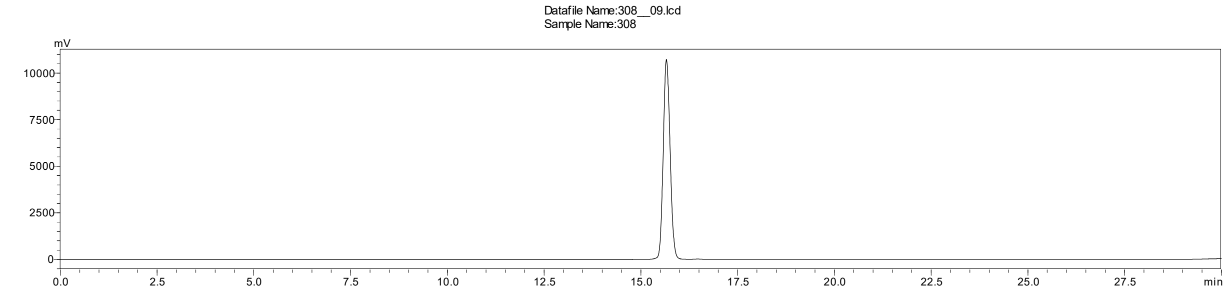

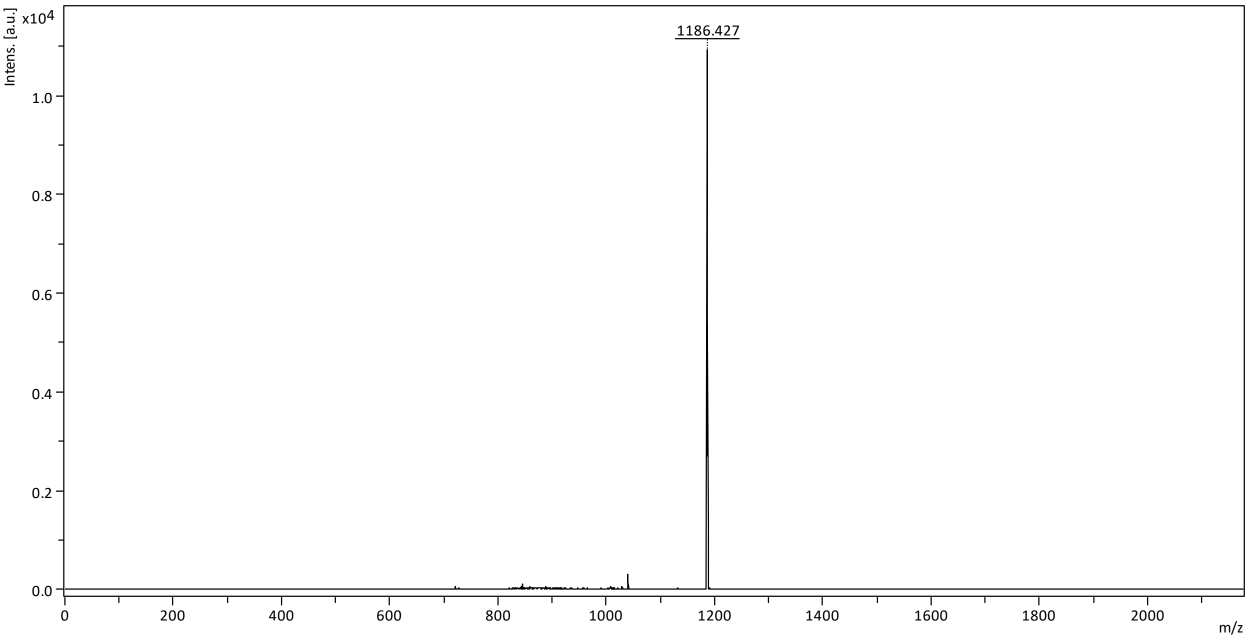

Galα1-3Galβ1-4GlcNAcβ1-3GalNAcα-Ser-Fmoc (76)

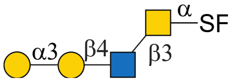

Compound **76** was prepared according to general procedure of α1-3 galactosylation with α3GalT. After lyophilization, **76** was obtained as white solid. Compound was characterized by HPLC, T_R =16.394 min. ^{1}H NMR (600 MHz, D_2O) δ 7.72 – 7.62 (m, 2H), 7.62 – 7.41 (m, 2H), 7.40 - 7.15 (m, 4H), 5.13 (d, *J* = 4.3 Hz, 1H), 4.70 (s, 1H), 4.58 – 4.36 (m, 4H), 4.23 (s, 1H), 4.21 – 4.03 (m, 4H), 3.99 (m, 2H), 3.96 – 3.55 (m, 20H), 1.97 (s, 3H), 1.92 (s, 3H). HRMS, $C_{46}H_{63}N_3O_{25}$, Calcd for: 1057.3751; found $[M+H]^+$ 1058.3836, $[M+Na]^+$ 1080.3667.

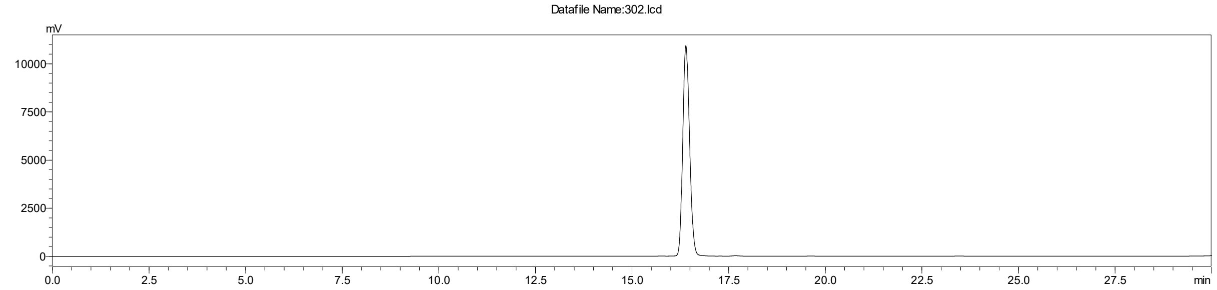

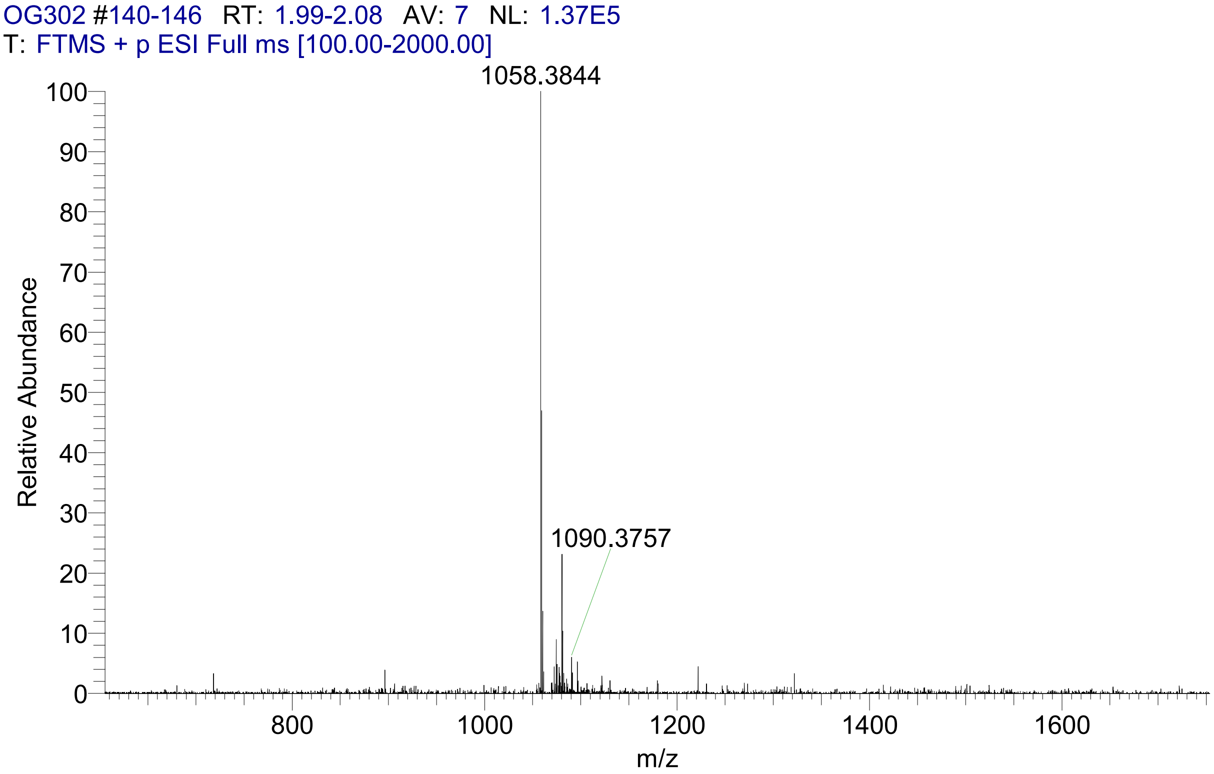

Galβ1-4(Fucα1-3)GlcNAcβ1-3GalNAcα-Ser-Fmoc (77)

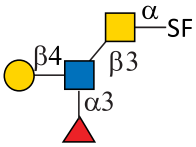

Compound **77** was prepared according to general procedure of α1-3 fucosylation with Hp3FT. After lyophilization, **77** was obtained as white solid. Compound was characterized by HPLC, T_R =16.782 min. ^{1}H NMR (600 MHz, D_2O) δ 7.99 – 7.86 (m, 2H), 7.80 – 7.63 (m, 2H), 7.56 - 7.36 (m, 4H), 5.09 (d, *J* = 3.6 Hz, 1H), 4.57 - 4.36 (m, 3H), 4.34 (s, 2H), 4.21 – 4.09 (m, 3H), 3.97 – 3.40 (m, 22 H), 1.97 (s, 3H), 1.94 (s, 3H), 1.19 (d, *J* = 6.5 Hz, 3H). HRMS, $C_{46}H_{63}N_3O_{24}$, Calcd for: 1041.3801; found $[M-H]^-$ 1040.392.

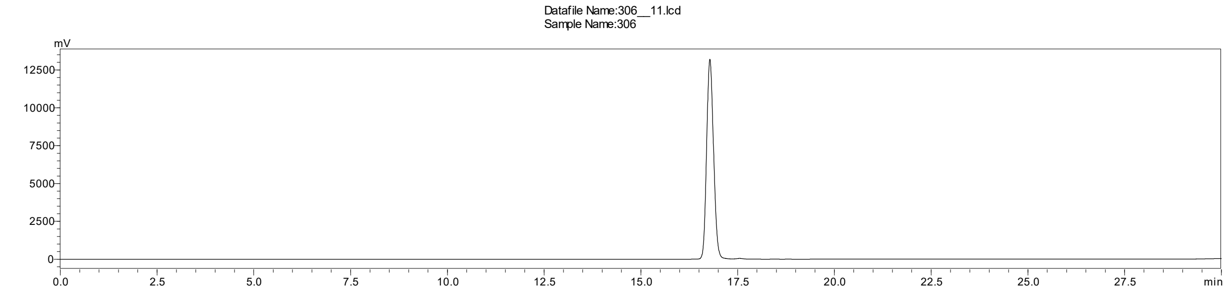

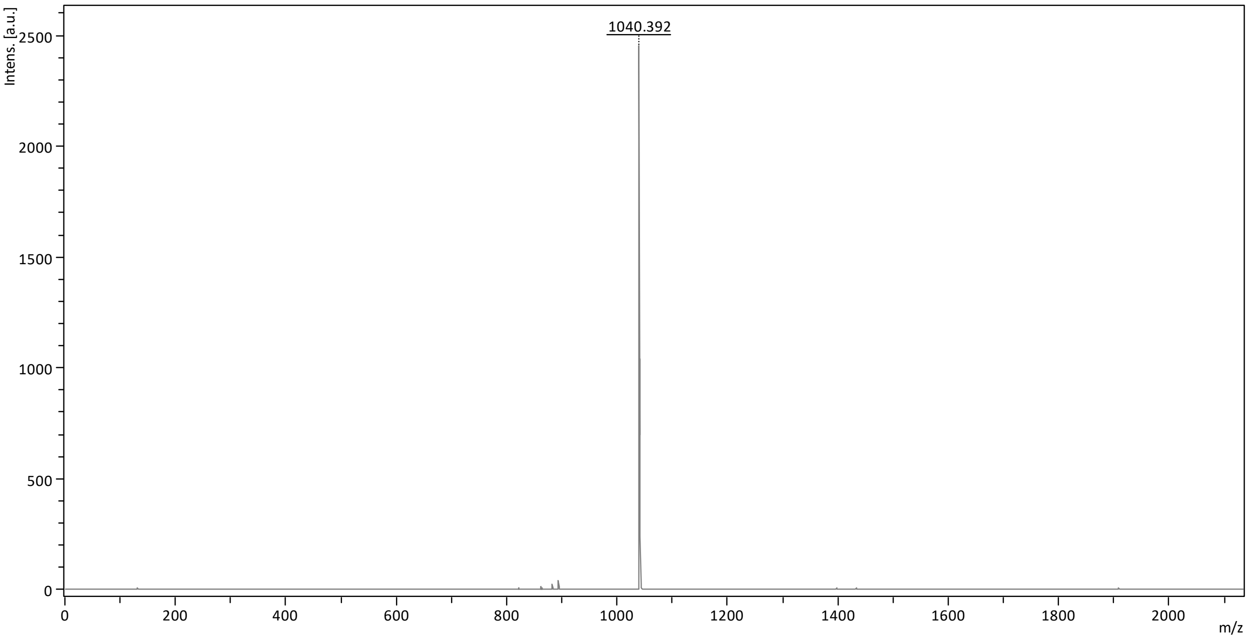

Neu5AcGalβ1-4(Fucα1-3)GlcNAcβ1-3GalNAcα-Ser-Fmoc (78)

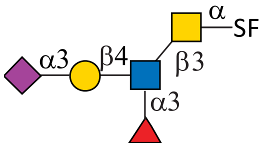

Compound **78** was prepared according to general procedure of α2-3 sialylation with PmST1-M144D. After lyophilization, **78** was obtained as white solid. Compound was characterized by HPLC, T_R =15.199 min. ^{1}H NMR (600 MHz, D_2O) δ 7.96 – 7.80 (m, 2H), 7.76 – 7.55 (m, 2H), 7.55 - 7.33 (m, 4H), 5.11 (d, J = 3.3 Hz, 1H), 4.73 - 4.60 (m, 2H), 4.60 – 4.48 (m, 2H), 4.31 (s, 1H), 4.27 (s, 1H), 4.21 – 4.09 (m, 3H), 4.03 – 3.41 (m, 28 H), 2.78 (dd, J = 12.7,4.5 Hz, 1H), 2.04 (s, 3H), 1.98 (s, 3H), 1.94 (s, 3H), 1.87 (d, J = 12.3 Hz, 1H), 1.19 (d, J = 6.6 Hz, 3H). HRMS, $C_{57}H_{80}N_4O_{32}$Calcd for: 1332.4756; found $[M-H]^-$ 1331.341.

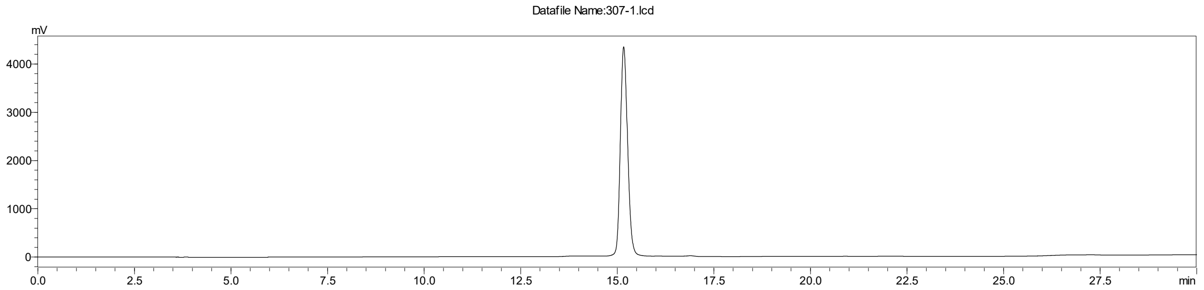

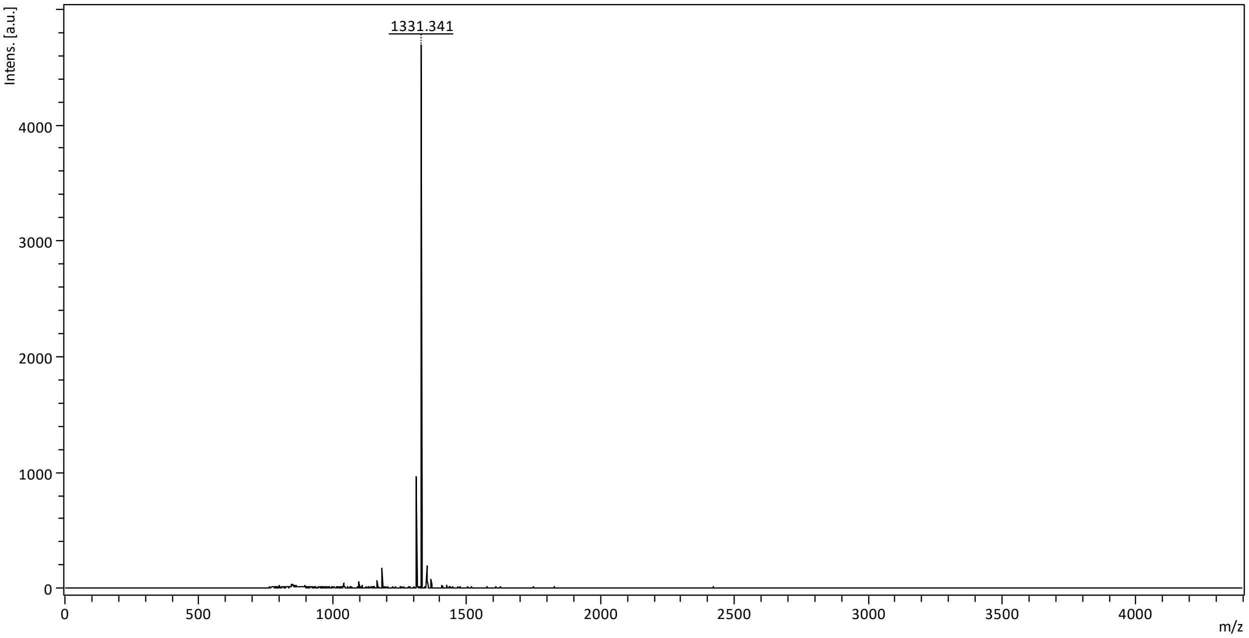

GalNAcβ1-4GlcNAcβ1-3GalNAcα-Ser-Fmoc (79)

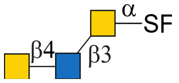

Compound **79** was prepared according to general procedure of β1-4-*N*-acetylgalatosaminylation with NmLgtBm. After lyophilization, **79** was obtained as white solid. Compound was characterized by HPLC, T_R =17.575 min. ^{1}H NMR (600 MHz, D_2O) δ 7.87 – 7.77 (m, 2H), 7.70 – 7.53 (m, 2H), 7.47 - 7.29 (m, 4H), 4.69 - 4.57 (m, 2H), 4.47 (d, J = 8.4 Hz, 1H), 4.34 (s, 1H), 4.27 (s, 1H), 4.18 (s, 1H), 4.14 – 4.01 (m, 2 H), 3.98 – 3.80 (m, 2H), 3.79 – 3.51 (m, 14), 3.38 – 3.26 (m, 2H), 2.03 (s, 3H), 1.91 (s, 3H), 1.86 (s, 3H). HRMS, $C_{42}H_{56}N_4O_{20}$, Calcd for: 936.3488; found $[M-H]^-$ 935.3528.

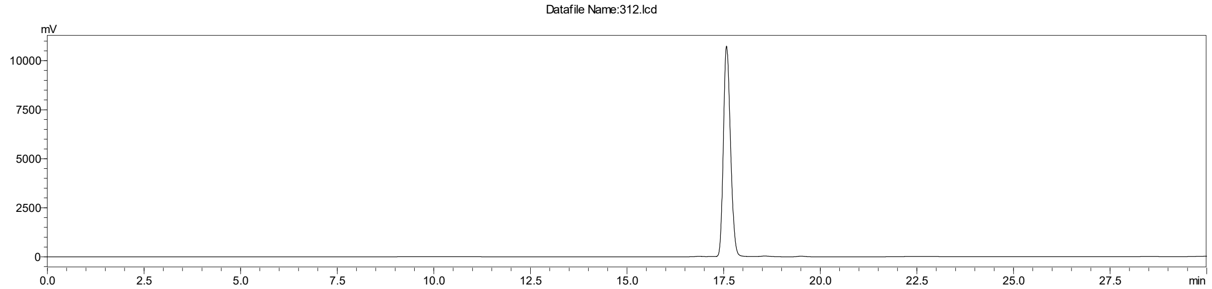

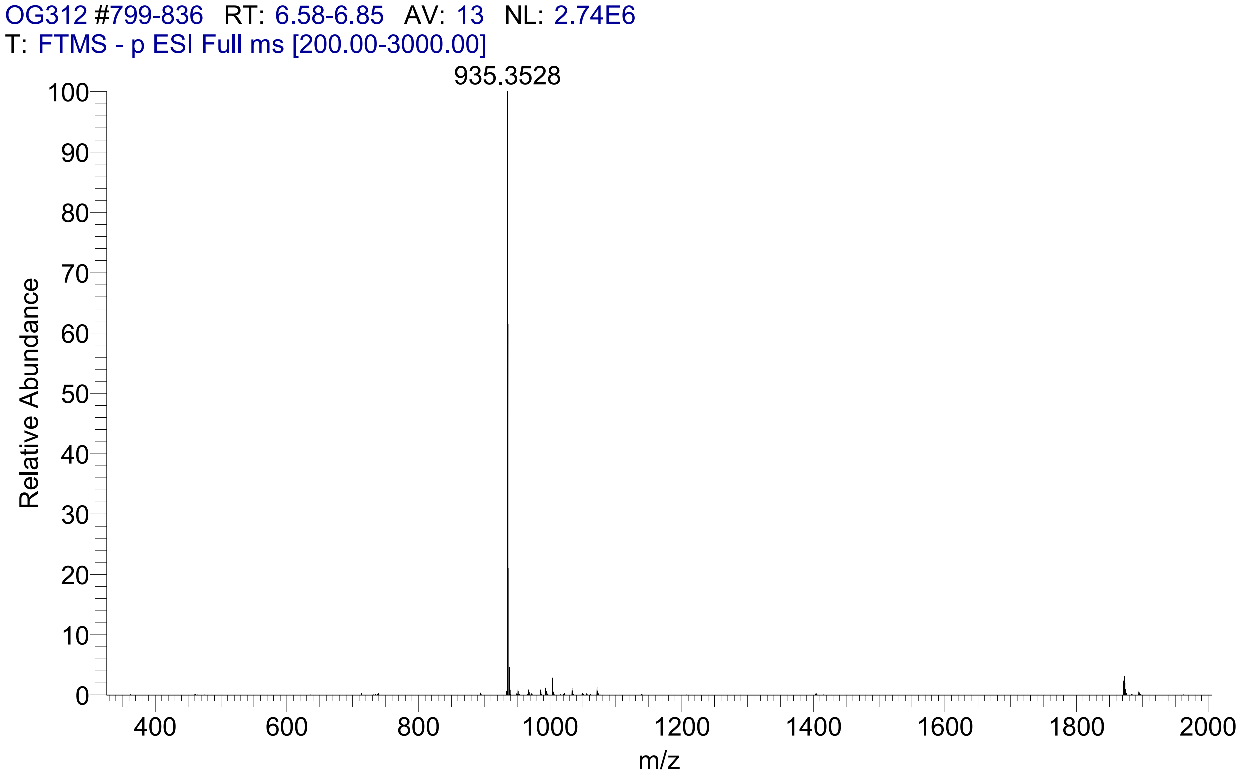

Galβ1-4GlcNAcβ1-3(Galβ1-4GlcNAcβ1-6)GalNAcα-Ser-Fmoc (80)

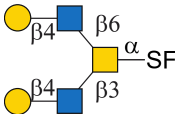

Compound **80** was prepared according to general procedure of β1-4 galactosylation with NmLgtB. After lyophilization, **80** was obtained as white solid. Compound was characterized by HPLC, T_R =12.556 min. ^{1}H NMR (600 MHz, D_2O) δ 7.88 – 7.74 (m, 2H), 7.69 – 7.50 (m, 2H), 7.48 - 7.31 (m, 4H), 4.70 (s, 1H), 4.57 (d, J = 4.6 Hz, 1H), 4.47 (d, J = 8.4 Hz, 3H), 4.39 – 4.31 (s, 2H), 4.24 - 4.09 (m, 3H), 3.98 – 3.85 (m, 7 H), 3.81 – 3.47 (m, 23H), 1.98 (s, 3H), 1.93 (s, 6H). HRMS, $C_{42}H_{56}N_4O_{20}$, Calcd for: 1260.4544; found [M-H]$^-$ 1259.462.

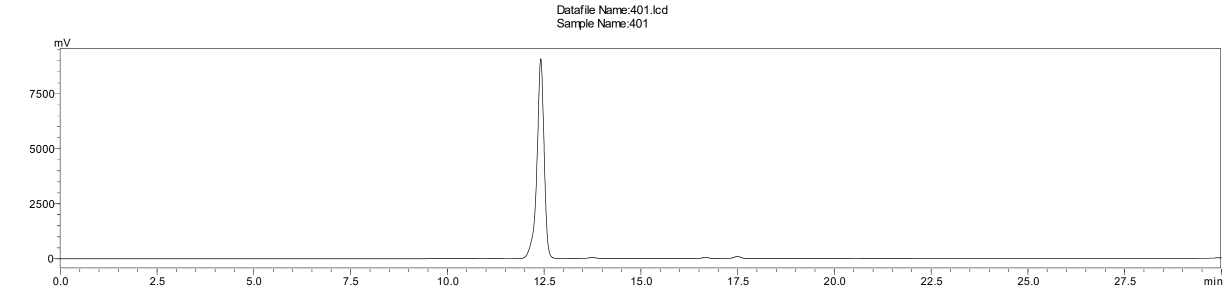

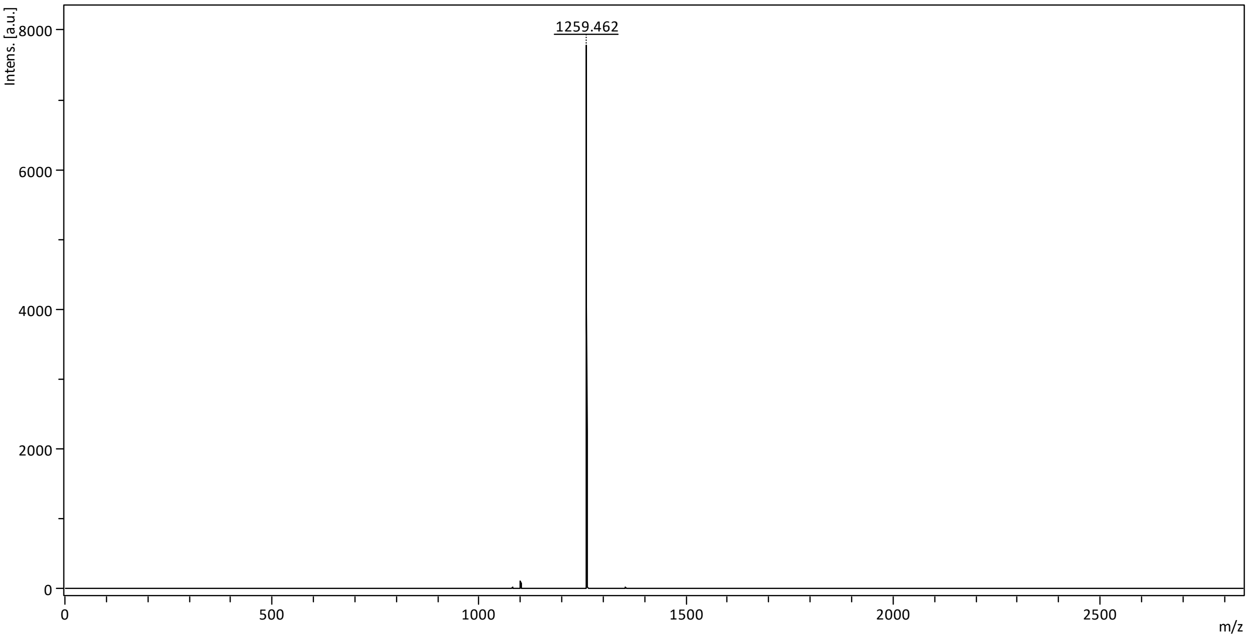

Neu5Acα2-3(GalNAcβ1-4)Galβ1-4GlcNAcβ1-3[Neu5Acα2-3(GalNAcβ1-4)Galβ1-4GlcNAcβ1-6]GalNAcα-Ser-Fmoc (81)

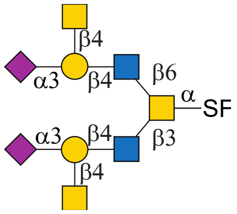

Compound **81** was prepared according to general procedure of β1-4-*N*-acetylgalatosaminylation with CgtA. After lyophilization, **81** was obtained as white solid. Compound was characterized by HPLC, T_R =7.835 min. ^{1}H NMR (600 MHz, D_2O) δ 7.99 – 7.88 (m, 2H), 7.80 – 7.63 (m, 2H), 7.59 - 7.42 (m, 4H), 4.59 (d, *J* = 7.6 Hz, 2H), 4.57 - 4.44 (m, 4H), 4.44 – 4.33 (m, 3H), 4.28 - 4.06 (m, 7H), 4.05 – 3.35 (m, 54 H), 2.69 (d, *J* = 11.8 Hz, 2H), 2.05 (s, 3H), 2.04 (s, 3H), 2.04 (s, 3H), 2.02 (s, 3H), 1.99 (s, 3H), 1.96 (s, 3H), 1.95 (s, 3H). HRMS, $C_{92}H_{136}N_8O_{56}$, Calcd for: 2248.804; found $[M-H]^-$ 2248.8014, $[M-2H]^{2-}$ 1123.8902.

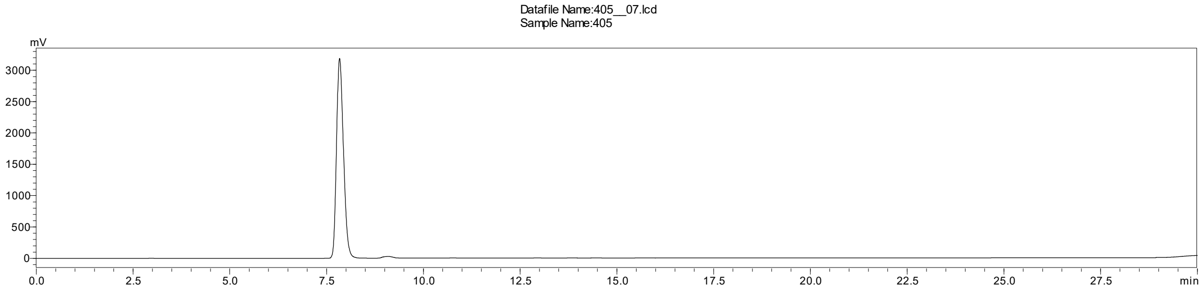

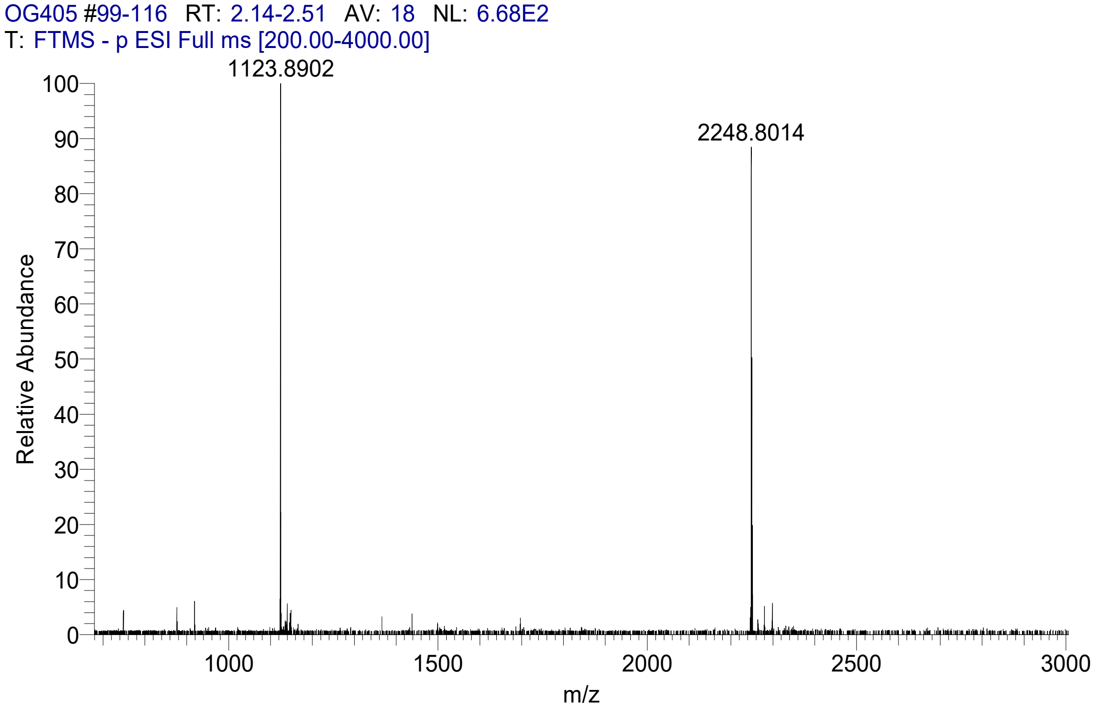

Neu5Acα2-6Galβ1-4GlcNAcβ1-3(Neu5Acα2-6Galβ1-4GlcNAcβ1-6)GalNAcα-Ser-Fmoc (82)

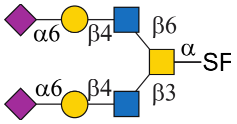

Compound **82** was prepared according to general procedure of α2-6 sialylation with Pd2,6ST. After lyophilization, **82** was obtained as white solid. Compound was characterized by HPLC, T_R =10.092 min. ^{1}H NMR (600 MHz, D_2O) δ 7.99 – 7.88 (m, 2H), 7.79 – 7.63 (m, 2H), 7.59 - 7.42 (m, 4H), 4.74 – 4.69 (m, 2H), 4.54 (d, J = 7.5 Hz, 2H), 4.48 (d, J = 7.5 Hz, 1H), 4.38 (m, 3H), 4.16 (s, 2H), 4.09 – 3.46 (m, 44 H), 2.67 (d, J = 13.0 Hz, 2H), 2.04 (s, 3H), 2.03 (s, 6H), 1.99 (s, 3H), 1.95 (s, 3H), 1.82 – 1.72 (m, 2H). HRMS, $C_{76}H_{110}N_6O_{46}$, Calcd for: 1842.6453; found $[M-H]^-$ 1841.6353, $[M-2H]^{2-}$ 920.3103.

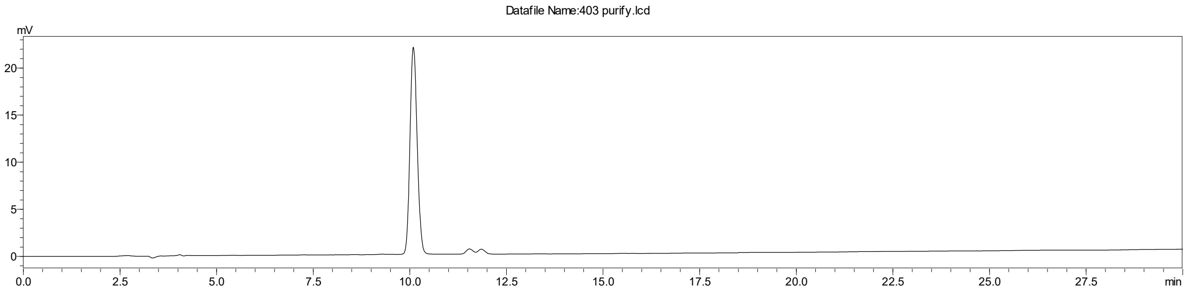

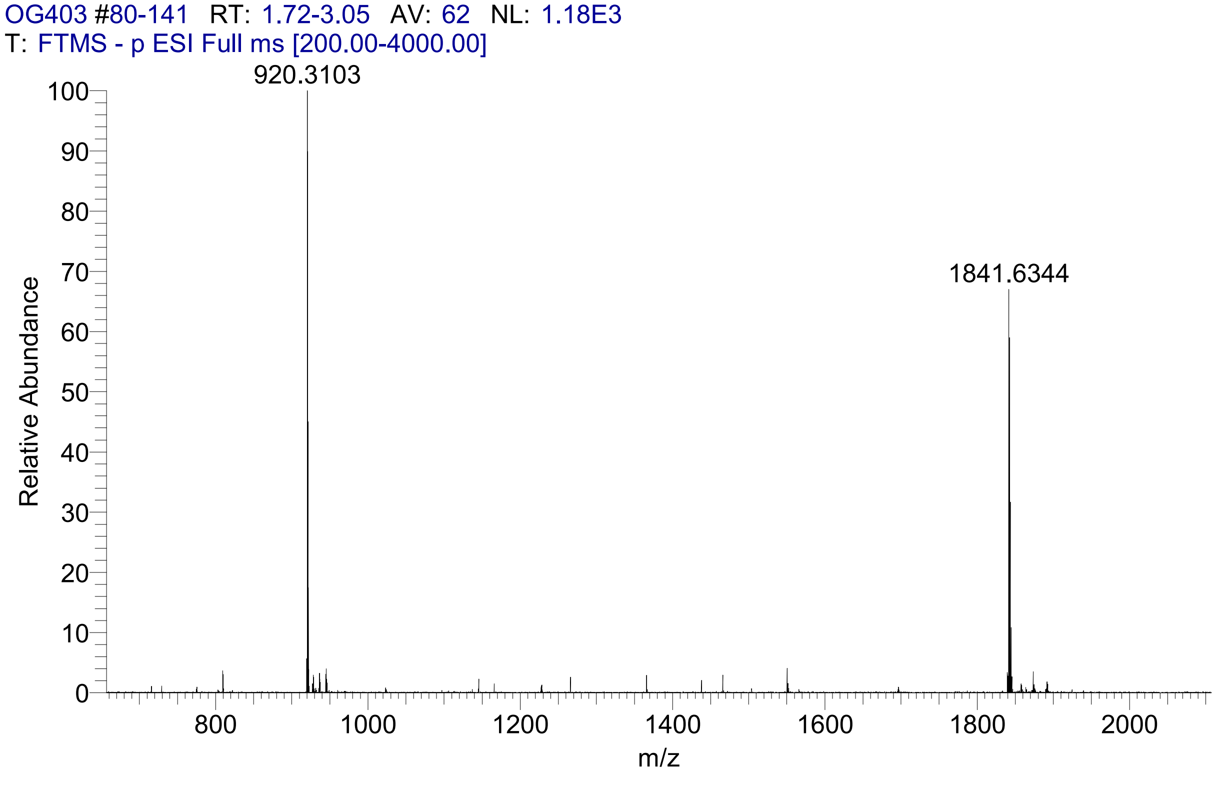

Fucα1-2Galβ1-4GlcNAcβ1-3(Fucα1-2Galβ1-4GlcNAcβ1-6)GalNAcα-Ser-Fmoc (83)

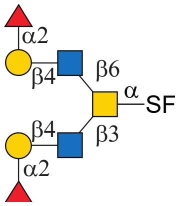

Compound **83** was prepared according to general procedure of α1-2 fucosylation with Hm2FT. After lyophilization, **83** was obtained as white solid. Compound was characterized by HPLC, T_R =11.878 min. ^{1}H NMR (600 MHz, D_2O) δ 7.97 – 7.88 (m, 2H), 7.81 – 7.65 (m, 2H), 7.57 - 7.40 (m, 4H), 5.35 – 5.26 (m, 2H), 4.62 – 4.53 (m, 3H), 4.51 (d, *J* = 8.2 Hz, 1H), 4.45 - 4.32 (m, 3H), 4.25 – 4.13 (m, 4H), 4.02 - 3.57 (m, 32 H), 3.50 – 3.37 (m, 2H), 2.78 (d, *J* = 12.5, 4.5 Hz, 2H), 2.02 (s, 3H), 1.97 (s, 3H), 1.96 (s, 3H), 1.23 (d, *J* = 6.5 Hz, 3H), 1.21 (d, *J* = 6.6 Hz, 3H). HRMS, $C_{66}H_{96}N_4O_{38}$, Calcd for: 1552.5703; found $[M-H]^-$ 1551.372.

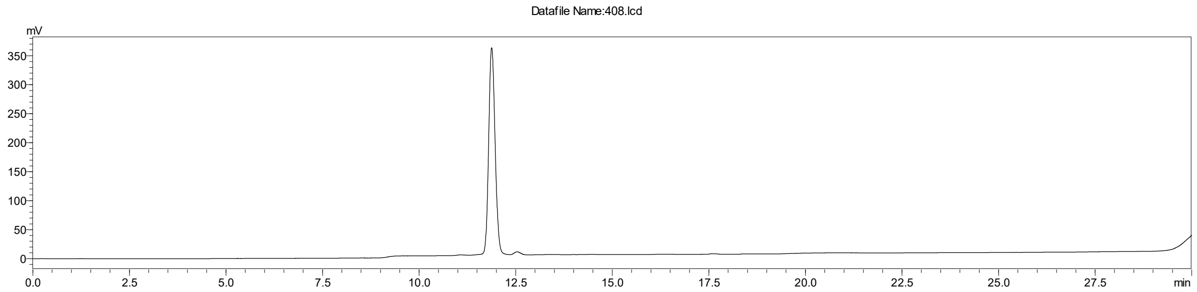

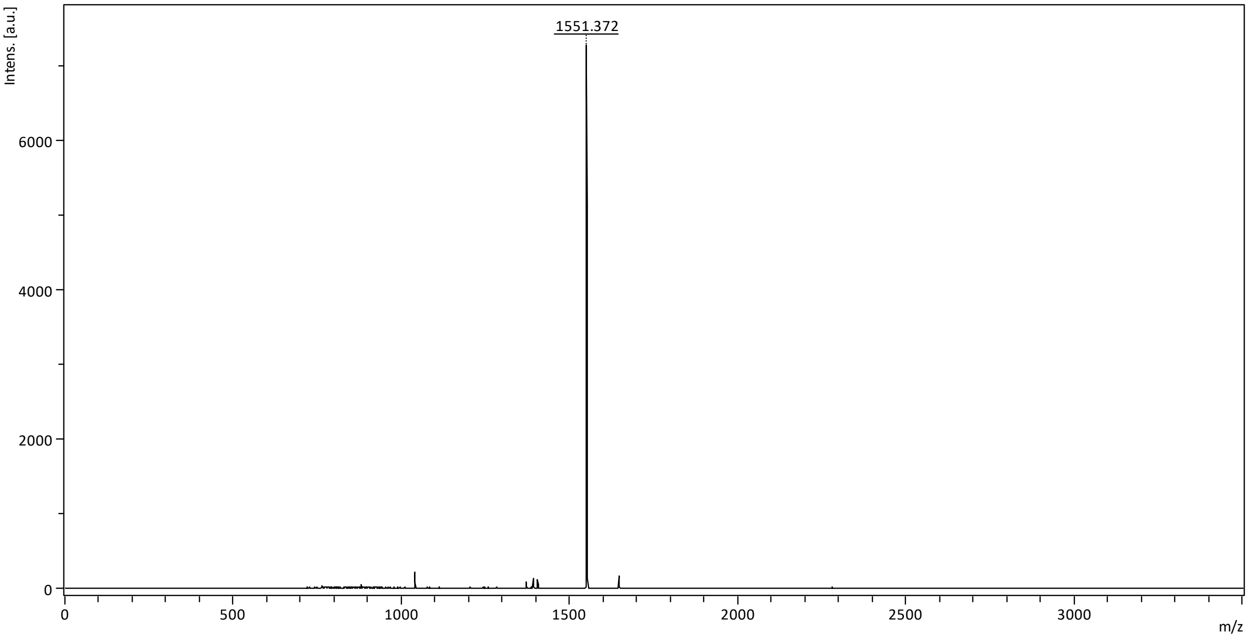

GalNAcα1-3(Fucα1-2)Galβ1-4GlcNAcβ1-3[GalNAcα1-3(Fucα1-2)Galβ1-4GlcNAcβ1-6]GalNAcα-Ser-Fmoc (84)

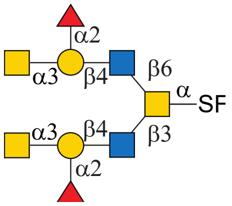

Compound **84** was prepared according to general procedure of α1-3-*N*-acetylgalatosaminylation with BgtA. After lyophilization, **84** was obtained as white solid. Compound was characterized by HPLC, T_R =11.803 min. ^{1}H NMR (600 MHz, D_2O) δ 7.97 – 7.84 (m, 2H), 7.79 – 7.60 (m, 2H), 7.55 - 7.37 (m, 4H), 5.36 (d, *J* = 3.9 Hz, 2H), 5.18 (d, *J* = 3.9 Hz, 2H), 4.65 – 4.54 (m, 3H), 4.49 (d, *J* = 8.1 Hz, 2H), 4.44 - 4.34 (m, 2H), 4.31 (d, *J* = 6.9 Hz, 2H), 4.25 (dd, *J* = 10.8, 3.5 Hz, 1H), 4.25 – 4.09 (m, 7H), 4.07 - 3.60 (m, 44 H), 3.49 – 3.35 (m, 2H), 2.05 (s, 6H), 2.01 (s, 3H), 1.95 (s, 6H), 1.32 - 1.19 (m, 6H). HRMS, $C_{82}H_{122}N_6O_{48}$, Calcd for: 1958.7290; found $[M-H]^-$ 1957.713.

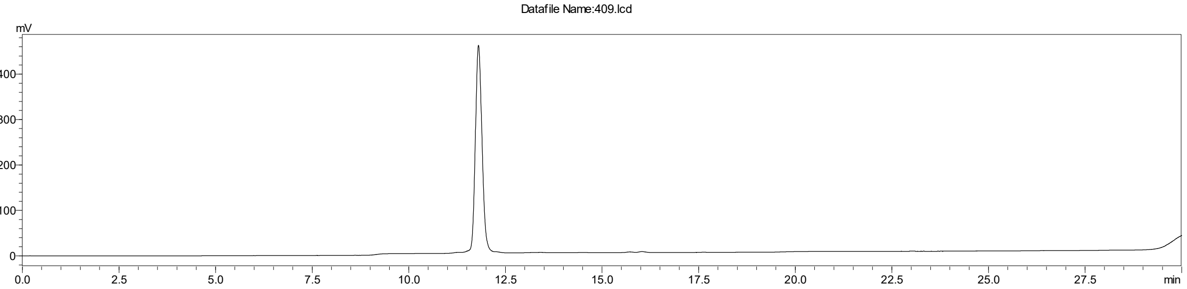

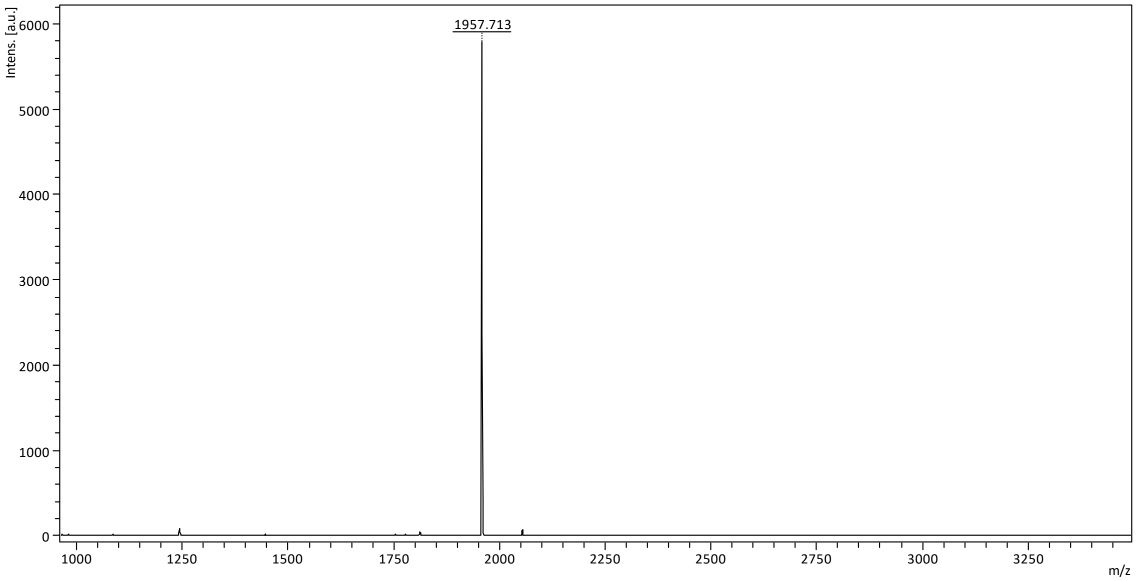

Galα1-3(Fucα1-2)Galβ1-4GlcNAcβ1-3[Galα1-3(Fucα1-2)Galβ1-4GlcNAcβ1-6]GalNAcα-Ser-Fmoc (85)

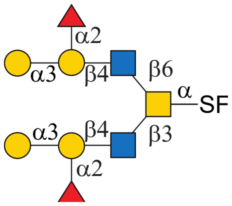

Compound **85** was prepared according to general procedure of α1-3 galactosylation with GTB. After lyophilization, **85** was obtained as white solid. Compound was characterized by HPLC, T_R =11.146 min. ^{1}H NMR (600 MHz, D_2O) δ 7.96 – 7.83 (m, 2H), 7.79 – 7.60 (m, 2H), 7.56 - 7.36 (m, 4H), 5.34 (d, *J* = 3.4 Hz, 2H), 5.25 (d, *J* = 3.7 Hz, 2H), 4.57 – 4.46 (m, 3H), 4.40 – 4.26 (m, 7H), 4.25 - 4.11 (m, 5H), 4.04 - 3.59 (m, 45 H), 3.50 – 3.35 (m, 2H), 2.01 (s, 3H), 1.95 (s, 6H), 1.25 (d, *J* = 6.8 Hz, 3H), 1.22 (d, *J* = 7.3 Hz, 3H). HRMS, $C_{78}H_{116}N_4O_{48}$, Calcd for: 1876.6759; found $[M-H]^-$ 1875.759.

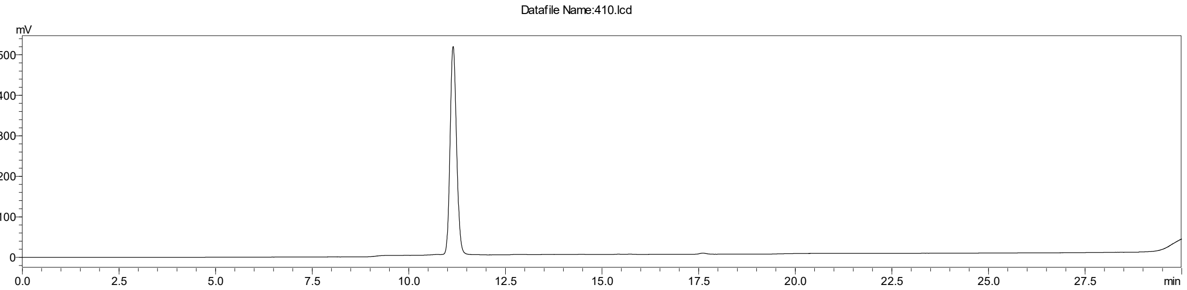

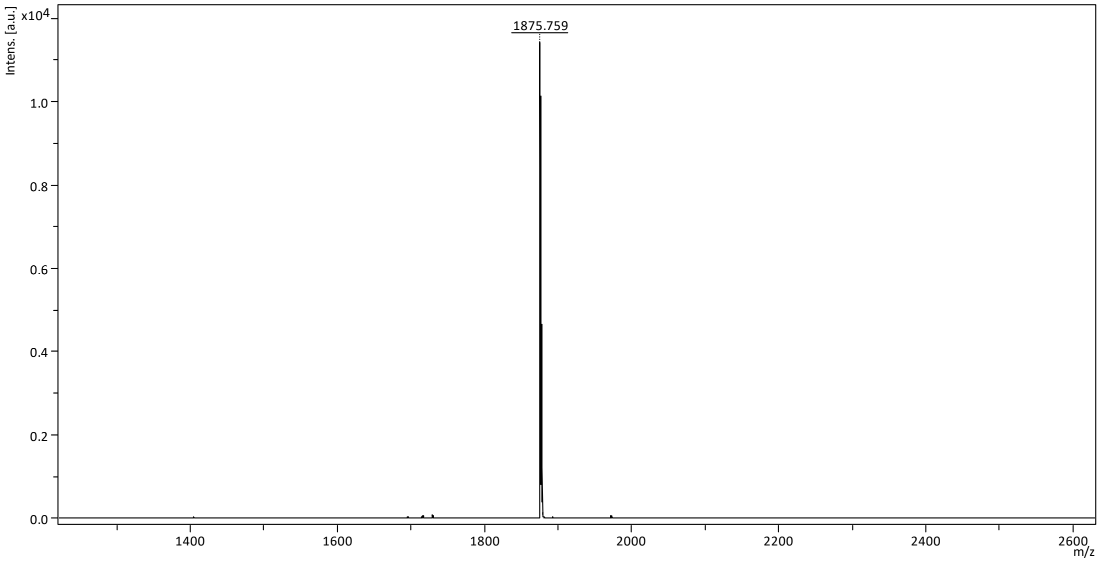

Fucα1-2Galβ1-4(Fucα1-3)GlcNAcβ1-3[Fucα1-2Galβ1-4(Fucα1-3)GlcNAcβ1-6]GalNAcα-Ser-Fmoc (86)

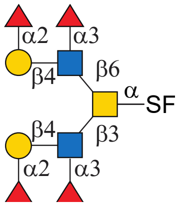

Compound **86** was prepared according to general procedure of α1-3 fucosylation with Hp3FT. After lyophilization, **86** was obtained as white solid. Compound was characterized by HPLC, T_R =10.425 min. ^{1}H NMR (600 MHz, D_2O) δ 7.96 – 7.85 (m, 2H), 7.80 – 7.62 (m, 2H), 7.56 - 7.39 (m, 4H), 5.27 (d, *J* = 3.2 Hz, 2H), 5.09 (d, *J* = 3.3 Hz, 2H), 4.56 – 4.50 (m, 2H), 4.43 – 4.32 (m, 3H), 4.24 (d, *J* = 6.6 Hz, 2H), 4.22 - 4.12 (m, 3H), 4.04 - 3.36 (m, 46 H), 1.99 (s, 3H), 1.96 (s, 6H), 1.33 – 1.17 (m, 12H). HRMS, $C_{78}H_{116}N_4O_{46}$, Calcd for: 1844.6861; found $[M-H]^-$ 1843.613.

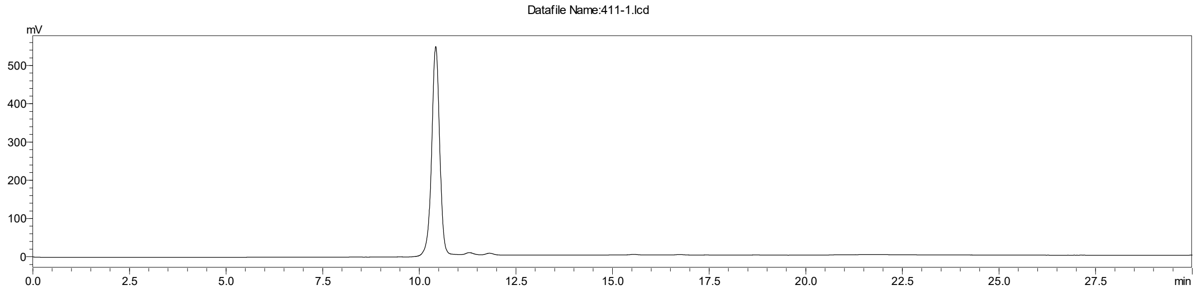

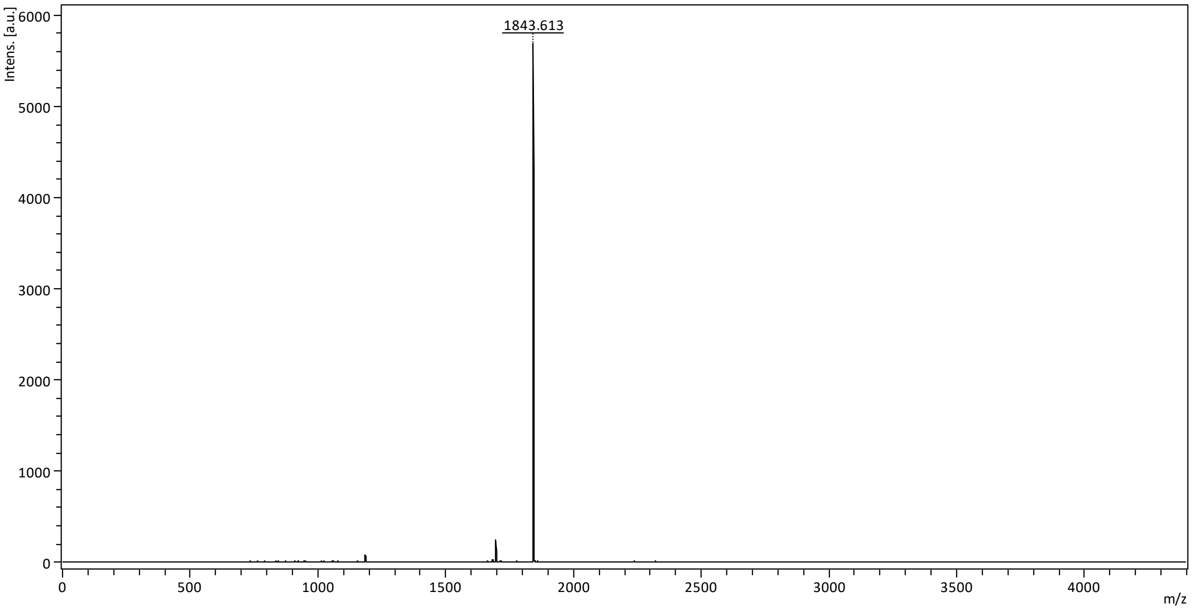

Galα1-3Galβ1-4GlcNAcβ1-3(Galα1-3Galβ1-4GlcNAcβ1-6)GalNAcα-Ser-Fmoc (87)

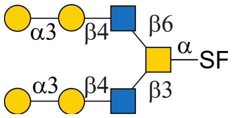

Compound **87** was prepared according to general procedure of α1-3 galactosylation with α3GalT. After lyophilization, **87** was obtained as white solid. Compound was characterized by HPLC, T_R =10.996 min. ^{1}H NMR (600 MHz, D_2O) δ 7.97 – 7.86 (m, 2H), 7.79 – 7.62 (m, 2H), 7.57 - 7.39 (m, 4H), 5.18 (d, *J* = 3.9 Hz, 1H), 5.15 (d, *J* = 4.0 Hz, 1H), 4.74 – 4.67 (m, 2H), 4.58 (d, *J* = 7.9 Hz, 1H), 4.51 (d, *J* = 7.6 Hz, 2H), 4.46 (d, *J* = 7.6 Hz, 1H), 4.34 (s, 2H), 4.25 – 4.10 (m, 6H), 4.03 (d, *J* = 2.9 Hz, 1H), 4.01 – 3.47 (m, 37H), 2.00 (s, 3H), 1.95 (s, 6H). HRMS, Calcd for: 1584.5601; found $[M-H]^-$ 1583.514.

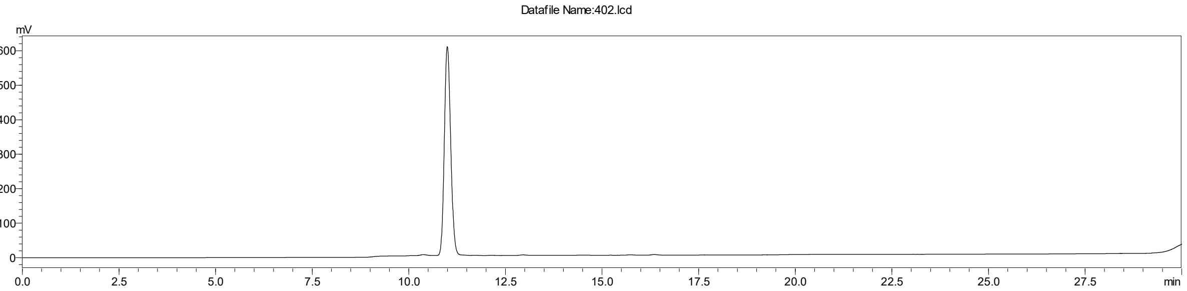

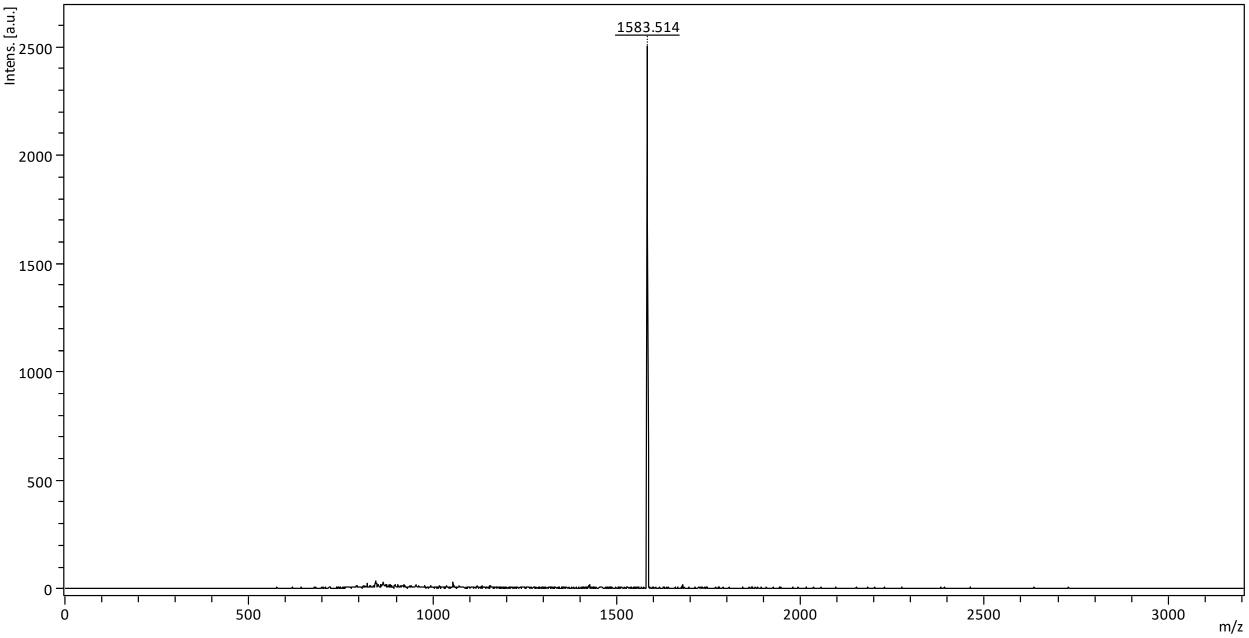

Galβ1-4(Fucα1-3)GlcNAcβ1-3[Galβ1-4(Fucα1-3)GlcNAcβ1-6]GalNAcα-Ser-Fmoc (88)

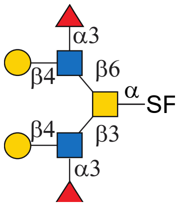

Compound **88** was prepared according to general procedure of α1-3 fucosylation with Hp3FT. After lyophilization, **88** was obtained as white solid. Compound was characterized by HPLC, T_R =10.394 min. ^{1}H NMR (600 MHz, D_2O) δ 7.96 – 7.84 (m, 2H), 7.78 – 7.58 (m, 2H), 7.57 - 7.36 (m, 4H), 5.14 – 5.06 (m, 2H, 2×Fuc-H-1), F4.58 – 4.45 (m, 3H), 4.44 - 4.28 (m, 3H), 4.18 – 4.09 (m, 2H), 4.05 - 3.45 (m, 36 H), 1.98 (s, 3H), 1.94 (s, 3H), 1.93 (s, 3H), 1.31 – 1.13 (m, 6H, 2× Fuc-CH_3). HRMS, $C_{66}H_{96}N_4O_{38}$, Calcd for: 1552.5703; found $[M+H]^+$ 1553.5776, $[M+Na]^+$ 1575.5573.

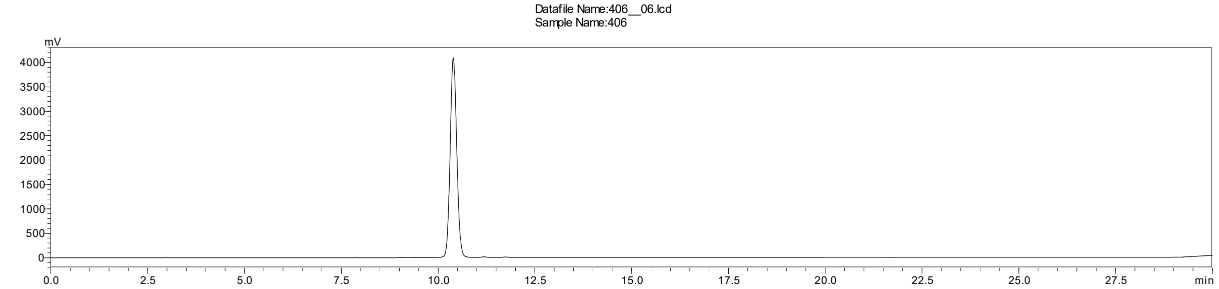

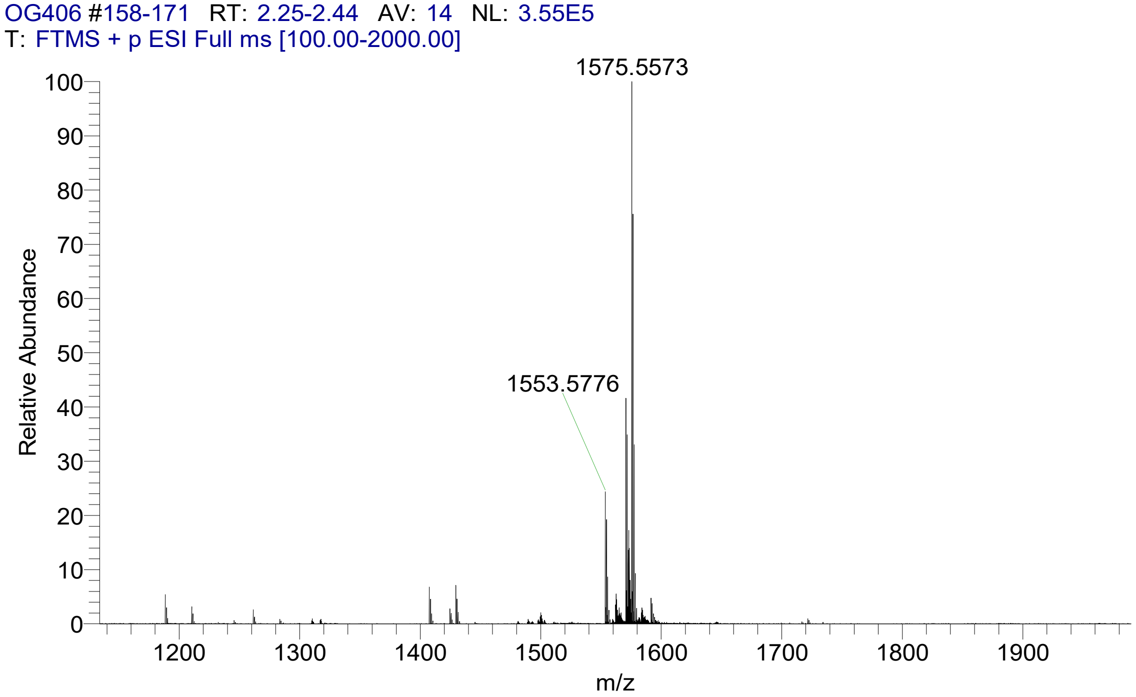

Neu5Acα2-3Galβ1-4(Fucα1-3)GlcNAcβ1-3[Neu5Acα2-3Galβ1-4(Fucα1-3)GlcNAcβ1-6]GalNAcα-Ser-Fmoc (89)

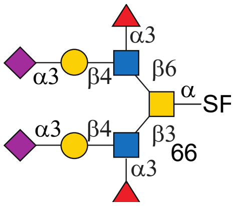

Compound **89** was prepared according to general procedure of α2-3 sialylation with PmST1-M144D. After lyophilization, **89** was obtained as white solid. Compound was characterized by HPLC, T_R =8.518 min. NMR (600 MHz, D_2O) δ 8.01 – 7.87 (m, 2H), 7.80 – 7.62 (m, 2H), 7.59 - 7.38 (m, 4H), 5.13 – 5.08 (m, 2H, 2×Fuc-H-1), 4.61 – 4.47 (m, 4H), 4.43 (d, J = 7.8 Hz, 1H), 4.41 - 4.31 (m, 2H), 4.20 – 4.06 (m, 4H), 4.05 - 3.47 (m, 51 H), 2.78 (d, J = 12.5, 4.5 Hz, 2H, 2×Neu5Ac H), 2.05 (s, 3H), 2.04 (s, 3H), 1.98 (s, 3H), 1.96 (s, 3H),1.94 (s, 3H), 1.84 (t, J = 12.3 Hz, 2H), 1.21 (d, J = 5.7 Hz, 3H), 1.17 (d, J = 6.1 Hz, 3H). HRMS, $C_{88}H_{130}N_6O_{54}$, Calcd for: 2134.7611; found $[M+Na]^+$ 2158.663.

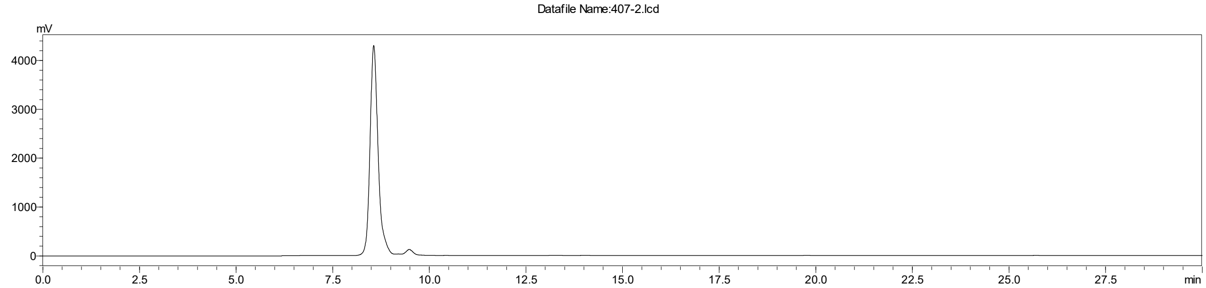

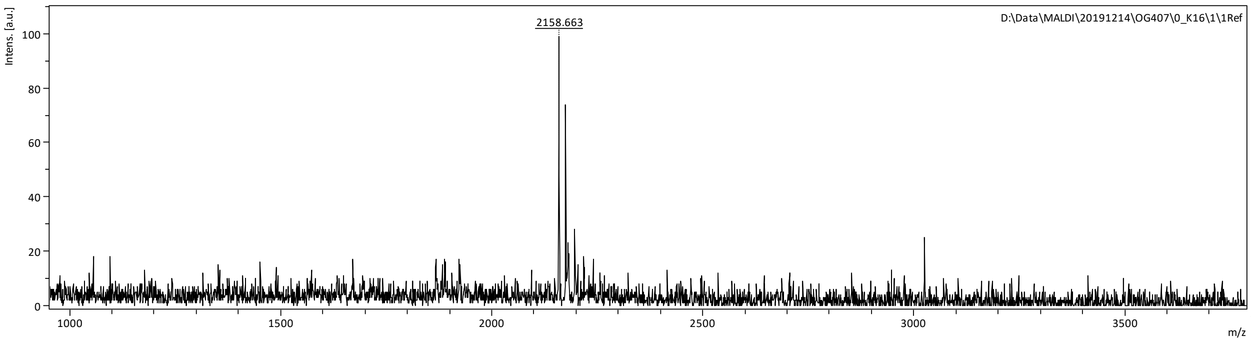

^{1}H

Galβ1-4GlcNAcβ1-6GalNAcα-Ser-Fmoc (90)

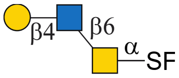

Compound **90** was prepared according to general procedure of β1-4 galactosylation with NmLgtB. After lyophilization, **90** was obtained as white solid. Compound was characterized by HPLC, T_R =17.904 min. ^{1}H NMR (600 MHz, D_2O) δ 7.93 – 7.82 (m, 2H), 7.76 – 7.59 (m, 2H), 7.54 - 7.36 (m, 4H), 4.65 – 4.57 (m, 1H), 4.51 (d, *J* = 8.2 Hz, 1H), 4.38 (d, *J* = 7.8 Hz, 1H), 4.32 (s, 1H), 4.30 (s, 1H), 4.10 - 3.49 (m, 21 H), 1.95 (s, 3H), 1.94 (s, 3H). HRMS, $C_{40}H_{53}N_3O_{20}$, Calcd for: 895.3222; found $[M+H]^+$ 896.3263.

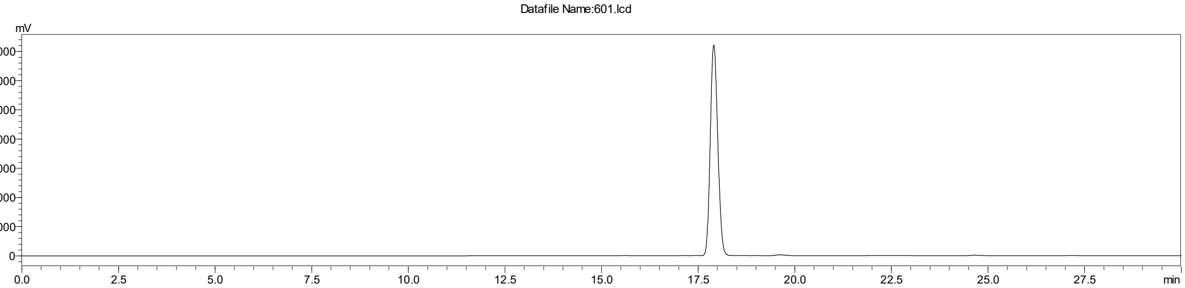

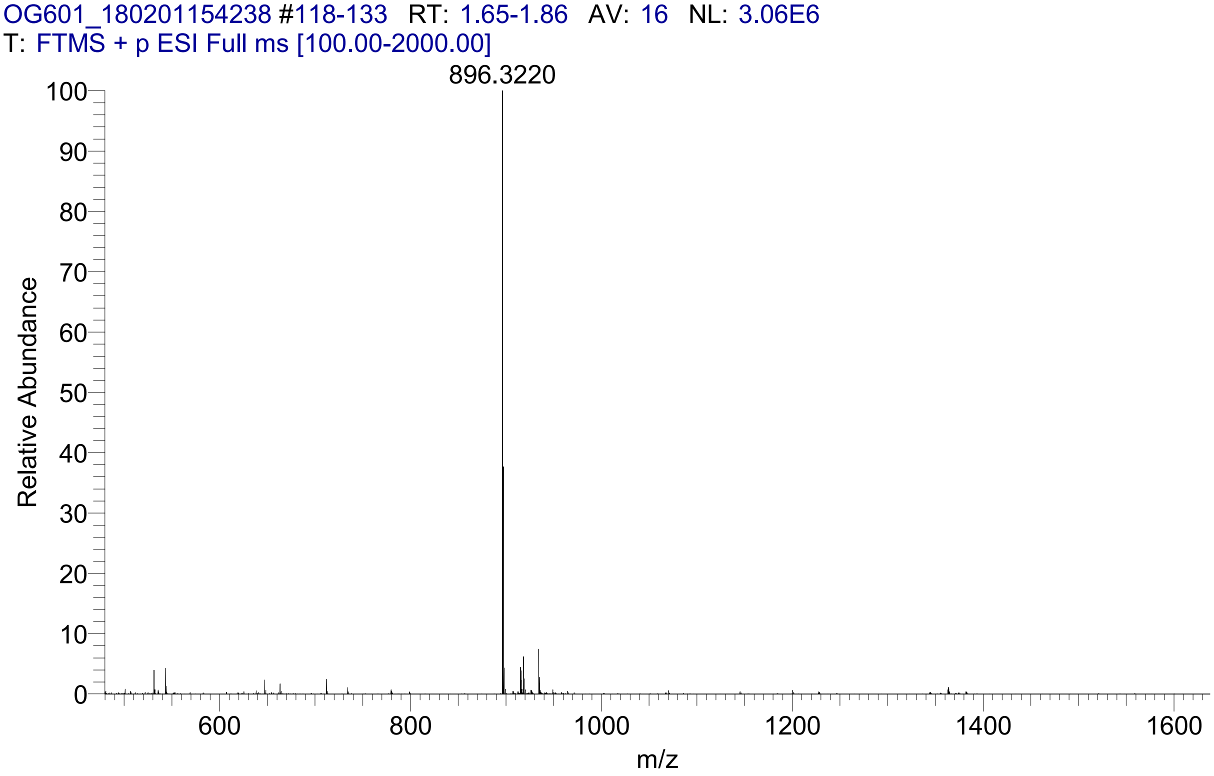

Neu5Acα2-3Galβ1-4GlcNAcβ1-6GalNAcα-Ser-Fmoc (91)

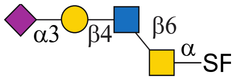

Compound **91** was prepared according to general procedure of α2-3 sialylation with PmST1-M144D. After lyophilization, **91** was obtained as white solid. Compound was characterized by HPLC, T_R =15.103 min. ^{1}H NMR (600 MHz, D_2O) δ 7.95 – 7.85 (m, 2H), 7.76 – 7.60 (m, 2H), 7.54 - 7.38 (m, 4H), 4.71 (m, 1H), 4.62 (m, 1H), 4.50 (d, *J* = 8.2 Hz, 1H), 4.44 (d, *J* = 7.8 Hz, 1H), 4.33 (m, 2H), 4.17 – 3.86 (m, 10H), 3.84 – 3.47 (m, 17H), 2.77 (dd, *J* = 12.5, 4.6 Hz, 1H), 2.04 (s, 3H), 1.95 (s, 3H), 1.94 (s, 3H), 1.84 (t, *J* = 12.2 Hz, 1H). HRMS, $C_{51}H_{70}N_4O_{28}$, Calcd for: 1186.4177; found $[M-H]^-$ 1185.306.

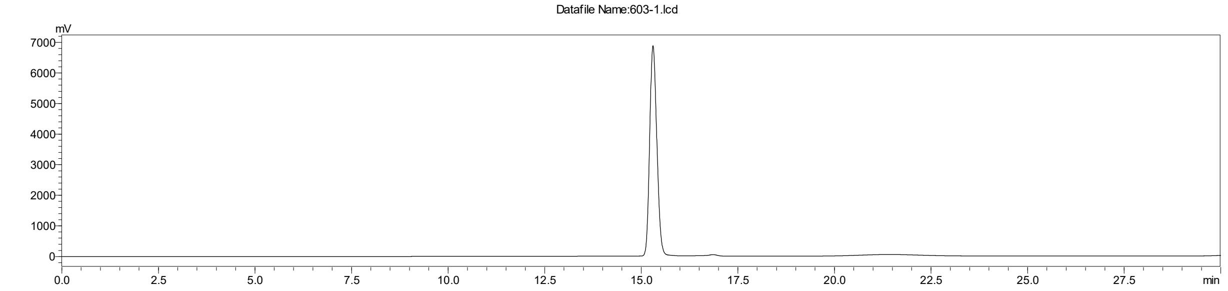

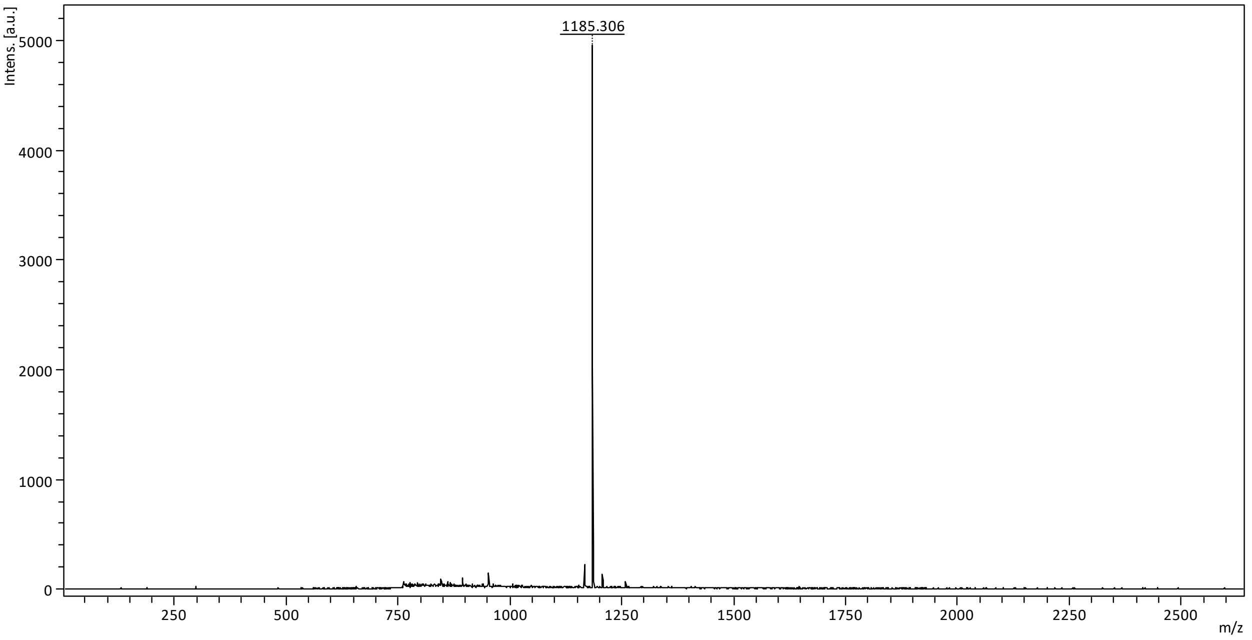

Neu5Acα2-3(GalNAcβ1-4)Galβ1-4GlcNAcβ1-6GalNAcα-Ser-Fmoc (92)

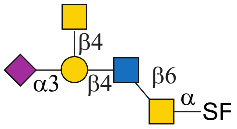

Compound **92** was prepared according to general procedure of β1-4-N-acetylgalatosaminylation with CgtA. After lyophilization, **92** was obtained as white solid. Compound was characterized by HPLC, T_R =13.680 min. ^{1}H NMR (600 MHz, D_2O) δ 7.97 – 7.86 (m, 2H), 7.77 – 7.62 (m, 2H), 7.55 - 7.39 (m, 4H), 4.72 (d, J = 8.5 Hz, 2H), 4.66 (m, 1H), 4.50 (d, J = 8.2 Hz, 1H), 4.50 (d, J = 8.3 Hz, 1H), 4.35 (m, 2H), 4.17 – 4.10 (m, 2H), 4.09 – 3.48 (m, 32H), 3.37 (t, J = 8.64 Hz, 1H), 2.04 (s, 3H), 1.02 (s, 3H), 1.95 (s, 3H), 1.94 (s, 3H). HRMS, $C_{59}H_{83}N_5O_{33}$, Calcd for: 1389.479; found $[M-H]^-$ 1388.472.

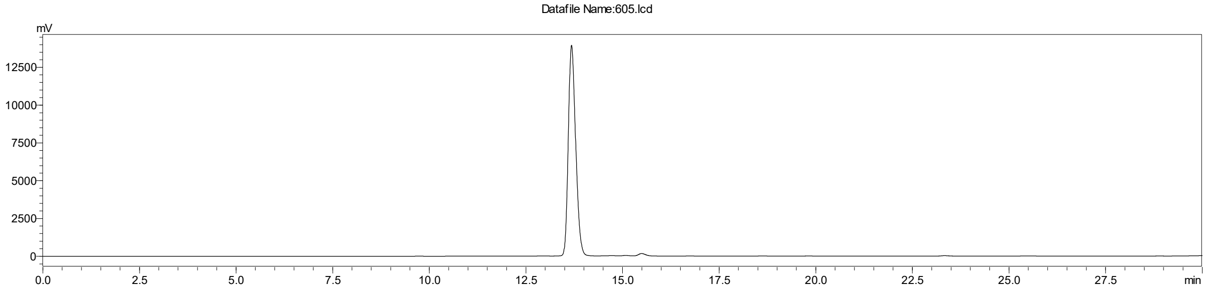

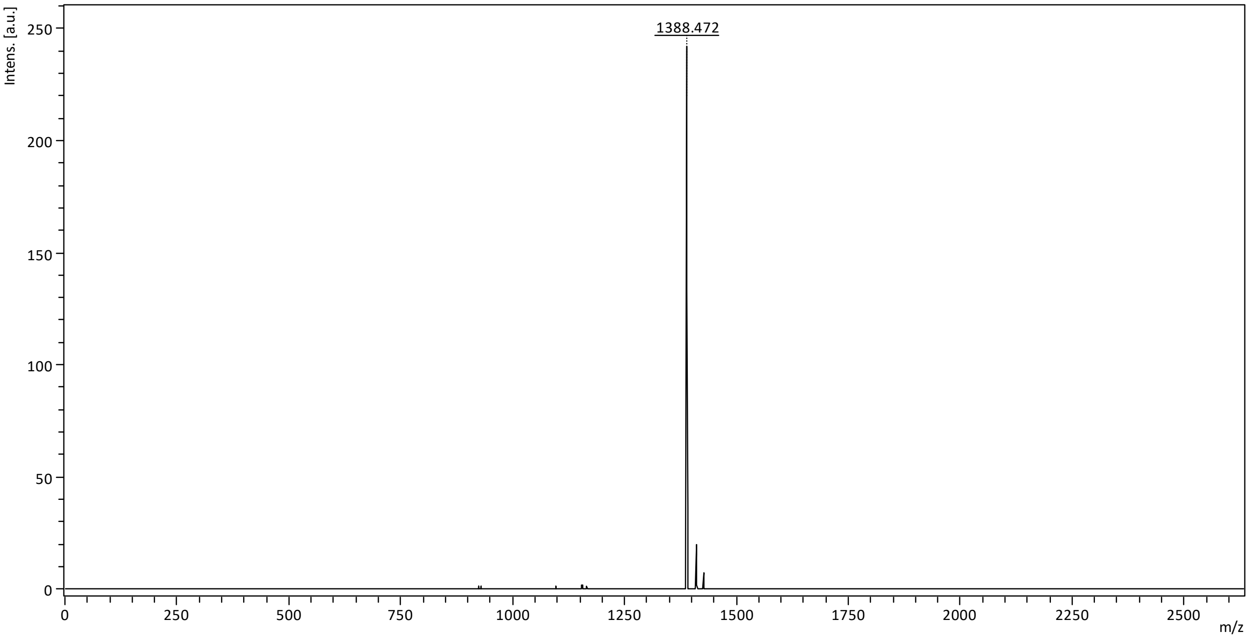

Neu5Acα2-6Galβ1-4GlcNAcβ1-6GalNAcα-Ser-Fmoc (93)

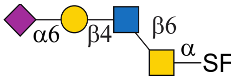

Compound **93** was prepared according to general procedure of α2-6 sialylation with Pd2,6ST. After lyophilization, **93** was obtained as white solid. Compound was characterized by HPLC, T_R =14.753 min. ^1H NMR (600 MHz, D_2O) δ 7.94 – 7.85 (m, 2H), 7.77 – 7.60 (m, 2H), 7.54 - 7.38 (m, 4H), 4.71 (m, 1H), 4.62 (m, 1H), 4.55 (d, *J* = 7.8 Hz, 1H), 4.41 – 4.29 (m, 3H), 4.10 – 3.46 (m, 27H), 2.67 (dd, *J* = 12.5, 4.6 Hz, 1H), 2.03 (s, 3H), 1.99 (s, 3H), 1.94 (s, 3H), 1.78 (t, *J* = 12.2 Hz, 1H). HRMS, $C_{51}H_{70}N_4O_{28}$, Calcd for: 1186.4177; found $[M-H]^-$ 1185.396.

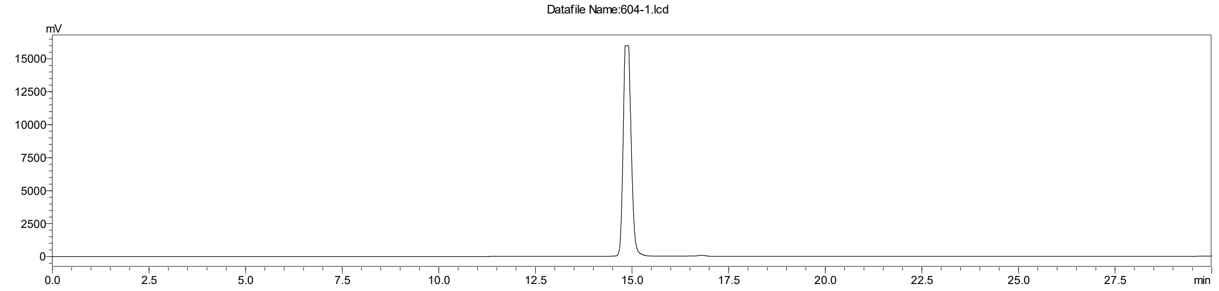

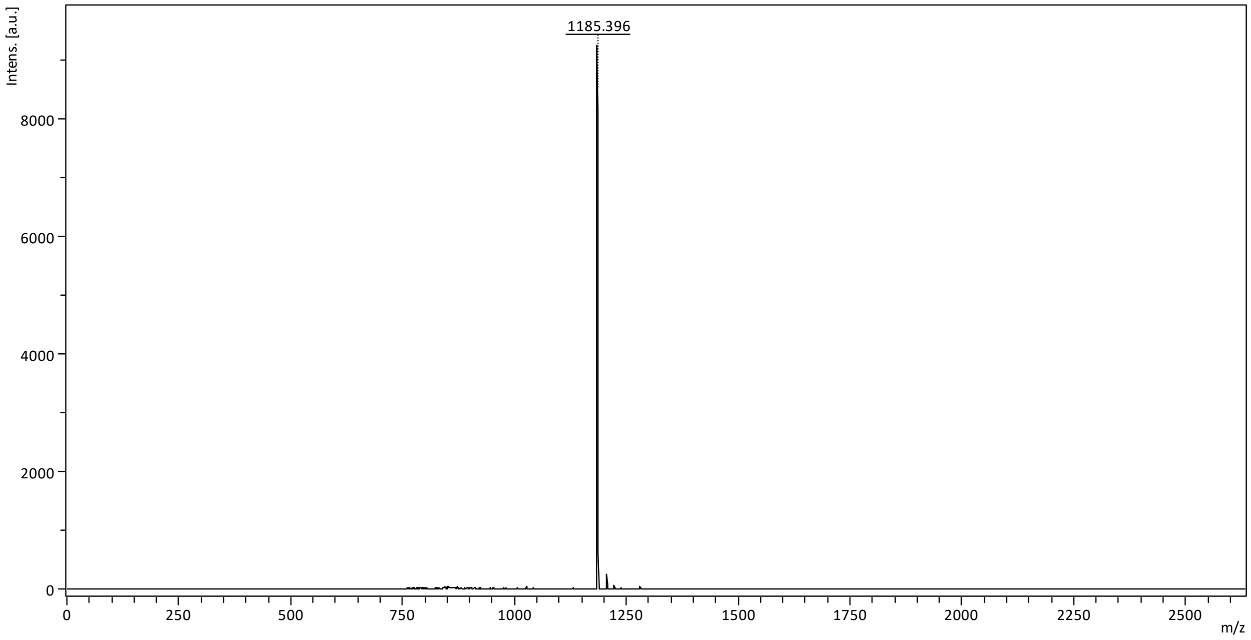

Fucα1-2Galβ1-4GlcNAcβ1-6GalNAcα-Ser-Fmoc (94)

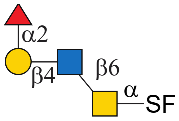

Compound **94** was prepared according to general procedure of α1-2 fucosylation with Hm2FT. After lyophilization, **94** was obtained as white solid. Compound was characterized by HPLC, T_R =17.252 min. ^{1}H NMR (600 MHz, D_2O) δ 7.94 – 7.84 (m, 2H), 7.76 – 7.60 (m, 2H), 7.53 -7.38 (m, 4H), 5.29 (d, *J* = 3.3 Hz, 1H), 4.74 – 4.67 (m, 1H), 4.59 (s, 1H), 4.51 (d, *J* = 8.3 Hz, 2H), 4.43 (d, *J* = 7.6 Hz, 1H), 4.33 (d, *J* = 15.6 Hz, 2H), 4.17 (d, *J* = 8.0 Hz, 1H), 4.07 (d, *J* = 12.0 Hz, 1H), 3.99 – 3.59 (m, 19H), 3.42 (s, 2H), 1.96 (s, 3H), 1.95 (s, 3H), 1.21 (d, *J* = 6.9 Hz, 3H). HRMS, $C_{46}H_{63}N_3O_{24}$, Calcd for: 1041.3801; found $[M-H]^-$ 1040.357.

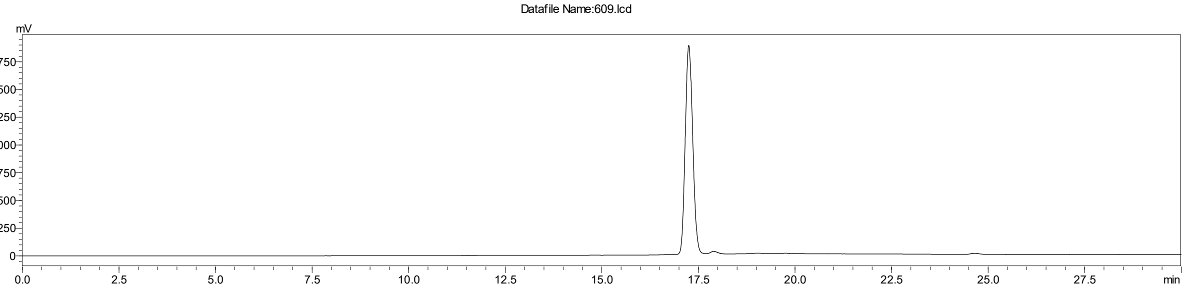

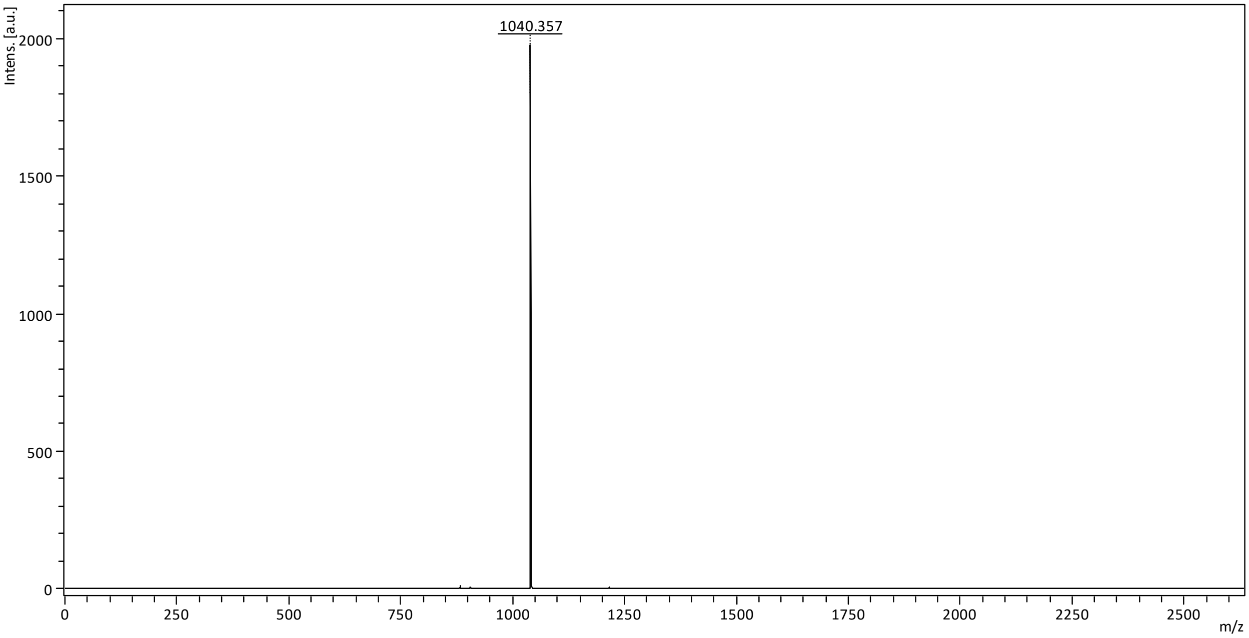

GalNAcα1-3(Fucα1-2)Galβ1-4GlcNAcβ1-6GalNAcα-Ser-Fmoc (95)

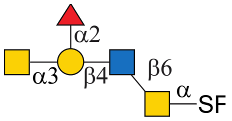

Compound **95** was prepared according to general procedure of α1-3-*N*-acetylgalatosaminylation with BgtA. After lyophilization, **95** was obtained as white solid. Compound was characterized by HPLC, T_R =16.753 min. ^{1}H NMR (600 MHz, D_2O) δ 7.93 – 7.82 (m, 2H), 7.76 – 7.57 (m, 2H), 7.53 – 7.37 (m, 4H), 5.36 (d, *J* = 3.9 Hz, 1H), 5.19 (d, *J* = 3.9 Hz, 1H), 4.71 (dd, *J* = 10.3, 5.4 Hz, 1H), 4.59 (s, 1H), 4.55 – 4.49 (m, 2H), 4.36 – 4.28 (m, 3H), 4.26 (d, *J* = 3.7 Hz, 1H), 4.25 – 4.21 (m, 2H), 4.11 – 3.60 (m, 25H), 3.43 (s, 1H), 2.05 (s, 3H), 1.96 (s, 3H), 1.94 (s, 3H), 1.24 (d, *J* = 6.8 Hz, 3H). HRMS, $C_{54}H_{76}N_4O_{29}$, Calcd for: 1244.4595; found [M-H]$^-$ 1243.431.

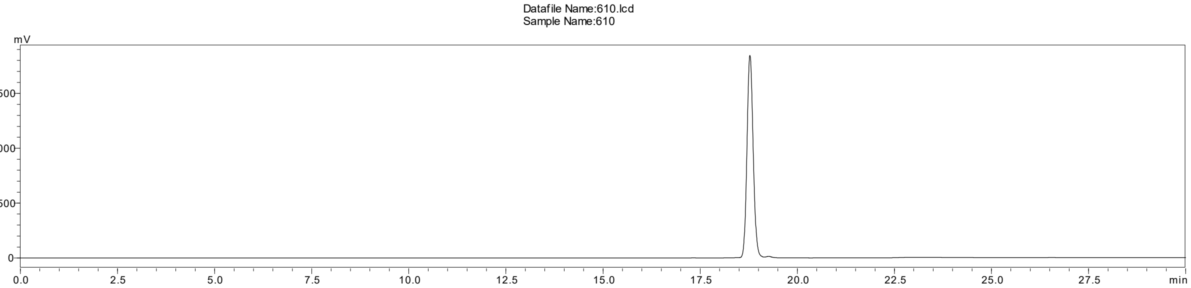

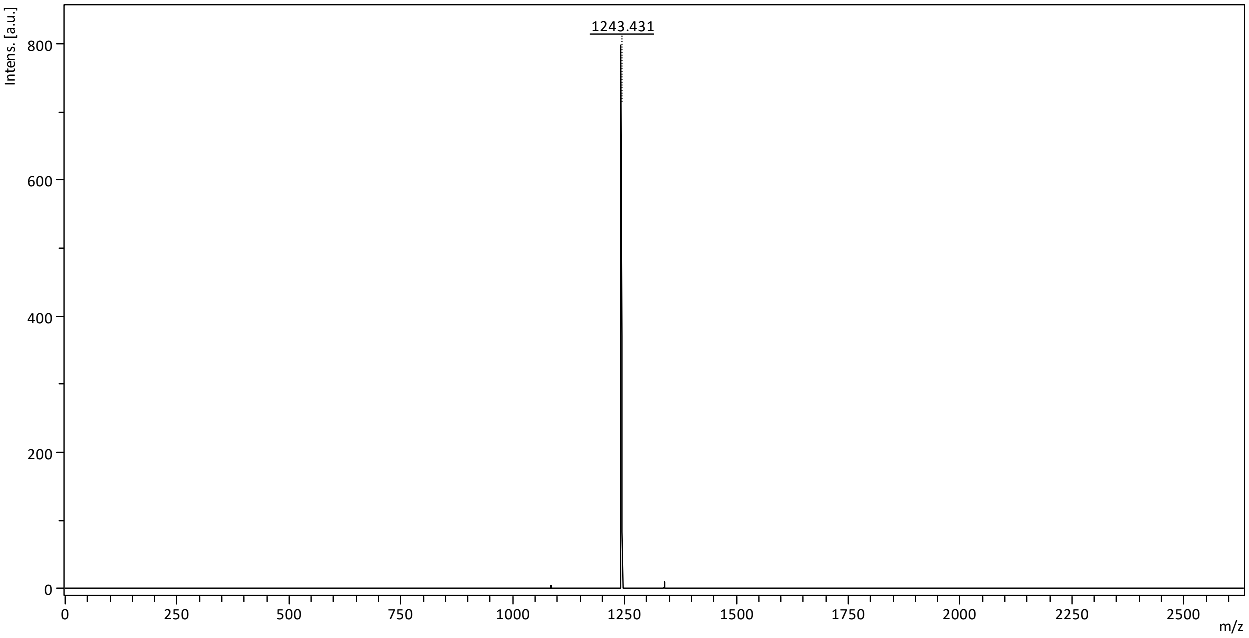

Galα1-3(Fucα1-2)Galβ1-4GlcNAcβ1-6GalNAcα-Ser-Fmoc (96)

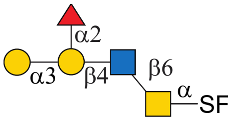

Compound **96** was prepared according to general procedure of α1-3 galactosylation with GTB. After lyophilization, **96** was obtained as white solid. Compound was characterized by HPLC, T_R =15.376 min. ^1H NMR (600 MHz, D_2O) δ 7.94 – 7.85 (m, 2H), 7.78 – 7.60 (m, 2H), 7.55 - 7.38 (m, 4H), 5.33 (d, *J* = 3.2 Hz, 1H), 5.26 (d, *J* = 2.8 Hz, 1H), 4.73 (s, 1H), 4.59 (s, 1H), 4.54 (d, *J* = 7.9 Hz, 1H), 4.51 (d, *J* = 8.4 Hz, 1H), 4.33 (s, 2H), 4.29 (s, 2H), 4.21 (t, *J* = 6.3 Hz, 1H), 4.07 (d, *J* = 11.5 Hz, 1H), 3.99 (s, 2H), 3.96 – 3.87 (m, 6H), 3.85 – 3.60 (m, 17H), 3.40 (s, 1H), 1.96 (s, 3H), 1.95 (s, 3H), 1.22 (d, *J* = 6.9 Hz, 3H). HRMS, $C_{52}H_{73}N_3O_{29}$, Calcd for: 1203.433, found $[M-H]^-$ 1202.420.

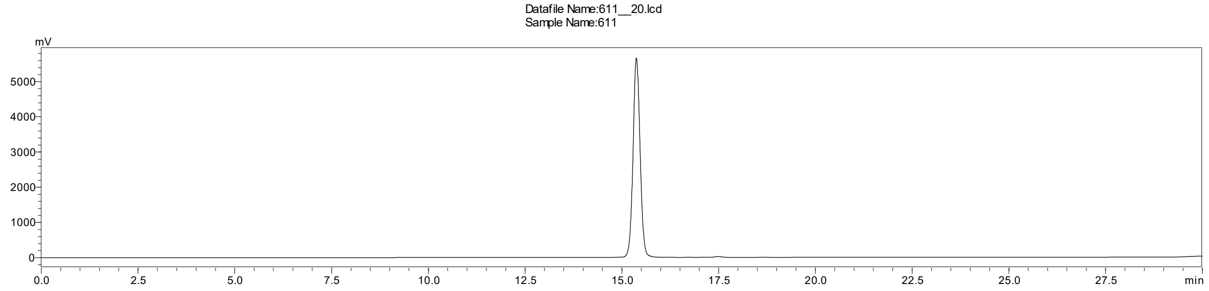

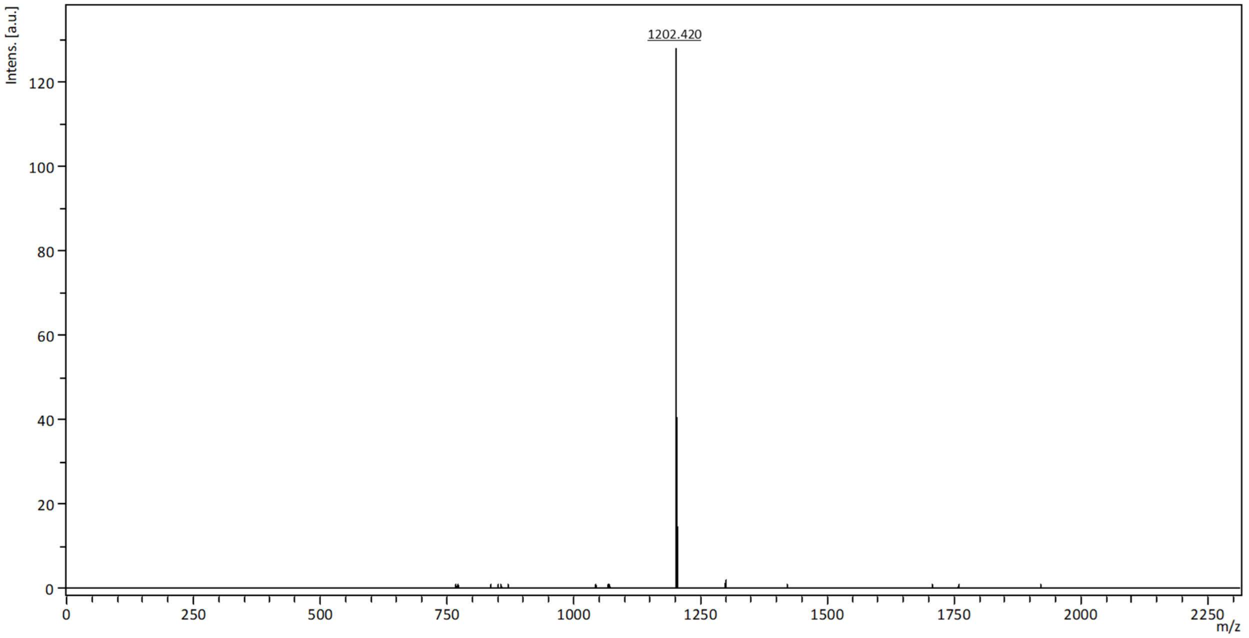

Fucα1-2Galβ1-4(Fucα1-3)GlcNAcβ1-6GalNAcα-Ser-Fmoc (97)

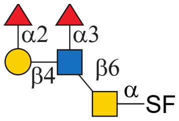

Compound **97** was prepared according to general procedure of α1-3 fucosylation with Hp3FT. After lyophilization, **97** was obtained as white solid. Compound was characterized by HPLC, T_R =14.510 min. ^{1}H NMR (600 MHz, D_2O) δ 7.94 – 7.79 (m, 2H), 7.75 – 7.58 (m, 2H), 7.53 – 7.36 (m, 4H), 5.26 (d, *J* = 3.4 Hz, 1H), 5.09 (d, *J* = 3.6 Hz, 1H), 4.68 (s, 1H), 4.53 (d, *J* = 8.2 Hz, 1H), 4.41 (d, *J* = 7.7 Hz, 1H), 4.31 (m, 2H), 4.24 – 4.16 (m, 1H), 4.07 (d, *J* = 11.7 Hz, 1H), 4.02 – 3.59 (m, 24H), 3.59 – 3.34 (m, 3H), 1.99 (s, 3H), 1.95 (s, 3H), 1.24 (t, *J* = 7.7 Hz, 6H). HRMS, $C_{52}H_{73}N_3O_{28}$, Calcd for: 1187.4381; found $[M-H]^-$ 1187.346.

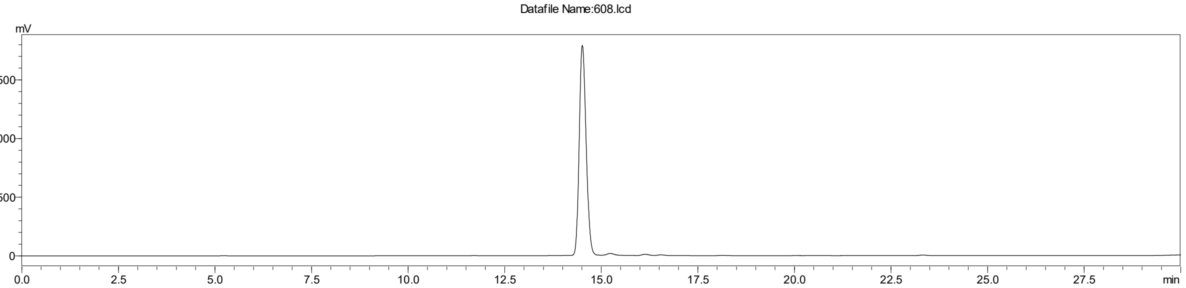

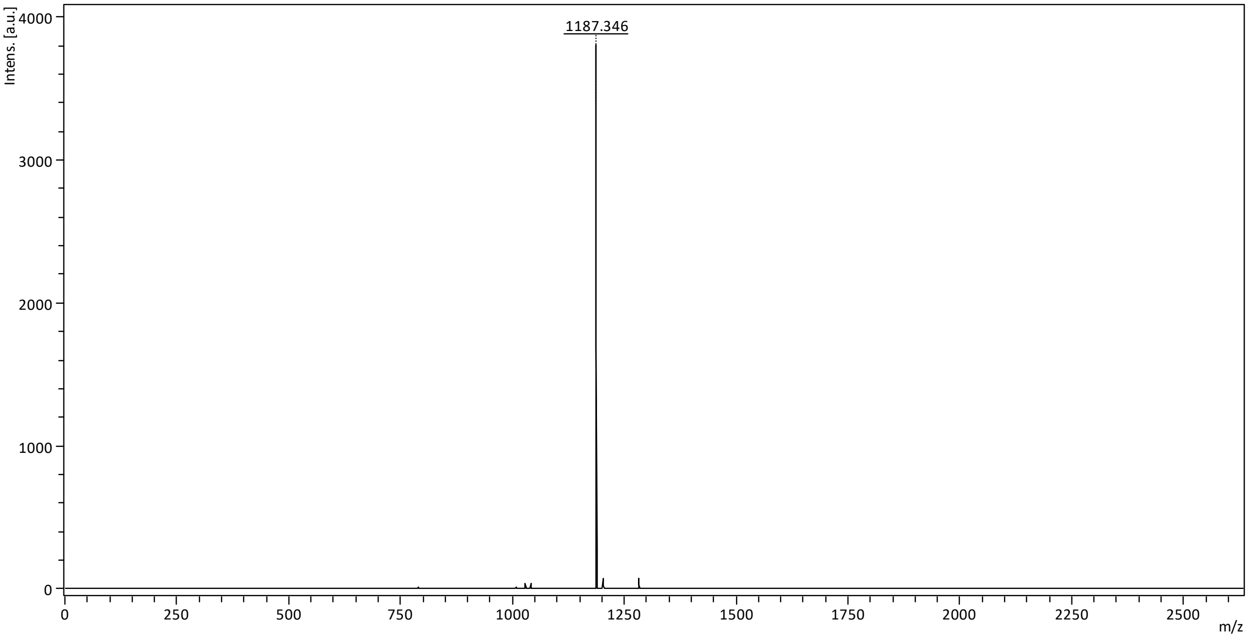

Galα1-3Galβ1-4GlcNAcβ1-6GalNAcα-Ser-Fmoc (98)

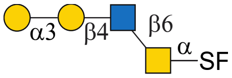

Compound **98** was prepared according to general procedure of α1-3 galactosylation with α3GalT. After lyophilization, **98** was obtained as white solid. Compound was characterized by HPLC, T_R =16.891 min. ^{1}H NMR (600 MHz, D_2O) δ 7.85 – 7.74 (m, 2H), 7.70 – 7.51 (m, 2H), 7.48 - 7.30 (m, 4H), 5.14 (d, *J* = 3.9 Hz, 1H), 4.50 (d, *J* = 8.2 Hz, 2H), 4.45 (d, *J* = 7.8 Hz, 1H), 4.33 (s, 1H), 4.20 (d, *J* = 6.4 Hz, 1H), 4.18 (d, *J* = 7.6 Hz, 2H), 4.10 – 4.01 (m, 2H), 3.96 (d, *J* = 3.3 Hz, 1H), 3.94 (d, *J* = 3.3 Hz, 1H), 3.94 – 3.84 (m, 4H), 3.83 3.62 (m, 17H), 1.95 (s, 3H), 1.94 (s, 3H). HRMS, $C_{46}H_{63}N_3O_{25}$, Calcd for: 1057.3751; found $[M+H]^+$ 1058.3741.

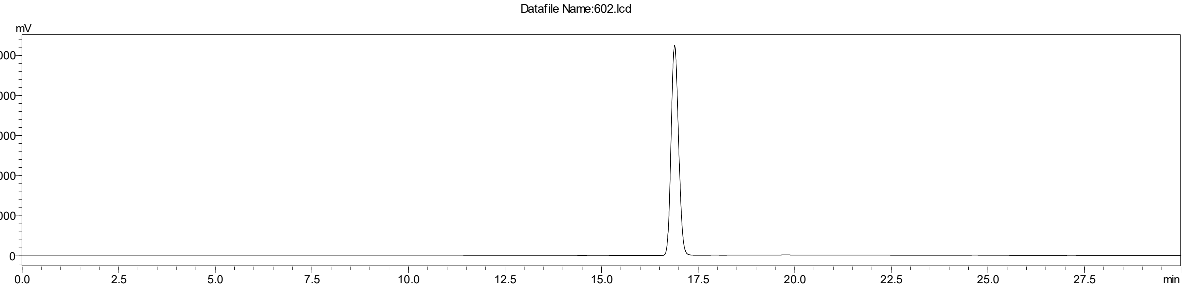

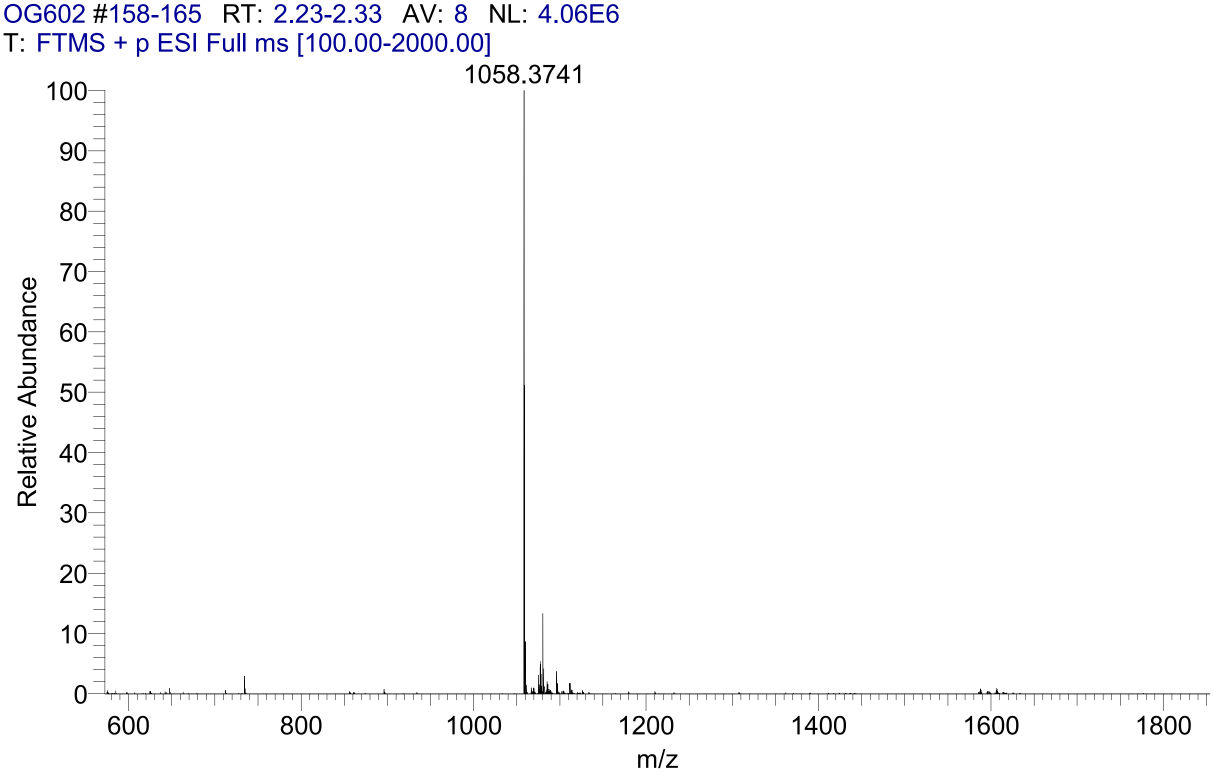

Galβ1-4(Fucα1-3)GlcNAcβ1-6GalNAcα-Ser-Fmoc (99)

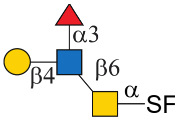

Compound **99** was prepared according to general procedure of α1-3 fucosylation with Hp3FT. After lyophilization, **99** was obtained as white solid. Compound was characterized by HPLC, T_R =15.232 min. ^{1}H NMR (600 MHz, D_2O) δ 7.95 – 7.85 (m, 2H), 7.78 – 7.60 (m, 2H), 7.56 - 7.38 (m, 4H), 5.09 (d, *J* = 3.2 Hz, 1H), 4.64 (m, 1H), 4.53 (d, *J* = 8.0 Hz, 1H), 4.38 (d, *J* = 7.5 Hz, 1H), 4.33 (m, 2H), 4.10 – 3.46 (m, 28 H), 3.37 (t, *J* = 8.64 Hz, 1H), 1.96 (s, 3H), 1.94 (s, 3H), 1.18 (d, *J* = 6.5 Hz, 1H). HRMS, $C_{46}H_{63}N_3O_{24}$, Calcd for: 1041.3801; found $[M-H]^-$ 1040.3856.

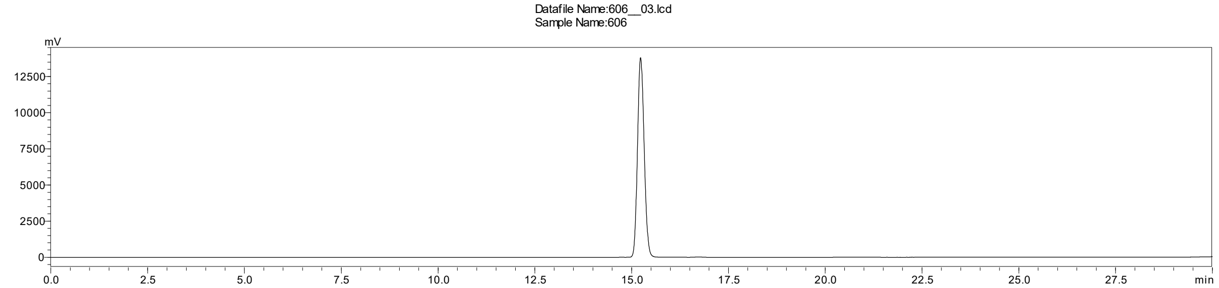

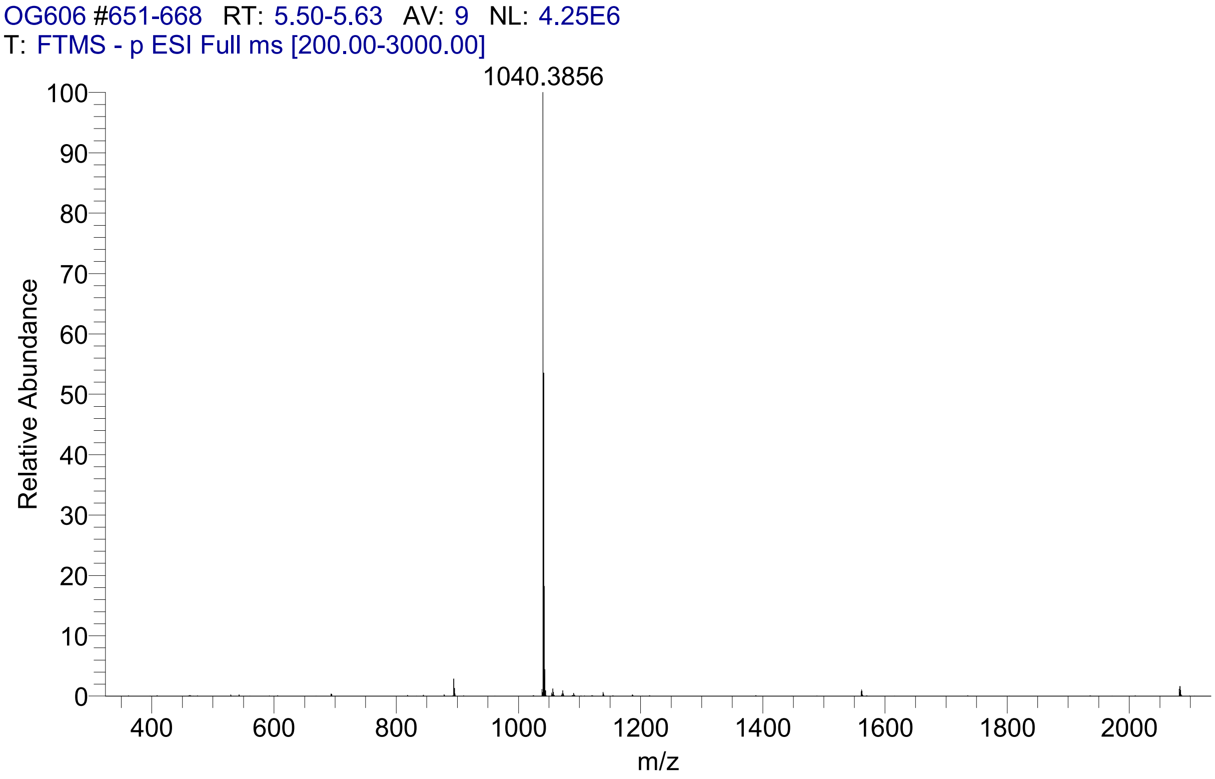

Neu5Acα2-3Galβ1-4(Fucα1-3)GlcNAcβ1-6GalNAcα-Ser-Fmoc (100)

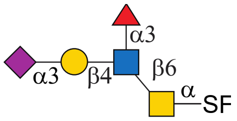

Compound **100** was prepared according to general procedure of α2-3 sialylation with PmST1-M144D. After lyophilization, **100** was obtained as white solid. Compound was characterized by HPLC, T_R =13.724 min. ^{1}H NMR (600 MHz, D_2O) δ 7.96 – 7.87 (m, 2H), 7.78 – 7.63 (m, 2H), 7.56 - 7.40 (m, 4H), 5.09 (d, *J* = 3.6 Hz, 1H), 4.65 (s, 1H), 4.52 (d, *J* = 8.3 Hz, 2H), 4.43 (d, *J* = 7.7 Hz, 1H), 4.36 (s, 1H), 4.31 (s, 1H), 4.08 (s, 2H), 3.99 – 3.48 (m, 31H), 2.78 (dd, *J* = 12.4, 4.6 Hz, 1H), 2.05 (s, 3H), 1.96 (s, 3H), 1.82 (t, *J* = 12.2 Hz, 1H), 1.17 (d, *J* = 6.5 Hz, 3H). HRMS, $C_{57}H_{80}N_4O_{32}$, Calcd for: 1332.4756; found $[M-H]^-$ 1331.486.

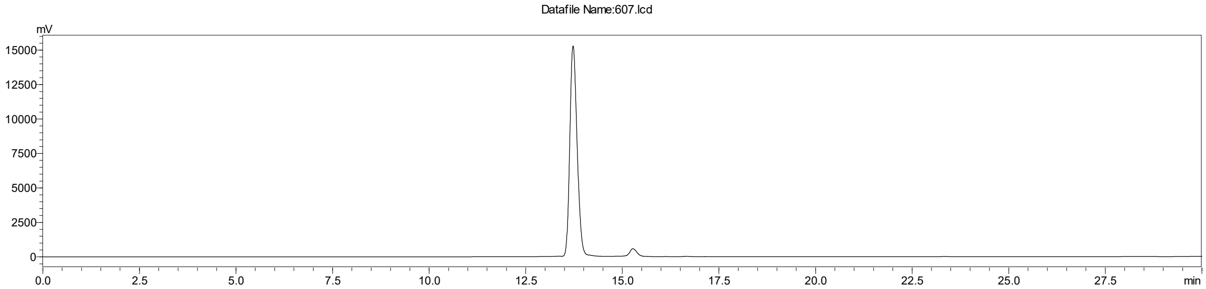

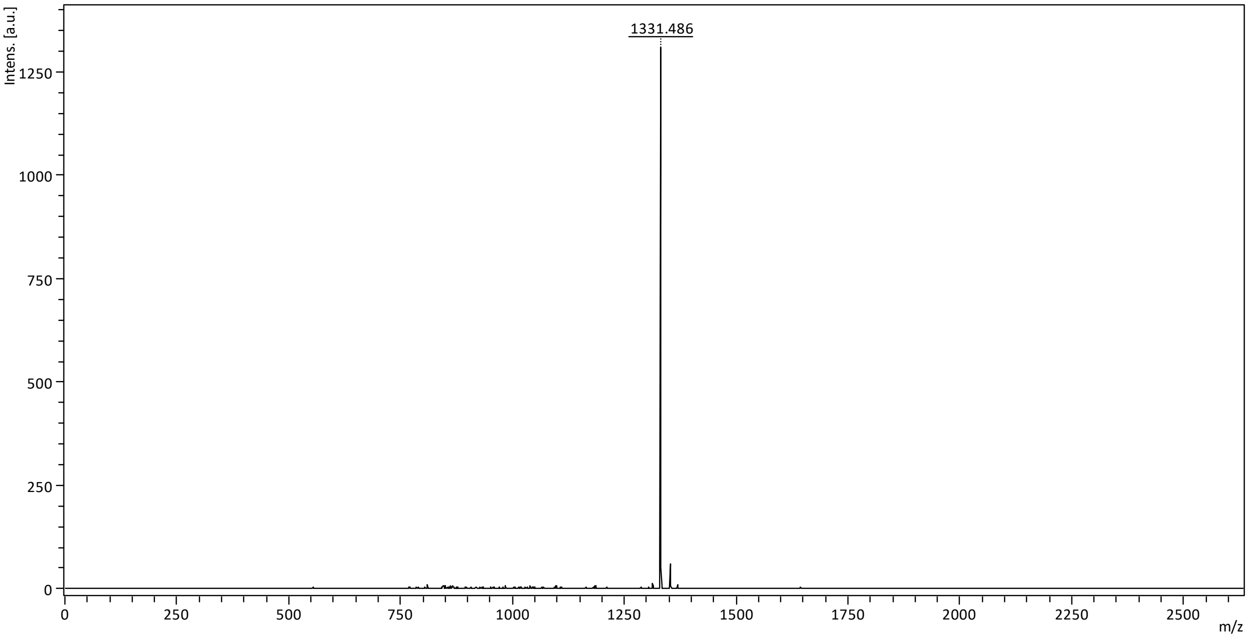

GalNAcβ1-4GlcNAcβ1-6GalNAcα-Ser-Fmoc (101)

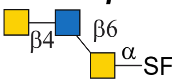

Compound **101** was prepared according to general procedure of β1-4-N-acetylgalatosaminylation with NmLgtBm. After lyophilization, **101** was obtained as white solid. Compound was characterized by HPLC, T_R =16.843 min. ^{1}H NMR (600 MHz, D_2O) δ 7.91 – 7.73 (m, 2H), 7.72 – 7.57 (m, 2H), 7.49 – 7.31 (m, 4H), 4.66 (s, 1H), 4.58 (d, J = 6.0 Hz, 1H), 4.46 – 4.35 (m, 2H), 4.30 (s, 1H), 4.24 (s, 1H), 4.01 (d, J = 12.1 Hz, 1H), 3.93 – 3.80 (m, 5H), 3.80 – 3.54 (m, 12H), 3.54 – 3.40 (m, 2H), 3.35 (s, 1H), 3.20 (s, 1H), 2.01 (s, 3H), 1.95 (s, 3H), 1.89 (s, 3H). HRMS, $C_{42}H_{56}N_4O_{20}$, Calcd for: 936.3488; found $[M-H]^-$ 935.362.

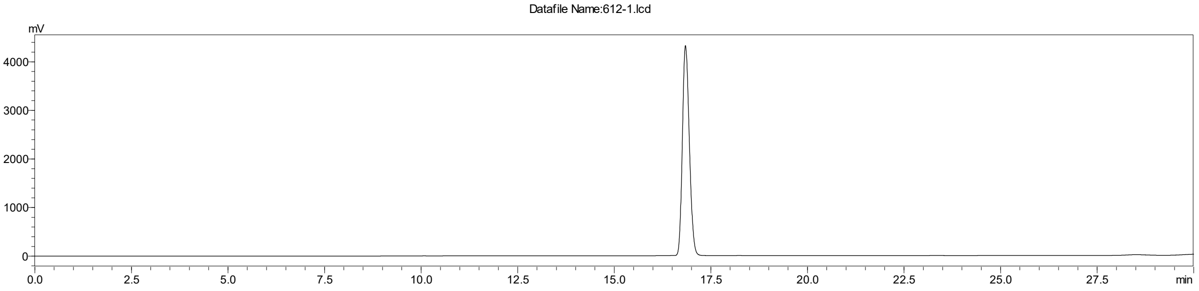

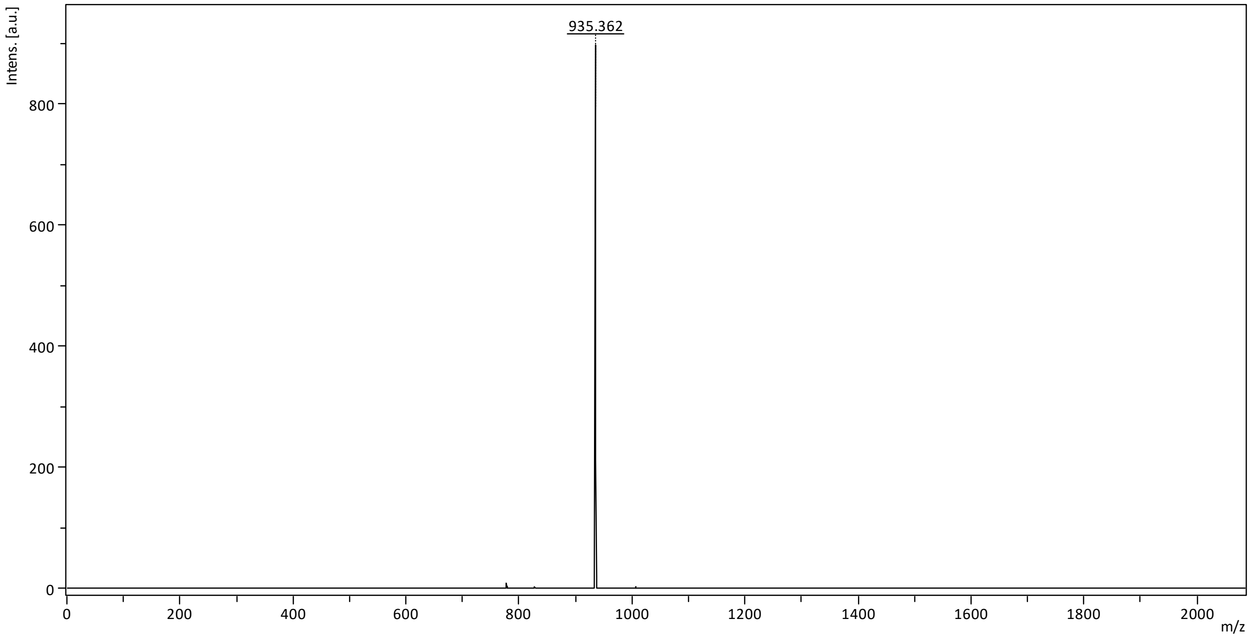

VI. NMR Spectra

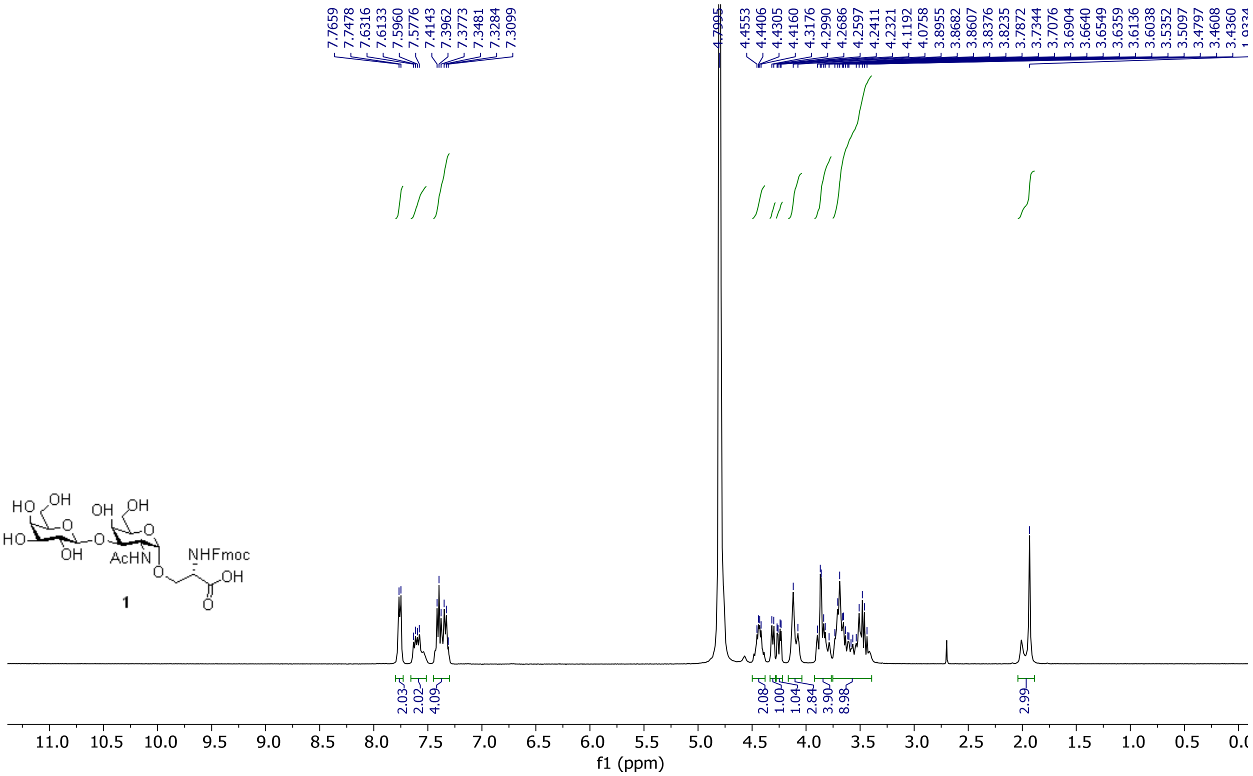

^{1}H NMR of 1

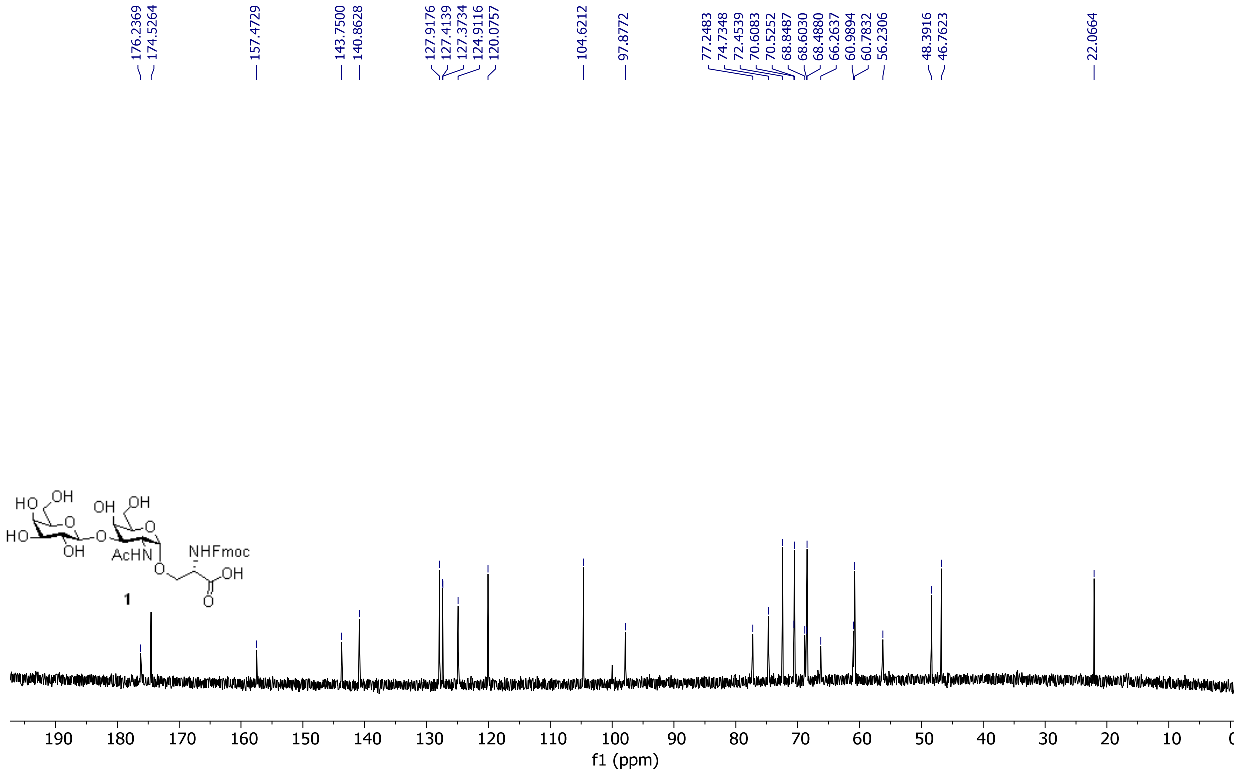

^{13}C NMR of 1

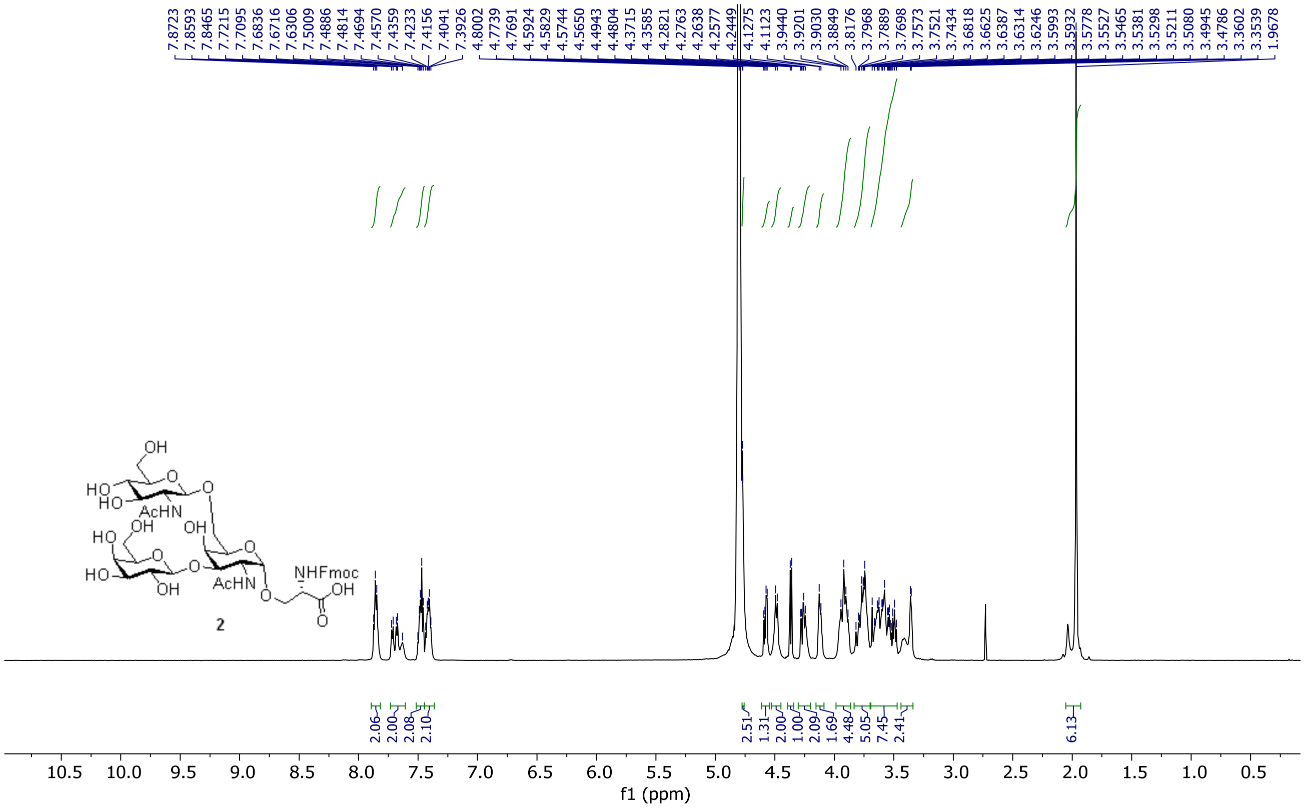

^{1}H NMR of 2

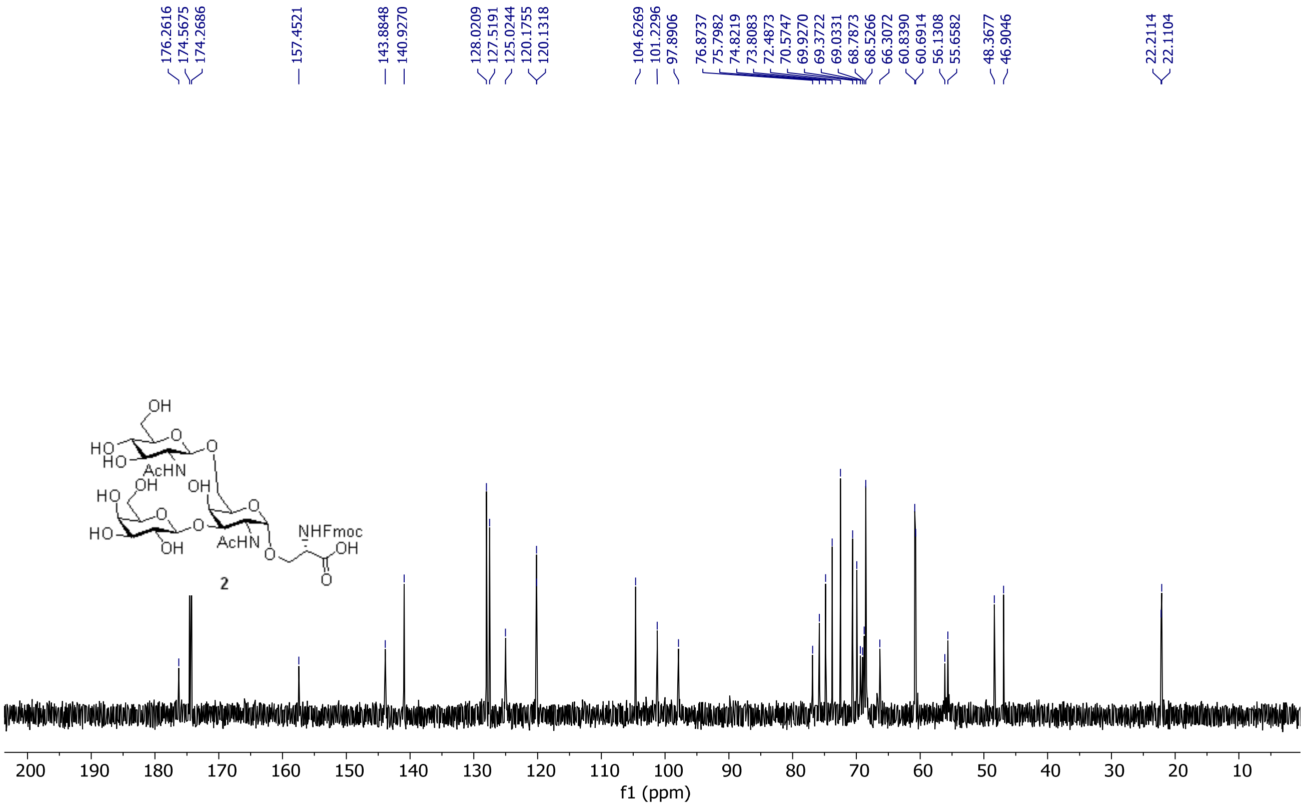

^{13}C NMR of 2

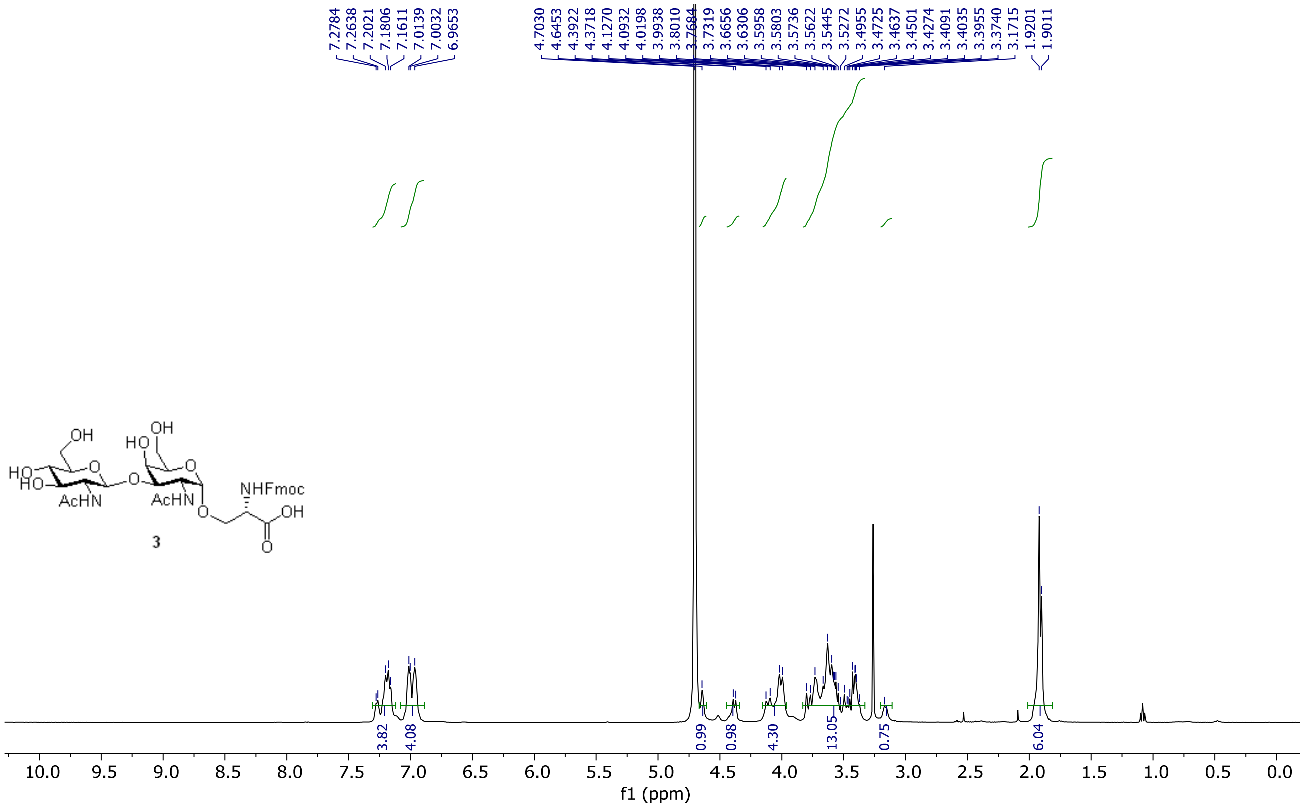

^{1}H NMR of 3

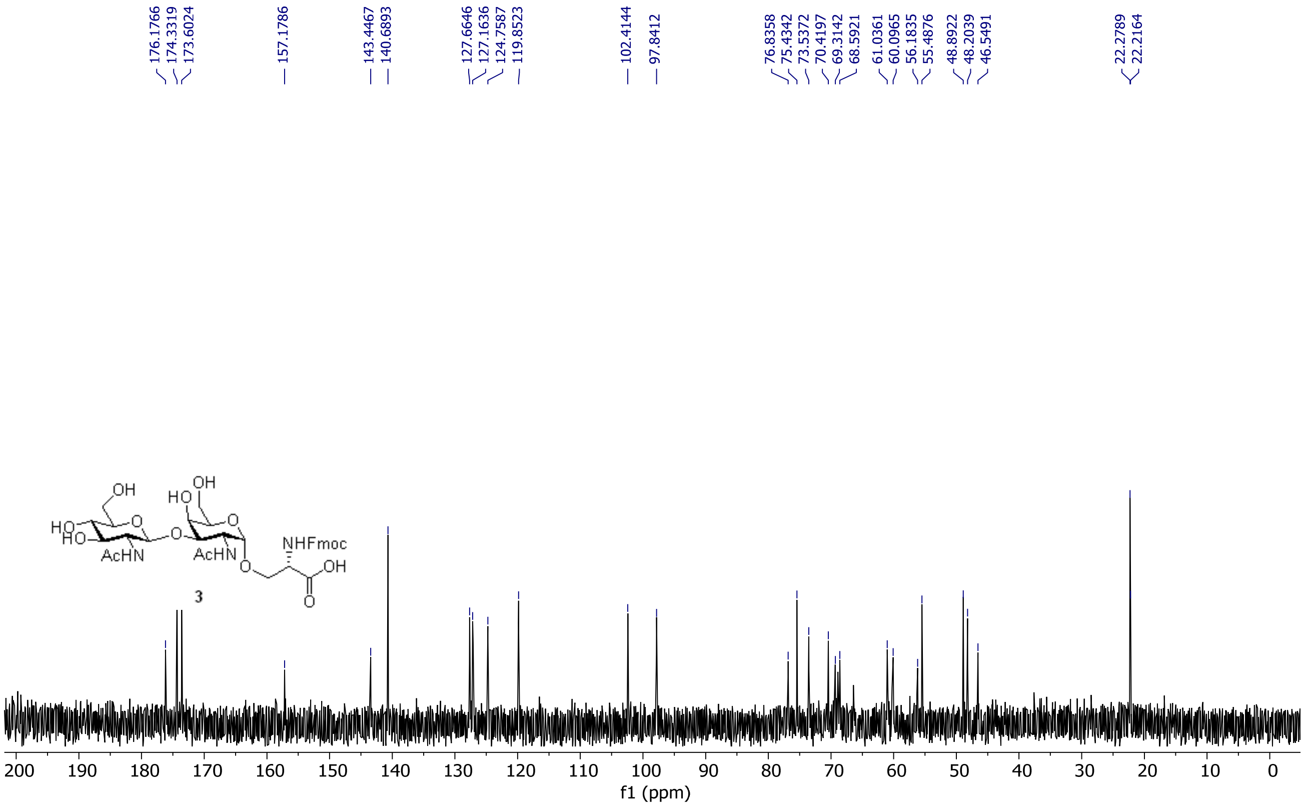

^{13}C NMR of 3

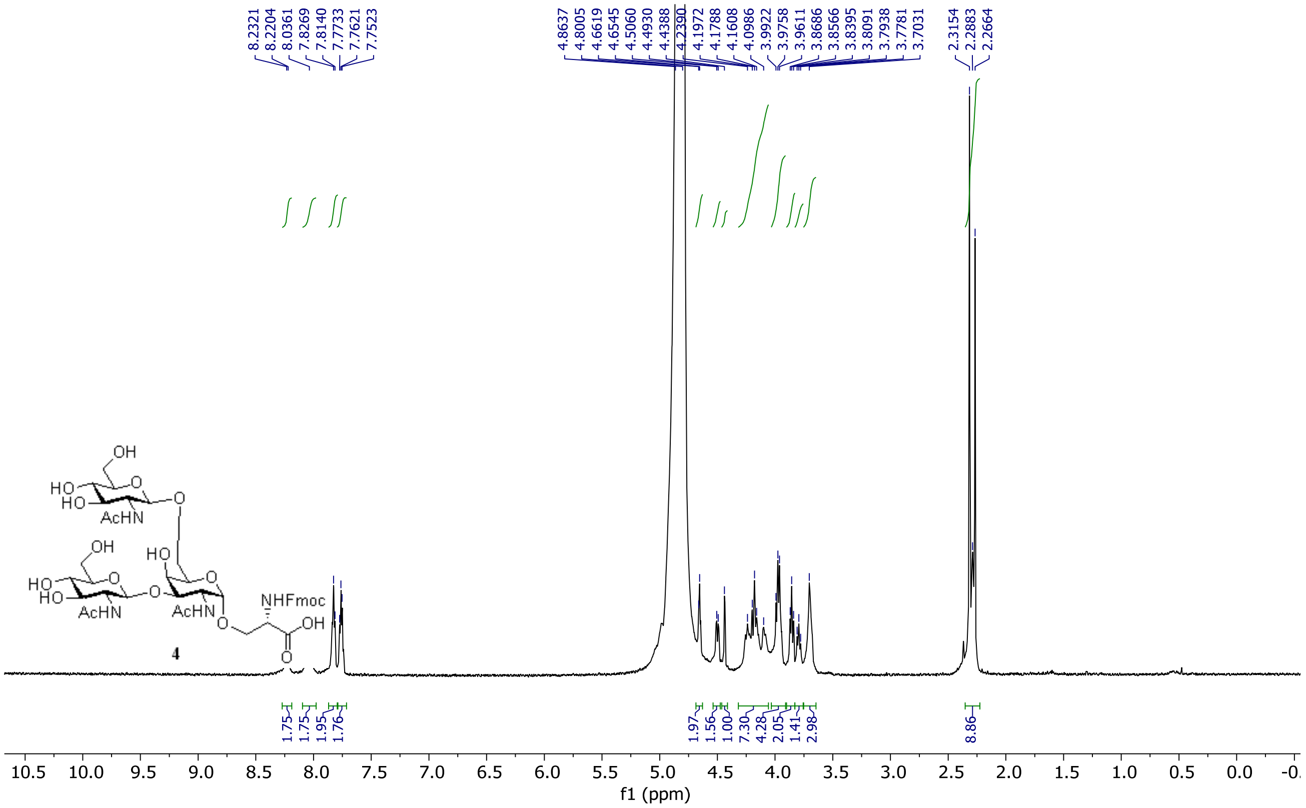

^{1}H NMR of 4

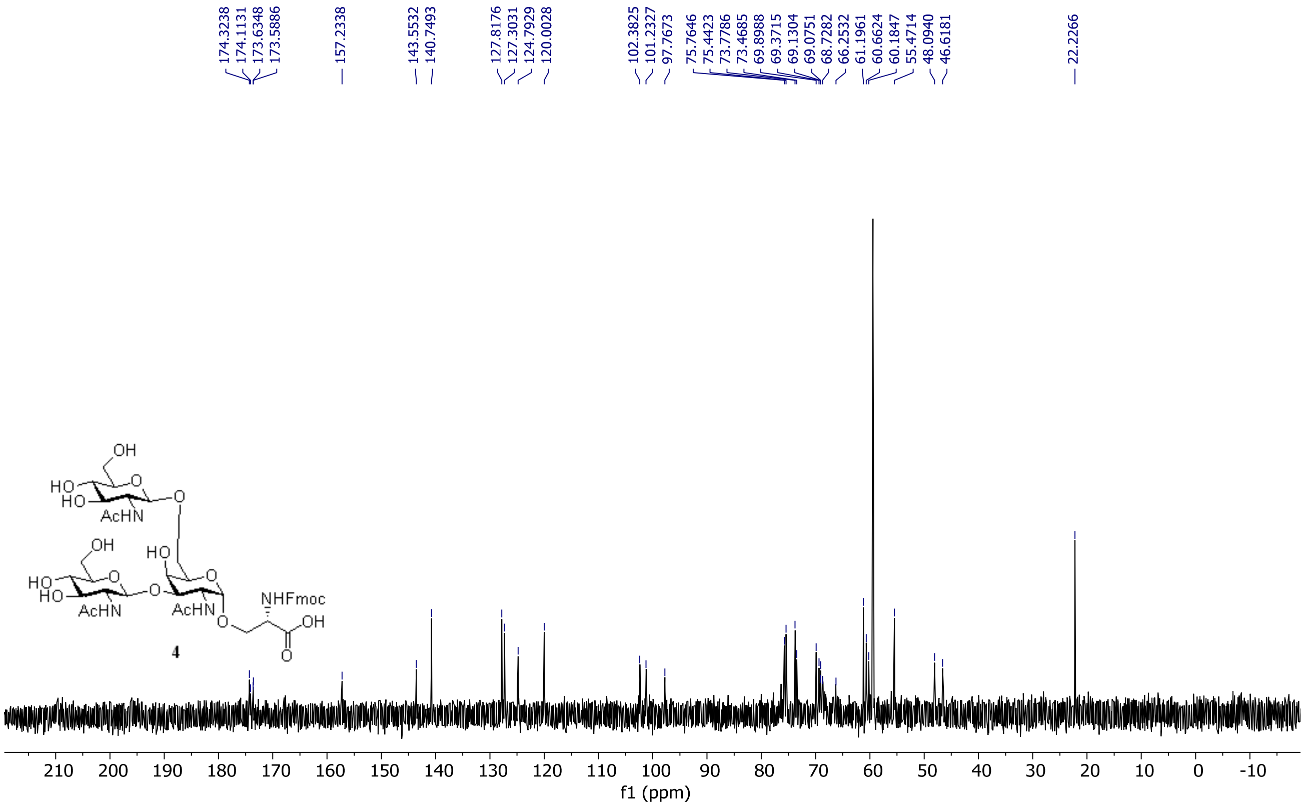

^{13}C NMR of 4

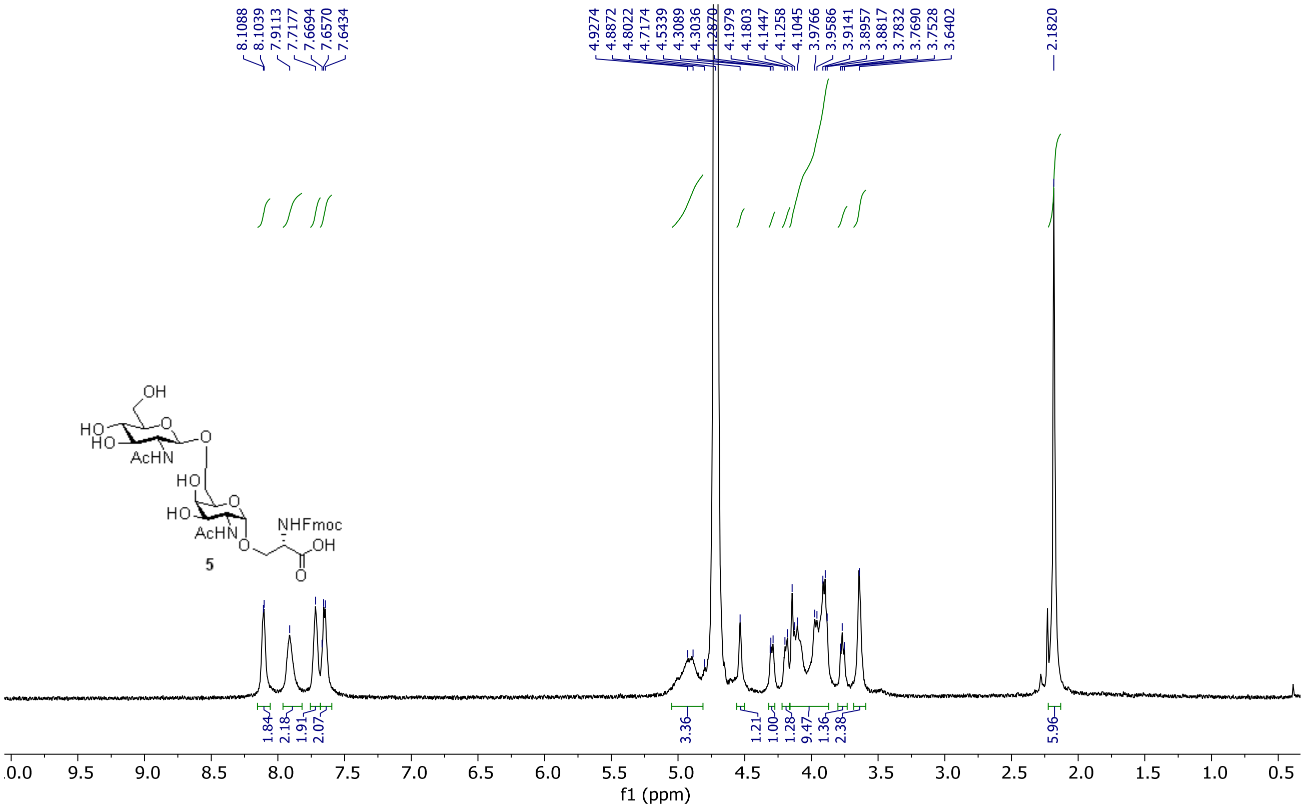

¹H NMR of 5

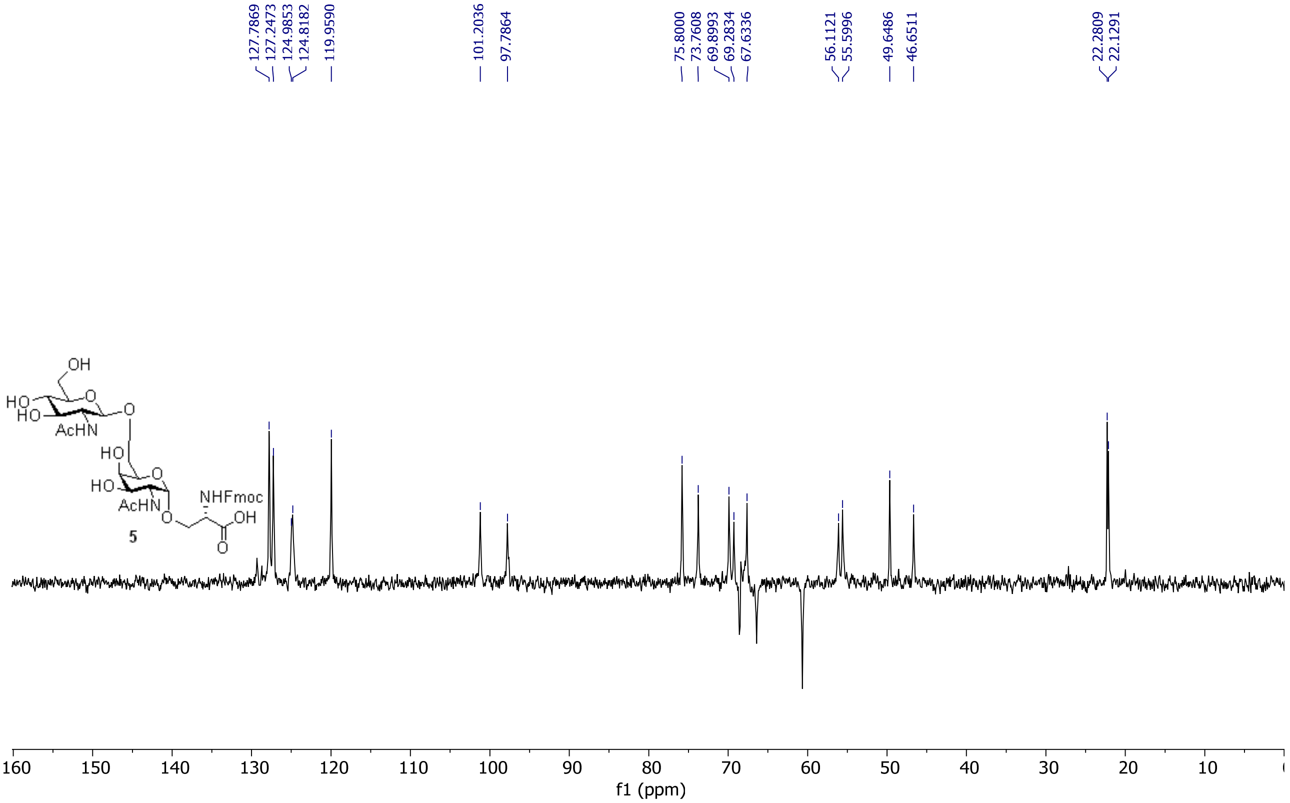

¹³C NMR of 5

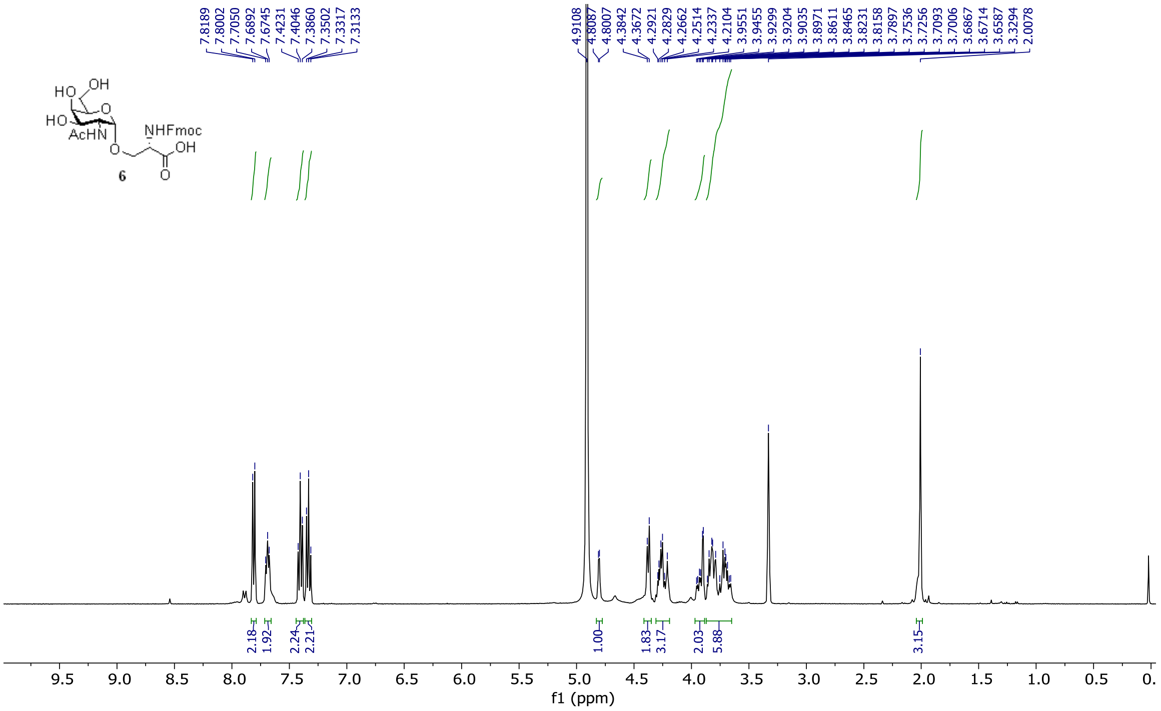

^{1}H NMR of 6

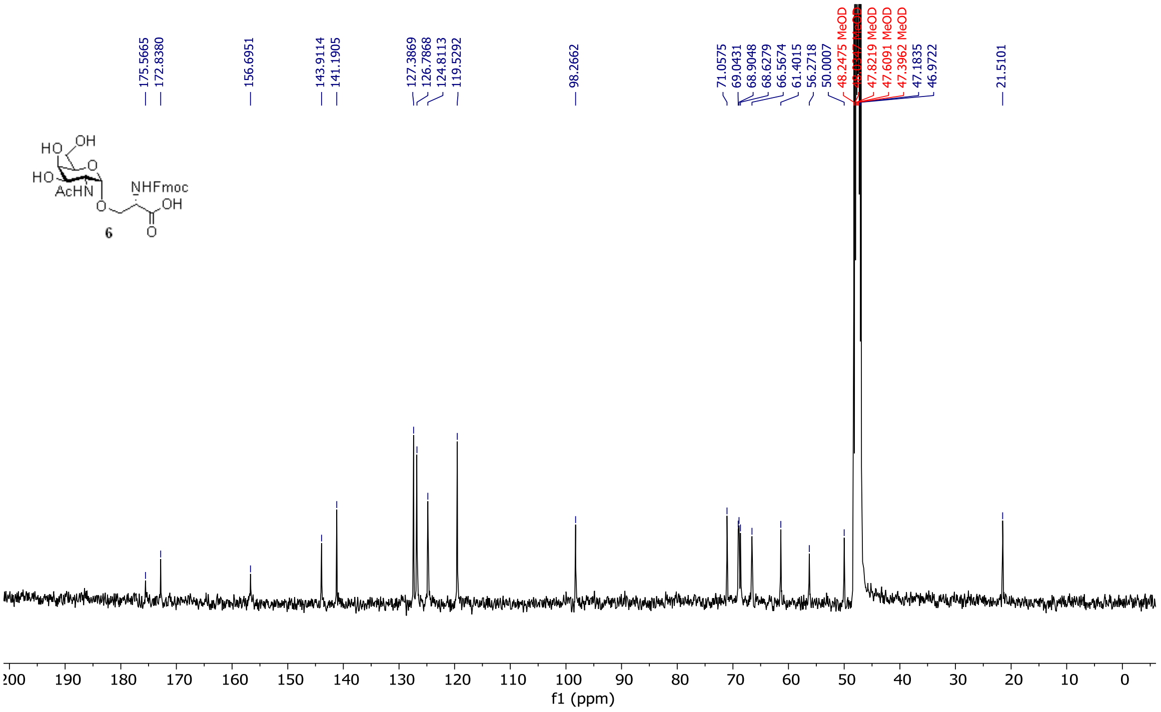

^{13}C NMR of 6

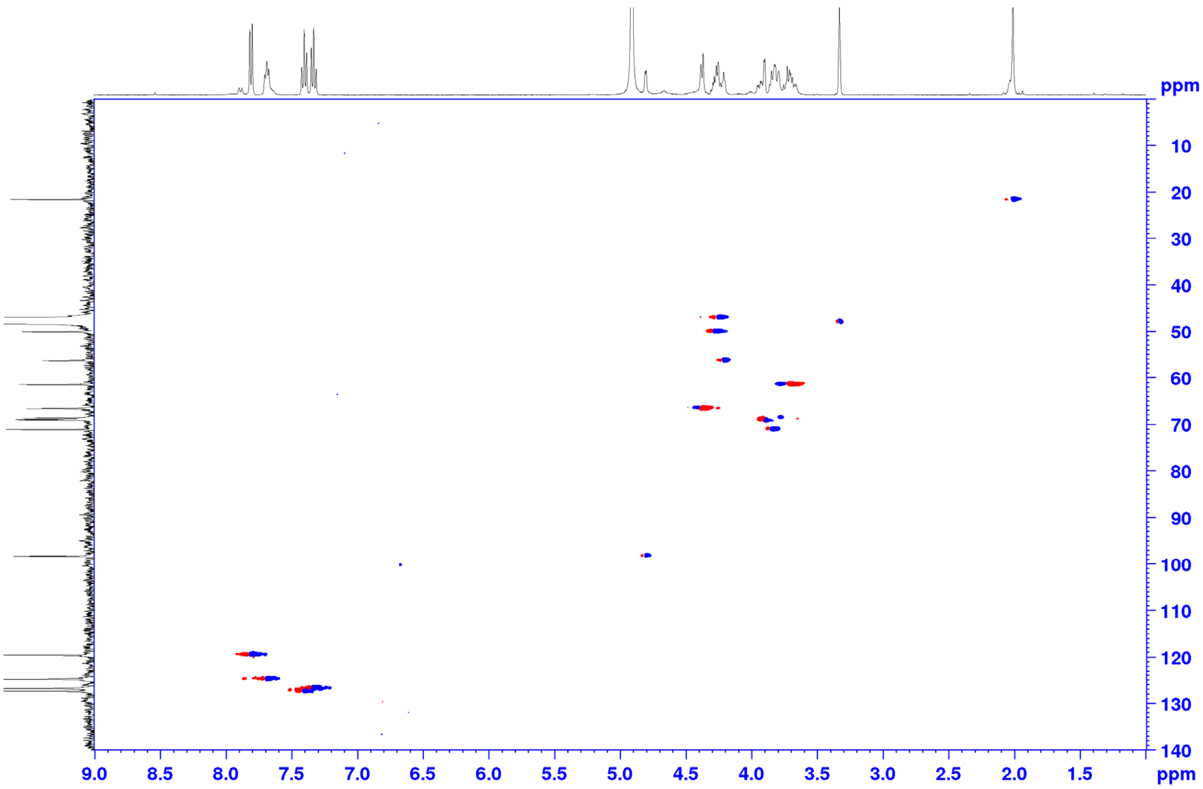

HSQC of 6

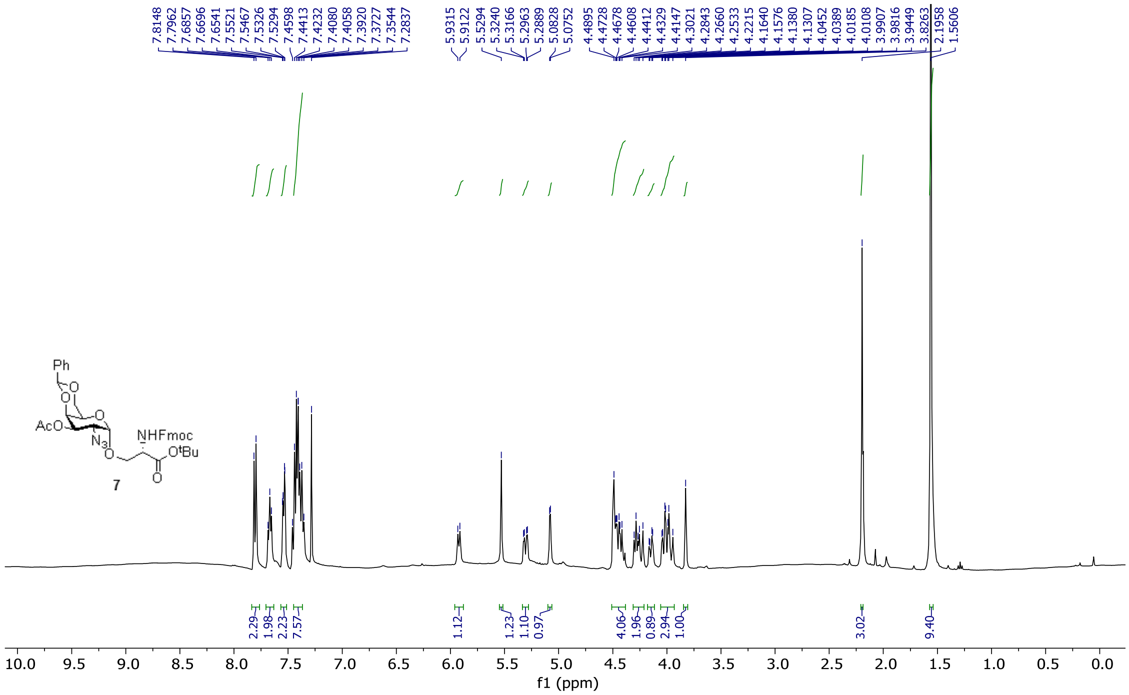

^{1}H NMR of 7

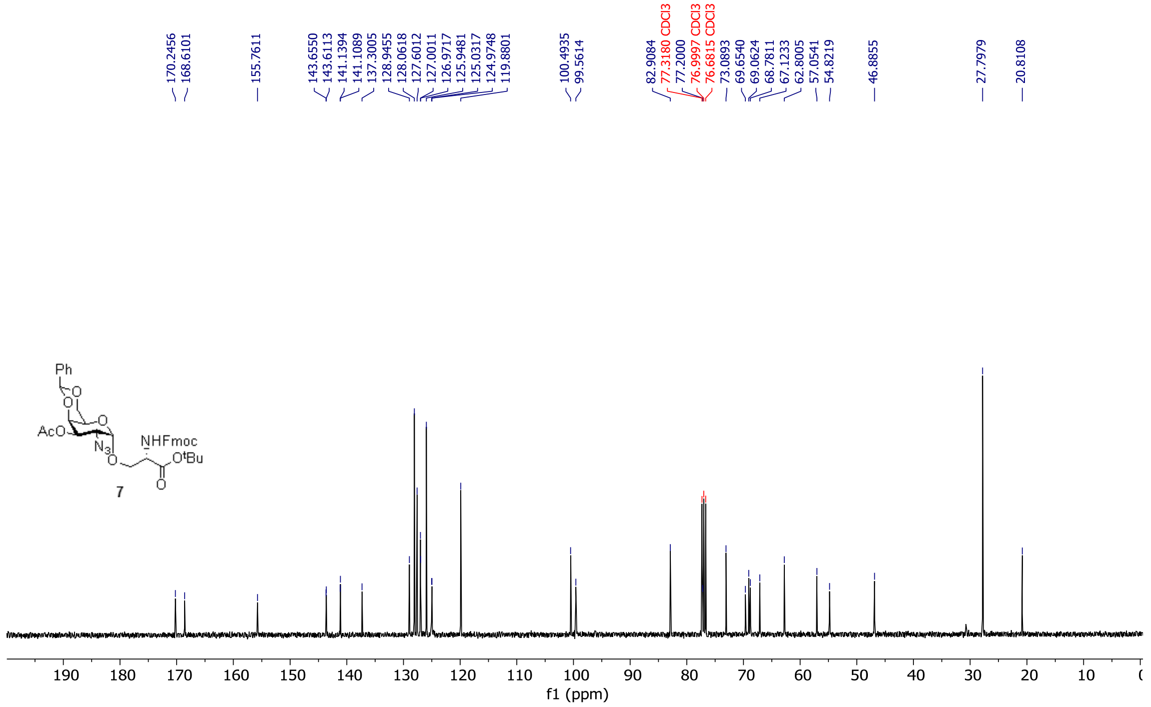

^{13}C NMR of 7

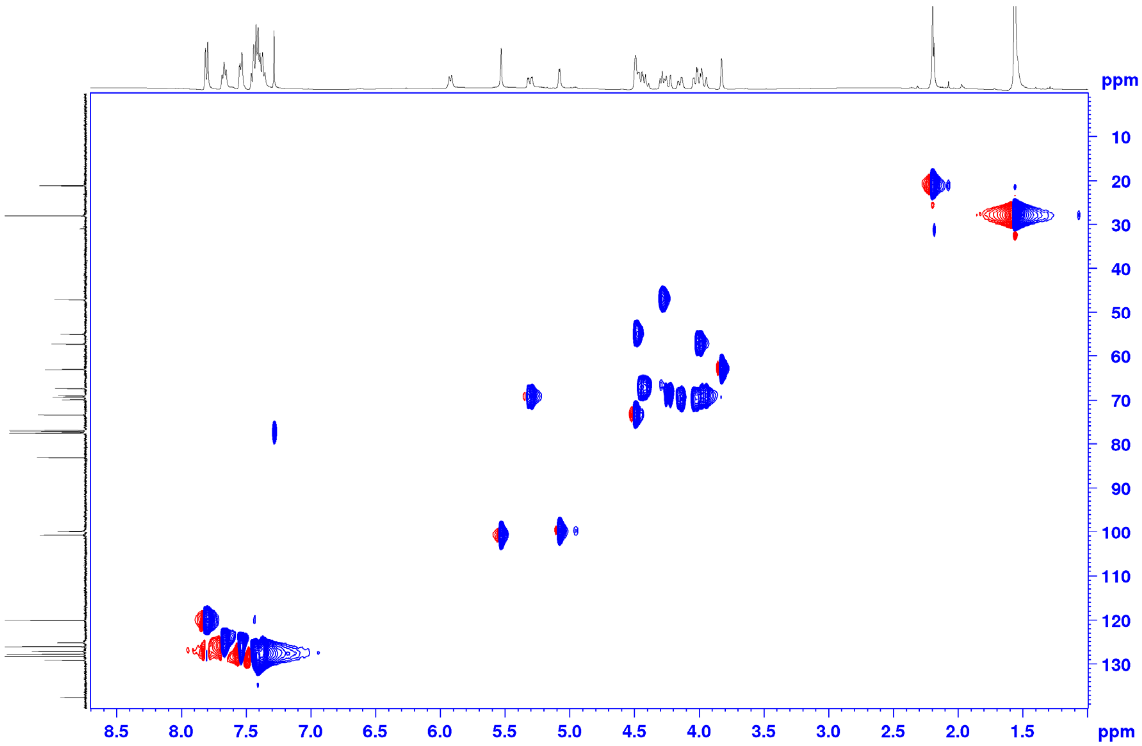

HSQC of 7

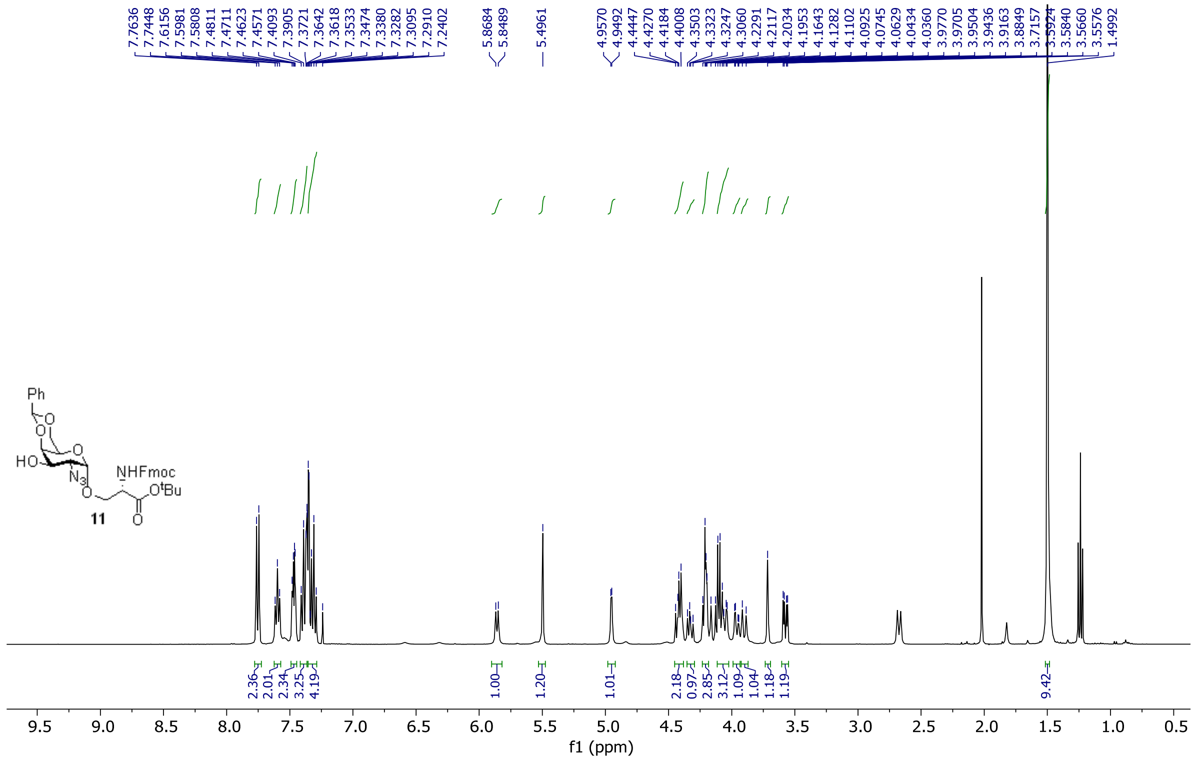

^{1}H NMR of 11

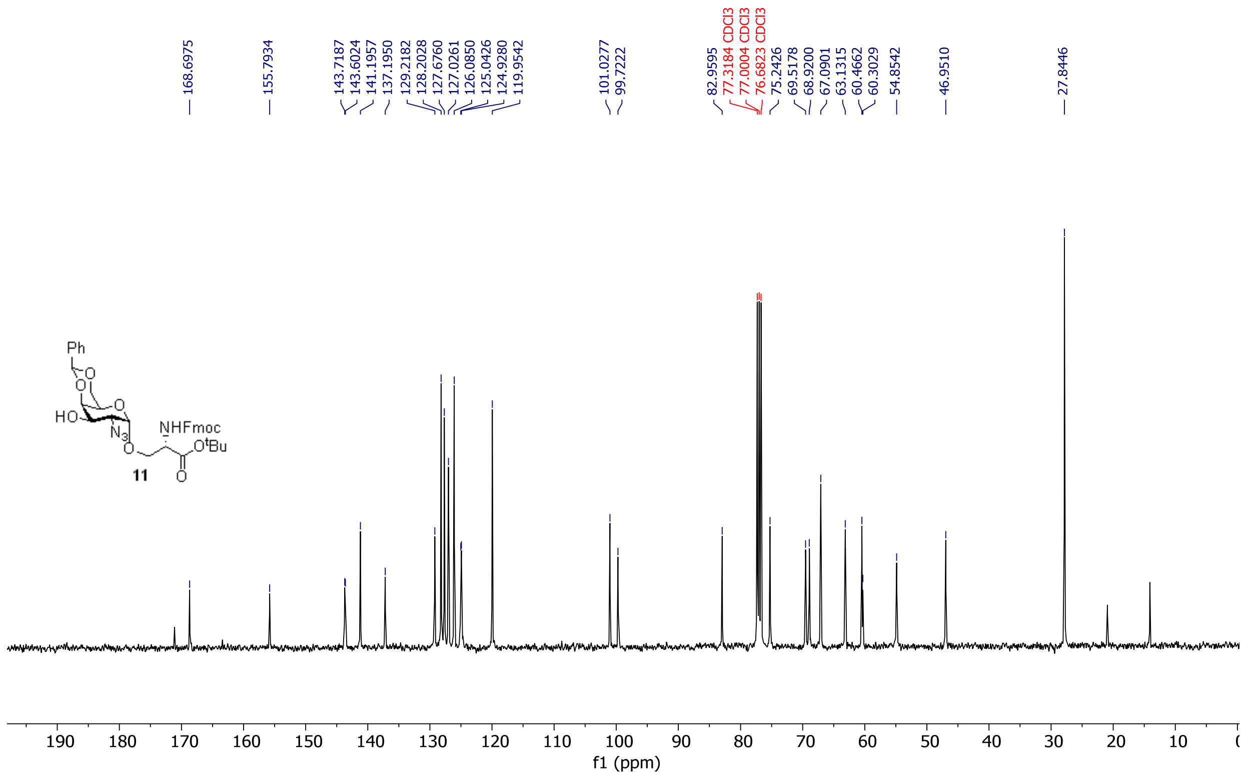

^{13}C NMR of 11

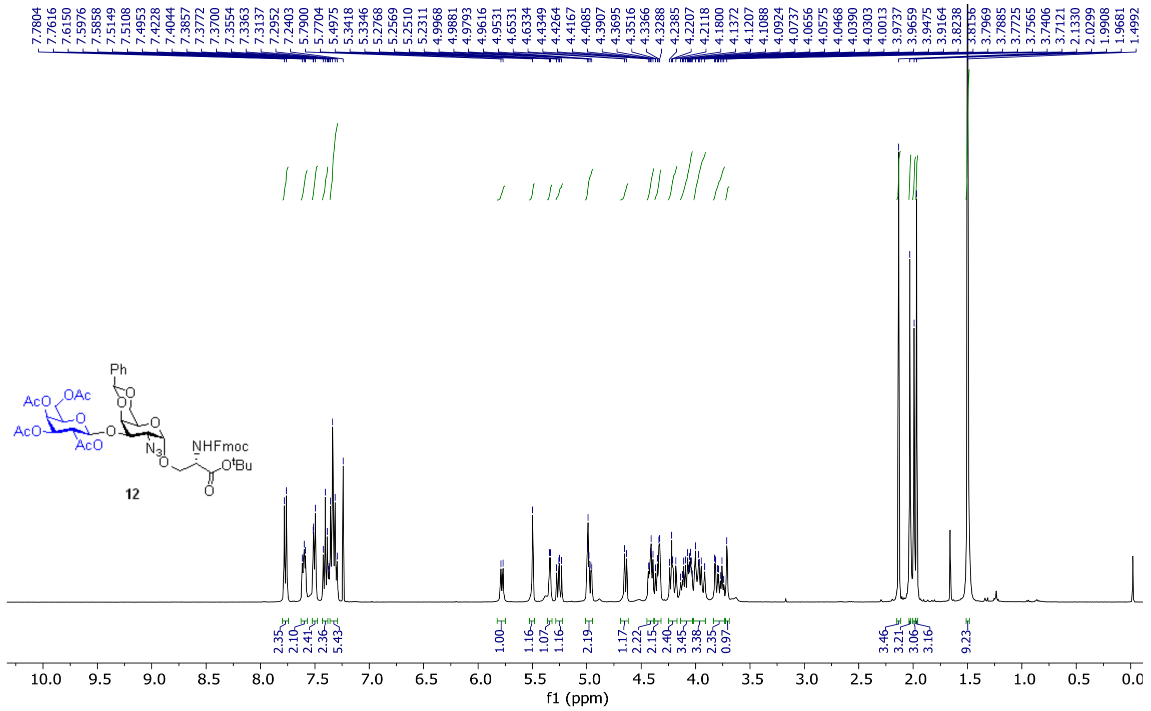

^{1}H NMR of 12

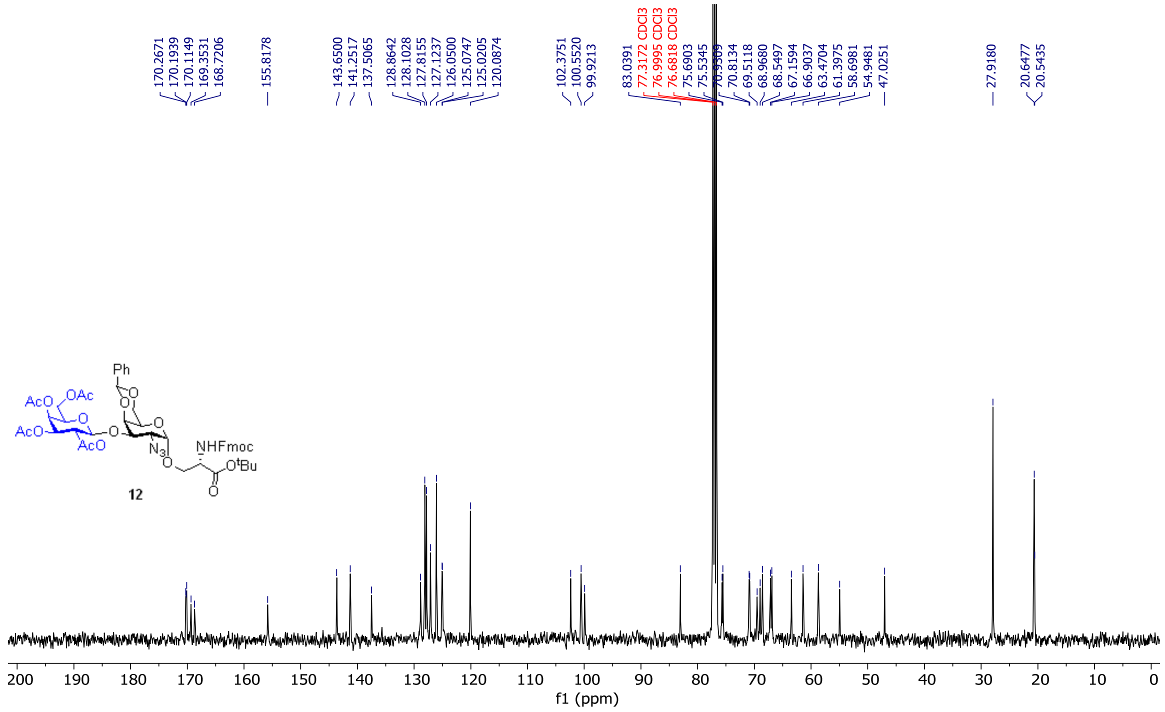

^{13}C NMR of 12

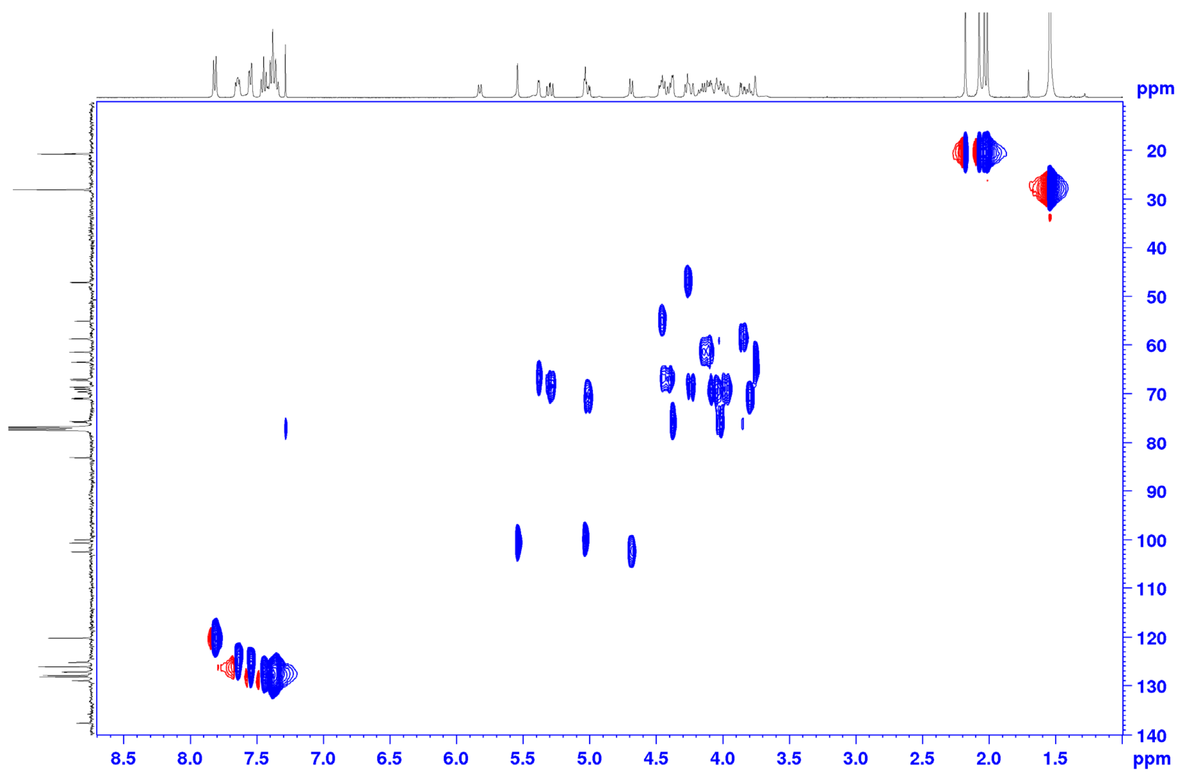

HSQC of 12

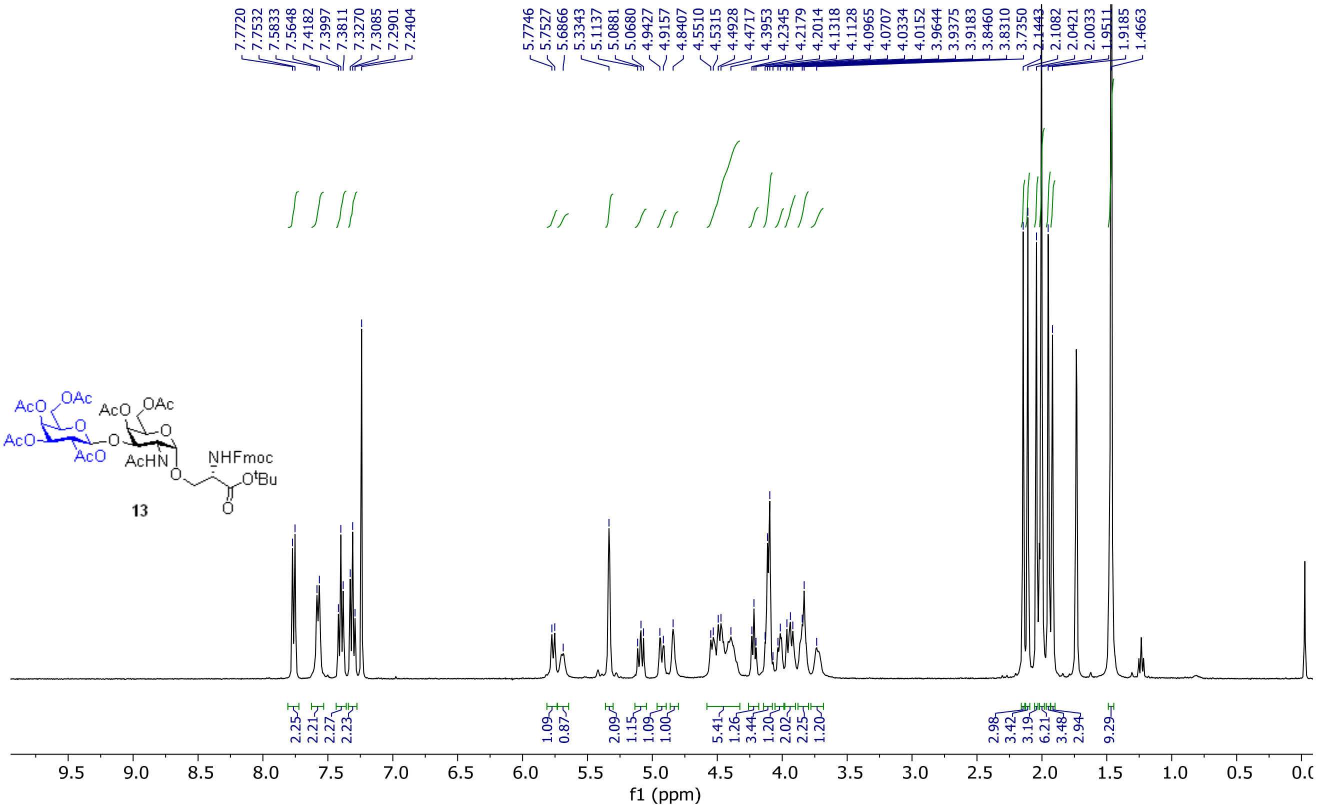

^{1}H NMR of 13

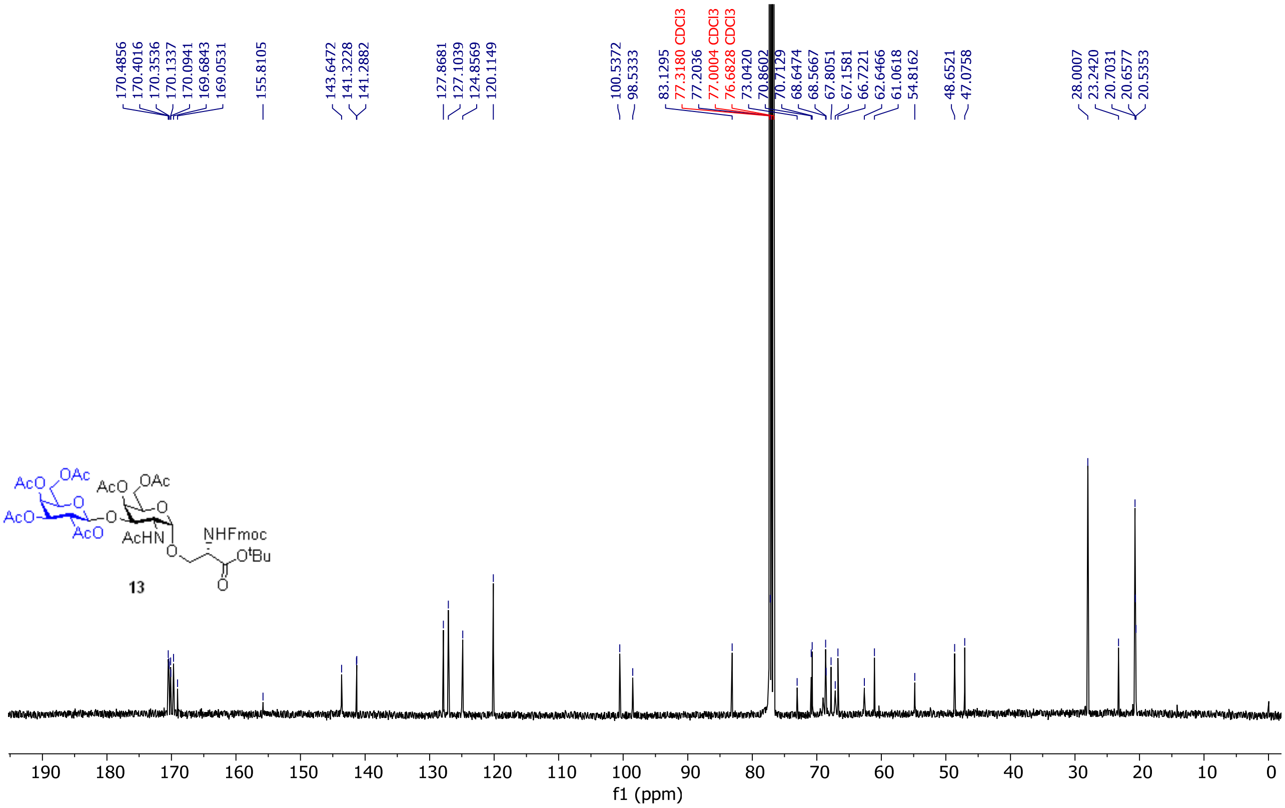

^{13}C NMR of 13

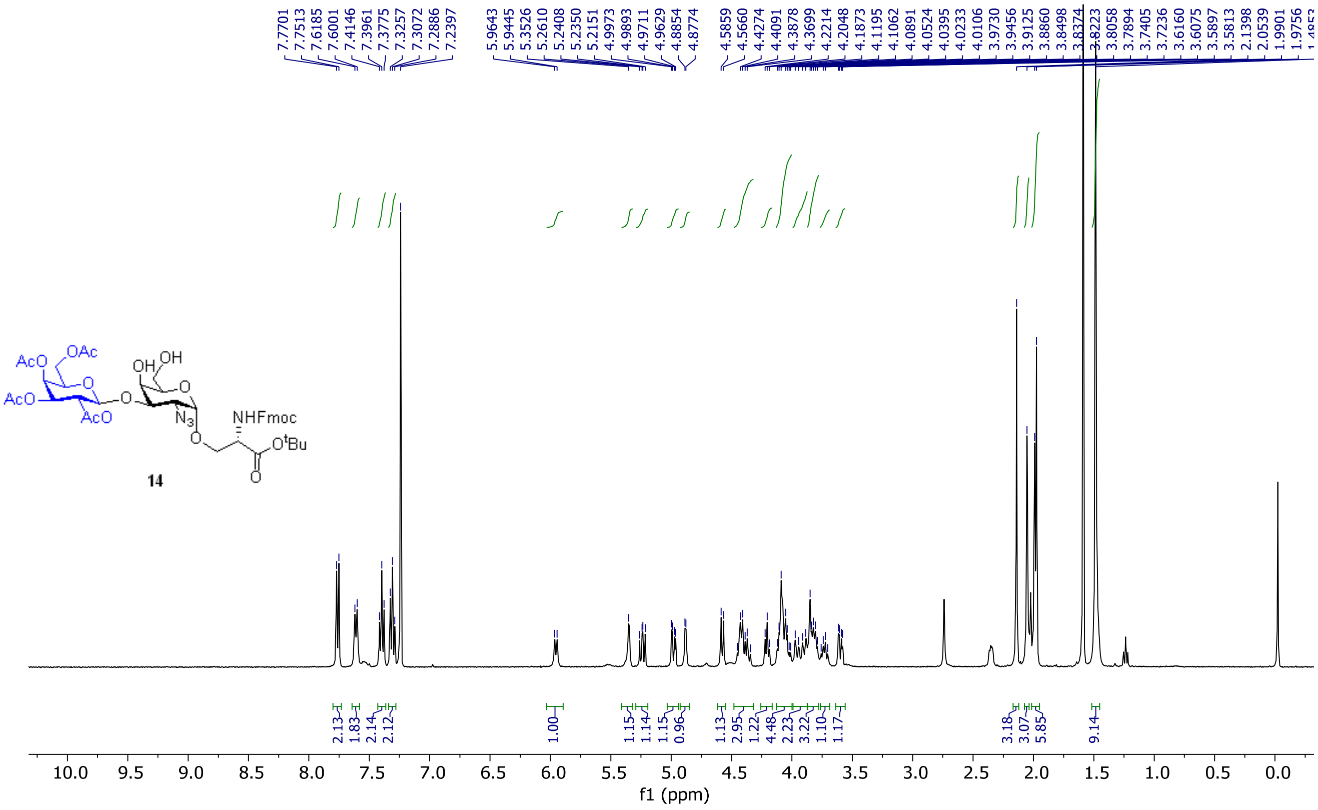

^{1}H NMR of 14

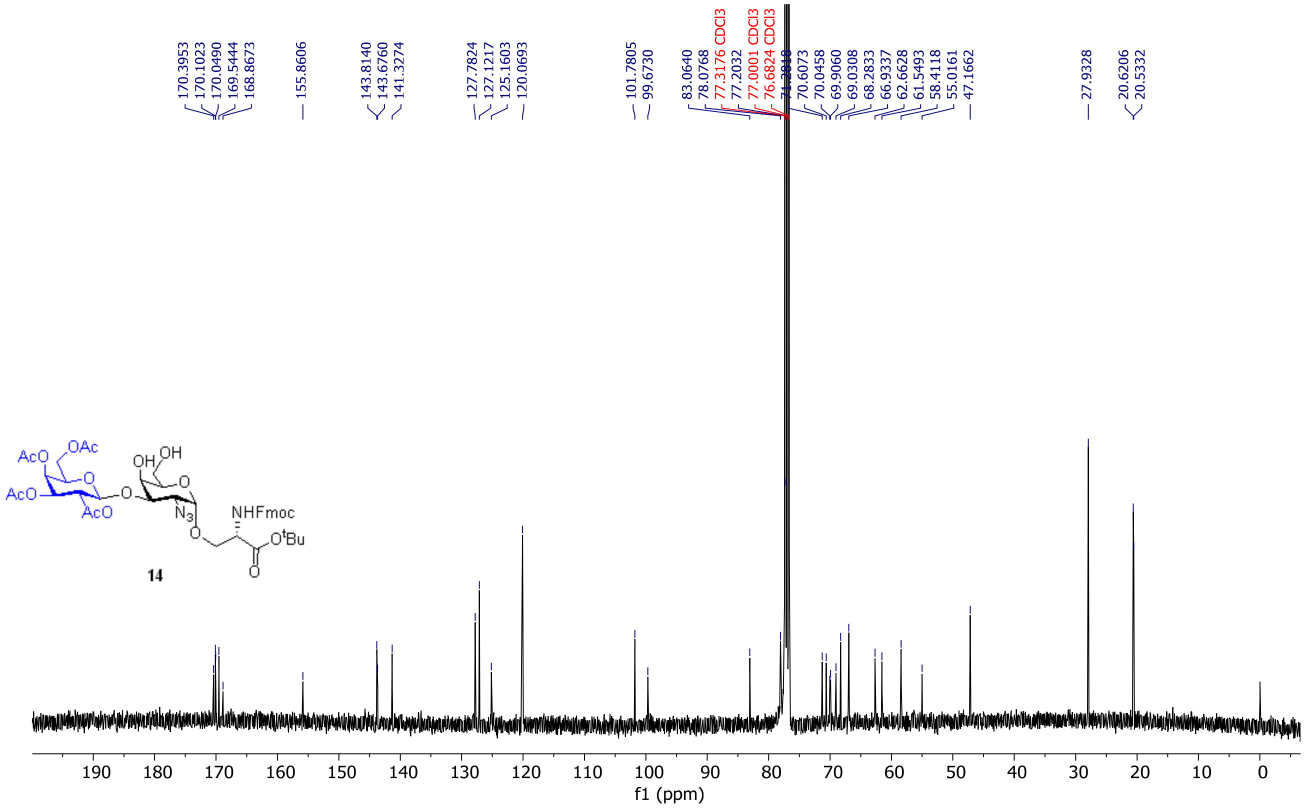

^{13}C NMR of 14

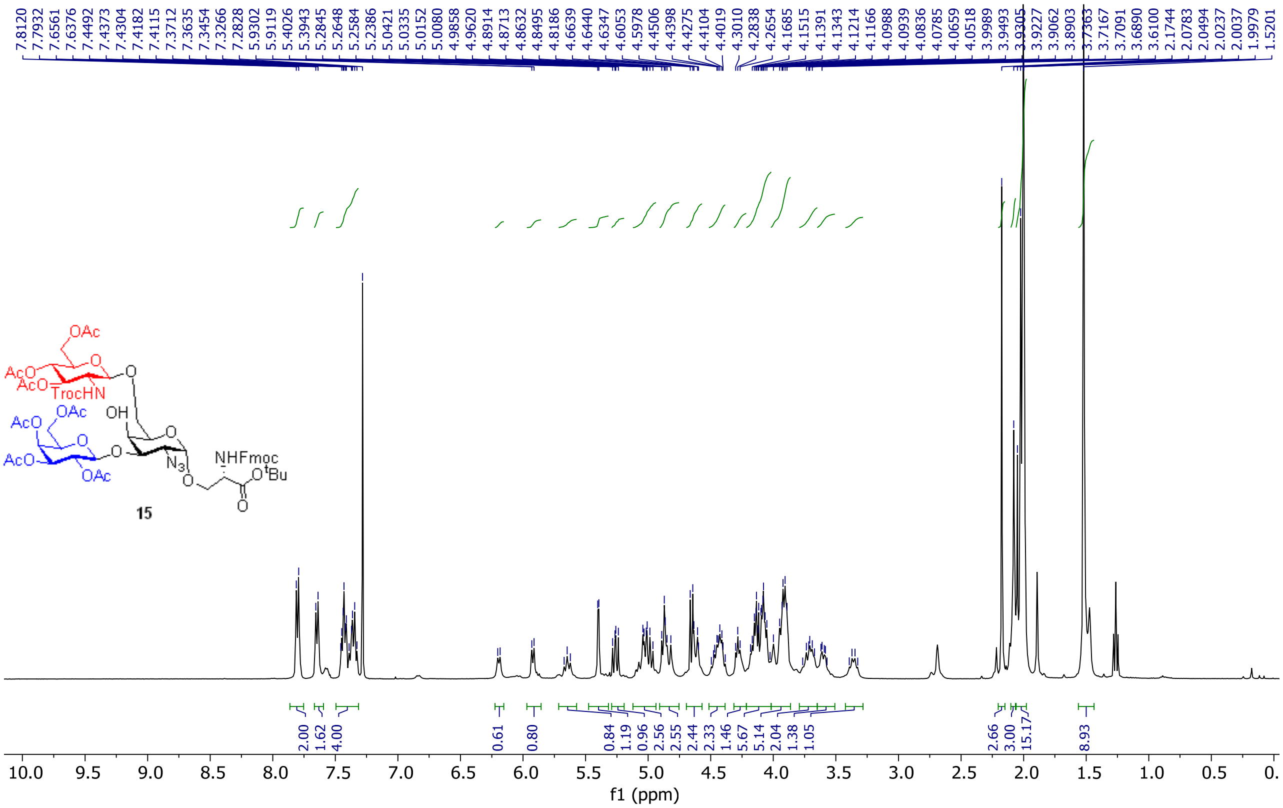

^{1}H NMR of 15

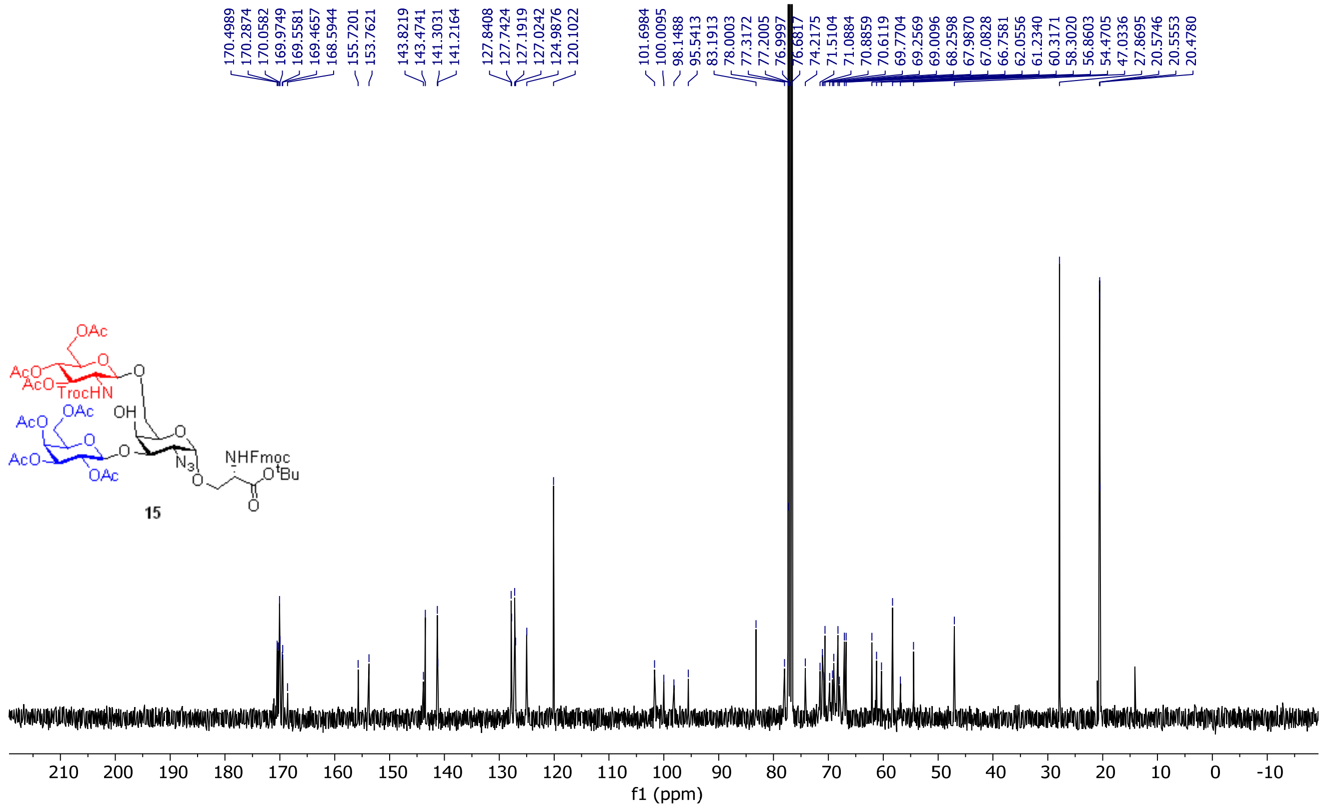

^{13}C NMR of 15

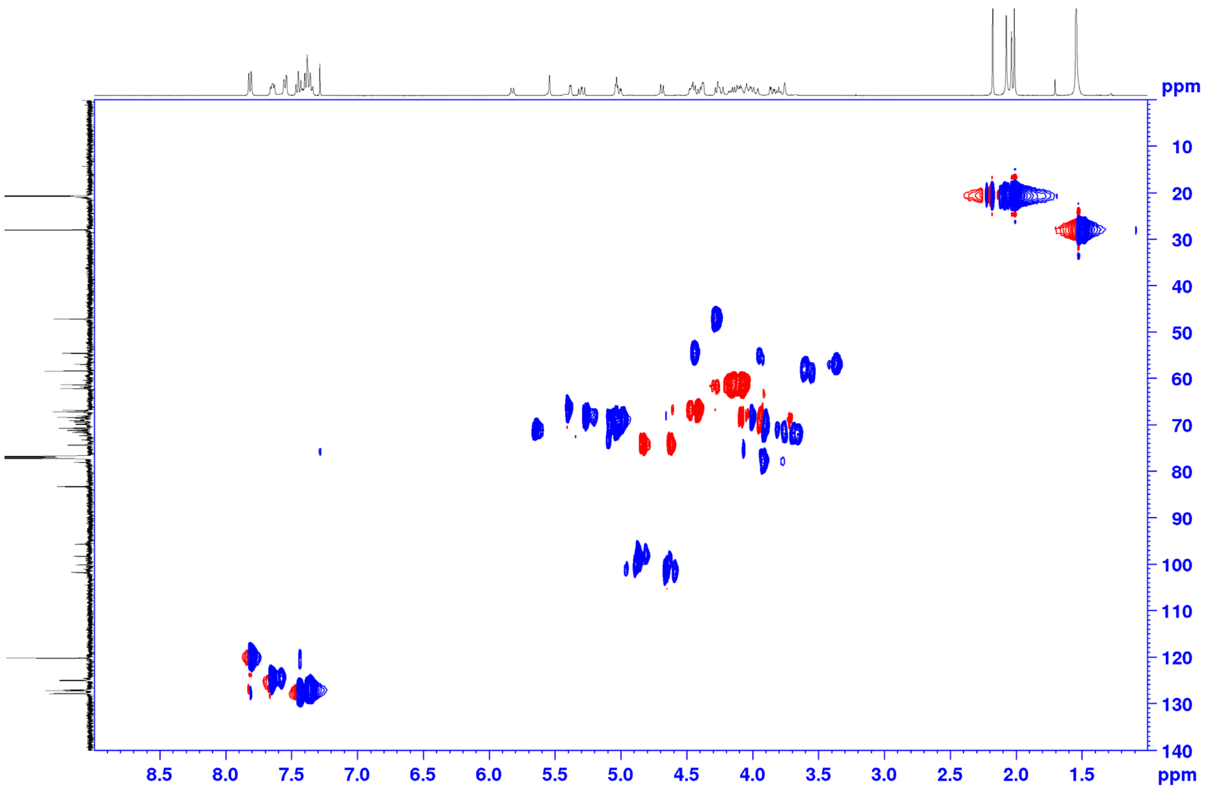

HSQC of 15

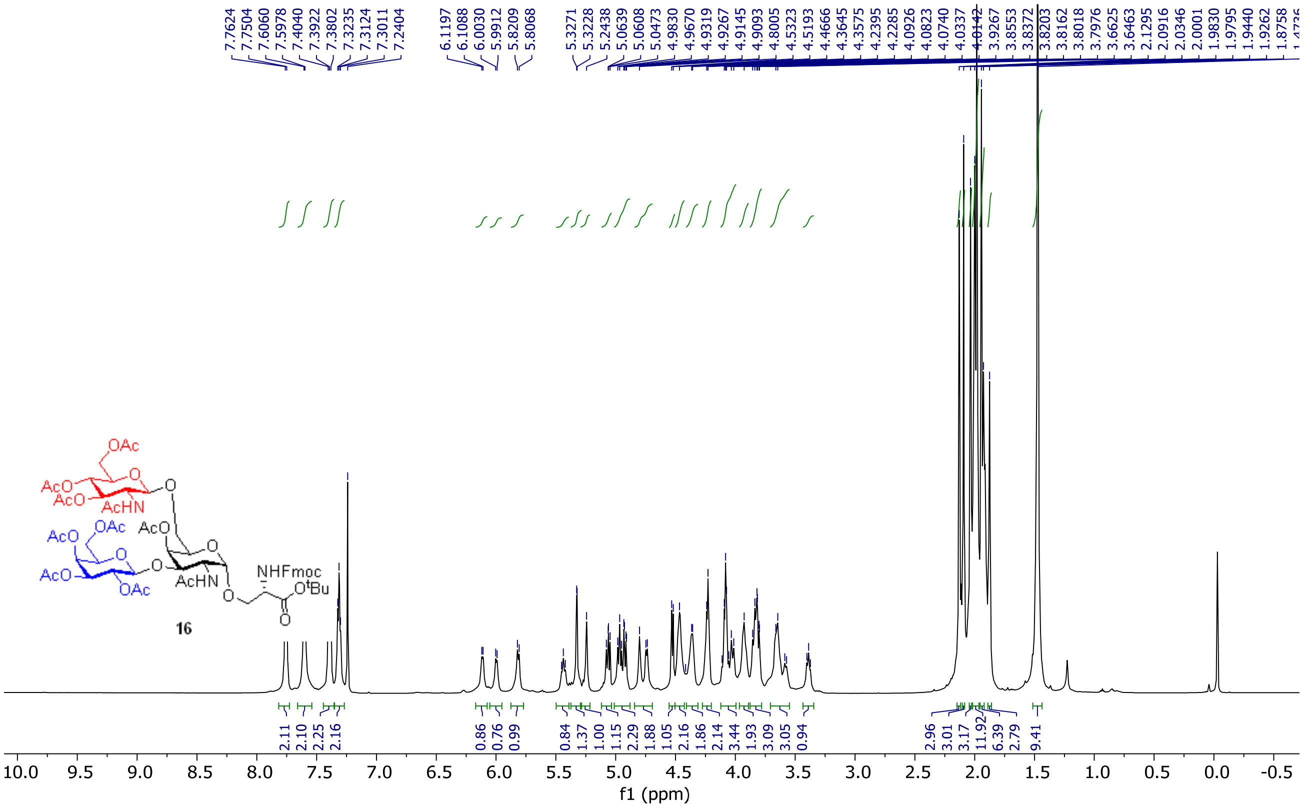

^{1}H NMR of 16

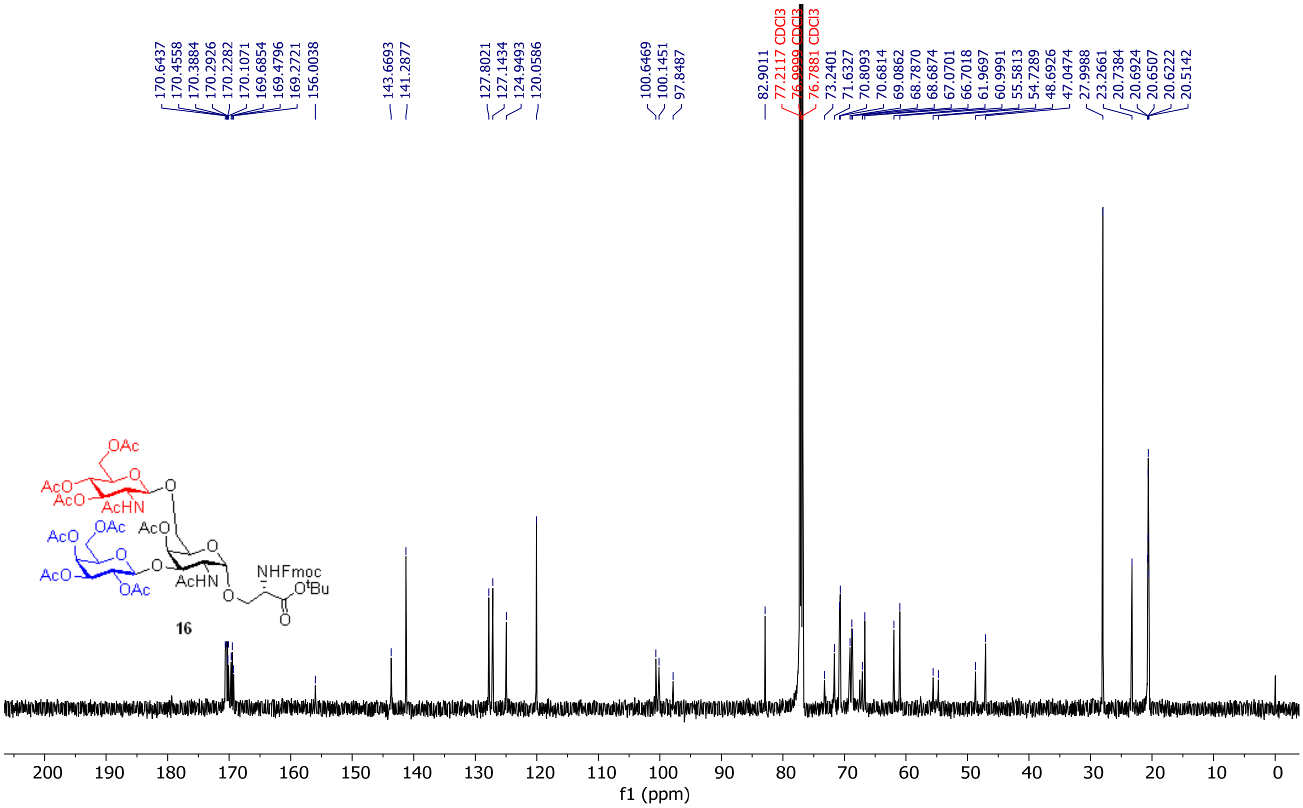

^{13}C NMR of 16

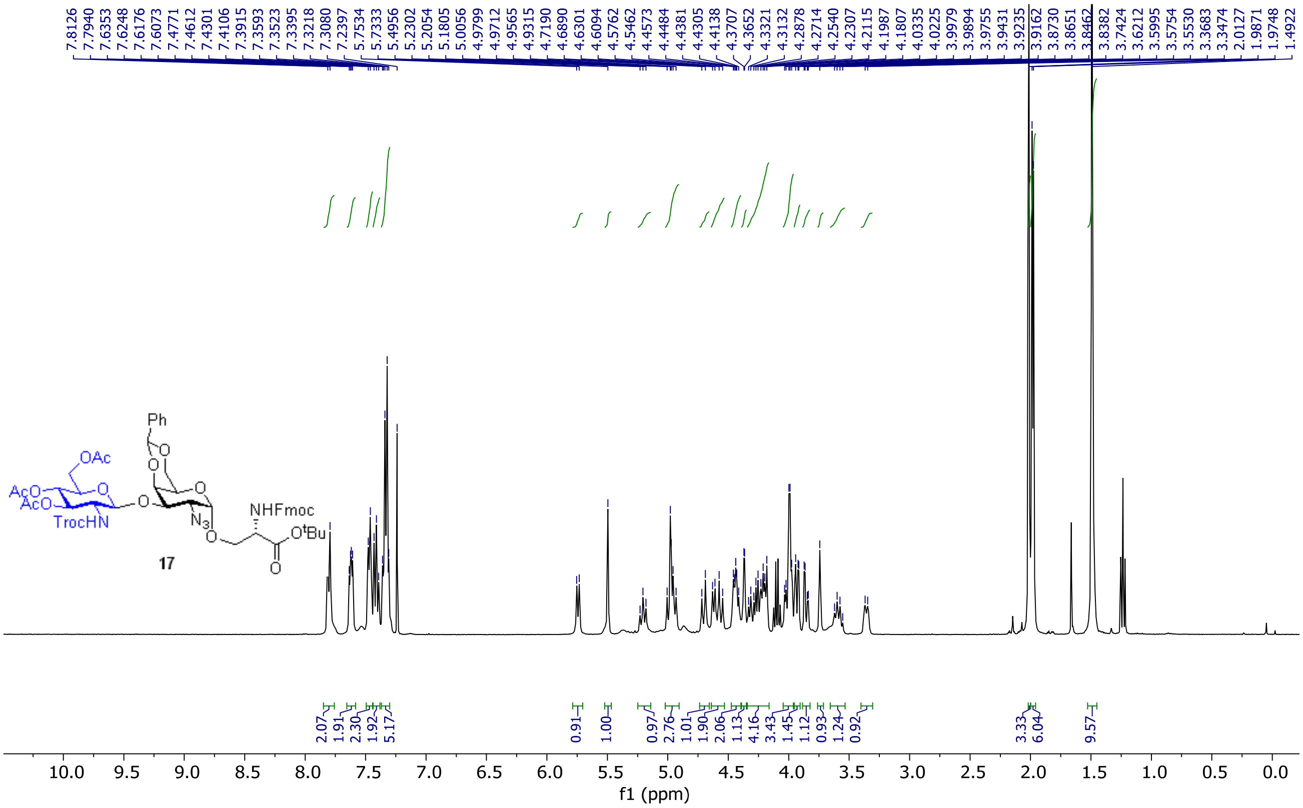

^{1}H NMR of 17

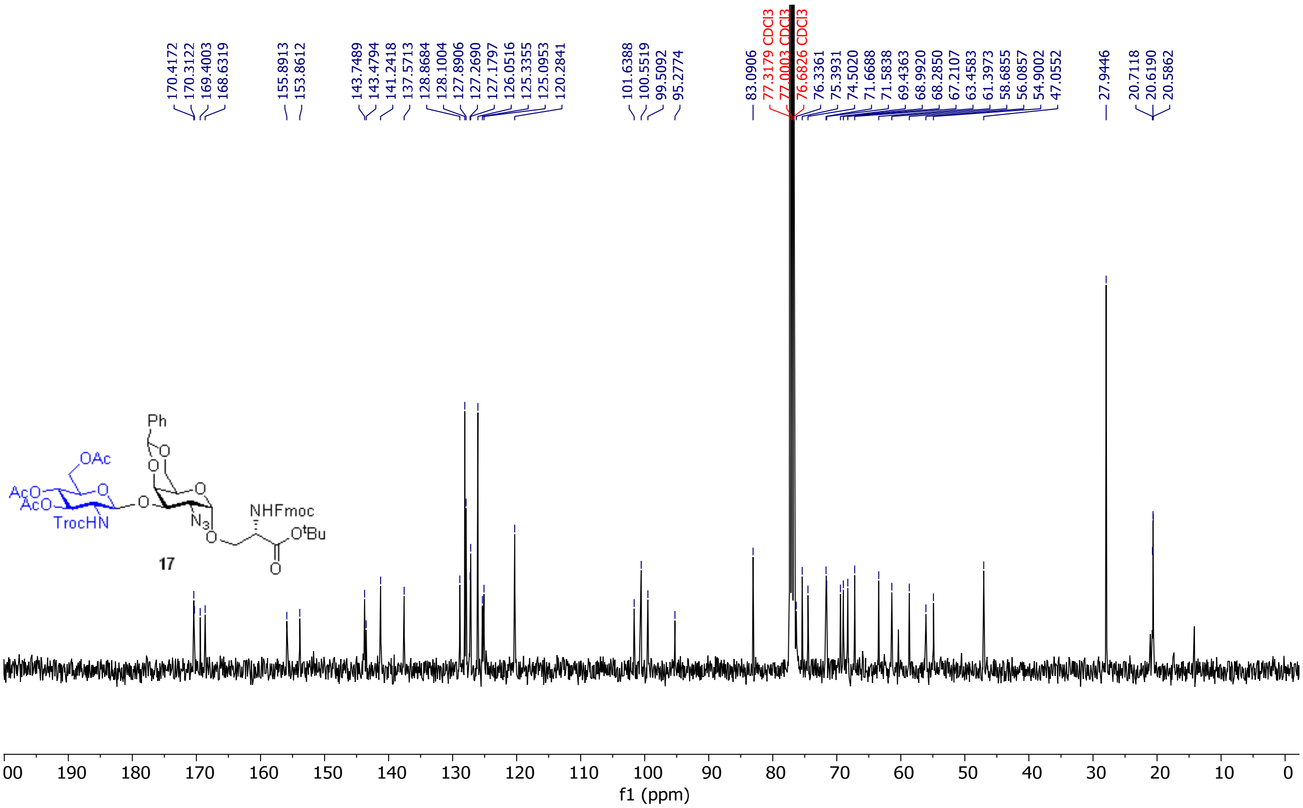

^{13}C NMR of 17

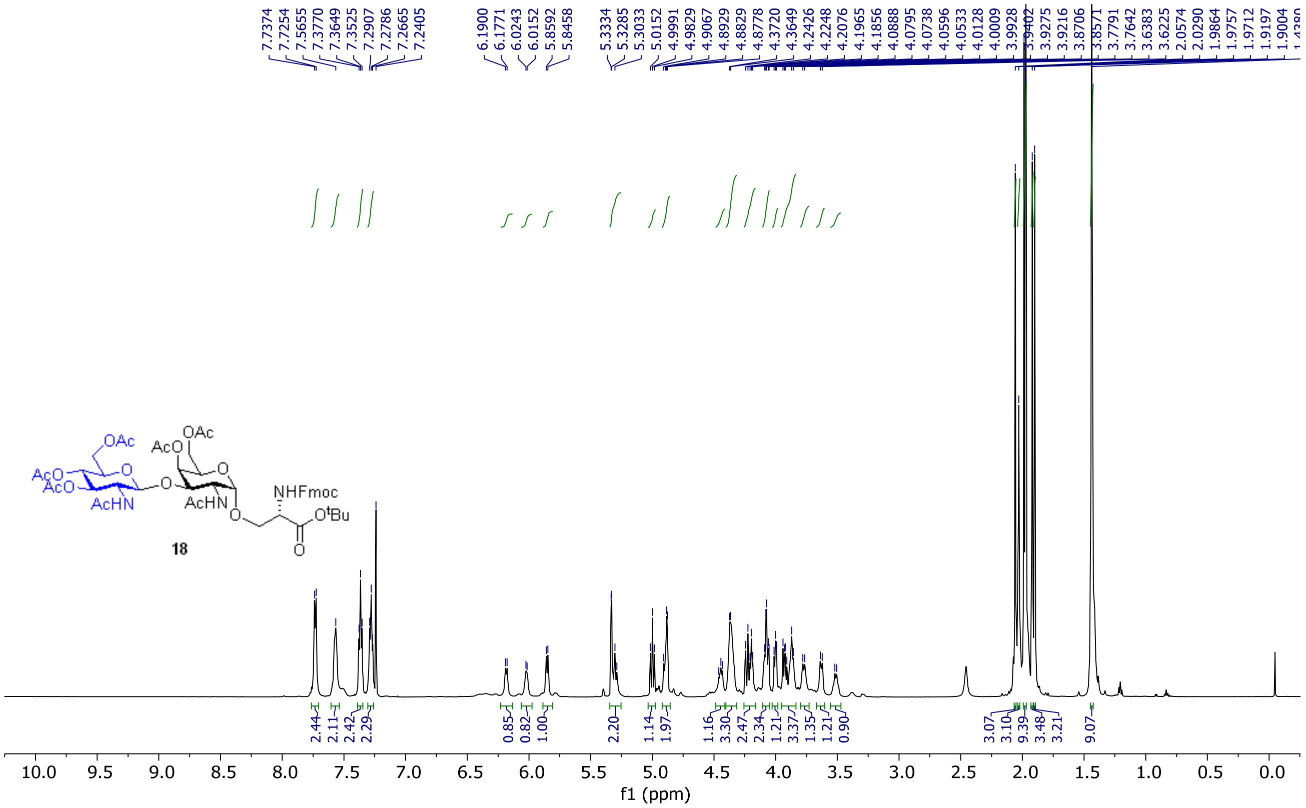

^{1}H NMR of 18

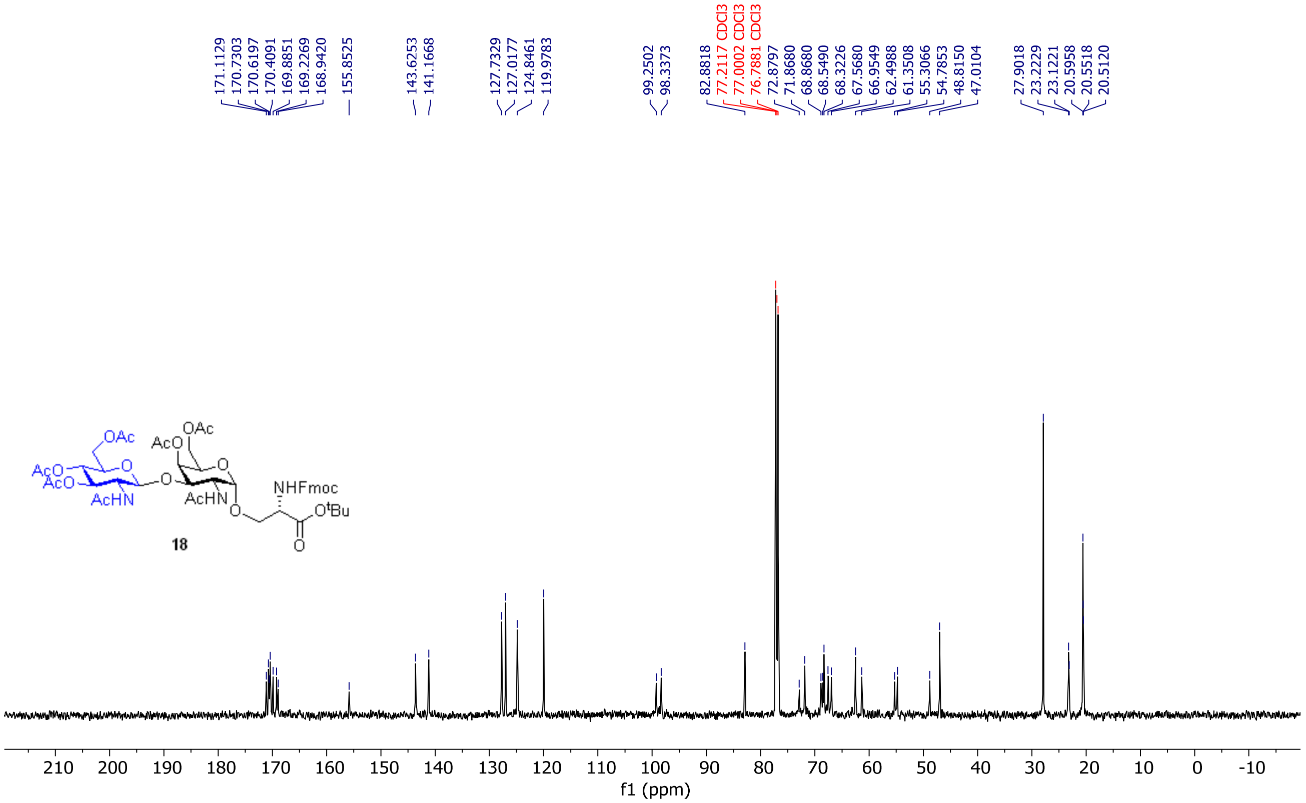

^{13}C NMR of 18

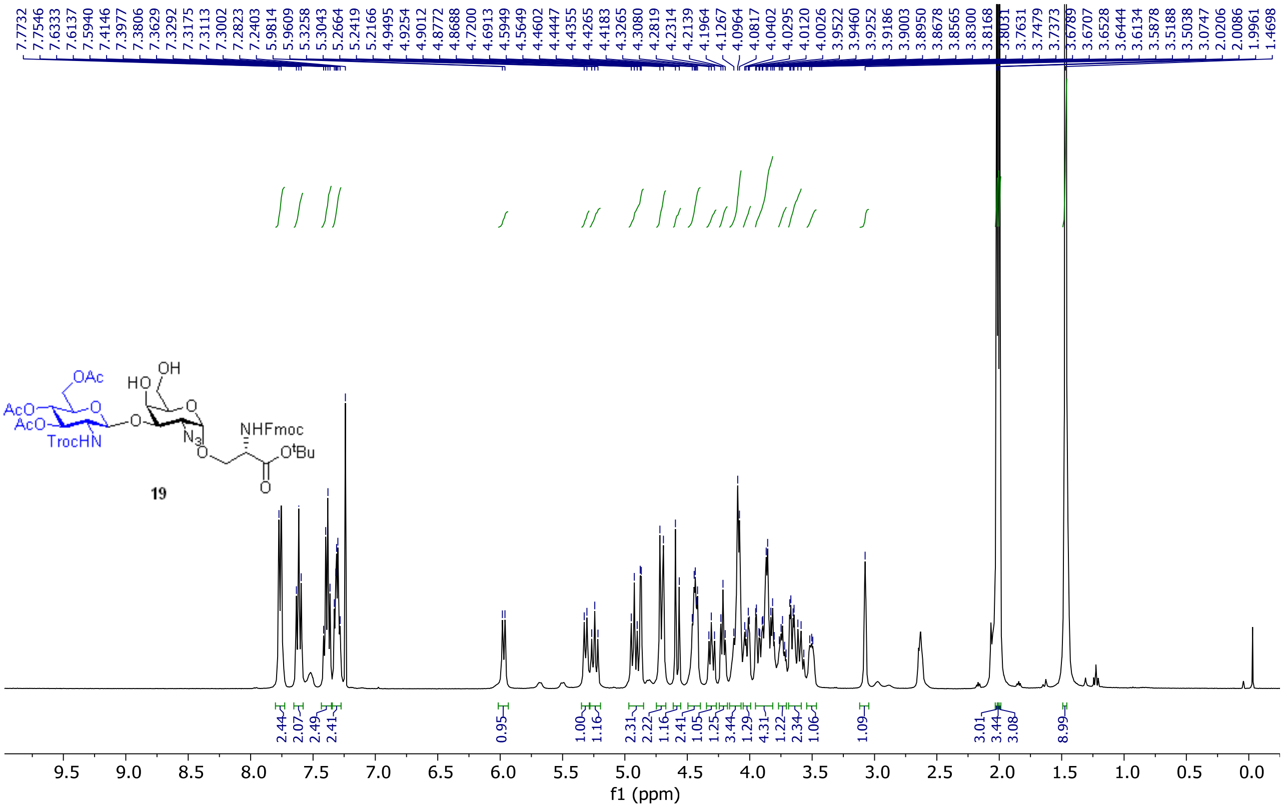

^{1}H NMR of 19

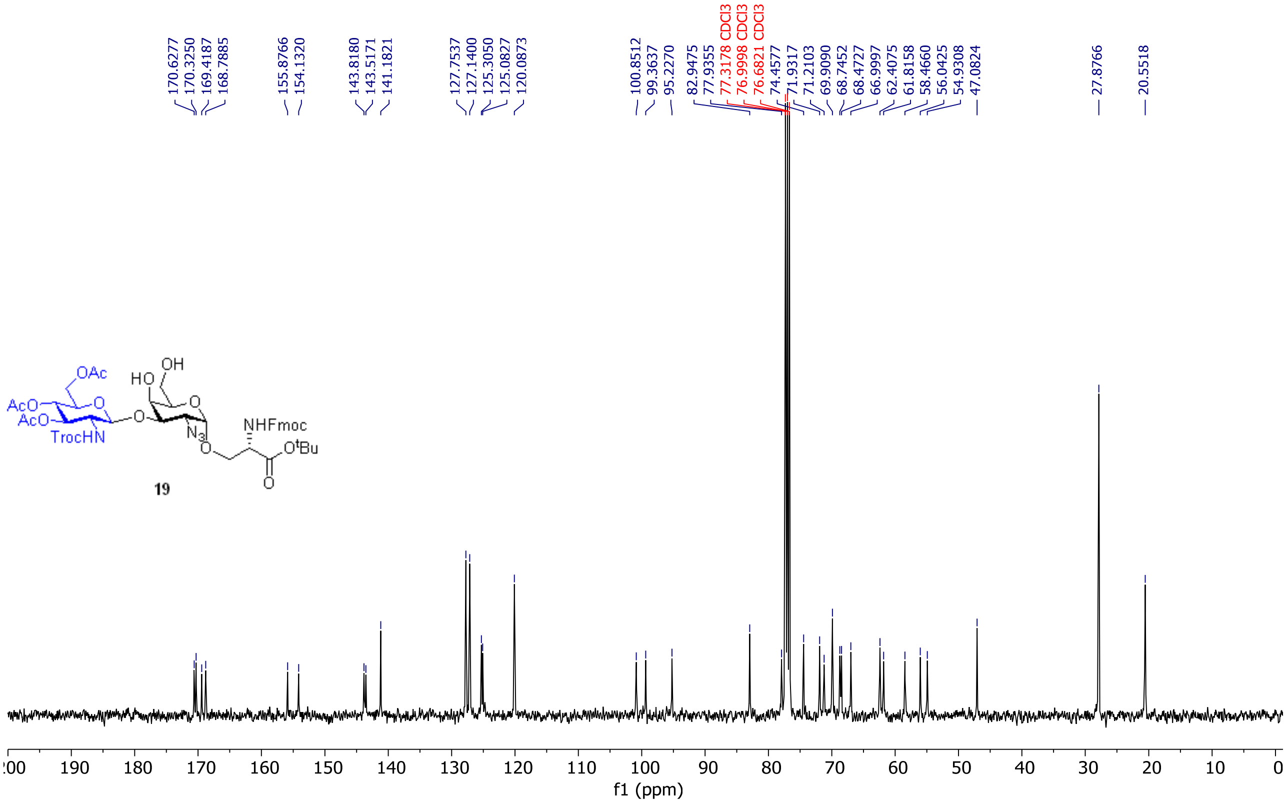

^{13}C NMR of 19

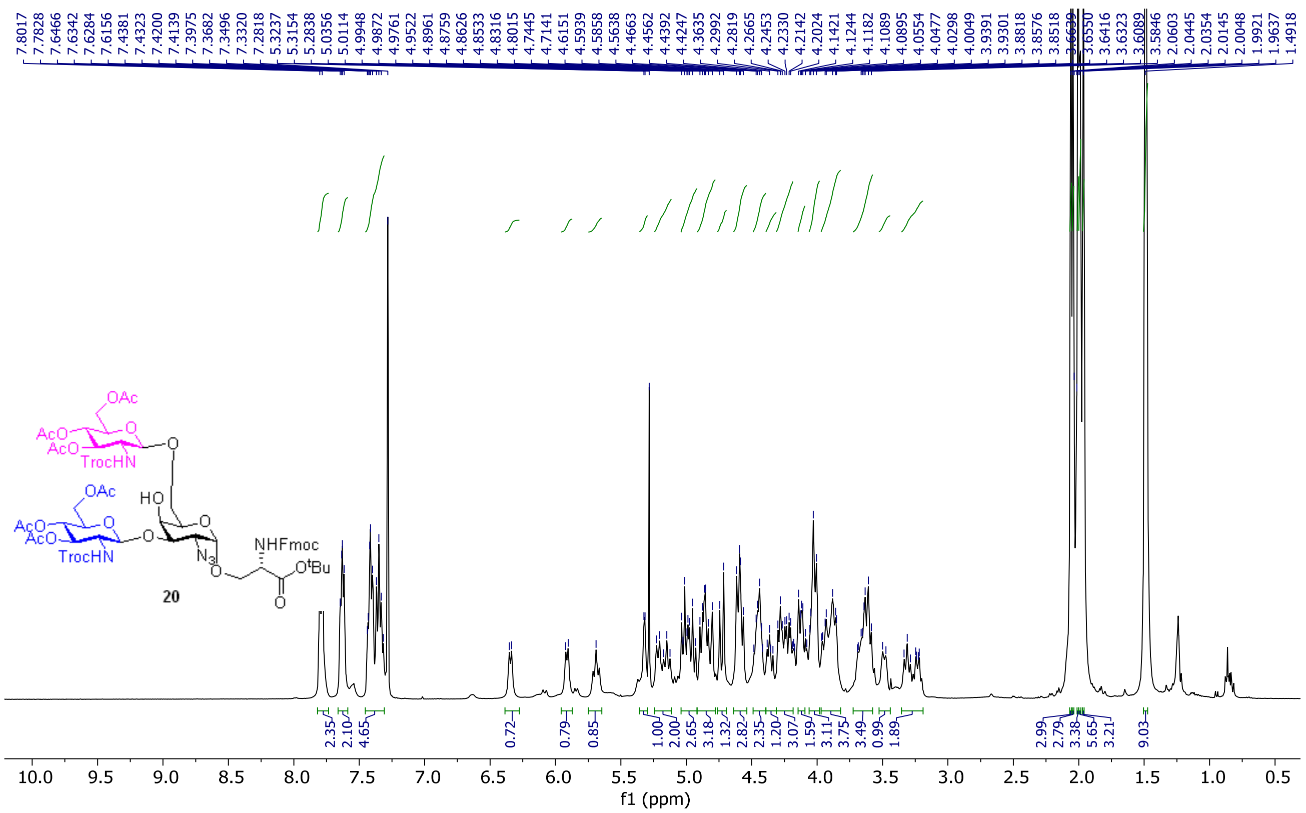

^{1}H NMR of 20

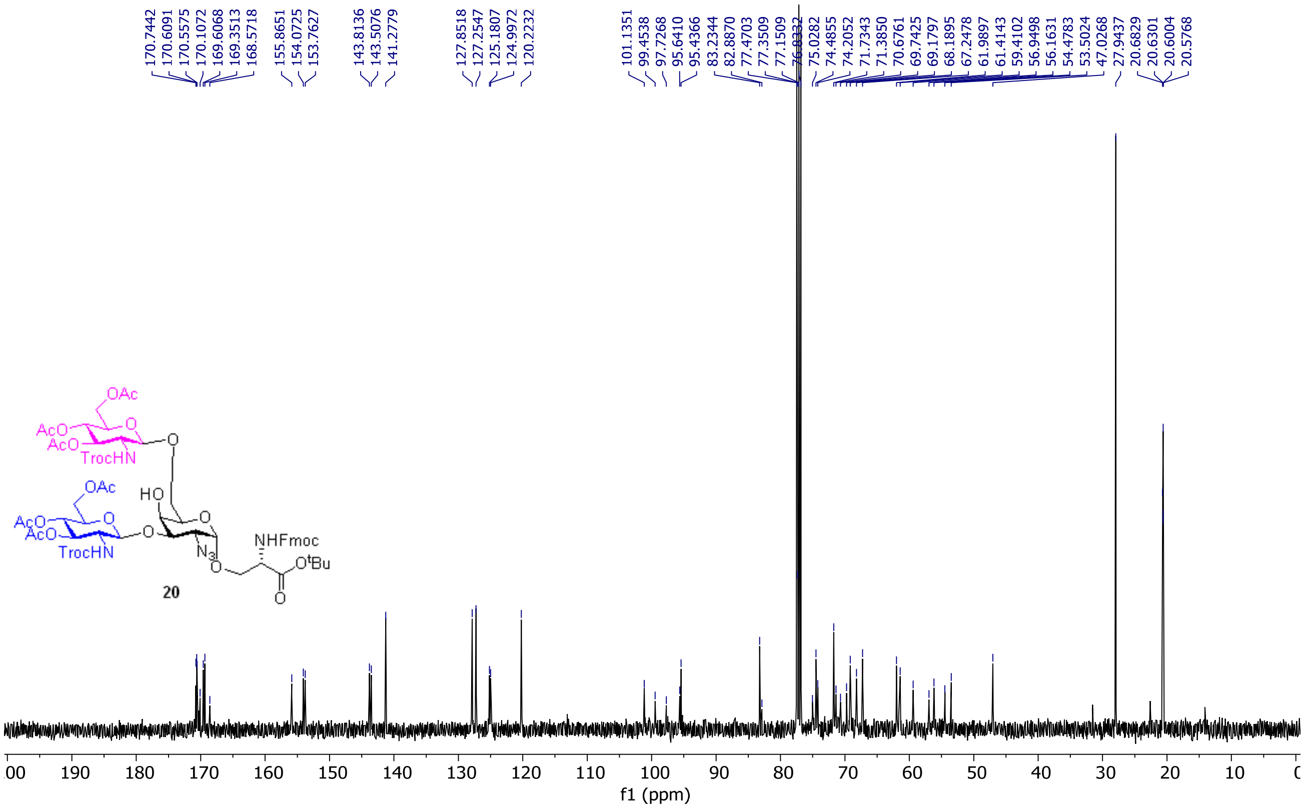

^{13}C NMR of 20

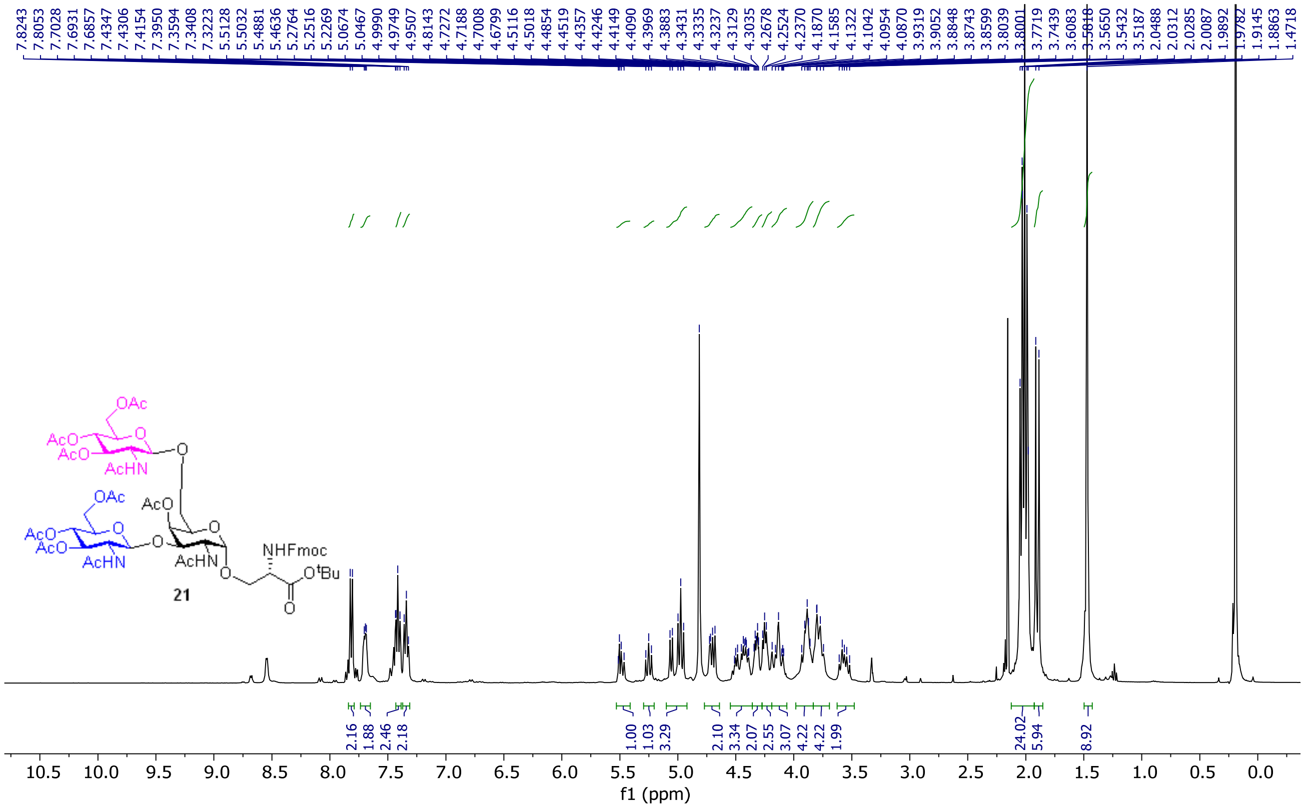

^{1}H NMR of 21

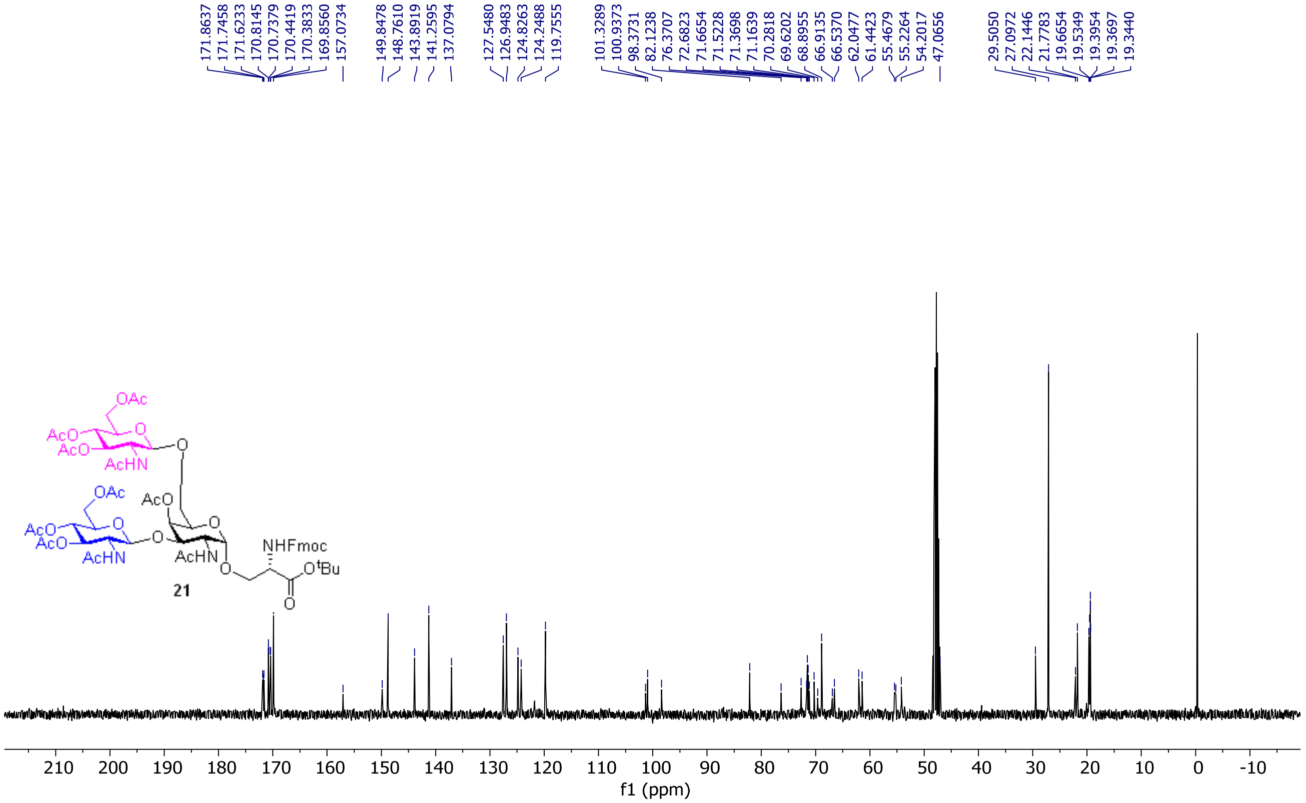

^{13}C NMR of 21

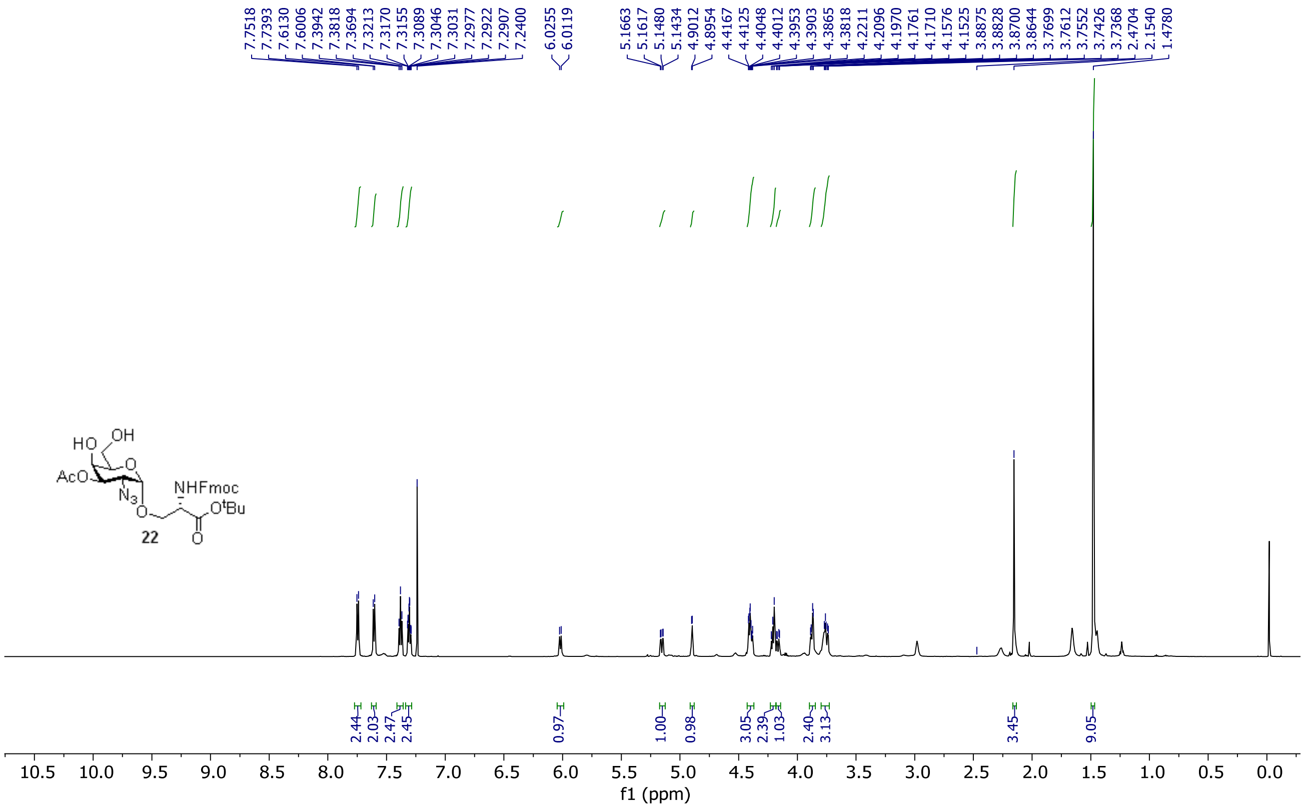

^{1}H NMR of 22

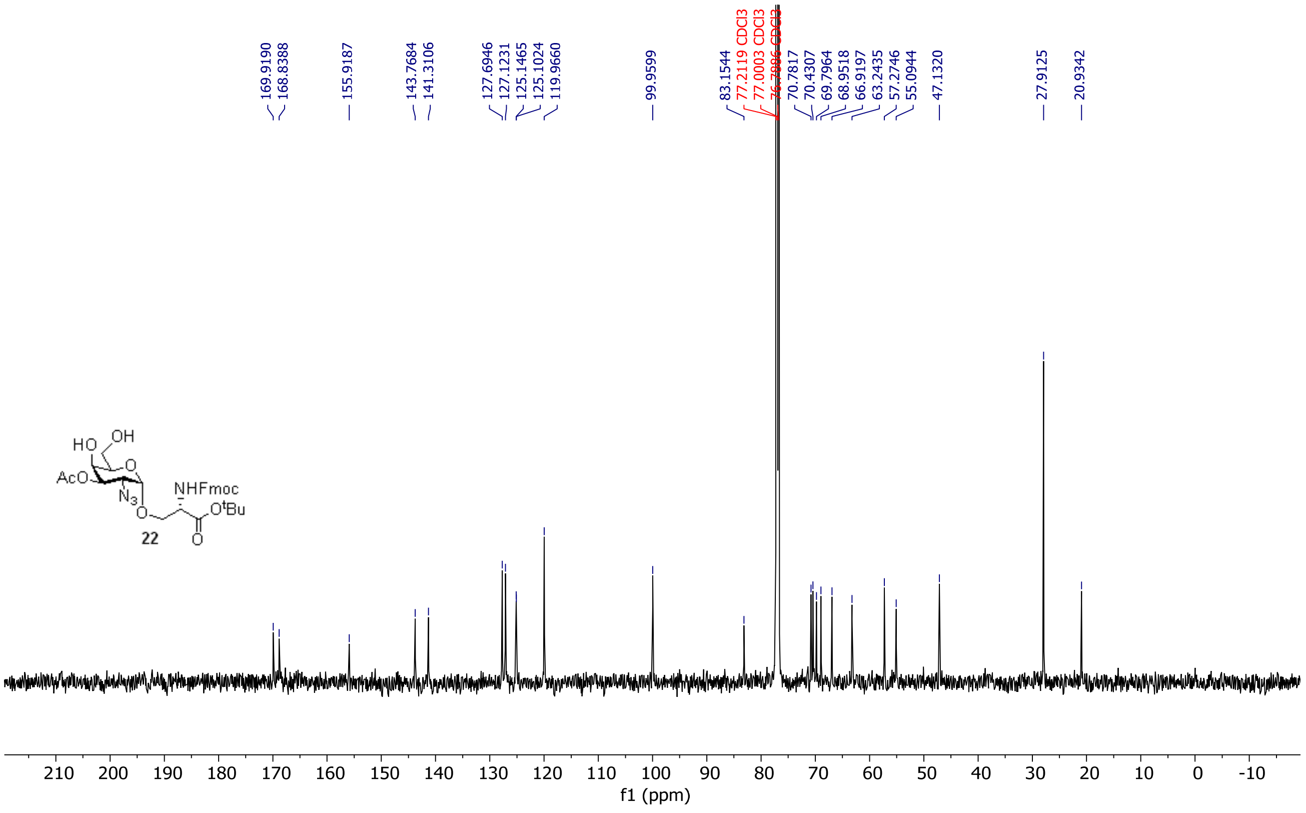

^{13}C NMR of 22

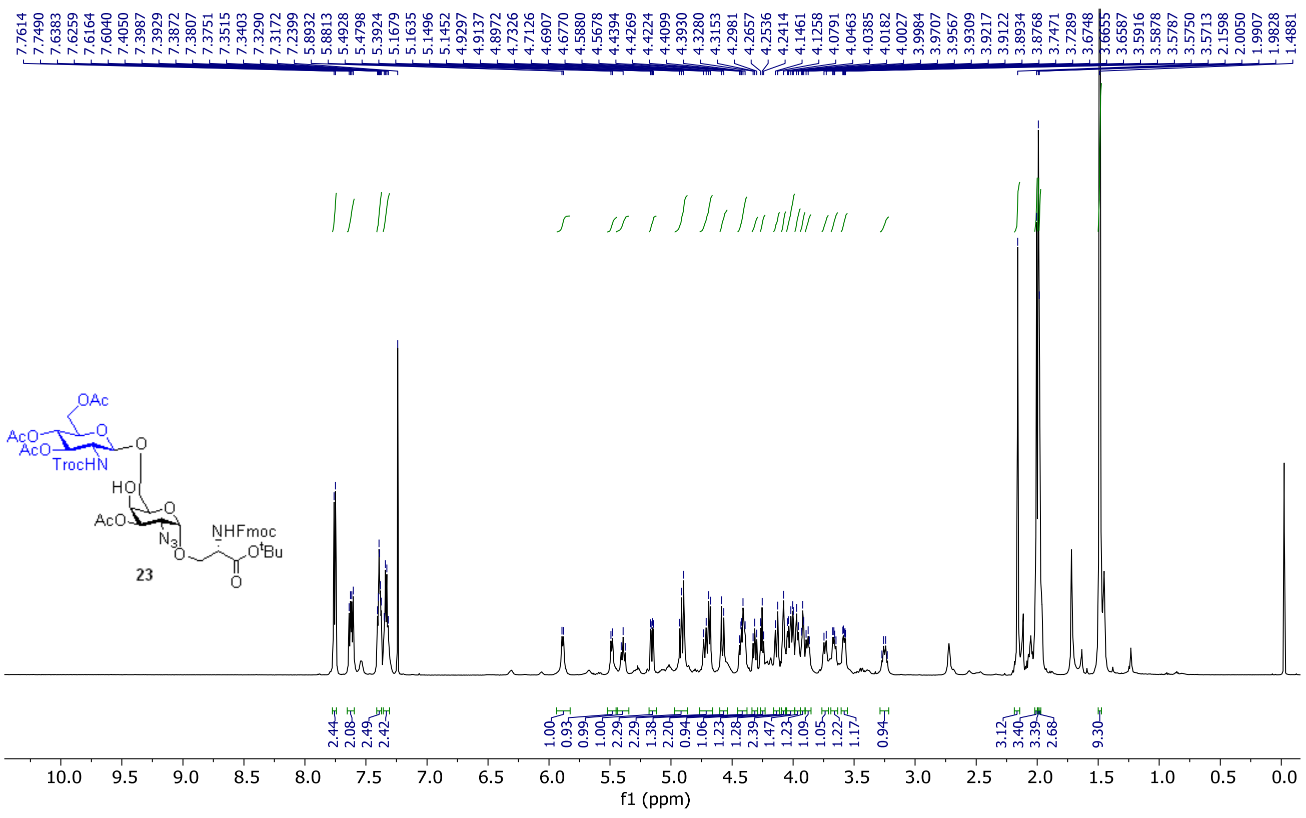

^{1}H NMR of 23

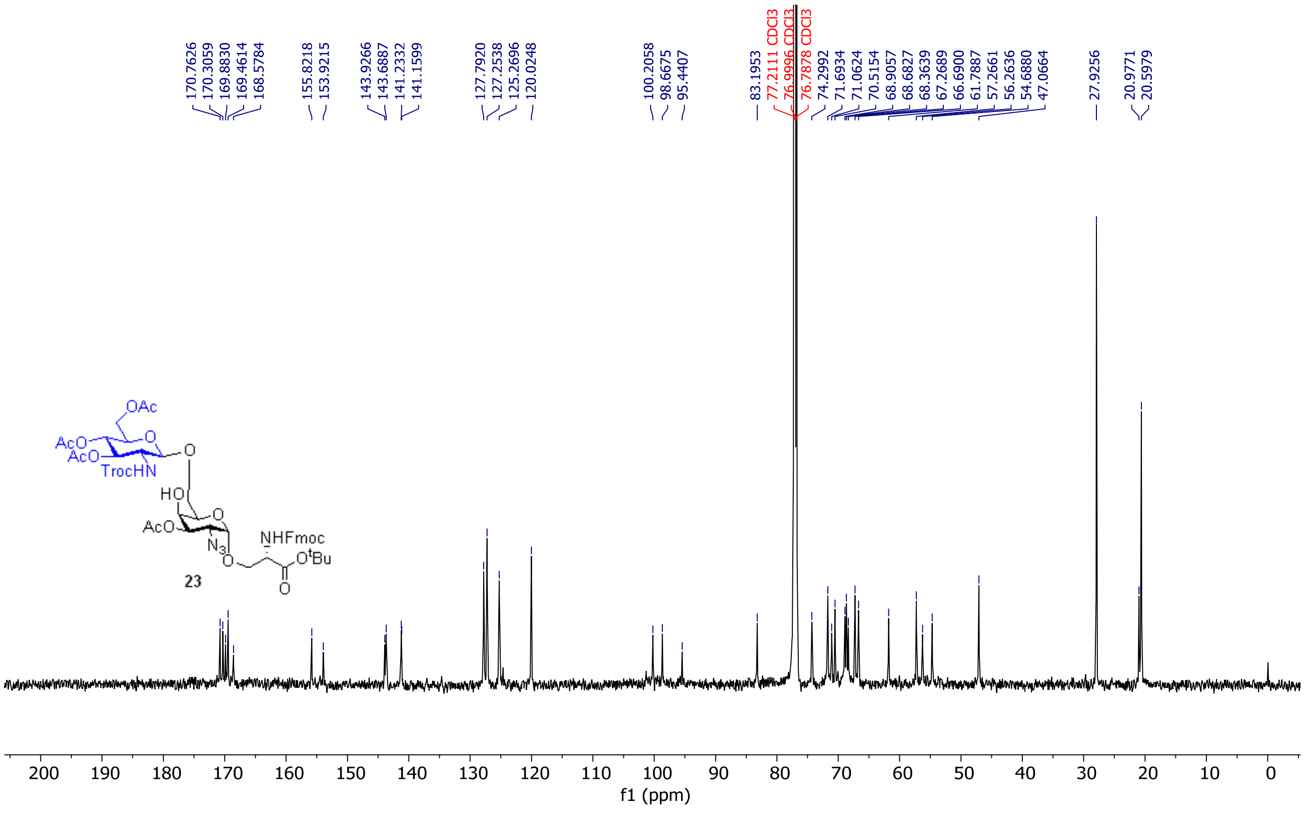

^{13}C NMR of 23

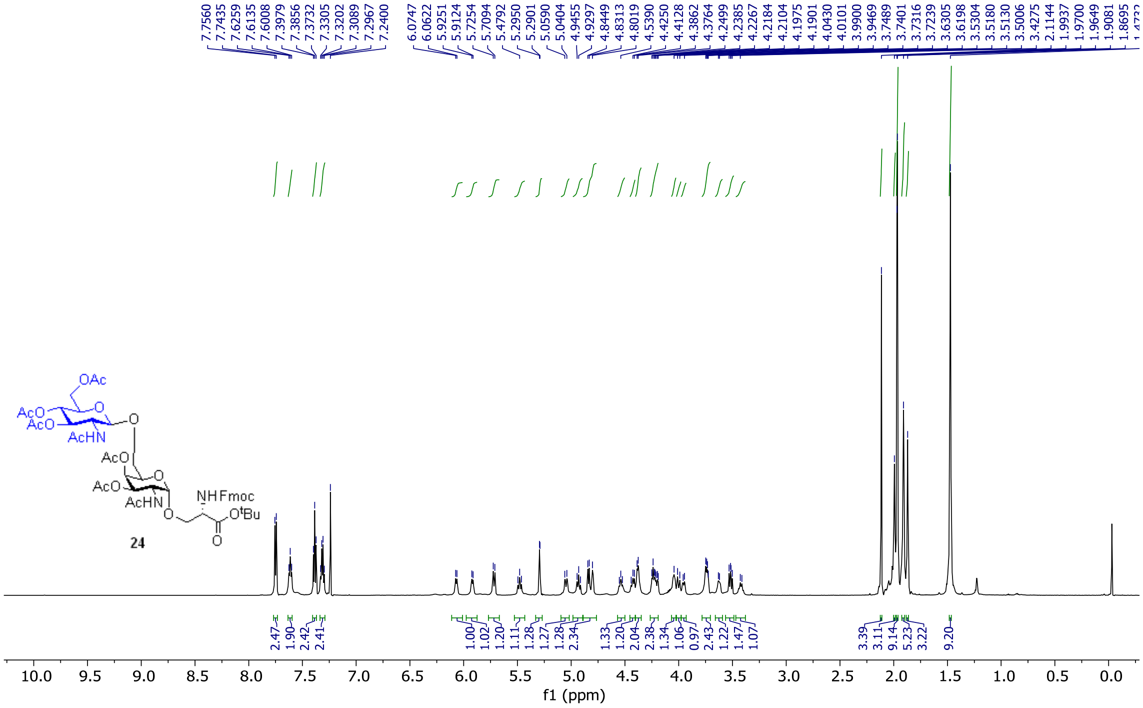

^{1}H NMR of 24

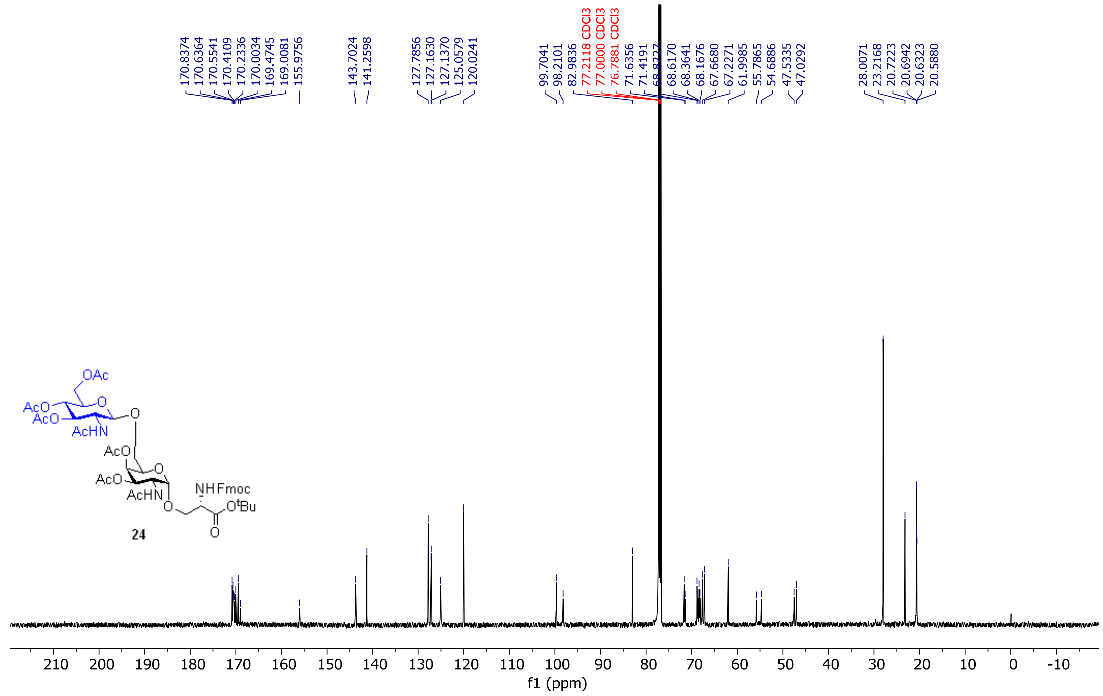

^{13}C NMR of 24

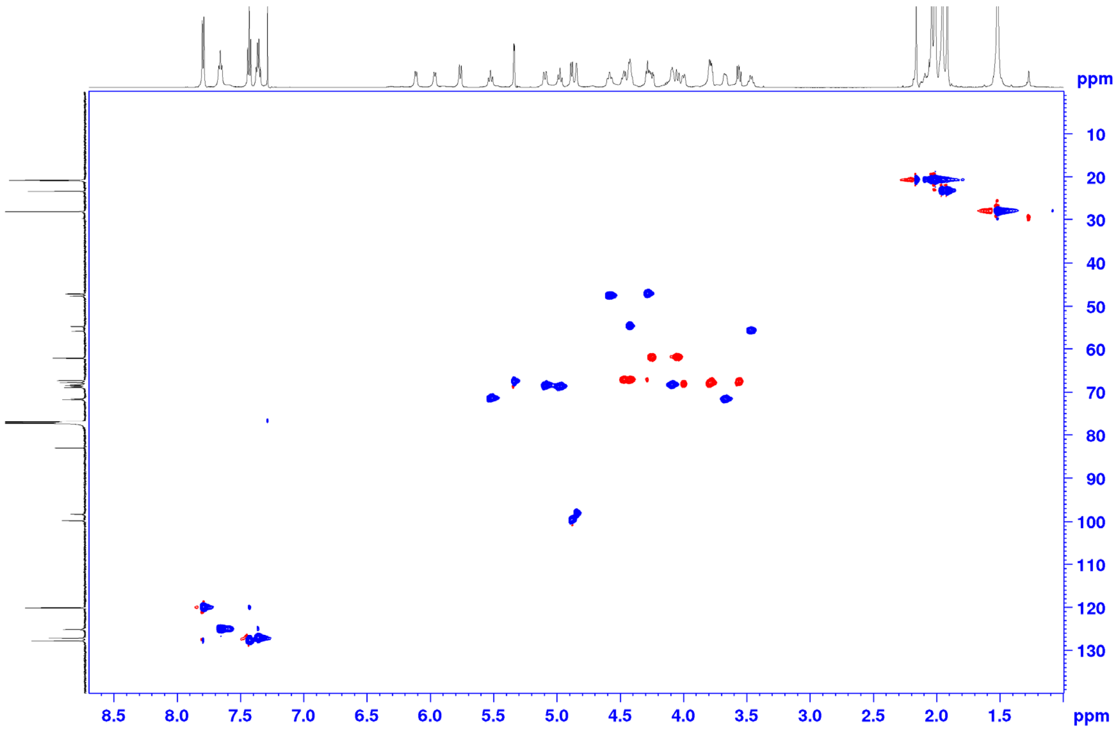

HSQC of **24**

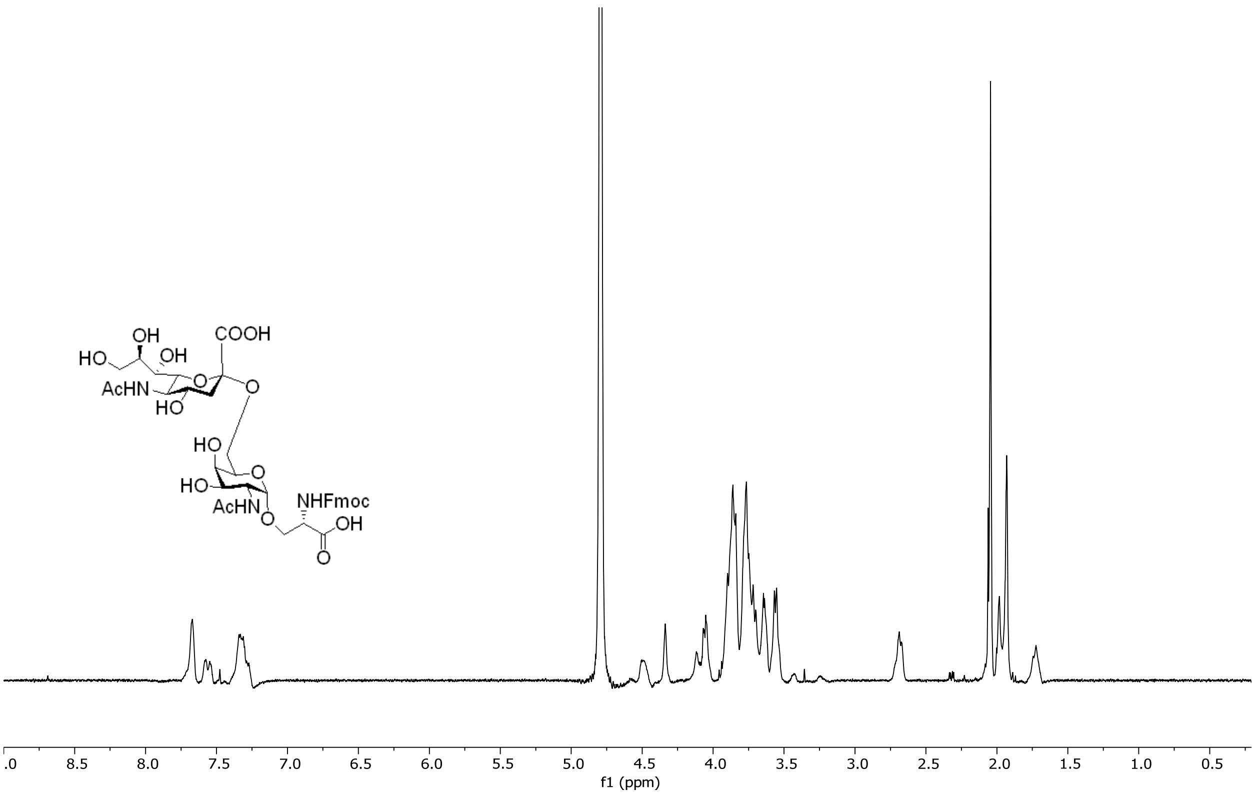

^{1}H NMR of 25

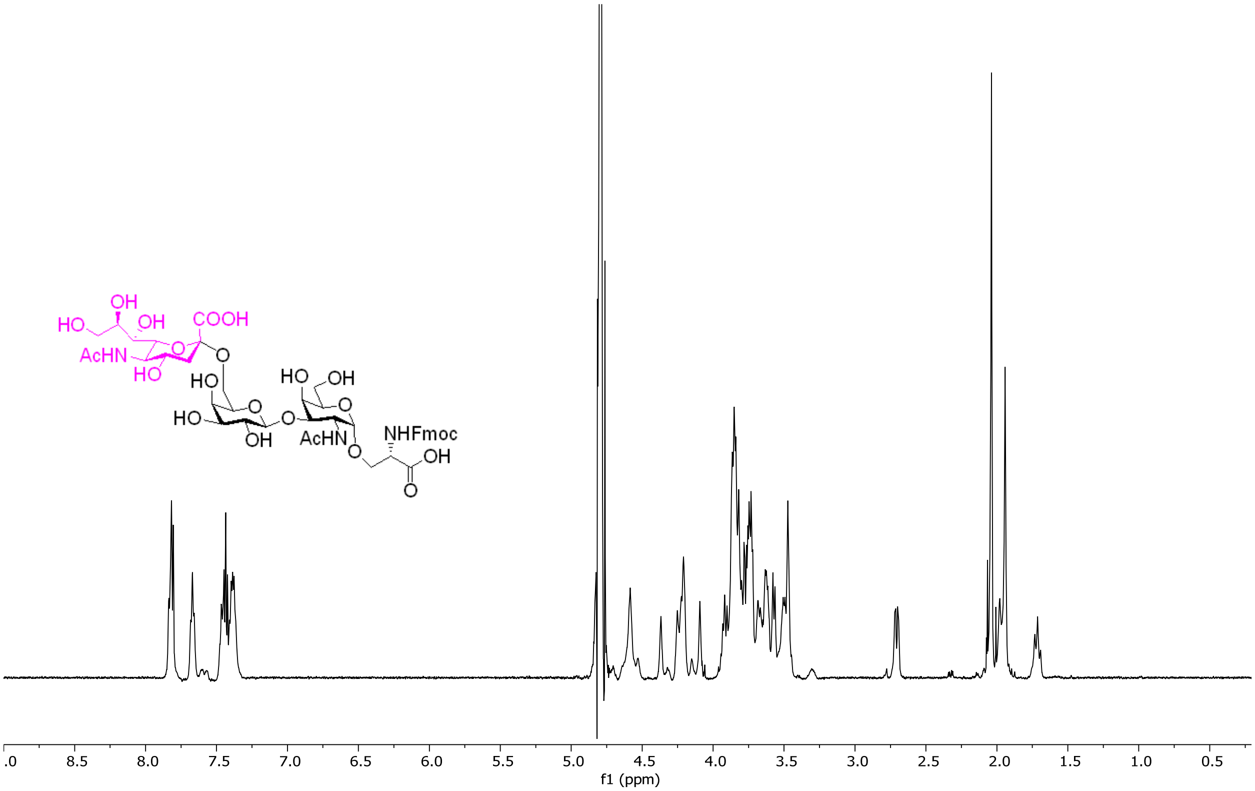

^{1}H NMR of 26

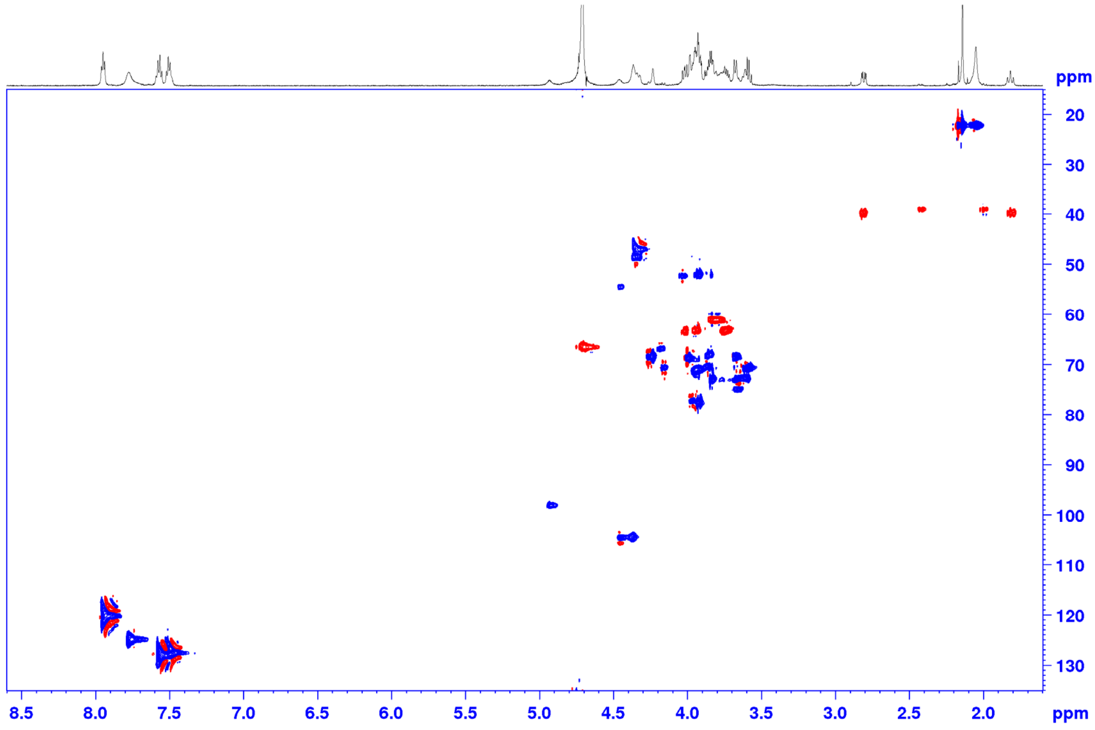

HSQC of 26

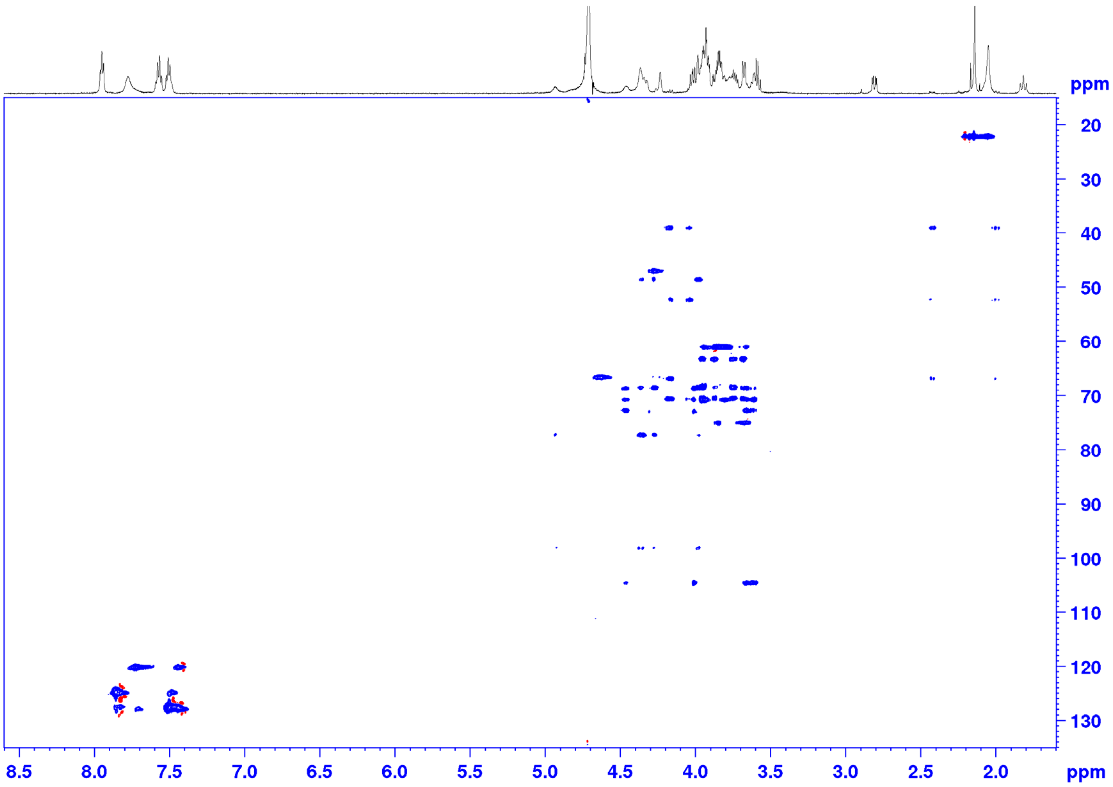

HSQC-Tocsy of 26

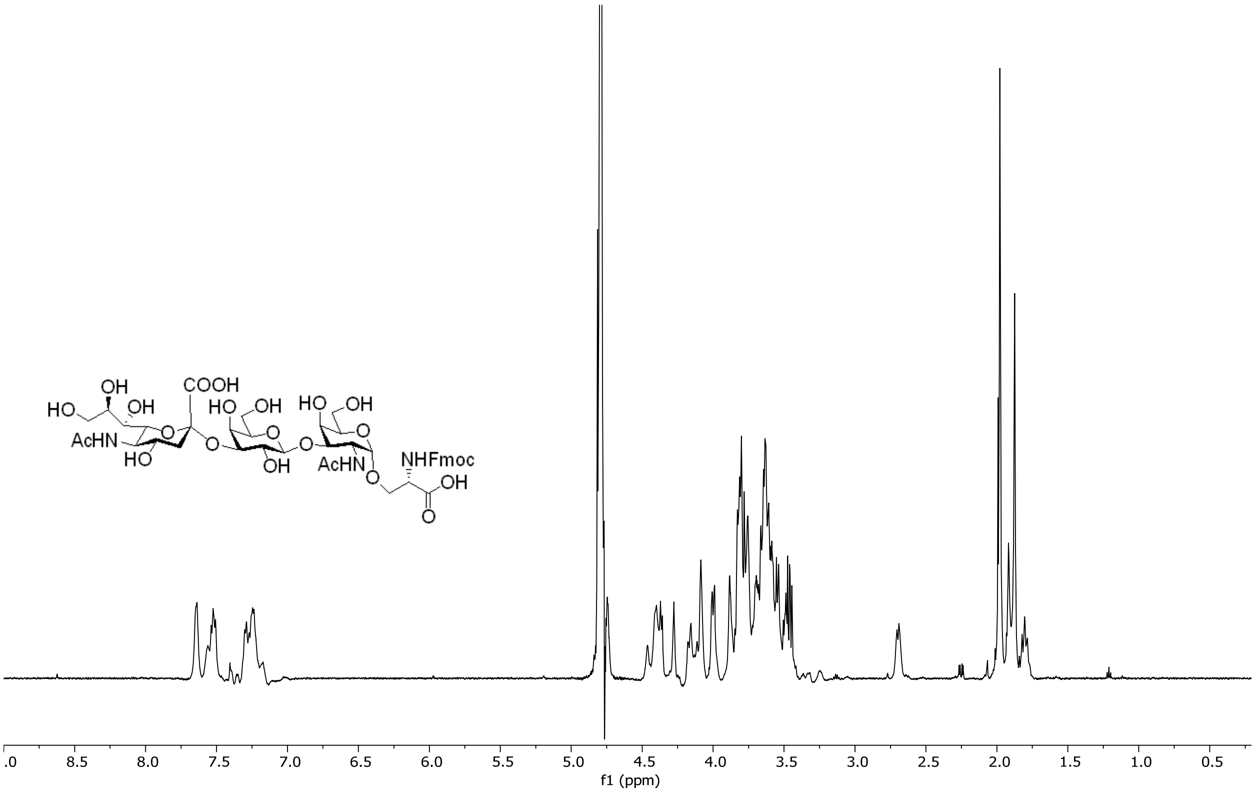

^{1}H NMR of 27

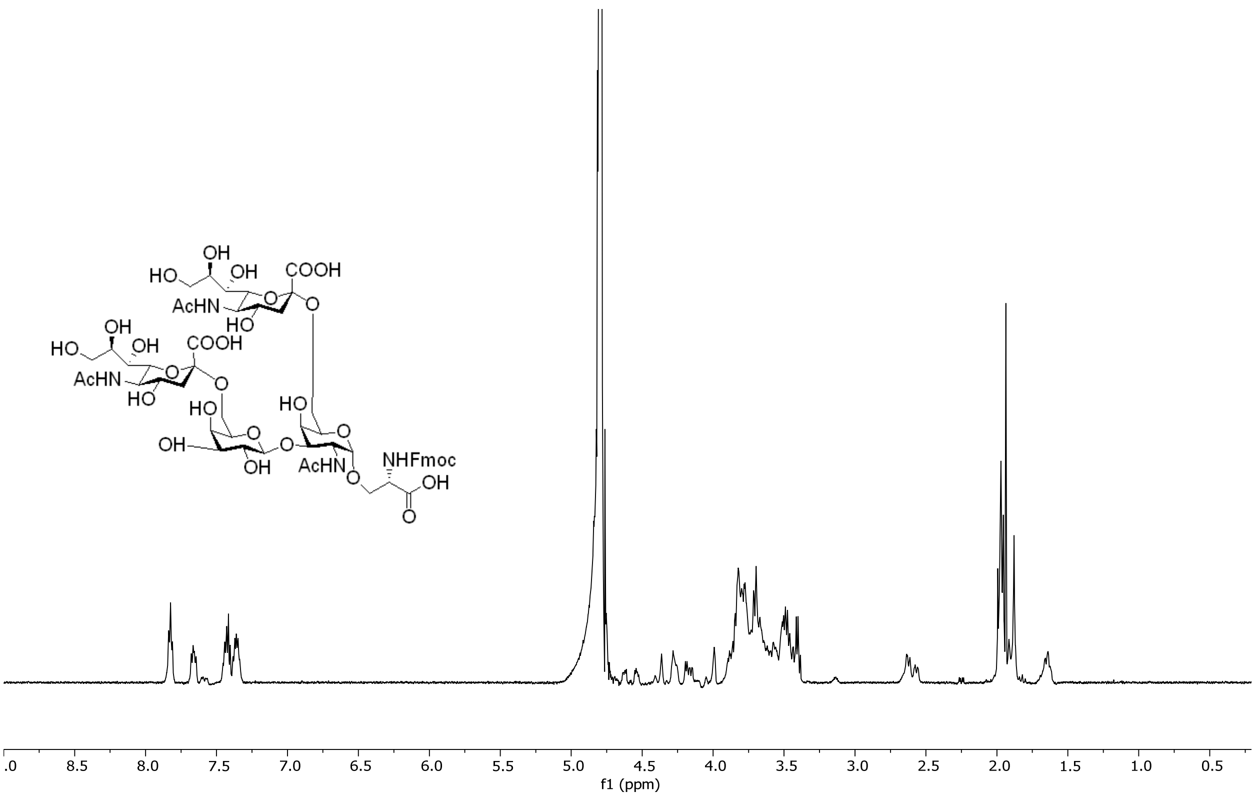

^{1}H NMR of 28

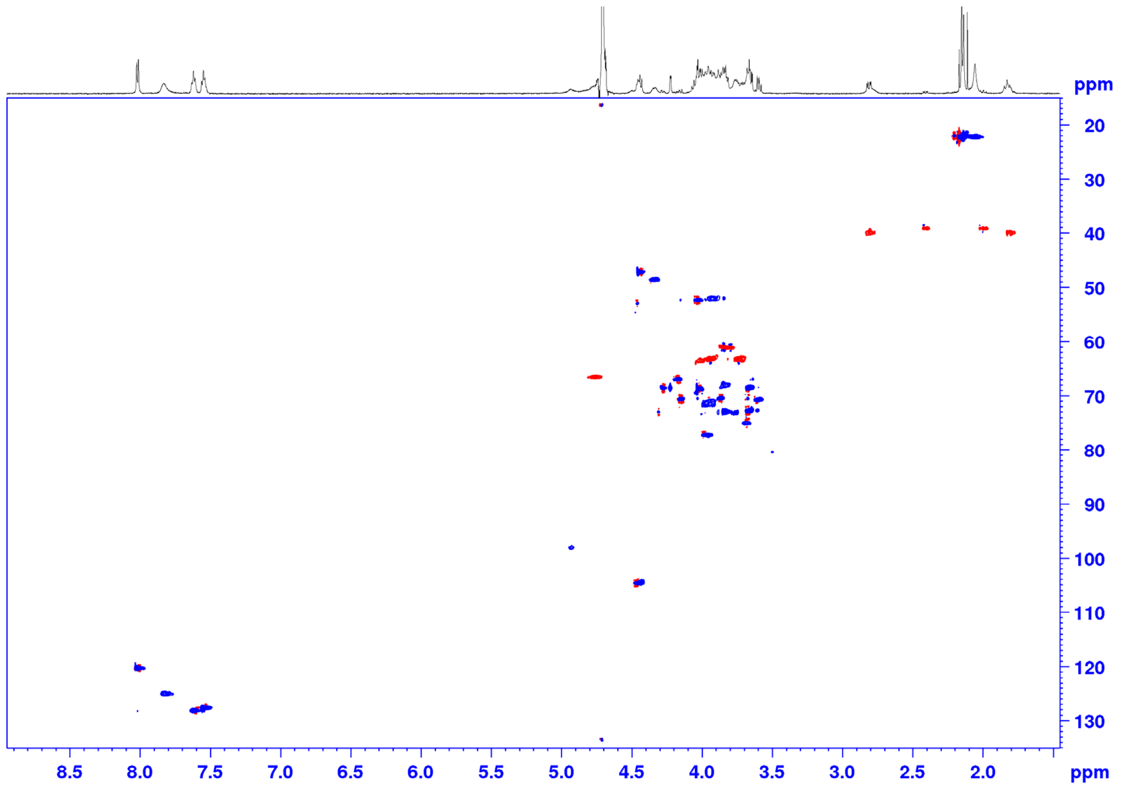

HSQC of 28

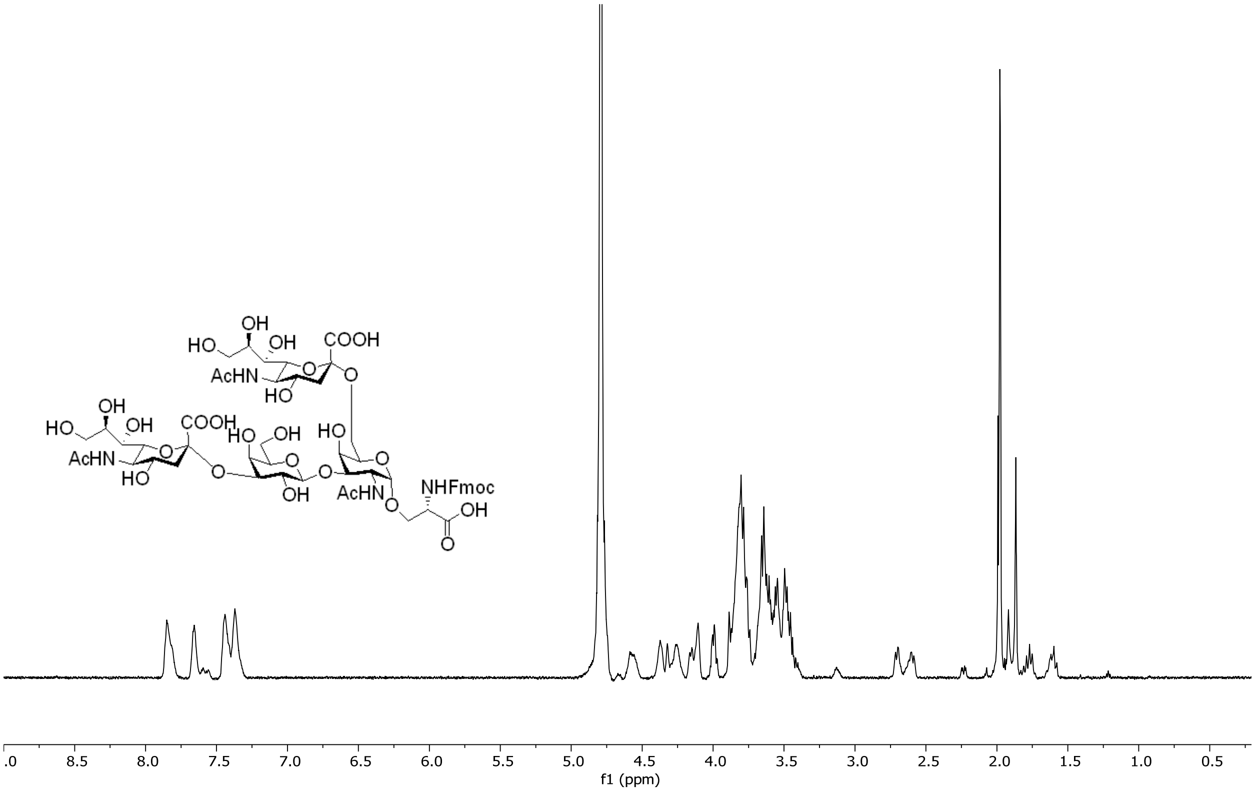

^{1}H NMR of 29

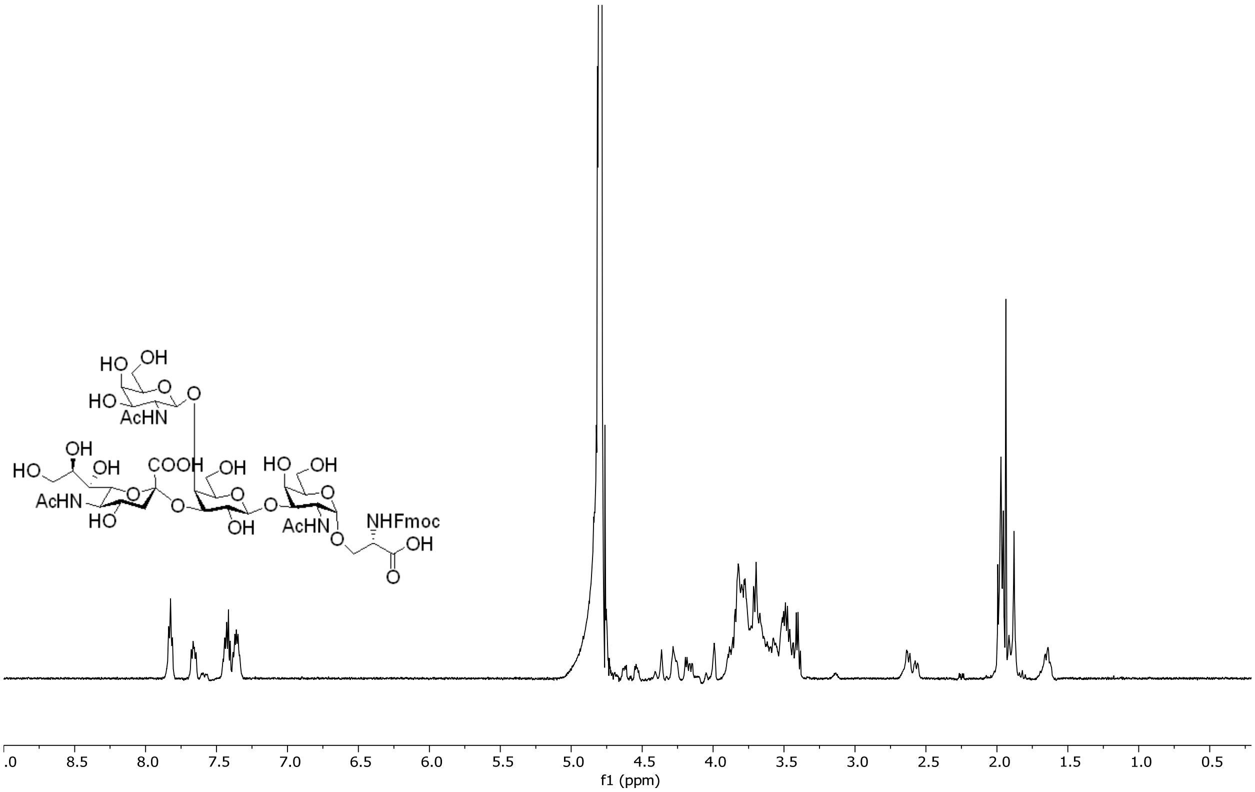

^{1}H NMR of 30

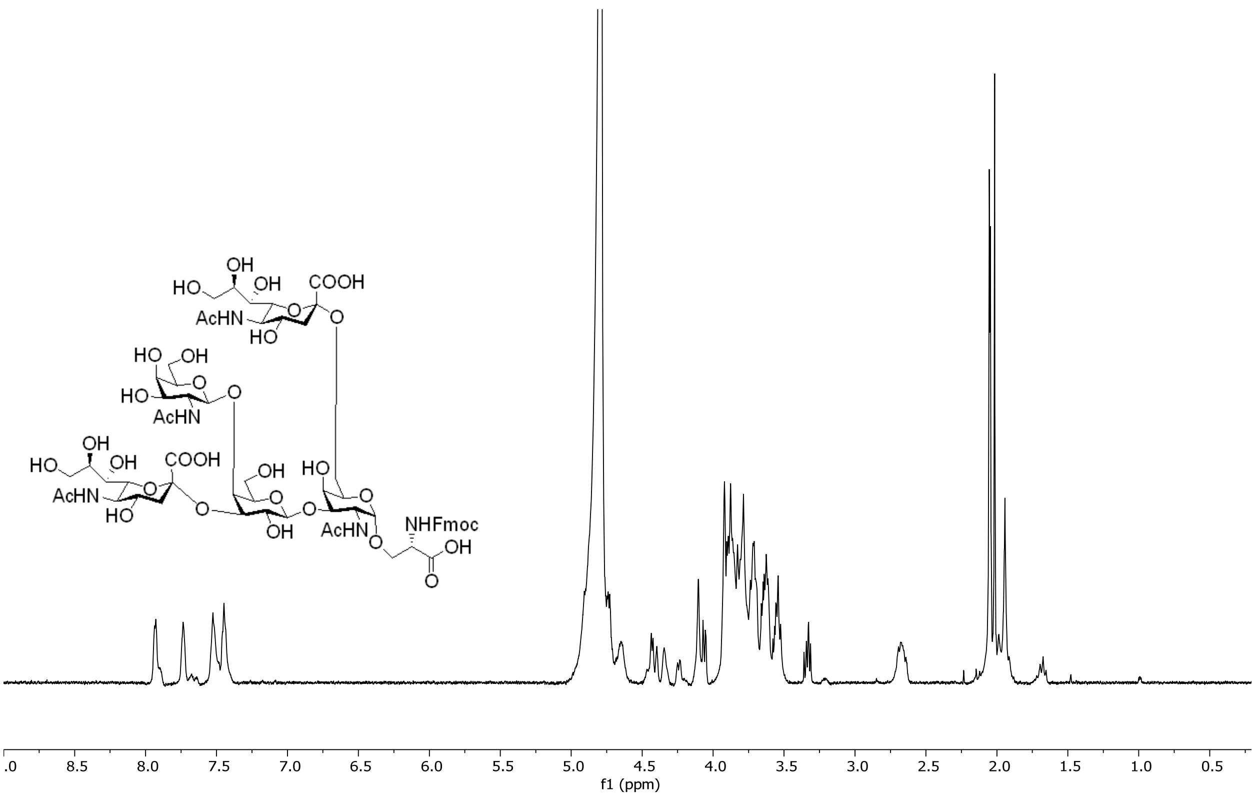

^{1}H NMR of 31

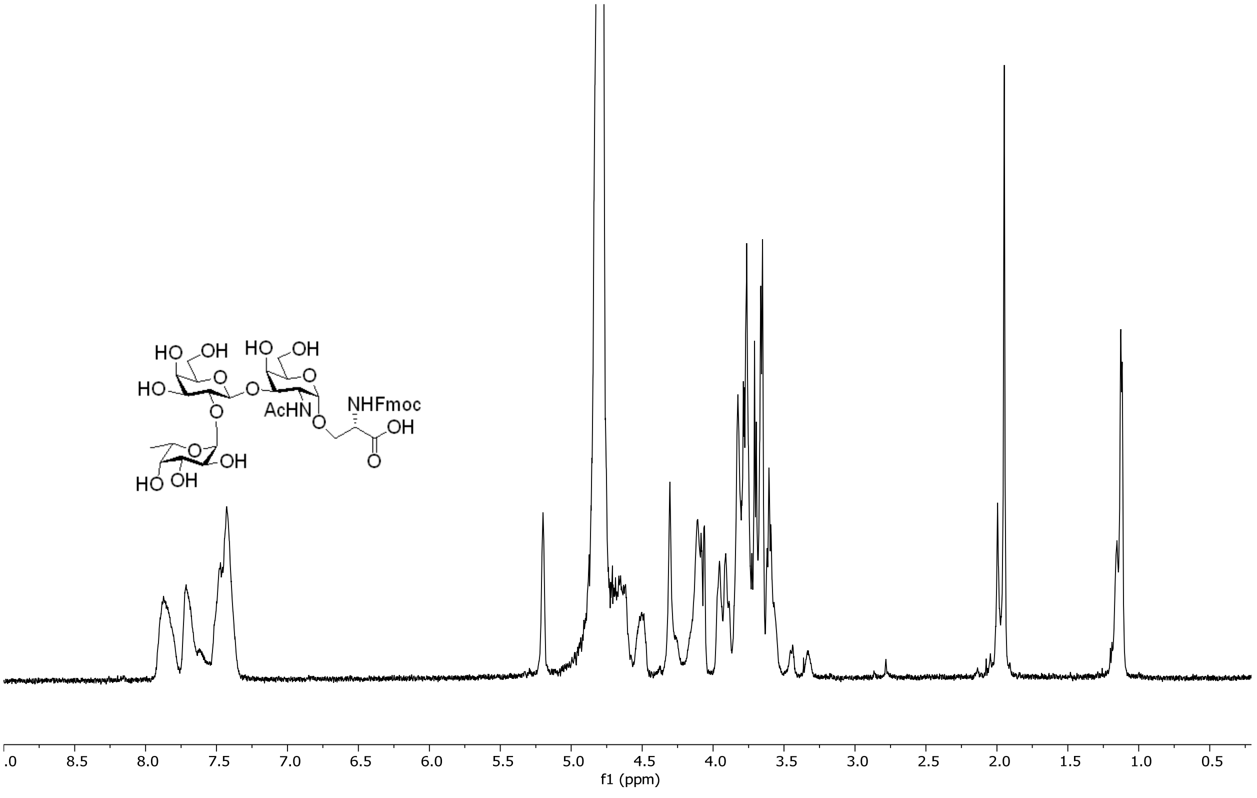

^{1}H NMR of 32

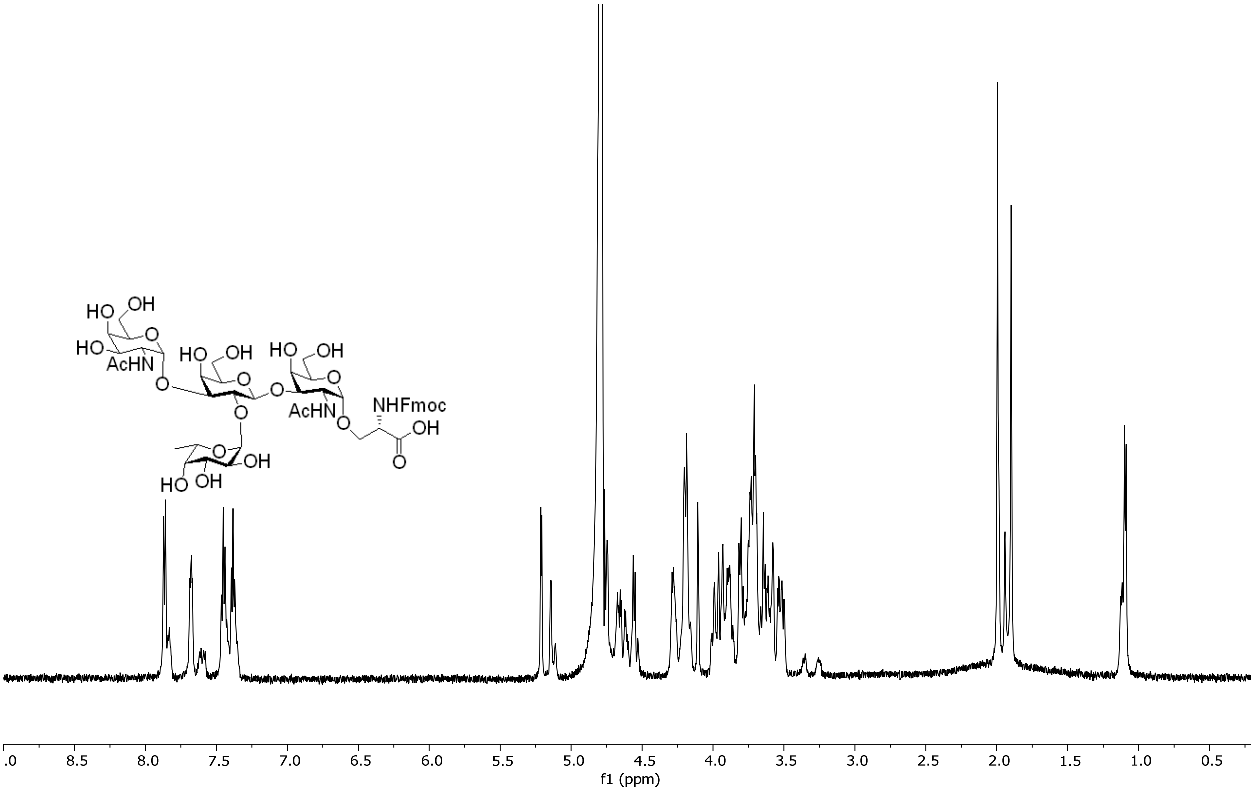

^{1}H NMR of 33

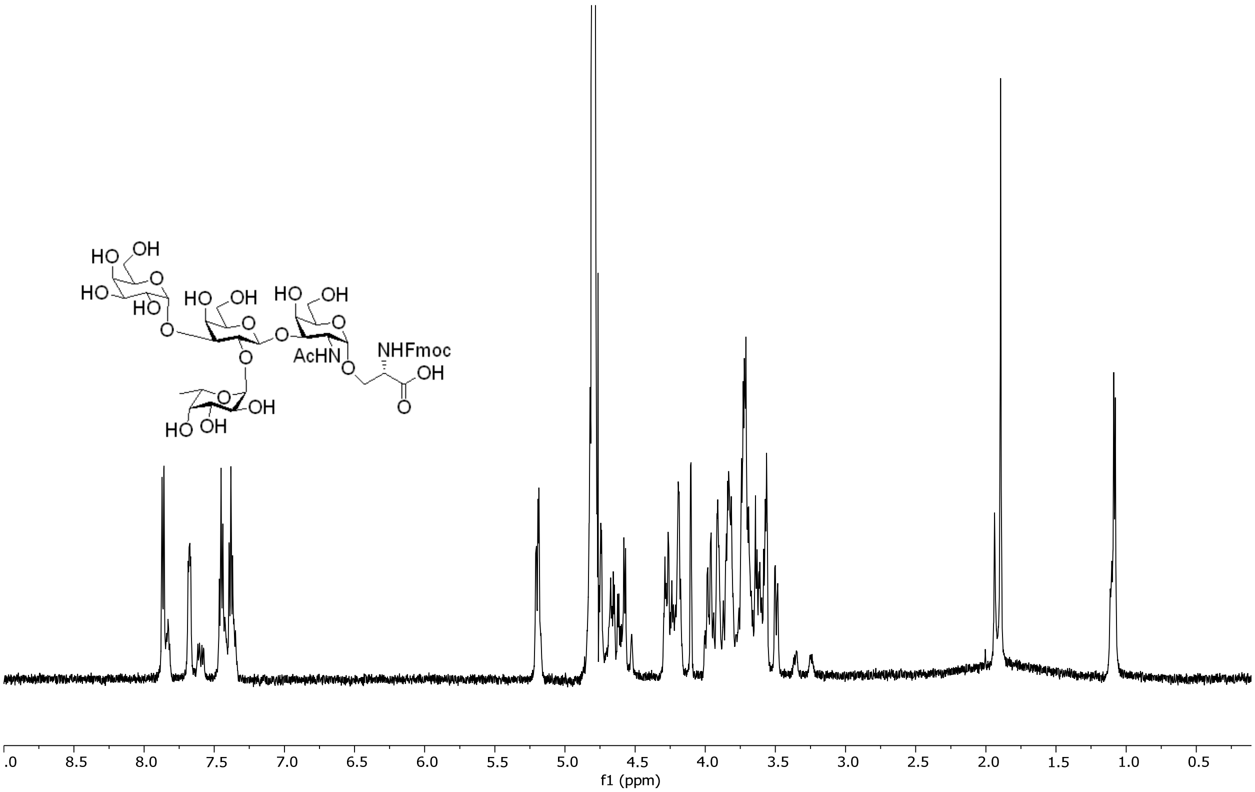

^{1}H NMR of 34

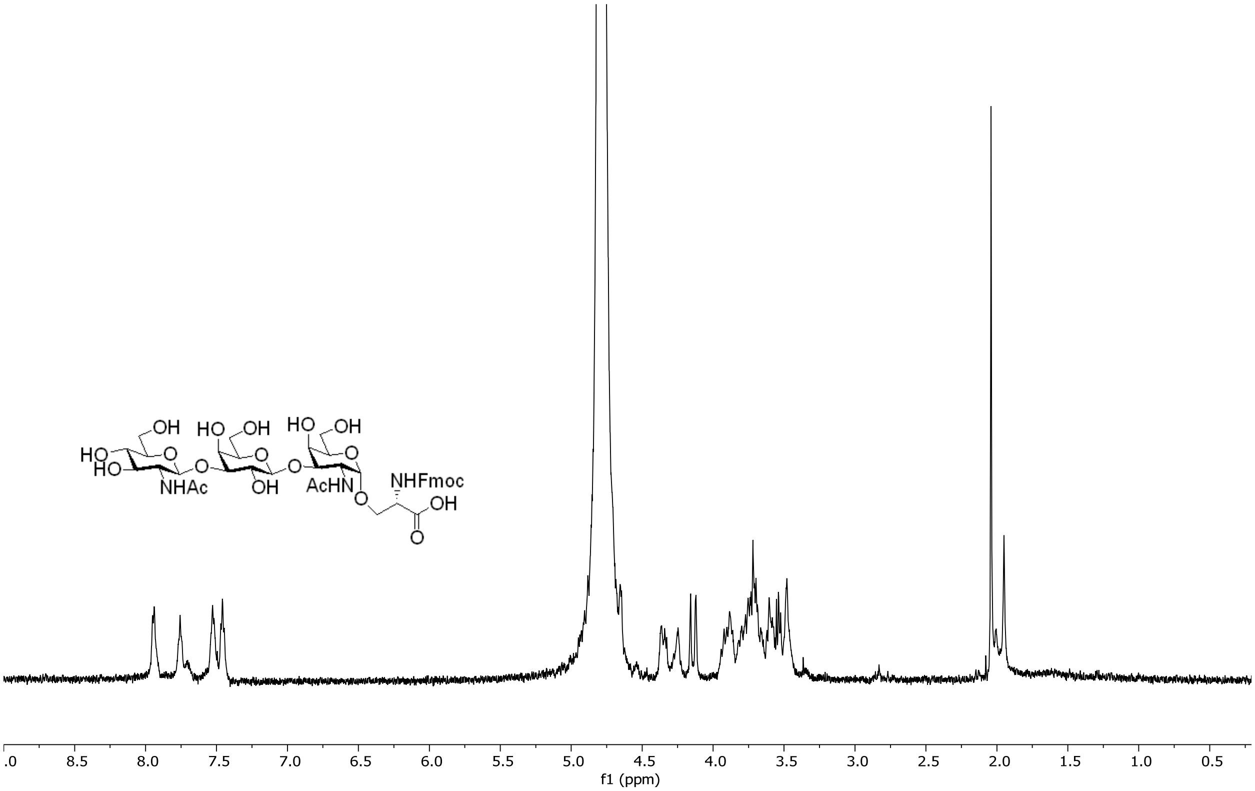

^{1}H NMR of 35

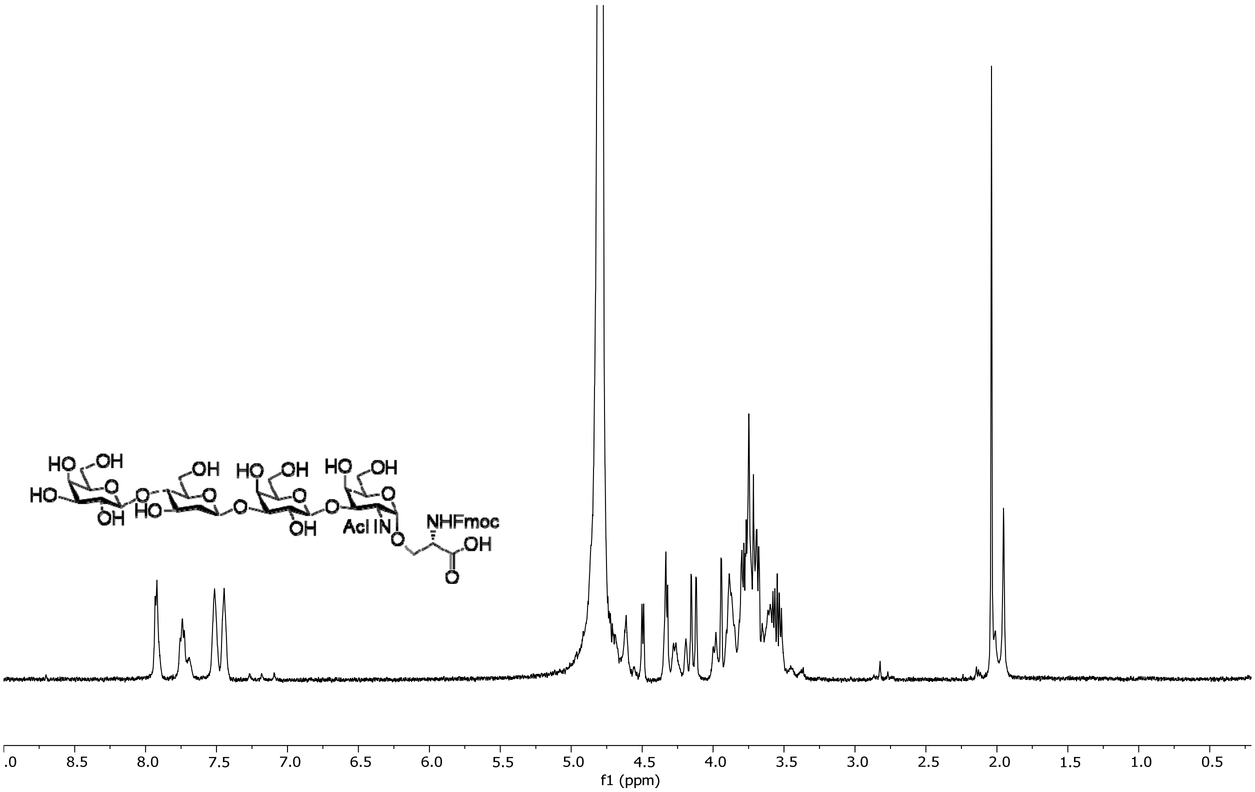

^{1}H NMR of 36

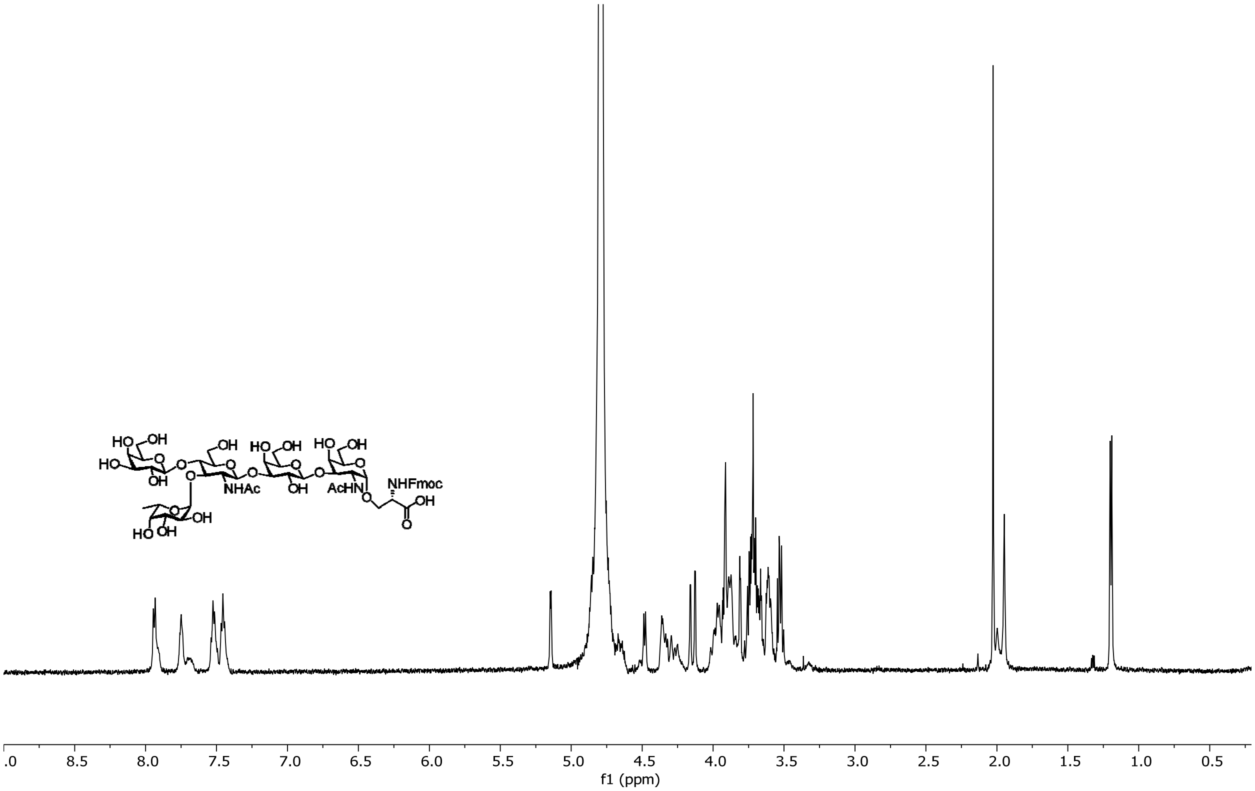

^{1}H NMR of 37

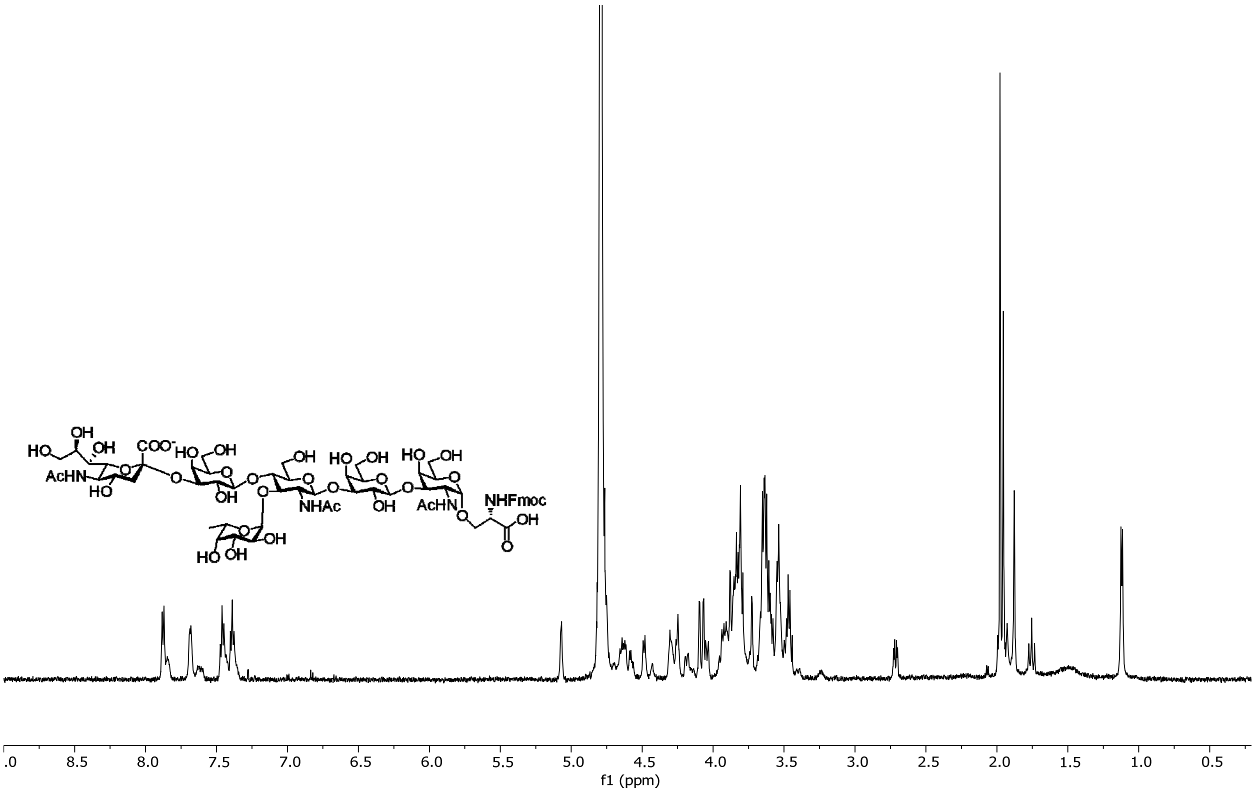

^{1}H NMR of 38

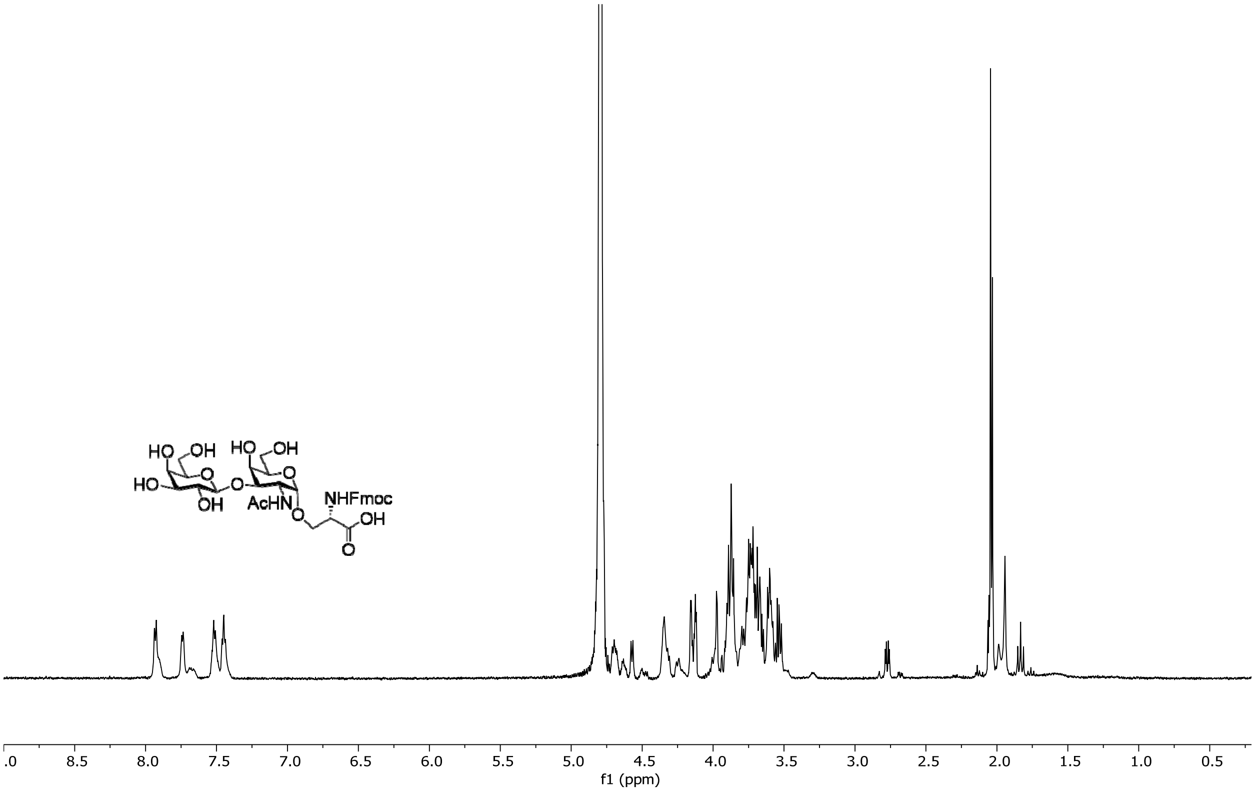

^{1}H NMR of 39

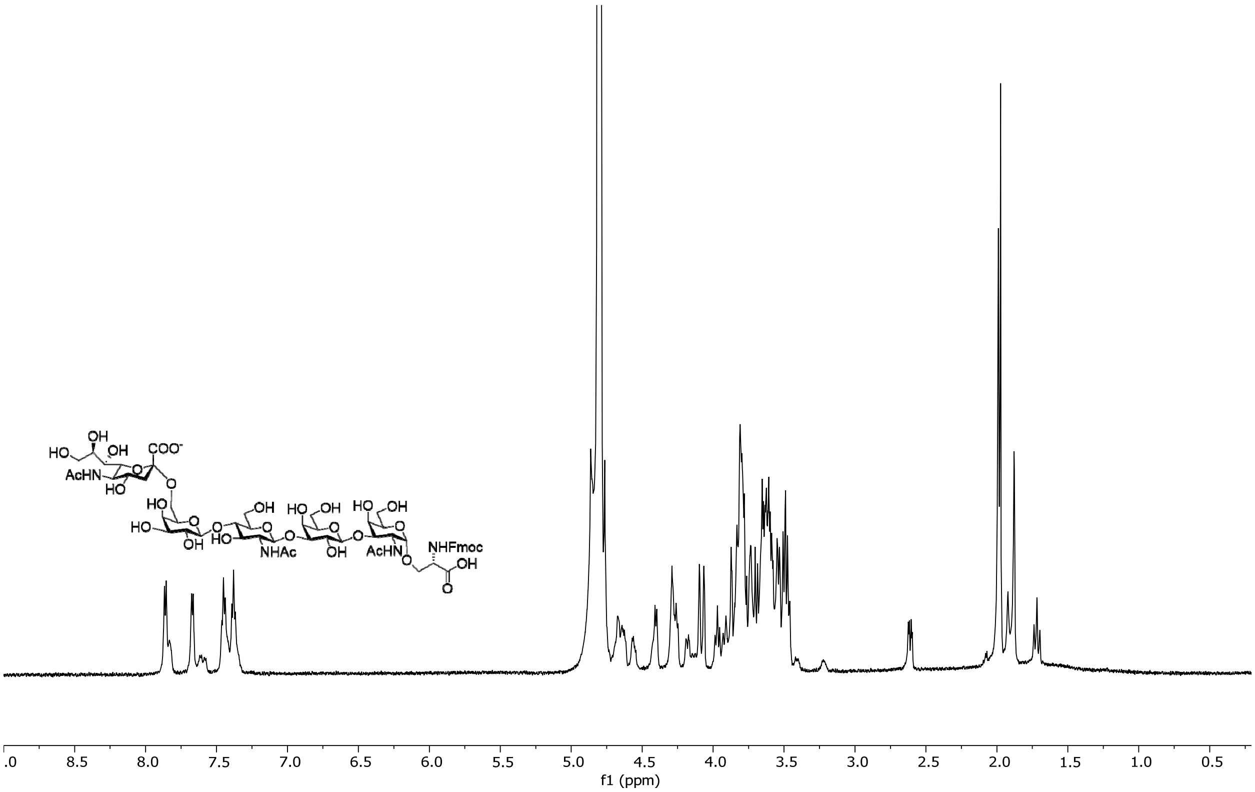

^{1}H NMR of 40

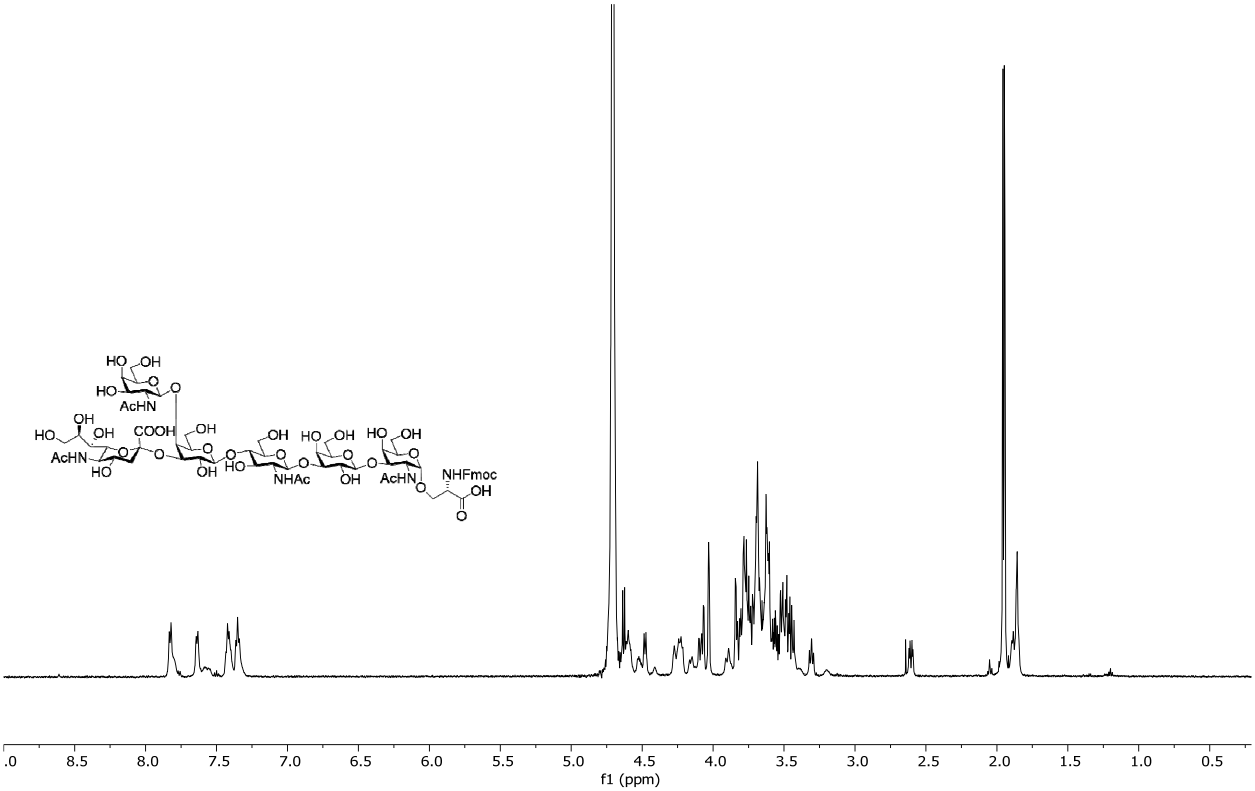

^{1}H NMR of 41

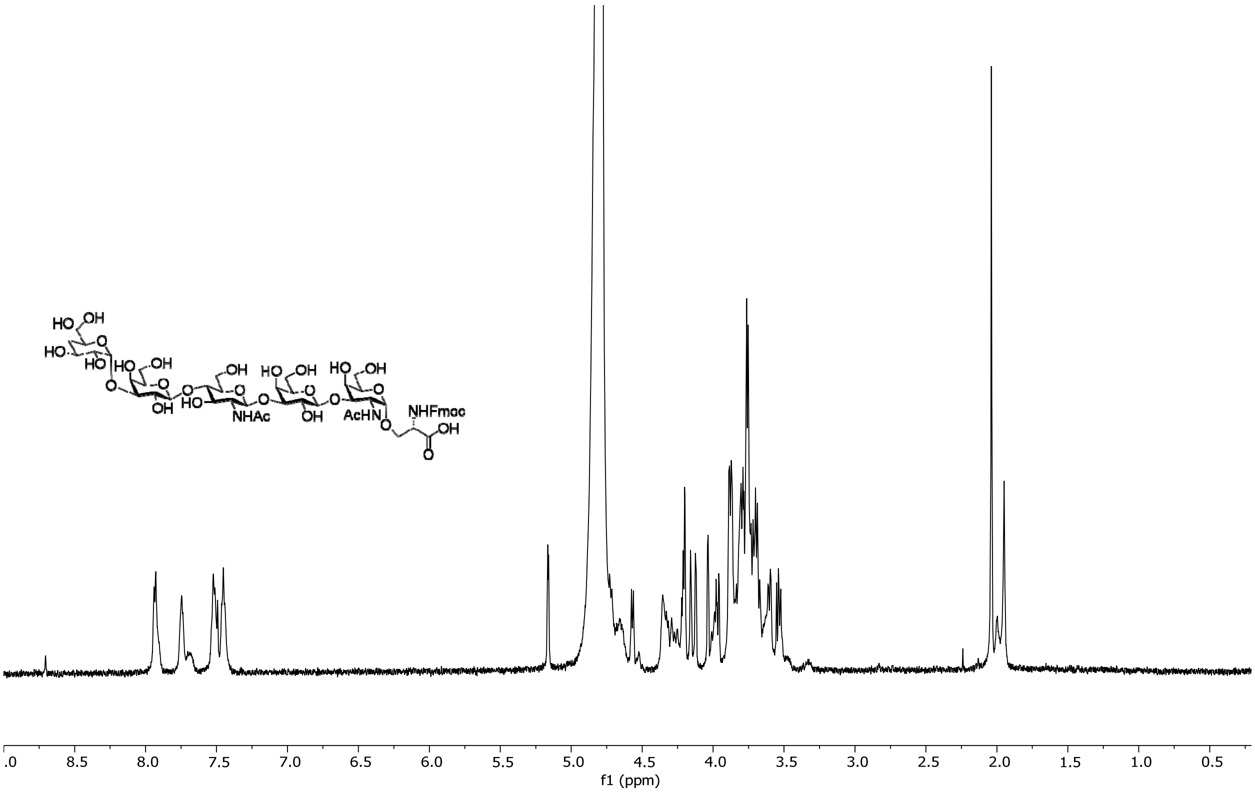

^{1}H NMR of 42

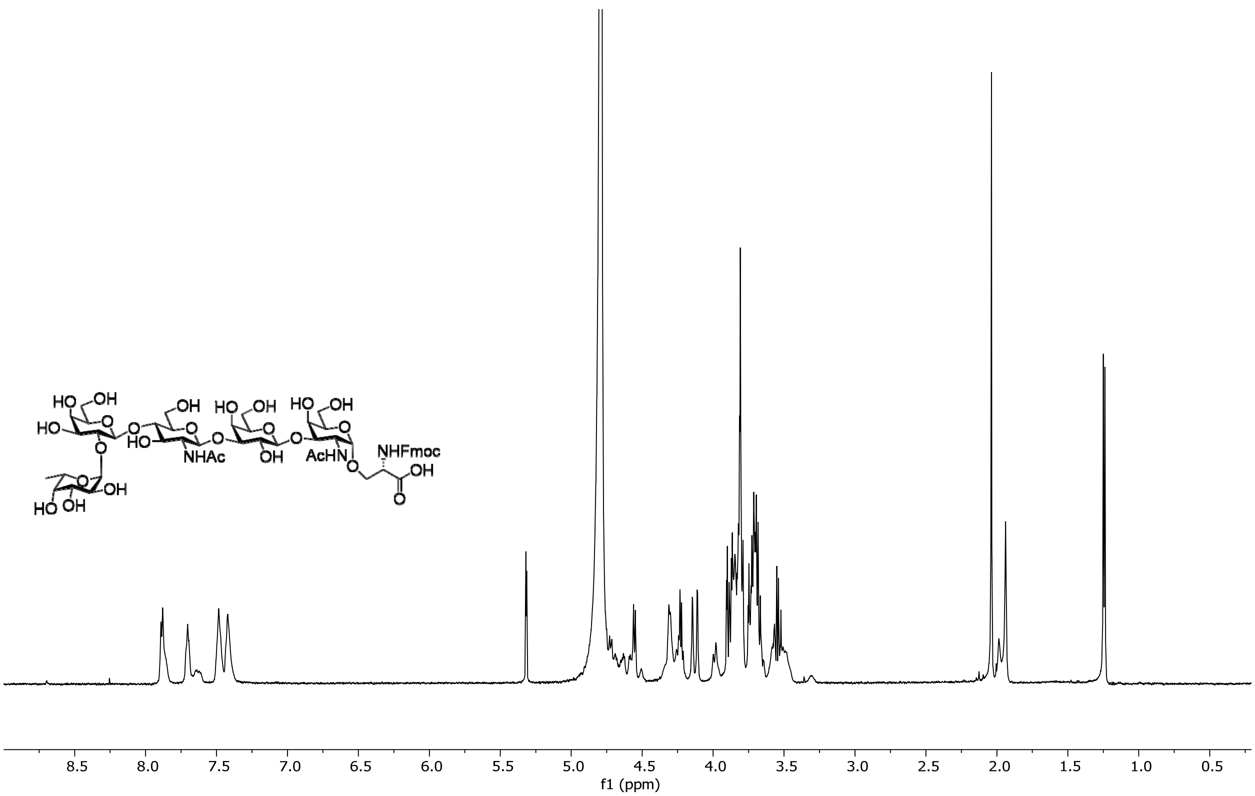

^{1}**H NMR of 43**

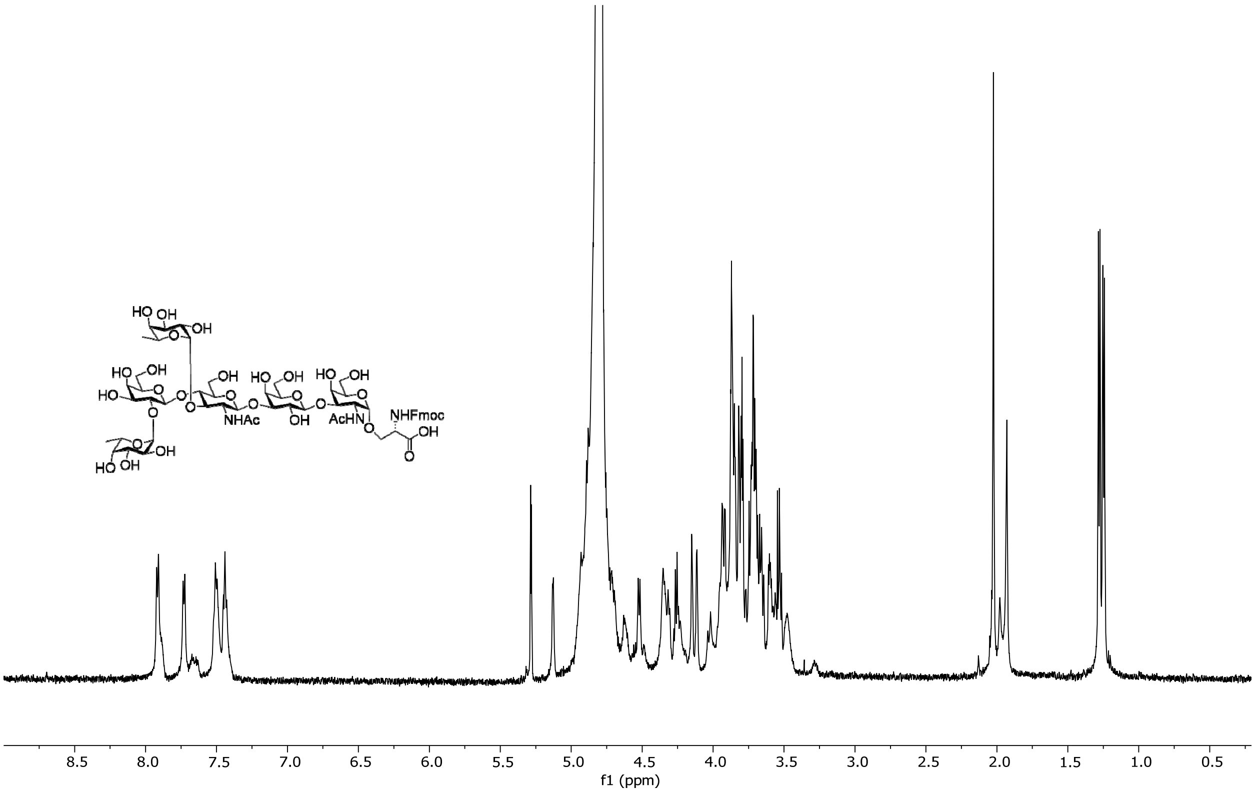

^{1}**H NMR of 44**

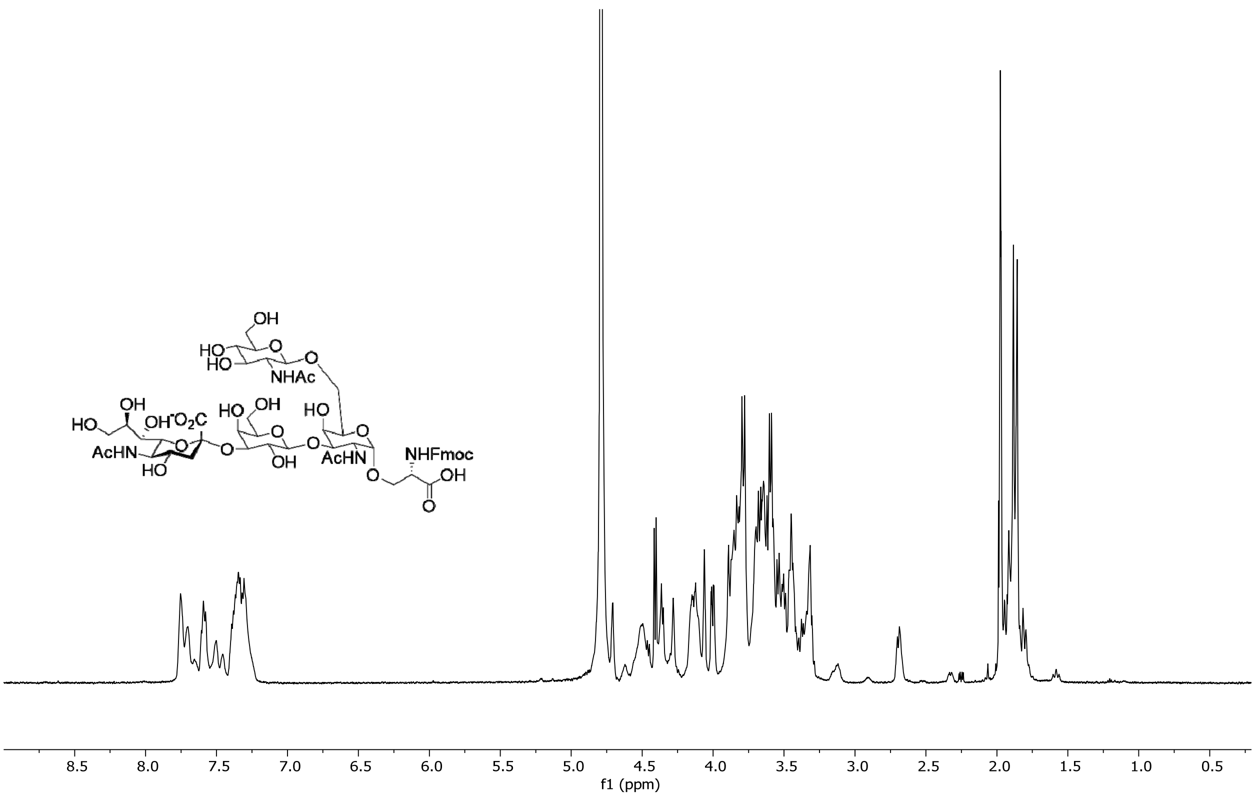

^{1}H NMR of 45

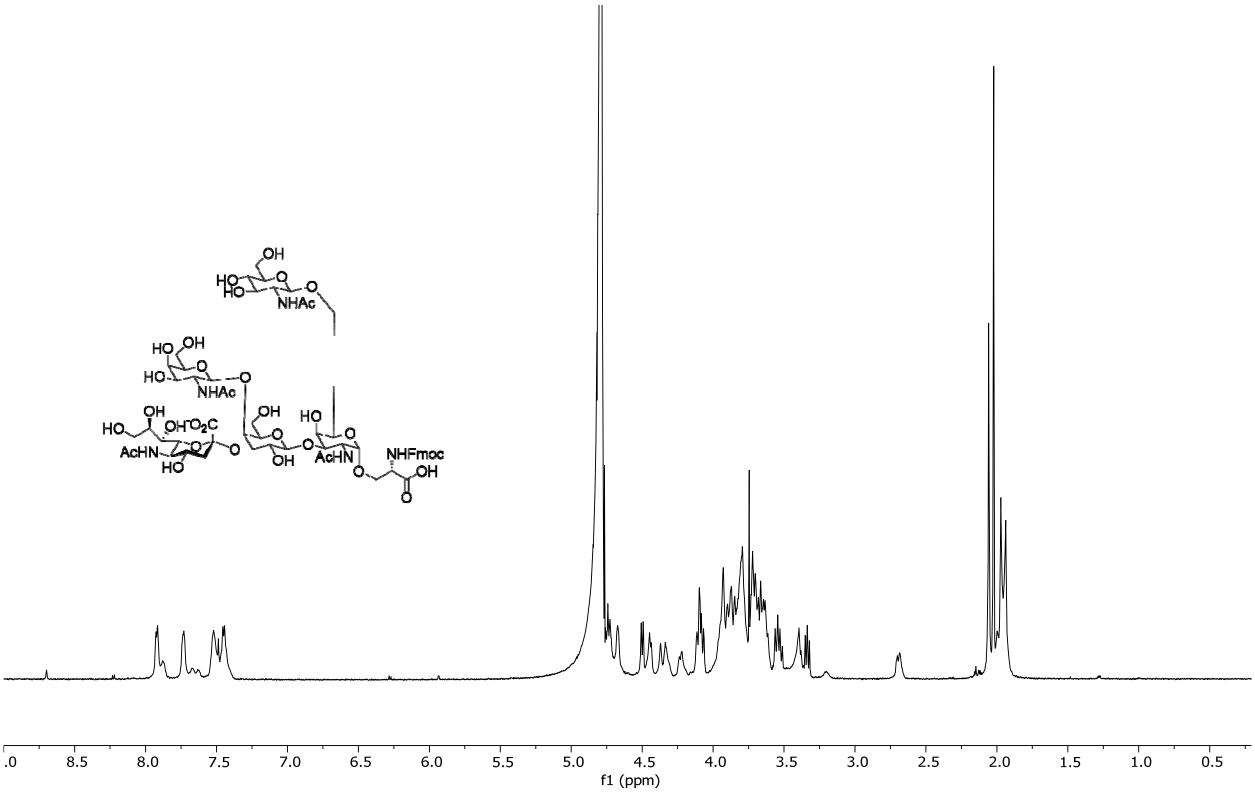

^{1}H NMR of 46

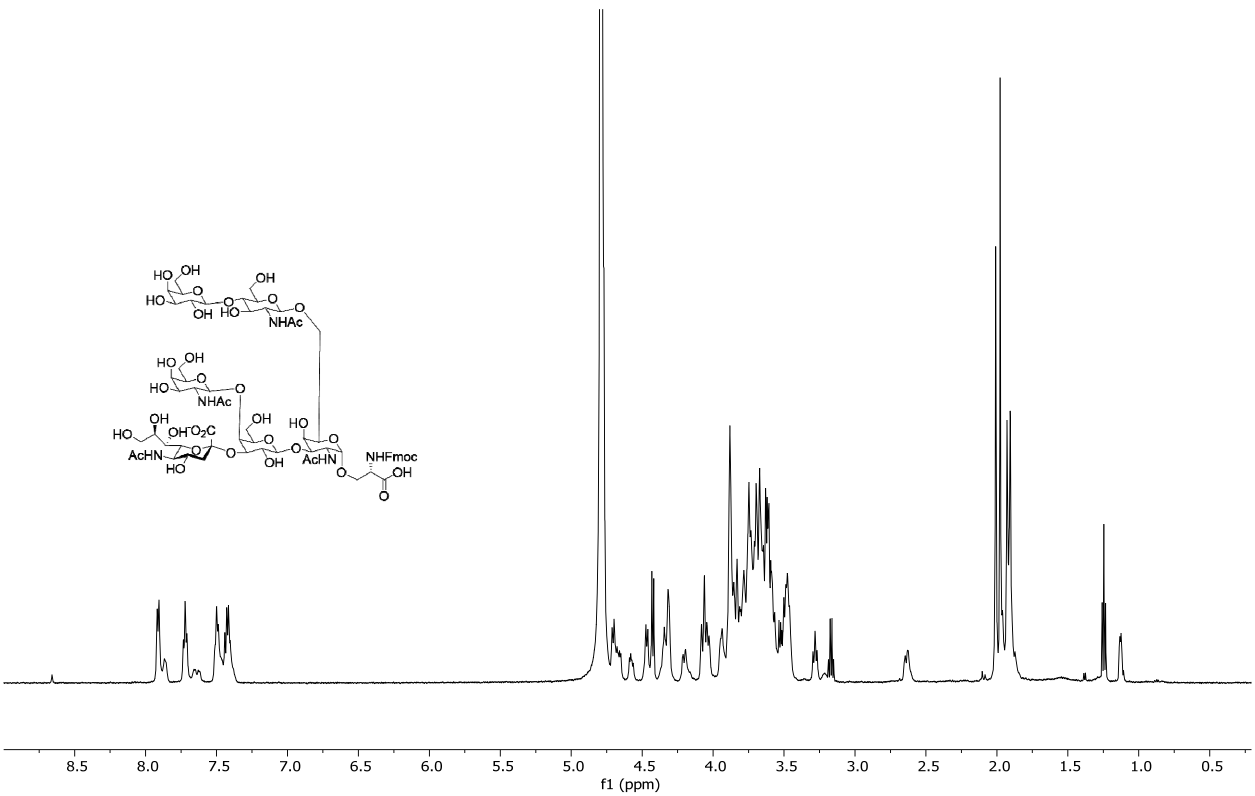

^{1}H NMR of 47

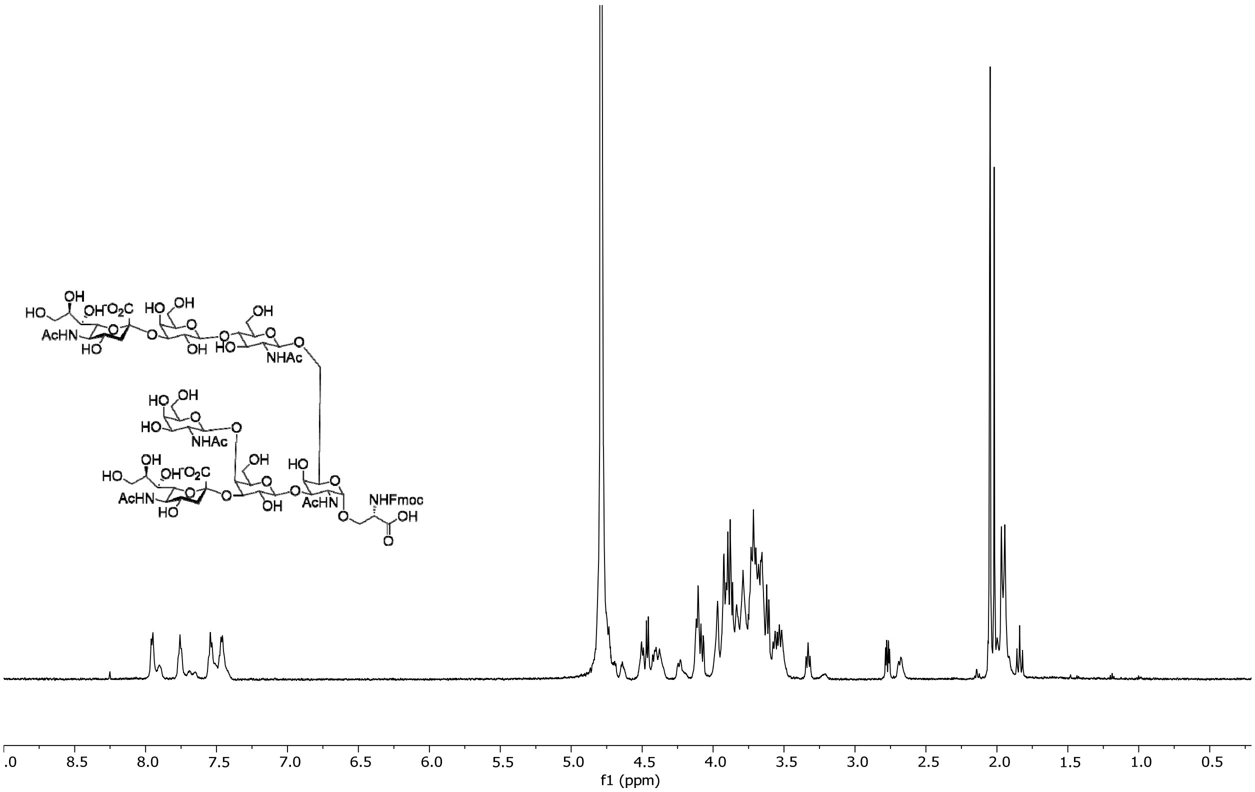

^{1}H NMR of 48

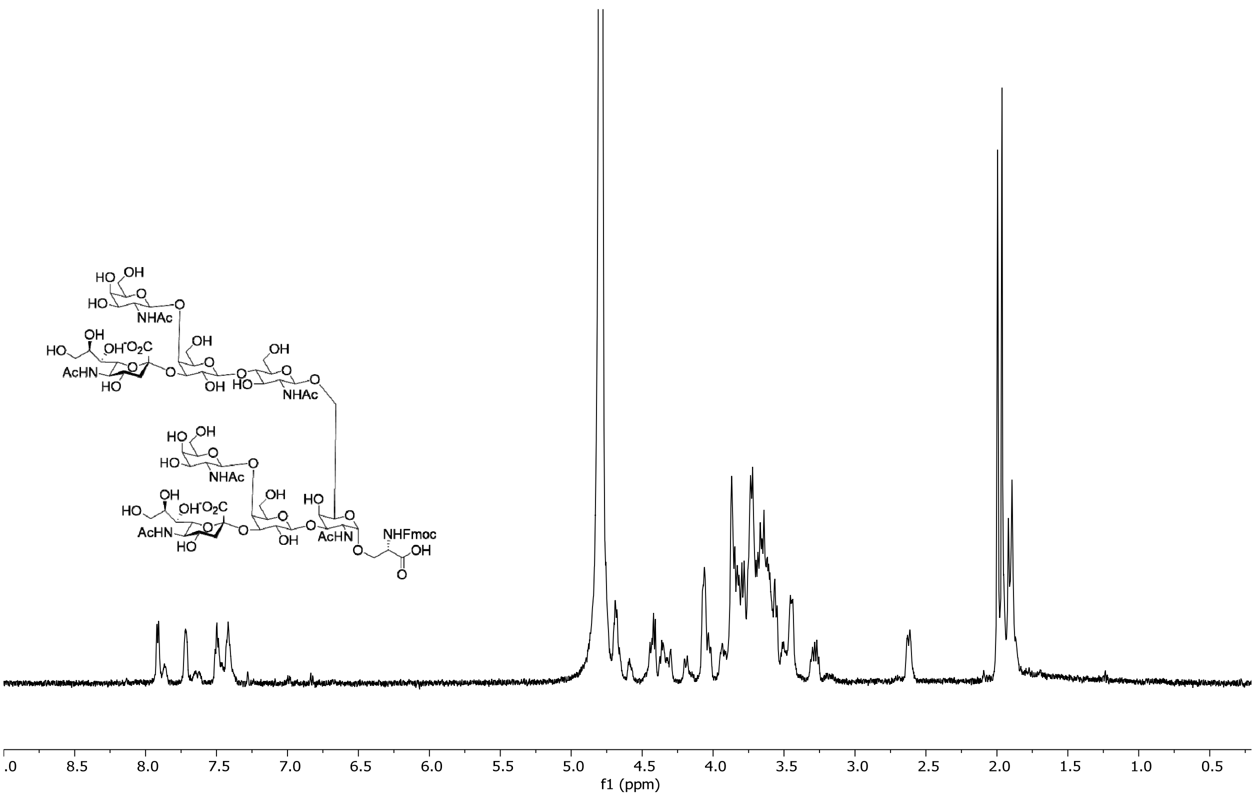

^{1}H NMR of 49

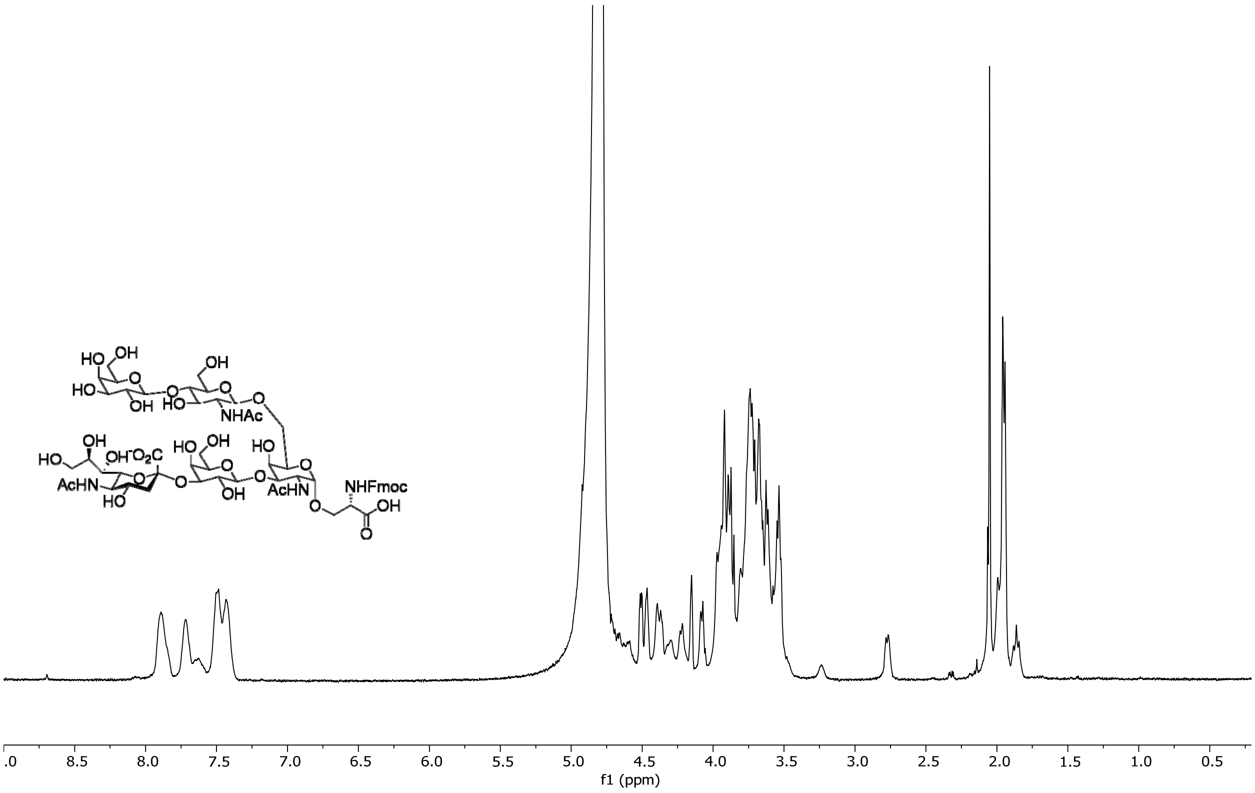

^{1}H NMR of 50

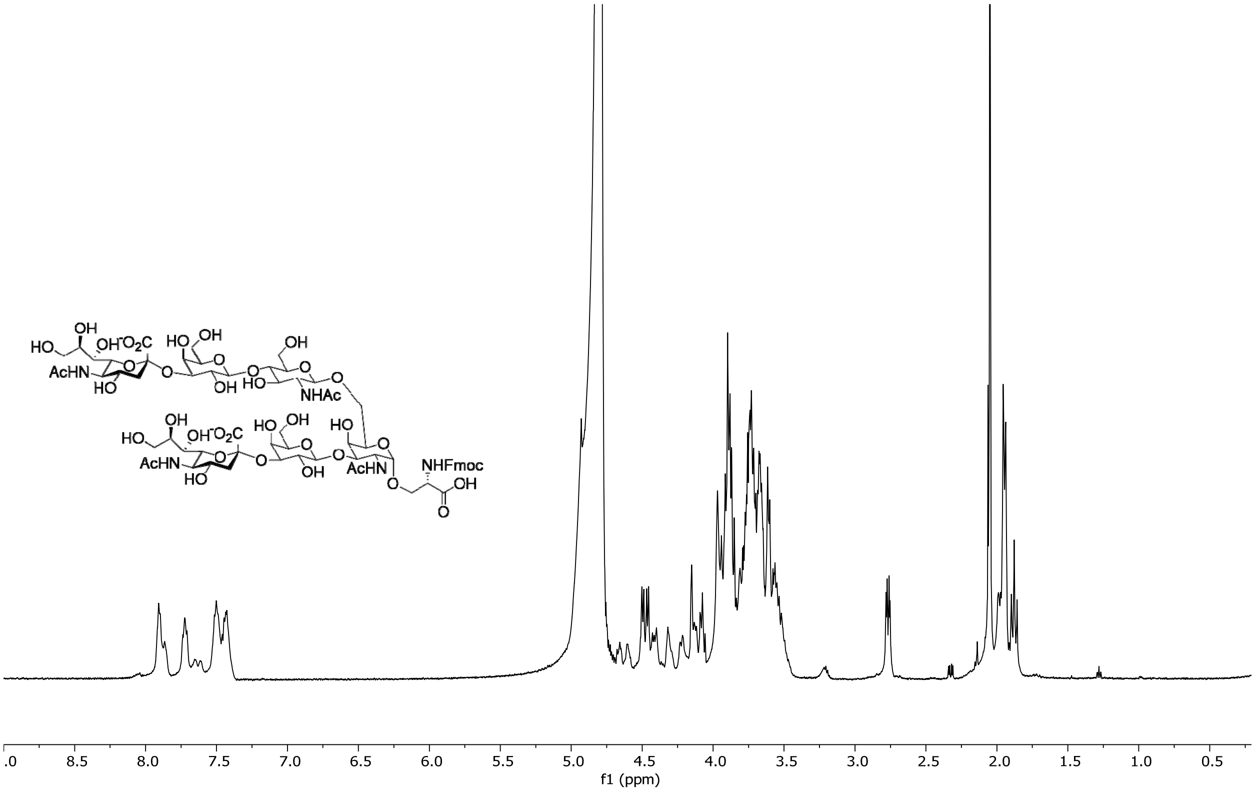

^{1}H NMR of 51

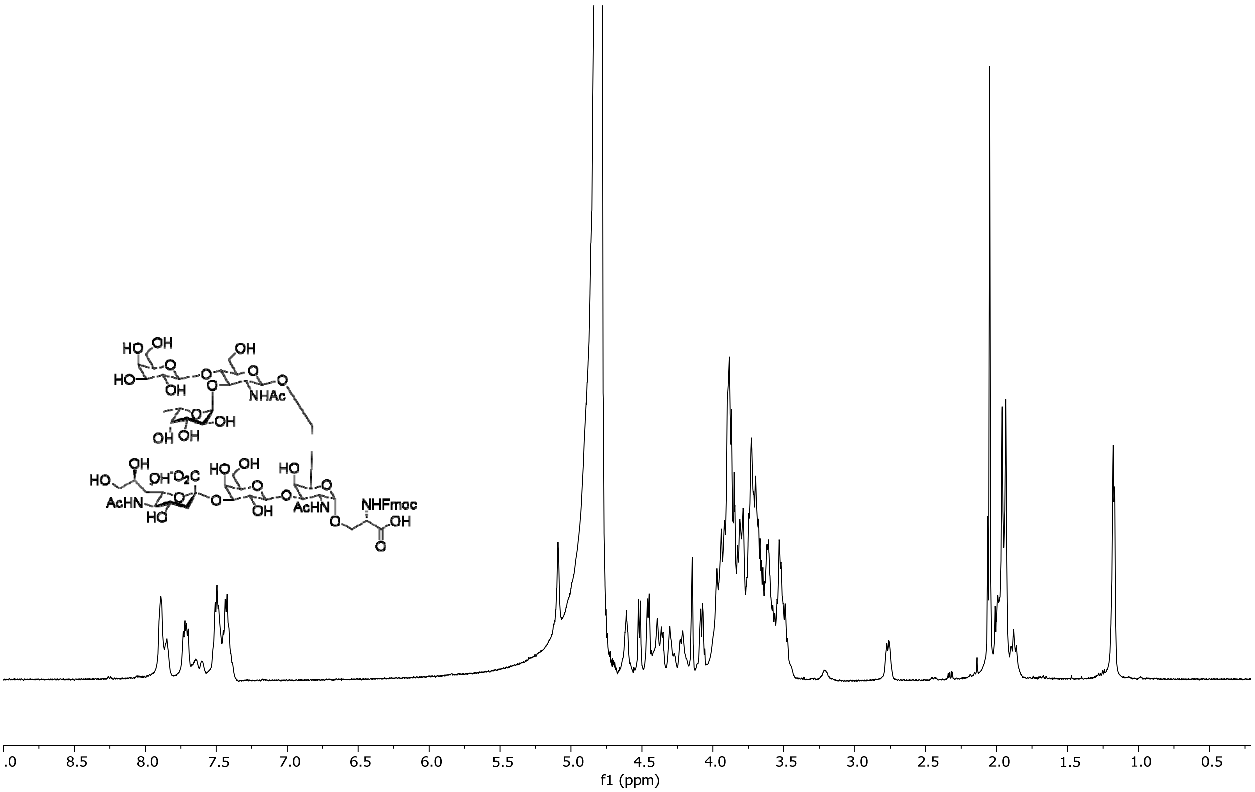

^{1}H NMR of 52

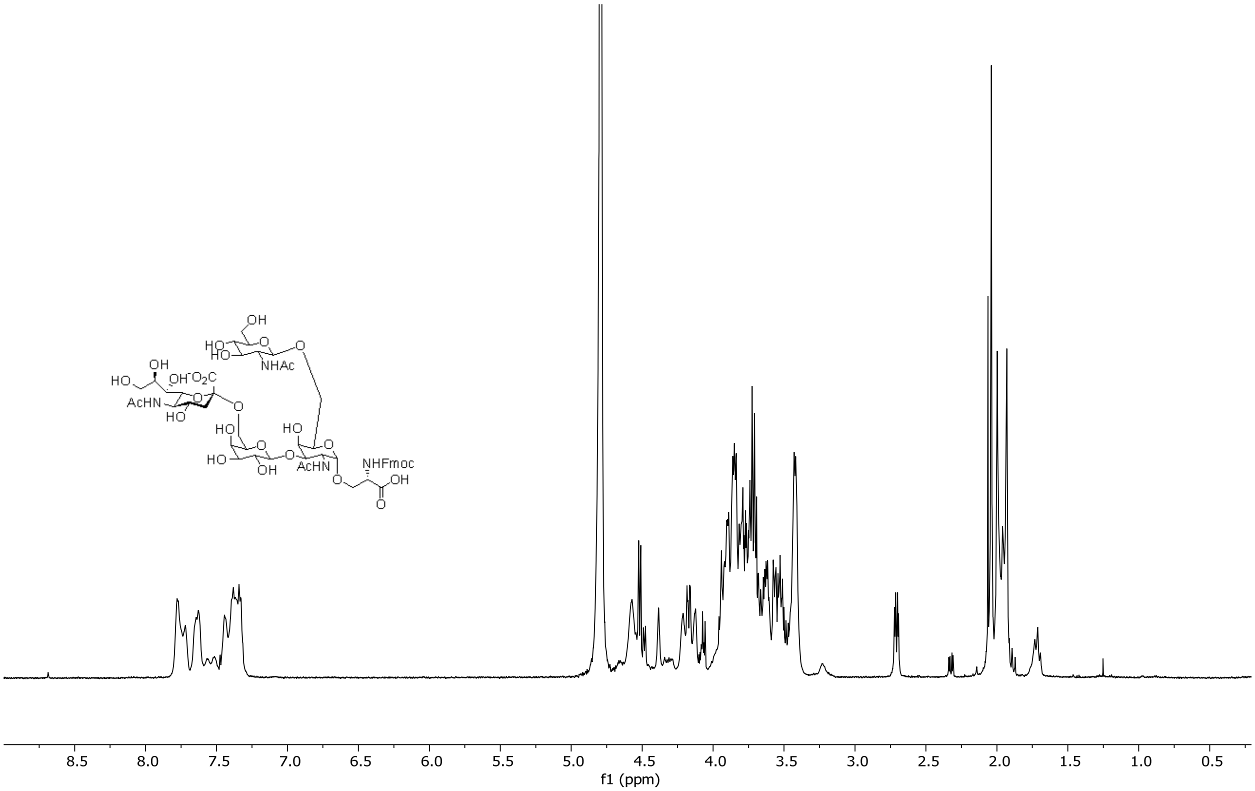

^{1}H NMR of 53

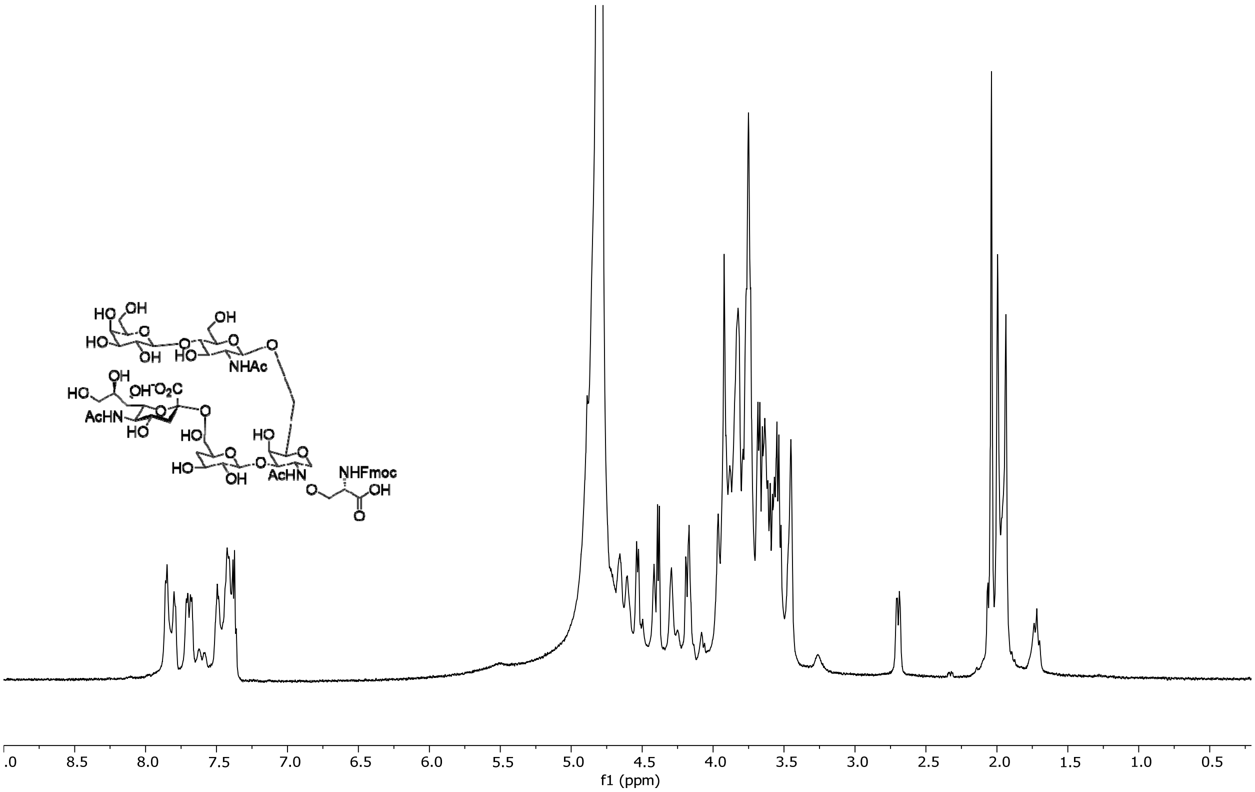

^{1}H NMR of 54

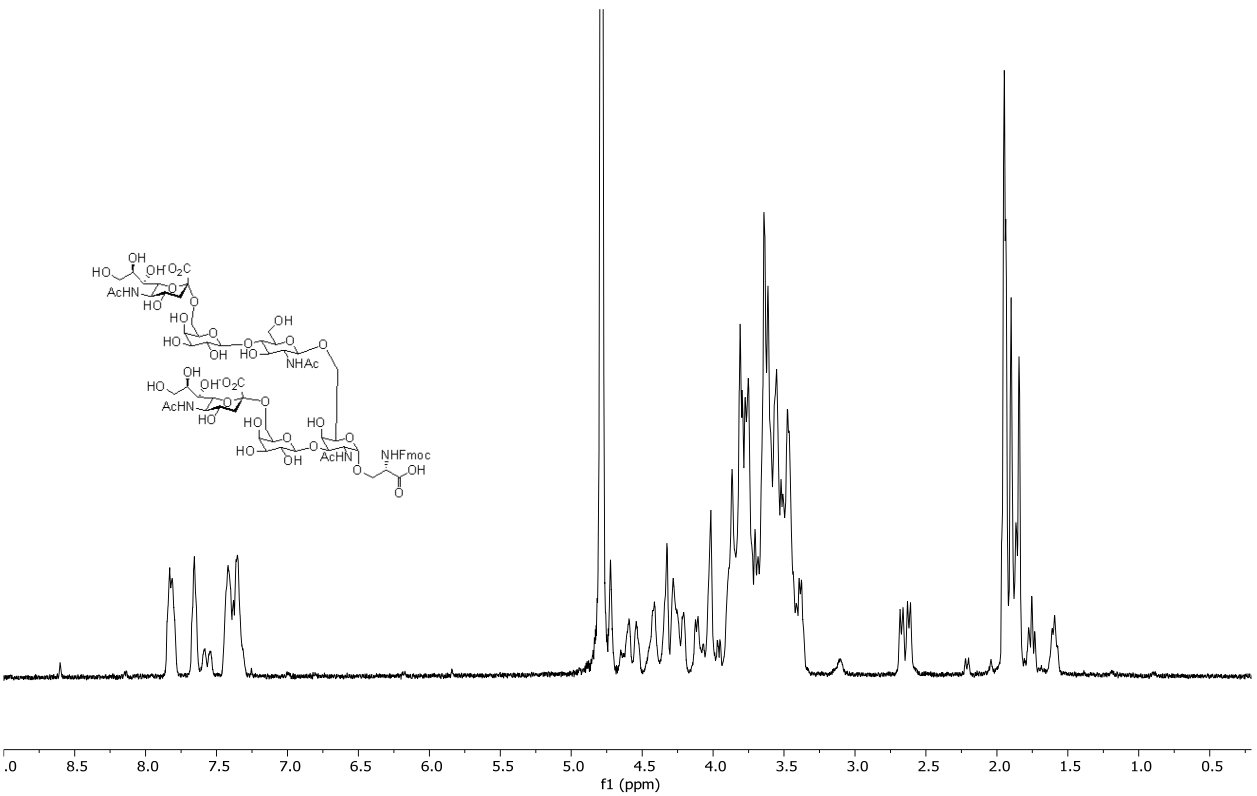

^{1}H NMR of 55

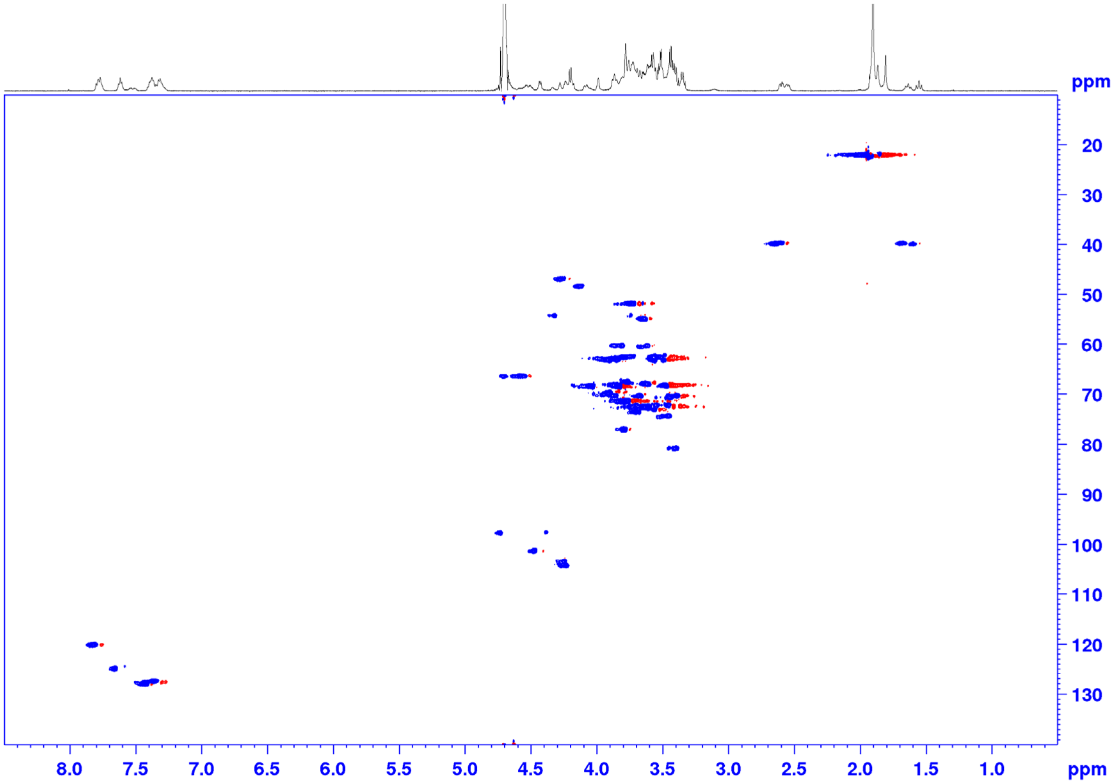

HSQC of 55

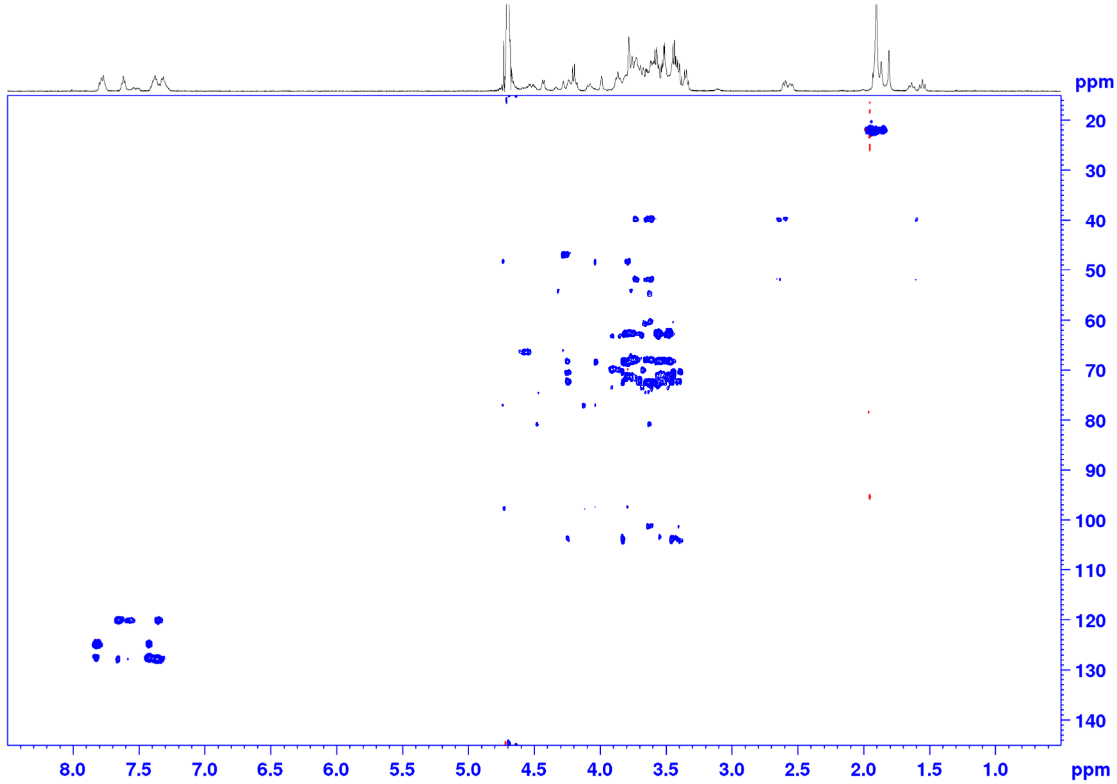

HSQC-TOCSY of 55

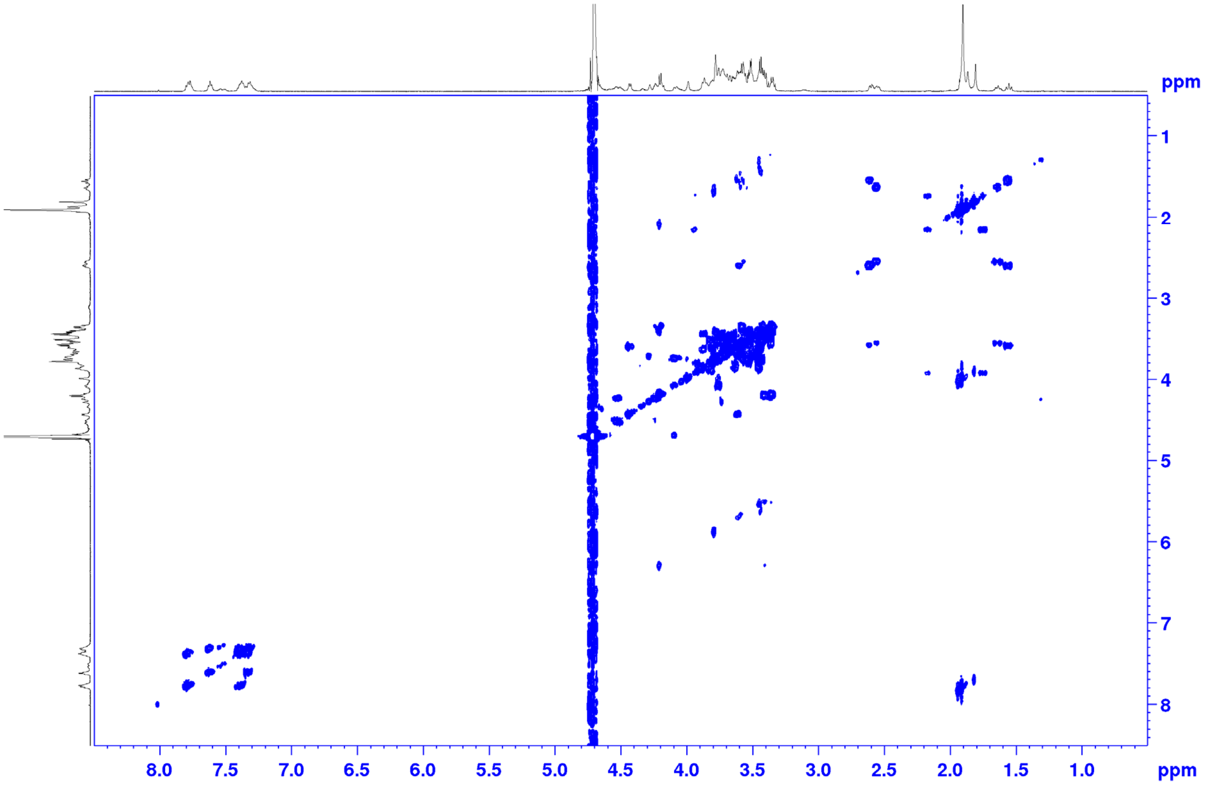

COSY of 55

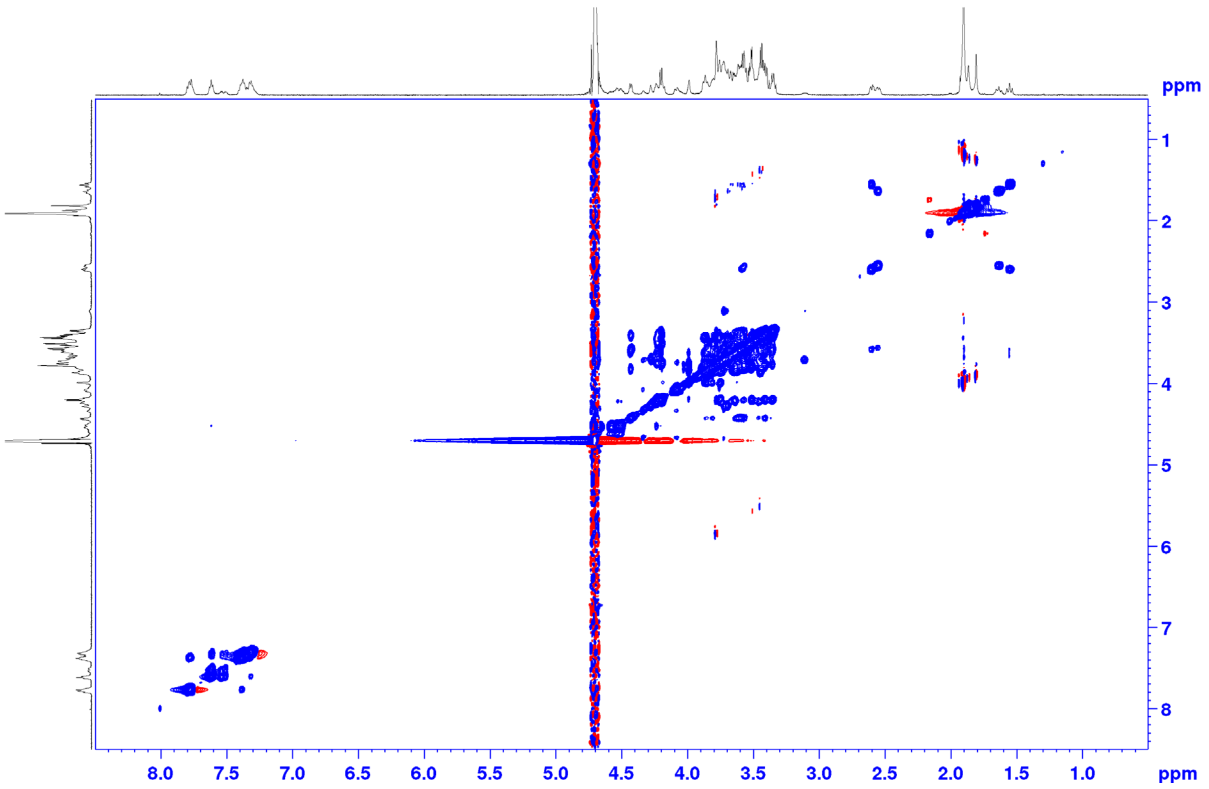

NOESY of 55

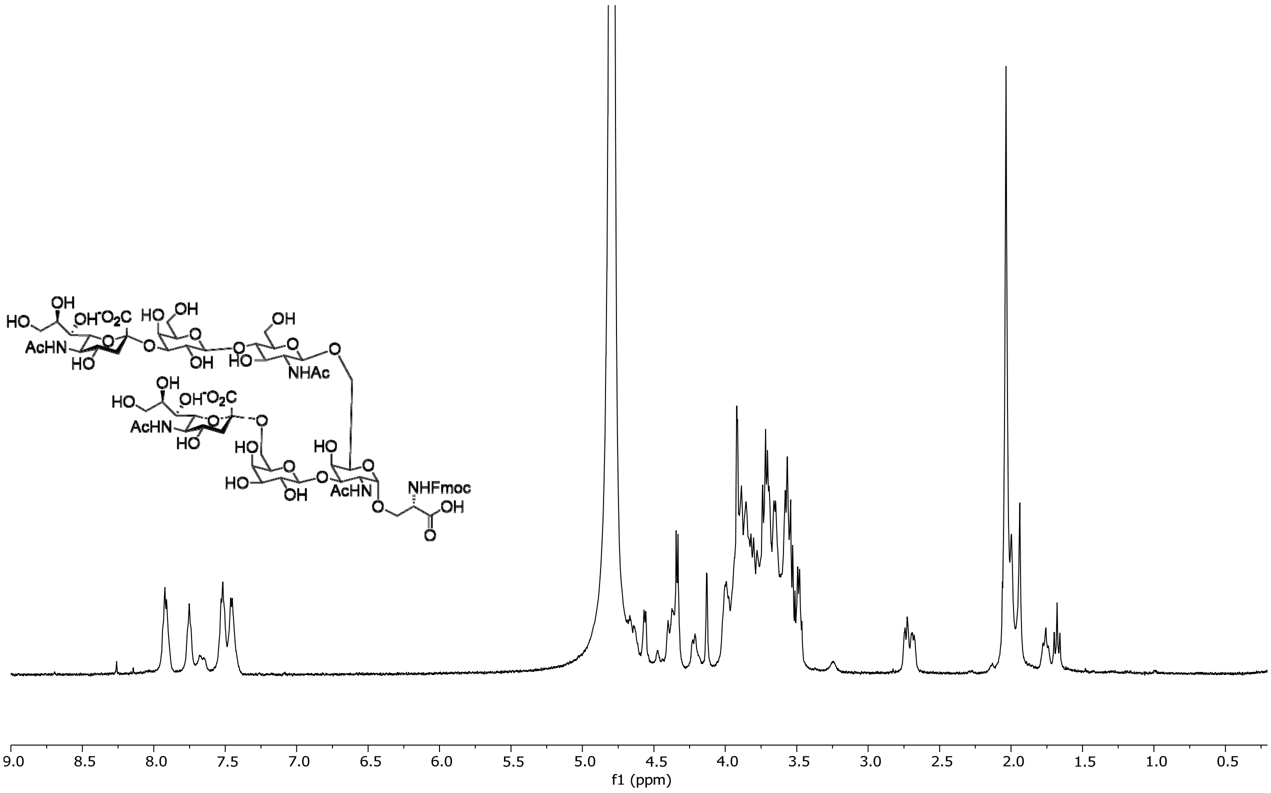

^{1}H NMR of 56

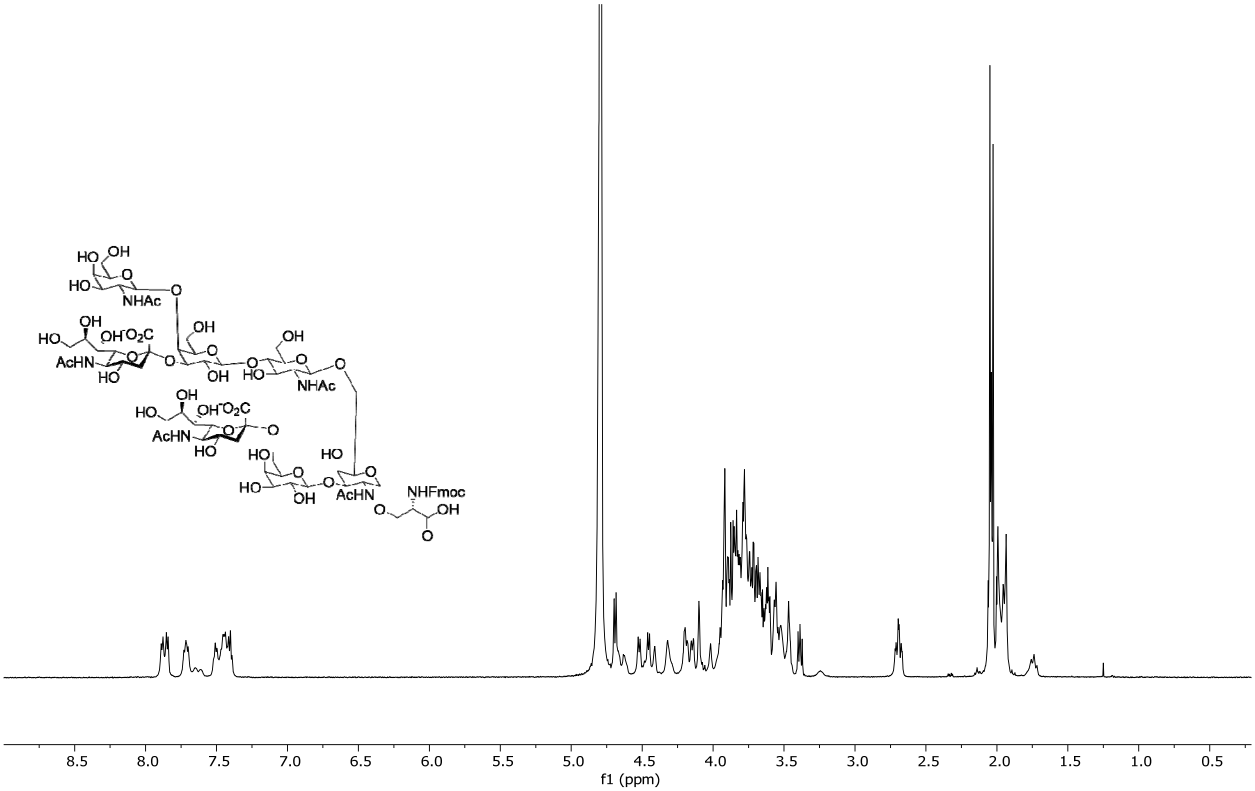

^{1}H NMR of 57

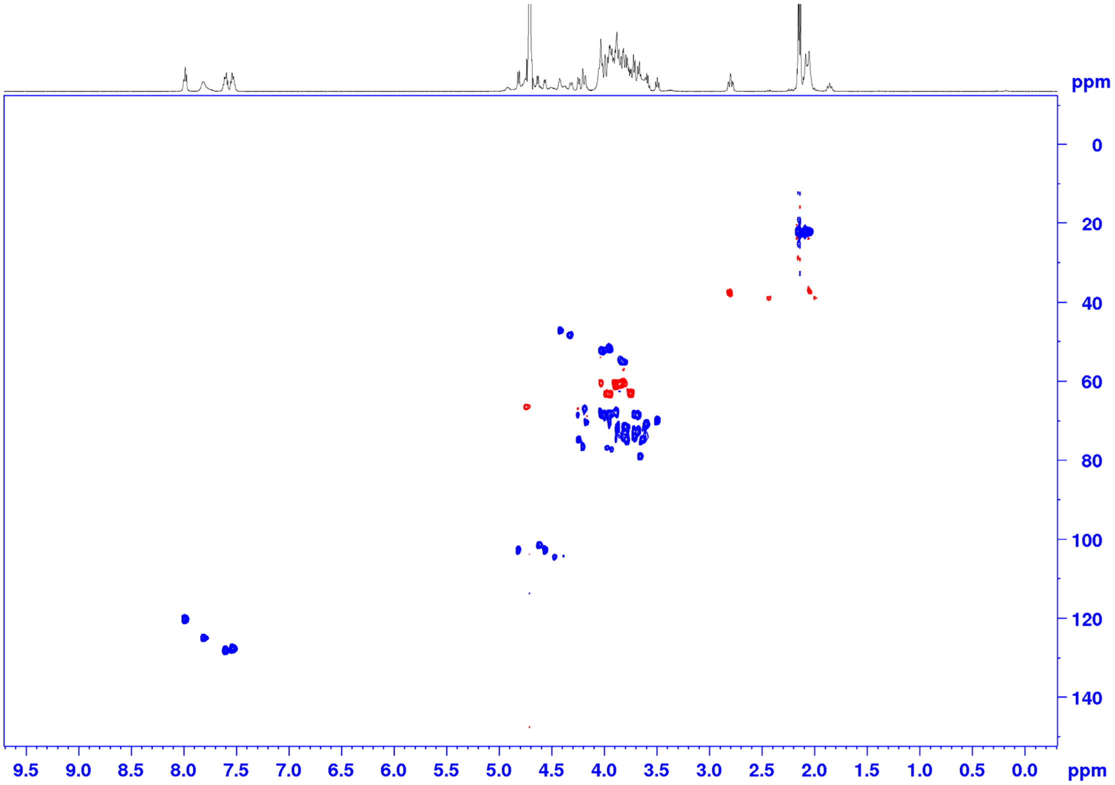

HSQC of 57

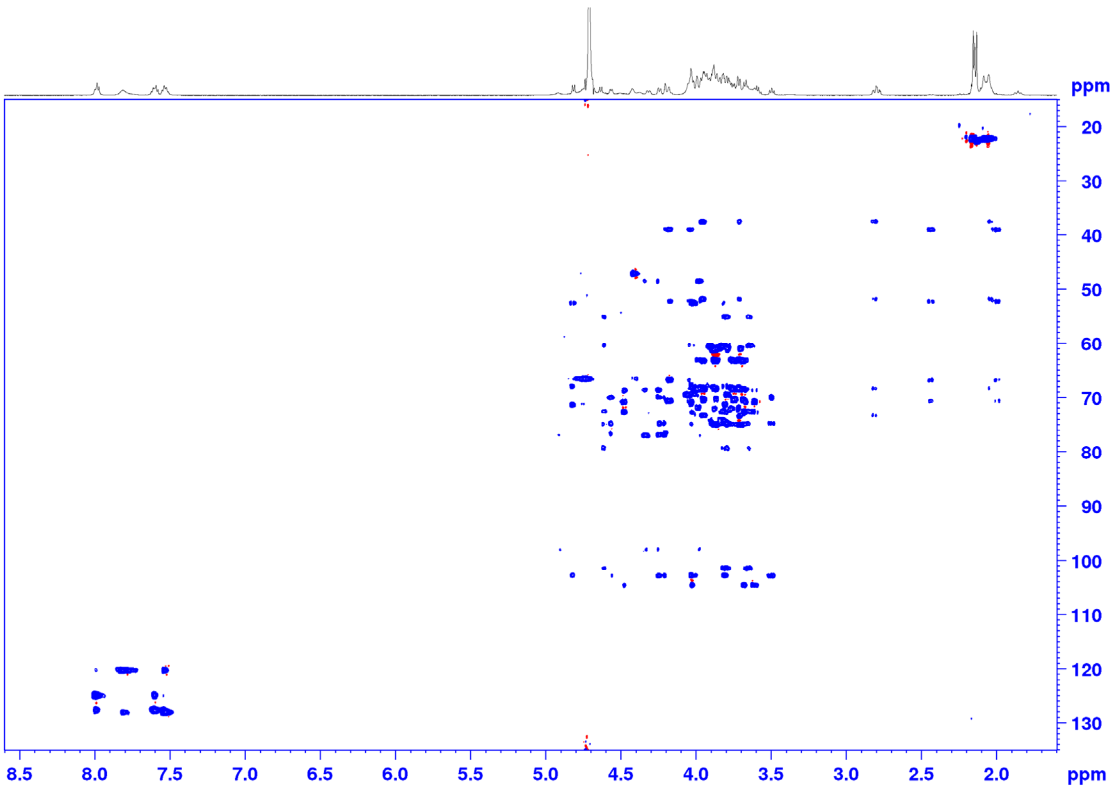

HSQC-TOCSY of 57

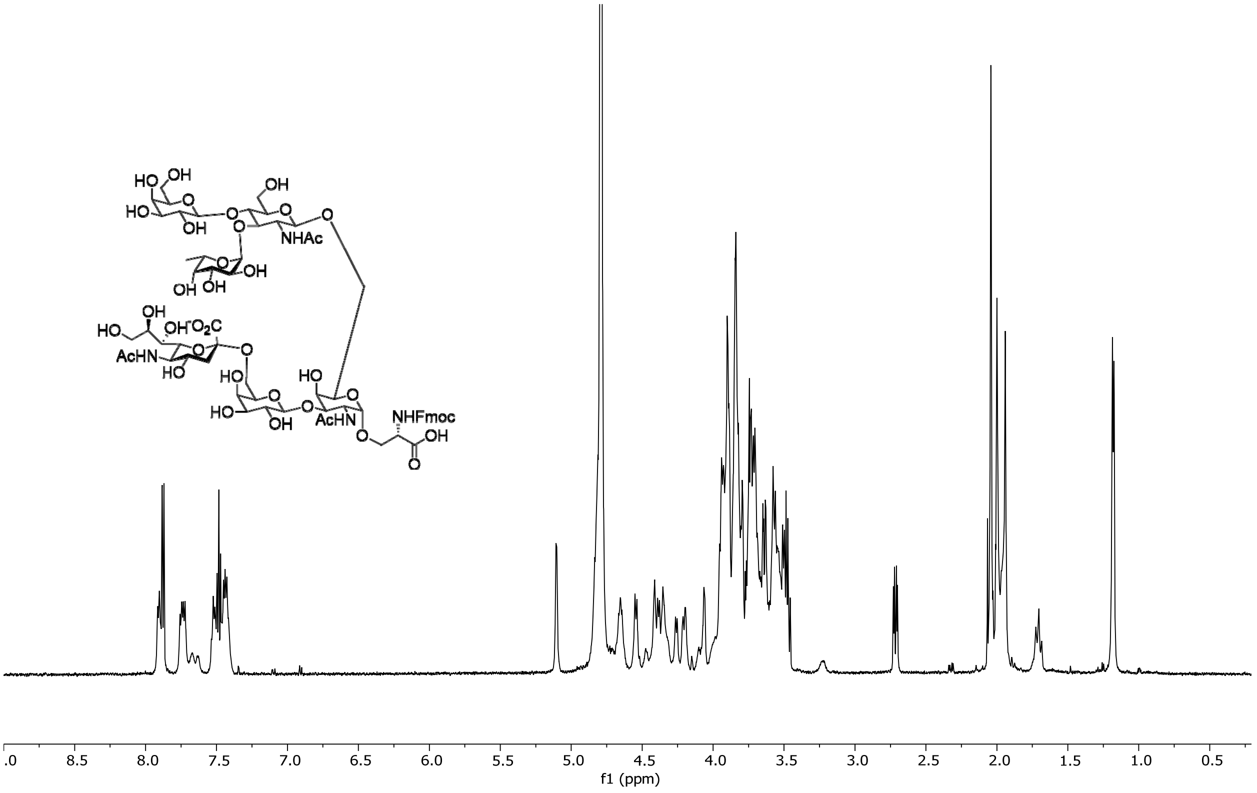

^{1}H NMR of 58

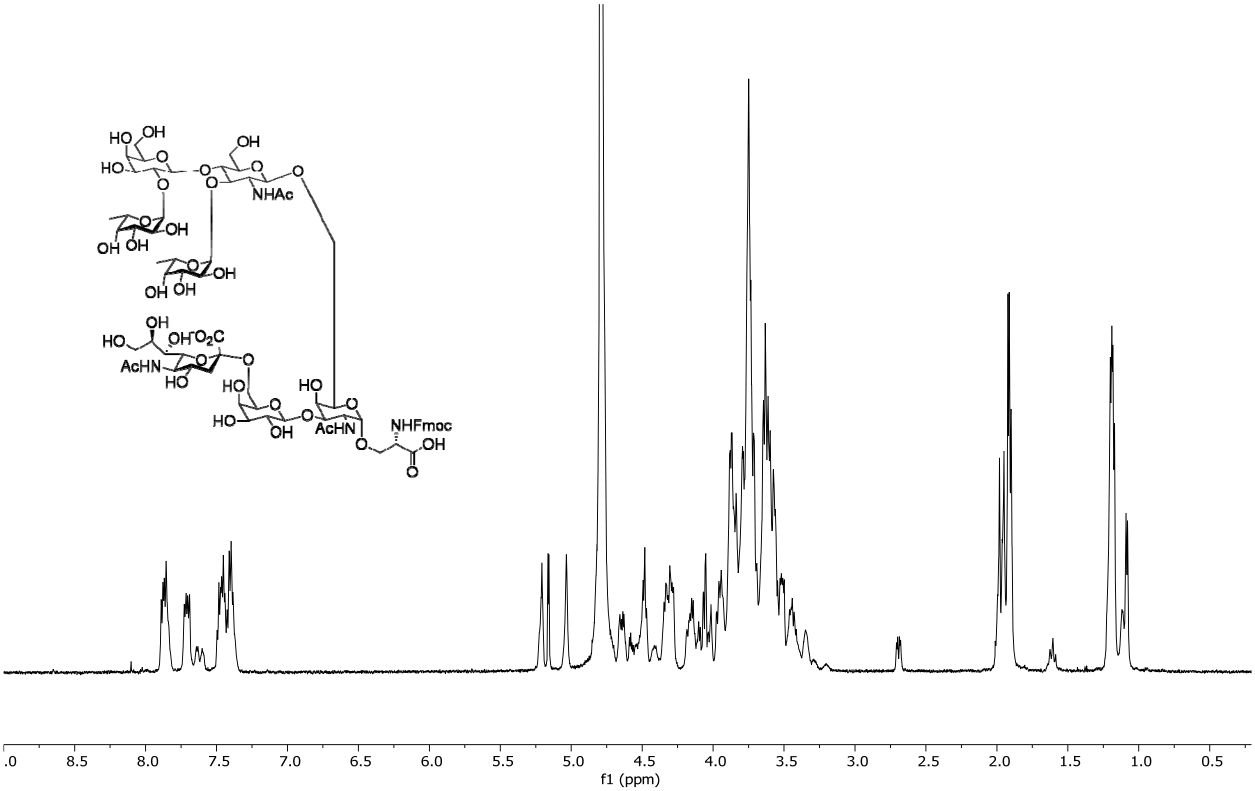

^{1}H NMR of 59

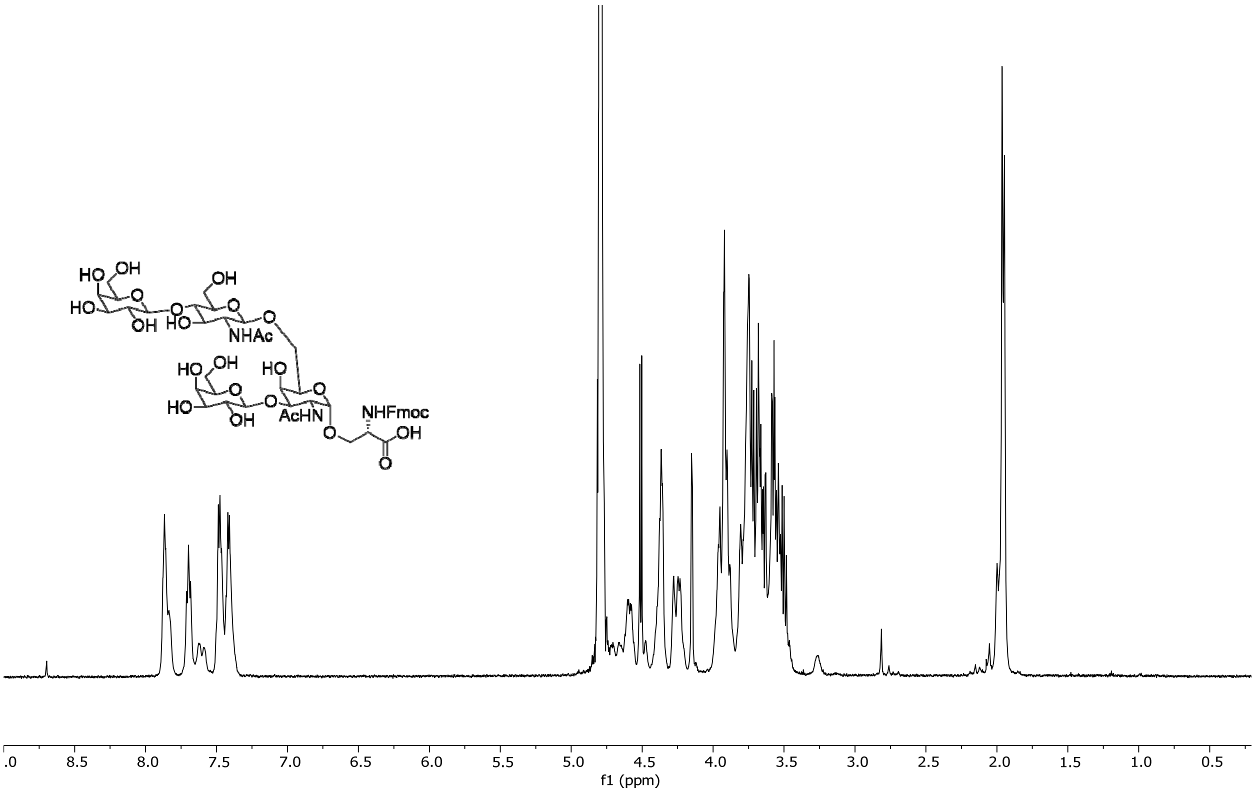

^{1}H NMR of 60

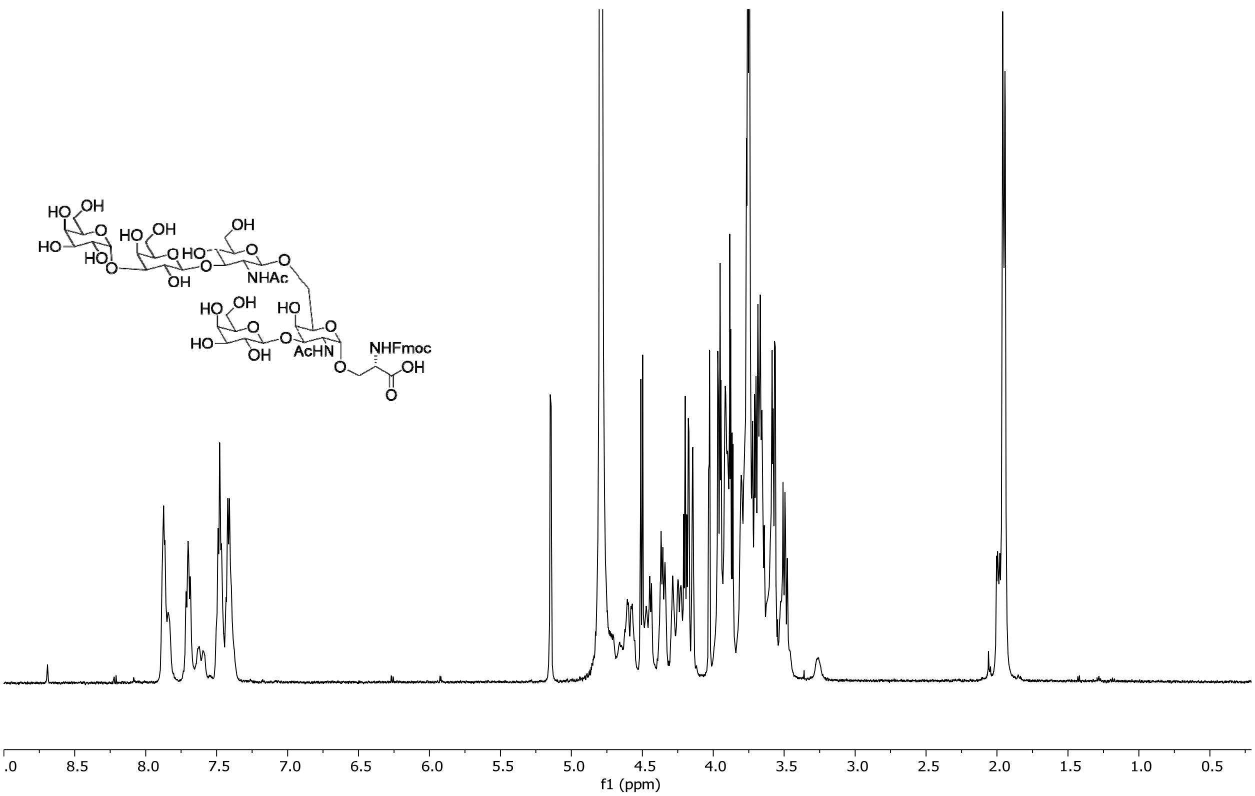

^{1}H NMR of 61

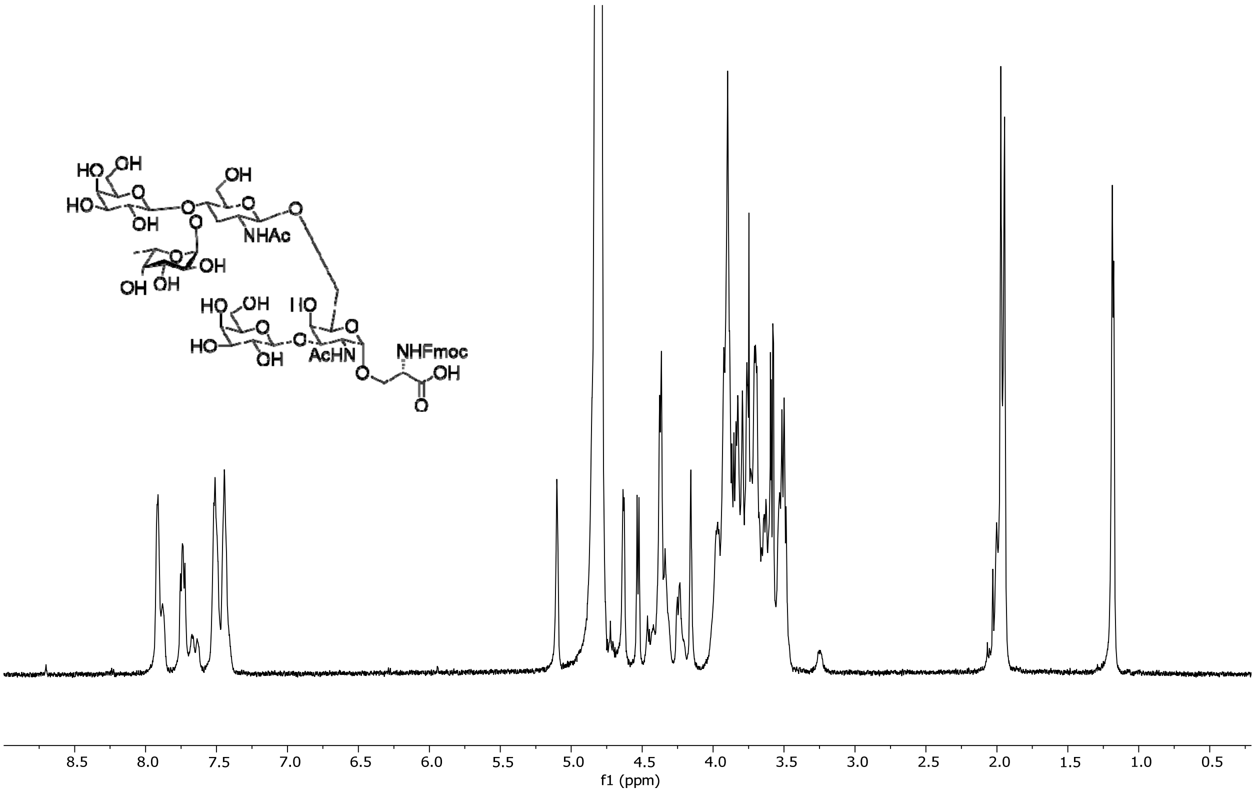

^{1}H NMR of 62

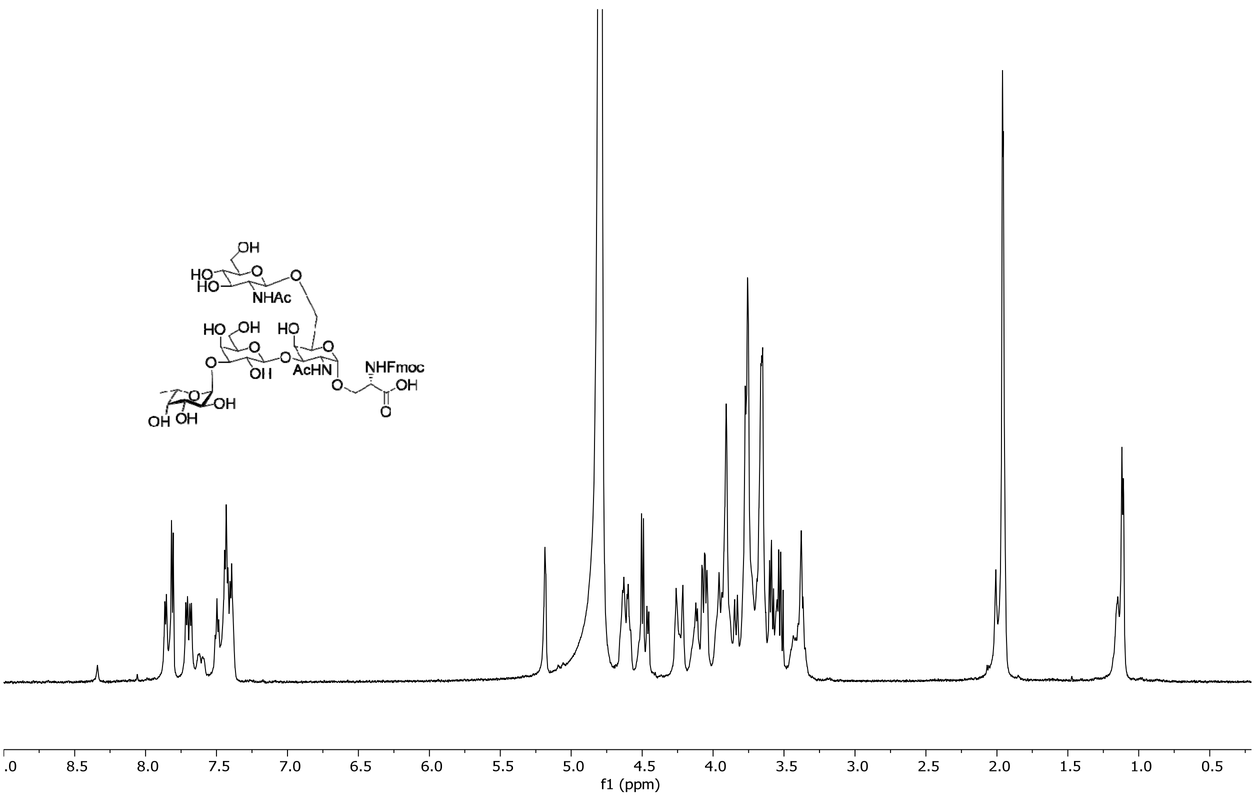

^{1}H NMR of 63

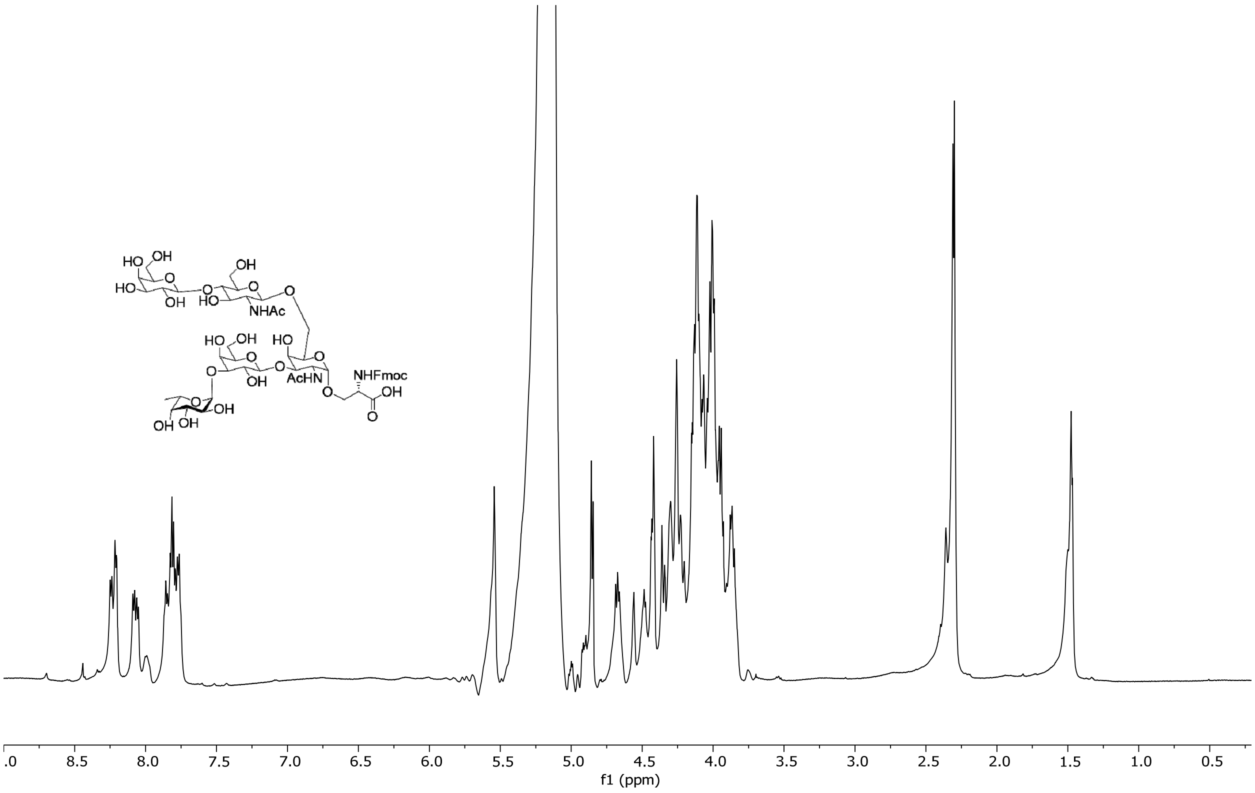

^{1}H NMR of 64

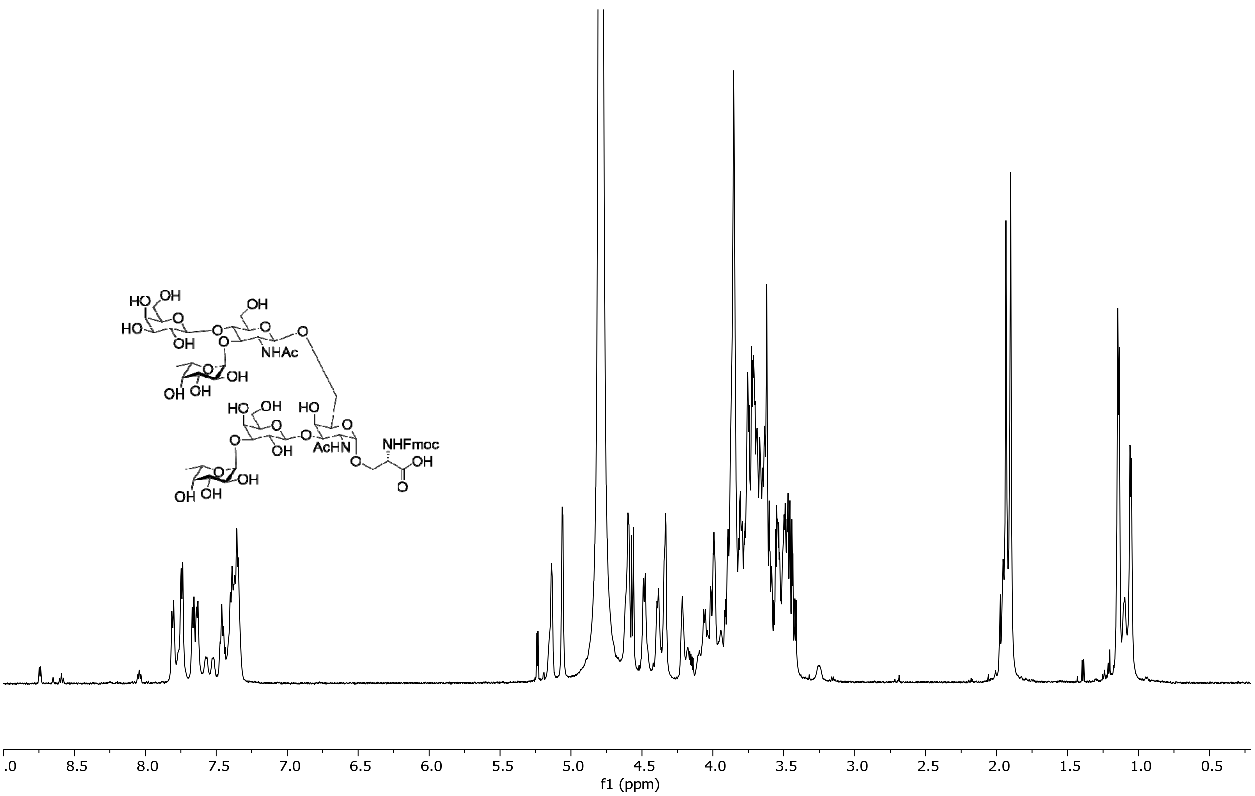

^{1}H NMR of 65

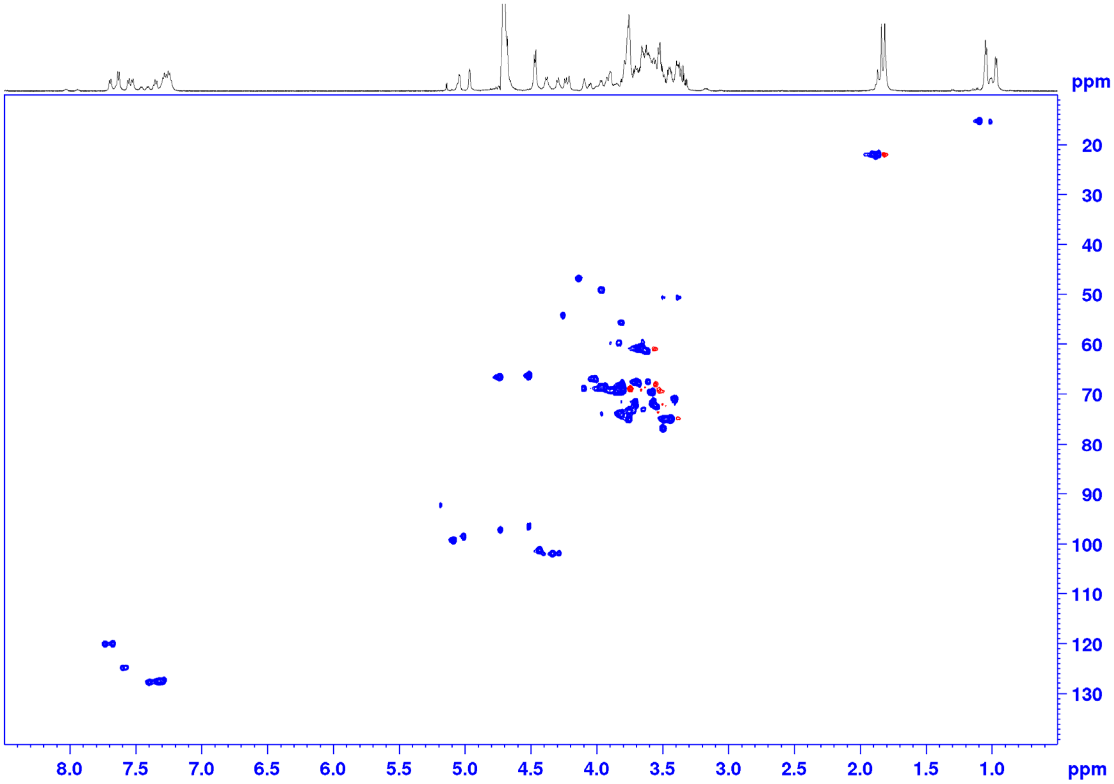

HSQC of 65

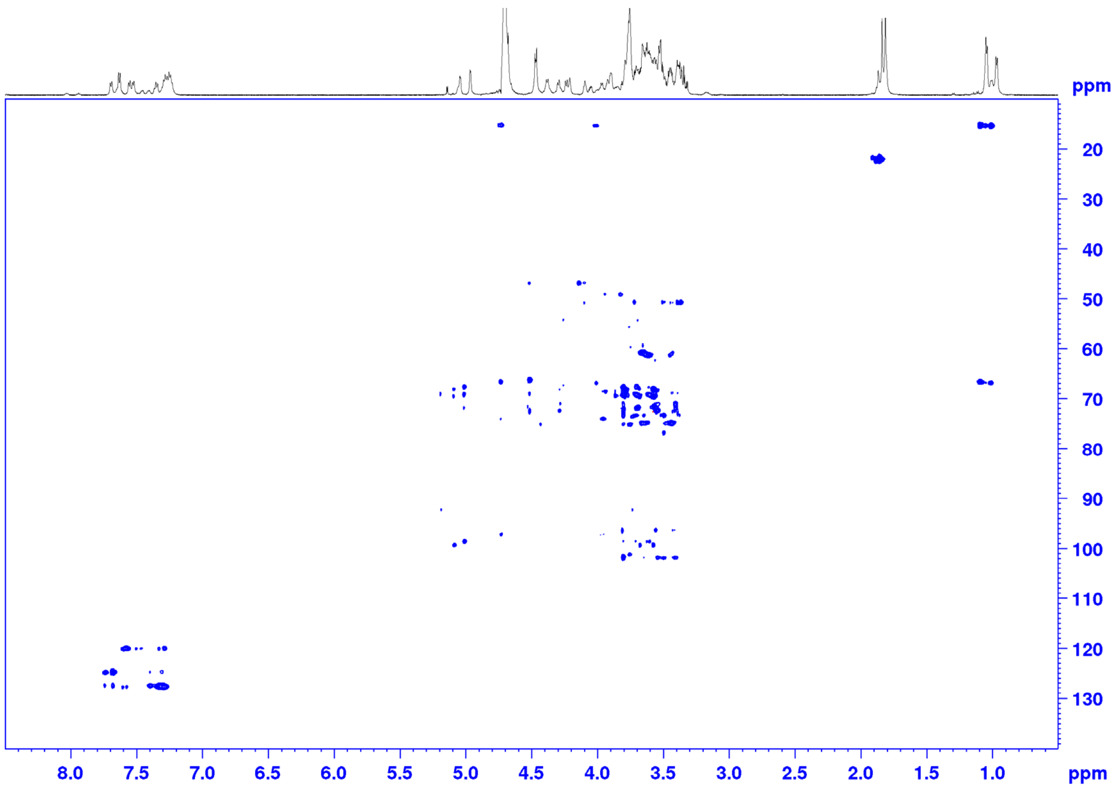

HSQC-TOCSY of 65

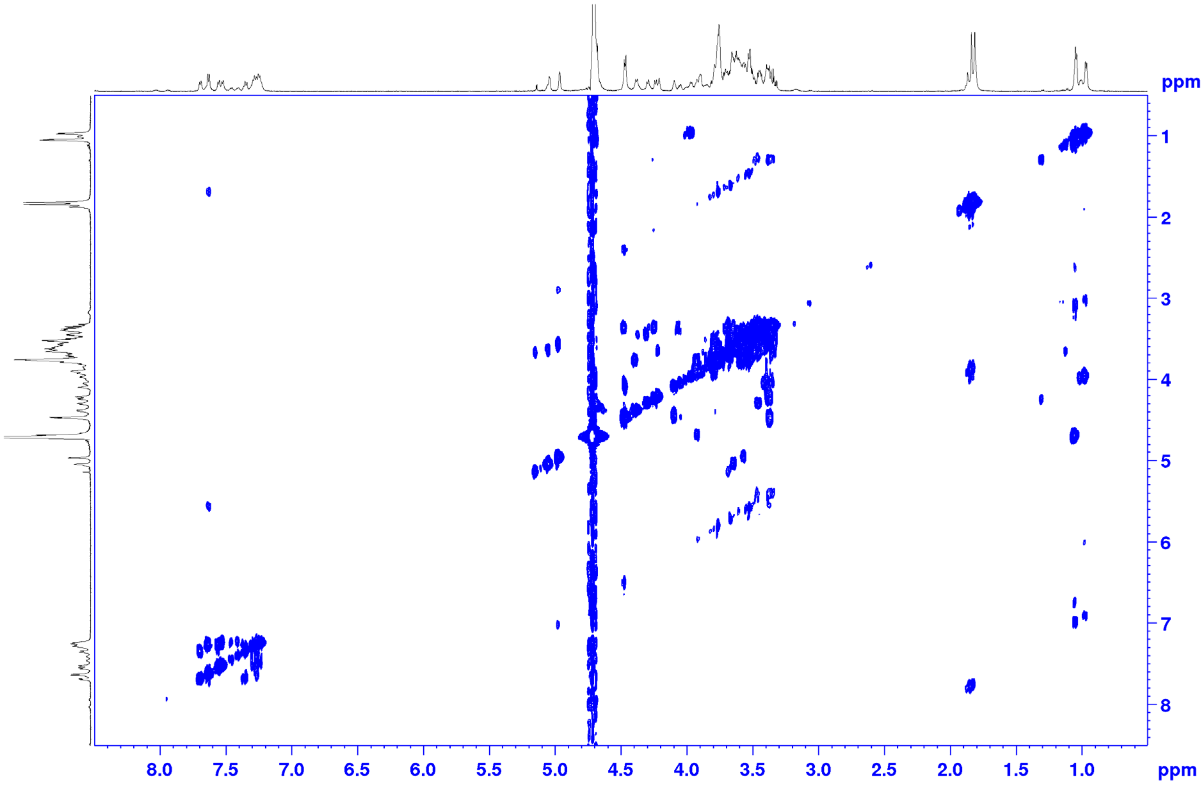

COSY of 65

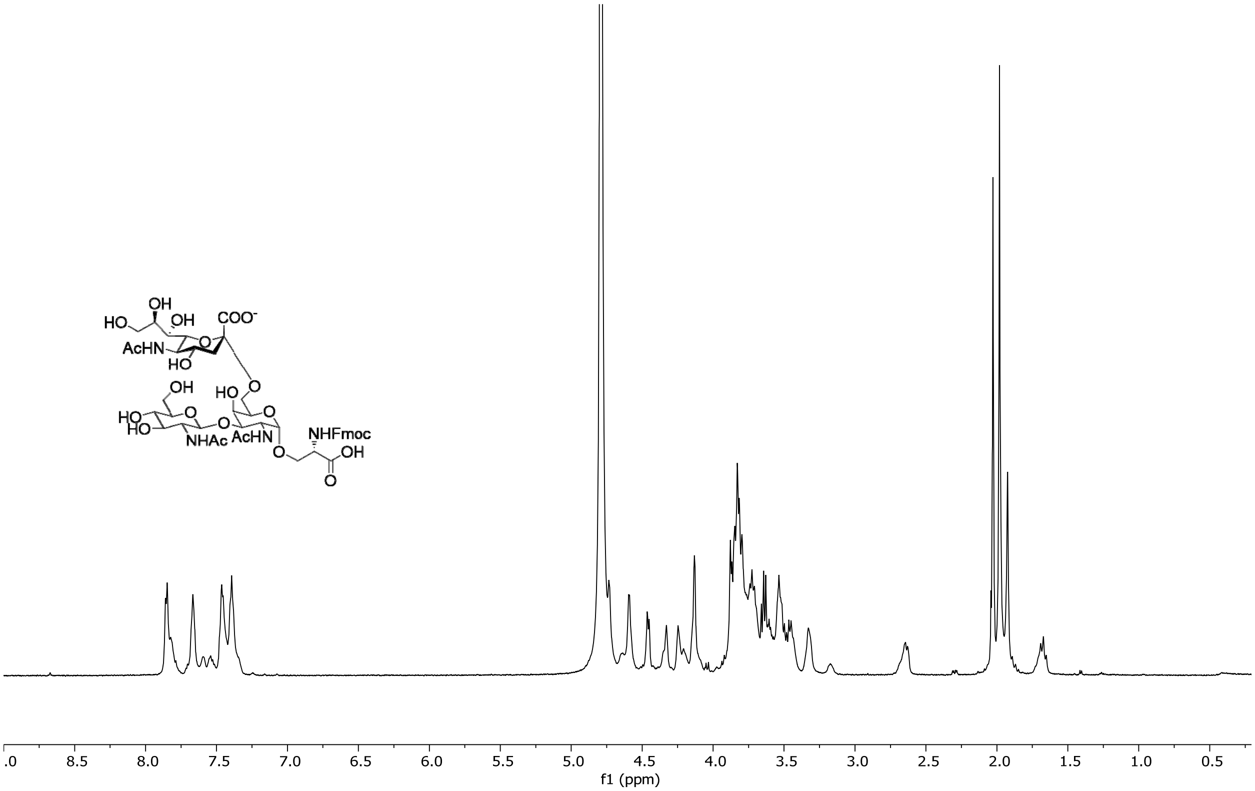

^{1}H NMR of 66

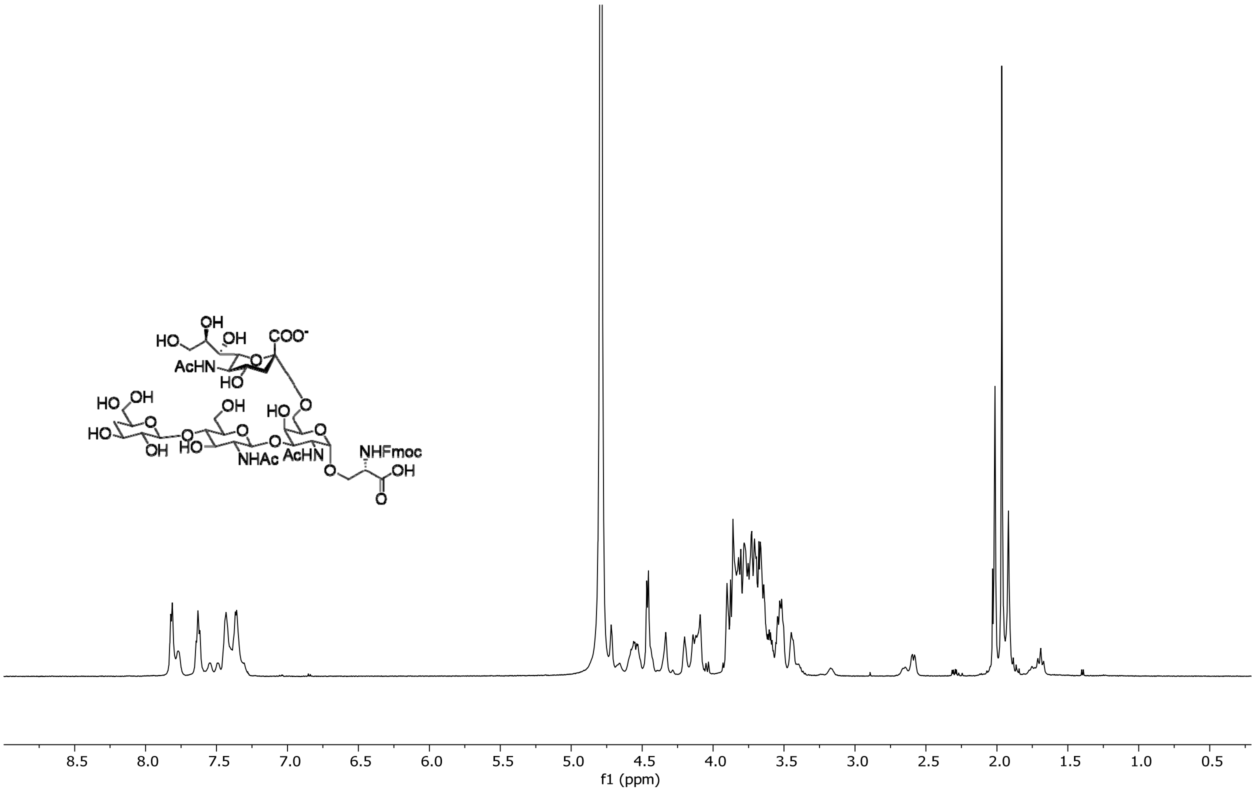

^{1}H NMR of 67

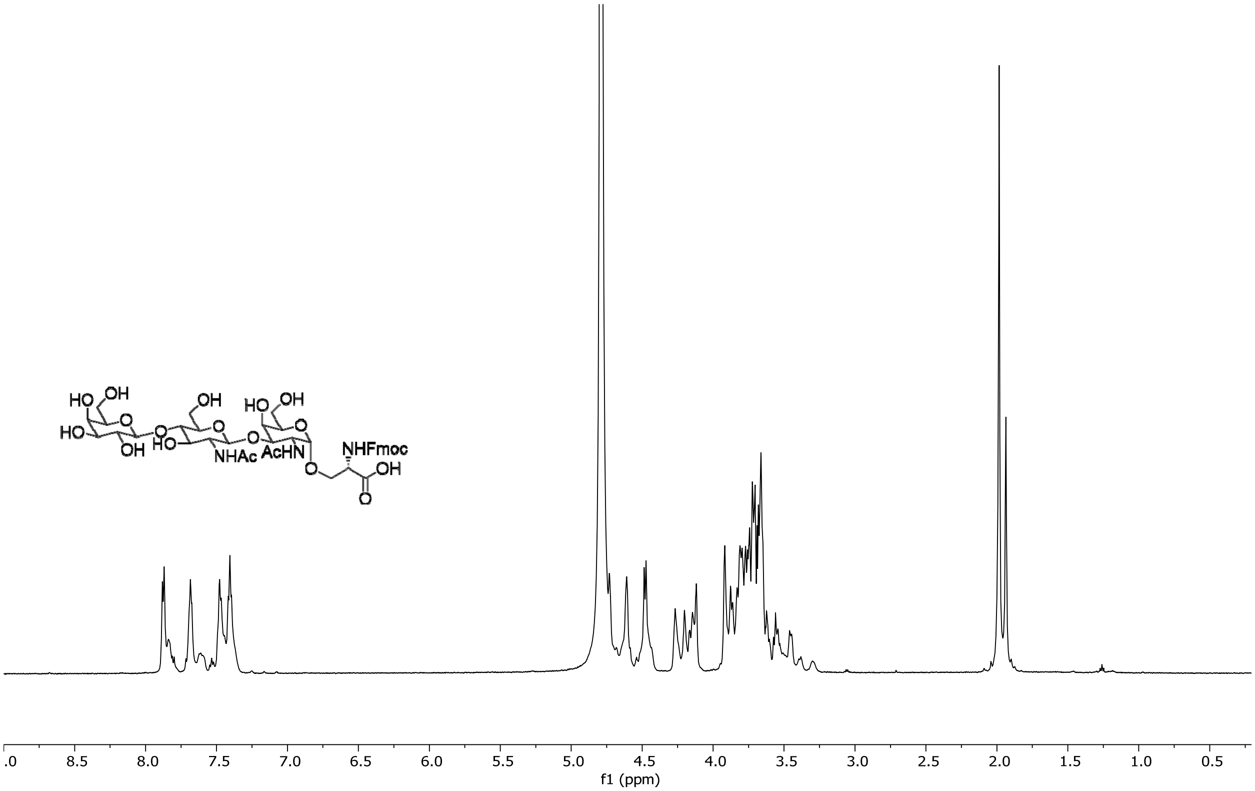

^{1}H NMR of 68

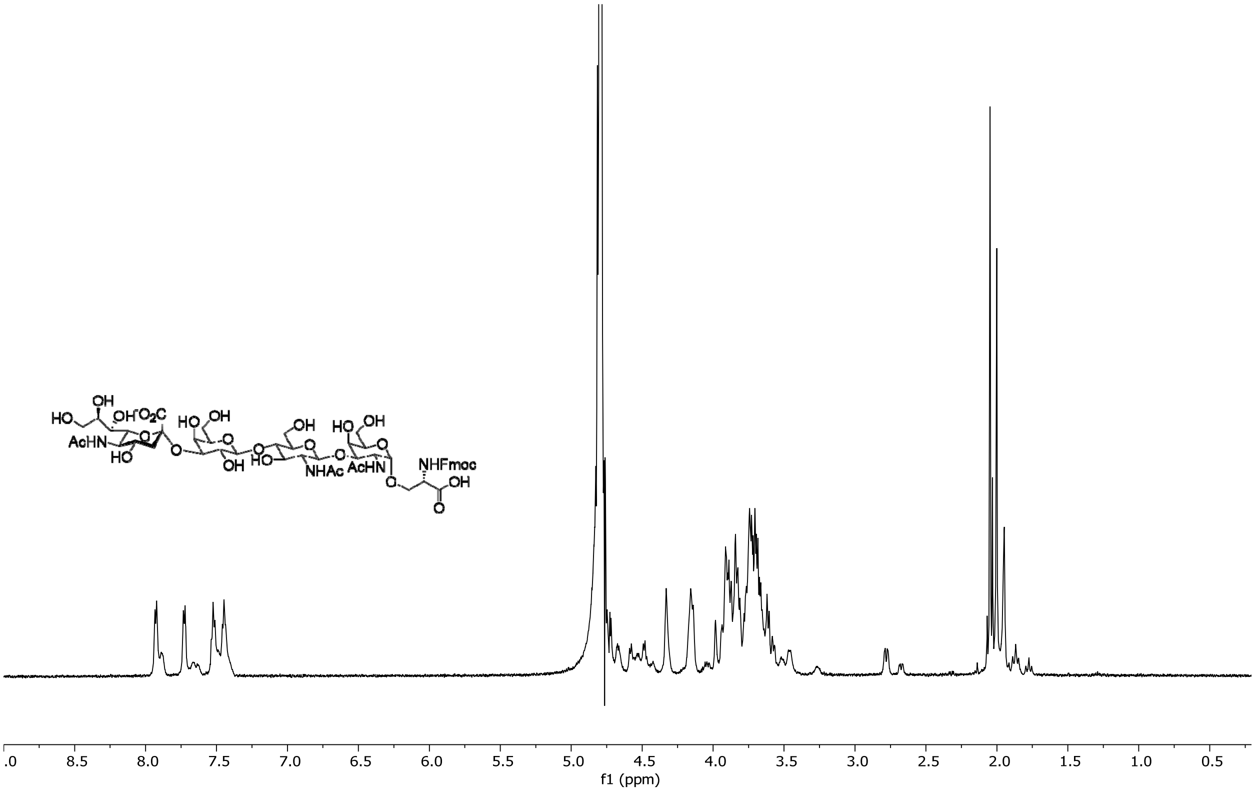

^{1}H NMR of 69

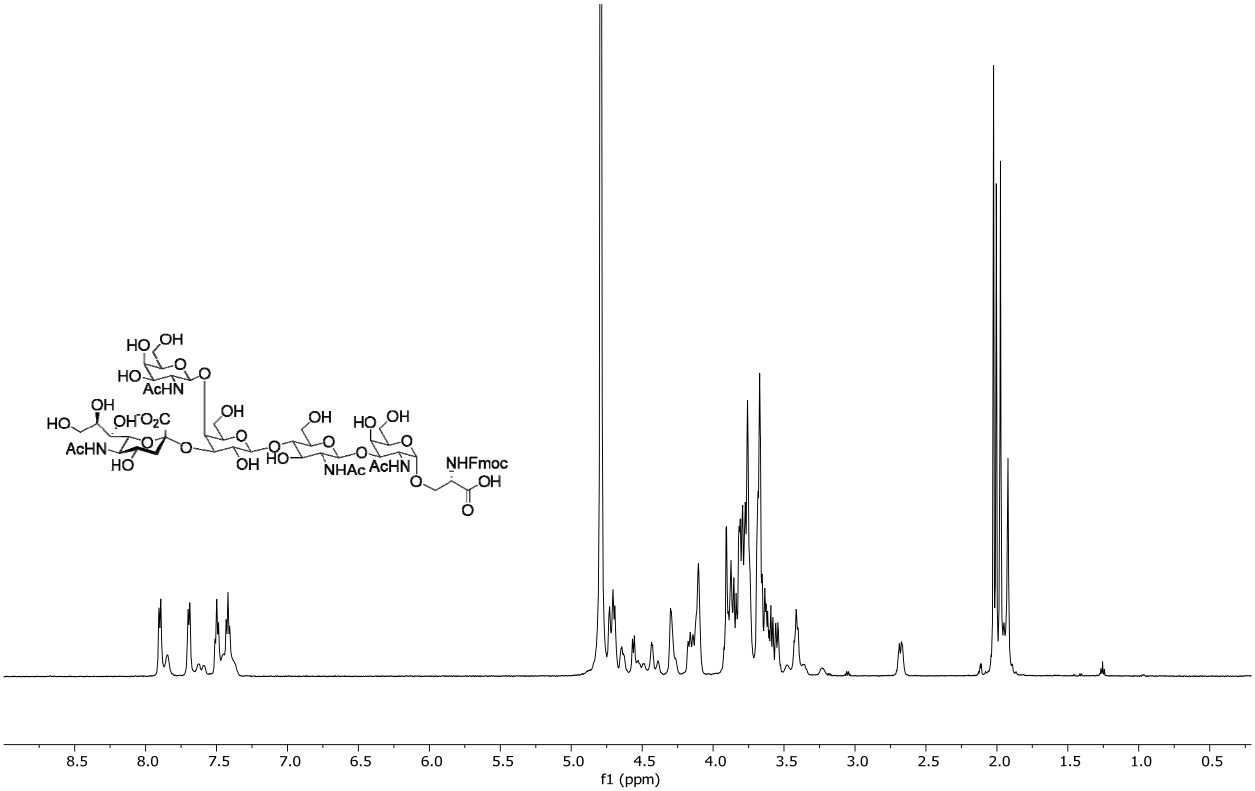

^{1}H NMR of 70

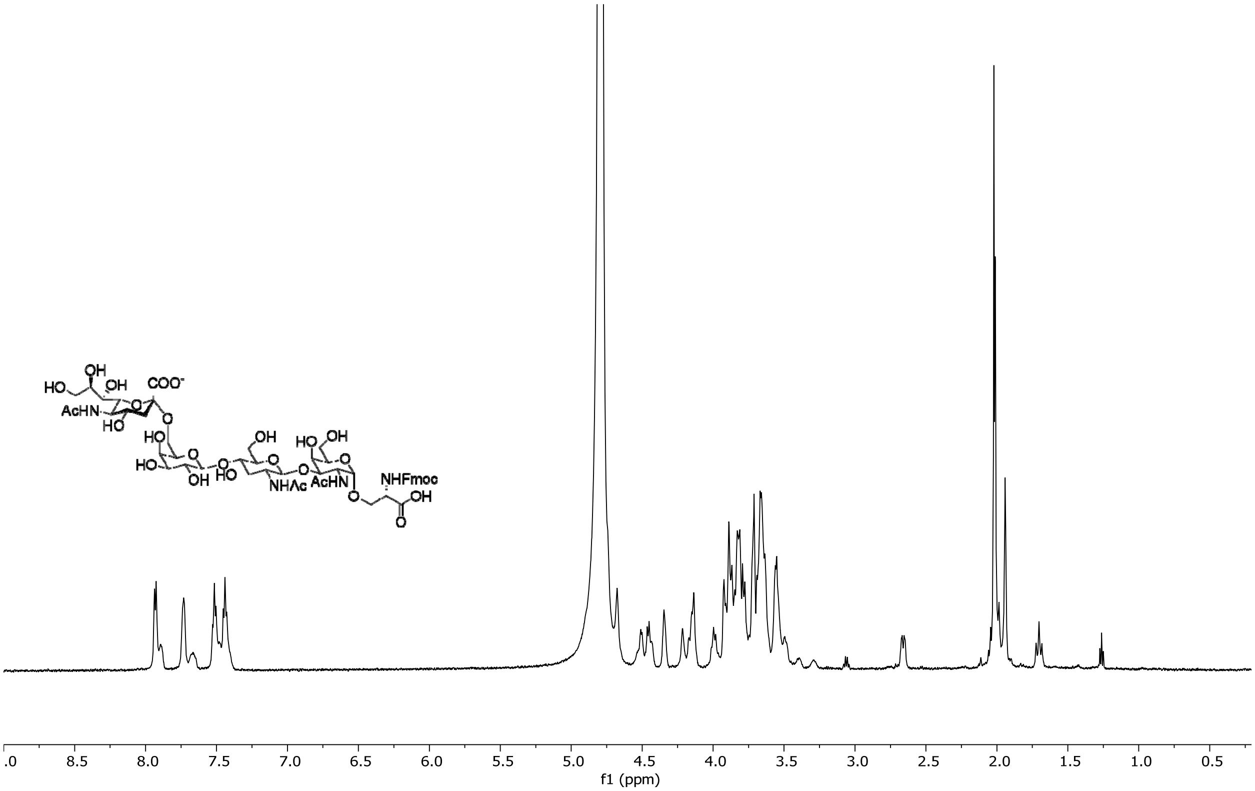

^{1}H NMR of 71

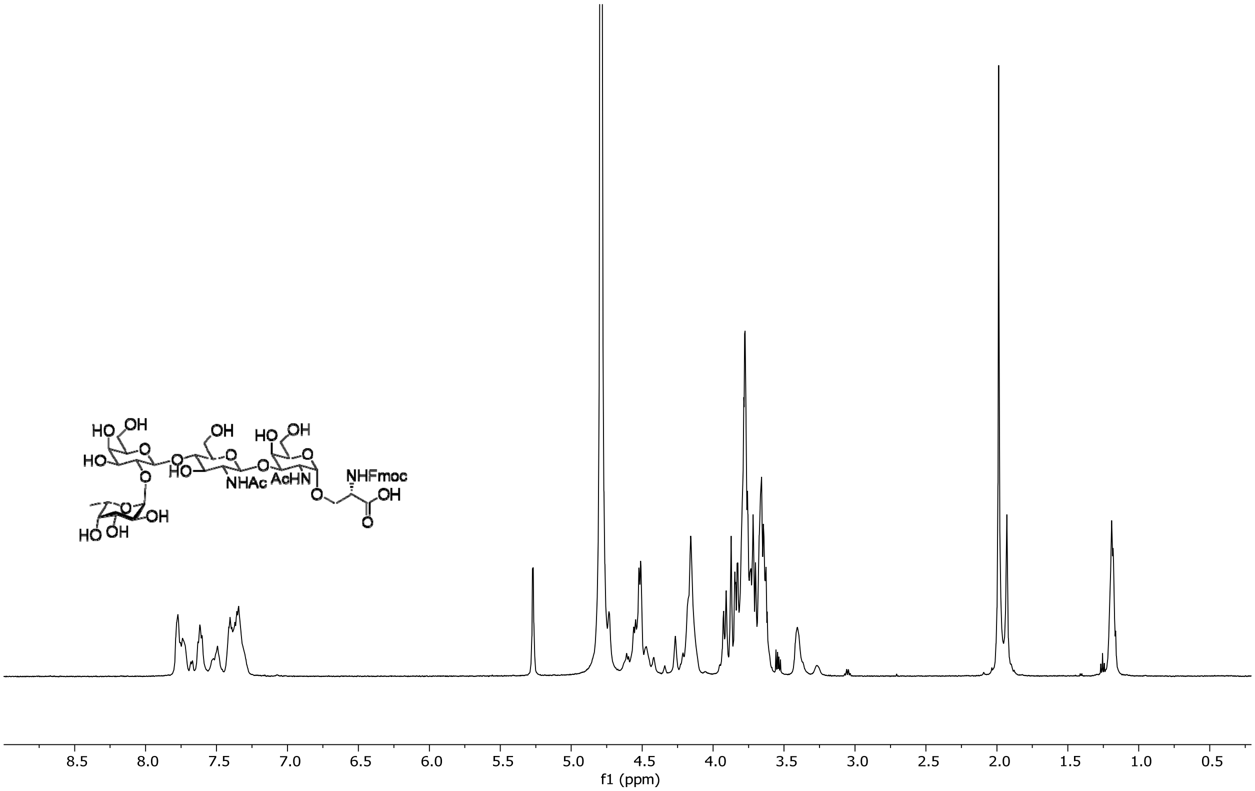

^{1}H NMR of 72

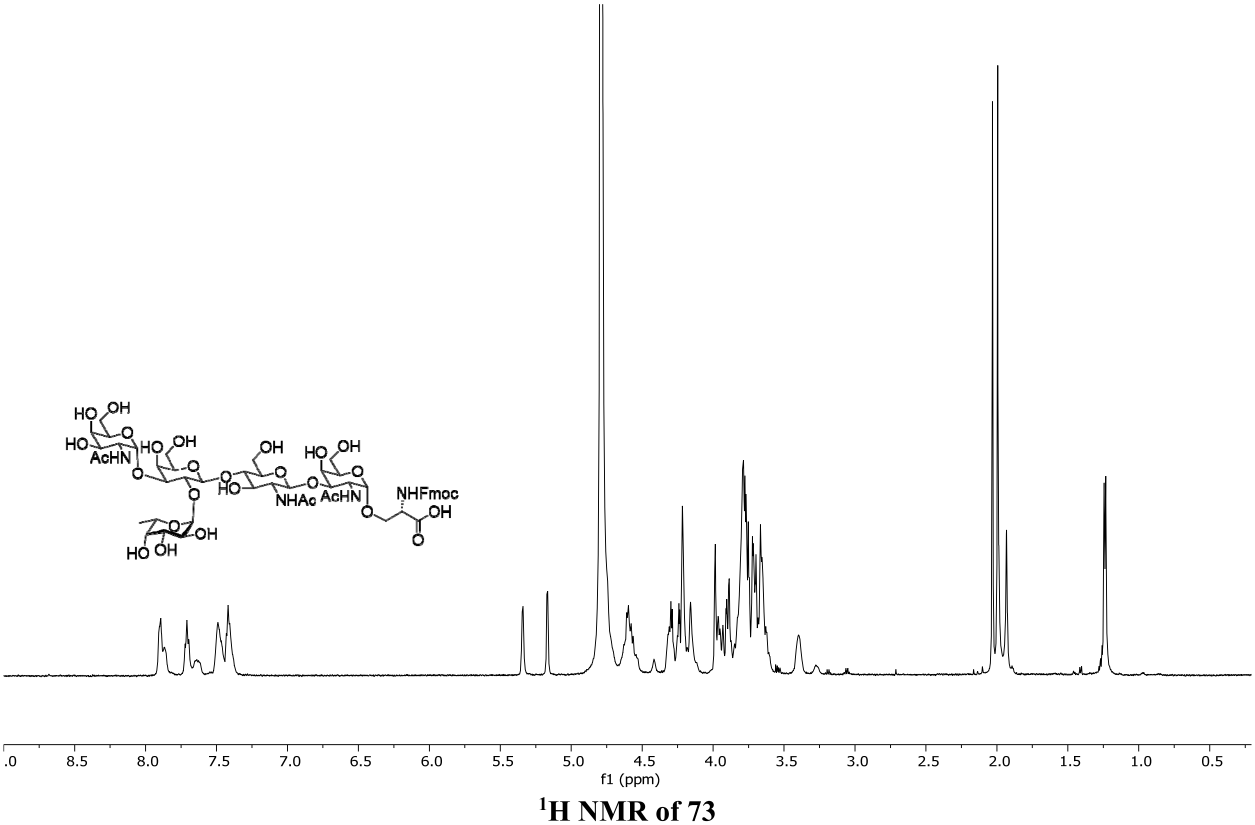

^{1}H NMR of 73

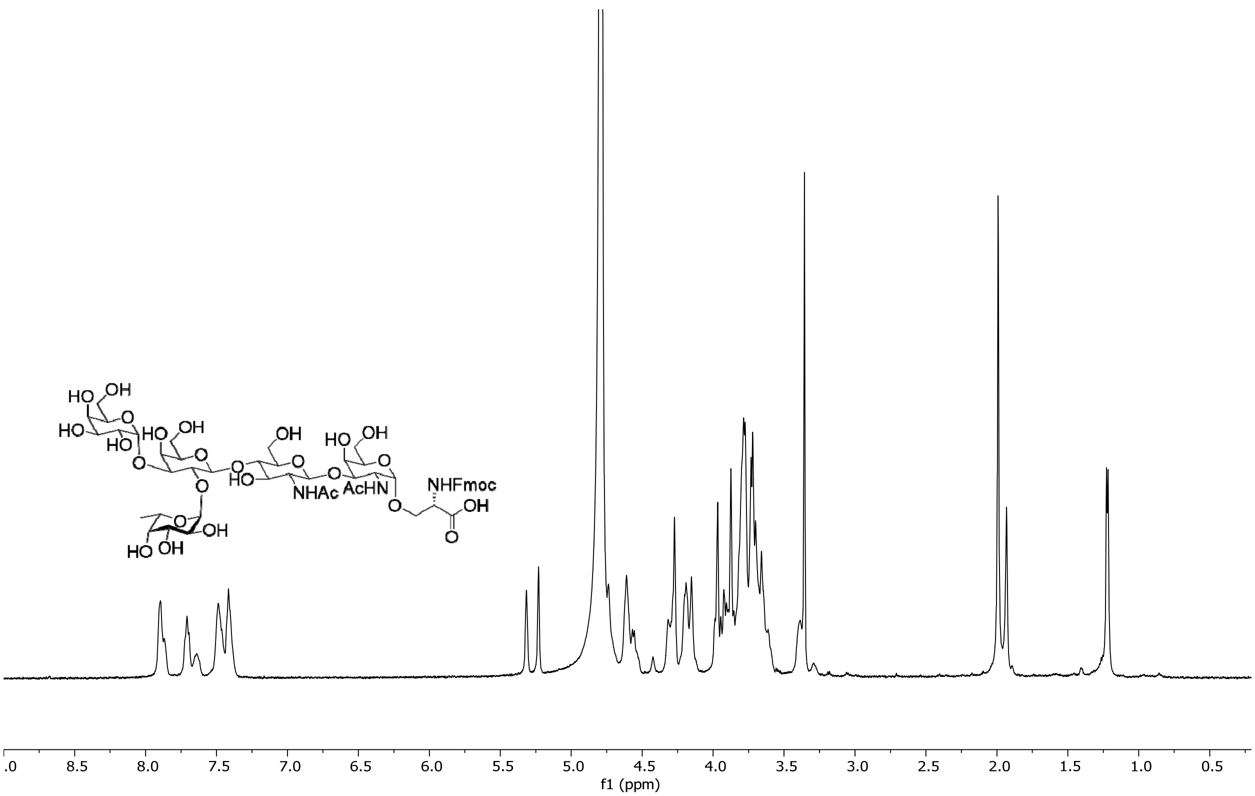

^{1}H NMR of 74

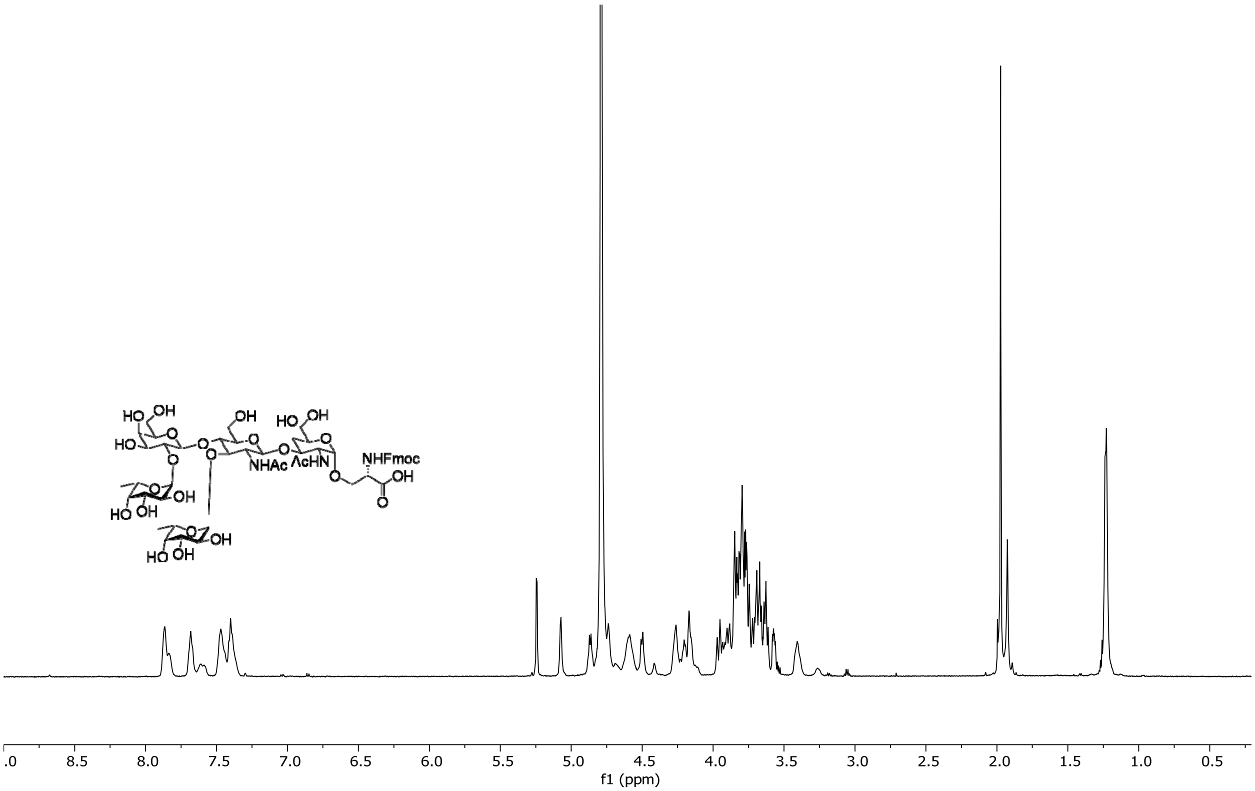

^{1}H NMR of 75

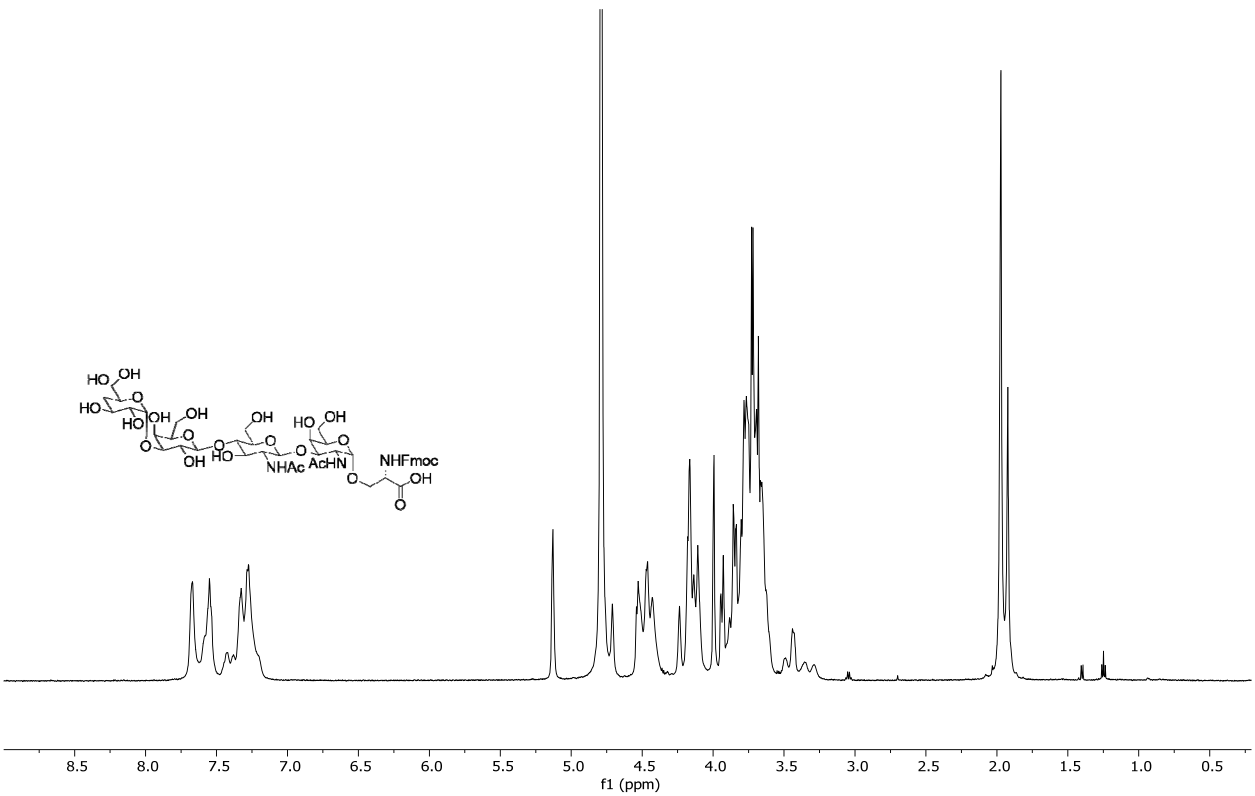

^{1}H NMR of 76

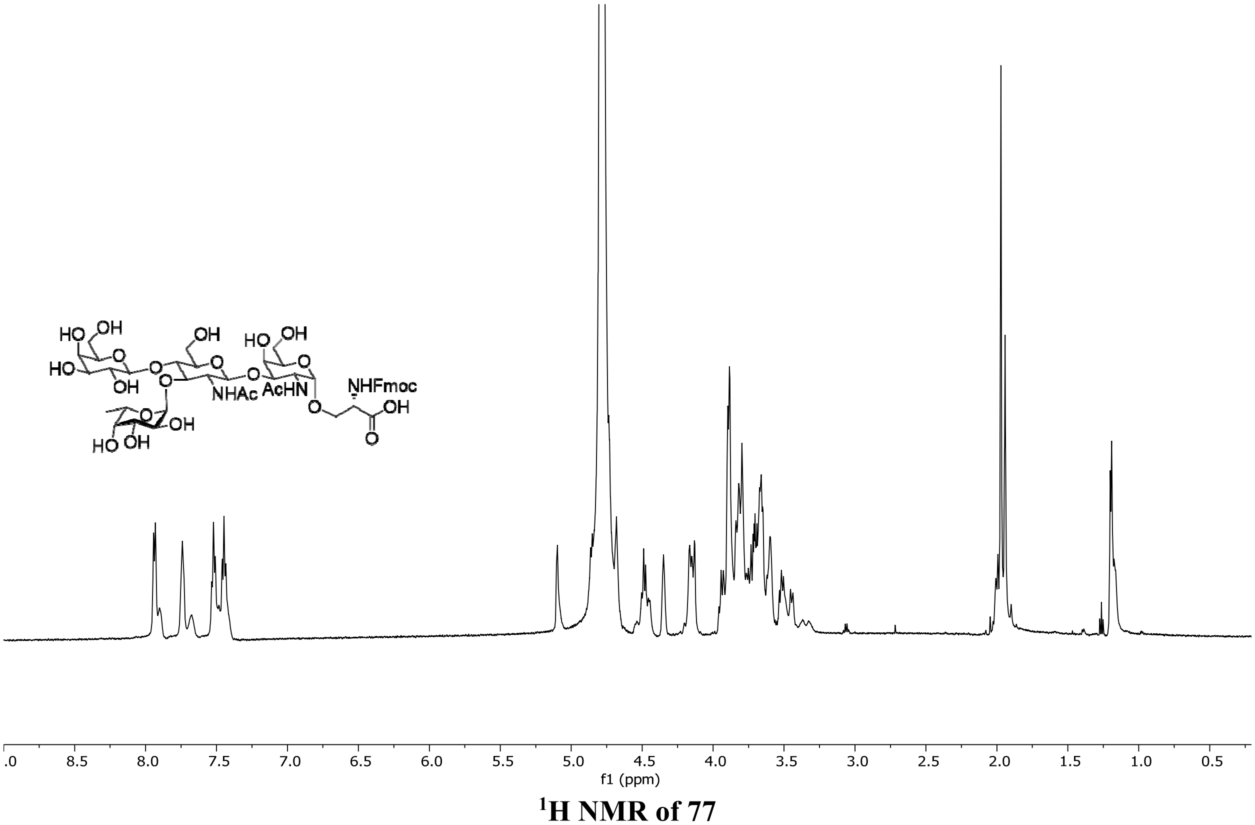

^{1}H NMR of 77

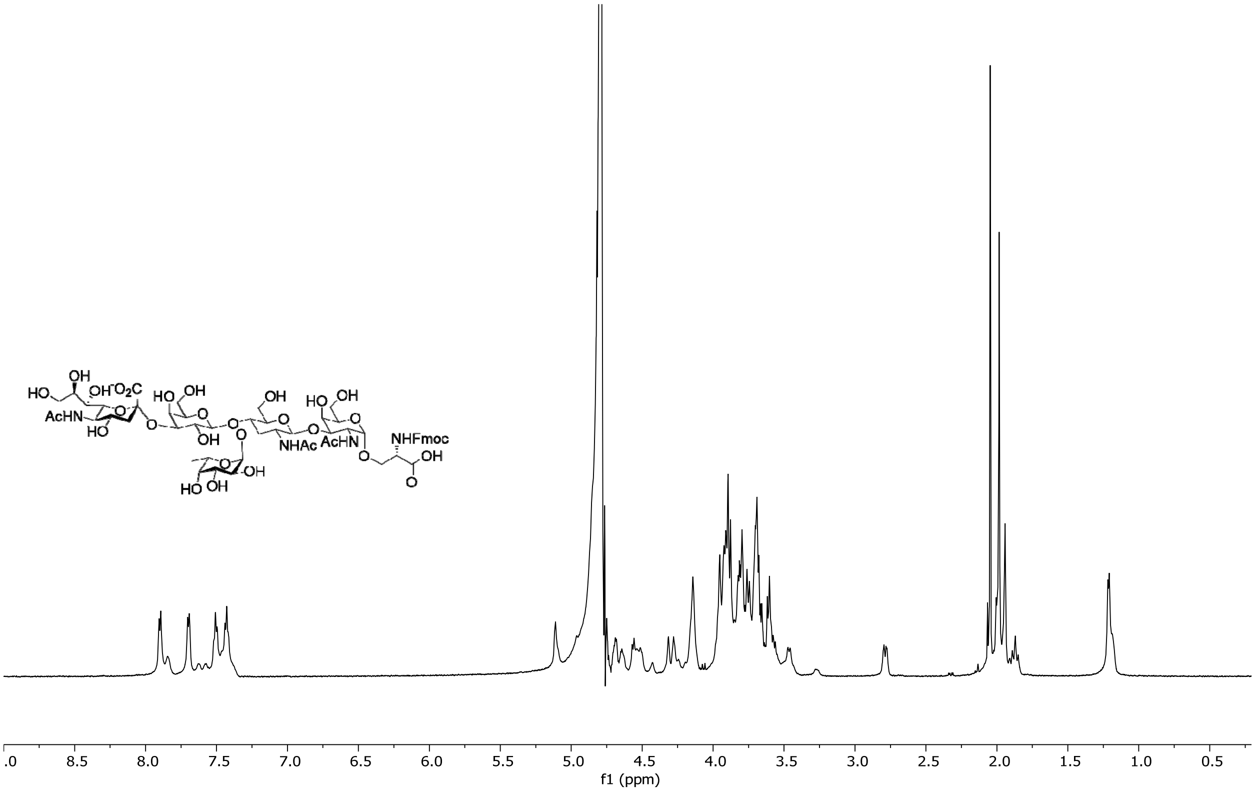

^{1}H NMR of 78

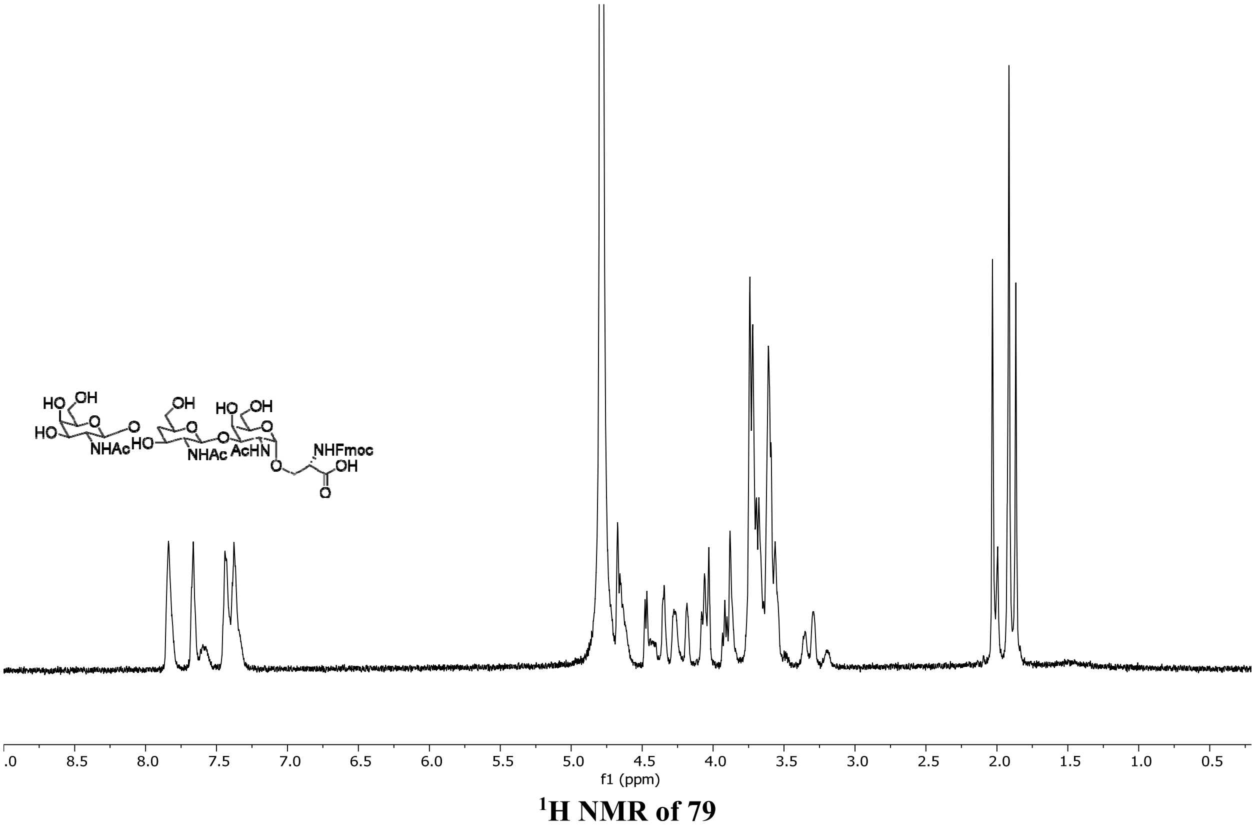

^{1}H NMR of 79

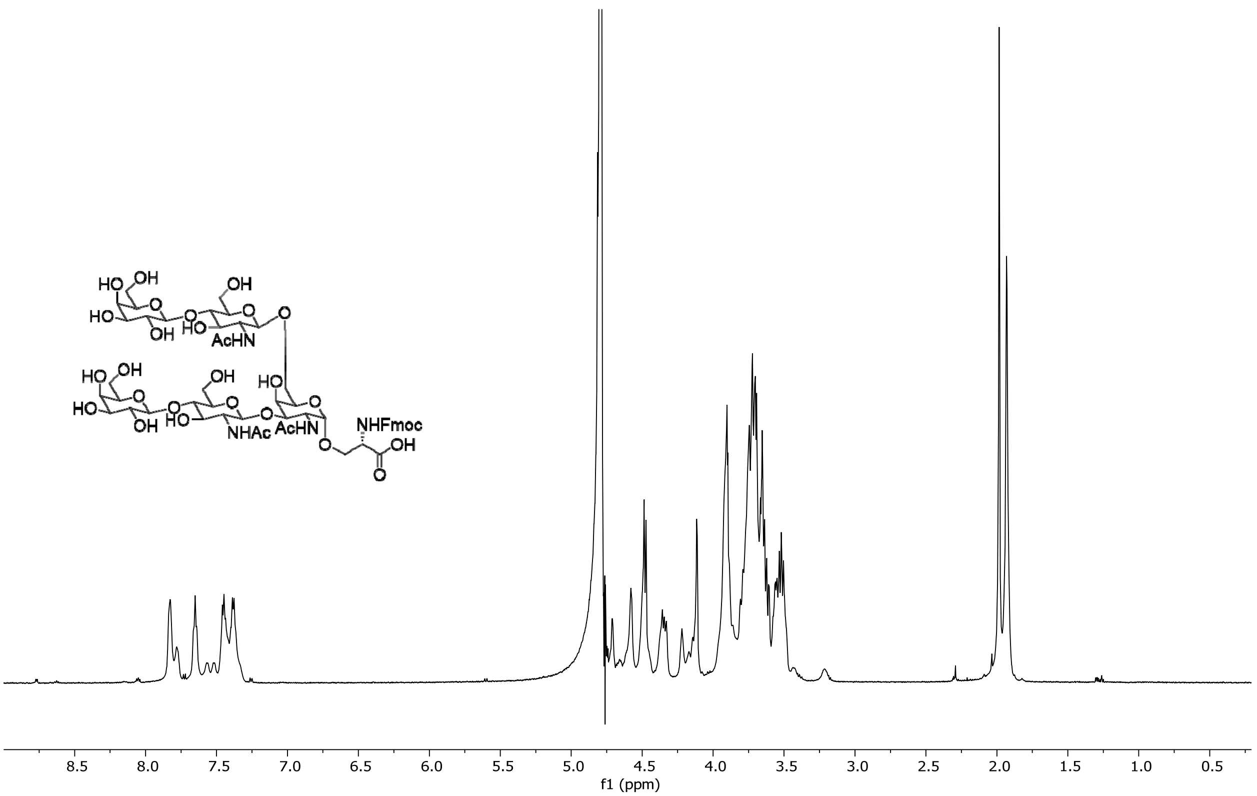

^{1}H NMR of 80

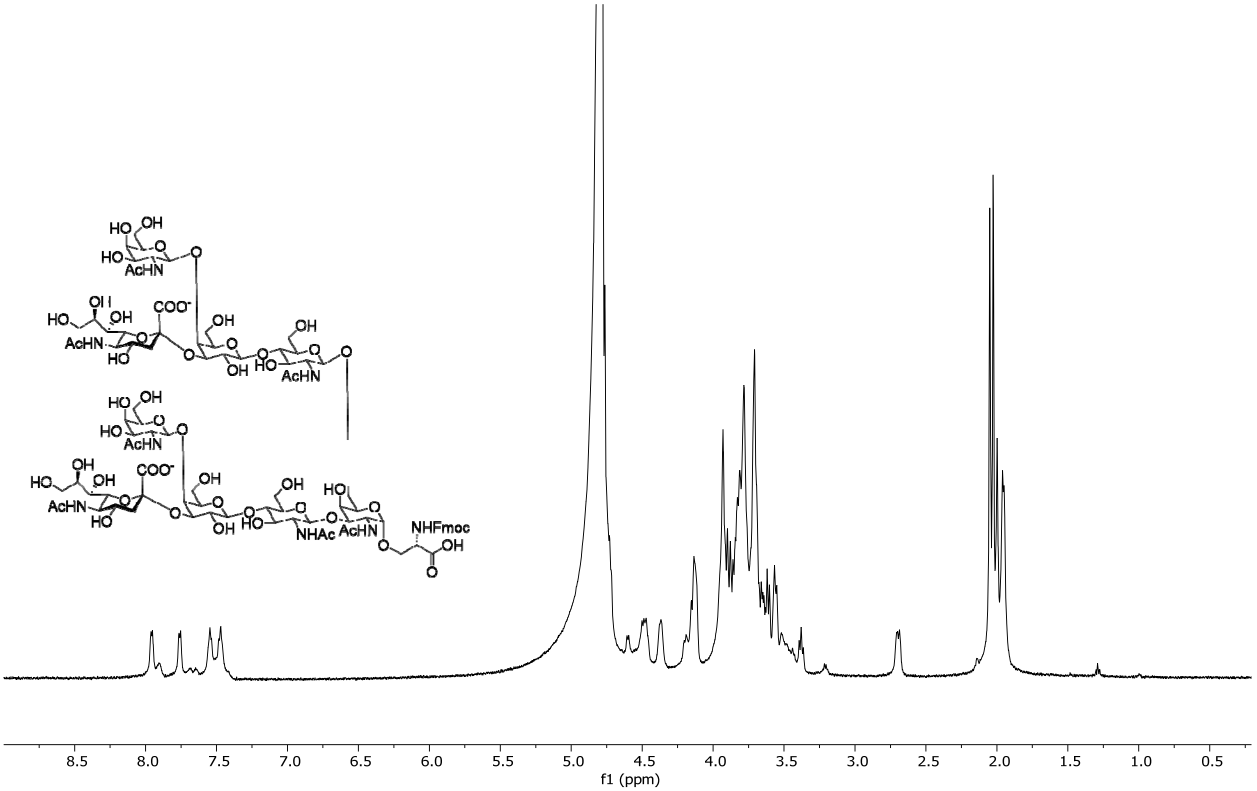

^{1}H NMR of 81

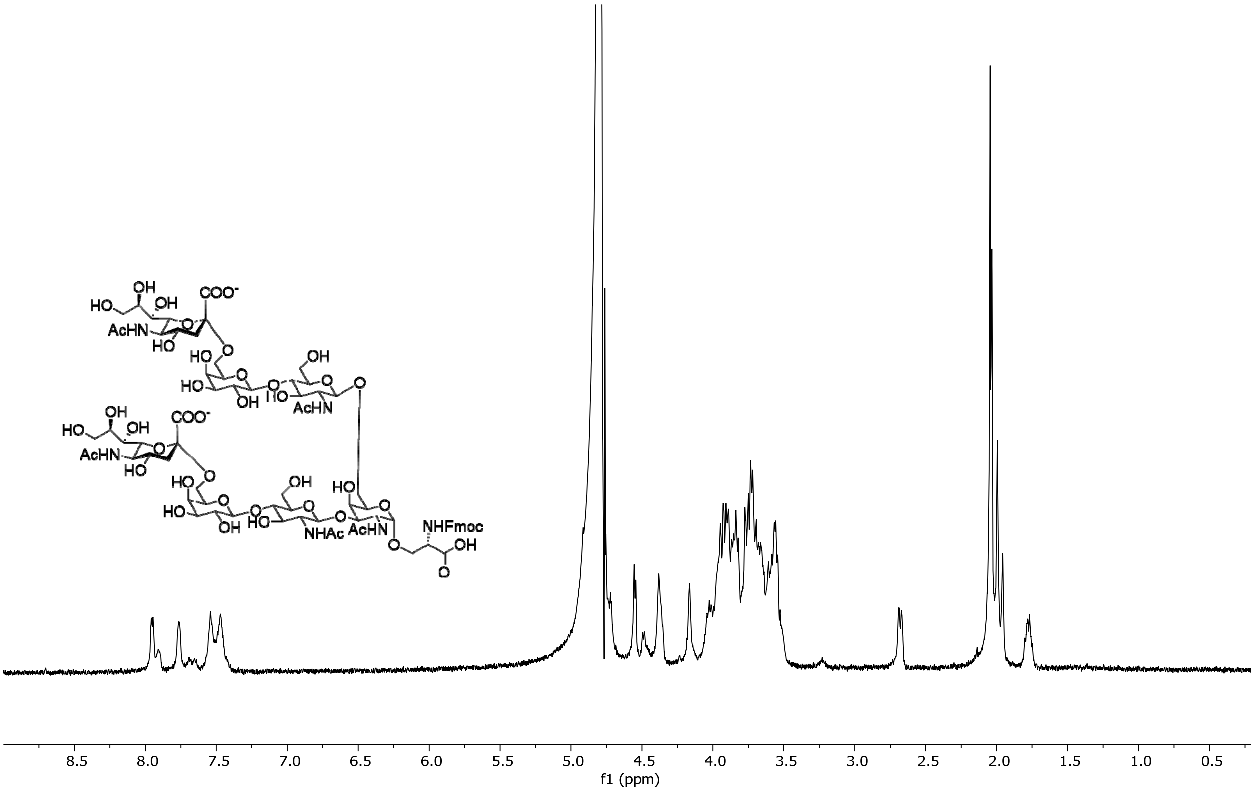

^{1}H NMR of 82

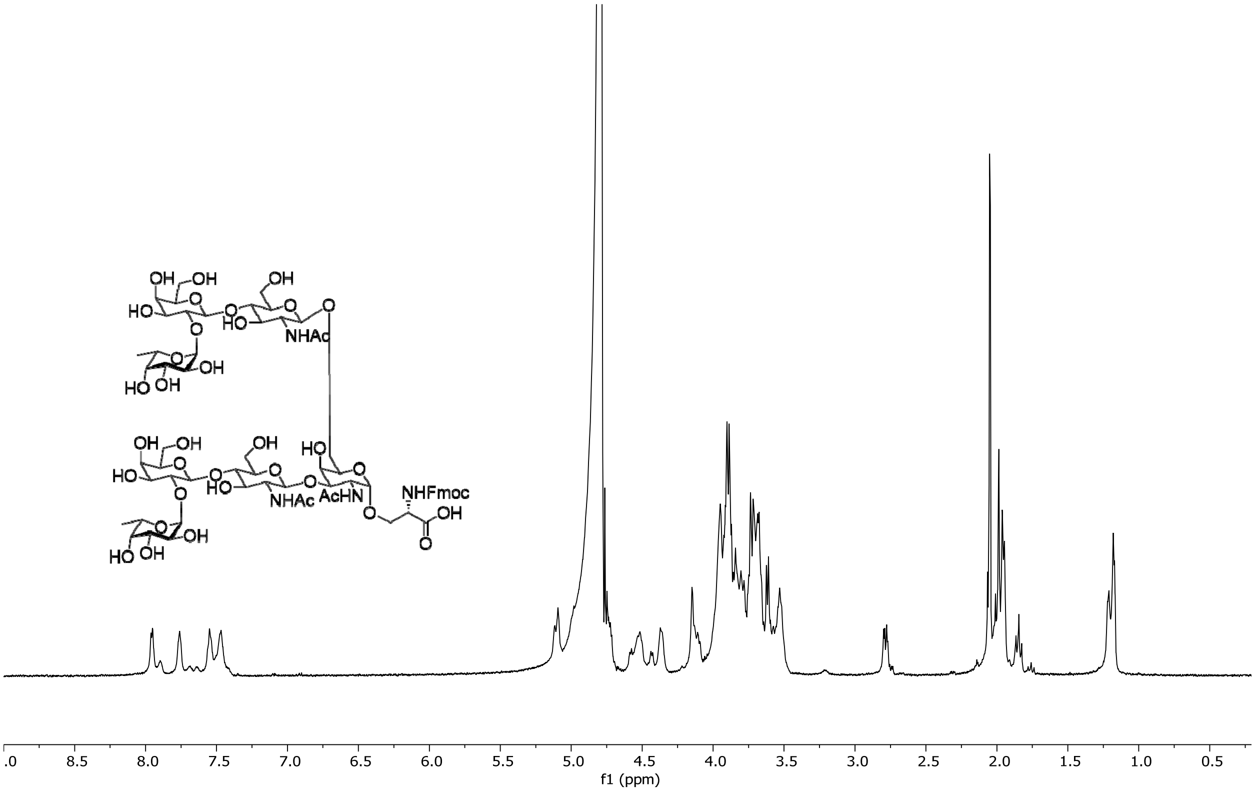

^{1}H NMR of 83

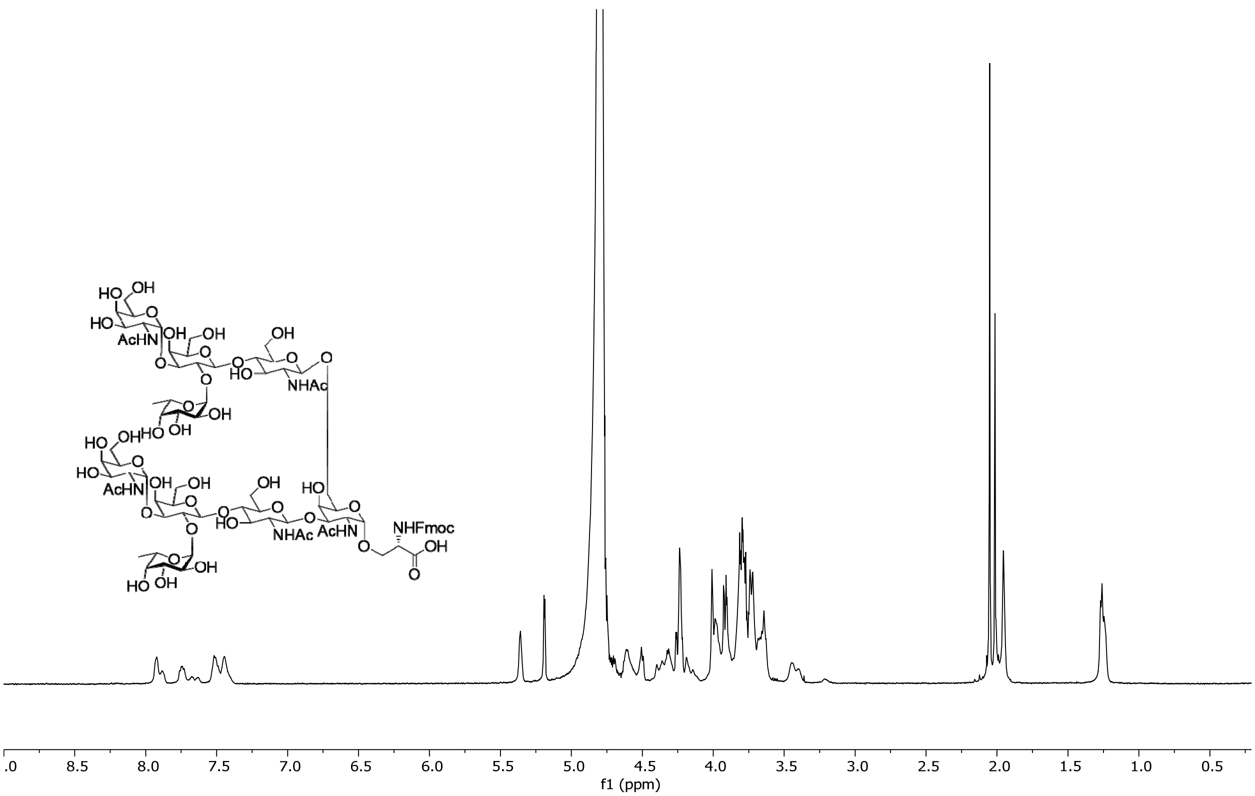

^{1}H NMR of 84

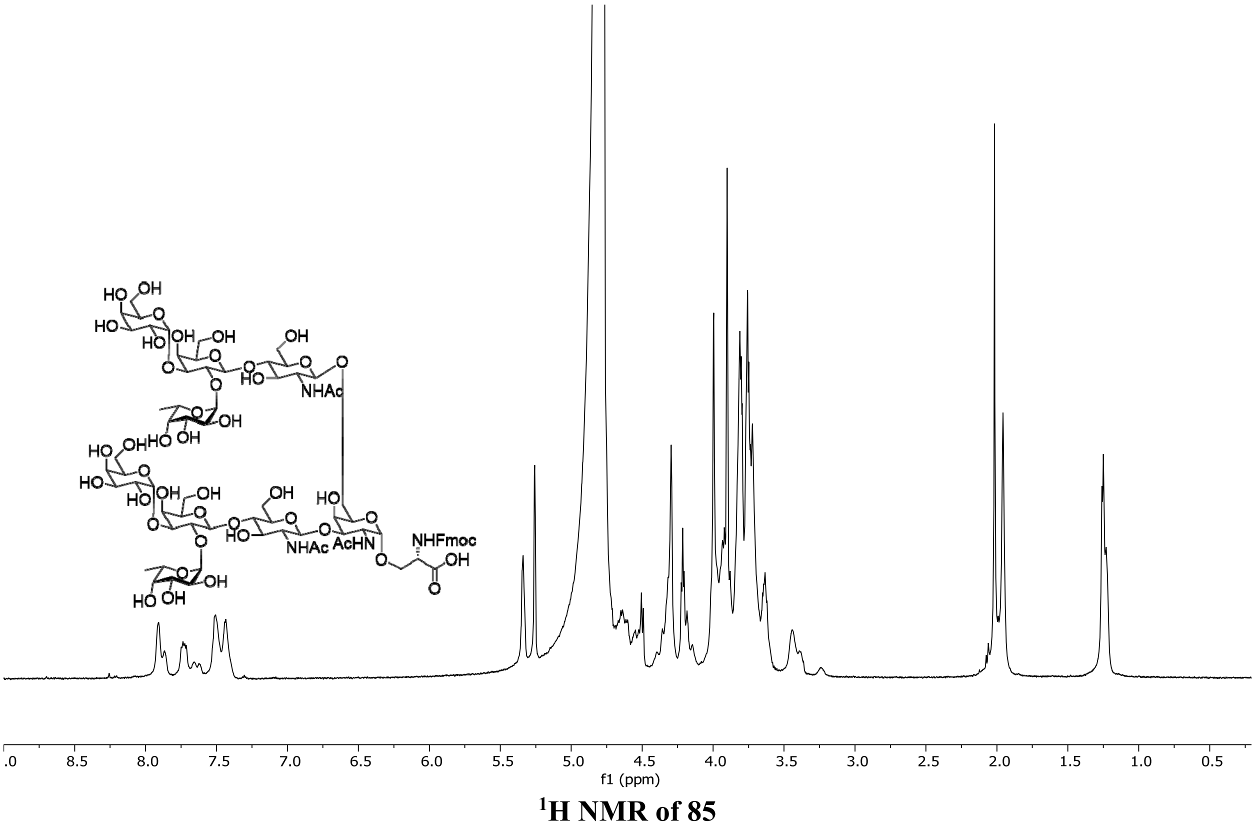

^{1}H NMR of 85

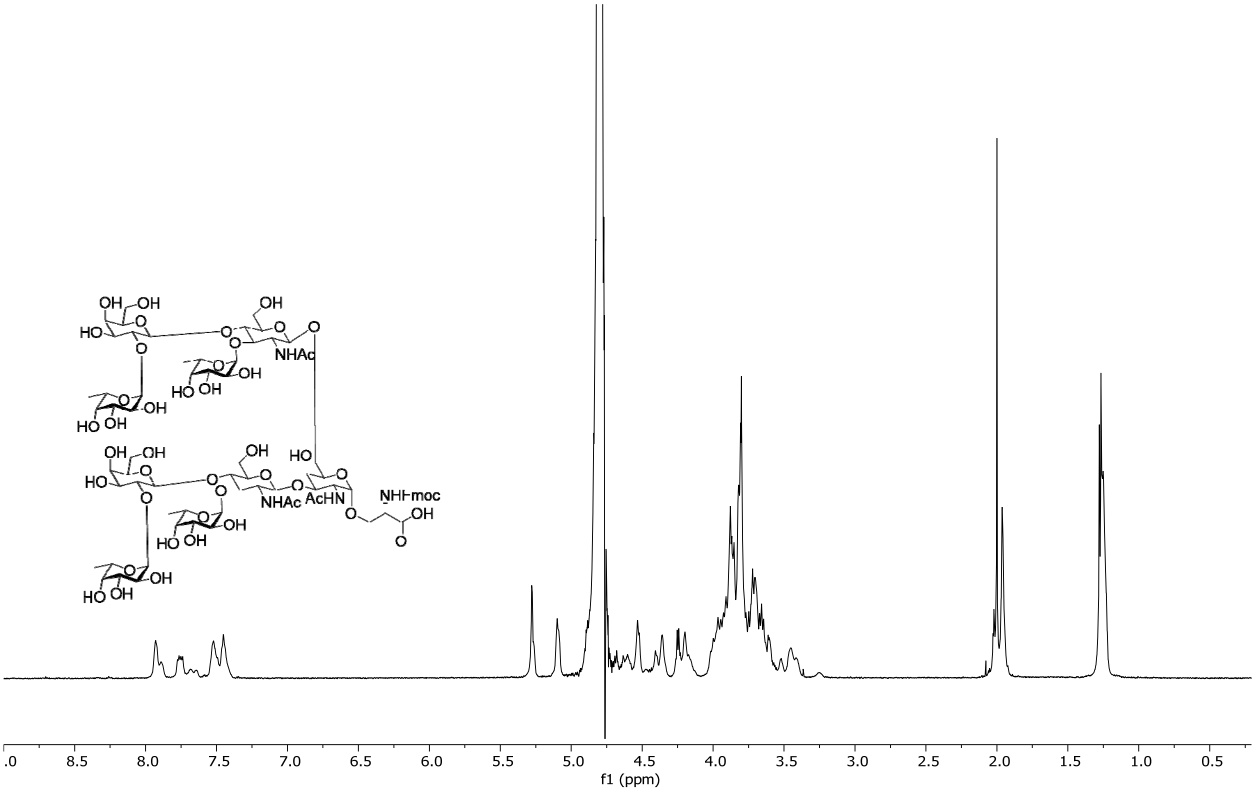

^{1}H NMR of 86

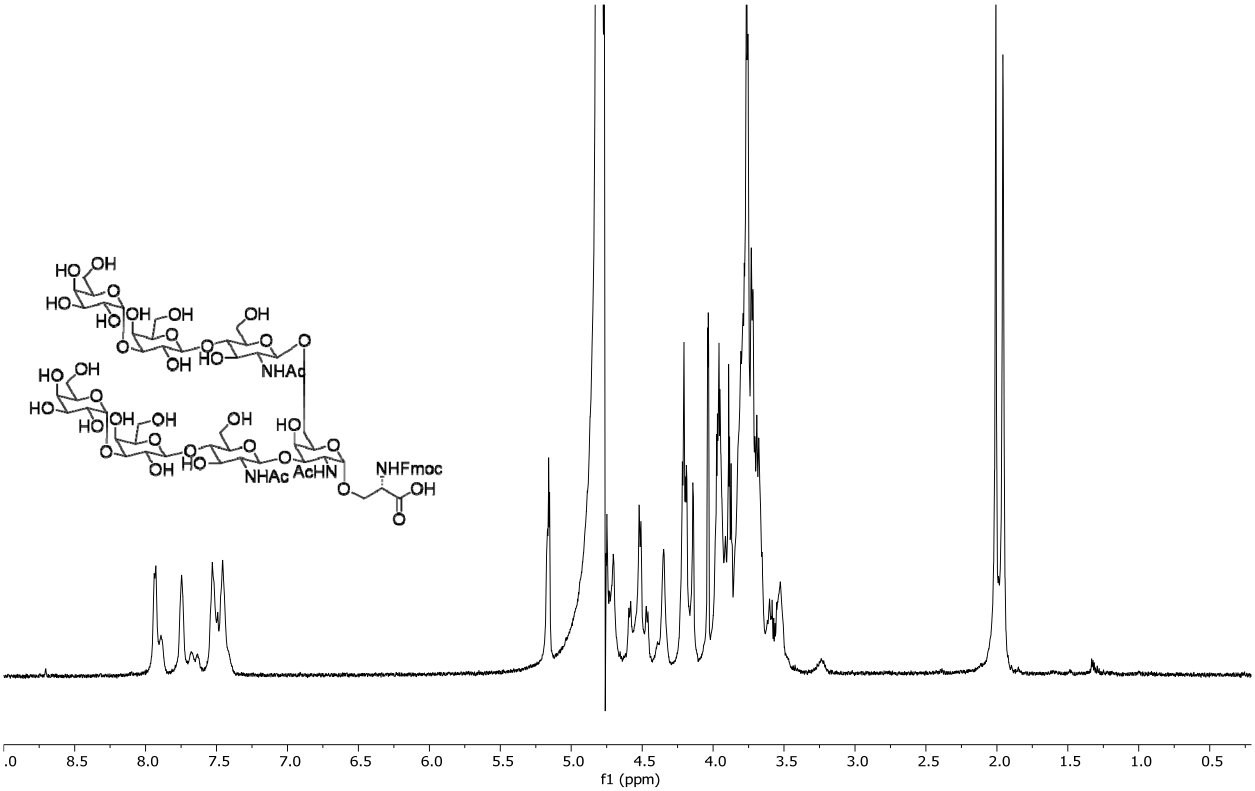

^{1}H NMR of 87

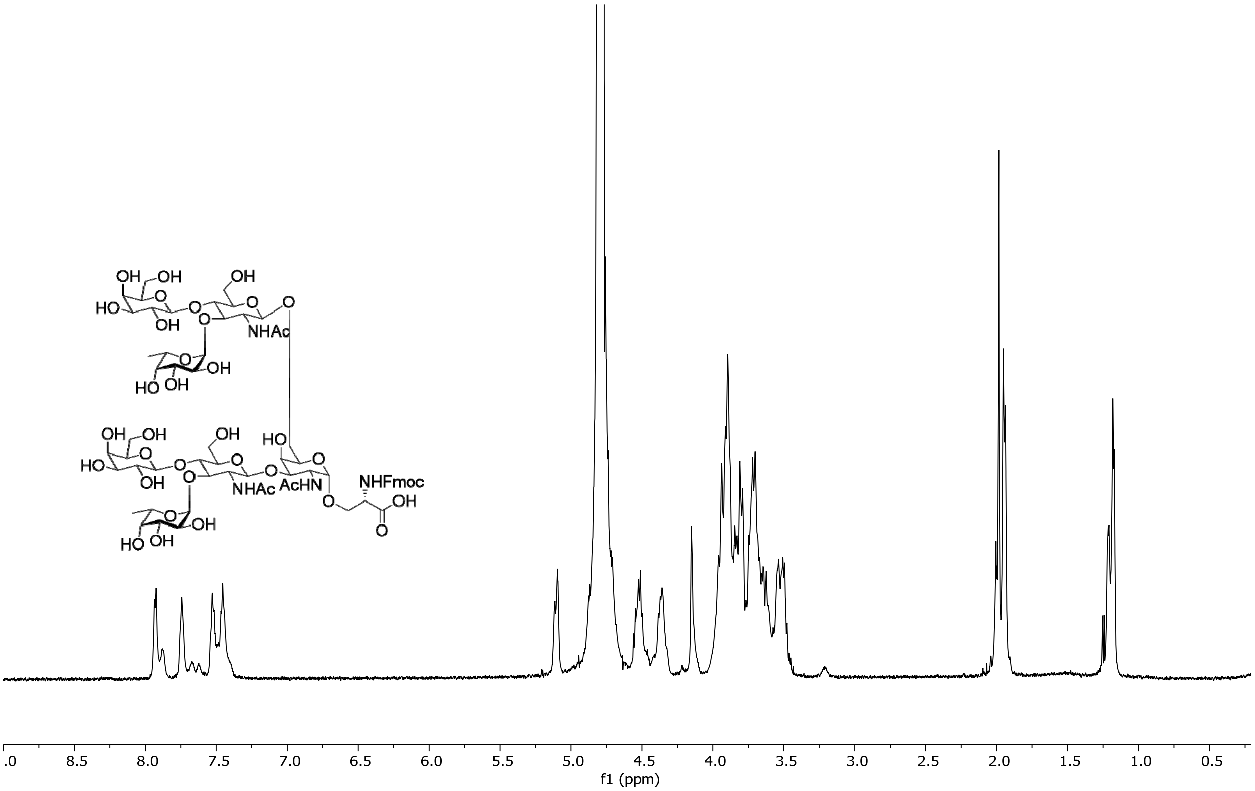

^{1}H NMR of 88

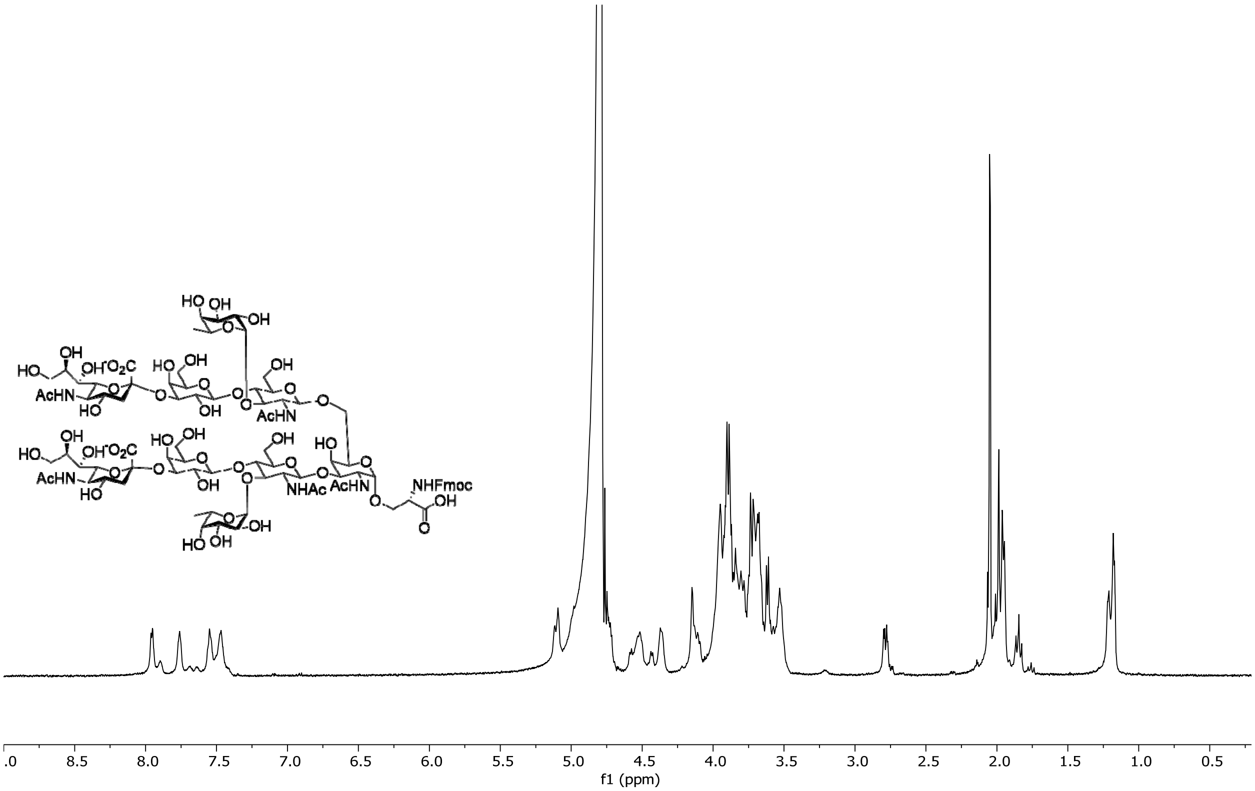

^{1}H NMR of 89

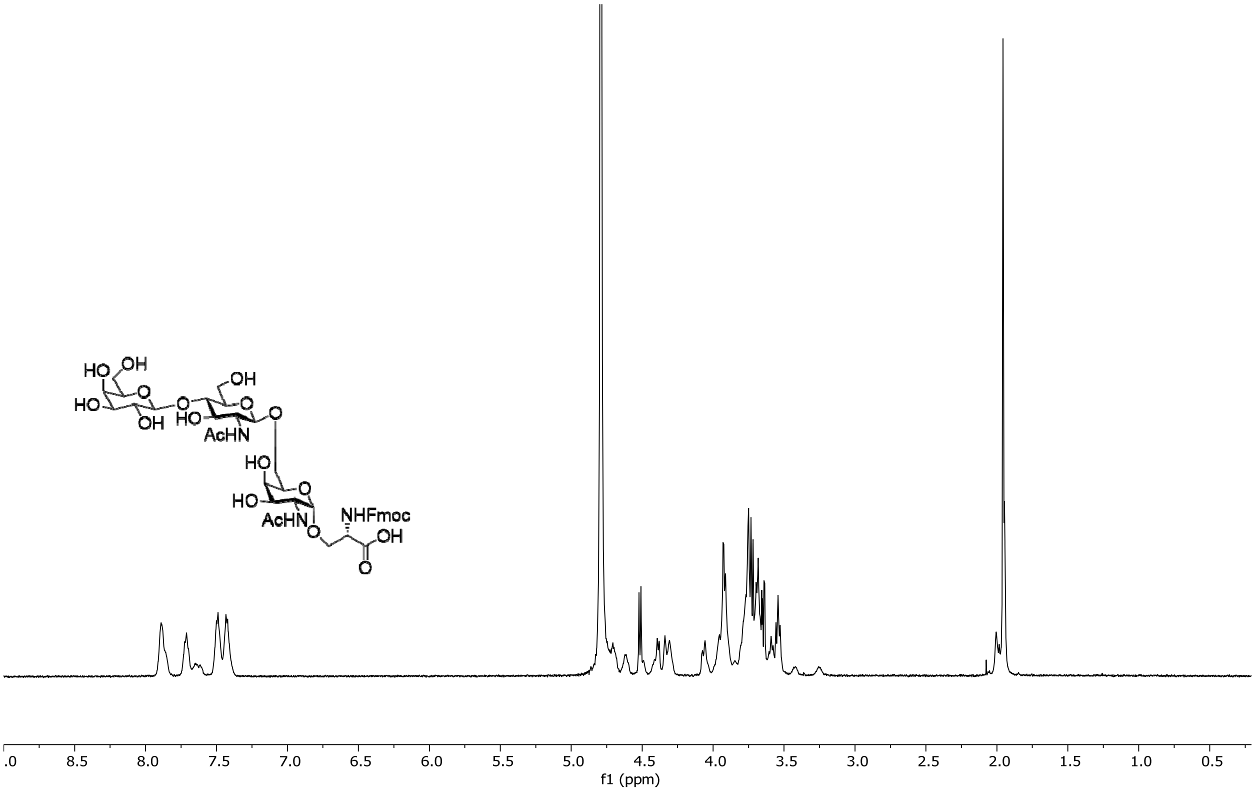

^{1}H NMR of 90

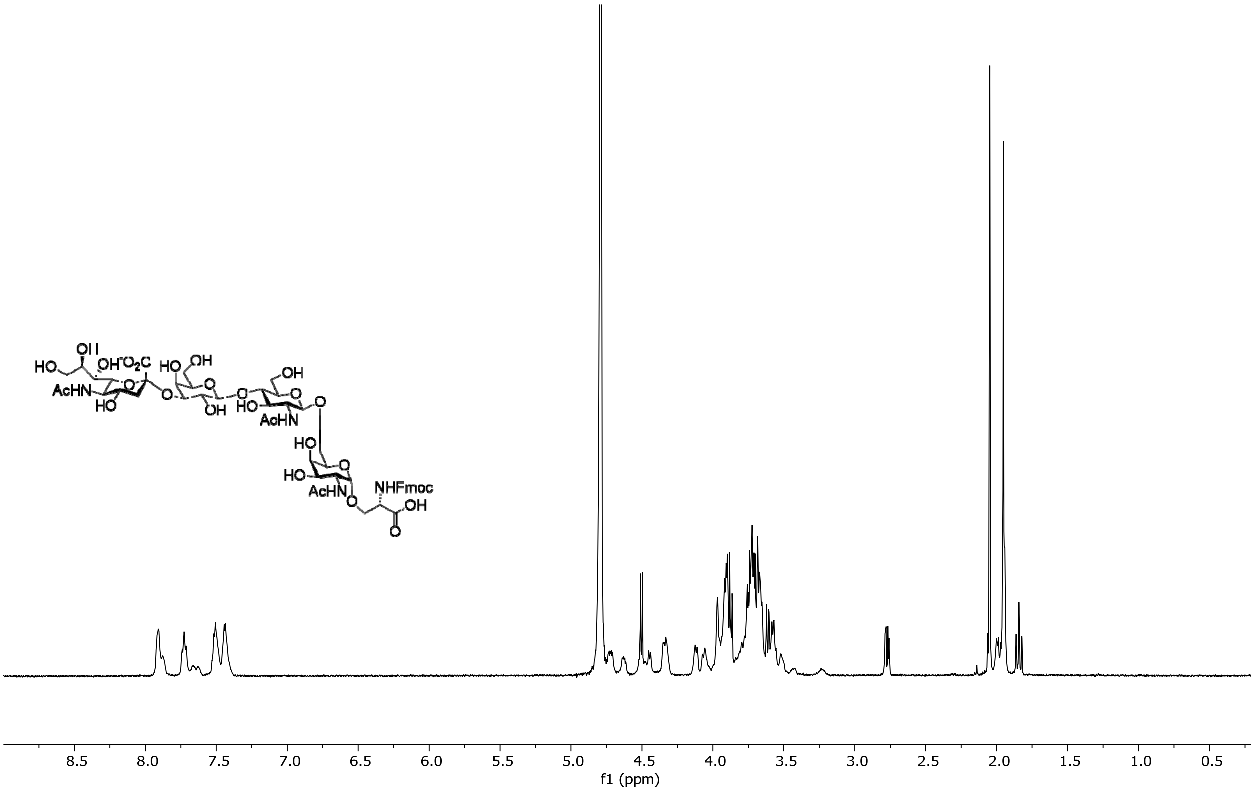

^{1}H NMR of 91

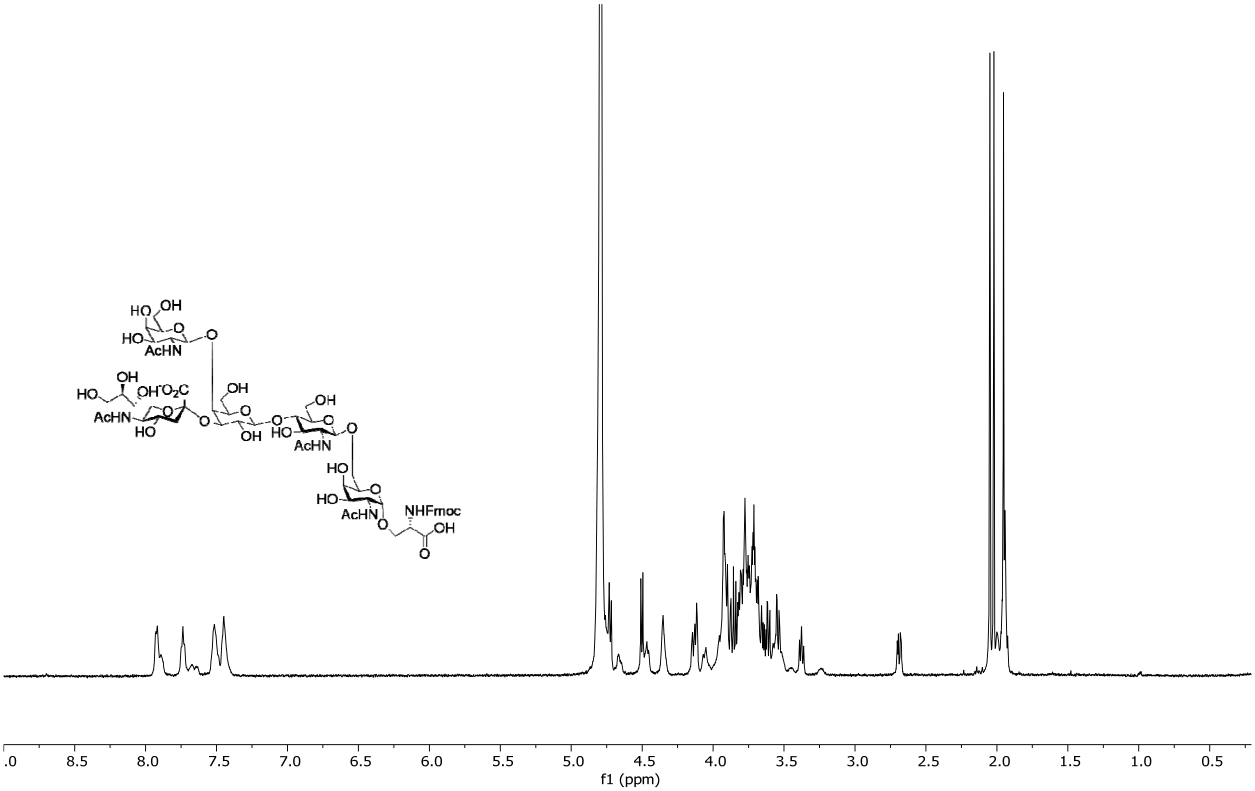

^{1}H NMR of 92

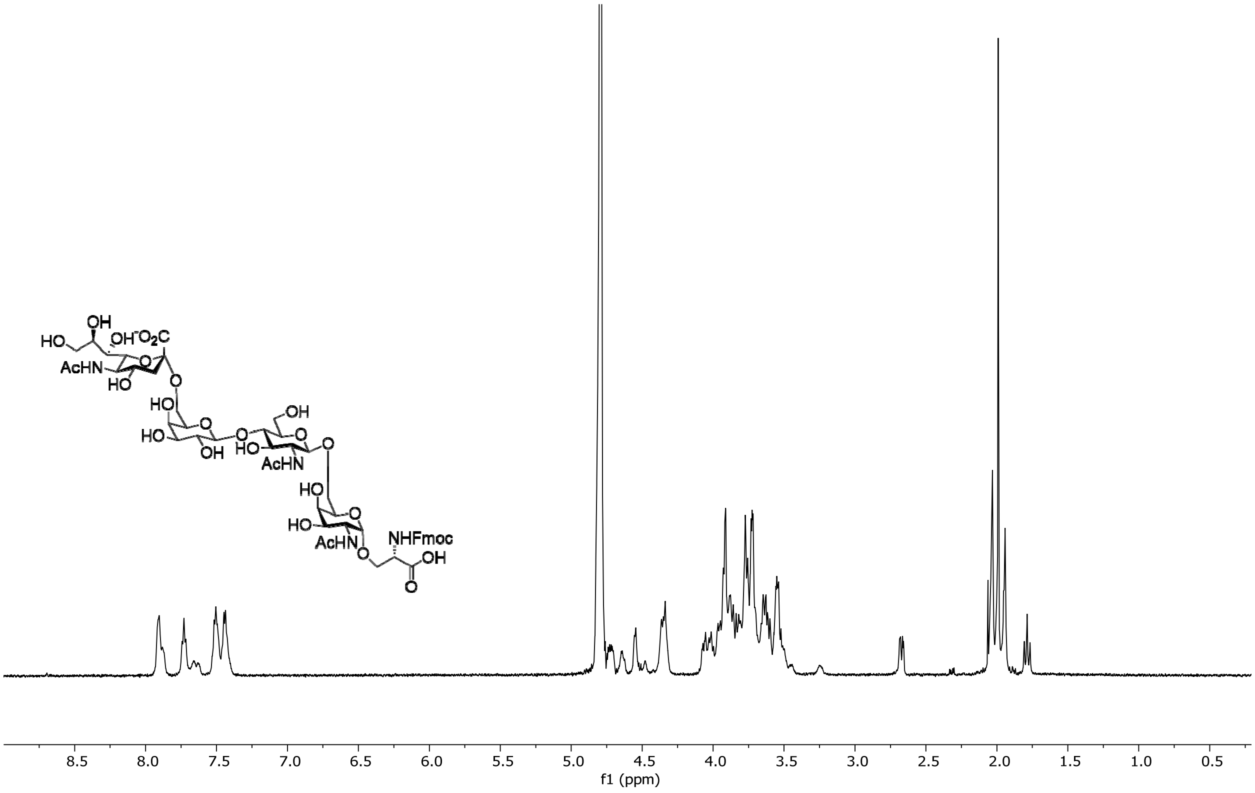

^{1}H NMR of 93

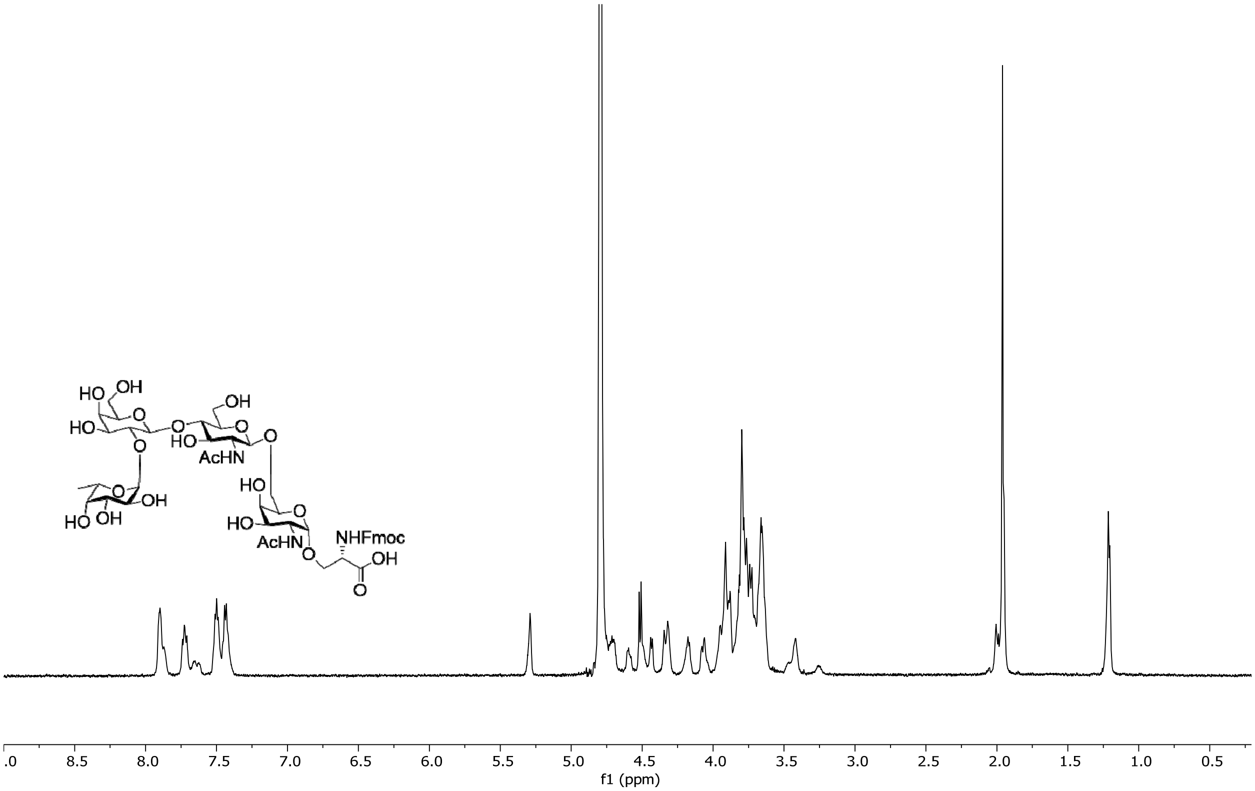

^{1}H NMR of 94

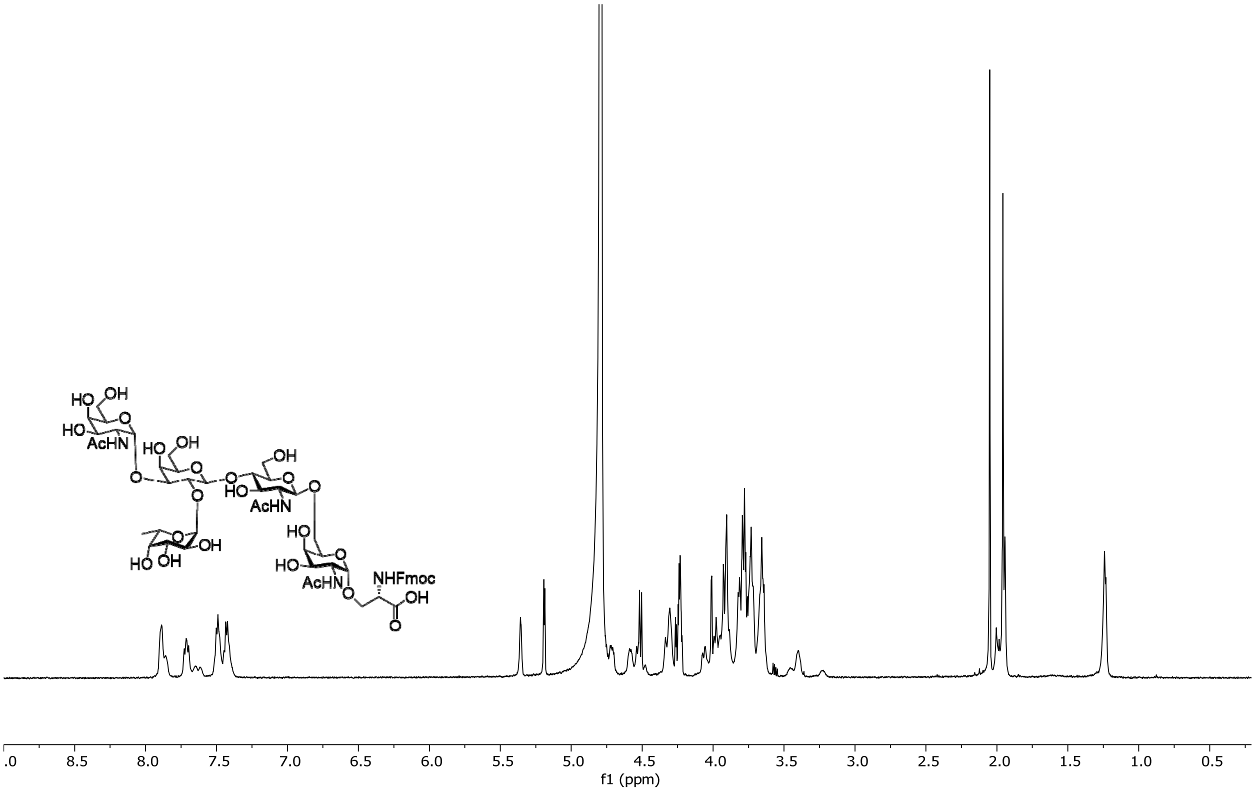

^{1}H NMR of 95

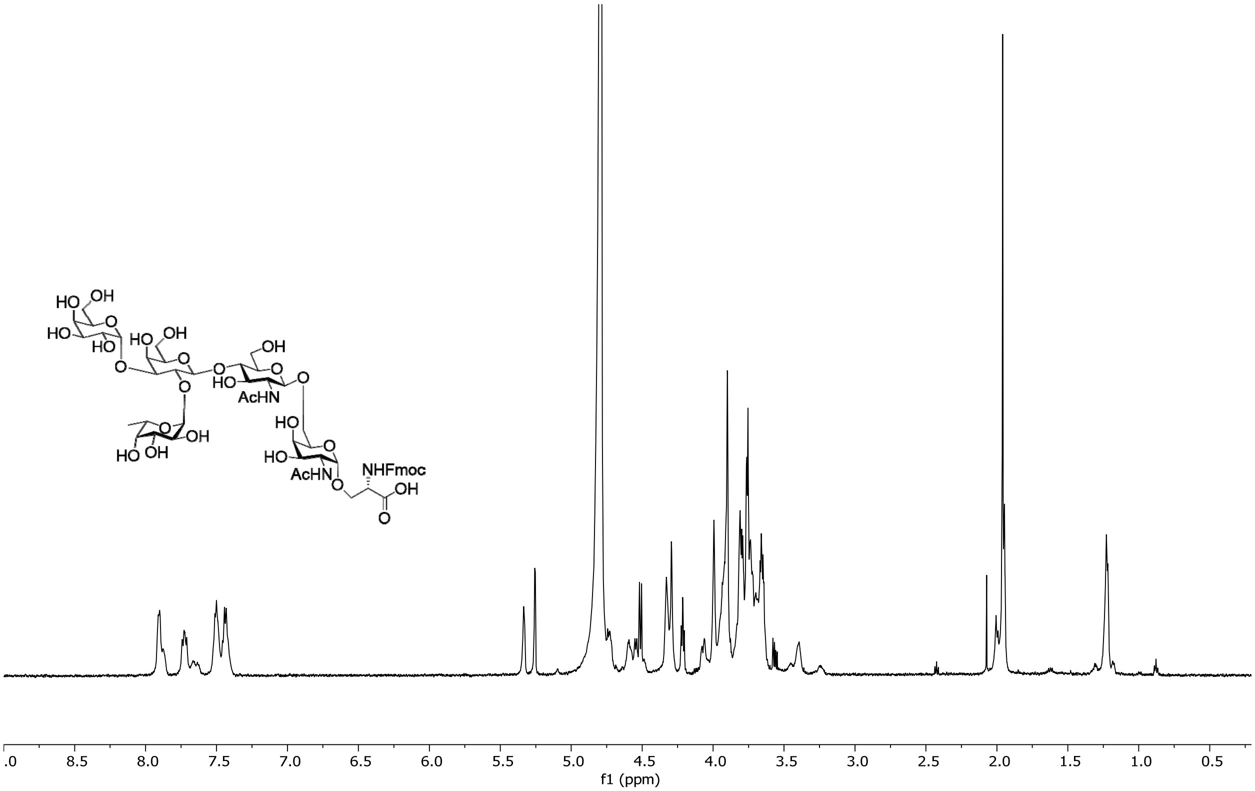

^{1}H NMR of 96

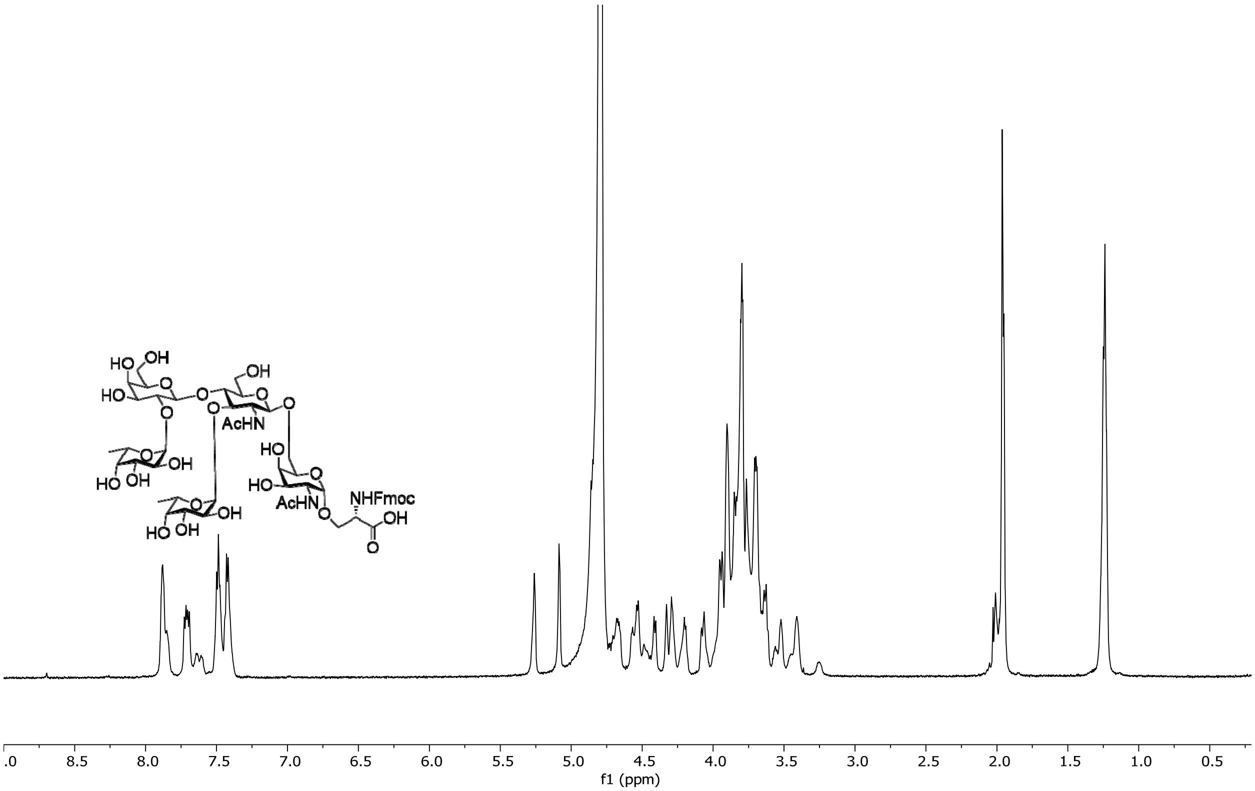

^{1}H NMR of 97

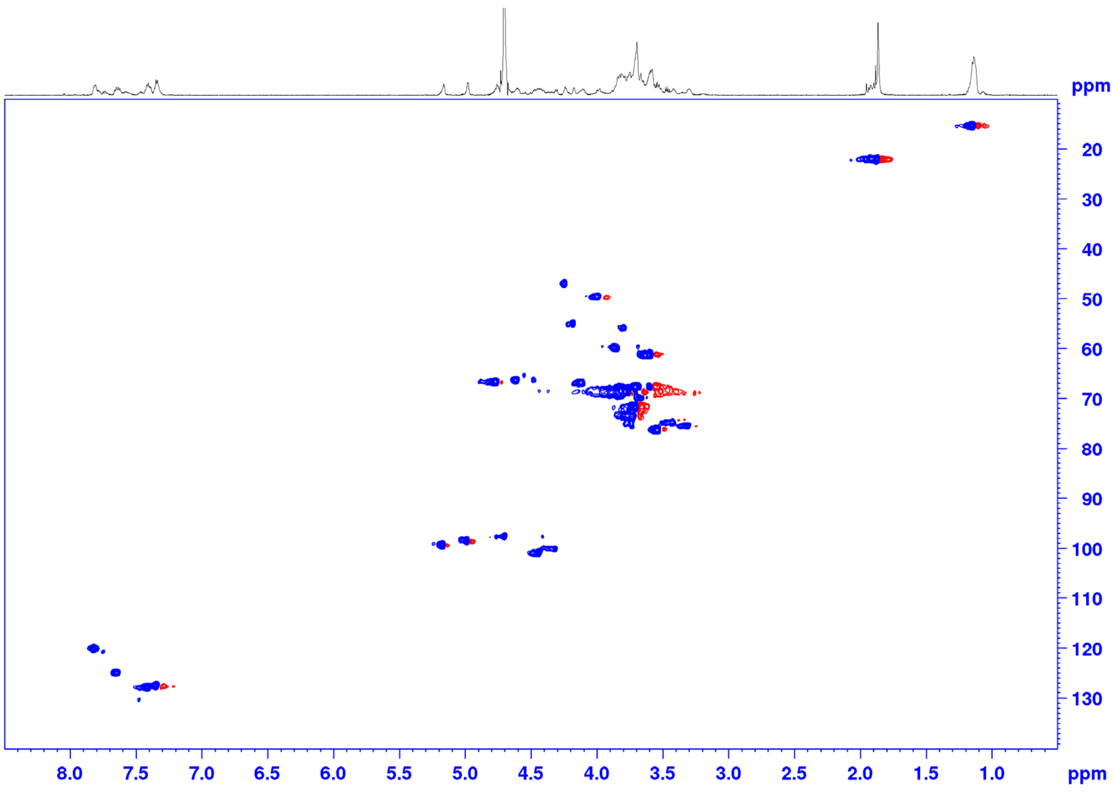

HSQC of 97

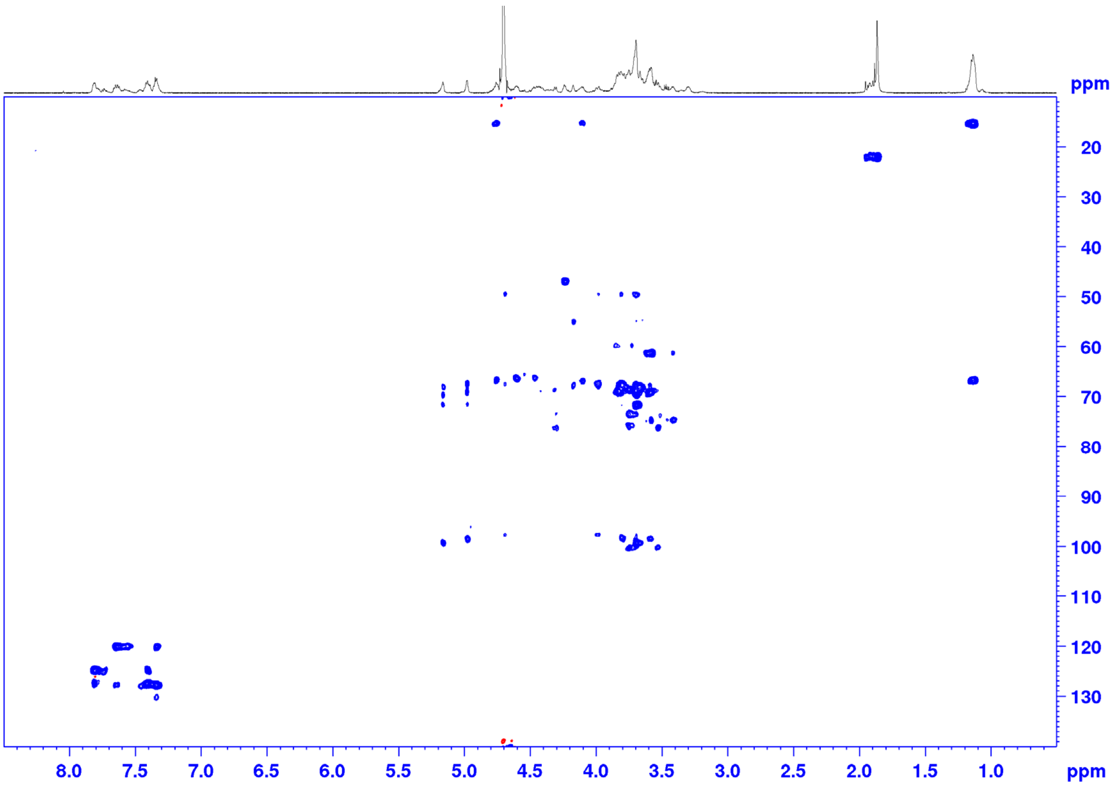

HSQC-TOCSY of 97

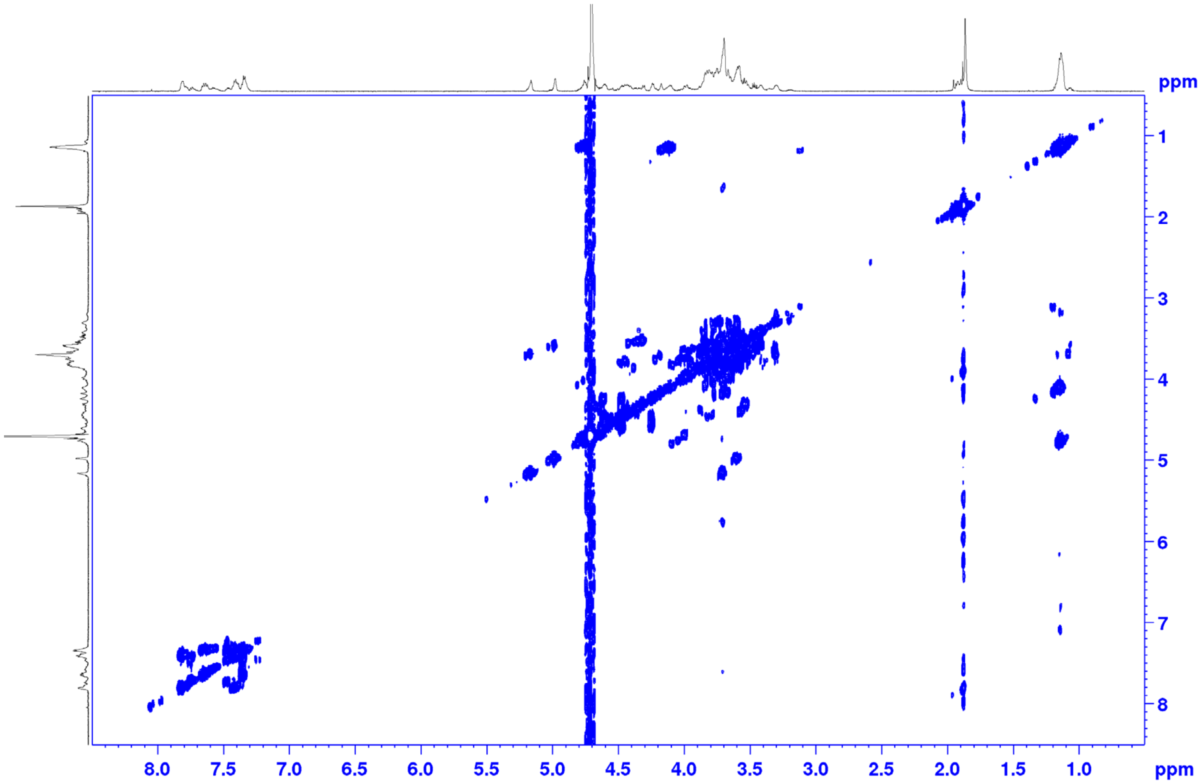

COSY of 97

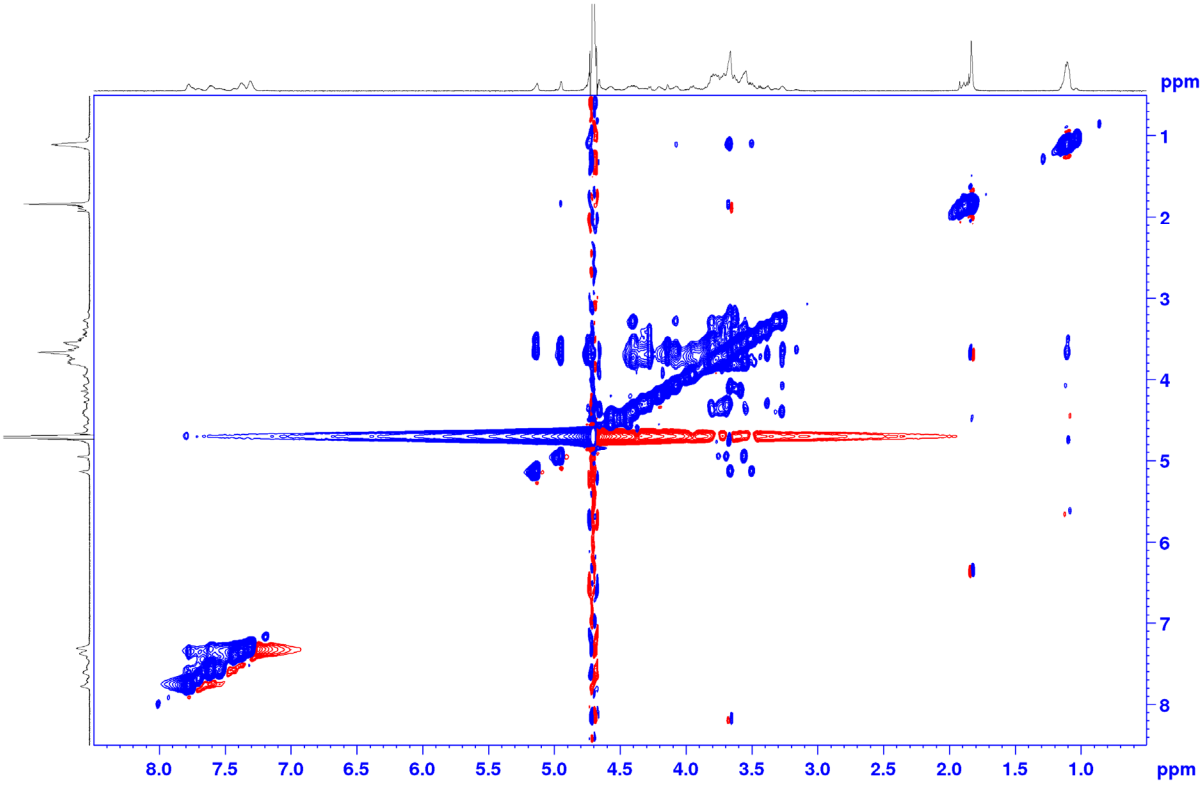

NOESY of 97

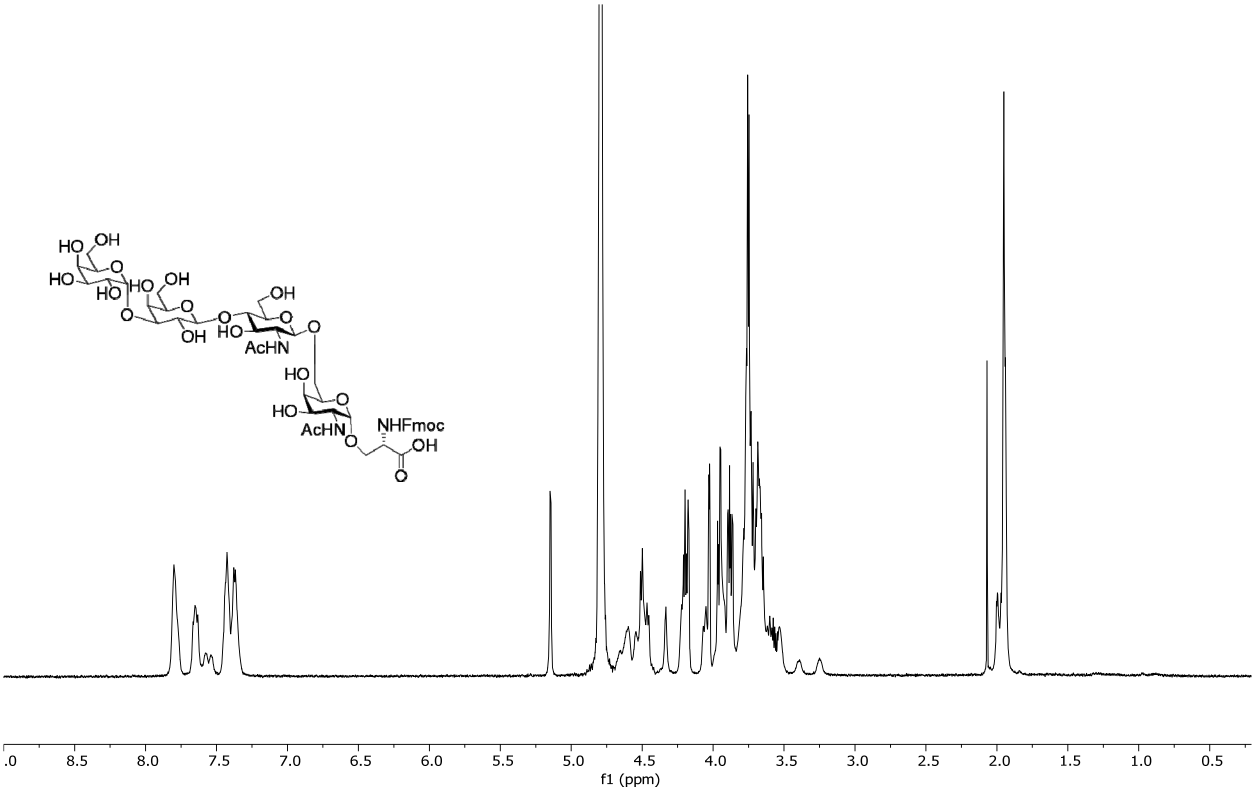

^{1}H NMR of 98

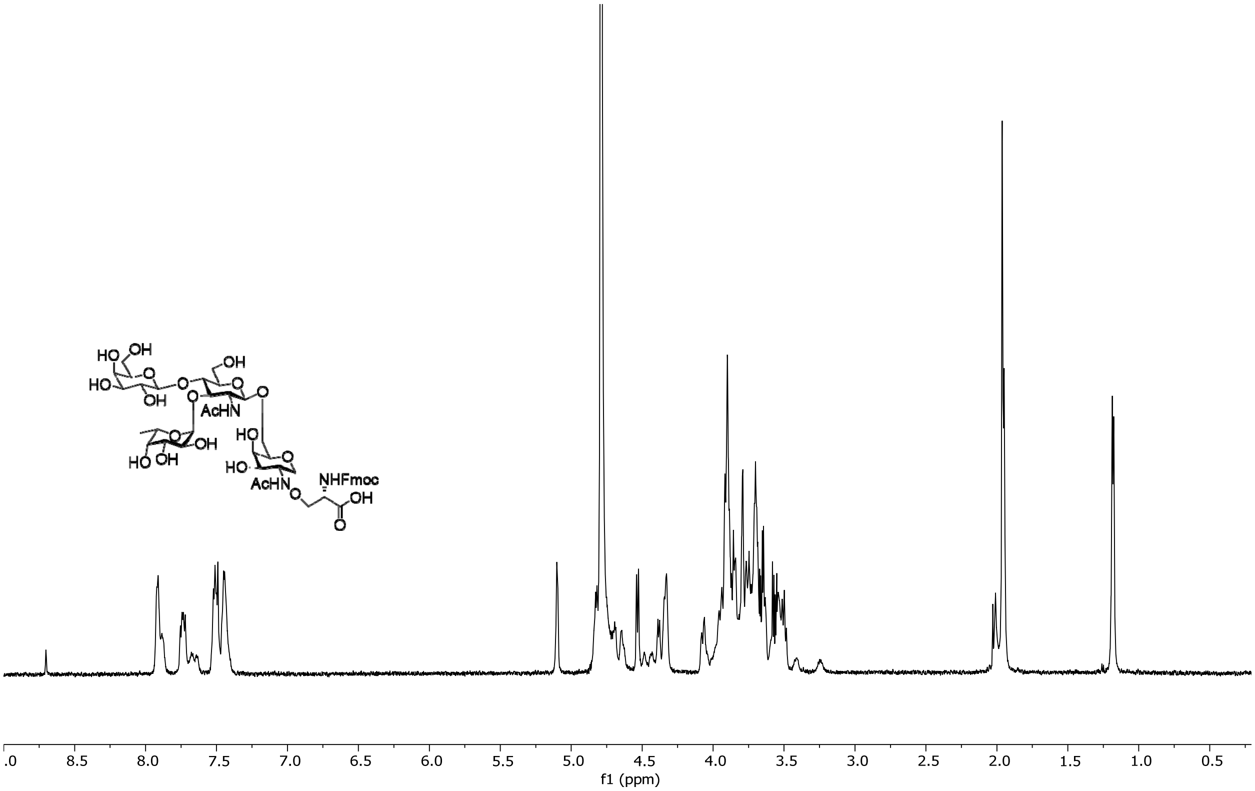

^{1}H NMR of 99

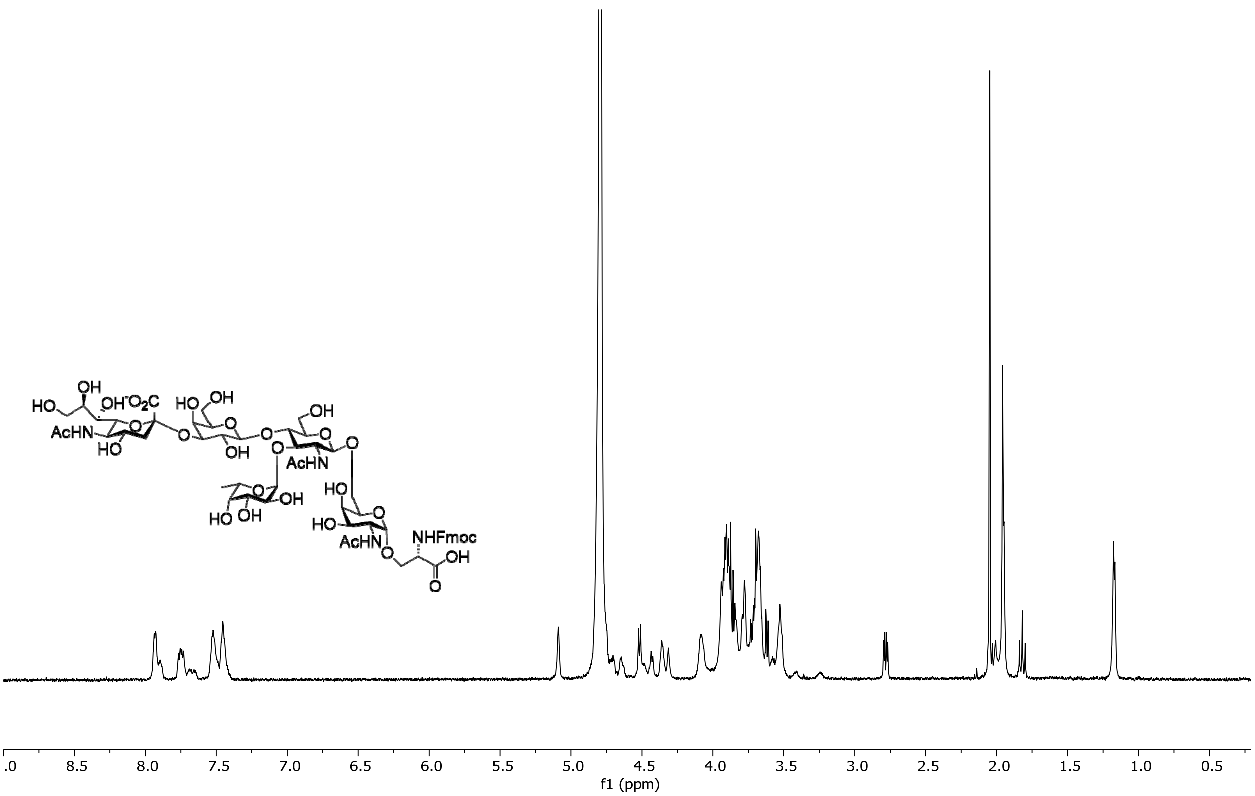

^{1}H NMR of 100

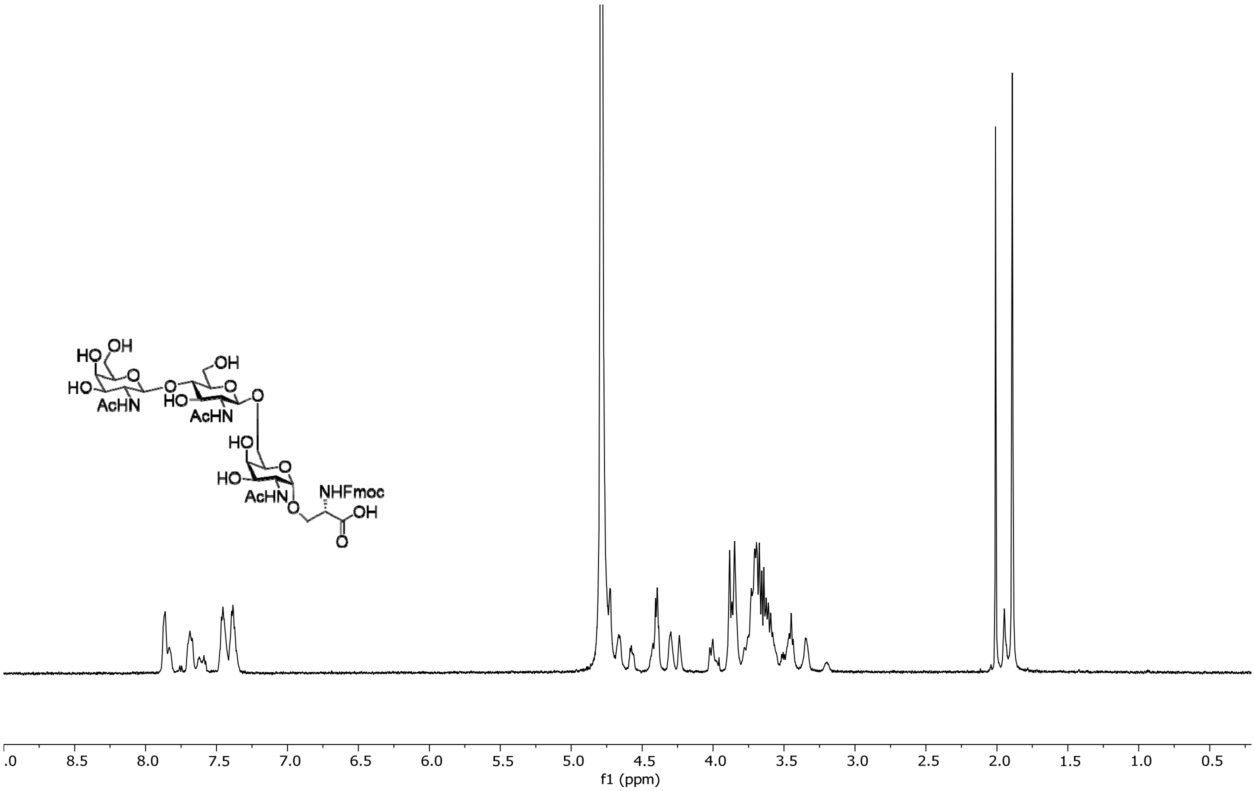

^{1}H NMR of 101

VII. Supplementary References

1. Santra A, Ghosh T, Misra AK. Expedient synthesis of two structurally close tetrasaccharides corresponding to the O-antigens of Escherichia coli O127 and Salmonella enterica O13. *Tetrahedron: Asymmetry* **23**, 1385-1392 (2012).
2. Knerr PJ, van der Donk WA. Chemical Synthesis and Biological Activity of Analogues of the Lantibiotic Epilancin 15X. *J Am Chem Soc* **134**, 7648-7651 (2012).
3. Cheng H*, et al.* Synthesis and enzyme-specific activation of carbohydrate−geldanamycin conjugates with potent anticancer activity. *J Med Chem* **48**, 645-652 (2005).
4. Paulsen H, Helpap B. Building blocks of oligosaccharides. Part XCVI. Synthesis and partial structures of the N-glycoproteins of the complex type. *Carbohydr Res* **216**, 289-313 (1991).
5. Muthana MM*, et al.* Efficient one-pot multienzyme synthesis of UDP-sugars using a promiscuous UDP-sugar pyrophosphorylase from Bifidobacterium longum (BLUSP). *Chem Commun* **48**, 2728-2730 (2012).
6. Zhao G, Guan W, Cai L, Wang PG. Enzymatic route to preparative-scale synthesis of UDP-GlcNAc/GalNAc, their analogues and GDP-fucose. *Nat Protoc* **5**, 636-646 (2010).
7. Lau K*, et al.* Highly efficient chemoenzymatic synthesis of β1–4-linked galactosides with promiscuous bacterial β1–4-galactosyltransferases. *Chem Commun* **46**, 6066-6068 (2010).
8. Ye J*, et al.* Diversity-Oriented Enzymatic Modular Assembly of ABO Histo-blood Group Antigens. *ACS Catal* **6**, 8140-8144 (2016).
9. Fang J*, et al.* Highly Efficient Chemoenzymatic Synthesis of α-Galactosyl Epitopes with a Recombinant α(1→3)-Galactosyltransferase. *J Am Chem Soc* **120**, 6635-6638 (1998).
10. Yu H, Yu H, Karpel R, Chen X. Chemoenzymatic synthesis of CMP-sialic acid derivatives by a one-pot two-enzyme system: comparison of substrate flexibility of three microbial CMP-sialic acid synthetases. *Bioorg Med Chem* **12**, 6427-6435 (2004).
11. Sugiarto G*, et al.* A Sialyltransferase Mutant with Decreased Donor Hydrolysis and Reduced Sialidase Activities for Directly Sialylating Lewisx. *ACS Chem Biol* **7**, 1232-1240 (2012).
12. McArthur JB, Yu H, Zeng J, Chen X. Converting Pasteurella multocidaalpha2-3-sialyltransferase 1 (PmST1) to a regioselective alpha2-6-sialyltransferase by saturation mutagenesis and regioselective screening. *Org Biomol Chem* **15**, 1700-1709 (2017).
13. Yu H, Huang S, Chokhawala H, Sun M, Zheng H, Chen X. Highly efficient chemoenzymatic synthesis of naturally occurring and non-natural alpha-2,6-linked sialosides: a P. damsela alpha-2,6-sialyltransferase with extremely flexible donor-substrate specificity. *Angew Chem Int Ed* **45**, 3938-3944 (2006).
14. Yu H*, et al.* Sequential One-Pot Multienzyme Chemoenzymatic Synthesis of Glycosphingolipid Glycans. *J Org Chem* **81**, 10809-10824 (2016).
15. Yi W, Shen J, Zhou G, Li J, Wang PG. Bacterial homologue of human blood group A transferase. *J Am Chem Soc* **130**, 14420-14421 (2008).
16. Li Y*, et al.* Donor substrate promiscuity of bacterial beta1-3-N-acetylglucosaminyltransferases and acceptor substrate flexibility of beta1-4-galactosyltransferases. *Bioorg Med Chem* **24**, 1696-1705 (2016).
17. Kamada N, Koizumi S. α1, 2-fucosyltransferase and DNA encoding the same. Google Patents (2007).
18. Lin SW, Yuan TM, Li JR, Lin CH. Carboxyl terminus of Helicobacter pylori alpha1,3-fucosyltransferase determines the structure and stability. *Biochem* **45**, 8108-8116 (2006).
19. Ramakrishnan B, Qasba PK. Structure-based design of beta 1,4-galactosyltransferase I (beta 4Gal-T1) with equally efficient N-acetylgalactosaminyltransferase activity: point mutation broadens beta 4Gal-T1 donor specificity. *J Biol Chem* **277**, 20833-20839 (2002).
20. Wen L*, et al.* A One-Step Chemoenzymatic Labeling Strategy for Probing Sialylated Thomsen-Friedenreich Antigen. *ACS Cent Sci* **4**, 451-457 (2018).
21. Einarsson S, Josefsson B, Lagerkvist S. Determination of amino acids with 9-fluorenylmethyl chloroformate and reversed-phase high-performance liquid chromatography. *J Chromatogr A* **282**, 609-618 (1983).
