# Supplementary material for: Chemoenzymatic modular assembly of O-GalNAc glycans for functional glycomics
Source: Nat Commun. 2021 Jun 11;12:3573. doi: 10.1038/s41467-021-23428-x (PMC8196059; doi:10.1038/s41467-021-23428-x)
Supplement: Supplementary file 2 — Reporting Summary [file 41467_2021_23428_MOESM2_ESM.pdf]

nature research

Corresponding author(s): Lei Li
Last updated by author(s): 04/16/2020

Reporting Summary

Nature Research wishes to improve the reproducibility of the work that we publish. This form provides structure for consistency and transparency in reporting. For further information on Nature Research policies, see our Editorial Policies and the Editorial Policy Checklist.

Statistics

For all statistical analyses, confirm that the following items are present in the figure legend, table legend, main text, or Methods section.

n/a	Confirmed	
☐	☒	The exact sample size (n) for each experimental group/condition, given as a discrete number and unit of measurement
☐	☒	A statement on whether measurements were taken from distinct samples or whether the same sample was measured repeatedly
☒	☐	The statistical test(s) used AND whether they are one- or two-sided<br>*Only common tests should be described solely by name; describe more complex techniques in the Methods section.*
☒	☐	A description of all covariates tested
☒	☐	A description of any assumptions or corrections, such as tests of normality and adjustment for multiple comparisons
☐	☒	A full description of the statistical parameters including central tendency (e.g. means) or other basic estimates (e.g. regression coefficient) AND variation (e.g. standard deviation) or associated estimates of uncertainty (e.g. confidence intervals)
☒	☐	For null hypothesis testing, the test statistic (e.g. F, t, r) with confidence intervals, effect sizes, degrees of freedom and P value noted<br>*Give P values as exact values whenever suitable.*
☒	☐	For Bayesian analysis, information on the choice of priors and Markov chain Monte Carlo settings
☒	☐	For hierarchical and complex designs, identification of the appropriate level for tests and full reporting of outcomes
☒	☐	Estimates of effect sizes (e.g. Cohen's d, Pearson's r), indicating how they were calculated

Our web collection on statistics for biologists contains articles on many of the points above.

Software and code

Policy information about availability of computer code

Data collection	Data from microarray was collected by the software (Genepix Pro6.1).
Data analysis	Data was analyzed by using Excel (Microsoft Office 365).

For manuscripts utilizing custom algorithms or software that are central to the research but not yet described in published literature, software must be made available to editors and reviewers. We strongly encourage code deposition in a community repository (e.g. GitHub). See the Nature Research guidelines for submitting code & software for further information.

Data

Policy information about availability of data

All manuscripts must include a data availability statement. This statement should provide the following information, where applicable:

- Accession codes, unique identifiers, or web links for publicly available datasets
- A list of figures that have associated raw data
- A description of any restrictions on data availability

The data supporting the findings of this study are available within the article and its Supplementary Information. Other relevant data are available from the corresponding author upon reasonable request. Source data are provided with this paper.

Field-specific reporting

Life sciences study design

All studies must disclose on these points even when the disclosure is negative.

Sample size	For O-GalNAc array assay, 19 glycan binding proteins (GBPs) and 58 human serum specimens (Table S5) from colorectal cancer patients and healthy people were used in this study. All related GBPs that commercially available and normally used in glycanarray analysis and enrichment are included in 19 GBPs. Human serum samples are sufficient for the assay as clearly results were observed.
Data exclusions	No data were excluded from the analysis.
Replication	Three replicates were applied for every data set and all attempts at replication were successful.
Randomization	Samples were not randomized as control group (health and cancer) were used. All serums were randomized collected.
Blinding	The investigators were not blinded as it was not relevant to this study. The assay were performed with serum samples and the aim of the assay was to investigated the difference between health and cancer group. All serums were randomized collected before the assay.

Reporting for specific materials, systems and methods

We require information from authors about some types of materials, experimental systems and methods used in many studies. Here, indicate whether each material, system or method listed is relevant to your study. If you are not sure if a list item applies to your research, read the appropriate section before selecting a response.

Materials & experimental systems

n/a	Involved in the study	
☐	☒	Antibodies
☒	☐	Eukaryotic cell lines
☒	☐	Palaeontology and archaeology
☒	☐	Animals and other organisms
☐	☒	Human research participants
☒	☐	Clinical data
☒	☐	Dual use research of concern

Methods

n/a	Involved in the study	
☒	☐	ChIP-seq
☒	☐	Flow cytometry
☒	☐	MRI-based neuroimaging

Antibodies

Antibodies used	1.Purified Mouse Anti-Human CD15s, Clone CSLEX1 (RUO)(BD Biosciences-US, #551344, 20 ug/mL)<br>2.Goat anti-Mouse IgG (H+L) Cross-Adsorbed Secondary Antibody, Alexa Fluor 647 (ThermoFisher, #A-21235, 5 μg/mL)<br>3.Sialosyl-Tn Antigen Monoclonal Antibody (STn 219) (ThermoFisher, # MA1-90577, 1:10)<br>4.MUC-1 Sheep anti-Human antibody(R&D Systems, #AF6298-SP, 1:50)<br>5.Anti-Sheep IgG (H+L), highly cross-adsorbed, CF™ 633 antibody produced in rabbit (Sigma, #SAB4600152-250UL, 5 μg/mL)<br>6.6x-His Tag Monoclonal Antibody (4E3D10H2/E3), Alexa Fluor 647 (ThermoFisher, #MA1-135-A647)<br>7.Goat anti-Human IgM Cross-Adsorbed Secondary Antibody, DyLight 550 (ThermoFisher, #SA5-10103)<br>8.Goat anti-Human IgG Fc Cross-Adsorbed Secondary Antibody, DyLight 650 (ThermoFisher, #SA5-10137)
Validation	Antibodies were commercially available with validations described on the associated company websites unless mention separately.<br>1. Purified Mouse Anti-Human CD15s, Clone CSLEX1 (RUO)(BD Biosciences-US, #551344, 20 ug/mL) [https://www.bdbiosciences.com/us/applications/research/stem-cell-research/cancer-research/human/purified-mouse-anti-human-cd15s-cslex1/p/551344]<br>2. Sialosyl-Tn Antigen Monoclonal Antibody (STn 219) (ThermoFisher, # MA1-90577, 1:10) [https://www.thermofisher.com/antibody/product/Sialosyl-Tn-Antigen-Antibody-clone-STn-219-Monoclonal/MA1-90577]<br>3. Goat anti-Mouse IgG (H+L) Cross-Adsorbed Secondary Antibody, Alexa Fluor 647 (ThermoFisher, #A-21235, 5 μg/mL) [https://www.thermofisher.com/antibody/product/Goat-anti-Mouse-IgG-H-L-Cross-Adsorbed-Secondary-Antibody-Polyclonal/A-21235]<br>4. MUC-1 Sheep anti-Human antibody(R&D Systems, #AF6298-SP, 1:50) [https://www.rndsystems.com/products/human-muc-1-antibody_af6298]<br>5. Anti-Sheep IgG (H+L), highly cross-adsorbed, CF™ 633 antibody produced in rabbit (Sigma, #SAB4600152-250UL, 5 μg/mL) [https://www.sigmaaldrich.com/catalog/product/sigma/sab4600152?lang=en&region=US]<br>6. 6x-His Tag Monoclonal Antibody (4E3D10H2/E3), Alexa Fluor 647 (ThermoFisher, #MA1-135-A647, 5 μg/mL) [https://www.rndsystems.com/products/his-tag-alexa-fluor-647-conjugated-antibody-ad1110r_ic0501r]<br>7. Goat anti-Human IgM Cross-Adsorbed Secondary Antibody, DyLight 550 (ThermoFisher, #SA5-10103, 5 μg/mL) [validate in this work]<br>8. Goat anti-Human IgG Fc Cross-Adsorbed Secondary Antibody, DyLight 650 (ThermoFisher, #SA5-10137, 5 μg/mL) [validate in previous literature, Nat Cell Biol 23, 401–412 (2021).]

Human research participants

Policy information about studies involving human research participants

Population characteristics	Table S5 lists the metadata for the patients from which the data were generated. There was no genotype information available from the participants.
Recruitment	Human serum specimens were collected with informed consent. No self-selection bias were detected.
Ethics oversight	Human serum specimens (Table S5) from colorectal cancer patients and normal people were provided by Georgia Cancer Center at Augusta University and stored at -80 °C until use. The protocol for serum specimen preparation was approved by the Institutional Review Board of Augusta University and was performed in accordance with the Helsinki Declaration. All participants gave written informed consent.

Note that full information on the approval of the study protocol must also be provided in the manuscript.
